# A pan-cancer analysis of homeobox family: expression characteristics and latent significance in prognosis and immune microenvironment

**DOI:** 10.3389/fonc.2025.1521652

**Published:** 2025-02-06

**Authors:** Yuanhui Wang, Jie Gao, Ziyi Ren, Ziyi Shen, Wei Gu, Qinyi Miao, Xiaomeng Hu, Yan Wu, Wei Liu, Jia Jia, Yi Cai, Chunpeng (Craig) Wan, Lei Sun, Tingdong Yan

**Affiliations:** ^1^ School of Life Sciences, Shanghai University, Shanghai, China; ^2^ Translational Medicine Center, Zhejiang Xinda Hospital, School of Medicine&Nursing, Huzhou University, Huzhou, China; ^3^ University and College Key Lab of Natural Product Chemistry and Application in Xinjiang, School of Chemistry and Environmental Science, Yili Normal University, Yining, China; ^4^ Key Laboratory of Molecular Target & Clinical Pharmacology and the State & National Medical Products Administration (NMPA) Key Laboratory of Respiratory Disease, School of Pharmaceutical Sciences & The Fifth Affiliated Hospital, Guangzhou Medical University, Guangzhou, China; ^5^ Jiangxi Key Laboratory for Postharvest Technology and Nondestructive Testing of Fruits and Vegetables, College of Agronomy, Jiangxi Agricultural University, Nanchang, China; ^6^ Department of Pathology, Beijing Ditan Hospital, Capital Medical University, Beijing, China

**Keywords:** Homeobox family, pan-cancer, immune microenvironment, prognosis, HOXB7, HOXC6

## Abstract

**Background:**

The Homeobox (HOX) gene family are conserved transcription factors that are essential for embryonic development, oncogenesis, and cancer suppression in biological beings. Abnormally expressed HOX genes in cancers are directly associated with prognosis.

**Methods:**

Public databases such as TCGA and the R language were used to perform pan-cancer analyses of the HOX family in terms of expression, prognosis, and immune microenvironment. The HOX score was defined, and potential target compounds in cancers were predicted by Connective Map. Immunohistochemistry was employed to validate protein expression levels. Gene knockdowns were used to verify the effects of HOXB7 and HOXC6 on the proliferation and migration of lung adenocarcinoma (LUAD) cells.

**Results:**

HOX genes play different roles in different cancers. Many HOX genes, especially HOXB7 and HOXC6, have higher expression and lower overall survival in specific cancers and are predicted as risk factors. The high expression of most HOX genes is mainly related to immune subtypes C1-C4 and C6. Potential anti-tumor compounds for down-regulating HOX gene expression were identified, such as HDAC inhibitors and tubulin inhibitors. LUAD Cell migration and proliferation were inhibited when HOXB7 or HOXC6 was knocked down.

**Conclusions:**

Many HOX genes may act as both oncogenes and tumor suppressor genes, necessitating precision medicine based on specific cancers. The HOX gene family plays a crucial role in the development of certain cancers, and their expression patterns are closely related to cancer prognosis and the tumor microenvironment (TME), which may affect cancer prognosis and response to immunotherapy. Compounds that are negatively correlated with the expression levels of the HOX family in various cancers, such as HDAC inhibitors, are potential anti-cancer drugs. HOXB7 and HOXC6 may serve as potential targets for cancer treatment and the development of targeted compounds in the future.

## 1 Introduction

The homeobox (HOX) gene family, a kind of key transcription factor family involved in embryonic development, is characterized by the presence of a highly conserved DNA sequence of 180 bp nucleotides, which encodes a 60 amino acid homeodomain that could bind to DNA in a sequence-specific way (1). The HOX genes were first found in Drosophila melanogaster, and their mutations resulted in body deformities (2). According to recent research, the HOX family in mammals contains 39 members and is classified into four clusters based on their structures, namely, HOXA, HOXB, HOXC, and HOXD (1). It could also be divided into 13 groups based on the similarity of sequence and the arrangement of genes on chromosomes, and each group of HOX genes has specific functions. The four subgroups of the human HOX genes are clustered on chromosomes 7p14, 17q21, 12q13, and 2q31, respectively (3). Besides, the genes of HOX subfamilies were amplified in evolution (4, 5), and the expression of HOX genes in the same subfamily is collinear; they are activated or silenced from the 3’ end to the 5’ end in turn according to a certain time sequence. The expression of HOX genes at the 3’ end is the earliest, expressing in the anterior tissue, while the expression of HOX genes at the 5’ end is the opposite (6, 7). That is to say, the spatial collinearity of HOX genes corresponds to their expression order along the anterior-posterior (A-P) axis in animals, and the temporal collinearity is also coordinated with the sequence of regulating the front and rear body axes of organisms during embryonic development.

Based on this special collinearity rule, the expression pattern of HOX genes in tumors has attracted wide attention and been gradually studied. Many studies have shown that HOX genes are related to a variety of cancers, including lung cancer (8–10), breast cancer (11, 12), ovarian cancer (13, 14), gastric cancer (15–17), prostate cancer (18), pancreatic cancer (19) and so on, and that they are closely linked to tumor stage, tumor occurrence location, and prognosis (20, 21). Initially, it was considered that HOX gene expression was only up-regulated in tumor tissues (22), but subsequent studies revealed that the HOX gene could be employed as a tumor promoter or inhibitor (23, 24). HOX genes that play a role in tumor inhibition are usually inhibited, but these HOX genes are abnormally expressed in other tissues, promoting the emergence and development of cancers. HOXB9, for example, can inhibit the proliferation, migration, and invasion of gastric cancer cells, induce MET transformation, and play an anti-cancer role (25); however, in pancreatic cancer, HOXB9 blocks the cell cycle process via the DNMT1/RBL2/c-Myc axis to inhibit pancreatic cancer cell proliferation (19).

The tumor microenvironment (TME) is composed of a heterogeneous population that includes cancer cells themselves, infiltrating immune cells, and stromal cells such as fibroblasts. Within this complex ecosystem, immune cells play a pivotal role in the initiation and progression of cancer. However, as immune cells with tumor-fighting capabilities target and eliminate cancer cells, these malignant cells often employ various mechanisms to evade immune surveillance (26). Concurrently, this ability to escape immune detection has paved the way for new therapeutic directions in cancer treatment, focusing on harnessing the TME’s immune cells to specifically recognize and attack cancer cells (27). The application of immune checkpoint modulators, such as anti-CTLA-4 and anti-PD-1 antibodies, along with adoptive immune cell therapies like CAR-T cells, has demonstrated unexpected anti-tumor effects across a spectrum of cancers, ushering in a new era for cancer treatment (28). Additionally, immunotherapy is not only influenced by the microenvironment but can also actively reshape its composition. In summary, uncovering the complex interplay between cancer and the immune microenvironment is of great significance for understanding the onset, progression of cancer, and the selection of therapeutic strategies.

Normal HOX gene expression is a vital part of organismal development, and when some HOX genes are abnormally over- or under-expressed, resulting in wrong cell differentiation, they may become regulatory factors for tumor formation or inhibition. These abnormal high- or low-expression HOX genes are often regulated by some special mechanisms, such as epigenetic modification such as hypermethylation of the CpG island promoter (29, 30), which further promotes the occurrence and progress of cancer. At present, the research on the association between HOX genes and cancers mostly focuses on the exploration of mechanisms, and systematic bioinformatics analyses on the expression and prognosis differences in different cancers are lacking. Therefore, it is of great value to analyze the expression and prognosis of HOX genes in pan-cancer, as well as the link with the tumor microenvironment and potential therapeutic targets. This study systematically evaluated the prognostic correlation and immune microenvironmental impact of HOX gene family expression. In lung adenocarcinoma cell lines, HOXB7 and HOXC6 were confirmed to markedly influence cell proliferation and migration, suggesting consideration as prognostic indicators. Importantly, the introduction of the HOX score to predict compounds that modulate HOX expression and pathways suggested a novel therapeutic approach targeting HOX genes in cancer treatment.

## 2 Materials and methods

### 2.1 Genetic alteration analysis

By using the cBioportal database (31) in April 2023 (https://www.cbioportal.org/), we inquired about the mutations of HOX family across 32 cancer studies (TCGA, Pan-Cancer Atlas), containing 10,967 samples total. Among them, mutation data, structural variants, and putative copy-number alterations (CNA) from GISTIC were chosen for genomic profiles, and the final result was displayed through the Oncoprint plate (showing the ‘study of origin’ and mutation of each HOX family gene). In addition, the Cancer Type Summary section showed the summary statistics of mutations in pan-cancer. Genetic alterations defined in cBioPortal are ‘Inframe Mutation’, ‘Missense Mutation’, ‘Splice Mutation’, ‘Truncating Mutation’, ‘Structural Variant’, ‘Amplification’, and ‘Deep Deletion’. Different colors in Figures were used to indicate each of the 32 pan-cancer studies or genetic alterations.

### 2.2 Expression analysis of HOX family

All cancers’ HTSeq-FPKM gene expression RNAseq data from the UCSC Xena were downloaded for pan-cancer expression analysis and correlation analysis (https://xenabrowser.net/datapages/). All data has been log_2_(FPKM+1) converted. Pan-cancer expression heatmaps are plotted by the R package ‘pheatmap’, and it also performs clustering and column normalization, only showing cancers with normal samples of not less than 5. Besides, the correlation matrix was calculated by Spearman’s test and the 'cor.test' function in R package ‘corrplot’ was utilized to evaluate the statistical significance of the correlation between HOX genes.

### 2.3 Univariate cox regression analysis and survival analysis

Survival data for 33 cancer types was also downloaded from the UCSC Xena. Using the median expression of each gene as the cutoff value, the high and low expression groups of HOX genes were compared through Kaplan-Meier survival curve analysis, including overall survival (OS), disease-specific survival (DSS), progression-free interval (PFI). The proportional hazards assumption was tested using the ‘survival’ package, followed by the fitting of a univariate Cox regression model. The hazard ratios (HR) derived from univariate Cox regression analysis are graphically represented through a dot plot, facilitating a more nuanced interpretation of the relative risks associated with each HOX gene. The survival analysis was carried out by Cox proportional hazards regression using the function coxph() from the ‘survival’ package. Kaplan-Meier survival plots with p-values and HRs were visualized using the ‘survminer’ and ‘ggplot2’ packages. Only survival curves with p-values < 0.05 could be visualized.

### 2.4 Correlation analysis between HOX and tumor microenvironment

Data relevant to immune subtypes, tumor mutation burden (VarScan2 Variant Aggregation and Masking from Somatic Mutation), and tumor stemness were downloaded from the UCSC Xena for further research (https://xenabrowser.net/datapages/). Six immune subtypes from the TCGA pan-cancer database were employed for calculating correlations with HOX family expression levels by Kruskal-Wallis test. The R package ‘estimate’ was used to examine the stromal score, immune score, and estimate score in the immune microenvironment, which can reflect the stromal and immune cell infiltration levels of each cancer. Additionally, we compute the Spearman correlation between HOX expression and these scores, and the outcomes are shown as a heatmap. Additionally, a perl script was used to generate the tumor mutation burden score (TMB) for each sample. Based on somatic mutation data obtained from TCGA (https://tcga.xenahubs.net), Microsatellite instability (MSI) scores were calculated for each sample. The Spearman correlation between TMB, MSI, DNA stemness score (DNAss), RNA stemness score (RNAss), and HOX expression were also examined. The radar chart of the Xiantao tool (https://www.xiantaozi.com/) shows the relationship between TMB, MSI, and the expression of HOXB7 and HOXC6. The correlation between the expression levels of HOX genes and the infiltration levels of 22 kinds of immune cells in pan-cancer were evaluated by ‘CIBERSORT.R’ and R-package 'ggplot2', ‘ggpubr’ and ‘ggExtra’. The correlation analysis of TMB, MSI, DNAss, and RNAss with gene expression was all conducted using the Spearman correlation test. The P-values were generated by the cor.test function to represent statistical significance.

### 2.5 The HOX score and connectivity map analysis

The HOX score was calculated by Z-normalized expression for the 39 HOX genes across all of the samples within each cancer type. The HOX score per sample was determined by calculating the mean value across the 39 HOX genes, which represents a relative and overall estimate of the HOX family. In light of the previous results, we next calculate the spearman correlation between HOX score and mRNA expression of all coding genes in each sample. For each cancer type, we identified the top 150 most positively and negatively correlated protein-coding genes with valid gene symbols in the database, which were then curated as input for the “Query” module of the Connectivity Map 1.0 (http://clue.io) (32). Query parameters were set “gene expression(L1000)”. Subsequently, the most relevant compounds (type: cp) for each of the 33 cancers were obtained. Finally, compounds with enrichment scores > 90 or < -90 in at least eight cancer types were selected for visualization to elucidate their potential therapeutic relevance.

### 2.6 Protein–protein interaction network construction and functional enrichment

The STRING online database (https://string-db.org/) was used to build the protein-protein interaction (PPI) network (33). Gene symbol names (HOXB7 and HOXC6) were subjected to PPI analysis. The minimum interaction score was set to medium confidence (0.400), and max number of interactors to show of 1st and 2st shell were all no more than 60 and 70 interactors. Next, we output the analysis results to a TSV format file, chose top 100 interacting proteins, and they were imported into Cytoscape v3.7.2 for detailed processing and visualization (34). The cytoHubba plugin was used to perform network topology analysis and node centrality analysis to find its hub genes and sub-networks. Functional enrichment analysis including Gene ontology (GO) and Kyoto Encyclopedia of Genes and Genomes (KEGG) were performed using the R-packages ‘limma’, ‘org.Hs.eg.db’, ‘clusterProfiler’ and ‘enrichplot’ based on the top 100 node genes.

### 2.7 Immunohistochemistry

Three lung adenocarcinoma cases and corresponding adjacent normal tissues collected from the Department of Pathology, Beijing Ditan Hospital, Capital Medical University were used to verify the expression of HOXB7 and HOXC6 by using immunohistochemical staining (IHC), respectively. All the images were taken under a 20x microscope. The slides performed a series of procedures, including deparaffinization, antigen retrieval solution for antigen restoration, 3% hydrogen peroxide to suppress endogenous peroxidase activity, and goat serum to minimize non-specific staining. Subsequently, HOXB7 rabbit polyclonal antibody (bs-17364R, 1:100, Bioss Antibodies, USA) and HOXC6 (PA4010, 1:100, Abmart, China) covered the slides respectively, and they were left at 4°C for the whole night. The next day, sheep anti-rabbit IgG polymer (PV-6000, Zhongshan Jinqiao Biotechnology Company, Beijing, China) was added for 30 minutes at room temperature, and then DAB was added for three 3 minutes. Finally, they were counterstained with hematoxylin, dehydrated and sealed with neutral glue.

### 2.8 Cell culture

The human embryonic kidney cell line 293T (HEK293T), the human lung cancer A549 and NCI-H1975 cell lines originated from American Type Culture Collection (ATCC). Both A549 and NCI-H1975 are human lung adenocarcinoma cells. 293T and A549 were all cultured in DMEM (HyClone, Logan, UT, USA) supplemented with 10% Fetal Bovine Serum (FBS; ExCell Bio, China) and 1% Penicillin-Streptomycin (Yuchun Bio, Shanghai, China) at 37°C and 5% CO_2_. The NCI-H1975 cell line were maintained in RPMI-1640 (ZETA life, USA) containing the same 10% FBS and 1% Penicillin-Streptomycin.

### 2.9 Construction of shRNA stably transfected cell line

All target shRNA were designed by GPP Web Portal of Broad institute (https://portals.broadinstitute.org/gpp/public/gene/search_clones). The oligonucleotide primers were annealed and connected to the pLKO.1 vector to produce the final plasmid expressing the target shRNA. According to the manufacturer’s protocol, 293T cells were used for lentivirus packaging of shRNA using Lipofectamine™ 2000 reagent (Invitrogen, USA). Next, A549 or NCI-H1975 cells were infected with packaged lentivirus, and a stable A549 or NCI-H1975 cell line containing shRNA was constructed after 1-2 weeks of puromycin screening. Oligo Sequences of shRNA were: shHOXB7:5′-CCGGCCTCACGGAAAGACAGATCAACTCGAGTTGATCTGTCTTTCCGTGAGGTTTTTG-3′; shHOXC6:5′CCGGGTGAGGCATTTCTCGACCTATCTCGAGATAGGTCGAGAAATGCCTCACTTTTTG-3′; shControl: 5’-CCGGGTTCTTGCGATTGTCTCTATTCTCGAGAATAGAGACAATCGCAAGAACTTTTTG-3’.

### 2.10 Quantitative RT-PCR

Total RNA was extracted using Trizol and One step qRT-PCR SYBR Green Kit (Vazyme Biotech Co., Nanjing, China) was used for Quantitative RT-PCR (qRT-PCR) assay. Ct values detected by Bio-Rad qRT-PCR were processed using GAPDH as an internal parameter. The 2^-ΔΔCt^ approach was employed to calculate the comparative expression. GraphPad Prism (9.0.0 version) was used for visualization. Primers used were shown as follows: GAPDH-F: 5′-CATGTTCGTCATGGGTGTGAACCA-3’, GAPDH-R: 5′-ATGGCATGGACTGTGGTCATGAGT-3’; HOXB7-F: 5′-TCCACATTACCGGGAGCC-3’, HOXB7-R: 5′-CTGGGAGCACTCTGGACG-3’; HOXC6-F: 5′-TGACATCTGGCTTGCGATTG-3’; HOXC6-R: 5′-GGCCCTCCAATCCGTCAG-3’.

### 2.11 CCK8, colony formation assay and wound healing assay

The shRNA-transfected A549 or NCI-H1975 cells were fully digested, inoculated into 96-well culture plates at 2 x 10^3^ cells per well and incubated at 37°C for different days. The original culture medium was removed and replaced with 100 µL of fresh culture medium and 10 µL of working solution containing CCK-8 working solution (TargetMol, USA) in each well. Then, the cells were incubated at 37°C for 2 h and the absorbance was measured using the CCK-8 at 450 nm on days 1, 3, 5 and 7, respectively. A total of 2 x 10^3^ transfected control, HOXB7 or HOXC6 shRNA-transfected A549 or NCI-H1975 cells were positioned on a new 6-well plate and cultured for 2 weeks as described above, during which times the medium was changed every 3 days. Formed colonies were fixed in 4% paraformaldehyde for 20 minutes, washed with PBS, and stained with Crystalline Violet (Beyotime, Shanghai, China) for 15-20 minutes. Finally, washed and photographed. Evenly transfected A549 or NCI-H1975 cells were inoculated in 6-well plates at 6 x 10^5^ cells per well for wound healing assay. After the cells completely adhered to the 6-well plates, a straight line was drawn vertically in the center of each well with a sterile 20μL pipette tip to ensure that the cells on this line were scraped off, which was regarded as 0 hour and marked it. After washing off the cell debris with PBS, the cells were incubated for 48 h. The migration of the cells was observed at 0 h, 24 h and 48 h, respectively.

### 2.12 Statistical analysis

The statistical software R (v4.2.1) was applied to perform the statistical analysis. P-value < 0.05 is deemed statistically significant. *P<0.05; **P<0.01; ***P<0.001. The Wilcoxon test was applied to investigate HOX expression according to the TCGA database. All survival analyses in this study were conducted using the Cox proportional hazard regression model. The qRT-PCR and CCK-8 assays employed the Student’s t-test to evaluate the statistical significance of differences between two groups. Preprocessing of expression data and calculation of TMB were analyzed by Perl (v5.30.0).

## 3 Results

### 3.1 Somatic alteration characteristics of HOX family in pan-cancer

**Figure 1:**
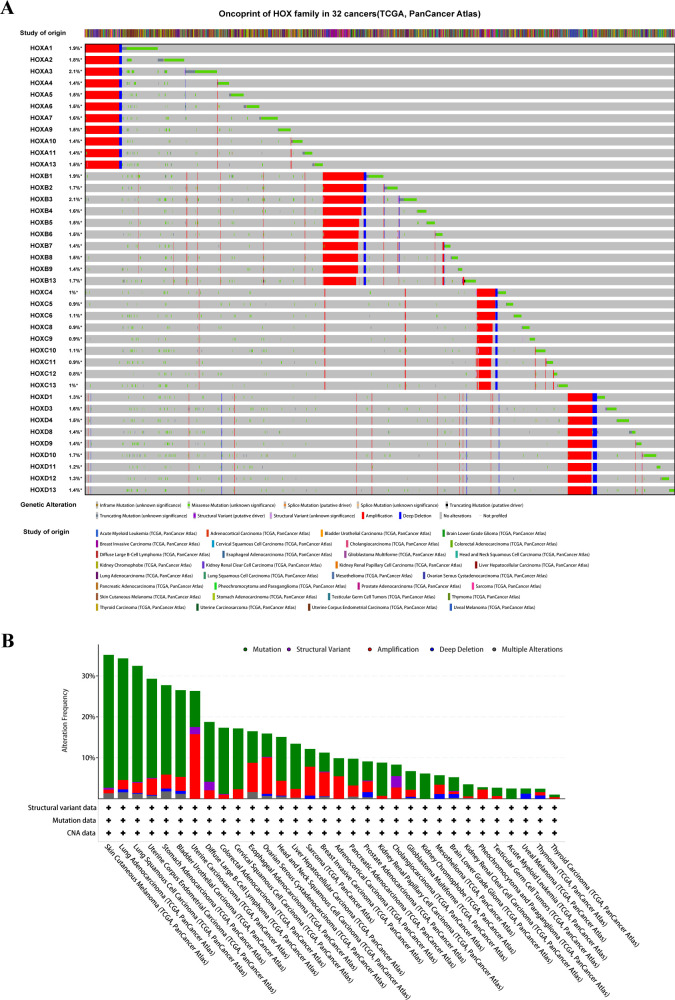
The Pan-Cancer Genome Landscape of the homeobox family (HOX). **(A)** The oncoprint of HOX family in 32 cancer types from TCGA showing the comprehensive genetic alternations. **(B)** The barplot is used to display the data on the alteration frequency of mutation and copy number alternations data of HOX gene family in 32 cancer types.

In order to explore the mutation of the HOX genes in pan-cancer, we used the cBioPortal web tool (31) to analyze the mutation of the whole HOX gene family. The oncoprint shows that the frequency of genetic changes in the HOX family in 32 cancer studies ranged from 0.8% to 2.1% (**Figure 1A**), with relatively few mutations in the whole genome. The mutation types of all HOX genes are similar, but the main mutation types in different cancers are different. There is a conservative mutation pattern in the same HOX subfamily, in which HOXA genes have the greatest mutations in pan-cancer and HOXC genes have the fewest mutations. This conservative mutation pattern may be linked to sequence similarity during evolution (4, 6). For the HOX family, among the 32 cancers, the most common genetic alterations are amplification, missense mutation, and deep deletion. Gene amplification occupied the largest proportion, followed by missense mutations. The alteration frequency of the HOX genes in patients with Skin cutaneous melanoma (SKCM) is highest across 32 cancers, and the majority of the gene alterations are mutations, followed by amplification (**Figure 1B**). In addition, we also predicted the mutation sites and types of each HOX gene in pan-cancer samples (**Supplementary Figure S1**).

### 3.2 Correlation between HOX gene expression and pan-cancer

**Figure 2:**
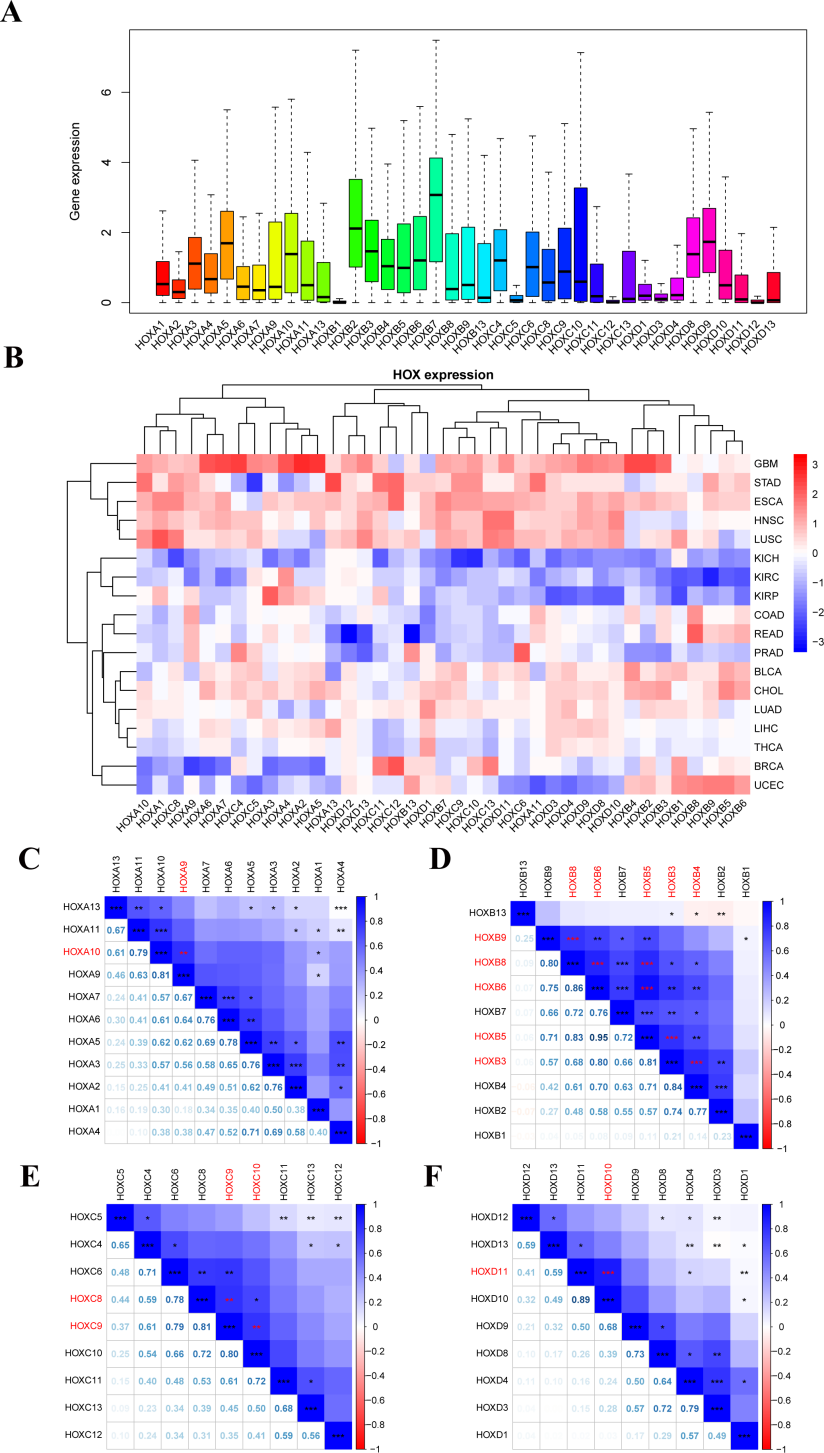
Human pan-cancer has abnormal expression of the 39 HOX genes. **(A)** Boxplot displaying the HOXs expression only in tumor tissues across all 33 different cancer types. **(B)** Heatmap showing the difference in HOXs expression between the primary tumor and normal tissues of 18 cancer types with more than five normal samples. **(C–F)** Spearman correlation plots between HOX gene expression in all 33 cancer types. The positive correlation coefficients are represented in blue and negative correlation coefficients are represented in red color. The numbers in the lower left part represent the correlation coefficients, and the color intensity and circle size in the upper right part are also proportional to the correlation coefficients. *P<0.05; **P<0.01; ***P<0.001.

We examined the overall expression of HOX genes in 33 cancer types from UCSC Xena database (**Figure 2A**) and discovered that there were obvious differences in the expression of each HOX gene in tumor samples, with the average expression levels of HOXA5, HOXA10, HOXB2, HOXB3, HOXB7, HOXC4, HOXD8, and HOXD9 being higher in pan-cancer samples, indicating that they might play an indispensable role. However, other genes, such as HOXB1, HOXC5, HOXC12, HOXD3, and HOXD12, have extremely low expression levels, suggesting that they are more expressed in normal tissue samples than in tumor samples. Excluding cancer types with less than five normal tissue samples, we analyzed the difference in expression of 39 HOX genes in 18 cancers between primary tumors and normal tissues with clustering (**Figure 2B**). The expression of most HOX genes shows tumor heterogeneity among different cancer types. For example, in GBM, STAD, ESCA, HNSC, LUSC, LUAD, LIHC, and other tumors, most HOX genes are up-regulated in primary tumor samples, whereas in KICH, KIRC, and KIRP, the expression is opposite (p<0.05, **Figure 2B**).

Among them, the up-regulation of HOX gene expression in glioma (GBM) is particularly obvious, indicating that the high expression of HOX genes may be closely related to the occurrence of GBM. Furthermore, HOX genes differ in expression, meaning they are up- or down-regulated in various tumors. We also looked at the Pearson correlation between 39 HOX genes to see how they related to one another. The results showed that some HOX genes in pan-cancer had significant strong correlation (R≥0.8; **Figures 2C–F**): HOXA9 with HOXA10, HOXB5 with HOXB6 and HOXB8, HOXB8 with HOXB6 and HOXB9, HOXB3 with HOXB4 and HOXB5 and HOXB6, HOXC8 with HOXC9, HOXC9 with HOXC10, HOXD10 with HOXD11. In addition, there are many HOX gene pairs with strong correlation (0.5<R ≤ 0.8). The majority of these gene pairs with strong correlation come from the same subfamily, they may have the same or similar functional characteristics, and may interact and play a synergistic role in the occurrence and progression of cancer.

### 3.3 Differential expression of HOX genes in pan-cancer

**Figure 3:**
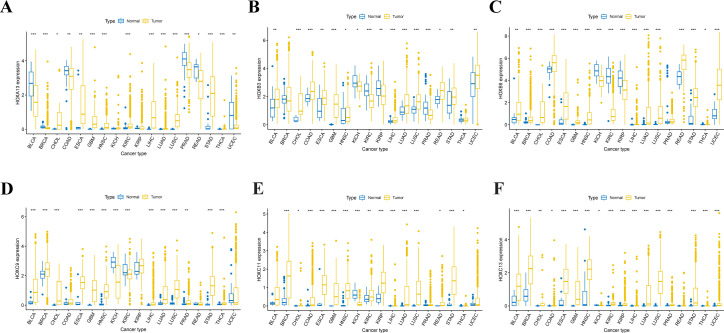
Expression level distribution of HOX gene family in primary tumor tissues and normal tissues across 18 cancer types which have more than five normal tissue samples. Boxplots showing the top 6 genes of HOXA13 **(A)**, HOXB3 **(B)**, HOXB9 **(C)**, HOXC9 **(D)**, HOXC11 **(E)** and HOXC13 **(F)** that are upregulated in tumor tissues at least 12 cancer types(P<0.05). *P<0.05; **P ≤ 0.01; ***P ≤ 0.001.

Based on the FPKM expression data of 33 cancers from the UCSC Xena, the expression of 39 HOX genes in 18 cancer types with more than 5 normal samples (normal tissues vs. tumor tissues) was inquired about (**Supplementary Figure S2**). A boxplot was used to depict the first six genes that were up-regulated in at least 12 different types of tumor tissues (**Figure 3**). The expression of HOXA13 was greatly up-regulated in 8 tumors, including GBM, HNSC, KIRC, KIRP, LIHC, LUAD, LUSC, and STAD (p<0.001). HOXB3 expression was greatly up-regulated in 7 tumors, including CHOL, COAD, GBM, LIHC, LUAD, LUSC, and PRAD (p<0.001). HOXB9 was shown to be considerably up-regulated in 12 different tumors, including CHOL, COAD, GBM, LIHC, LUAD, LUSC, et al. (p<0.001). HOXC9 was shown to be considerably up-regulated in 11 different tumors, including BLCA, BRCA, CHOL, LIHC, LUAD, LUSC, et al. (p<0.001). HOXC11 was shown to be considerably up-regulated in 10 different tumor tissues, including BRCA, COAD, LIHC, LUAD, LUSC, et al. (p<0.001). HOXC13 was significantly up-regulated in 14 tumors such as BLCA, BRCA, ESCA, GBM, LIHC, LUAD, et al. (p<0.001). Of course, some of the mentioned genes that show considerable up-regulation in certain tumors do not rule out the possibility that there are too few samples accessible.

### 3.4 Prognostic analysis of HOX genes in pan-cancer

**Figure 4:**
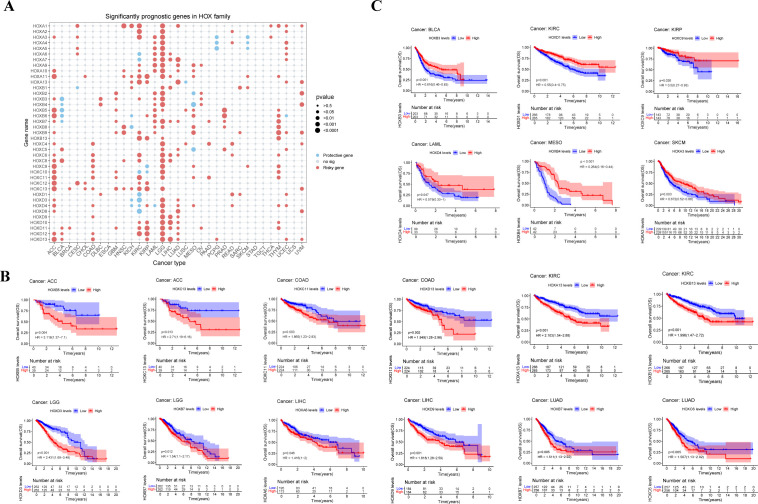
Relationship between HOX gene expression and prognosis in pan-cancer. **(A)** Conclusion of cox regression correlation of HOX with survival. Red dots indicate risky genes, while blue dots indicate protective genes, and gray dots indicate no significance(P>0.05). The size of dot is proportional to the P value. Survival of patients with high and low expression of HOX genes were predicted in a Kaplan–Meier survival curve analysis. High levels of HOX expression are linked to both worse **(B)** and better **(C)** overall survival. P-values, HRs and 95% confidence interval (CI) values are presented on the survival plots.

In order to further understand the possible prognosis and therapeutic effect of the HOX family in pan-cancer, we conducted the univariate cox regression analysis using the expression and survival data of HOX genes from UCSC Xena. Firstly, we calculated the Hazard ratio (HR) with their 95% confidence interval for each gene in the HOX family within each cancer and visualized it as a risk heatmap to show the significant prognostic genes in the HOX family (**Figure 4A**). Protective genes (HR<1) are depicted by red dots, whereas risky genes (HR>1) are represented by blue dots. Overall, HOX genes showed risk in most cancers, with just a few HOX genes being protective in some cancers (p<0.05). At least 18 HOX genes are risky genes in ACC, KIRC, and LGG, with LGG having the highest HOX risk genes. Furthermore, five HOXB genes are protective in BLCA, and four HOXD genes are protective in KIRC. Interestingly, three genes, HOXA4, HOXA5, and HOXA6, are found in both PCPG and SKCM.

Then, we analyzed the Kaplan-Meier survival curve of all HOX genes in pan-cancer based on the expression data of HOX genes from cancer patients in the TCGA database in order to explore the prognostic effect of the HOX family in pan-cancer. High expression of genes such as HOXB5, HOXC11, HOXA13, HOXD13, HOXA6, and HOXC6 in certain cancers is linked to poor overall survival (OS) (**Figure 4B**). On the contrary, there are also some genes, such as HOXB3, HOXD1, HOXC9, and HOXA13, that are highly expressed in some cancers and have better OS (p<0.05) (**Figure 4C**). However, some genes exhibit cancer heterogeneity, such as HOXA13, whose high expression has better OS in SKCM and worse OS in KIRC. We also chose Disease Specific Survival (DSS) (**Supplementary Figure S3**), and Progression Free Interval (PFI) to investigate the prognosis of HOX family in pan-cancer (**Supplementary Figure S4**).

The results of the risk heatmap and survival curve are independent between each other. Some genes have opposite results in these two analyses. In the survival curve analysis, for example, high expression of HOXA13 indicates better survival in SKCM and worse survival in KIRC (p<0.05); nevertheless, in the risk heatmap obtained by cox regression analysis, HOXA13 showed no significance in SKCM or KIRC. Simultaneously, some genes showed consistent results in both analyses. For example, in the K-M survival curve analysis, the high expression of HOXB7 and HOXC6 in LUAD led to worse OS. At the same time, consistent with the survival analysis, these two genes were also predicted as risky genes in the risk heatmap. The consistent findings establish a solid groundwork for further research.

### 3.5 Relationship between HOX genes and immune subtypes and immune microenvironment in pan-cancer

**Figure 5:**
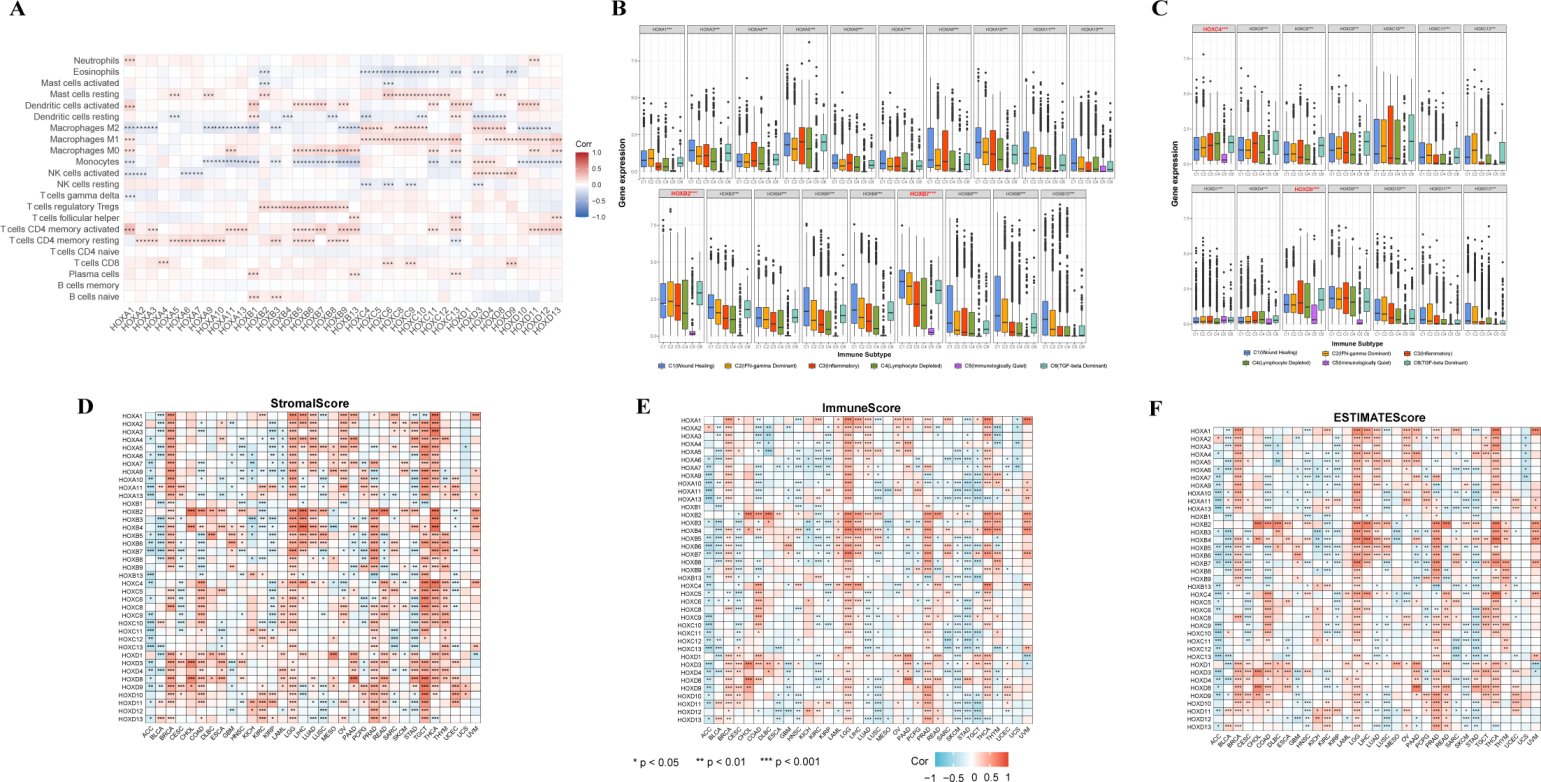
Relationship between the expression of the HOXs and tumor microenvironment. **(A)** Correlation between HOXs and 22 immune cells in pan-cancer based on CIBERSORT. **(B, C)** Relationship between the expression of the HOXs and the immune-infiltrating subtypes across all 33 cancer types. HOX genes with significant differences are labeled. C1: Wound healing, C2: INF-gamma dominant, C3: Inflammatory, C4: Lymphocyte depleted, C5: Immunologically quiet, and C6: TGF-beta dominant. Spearman correlation analysis was used to assess stromal scores **(D)**, immune scores **(E)**, and estimate scores **(F)**. The larger size and the color intensity of point, the better the correlation. Red indicates positive correlation and blue indicates negative correlation. *P<0.05; **P<0.01; ***P<0.001.

To gain further insights into the relationship between HOX and immune cell infiltration, we conducted an in-depth analysis of the correlation between the HOX family and immune cell infiltration using the CIBERSORT method in pancancer (**Figure 5A**). Different HOX subgroups exhibit similar correlation patterns with specific immune cells. The HOXB subgroup is positively correlated with regulatory T cells (Tregs), suggesting that the HOXB subgroup may influence immune cell infiltration by enhancing the immunosuppressive functions of Tregs. Studies have shown that HOXB7 binds with members of the PBX family in melanoma, promoting the expression of miR-221 and miR-222, which in turn regulate downstream factors, leading to increased cell proliferation and infiltration, blocking cell differentiation, and reducing cell apoptosis (35, 36). Additionally, HOXD9 is positively correlated with CD8+ T cells and negatively correlated with naive B cells, showing a starkly opposite correlation pattern among different types of immune cells. Studies have indicated that specific epigenetic regulatory factors, such as Dnmt3a, Tet2, and Asxl1, can modulate the response of CD8+ T cells in immunotherapy by affecting their stemness (37). HOXD9 may affect the epigenetic state of CD8+ T cells through similar mechanisms, thereby enhancing their anti-tumor activity. Furthermore, as CD8+ T cells play a central role in anti-tumor immunity, HOXD9 may improve the efficacy of immunotherapy by enhancing their function. Concurrently, the inhibitory effect of HOXD9 on naive B cells may reduce the number of immunosuppressive B cells in the TME, further potentiating the power of immunotherapy.

In order to clarify the relationship between HOX genes and immune infiltration subtypes, we studied the relationship between each HOX gene and six immune infiltration subtypes related to tumor immune promotion or inhibition in various cancers (**Figures 5B, C**). Previous studies have divided 33 kinds of tumors in TCGA into six immune subtypes (38): C1 (Wound Healing), C2 (INF-γ Dominant), C3 (Inflammatory), C4 (Lymphocyte Depleted), C5 (Immunologically Quiet), and C6 (TGF-β Dominant). Among them, the C1, C2, and C6 subtypes are related to tumors. Patients with C3 and C5 subtypes have better survival, while patients with C1, C2, C4, and C6 have worse survival, possibly because these four subtypes are more aggressive (39). The expression level of almost all HOX genes in the C5 subtype is very low, indicating that they may play a role in promoting tumorigenesis. However, HOXB7, HOXC4, and HOXD8 are highly expressed in six immune subtypes, with C5 subtype expression levels greater than other HOX genes, indicating that they may be a new breakthrough in immunosuppression. In addition, the expression levels of HOXB1, HOXC5, HOXC12, and HOXD12 in six immune subtypes are much lower than those of other HOX genes. Because of the poor prognosis of C4 and C6 immune subtypes, it is characterized by high macrophage content, limited lymphocyte infiltration, and high M2 macrophage content. The high expression of HOXB2 and HOXB7 in C4 and C6 subtypes also indicates that the two genes are highly correlated with immune infiltration and the possibility of immunosuppressive therapy. Additionally, HOXB2 and HOXB7 are highly expressed in C1, C2, and C6 subtypes, which also indicates that they play a role in promoting tumor progression. These three subtypes are known to have high cell proliferation and poor survival (38). Among the HOXB genes, except for HOXB1 and HOXB2, the order of expression levels of other genes in various immune subtypes is C1>C2>C3>C4, showing a relatively consistent trend.

We subsequently investigated the relationship between HOX gene expression levels, the infiltration levels of stromal and immune cells, and tumor purity (**Figures 5D–F**). Among them, the tumor purity is evaluated based on the stromal score, immune score, and estimate score of each sample in TCGA using the ESTIMATE algorithm (40). In the process of tumor occurrence and development, macrophages, dendritic cells, neutrophils, B cells, T cells, cancer associated fibroblasts (CAFs), and other cells are recruited into the microenvironment around tumor cells, playing their respective roles, and together with extracellular matrix and other elements, constitute the tumor immune microenvironment. In the tumor immune microenvironment, except tumor cells, other cells are generally divided into two categories: stromal cells and immune cells, and the content of these two cells is used as an index to judge the tumor purity. In each cancer sample, the higher the stromal cell score, the higher the stromal cell content, and the higher the immune cell score, the higher the immune cell content. The estimate score is the sum of the immune cell score and the stromal cell score. The lower the estimate score, the lower the content of these two kinds of cells in the tumor sample, and the more tumor cells it contains, the higher the tumor purity. There are great differences in stromal score, immune score, and estimate score of HOX genes in different cancers, but overall, the trend of the three scores is similar. Among BRCA, LGG, PRAD, TGCT, THCA, and THYM, more than half of HOX genes were positively correlated with stromal score (p<0.05). On the contrary, in ACC, KIRP, and STAD, more than half of HOX genes were negatively correlated with stromal score (p<0.05) (**Figure 5D**). The correlation between specific HOX genes and stromal scores in different cancer types can also be completely opposite. For example, HOXB7 was positively correlated with the stromal scores of patients with BRCA, LGG, LIHC, PRAD, THCA, and THYM (p<0.05). On the contrary, HOXB7 was negatively correlated with the stromal scores of patients with ACC, BLCA, CESC, COAD, HNSC, KIRP, LUSC, PCPG, and STAD. Similarly, in the correlation between HOX genes and immune score and estimate score, it showed a similar trend to stromal score (**Figures 5E, F**). For example, HOXB7 was positively correlated with the immune scores of patients with BRCA, LGG, LIHC, PRAD, SARC, THCA, THYM, and UVM (p<0.001), while HOXB7 was positively correlated with BRCA and LGG. However, in TGCT, the correlation between most HOX genes and stromal score is opposite to its correlation with immune cells.

In addition, we also studied the relationship between the HOX gene and tumor stemness and evaluated the similarity between tumor cells and stem cells through the stemness score. The stemness score is related to the active biological process in stem cells and the higher degree of tumor dedifferentiation (41). Based on RNAss and DNAss, we explore tumor stemness score from two aspects: gene expression and DNA methylation characteristics (**Supplementary Figure S5**). The high degree of immune infiltration is characterized by low RNAss or high DNAss. We found that most HOX genes were negatively correlated with RNAss in BRCA, KIRC, KIRP, LGG, TGCT, and THCA. At the same time, most HOX genes were positively correlated with DNAss in CHOL, GBM, LGG, and STAD. It is worth noting that most HOX genes are negatively correlated with RNAss and DNAss in TGCT.

### 3.6 Identification of potential compounds targeting HOX family using connective map analysis

**Figure 6:**
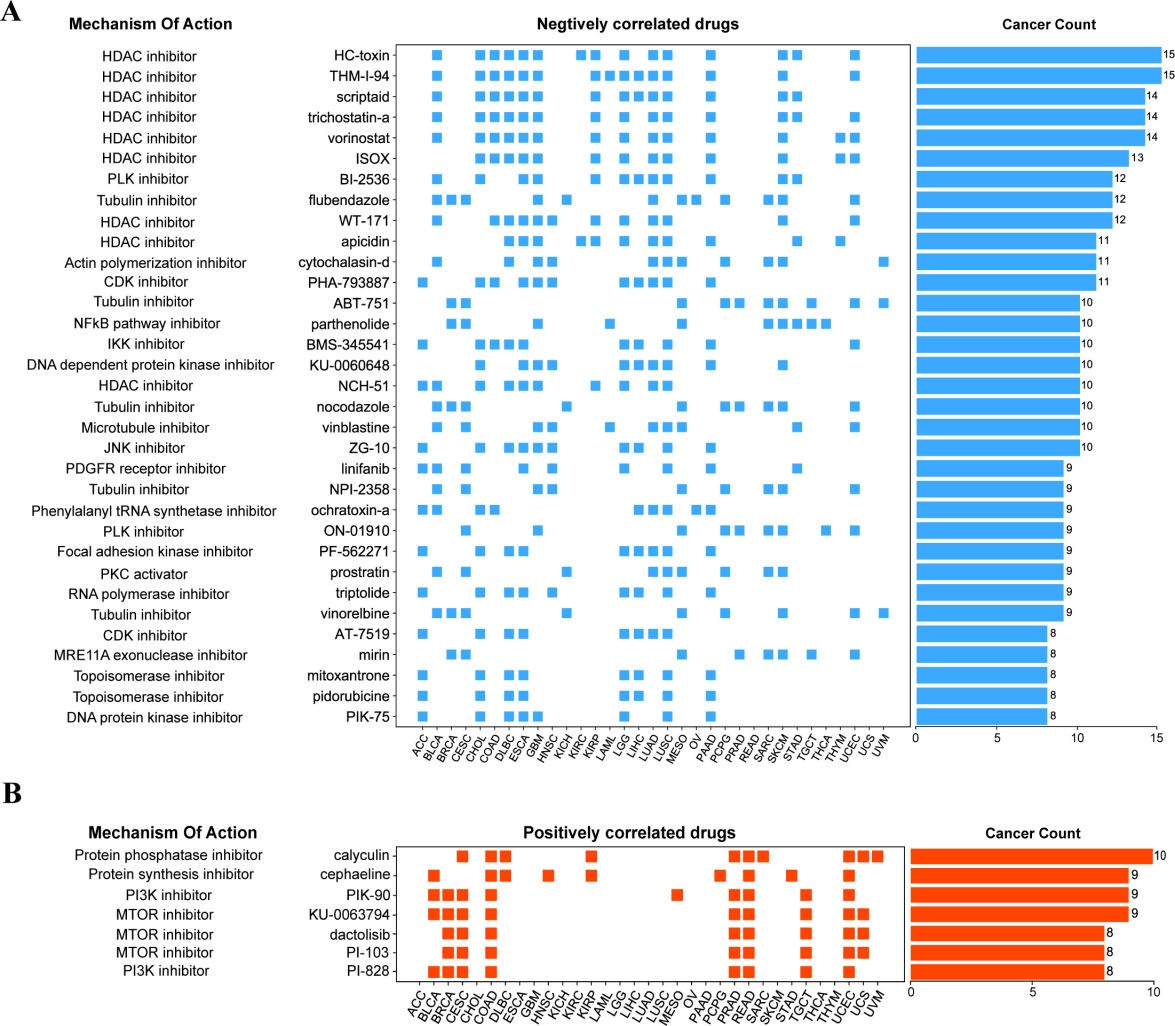
Correlation of HOX gene family with drugs from the Connectivity map. **(A, B)** The compounds negatively correlated **(A)** and positively correlated **(B)** with HOX score in pan-cancer are displayed by heatmap. The compounds are arranged in descending order according to its number of occurrences in significantly correlated cancer types.

**Figure 7:**
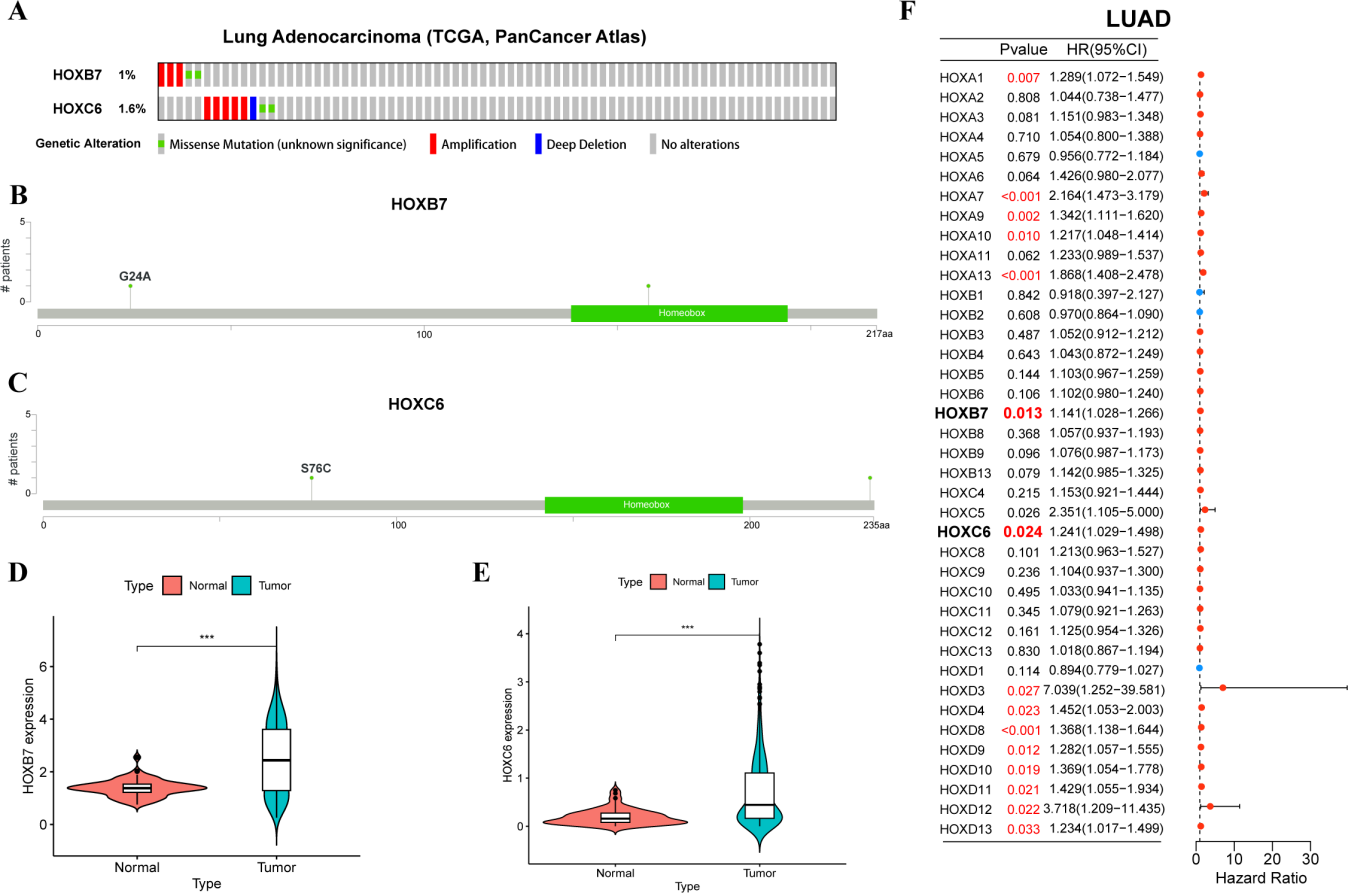
Mutation, expression, and univariate cox regression analysis of HOXB7 and HOXC6 in LUAD. **(A)** Oncoprint showing genetic alternations of HOXB7 and HOXC6 in LUAD. Mutation sites of HOXB7 **(B)** and HOXC6 **(C)** in LUAD by cBioportal. Violin plots showing differential expression HOXB7 **(D)** and HOXC6 **(E)** between normal and tumor tissues in LUAD. **(F)** Forest plot of univariate cox regression analysis of HOX family in LUAD. The symbol *** provided in **Figures 7D, E** represents that their p-values < 0.001.

The fundamental goal of biomedical research is to establish the connection between diseases, physiological processes, and small-molecule drug therapy. The Connective map is such a powerful tool to help achieve this goal (32, 42). It can find small molecules or drugs that share the mechanism of action through gene expression characteristics. Therefore, we further searched the small molecular compounds that may target the HOX family through the Connective map tool (version 1.0) in the hope of finding potential targets for disease treatment. We further set a threshold to narrow the scope of the final target compounds after collecting compounds with an absolute value of connective score greater than 90: at least 8 of the 33 cancer types are related to HOX score. Finally, we found 33 negatively related compounds (**Figure 6A**) and 7 positively related compounds (**Figure 6B**).

Among the compounds negatively related to the expression of HOX, 9 compounds belong to HDAC inhibitors, which are widely used as anti-tumor drugs (43), especially in the treatment of glioma, which means that inhibiting histone deacetylase may inhibit the expression of HOX, and the negative correlation between the compounds of this pathway and the expression of HOX can be found in 15 different cancers at most, including BLCA, CHOL, COAD, GBM, LUAD, and LUSC (**Figure 6A**). For example, Scriptaid has also been predicted to be a potential targeting drug that is negatively correlated with the expression of the oncogene TGIF1 in gliomas (44), which is consistent with our research findings. Besides, existing studies have demonstrated the effective role of scriptaid in cancer treatment. The combination of trichostatin A and scriptaid can enhance fenretinide-induced apoptosis in fenretinide-sensitive hepatocellular carcinoma (HCC) cells, reducing the resistance of HCC cells to fenretinide-induced apoptosis (45). At the same time, the Tubulin inhibitor and NF-κB pathway inhibitor are also drugs that are negatively correlated with HOX genes in many cancer types. For example, the Tubulin inhibitor flubendazole may down-regulate the expression of the HOX gene in 12 different cancers, including BLCA, BRCA, CESC, GBM, KICH, and LUAD, thus inhibiting the occurrence or progression of these cancers to some extent. Protein kinase inhibitors, protein synthesis inhibitors, and mTOR inhibitors, on the other hand, have been linked to increased HOX expression. The mTOR inhibitor dactolisib (BEZ-235), in combination with BMS-1166, promotes apoptosis in colorectal cancer cells by blocking the PI3K/mTOR pathway and disrupting the crosstalk of the MAPK pathway (46). Inhibition of these physiological processes can induce the expression of HOX genes.

### 3.7 Mutation, expression, and prognostic analysis of HOXB7 and HOXC6 in LUAD

Lung cancer, as one of the main causes of cancer death in the world, includes two pathological types: small cell lung cancer (SCLC, 15%) and non-small cell lung cancer (NSCLC, 85%) (47). Lung adenocarcinoma (LUAD) is the most prevalent histological subtype of non-small cell lung cancer with a poor prognosis, accounting for about 40% of lung malignant tumors (48). The molecular mechanism of LUAD is not clear enough at present, so it is important to identify and screen the markers targeting LUAD in time, which can help us better understand the critical role these genes play in the occurrence and development of LUAD.

Based on the above expression data, survival analysis, and results of univariate cox regression analysis, we chose HOXB7 and HOXC6 from LUAD for further analysis. They are identified by higher expression in LUAD tissue (p<0.05), worse OS in survival analysis (HR>1.5, p<0.05), and being predicted as risky genes in the univariate cox regression analysis (HR>1, p<0.05). Only HOXB7 and HOXC6 meet all three criteria in LUAD, thus making them more credible candidates for further research. In LUAD, the frequency of genetic alternations in HOXB7 only accounts for 1% of the genome, and HOXC6 accounts for 1.6% (**Figure 7A**). Gene amplification is their main alternation type. Furthermore, HOXC6 exhibits deep deletion, while HOXB7 does not. The lollipop chart of the mutation site revealed that both HOXB7 and HOXC6 had a mutation at a certain amino acid location, G24A and S76C, respectively (**Figures 7B, C**). Besides, we also evaluated the expression differences of HOXB7 (**Figure 7D**) and HOXC6 (**Figure 7E**) between tumor tissues and normal tissues in LUAD (based on log2(FPKM+1)), and both exhibit greater expression in tumor tissues with significant differences (p<0.001). Next, we performed univariate cox regression analysis on the HOX family in order to identify the HOX genes in LUAD that may have prognostic value (**Figure 7F**). HOXB7 and HOXC6 may be employed as prognostic genes in LUAD, as expected (HR>1, p<0.05).

### 3.8 Analysis of tumor mutation burden, microsatellite instability of HOXB7 and HOXC6 in LUAD

**Figure 8:**
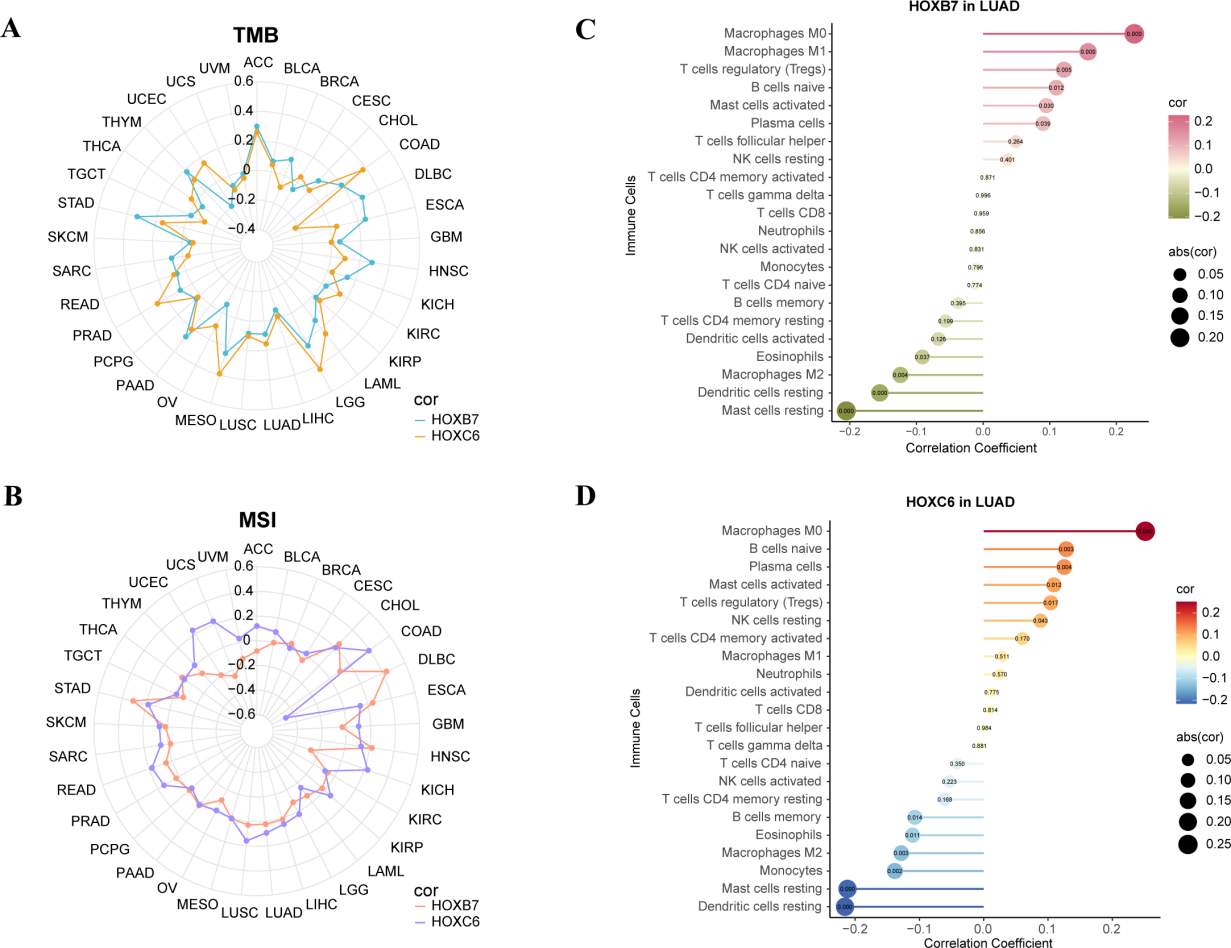
Analysis of TMB, MSI, CIBERSORT of the expression of HOXB7 and HOXC6 in cancers. **(A)** Radar chart to show the correlation between HOX expression and Tumor Mutation Burden (TMB) in pan-cancer. **(B)** Radar chart to show the correlation between HOX expression and Microsatellite Instability (MSI) in pan-cancer. **(C, D)** Lollipop charts of correlation between HOXB7 **(C)**, HOXC6 **(D)** expression and immune cells in LUAD based on CIBERSORT analysis. The numbers in circle are p-value.

As a biomarker of immunotherapy, Tumor mutation burden (TMB) can measure the number of tumor mutations. The higher TMB, the more new antigens are produced, and the easier it is to cause an immune response. Therefore, we explored the relationship between the expression of the HOX genes and TMB. HOXB7 and HOXC6 were positively correlated with TMB in most cancers, and HOXB7 was strongly correlated with TMB in ACC, HNSC, PAAD, and STAD, and HOXC6 was strongly correlated with TMB in COAD, LGG, MESO, and PRAD (R>0.2, **Figure 8A**).

Microsatellite instability (MSI) associated with DNA mismatch repair functional defects is an important tumor marker in clinical research (49, 50). Multiple cancer forms, including colon cancer, glioblastoma, ovarian cancer, gastric cancer, and prostate cancer, have been reported to be associated with MSI. Therefore, we analyzed the microsatellite instability of the above six genes. We found that HOXB7 had a stronger positive correlation with MSI in CHOL, DLBC, ESCA, HNSC, and STAD (R>0.2), while HOXC6 had a stronger positive correlation with MSI in COAD, KICH, UCEC, and UCS (R>0.2). Strangely, HOXC6 had a strong negative correlation with DLBC (R<-0.4, **Figure 8B**).

Then, the reference marker gene expression matrix “LM22.txt” of 22 kinds of immune cells was downloaded from CIBERSORT website (51). We studied the correlation between the two genes and 22 immune cell subtypes in LUAD (**Figures 8C, D**). We found that HOXB7 in LUAD had the strongest positive correlation with macrophage M0 and macrophage M1 and the strongest negative correlation with mast cells resting and dendritic cells resting (p<0.001). HOXC6 in LUAD had the strongest positive correlation with macrophage M0 and the strongest negative correlation with mast cells resting and dendritic cells resting (p<0.001).

### 3.9 Exploration on the interaction genes and functions of HOXB7 and HOXC6 in LUAD

**Figure 9:**
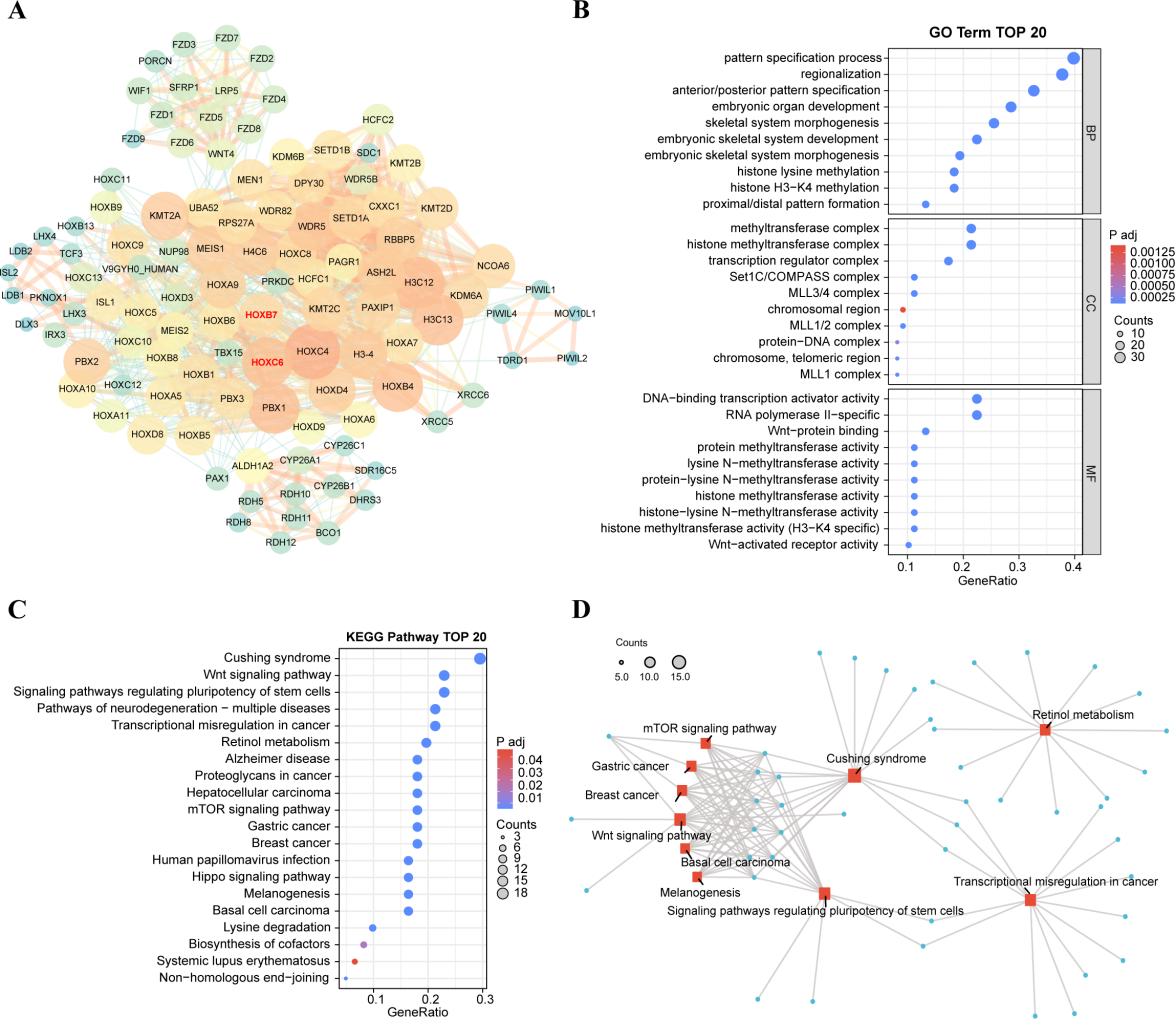
Functional exploration of HOXB7 and HOXC6. **(A)** Construction of PPI network involved in top 100 interacting genes with HOXB7 and HOXC6 from STRING database. The interacting genes were visualized in Cytoscape. Degree is shown in terms of node size and node color. **(B)** Gene Ontology functional enrichments in the network of top 100 interacting genes from STRING database. The strength of functional association between their respective products of top 30 GO terms are showed. **(C)** TOP 20 KEGG pathway of interacting genes related to HOXB7 and HOXC6. **(D)** Network of gene interactions of top 10 KEGG pathways.

In order to further understand the functions of these six genes, we used the STRING database to construct PPI networks of six interacting proteins of HOX genes and visualized them by Cytoscape (**Figure 9A**). The genes marked in red are the two HOX genes of interest in this project. Node size indicates the number of genes interacting with the node. We found that interaction genes which has high degree include HOXC4, H3C12, PBX1, WDR5, KMT2A, RBBP5, and so on. Like the HOX family, PBX1 has a strong correlation with organogenesis, differentiation, and development, and it is also a key character of the hallmarks of cancer, its up-regulation of expression is linked to carcinogenesis and a worse prognosis (52). WDR5, a member of the WD40 protein family, up-regulates expression in oral squamous cell carcinoma (OSCC), and silencing WDR5 can inhibit the occurrence of OSCC (53), and it might be a target for OSCC treatment. KMT2A is involved in coding lysine methyltransferase and epigenetics, and is also associated with various processes of embryonic development, which are conservative in evolution and mostly connected to the emergence of hematological malignant tumors (54). RBBP5, retinoblastoma binding protein 5, has been proven to be related to the progress of hepatocellular carcinoma (55).

Next, we visualized the functions of top 100 interacting genes predicted by the STRING database and showed the top 30 terms of GO enrichment results, each of which corresponds to three categories: biological process (BP), cellular component (CC), and molecular function (MF) (**Figure 9B**). GO enrichment function showed that the genes interacting with HOXB7 and HOXC6 are involved in the following terms: pattern specification process, embryonic organ development, histone H3−K4 methylation, Wnt−protein binding and so on. Besides, these genes play a role in several significant KEGG pathways (**Figures 9C, D**), including the Wnt signaling pathway, pathways of neurodegeneration−multiple diseases, transcriptional misregulation in cancer, hepatocellular carcinoma and mTOR signaling pathway, which are closely tied to cancer. These results suggest a potential link between carcinogenesis and HOXB7 and HOXC6.

### 3.10 Expression verification of HOXB7 and HOXC6 in LUAD following immunohistochemistry

**Figure 10:**
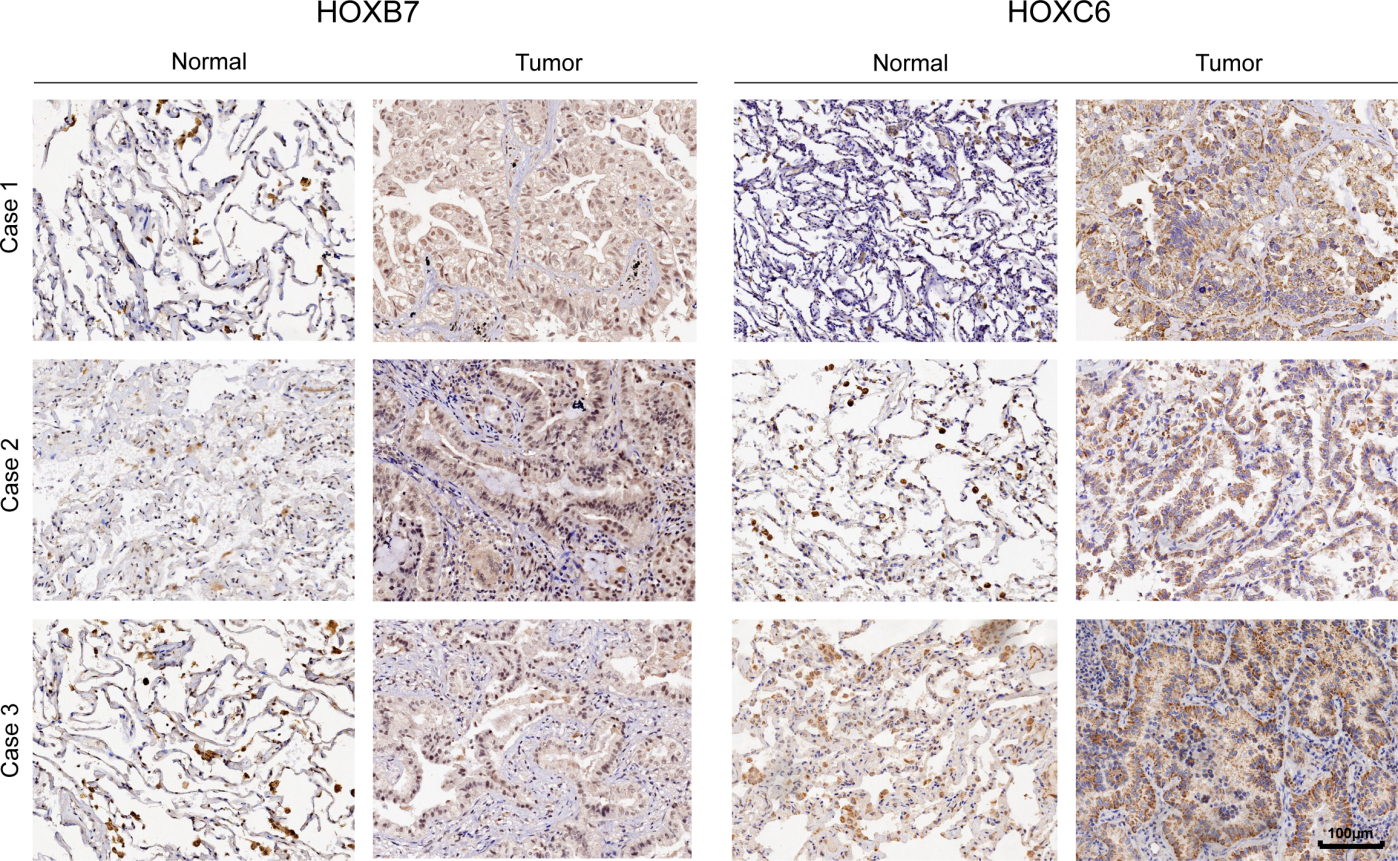
The representative immunohistochemistry images of HOXB7 and HOXC6 genes expression in normal and tumor tissue in LUAD.

We further used immunohistochemistry to confirm the essential function of HOXB7 and HOXC6 in LUAD at the tissue level. Consistent with the previous results, HOXB7 and HOXC6 showed significantly higher expression in tumor tissues as compared to the adjacent normal tissues. Besides, HOXB7 is expressed in the nucleus, whereas HOXC6 is in the cytoplasm (**Figure 10**).

### 3.11 HOXB7 and HOXC6 affected the proliferation and migration of LUAD cells

**Figure 11:**
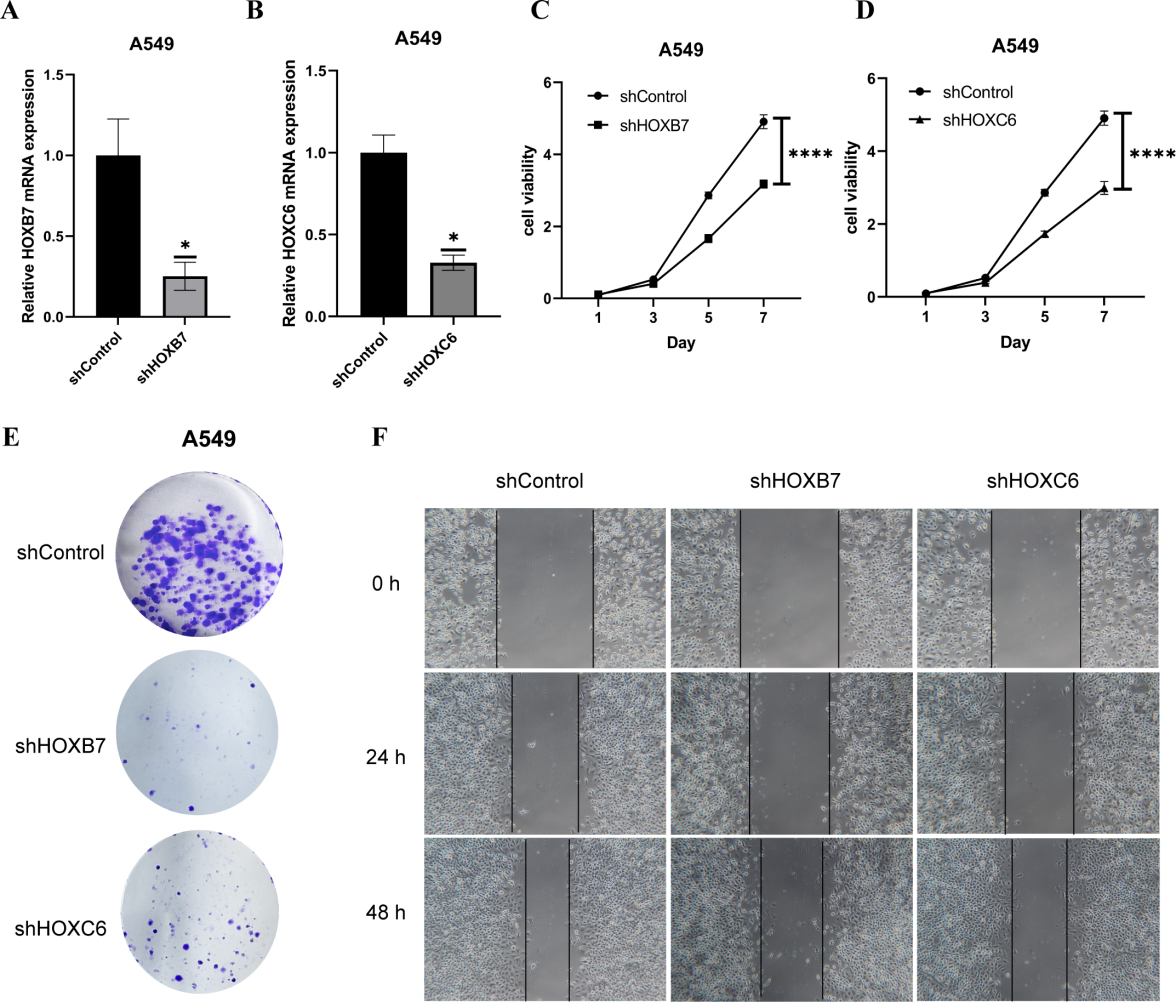
Knockdown of HOXB7 and HOXC6 inhibits cell viability, proliferation and migration. The mRNA expression levels of HOXB7 **(A)** and HOXC6 **(B)** in A549 cell line. The results of CCK-8 showed that the knock-down of HOXB7 **(C)** and HOXC6 **(D)** in A549 can inhibits cell viability. **(E)** Colony formation assay of A549-shControl/shHOXB7/shHOXC6 for 14 days. **(F)** Wound healing assays for evaluating the migration capacity of A549 cells with shControl/shHOXB7/shHOXC6. (****p< 0.0001, compared with shControl).

We further verified their role in the development of lung adenocarcinoma by knocking down HOXB7 and HOXC6 in A549 and NCI-H1975 cells respectively (**Figure 11**, **Supplementary Figure S6**). RT-PCR results showed that the knock-down efficiency of HOXB7 and HOXC6 was more than 70% in A549 and NCI-H1975. We studied their effects on cell viability, proliferation and migration. Therefore, the outcomes of these experiments *in vitro* showed that HOXB7 and HOXC6 are key oncogenes that promote tumor malignant behavior and cell proliferation in LUAD cell lines.

## 4 Discussion

Numerous studies have shown that the normal expression of the HOX gene is strongly related to embryonic development, cell differentiation, and cancer. When HOX expression is aberrant, it can disrupt downstream signal transduction by itself or in collaboration with other molecules, leading to the occurrence and development of tumors. At the same time, in some tumors, if the signal pathway of organ development related to HOX genes is disturbed during embryonic development, it may affect the differentiation and growth of normal cells, and the cells will turn in a worse direction, thus promoting the emergence of tumors.

Currently, most HOX gene research focuses on a single gene and a single cancer, a single gene and multiple cancers, and multiple genes and a single cancer. A previous study found that HOXB9 can mediate tumor angiogenesis, epithelial-mesenchymal transition (EMT), and cancer stemness through the TGF-β pathway, leading to chemical resistance and poor survival in pancreatic cancer patients (56); There are also studies to explore the carcinogenesis of the HOX gene cluster in head and neck squamous cell carcinoma (57) and potential tumor suppressor in renal clear cell carcinoma (20); HOXA4 and HOXA5 affect the function and prognosis of lung cancer cells and may be potential biomarkers of LUAD (58). However, research on the HOX gene family in pan-cancer is still limited, so it is necessary for us to conduct a comprehensive study on it to learn about the overall situation of the HOX family in pan-cancer and provide some evidence for further exploring the mechanism of HOX genes and cancers and finding potential therapeutic targets.

This study examined the mutation landscape of the HOX family using cBioPortal data. Results revealed that the types of genetic alterations within the HOX family subtypes were comparable, which might be attributed to their common genetic background and evolutionary conservatism (59). Furthermore, this HOX family mutation pattern has some tumor specificity. Additionally, the expression data analysis based on the TCGA database revealed the cancer heterogeneity of HOX gene expression, which may play distinct roles in different cancers. Previous research had shown that reducing HOXA5 expression contributes to the proliferation of breast cancer cells (14); In addition, MicroRNA-224 promotes the migration and invasion of hepatocellular carcinoma cells by targeting the expression of HOXA5 (60). Down-regulating its expression by regulating hypermethylation in the promoter region of HOXA5 can be regarded as a new direction for a future therapeutic target (61). According to this study, HOXA5 is down-regulated in BRCA, LUAD, COAD, and READ but up-regulated in LIHC, GBM, and HNSC, which coincides with previous studies. Similar genes include HOXB7, HOXC6, HOXA6, et al. In addition, the expression of HOX genes in GBM is almost up-regulated, which is consistent with the conclusion that HOX genes are usually oncogenes in GBM in previous studies (62).

The tumor immune microenvironment (TME) is made up of cellular components (such as tumor cells, vascular endothelial cells, immune cells, and mesenchymal stem cells) as well as non-cellular components (such as cytokines, adhesion molecules, and growth factors) that work together to form a barrier for tumor cells. Additionally, as integral non-neoplastic components of TME, the scoring of stromal and immune cells can be utilized as significant indicators for the survival, recurrence, and metastasis in cancer patients (63). A burgeoning scientific literature corroborates that immune cell infiltration is a pivotal factor in both tumor progression and the efficacy of immunotherapeutic interventions (64). The HOX gene plays an important role in the tumor immune microenvironment (22, 65). In colorectal cancer, for example, high HOXC6 expression is highly associated with the remodeling of TME, which can be used as a potential biomarker to predict the efficacy of immunotherapy for non-metastatic colorectal cancer (22). Therefore, we explored the relationship between HOX expression and the immune microenvironment, immune subtypes, and immune infiltration in pan-cancer via bioinformatics analysis and evaluated the tumor purity. The results showed that the expression of most HOX genes had a strong relationship with immune subtypes (p<0.001). HOXB7, HOXC4, and HOXD8 are all highly expressed in six immune subtypes, which indicates that they may be a new immunosuppressive breakthrough. In some cancers, such as BRCA, KIRC, LGG, TGCT, CHOL, GBM, and STAD, most HOX genes have significant levels of immune infiltration—higher DNAss or lower RNAss. Consequently, the exploration of the association between HOX genes and the immune microenvironment can aid in identifying biomarkers related to TME, which may be correlated with disease prognosis, therapeutic response, or disease progression. We can also shed light on the associated genes and pathways to help us understand the molecular mechanisms of diseases and provide a basis for developing new treatment strategies. Given the intricacies of the tumor microenvironment, the translation of cancer research into clinical practice remains a formidable challenge for future oncology.

Immunosuppressant therapy has become a new milestone in the treatment of malignant tumors. Among them, TMB, MSI, mismatch repair (MMR), and other biomarkers have been proven to have certain predictive values for immune efficacy and have been widely recognized. Based on the results of expression data, survival analysis and cox regression analysis, HOXB7 and HOXC6 were further analyzed, including TMB, MSI, and CIBERSORT immune infiltration analysis. HOXB7 showed strong positive correlations with TMB and MSI in STAD, and HOXC6 showed strong positive correlations with both TMB and MSI in COAD, suggesting that they might be employed as biomarkers in STAD and COAD in the future.

The abundance of immune cells, especially T cells, is closely related to tumor progression. Regulatory T Cells (Tregs) are the main immunosuppressive and anti-inflammatory cells, which can inhibit T cell reactions and lead to T cell failure and immune escape (66, 67). Natural killer (NK) cells have an anti-tumor effect (68). Tumor-associated Macrophages (TAMs) can be polarized into different functional phenotypes, including macrophages M1 and macrophages M2. Macrophages M1 can inhibit the proliferation of tumor cells by presenting antigens to adaptive immune cells, producing proinflammatory cytokines, and phagocytizing tumor cells (69). Macrophages M2 contribute to cell proliferation and promote the progress of cancer (70). M0 macrophages developed from M1 macrophages can also kill tumor cells (71, 72). The correlation of immune cell infiltration indicates that the relationship between HOX genes and immune cell infiltration is multifaceted, reflecting their role in shaping the TME. Studies have demonstrated that the expression of HOX genes correlates with the level of immune cell infiltration, particularly in certain types of cancer where the expression patterns of HOX genes are associated with the presence and status of immune cells within the TME (65). For instance, HOXB-AS1 has been shown to have significantly higher expression levels in glioma compared to low-grade glioma tissues. *In vitro* cellular functional experiments have indicated that HOXB-AS1 supports tumor stem cells and plays a crucial role in the metastasis of glioma (73). This suggests that HOX genes may influence the infiltration and function of immune cells by modulating the characteristics of tumor stem cells. Furthermore, the role of HOX genes in the TME extends beyond immune cell infiltration; they are also implicated in immune regulation and the efficacy of immunotherapy. In hepatocellular carcinoma (HCC), the expression pattern of the HOX gene family defines the TME and the effects of immunotherapy. HCC samples with a high HOXscore exhibit a richer infiltrate of suppressive immune cells and are more sensitive to certain anticancer drugs such as mitomycin and cisplatin (65). Importantly, the HOXscore is associated with the therapeutic effects of PD-L1 blockade, indicating that HOX genes may modulate immune responses within the TME by affecting the expression and function of immune checkpoints. In addition, the correlation of two HOX genes in 22 kinds of immune cells were evaluated by CIBERSORT. In LUAD, we observed a robust positive correlation between HOXB7 and macrophages M0 and M1, suggesting an association with tumor inhibition. Interestingly, HOXC6 exhibited a similar pattern, contrary to prior research findings. The positive correlation between HOXB7, HOXC6, and tumor suppressor genes may have certain limitations. We hypothesize that the expression of HOXB7 and HOXC6 follows a normal distribution trend during cancer cell occurrence and development. Elevated expression of HOXB7 and HOXC6 potentially facilitates cancer progression, leading to the accumulation of cancer cells. Upon reaching a critical mass, immune cells associated with cancer suppression gather, resulting in the observed positive correlation phenomenon.

In summary, the correlation between HOX genes and immune cell infiltration is a complex phenomenon that involves the shaping of the TME, immune regulation, and the effects of immunotherapy. These findings provide new perspectives and potential targets for future cancer treatment strategies targeting HOX genes. Future research is needed to further elucidate how HOX genes precisely regulate the behavior of immune cells and their specific mechanisms of action within the TME.

In order to establish the relationship between diseases, physiological processes and small molecular drugs, we searched for potential targeted compounds with positive and negative correlation with the HOX score through the Connective Map, which laid the foundation for future clinical cancer treatment. Most of the predicted compounds belong to HDAC inhibitors, Tubulin inhibitors, and NF-κB inhibitors, which can down-regulate the expression of HOX in specific cancers, thus inhibiting the development of tumors. HDAC inhibitors are widely used as anti-tumor drugs (43), especially in the treatment of glioma. Tubulin inhibitors have an anti-tumor effect and can limit the proliferation of several cancer cell lines (74, 75). The dysfunction of NF-κB regulation is related to many human disorders, such as cancer, inflammation, autoimmune diseases, and aberrant immune system development, and the occurrence and development of these diseases can be hindered by suppressing the NF-κB pathway (76, 77). On the contrary, protein kinase inhibitors, protein synthesis inhibitors, and mTOR inhibitors can induce the expression of HOX genes, so we can induce the expression of HOX genes as tumor suppressors by using related small molecular drugs, thus inhibiting the proliferation of tumor cells.

The exploration of gene family expression across various cancers and its significance in prognosis and the immune microenvironment in 33 types of cancer is of great importance. Firstly, the expression levels of gene family members have the potential to predict prognosis and response to immunotherapy in cancer. For instance, the expression of APOBEC family genes in bladder cancer is associated with patient prognosis and response to immunotherapy (78). Secondly, the immune microenvironment correlates with tumor prognosis and can predict treatment response and outcomes in cancers such as breast cancer. In HER2+ breast cancer, the expression profile of immune genes and tumor-infiltrating lymphocytes (TILs) serve as predictive factors, indicating the prognostic value of early treatment (79). Thirdly, gene expression not only affects the biological characteristics of tumor cells but may also regulate immune cell infiltration and immune responses within the tumor microenvironment. For example, the expression of CXCL13 in the microenvironment of mouse breast cancer can regulate the infiltration of immune cells to induce anti-tumor immune responses (80). Finally, the expression patterns of gene family members can interact with the tumor immune microenvironment, offering new perspectives for cancer treatment. They can serve not only as prognostic biomarkers but also as potential targets for immunotherapy. The P2Y family of genes may emerge as new targets for tumor immunotherapy (81).

HOX genes have a complex relationship with immune responses, being involved not only in the initiation and progression of tumors but also potentially influencing the tumor’s immune microenvironment and the efficacy of immunotherapy through various mechanisms. The activation of the TGF-beta signaling pathway dependent on HOXB7 leads to increased cell motility and invasiveness, while also recruiting and activating macrophages of the immune system, indicating that HOXB7 is a crucial hub for tumor cells to activate and exploit their own immune system (82). HOXC8 not only affects the progression and prognosis of CRC by mediating the upregulation of tumor escape-related pathways such as EMT, but it may also be involved in immune regulation within the TME (83). The role of HOXB7 extends beyond its influence on tumor-associated macrophages (TAMs). It also promotes immune modulation by regulating the recruitment and polarization of these cells, thereby affecting their activity within the tumor microenvironment. This influence may steer TAMs towards an M2-like anti-inflammatory phenotype, ultimately suppressing the immune response against the tumor and actively facilitating immune evasion by the cancer (65, 84).

To sum up, by examining gene expression across 33 cancers in the context of the immune microenvironment and prognosis, we can better understand the role of gene families in cancer development, identify prognostic biomarkers, and uncover potential targets for immunotherapy. Such insights are instrumental not only for the improvement of early diagnostic paradigms and therapeutic modalities within clinical oncology but also for formulating new treatment strategies that may reshape the therapeutic landscape for cancer patients.

In this research, we executed an examination of the HOX family’s oncogenic and prognostic significance spanning diverse tumors, a novel contribution to the field. Our analysis revealed that HOXB7 and HOXC6 are prognostic indicators in LUAD, markedly affecting cell migration and proliferation. Unlike typical pan-cancer studies, we introduced a HOX score to denote average expression levels across cancers. Using connective map analysis, we identified drugs that modulate HOX expression or related pathways, suggesting that HOX gene targeting could be a promising therapeutic strategy. The impact of these compounds on HOX expression and cancer progression merits further research. Our findings also hold significant implications for clinical applications. Firstly, the high expression of HOXB7 and HOXC6, along with poor prognosis, suggests they may serve as potential therapeutic targets, with their gene expression being utilizable for assessing patient outcomes. Given the dual roles of some HOX genes in different tumors, precision medicine and tailored treatment plans for patients are essential. Secondly, the positive correlation between the HOXB subgroup and the infiltration level of Tregs indicates that the immunosuppressive function of Tregs in tumor tissues is closely related to their ability to take up lactate (85). It is anticipated that directly targeting lactate metabolism or inhibiting tumor acidity could disrupt Treg-mediated immunosuppression, thereby synergizing with cancer immunotherapies. Additionally, the correlation of the HOX family with indicators such as TMB and MSI suggests that the expression levels of HOX genes could predict patient responses to immunotherapy, helping to identify potential beneficiaries of immunotherapy and guide treatment decisions and immune effect assessments. Lastly, combining results from cMap, the development of small molecule inhibitors targeting HOX genes, such as HDAC inhibitors, may emerge as a new therapeutic strategy. The above summarizes the distinctive contributions of our research.

However, it is essential to acknowledge the limitations inherent in our research. Initially, the expression data and clinical annotations utilized in this study were derived exclusively from UCSC Xena, which may bias results due to limited normal tissue samples. Future work should include more databases, an independent cohort for validation, and expanded sample cohorts. Furthermore, Pan-cancer analysis deals with a vast amount of data with varying biological and molecular characteristics, which increases the complexity of analysis and computational demands. Algorithms may have limitations and need continuous optimization to improve the precision and reliability of results. While our findings suggest prognostic value of certain HOX genes and potential therapeutic targets, further *in vivo* or clinical trials are needed to clarify mechanisms and ensure safe, effective treatments. Moving forward, it is imperative to conduct in-depth investigations into the expression and regulatory mechanisms of the HOX family in cancer, utilizing a multifaceted approach to enrich our understanding and pave the way for potential therapeutic advancements.

## Data Availability

Publicly available datasets were analyzed in this study. This data can be found here: https://xenabrowser.net/datapages/.

## Supplementary material

Supplementary Figure 1 Mutations of each HOX gene in 32 cancers (TCGA, PanCancer Atlas) by cBioportal.

Supplementary Figure 2 Expression level of HOX family in primary tumor tissues and normal tissues across 18 cancer types which have more than five normal tissue samples.

Supplementary Figure 3 Disease Specific Survival (DSS) of HOX family in pan-cancer(p<0.05).

Supplementary Figure 4 Progression Free Interval (PFI) of HOX family in pan-cancer(p<0.05).

Supplementary Figure 5 Correlation analysis between tumor stemness and the expression of HOX gene family in pan-cancer.

Supplementary Figure 6 The impact of knocking down HOXB7 and HOXC6 on the cell viability, proliferation, and migration of the NCI-H1975 lung adenocarcinoma cell line.

## References

[1] Salomone J Farrow E Gebelein B. Homeodomain complex formation and biomolecular condensates in hox gene regulation. Semin Cell Dev Biol. (2022) 152-153:93–100. doi: 10.1016/j.semcdb.2022.11.016 36517343 PMC10258226

[2] Lewis EB. A gene complex controlling segmentation in Drosophila. Nature. (1978) 276:565–70. doi: 10.1038/276565a0 103000

[3] Smith J Zyoud A Allegrucci C. A case of identity: hox genes in normal and cancer stem cells. Cancers (Basel). (2019) 11:512. doi: 10.3390/cancers11040512 30974862 PMC6521190

[4] Mallo M. Reassessing the role of hox genes during vertebrate development and evolution. Trends Genet. (2018) 34:209–17. doi: 10.1016/j.tig.2017.11.007 29269261

[5] Quinonez SC Innis JW. Human hox gene disorders. Mol Genet Metab. (2014) 111:4–15. doi: 10.1016/j.ymgme.2013.10.012 24239177

[6] Meyer A. Hox gene variation and evolution. Nature. (1998) 391:227–8. doi: 10.1038/34530 9440682

[7] McGinnis W Krumlauf R. Homeobox genes and axial patterning. Cell. (1992) 68:283–302. doi: 10.1016/0092-8674(92)90471-n 1346368

[8] Pearson JC Lemons D McGinnis W. Modulating hox gene functions during animal body patterning. Nat Rev Genet. (2005) 6:893–904. doi: 10.1038/nrg1726 16341070

[9] Tiberio C Barba P Magli MC Arvelo F Chevalier TL Poupon MF. Hox gene expression in human small-cell lung cancers xenografted into nude mice. Int J Cancer. (2010) 58:608–15. doi: 10.1002/ijc.2910580426 7914516

[10] Zhang B Li N Zhang H. Knockdown of homeobox B5 (Hoxb5) inhibits cell proliferation, migration, and invasion in non-small cell lung cancer cells through inactivation of the wnt/beta-catenin pathway. Oncol Res. (2018) 26:37–44. doi: 10.3727/096504017X14900530835262 28337958 PMC7844563

[11] Aparecida d Mafalda A Tiago P Renata F. Hoxb7 overexpression leads triple-negative breast cancer cells to a less aggressive phenotype. Biomedicines. (2021) 9:515. doi: 10.3390/biomedicines9050515 34063128 PMC8148148

[12] Quanfang C Qingyun P Han G Yingju W Xiaoning Z. Mir-17-5p/hoxa7 is a potential driver for brain metastasis of lung adenocarcinoma related to ferroptosis revealed by bioinformatic analysis. Front Neurol. (2022) 13:878947. doi: 10.3389/fneur.2022.878947 35693013 PMC9174431

[13] Rusan M Andersen RF Jakobsen A Steffensen KD. Hoxa9 methylation in circulating tumor DNA as a prognostic biomarker in brca-mutated ovarian cancer patients treated with parp inhibitor. Ann Oncol. (2018) 29:viii341. doi: 10.1093/annonc/mdy285.162

[14] Raman V Martensen SA Reisman D Evron E Odenwald WF Jaffee E. Compromised hoxa5 function can limit P53 expression in human breast tumours. Nature. (2000) 405:974–8. doi: 10.1038/35016125 10879542

[15] Alka S Sameer G Jaydeep AB Manisha S. Detection of aberrant methylation of hoxa9 and hic1 through multiplex methylight assay in serum DNA for the early detection of epithelial ovarian cancer. Int J Cancer. (2020) 147:1740–52. doi: 10.1002/ijc.32984 32191343

[16] Li X Chen S Zhu Y Fei J Song L Sun G. Comprehensive bioinformatics analyses identified homeobox B9 as a potential prognostic biomarker and therapeutic target for gastric cancer. J Gastrointestinal Oncol. (2021) 12:2132–49. doi: 10.21037/jgo-21-598 PMC8576221 34790380

[17] Zhang J Chen B Wang Y Liu X Yan H Wong KY. The E2f1–hoxb9/pbx2–cdk6 axis drives gastric tumorigenesis and serves as a therapeutic target in gastric cancer. J Pathol. (2023) 260:402–16. doi: 10.1002/path.6091 37272544

[18] Jianjiao L Huiqiong Z Linjie H Weimei T Jing W Hongsong H. Coexpression of hoxa6 and pbx2 promotes metastasis in gastric cancer. Aging. (2021) 12:6606–24. doi: 10.18632/aging.202426 PMC7993744 33535170

[19] Hong YQ Hong Z Fu W Wang Q Zeng Y Qi L. Microrna-384 is lowly expressed in human prostate cancer cells and has anti-tumor functions by acting on hoxb7. Biomed Pharmacother. (2019) 114:108822. doi: 10.1016/j.biopha.2019.108822 30951946

[20] Di Z Jinzhuo N Yuqi X Yuan R Fan C. Comprehensive analysis of a homeobox family gene signature in clear cell renal cell carcinoma with regard to prognosis and immune significance. Front Oncol. (2022) 12:1008714. doi: 10.3389/fonc.2022.1008714 36387262 PMC9660242

[21] Yang Y Chao L Bojun W Xin G Lin F Fei Z. Hoxb9 blocks cell cycle progression to inhibit pancreatic cancer cell proliferation through the dnmt1/rbl2/C-myc axis. Cancer Lett. (2022) 533:215595. doi: 10.1016/j.canlet.2022.215595 35182659

[22] Lina Q Chenyang Y Ding Z Rui B Shu Z Wangxiong H. The effects of differentially-expressed homeobox family genes on the prognosis and hoxc6 on immune microenvironment orchestration in colorectal cancer. Front Immunol. (2021) 12:781221. doi: 10.3389/fimmu.2021.781221 34950145 PMC8688249

[23] Cory A-S. Deregulated homeobox gene expression in cancer: cause or consequence? Nat Rev Cancer. (2002) 2:777–85. doi: 10.1038/nrc907 12360280

[24] Serena C Vita F Davide M. Hox genes family and cancer: A novel role for homeobox B9 in the resistance to anti-angiogenic therapies. Cancers. (2020) 12:3299. doi: 10.3390/cancers12113299 33171691 PMC7695342

[25] Dan L Ting YW Sheng ZP. Hoxb4 inhibits the proliferation and tumorigenesis of cervical cancer cells by downregulating the activity of wnt/B-catenin signaling pathway. Cell Death Dis. (2021) 12:105. doi: 10.1038/s41419-021-03411-6 33479226 PMC7820415

[26] Lei X Lei Y Li J-K Du W-X Li R-G Yang J. Immune cells within the tumor microenvironment: biological functions and roles in cancer immunotherapy. Cancer Lett. (2020) 470:126–33. doi: 10.1016/j.canlet.2019.11.009 31730903

[27] Yost KE Satpathy AT Wells DK Qi Y Wang C Kageyama R. Clonal replacement of tumor-specific T cells following pd-1 blockade. Nat Med. (2019) 25:1251–9. doi: 10.1038/s41591-019-0522-3 PMC6689255 31359002

[28] Schindler K Harmankaya K Postow M Frantal S Bello D Ariyan C. Pretreatment levels of absolute and relative eosinophil count to improve overall survival (Os) in patients with metastatic melanoma under treatment with ipilimumab, an anti ctla-4 antibody. J Clin Oncol. (2013) 31:9024–. doi: 10.1200/jco.2013.31.15_suppl.9024

[29] Chang Q Zhang L He C Zhang B Zhu Z. Hoxb9 induction of mesenchymal-to-epithelial transition in gastric carcinoma is negatively regulated by its hexapeptide motif. Oncotarget. (2015) 6:42838–53. doi: 10.18632/oncotarget.v6i40 PMC4767475 26536658

[30] Daojiang L Yang B Zhicai F Wanwan L Chunxing Y Yihang G. Study of promoter methylation patterns of hoxa2, hoxa5, and hoxa6 and its clinicopathological characteristics in colorectal cancer. Front Oncol. (2019) 9:394. doi: 10.3389/fonc.2019.00394 31165042 PMC6536611

[31] Cerami E Gao J Dogrusoz U Gross BE Sumer SO Aksoy BA. The cbio cancer genomics portal: an open platform for exploring multidimensional cancer genomics data. Cancer Discovery. (2012) 2(5):401–4. doi: 10.1158/2159-8290.CD-12-0095 PMC3956037 22588877

[32] Lamb J Crawford ED Peck D Modell JW Blat IC Wrobel MJ. The connectivity map: using gene-expression signatures to connect small molecules, genes, and disease. Science. (2006) 313:1929–35. doi: 10.1126/science.1132939 17008526

[33] Szklarczyk D Gable AL Lyon D Junge A Wyder S Huerta-Cepas J. String V11: protein-protein association networks with increased coverage, supporting functional discovery in genome-wide experimental datasets. Nucleic Acids Res. (2018) 47(D1):D607–D13. doi: 10.1093/nar/gky1131 PMC6323986 30476243

[34] Jan G Doncheva NT Morris JH Gorodkin J Jensen LJ. Cytoscape stringapp: network analysis and visualization of proteomics data. J Proteome Res. (2019) 18:623–32. doi: 10.1021/acs.jproteome.8b00702 PMC6800166 30450911

[35] Felicetti F Errico MC Bottero L Segnalini P Stoppacciaro A Biffoni M. The promyelocytic leukemia zinc finger-microrna-221/-222 pathway controls melanoma progression through multiple oncogenic mechanisms. Cancer Res. (2008) 68:2745–54. doi: 10.1158/0008-5472.Can-07-2538 18417445

[36] Errico MC Felicetti F Bottero L Mattia G Boe A Felli N. The abrogation of the hoxb7/pbx2 complex induces apoptosis in melanoma through the mir-221&222-C-fos pathway. Int J Cancer. (2013) 133:879–92. doi: 10.1002/ijc.28097 PMC3812682 23400877

[37] Kang TG Lan X Mi T Chen H Alli S Lim SE. Epigenetic regulators of clonal hematopoiesis control cd8 T cell stemness during immunotherapy. Science. (2024) 386:eadl4492. doi: 10.1126/science.adl4492 39388542 PMC11697317

[38] Thorsson V Gibbs DL Brown SD Wolf D Bortone DS Yang T-HO. The immune landscape of cancer. Immunity. (2018) 48:812–30.e14. doi: 10.1016/j.immuni.2018.03.023 29628290 PMC5982584

[39] Li L Wang Y Song G Zhang X Gao S Liu H. Hox cluster-embedded antisense long non-coding rnas in lung cancer. Cancer Lett. (2019) 450:14–21. doi: 10.1016/j.canlet.2019.02.036 30807784

[40] Kosuke Y Maria S Emmanuel M Rahulsimham V Hoon K Wandaliz T-G. Inferring tumour purity and stromal and immune cell admixture from expression data. Nat Commun. (2013) 4:2612. doi: 10.1038/ncomms3612 24113773 PMC3826632

[41] Malta TM Sokolov A Gentles AJ Burzykowski T Poisson L Weinstein JN. Machine learning identifies stemness features associated with oncogenic dedifferentiation. Cell. (2018) 173:338–54.e15. doi: 10.1016/j.cell.2018.03.034 29625051 PMC5902191

[42] Subramanian A Narayan R Corsello SM Peck DD Natoli TE Lu X. A next generation connectivity map: L1000 platform and the first 1,000,000 profiles. Cell. (2017) 171:1437–52.e17. doi: 10.1016/j.cell.2017.10.049 29195078 PMC5990023

[43] Xiaolei L Xiao S Rui L Yongsha P Jiankai F Lijuan C. Hdac inhibition potentiates anti-tumor activity of macrophages and enhances anti-pd-L1-mediated tumor suppression. Oncogene. (2021) 40:1836–50. doi: 10.1038/s41388-020-01636-x PMC7946638 33564072

[44] Wang B Ma Q Wang X Guo K Liu Z Li G. Tgif1 overexpression promotes glioma progression and worsens patient prognosis. Cancer Med. (2022) 11:5113–28. doi: 10.1002/cam4.4822 PMC9761070 35569122

[45] Yang H Zhan Q Wan YJ. Enrichment of nur77 mediated by retinoic acid receptor B Leads to apoptosis of human hepatocellular carcinoma cells induced by fenretinide and histone deacetylase inhibitors. Hepatol (Baltimore Md). (2011) 53:865–74. doi: 10.1002/hep.24101 PMC3077573 21319187

[46] Liu X Xu W Li L Zhang Z Lu M Xia X. Dual pi3k/mtor inhibitor bez235 combined with bms-1166 promoting apoptosis in colorectal cancer. Int J Med Sci. (2024) 21:1814–23. doi: 10.7150/ijms.84320 PMC11302559 39113885

[47] Rodriguez-Canales J Parra-Cuentas E Wistuba II. Diagnosis and molecular classification of lung cancer. In: Reckamp KL, editor. Lung Cancer: Treatment and Research. Springer International Publishing, Cham (2016). p. 25–46. 10.1007/978-3-319-40389-2_2 27535388

[48] Dela Cruz CS Tanoue LT Matthay RA. Lung cancer: epidemiology, etiology, and prevention. Clin Chest Med. (2011) 32(4):605–44. doi: 10.1016/j.ccm.2011.09.001 PMC3864624 22054876

[49] Palmeri M Mehnert J Silk AW Jabbour SK Ganesan S Popli P. Real-world application of tumor mutational burden-high (Tmb-high) and microsatellite instability (Msi) confirms their utility as immunotherapy biomarkers. ESMO Open. (2021) 7:100336. doi: 10.1016/j.esmoop.2021.100336 34953399 PMC8717431

[50] Yoshinari A Hideaki I Kazuhiko K. Pd-1 blockade in tumors with mismatch-repair deficiency. New Engl J Med. (2015) 373:2509–20. doi: 10.1056/NEJMoa1500596 PMC4481136 26028255

[51] Chen B Khodadoust MS Liu CL Newman AM Alizadeh AA. Profiling tumor infiltrating immune cells with cibersort. Methods Mol Biol (Clifton NJ). (2018) 1711:243–59. doi: 10.1007/978-1-4939-7493-1_12 PMC5895181 29344893

[52] Veiga RN de Oliveira JC Gradia DF. Pbx1: A key character of the hallmarks of cancer. J Mol Med (Berl). (2021) 99:1667–80. doi: 10.1007/s00109-021-02139-2 34529123

[53] Lyu XY Shui YS Wang L Jiang QS Meng LX Zhan HY. Wdr5 promotes the tumorigenesis of oral squamous cell carcinoma via carm1/B-catenin axis. Odontology. (2022) 110(1):138–47. doi: 10.1007/s10266-021-00649-6 34398317

[54] Castiglioni S Di Fede E Bernardelli C Lettieri A Parodi C Grazioli P. Kmt2a: umbrella gene for multiple diseases. Genes. (2022) 13(3):514. doi: 10.3390/genes13030514 35328068 PMC8949091

[55] Zhou H Bao J Zhu X Dai G Jiang X Jiao X. Retinoblastoma binding protein 5 correlates with the progression in hepatocellular carcinoma. Biomed Res Int. (2018) 2018:1–9. doi: 10.1155/2018/1073432 PMC6247687 30533424

[56] Naokazu C Shigeto O Takahiro G Toshimichi K Toru S Koichi T. Hoxb9 mediates resistance to chemotherapy and patient outcomes through the tgfβ Pathway in pancreatic cancer. Oncotarget. (2022) 13:747–54. doi: 10.18632/oncotarget.28235 PMC9132260 35634239

[57] Sangeetha SU Richard M Keith H Prasada KS Raghu R. Integrated computational analysis reveals hox genes cluster as oncogenic drivers in head and neck squamous cell carcinoma. Sci Rep. (2022) 12:7952. doi: 10.1038/s41598-022-11590-1 35562533 PMC9106698

[58] Li G RongQuan H ZhiGuang H GuoSheng L JiangHui Z JiaYin H. Expression landscape and functional roles of hoxa4 and hoxa5 in lung adenocarcinoma. Int J Med Sci. (2022) 19:572–87. doi: 10.7150/ijms.70445 PMC8964330 35370463

[59] Kumar BD Darland DC. The hox protein conundrum: the “Specifics” of DNA binding for hox proteins and their partners. Dev Biol. (2021) 477:284–92. doi: 10.1016/j.ydbio.2021.06.002 PMC8846413 34102167

[60] Liu Y Lin C Wang D Wen S Wang J Chen Z. Microrna-224 promotes cell migration and invasion by targeting hoxa5 expression in hepatocellular carcinoma. bioRxiv 2020.08.27.269654. (2021). doi: 10.1101/2020.08.27.269654

[61] Fan F Haoyang M Hao Z Ziyu D Zeyu W Chunrun Q. Hoxa5: A crucial transcriptional factor in cancer and a potential therapeutic target. Biomed Pharmacother. (2022) 155:113800. doi: 10.1016/j.biopha.2022.113800 36271576

[62] Yun-Bao G Yi-Meng S Jing C Song-Bai X Xing-Dong Z Mao-Ren W. Effect of overexpression of hox genes on its invasive tendency in cerebral glioma. Oncol Lett. (2016) 11:75–80. doi: 10.3892/ol.2015.3893 26870170 PMC4727036

[63] Schwab CL English DP Roque DM Pasternak M Santin AD. Past, present and future targets for immunotherapy in ovarian cancer. Immunotherapy. (2014) 6:1279–93. doi: 10.2217/imt.14.90 PMC4312614 25524384

[64] Sadeghi Rad H Monkman J Warkiani ME Ladwa R O’Byrne K Rezaei N. Understanding the tumor microenvironment for effective immunotherapy. Medicinal Res Rev. (2021) 41:1474–1498. doi: 10.1002/med.21765 PMC8247330 33277742

[65] Yi C Wei W Wan M Chen Y Zhang B Wu W. Expression patterns of hox gene family defines tumor microenvironment and immunotherapy in hepatocellular carcinoma. Appl Biochem Biotechnol. (2023) 195:5072–93. doi: 10.1007/s12010-023-04443-8 36976502

[66] Greten TF Wang XW Korangy F. Current concepts of immune based treatments for patients with hcc: from basic science to novel treatment approaches. Gut. (2015) 64:842–8. doi: 10.1136/gutjnl-2014-307990 PMC6311419 25666193

[67] Zhang H Jiang Z Zhang L. Dual effect of T helper cell 17 (Th17) and regulatory T cell (Treg) in liver pathological process: from occurrence to end stage of disease. Int Immunopharmacol. (2019) 69:50–9. doi: 10.1016/j.intimp.2019.01.005 30669025

[68] Sonnenberg GF Hepworth MR. Functional interactions between innate lymphoid cells and adaptive immunity. Nat Rev Immunol. (2019) 19:599–613. doi: 10.1038/s41577-019-0194-8 31350531 PMC6982279

[69] Murray PJ Allen JE Biswas SK Fisher EA Gilroy DW Goerdt S. Macrophage activation and polarization: nomenclature and experimental guidelines. Immunity. (2014) 41:14–20. doi: 10.1016/j.immuni.2014.06.008 25035950 PMC4123412

[70] Chen W Chaogang Y Shuyi W Dongdong S Chunxiao Z Xiaobin L. Crosstalk between cancer cells and tumor associated macrophages is required for mesenchymal circulating tumor cell-mediated colorectal cancer metastasis. Mol Cancer. (2019) 18:64. doi: 10.1186/s12943-019-0976-4 30927925 PMC6441214

[71] Yiqing T Ran S Lei L Dejuan Y Qin X Li L. Tumor suppressor drd2 facilitates M1 macrophages and restricts nf-Kb signaling to trigger pyroptosis in breast cancer. Theranostics. (2021) 11:5214–31. doi: 10.7150/thno.58322 PMC8039962 33859743

[72] Yueyun P Yinda Y Xiaojian W Ting Z. Tumor-associated macrophages in tumor immunity. Front Immunol. (2020) 11:583084. doi: 10.3389/fimmu.2020.583084 33365025 PMC7751482

[73] Shao W Ding Q Guo Y Xing J Huo Z Wang Z. A pan-cancer landscape of hox-related lncrnas and their association with prognosis and tumor microenvironment. Front Mol Biosci. (2021) 8:767856. doi: 10.3389/fmolb.2021.767856 34805277 PMC8602076

[74] Chen H Lin Z Arnst KE Miller DD Li W. Tubulin inhibitor-based antibody-drug conjugates for cancer therapy. Molecules. (2017) 22:1281. doi: 10.3390/molecules22081281 28763044 PMC6152078

[75] Wei Z Jia-Ke B Hong-Mei L Tao C Ya-Jie T. Tubulin structure-based drug design for the development of novel 4β-sulfur-substituted podophyllum tubulin inhibitors with anti-tumor activity. Sci Rep. (2015) 5:10172. doi: 10.1038/srep10172 25959922 PMC4426677

[76] Hui Y Liangbin L Zhiqiang Z Huiyuan Z Hongbo H. Targeting nf-Kb pathway for the therapy of diseases: mechanism and clinical study. Signal Transduct Targeted Ther. (2020) 5:209. doi: 10.1038/s41392-020-00312-6 PMC7506548 32958760

[77] Jürgen R. Return to homeostasis: downregulation of nf-Kb responses. Nat Immunol. (2011) 12:709–14. doi: 10.1038/ni.2055 21772279

[78] Shi R Wang X Wu Y Xu B Zhao T Trapp C. Apobec-mediated mutagenesis is a favorable predictor of prognosis and immunotherapy for bladder cancer patients: evidence from pan-cancer analysis and multiple databases. Theranostics. (2022) 12:4181. doi: 10.7150/thno.73235 35673559 PMC9169361

[79] Nuciforo P Pascual T Cortés J Llombart-Cussac A Fasani R Paré L. A predictive model of pathologic response based on tumor cellularity and tumor-infiltrating lymphocytes (Celtil) in her2-positive breast cancer treated with chemo-free dual her2 blockade. Ann Oncol Off J Eur Soc Med Oncol. (2018) 29(1):P170–177. doi: 10.1093/annonc/mdx647 29045543

[80] Ma Q Chen Y Qin Q Guo F Wang Y-s Li D. Cxcl13 expression in mouse 4t1 breast cancer microenvironment elicits antitumor immune response by regulating immune cell infiltration. Precis Clin Med. (2021) 4:155–67. doi: 10.1093/pcmedi/pbab020 PMC8982548 35693216

[81] Liu C Wang X Wang S Xiang J Xie H Tan Z. Comprehensive analysis of P2y family genes expression, immune characteristics, and prognosis in pan-cancer. Trans Oncol. (2023) 37:101776. doi: 10.1016/j.tranon.2023.101776 PMC10485639 37672858

[82] Liu S Jin K Hui Y Fu J Jie C Feng S. Hoxb7 promotes Malignant progression by activating the tgfβ Signaling pathway. Cancer Res. (2015) 75:709–19. doi: 10.1158/0008-5472.Can-14-3100 PMC4352303 25542862

[83] Wu S Zhu D Feng H Li Y Zhou J Li Y. Comprehensive analysis of hoxc8 associated with tumor microenvironment characteristics in colorectal cancer. Heliyon. (2023) 9:e21346. doi: 10.1016/j.heliyon.2023.e21346 37885723 PMC10598528

[84] Tandon I Sharma NK. Macrophage flipping from foe to friend: A matter of interest in breast carcinoma heterogeneity driving drug resistance. Curr Cancer Drug Targets. (2019) 19:189–98. doi: 10.2174/1568009618666180628102247 29952260

[85] Watson MJ Vignali PDA Mullett SJ Overacre-Delgoffe AE Peralta RM Grebinoski S. Metabolic support of tumour-infiltrating regulatory T cells by lactic acid. Nature. (2021) 591:645–51. doi: 10.1038/s41586-020-03045-2 PMC7990682 33589820

