# Supplementary material for: A pan-cancer analysis of homeobox family: expression characteristics and latent significance in prognosis and immune microenvironment
Source: Front Oncol. 2025 Feb 6;15:1521652. doi: 10.3389/fonc.2025.1521652 (PMC11840236; doi:10.3389/fonc.2025.1521652)
Supplement: Supplementary Figure 1 — Mutations of each HOX gene in 32 cancers (TCGA, PanCancer Atlas) by cBioportal. [file DataSheet1.zip › Suppl.files/Abbreviation.docx]

| **Abbreviation** | **Full name** |
| --- | --- |
| ACC | Adrenocortical carcinoma |
| BLCA | Bladder urothelial carcinoma |
| BRCA | Breast invasive carcinoma |
| CESC | Cervical squamous cell carcinoma and endocervical adenocarcinoma |
| CHOL | Cholangio carcinoma |
| COAD | Colon adenocarcinoma |
| DLBC | Lymphoid neoplasm diffuse large B-cell lymphoma |
| ESCA | Esophageal carcinoma |
| GBM | Glioblastoma multiforme |
| HNSC | Head and neck squamous cell carcinoma |
| KICH | Kidney chromophobe carcinoma |
| KIRC | Kidney renal clear cell carcinoma |
| KIRP | Kidney renal papillary cell carcinoma |
| LAML | Acute myeloid leukemia |
| LGG | Brain lower grade glioma |
| LIHC | Liver hepatocellular carcinoma |
| LUAD | Lung adenocarcinoma |
| LUSC | Lung squamous cell carcinoma |
| MESO | Mesothelioma |
| OV | Ovarian serous cystadenocarcinoma |
| PAAD | Pancreatic adenocarcinoma |
| PCPG | Pheochromocytoma and paraganglioma |
| PRAD | Prostate adenocarcinoma |
| READ | Rectum adenocarcinoma |
| SARC | Sarcoma |
| SKCM | Skin cutaneous melanoma |
| STAD | Stomach adenocarcinoma |
| TGCT | Testicular germ cell tumors |
| THCA | Thyroid carcinoma |
| THYM | Thymoma |
| UCEC | Uterine corpus endometrial carcinoma |
| UCS | Uterine carcinosarcoma |
| UVM | Uveal melanoma |
| HCC | Hepatocellular carcinoma |
| CRC | Colorectal Cancer |
| HOX | Homeobox |
| TCGA | The Cancer Genome Atlas |
| TME | Tumor microenvironment |
| CNA | Copy-number alteration |
| GDC | Genomic Data Commons |
| OS | Overall survival |
| DSS | Disease-specific survival |
| PFI | Progression-free interval |
| HR | Hazard ratio |
| TMB | Tumor mutation burden |
| MSI | Microsatellite instability |
| DNAss | DNA stemness score |
| RNAss | RNA stemness score |
| PPI | Protein-protein interaction |
| GO | Gene ontology |
| KEGG | Kyoto Encyclopedia of Genes and Genomes |
| IHC | Immunohistochemical staining |
| ATCC | American Type Culture Collection |
| CAFs | Cancer associated fibroblasts |
| cMap | The Connective map |
| SCLC | Small cell lung cancer |
| NSCLC | Non-small cell lung cancer |
| BP | Biological process |
| CC | Cellular component |
| MF | Molecular function |
| EMT | Epithelial-mesenchymal transition |
| MMR | Mismatch repair |
| NK | Natural killer cell |
| TAMs | Tumor-associated Macrophages |
| TILs | Tumor-infiltrating lymphocytes |
| Tregs | Regulatory T Cells |
|  |  |
