# Supplementary material for: A pan-cancer analysis of homeobox family: expression characteristics and latent significance in prognosis and immune microenvironment
Source: Front Oncol. 2025 Feb 6;15:1521652. doi: 10.3389/fonc.2025.1521652 (PMC11840236; doi:10.3389/fonc.2025.1521652)
Supplement: Supplementary Figure 1 — Mutations of each HOX gene in 32 cancers (TCGA, PanCancer Atlas) by cBioportal. [file DataSheet1.zip › Suppl.files/S2_File.pdf]

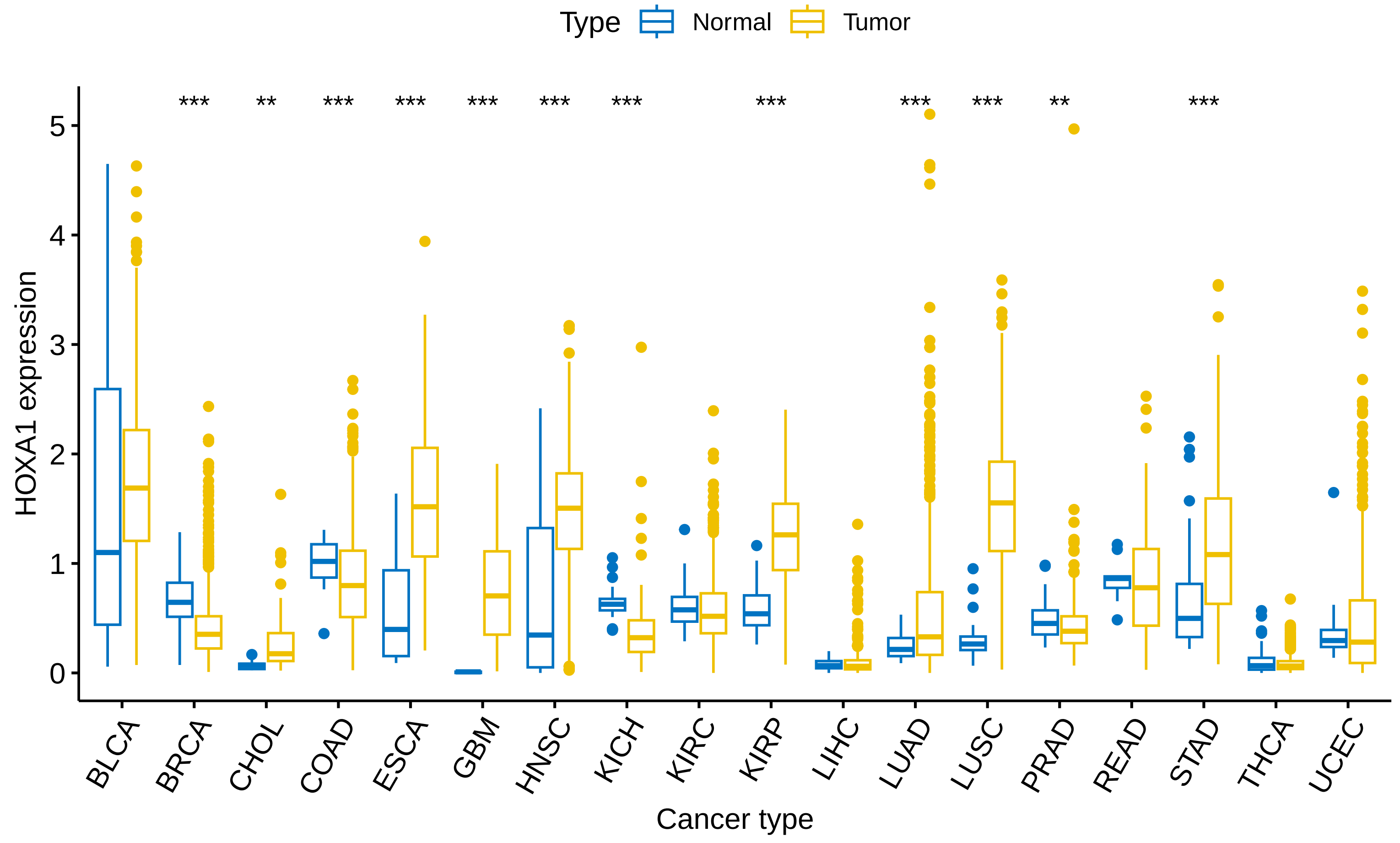

Type
Normal
Tumor
HOXA1 expression
0
1
2
3
4
5
*** ** *** *** *** *** *** *** *** *** ** ***
BLCA
BRCA
CHOL
COAD
ESCA
GBM
HNSC
KICH
KIRC
KIRP
LIHC
LUAD
LUSC
PRAD
READ
STAD
THCA
UCEC
Cancer type

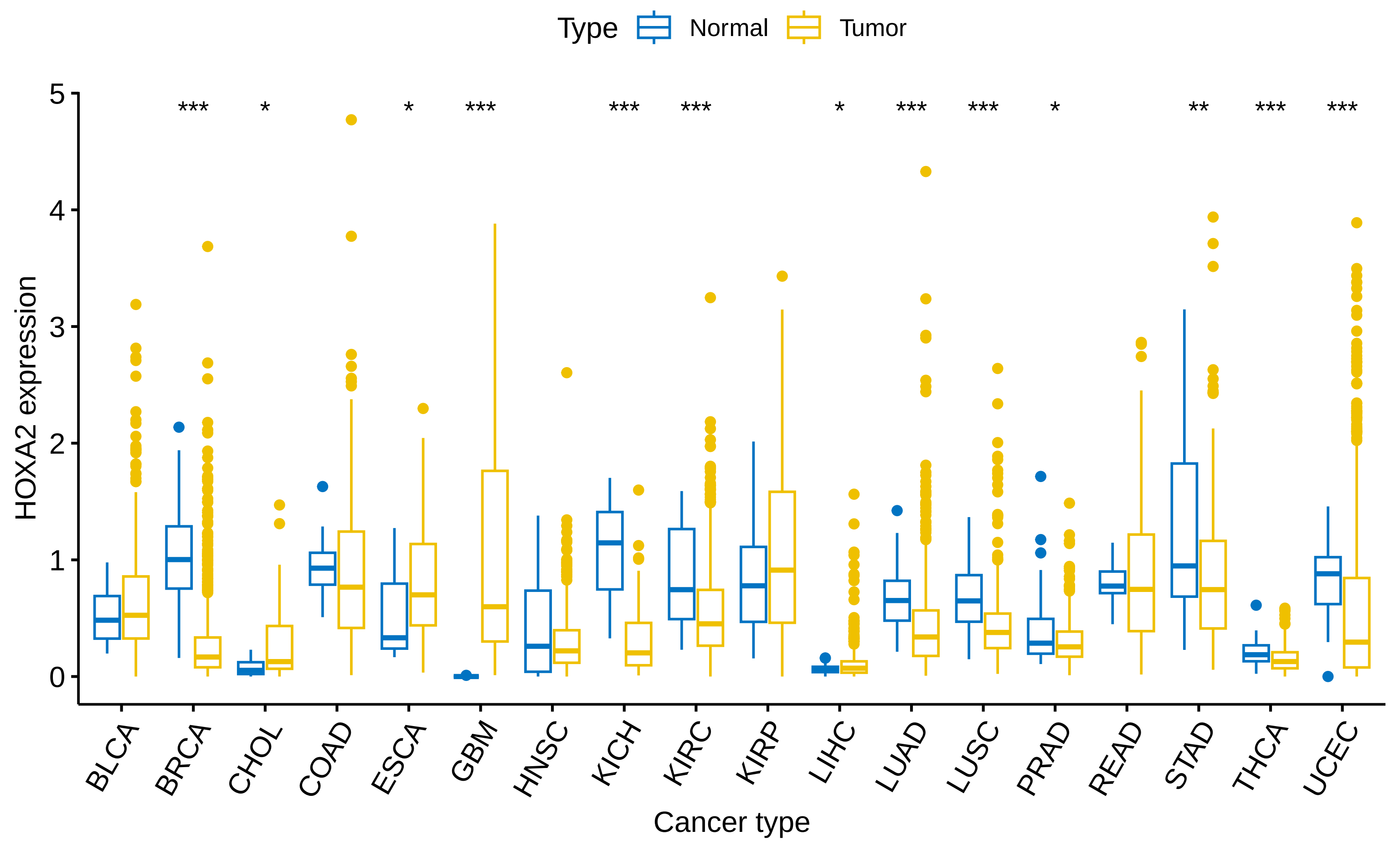

Type
Normal
Tumor
HOXA2 expression
5
4
3
2
1
0
***
*
*
***
***
***
*
***
***
*
**
***
***
BLCA
BRCA
CHOL
COAD
ESCA
GBM
HNSC
KICH
KIRC
KIRP
LIHC
LUAD
LUSC
PRAD
READ
STAD
THCA
UCEC
Cancer type

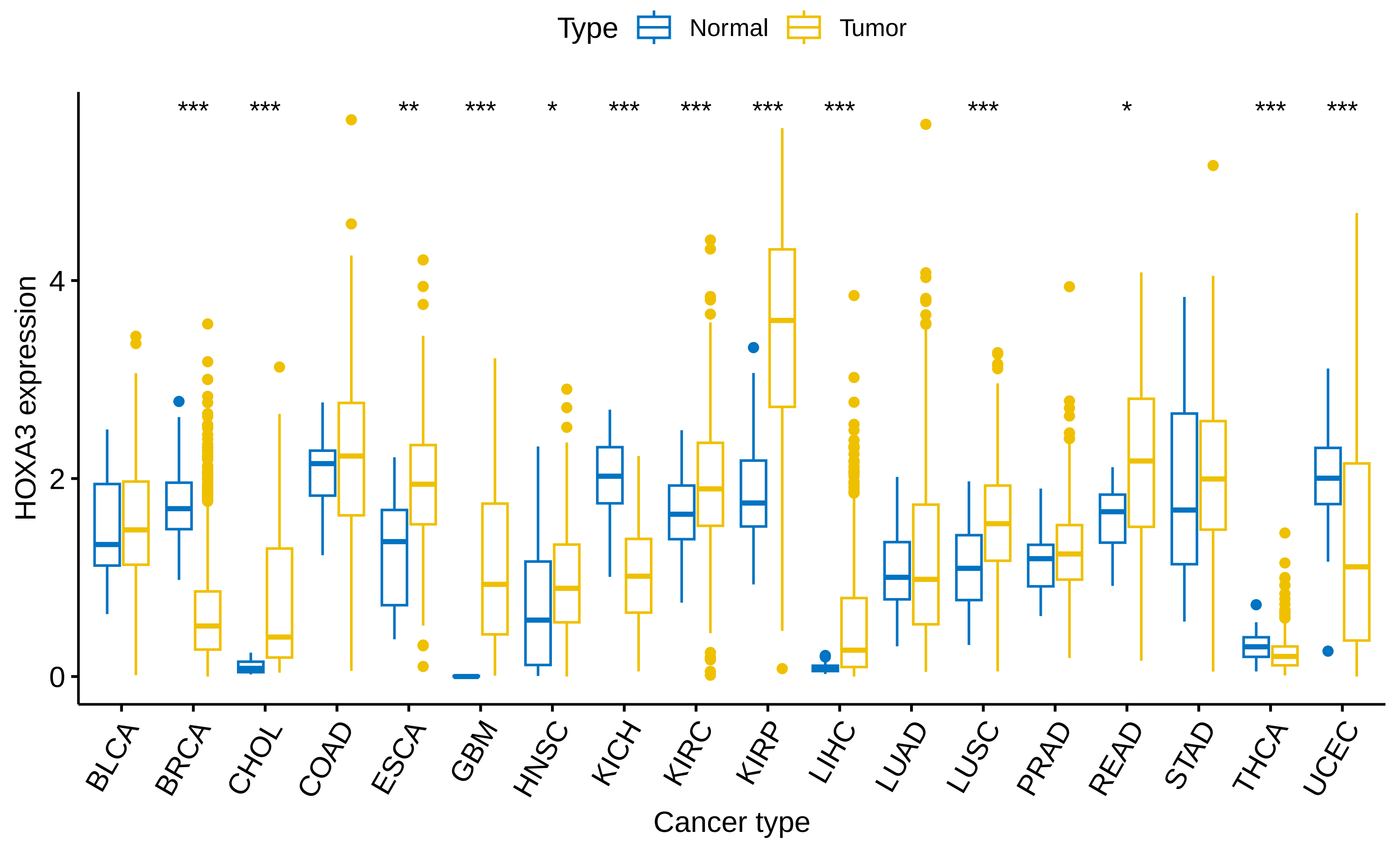
Type
Normal
Tumor
HOXA3 expression
4
2
0
***
***
**
***
*
***
***
***
***
***
*
***
***
BLCA
BRCA
CHOL
COAD
ESCA
GBM
HNSC
KICH
KIRC
KIRP
LIHC
LUAD
LUSC
PRAD
READ
STAD
THCA
UCEC
Cancer type

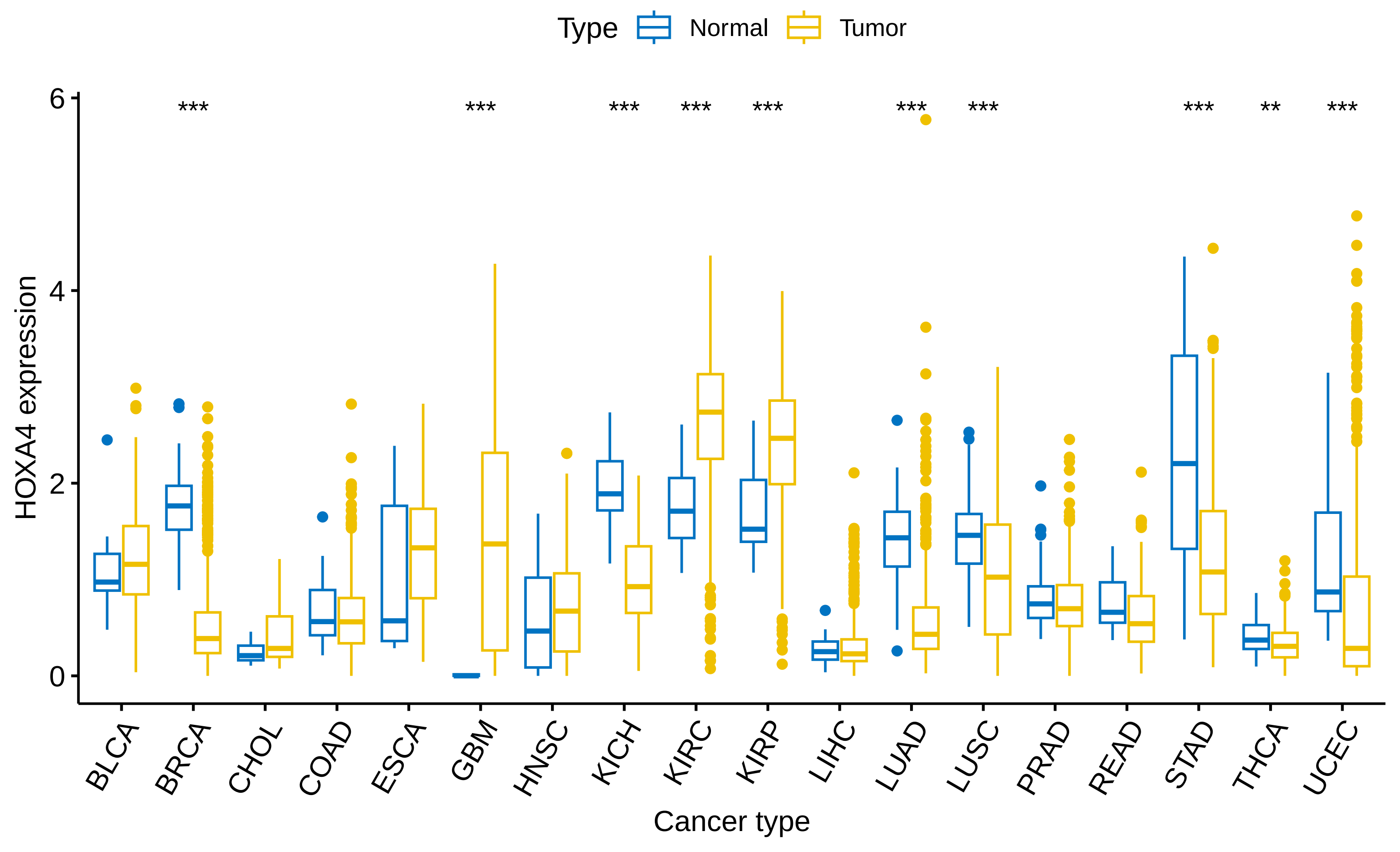

Type
Normal
Tumor
HOXA4 expression
6
4
2
0
***
***
***
***
***
***
***
***
**
***
BLCA
BRCA
CHOL
COAD
ESCA
GBM
HNSC
KICH
KIRC
KIRP
LIHC
LUAD
LUSC
PRAD
READ
STAD
THCA
UCEC
Cancer type

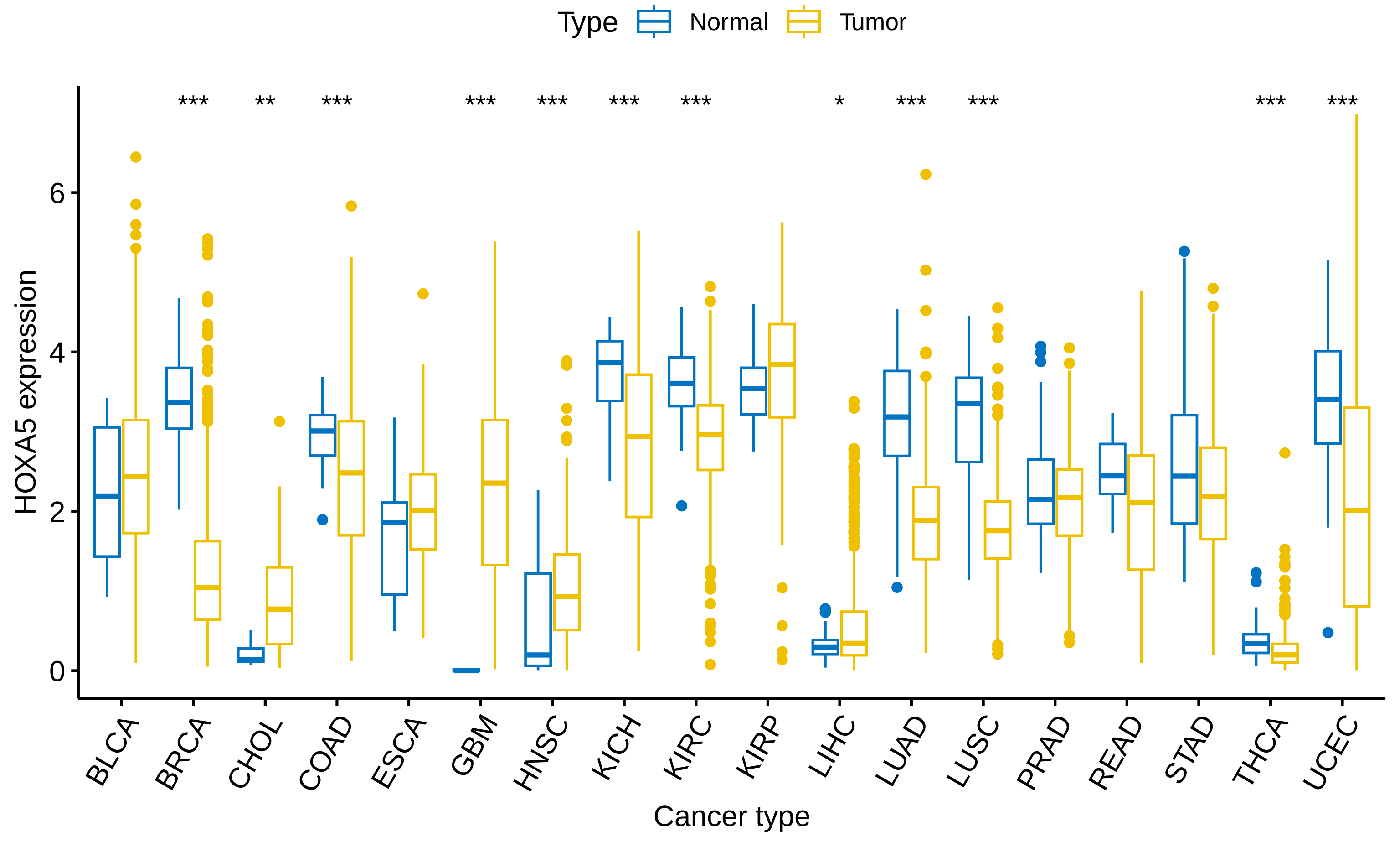

Type
Normal
Tumor
HOXA5 expression
0
2
4
6
*** ** *** *** *** *** *** * *** *** *** ***
BLCA
BRCA
CHOL
COAD
ESCA
GBM
HNSC
KICH
KIRC
KIRP
LIHC
LUAD
LUSC
PRAD
READ
STAD
THCA
UCEC
Cancer type

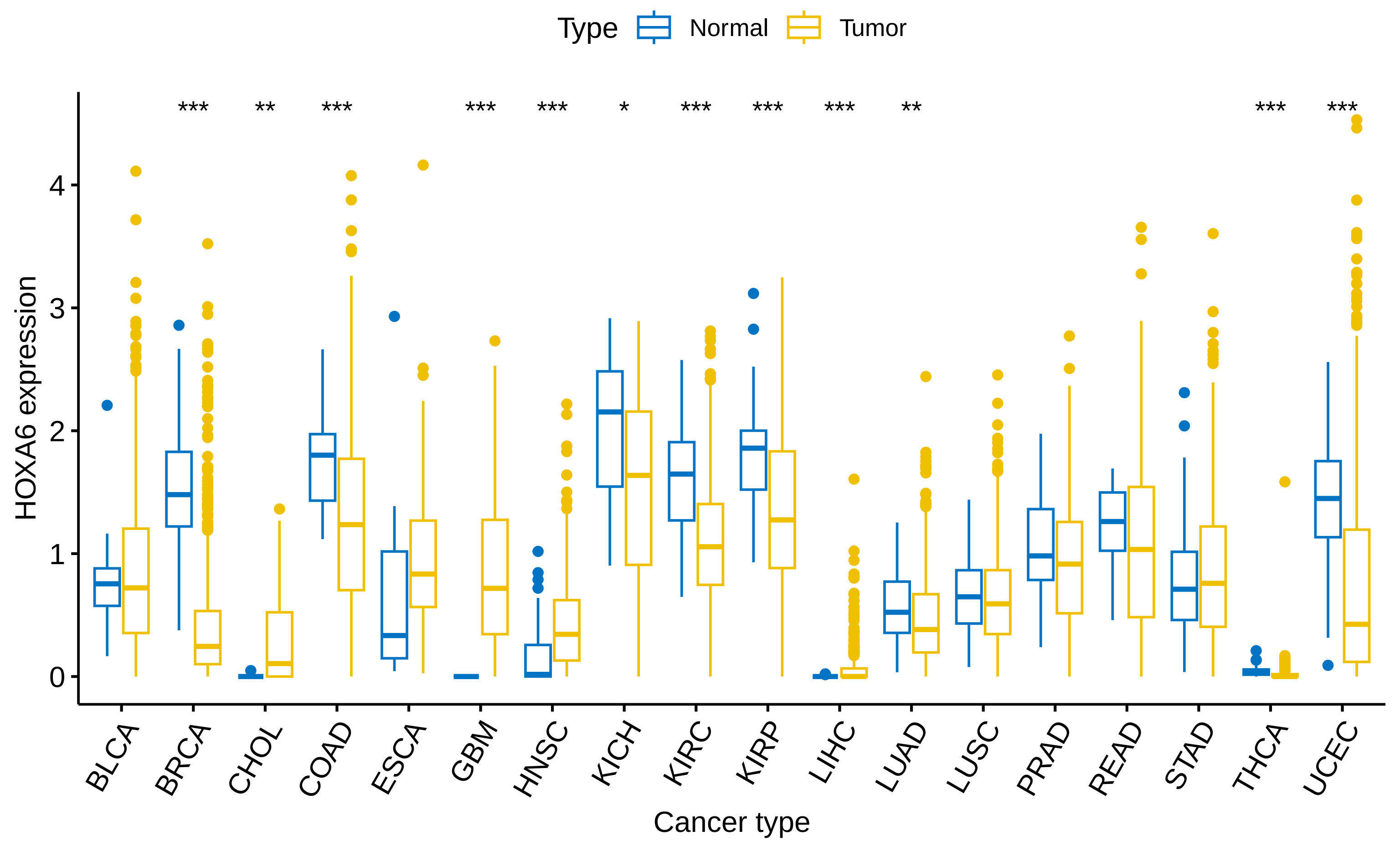
Type
Normal
Tumor
HOXA6 expression
0
1
2
3
4
***
**
***
***
***
*
***
***
***
**
***
***
BLCA
BRCA
CHOL
COAD
ESCA
GBM
HNSC
KICH
KIRC
KIRP
LIHC
LUAD
LUSC
PRAD
READ
STAD
THCA
UCEC
Cancer type

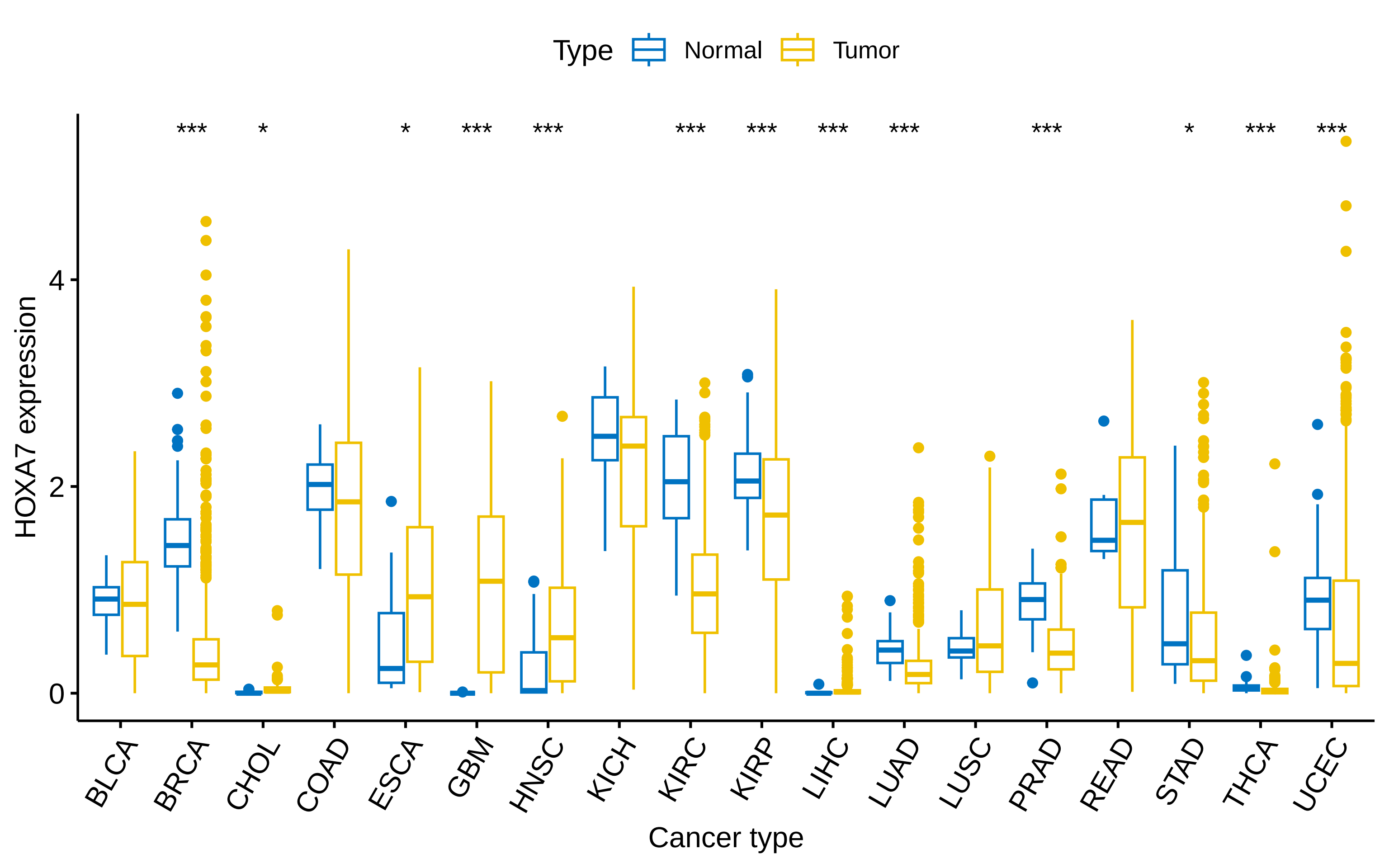

Type
Normal
Tumor
*** * * *** *** *** *** *** *** *** * *** ***
HOXA7 expression
0
2
4
BLCA
BRCA
CHOL
COAD
ESCA
GBM
HNSC
KICH
KIRC
KIRP
LIHC
LUAD
LUSC
PRAD
READ
STAD
THCA
UCEC
Cancer type

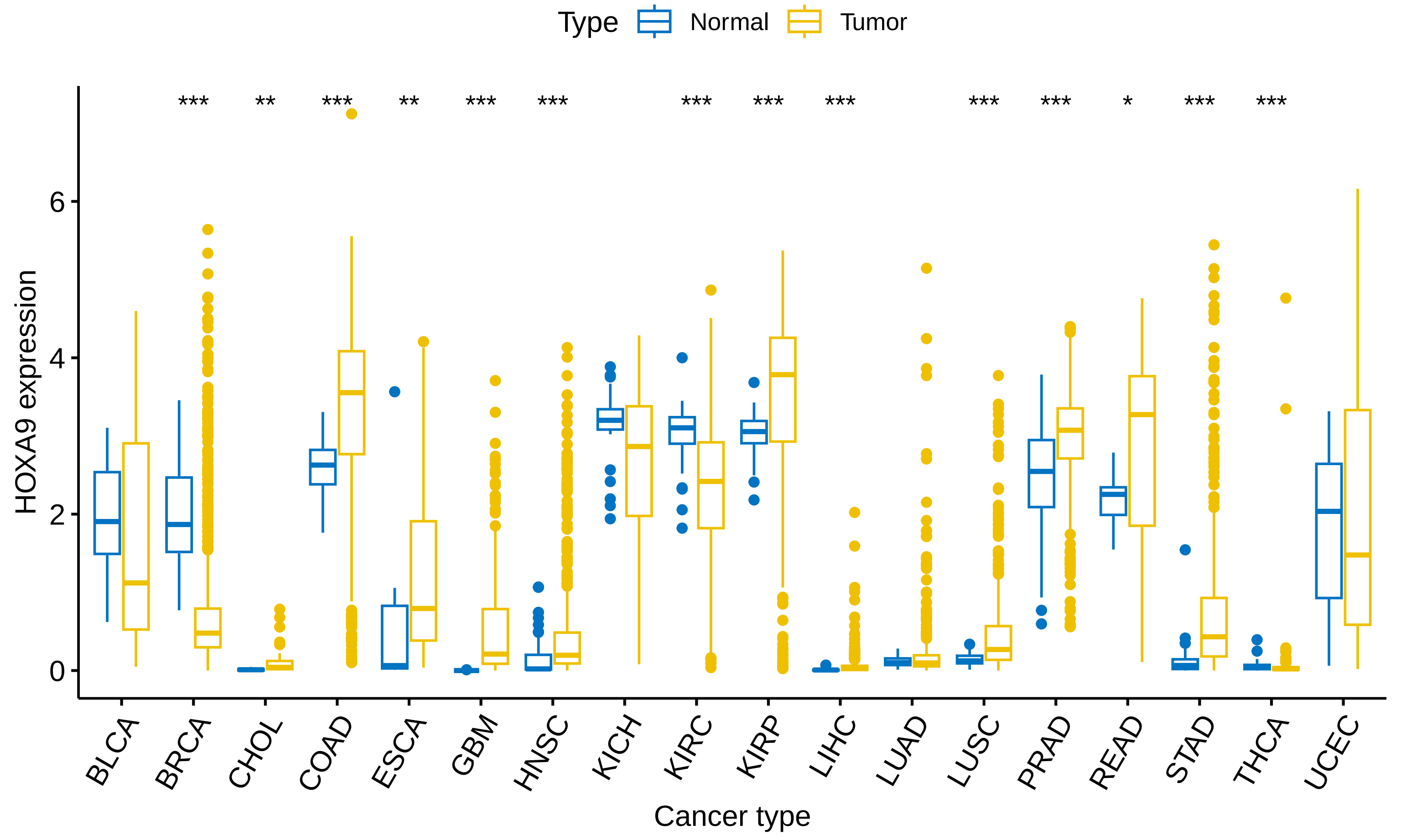

Type
Normal
Tumor
HOXA9 expression
0
2
4
6
*** ** *** ** *** *** *** *** *** *** *** * *** ***
BLCA
BRCA
CHOL
COAD
ESCA
GBM
HNSC
KICH
KIRC
KIRP
LIHC
LUAD
LUSC
PRAD
READ
STAD
THCA
UCEC
Cancer type

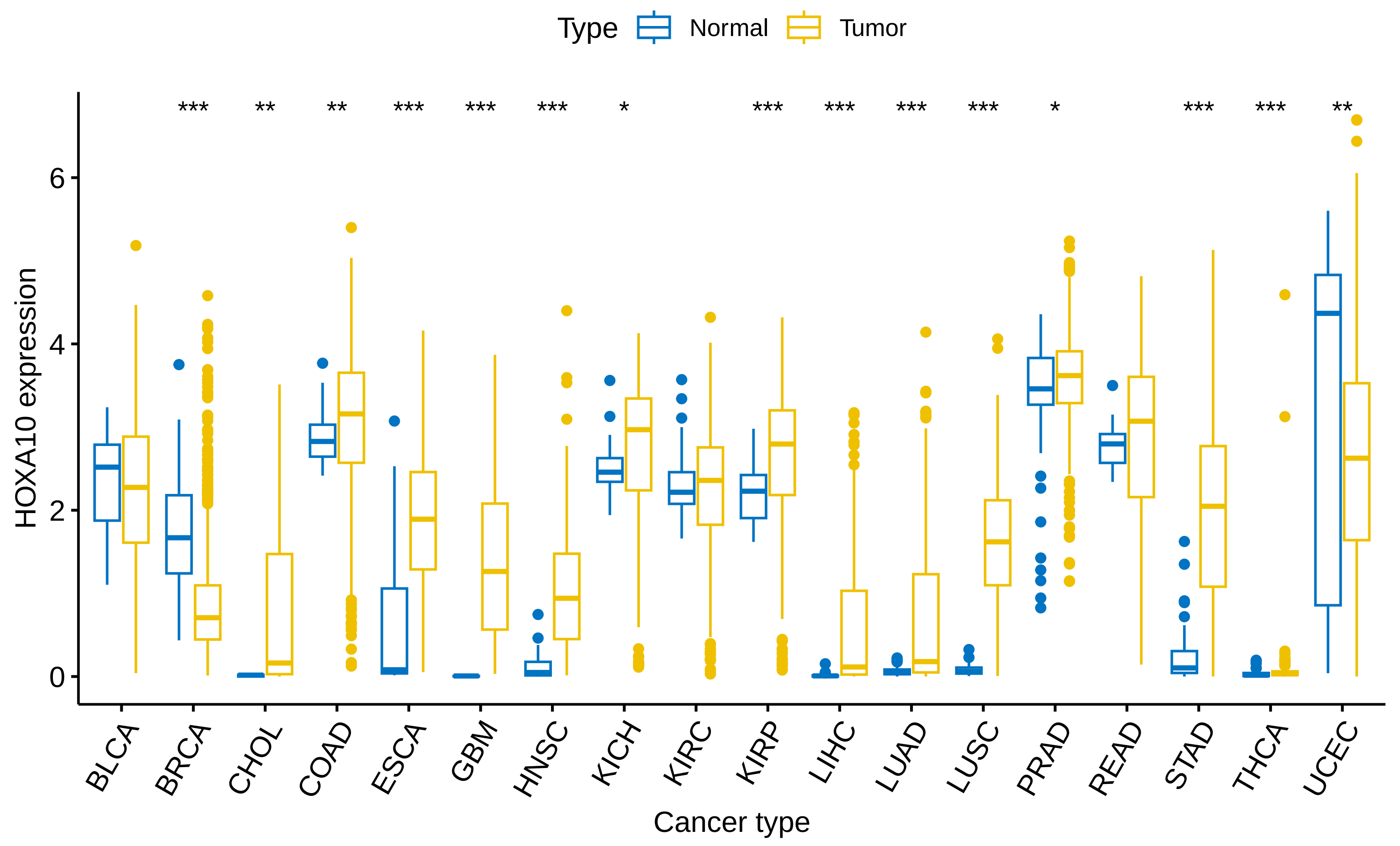

Type
Normal
Tumor
HOXA10 expression
6
4
2
0
*** ** ** *** *** *** * *** *** *** *** * *** *** **
BLCA
BRCA
CHOL
COAD
ESCA
GBM
HNSC
KICH
KIRC
KIRP
LIHC
LUAD
LUSC
PRAD
READ
STAD
THCA
UCEC
Cancer type

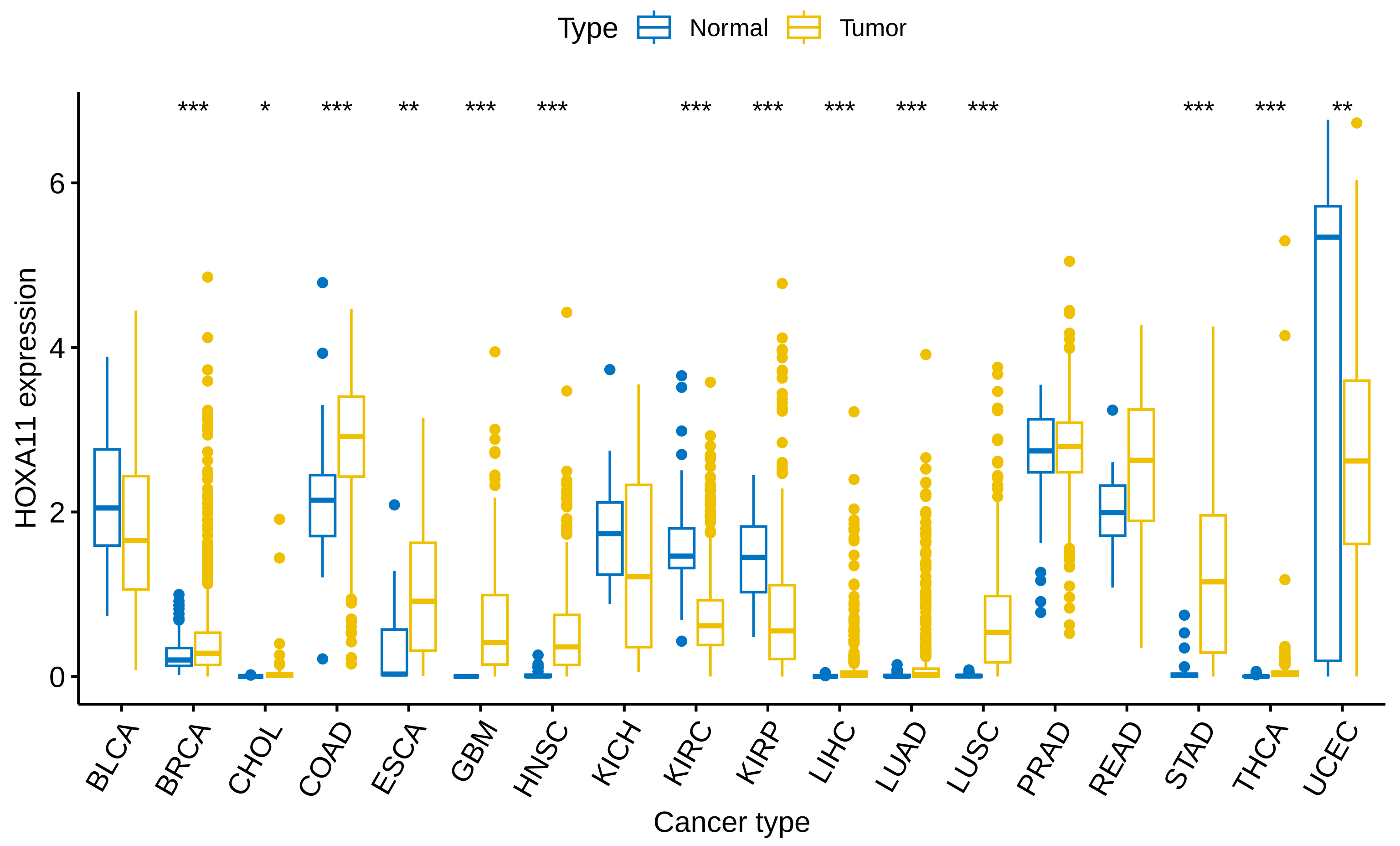

Type
Normal
Tumor
HOXA11 expression
0
2
4
6
*** * *** ** *** *** *** *** *** *** *** *** *** **
BLCA
BRCA
CHOL
COAD
ESCA
GBM
HNSC
KICH
KIRC
KIRP
LIHC
LUAD
LUSC
PRAD
READ
STAD
THCA
UCEC
Cancer type

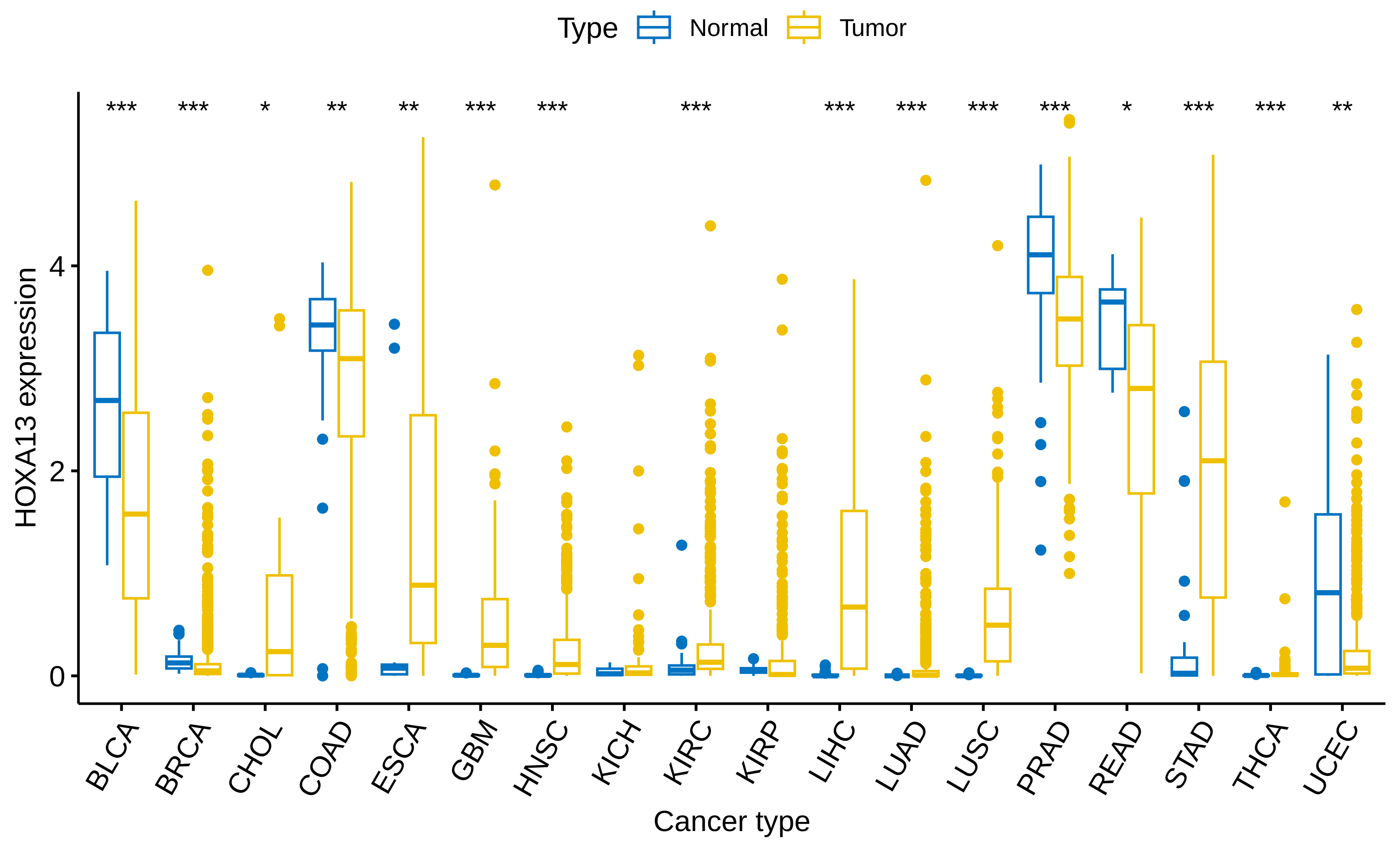
Type
Normal
Tumor
*** *** * ** ** *** *** *** *** *** *** *** * *** *** **
HOXA13 expression
0
2
4
BLCA
BRCA
CHOL
COAD
ESCA
GBM
HNSC
KICH
KIRC
KIRP
LIHC
LUAD
LUSC
PRAD
READ
STAD
THCA
UCEC
Cancer type

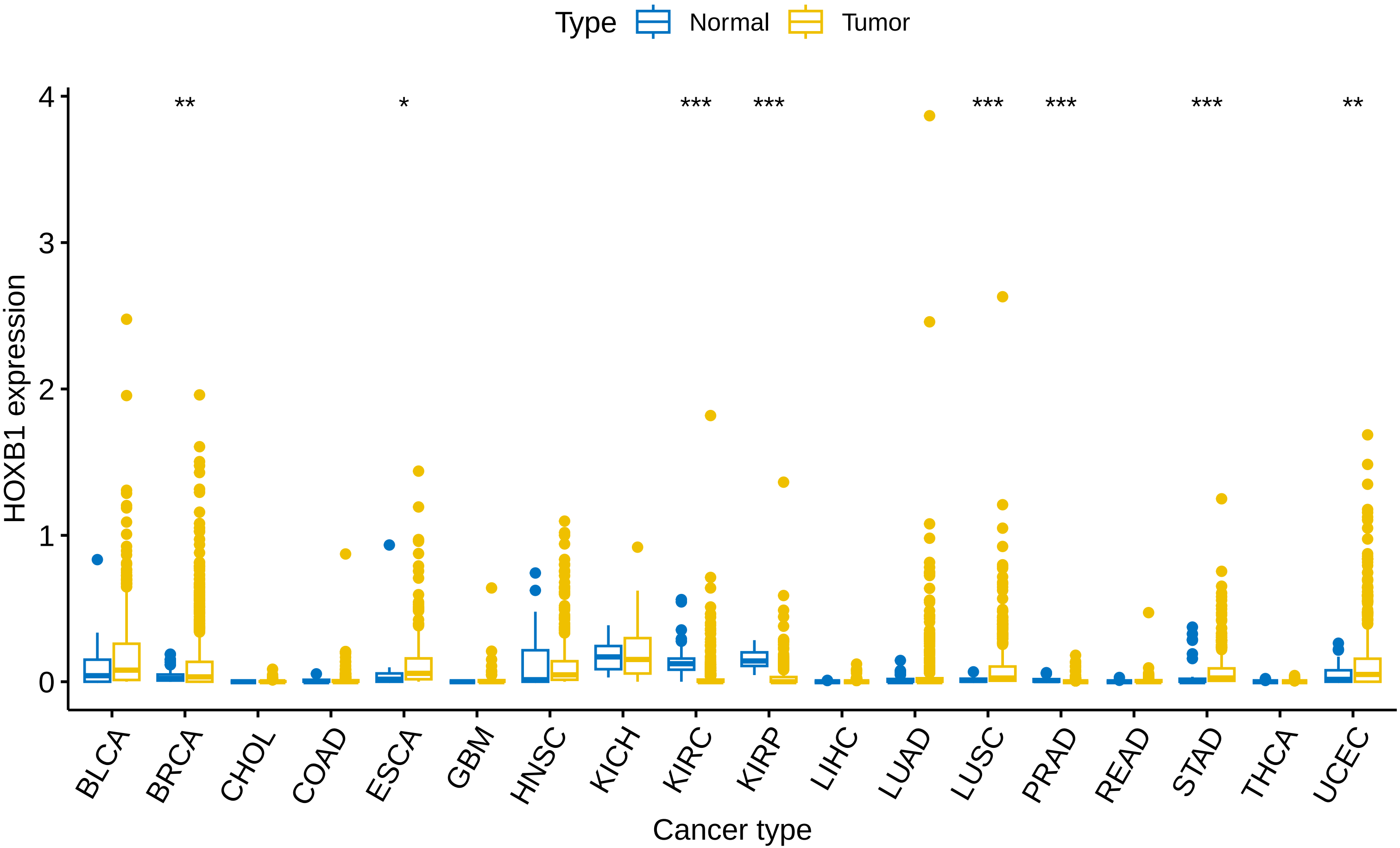

Type
Normal
Tumor
HOXB1 expression
0
1
2
3
4
**
*
***
***
***
***
***
**
BLCA
BRCA
CHOL
COAD
ESCA
GBM
HNSC
KICH
KIRC
KIRP
LIHC
LUAD
LUSC
PRAD
READ
STAD
THCA
UCEC
Cancer type

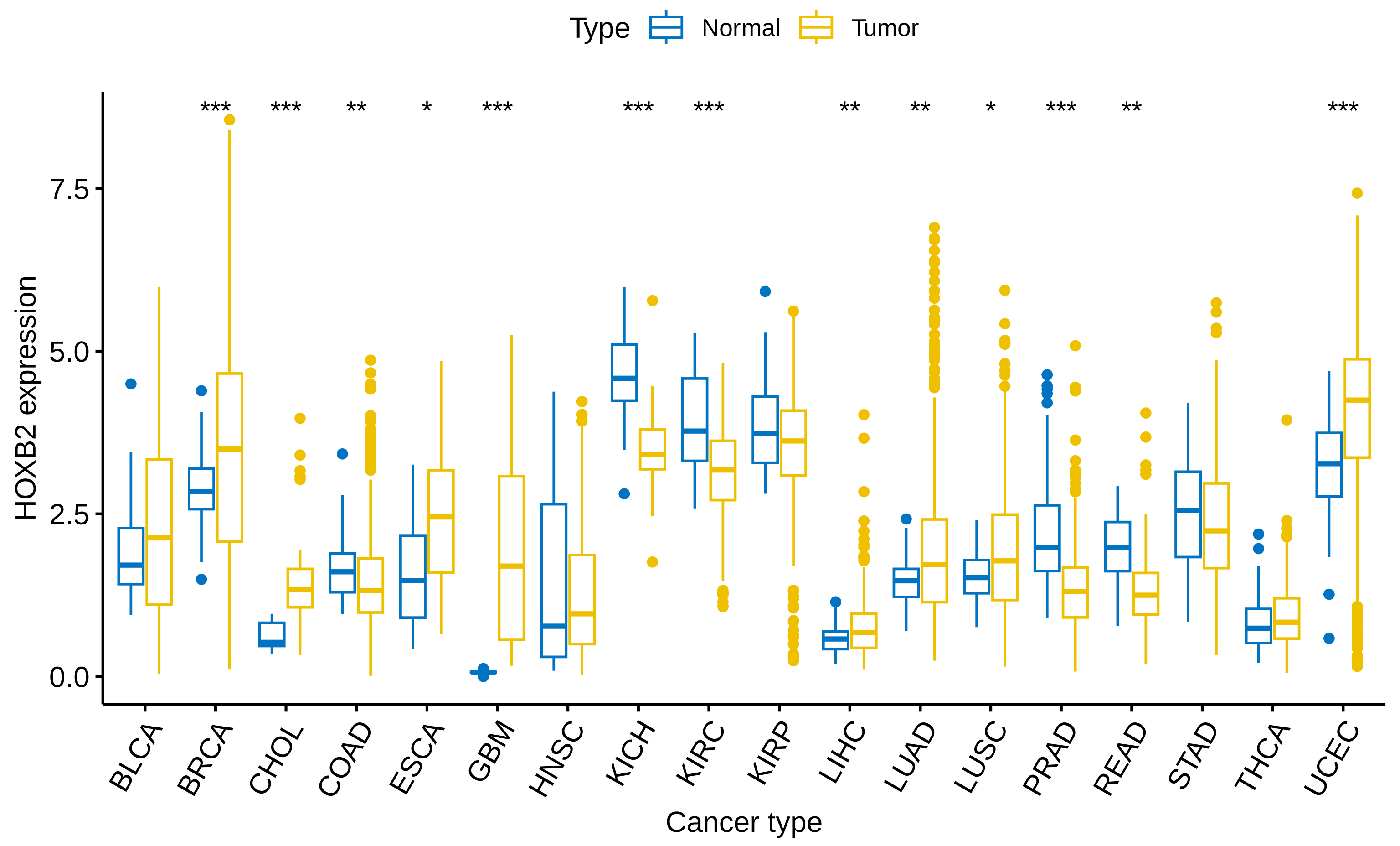

Type
Normal
Tumor
HOXB2 expression
7.5
5.0
2.5
0.0
***
***
**
*
***
***
***
**
**
*
***
**
***
BLCA
BRCA
CHOL
COAD
ESCA
GBM
HNSC
KICH
KIRC
KIRP
LIHC
LUAD
LUSC
PRAD
READ
STAD
THCA
UCEC
Cancer type

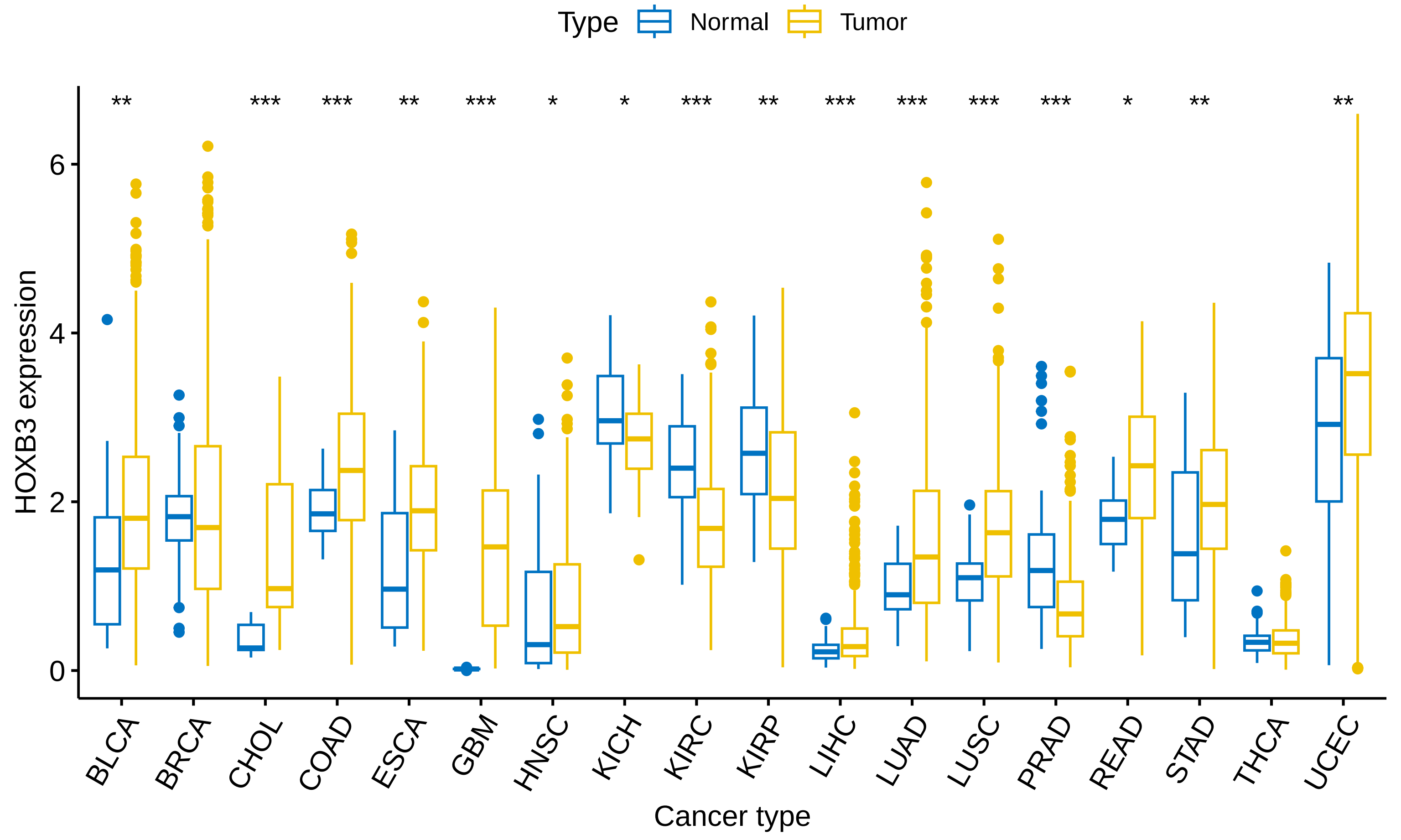

Type
Normal
Tumor
** *** *** ** *** * * *** ** *** *** *** *** * ** **
HOXB3 expression
0
2
4
6
BLCA
BRCA
CHOL
COAD
ESCA
GBM
HNSC
KICH
KIRC
KIRP
LIHC
LUAD
LUSC
PRAD
READ
STAD
THCA
UCEC
Cancer type

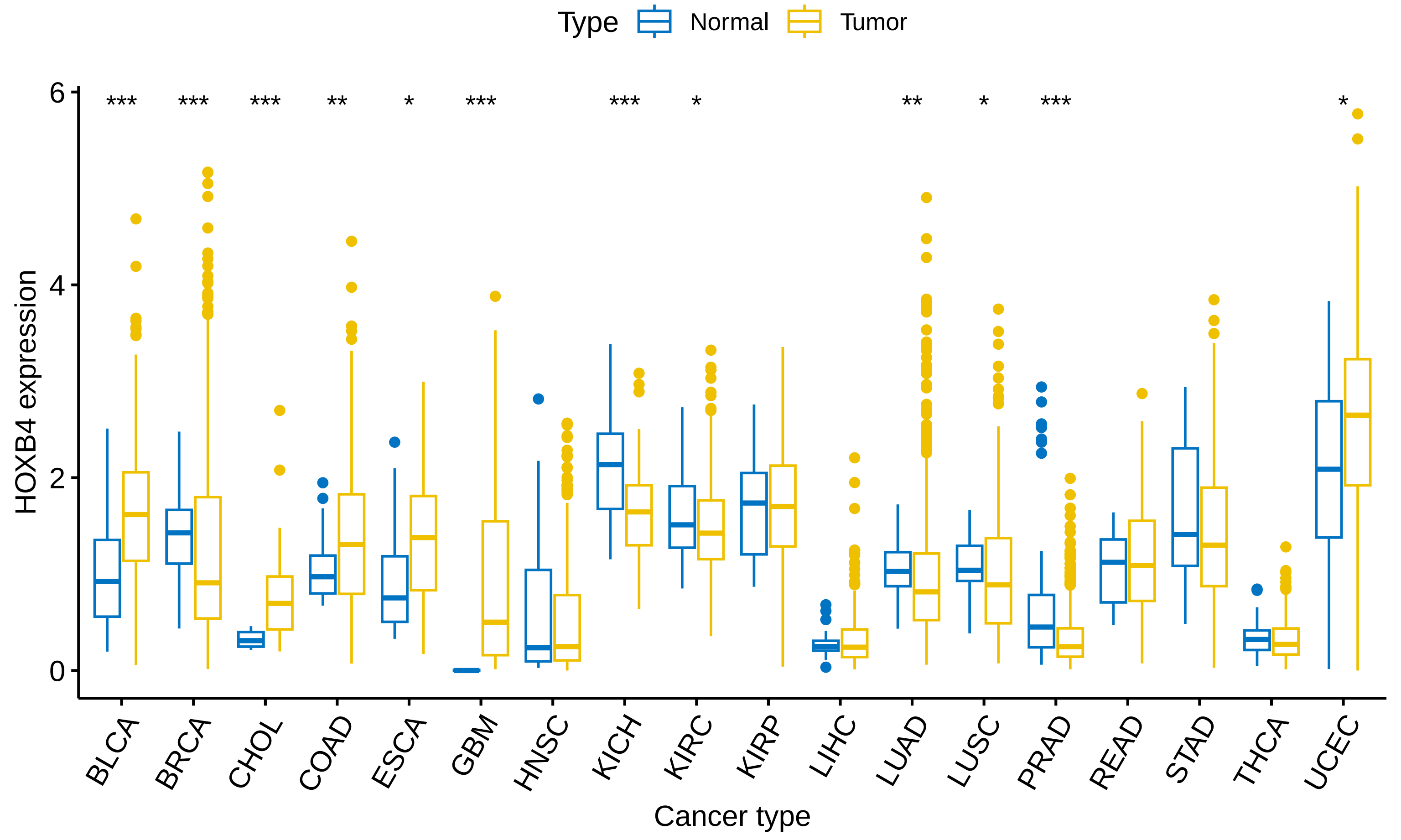

Type
Normal
Tumor
HOXB4 expression
0
2
4
6
*** *** *** ** * *** *** * ** * *** *
BLCA
BRCA
CHOL
COAD
ESCA
GBM
HNSC
KICH
KIRC
KIRP
LIHC
LUAD
LUSC
PRAD
READ
STAD
THCA
UCEC
Cancer type

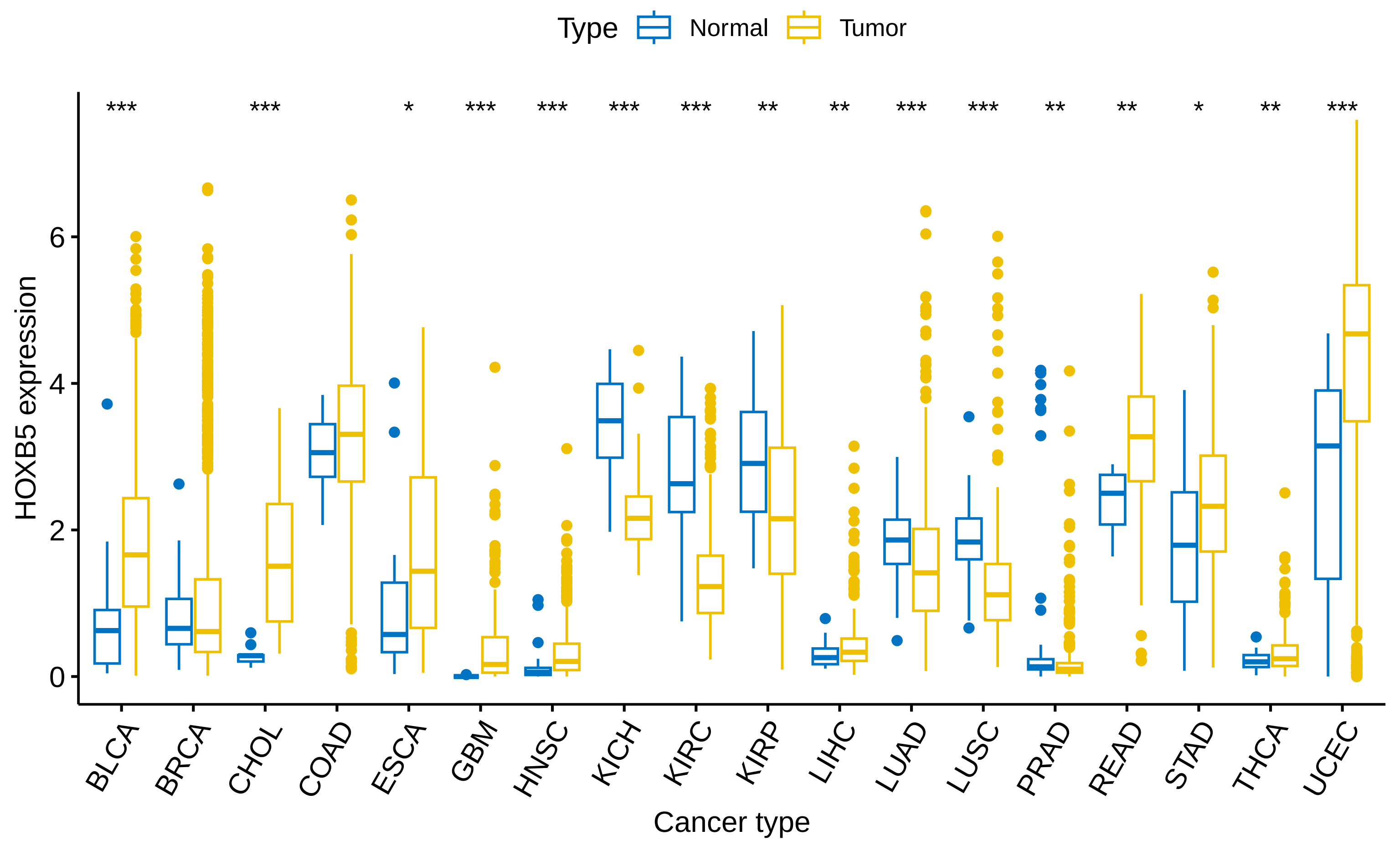

Type
Normal
Tumor
HOXB5 expression
0
2
4
6
*** *** * *** *** *** *** ** ** *** *** ** ** * ** ***
BLCA
BRCA
CHOL
COAD
ESCA
GBM
HNSC
KICH
KIRC
KIRP
LIHC
LUAD
LUSC
PRAD
READ
STAD
THCA
UCEC
Cancer type

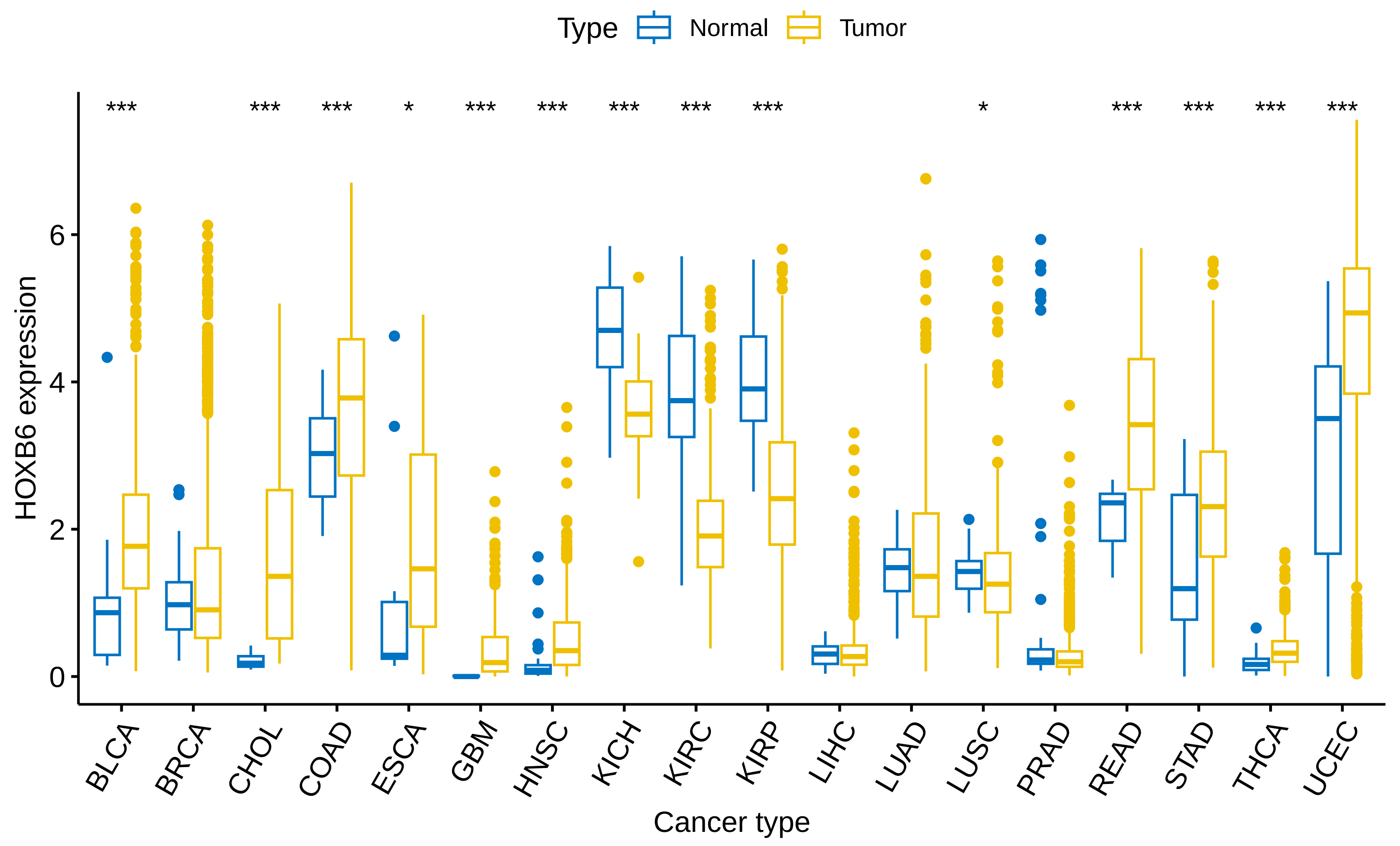

Type
Normal
Tumor
HOXB6 expression
0
2
4
6
***
***
***
*
***
***
***
***
***
*
***
***
***
***
BLCA
BRCA
CHOL
COAD
ESCA
GBM
HNSC
KICH
KIRC
KIRP
LIHC
LUAD
LUSC
PRAD
READ
STAD
THCA
UCEC
Cancer type

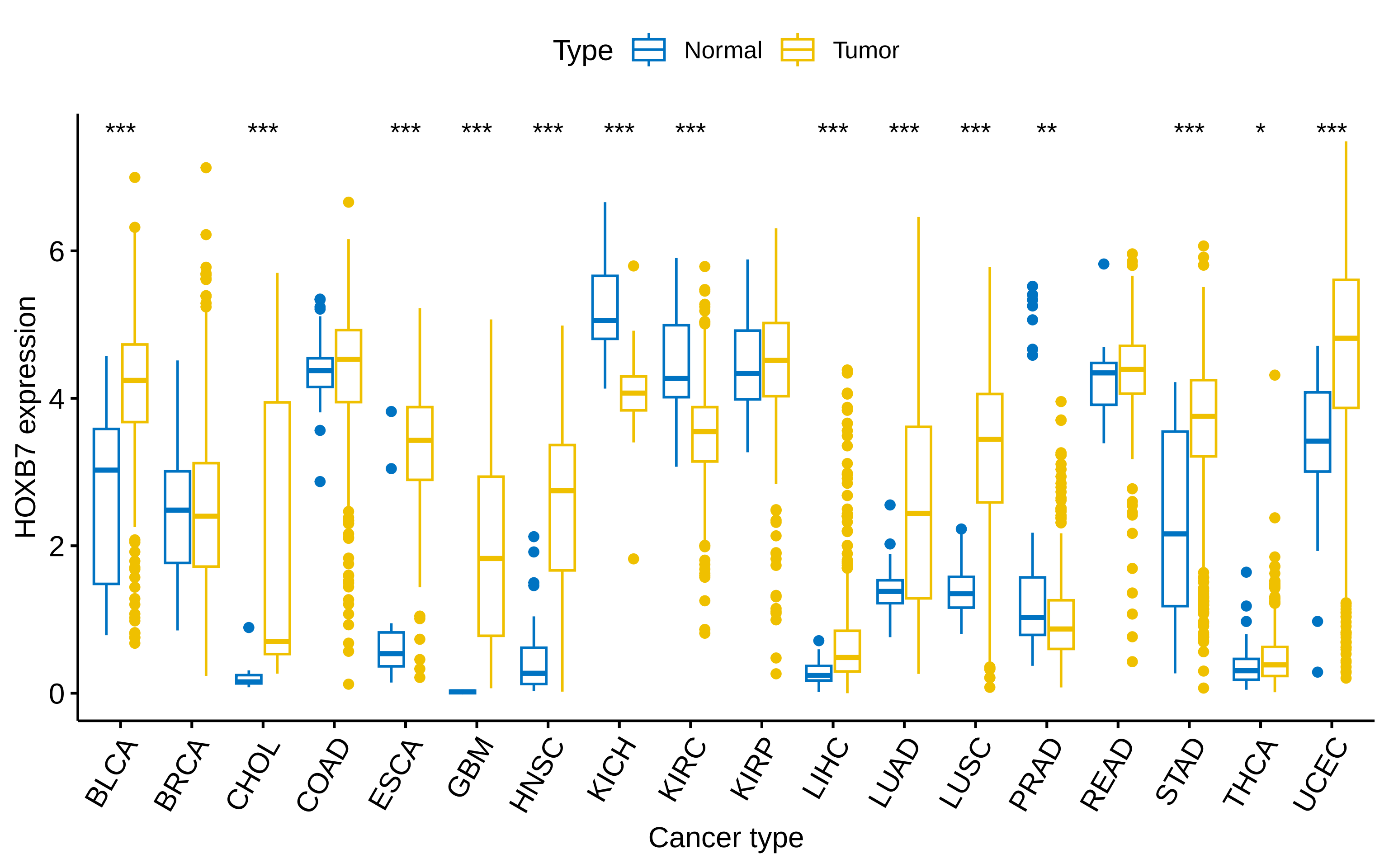
Type Normal Tumor
*** *** *** *** *** *** *** *** *** *** ** *** * ***
HOXB7 expression
0
2
4
6
BLCA BRCA CHOL COAD ESCA GBM HNSC KICH KIRC KIRP LIHC LUAD LUSC PRAD READ STAD THCA UCEC
Cancer type

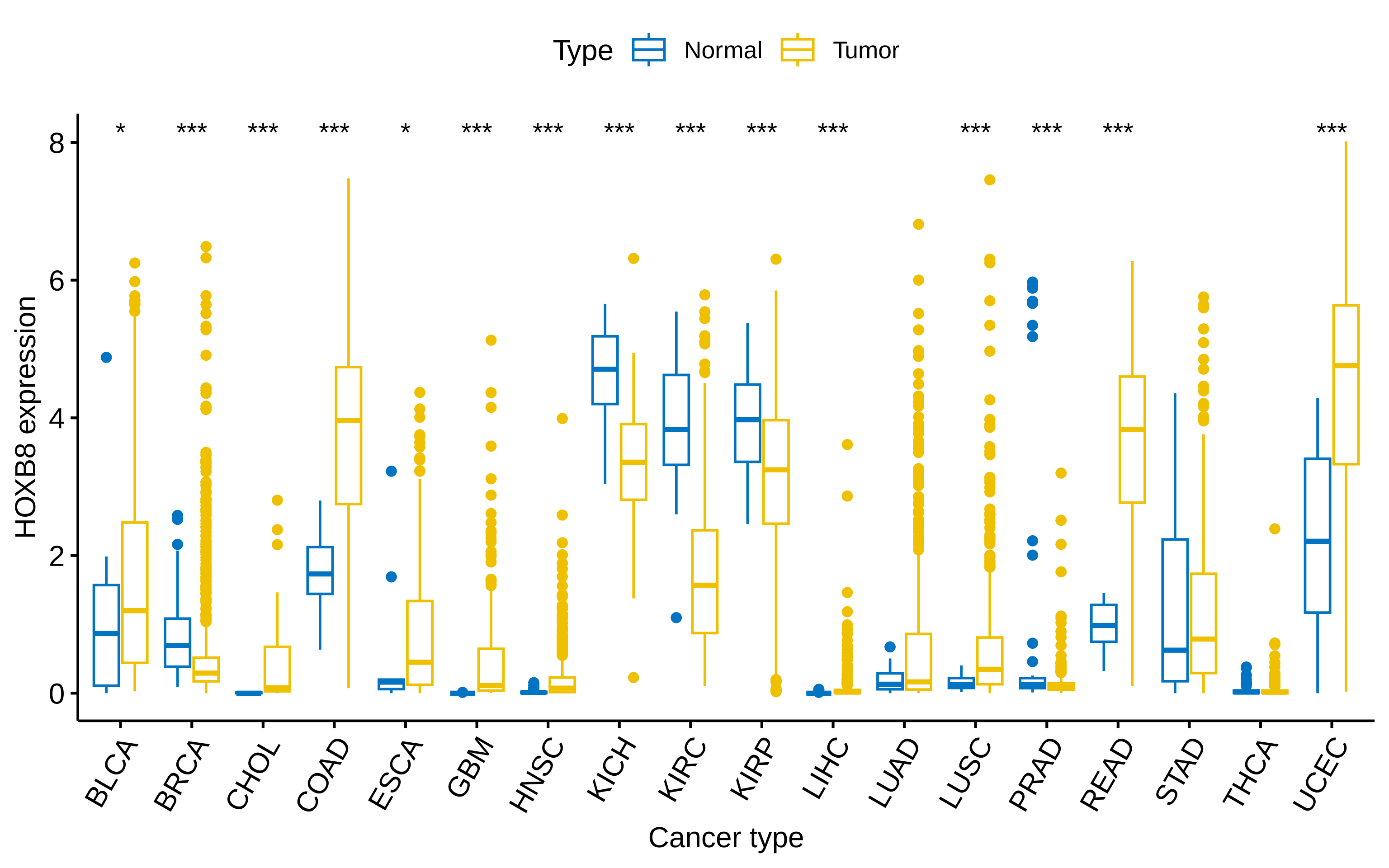

Type
Normal
Tumor
HOXB8 expression
Cancer type
*
***
***
***
*
***
***
***
***
***
***
***
***
***
***
8
6
4
2
0
BLCA
BRCA
CHOL
COAD
ESCA
GBM
HNSC
KICH
KIRC
KIRP
LIHC
LUAD
LUSC
PRAD
READ
STAD
THCA
UCEC

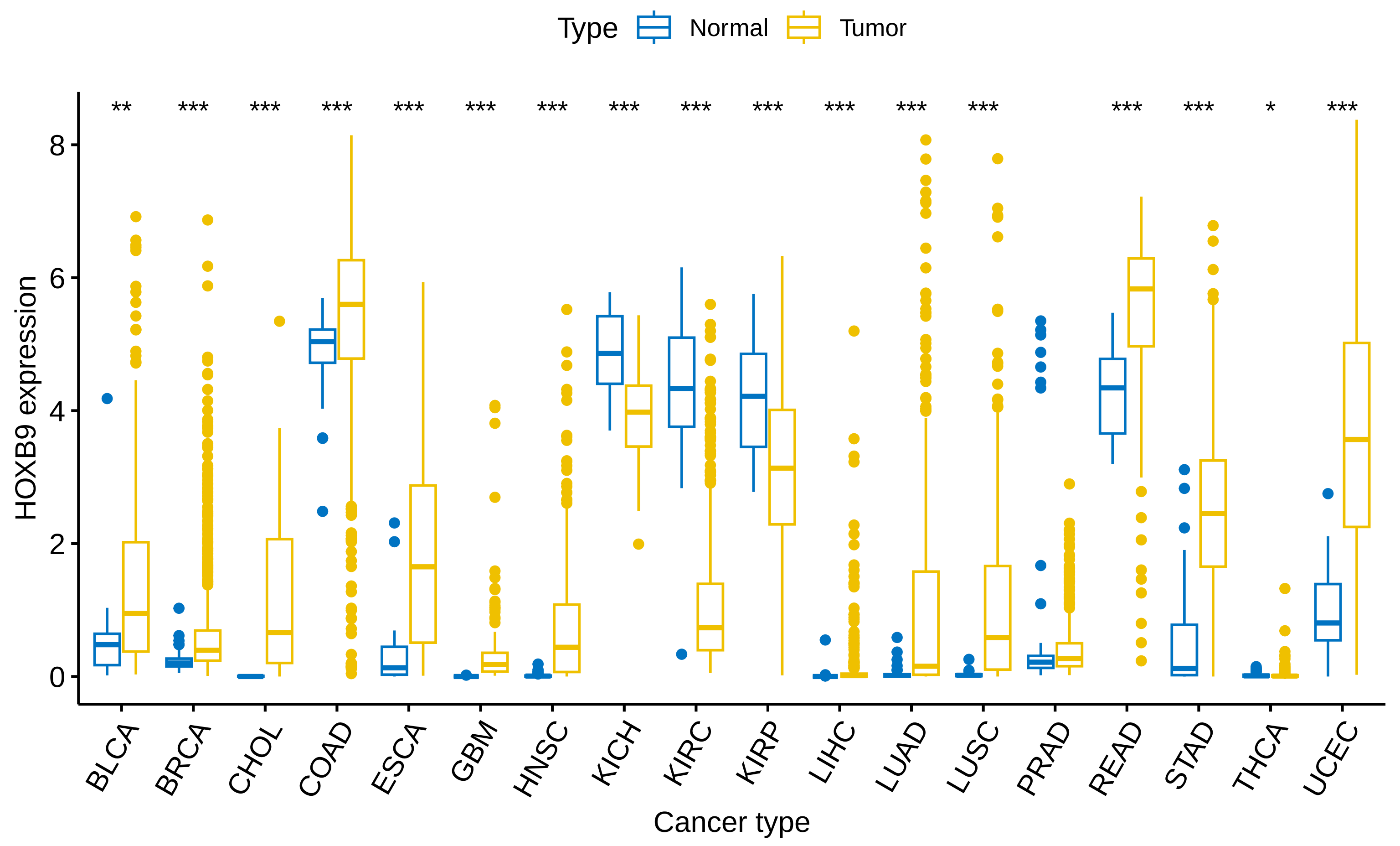
Type
Normal
Tumor
HOXB9 expression
0
2
4
6
8
**
***
***
***
***
***
***
***
***
***
***
***
***
***
***
*
***
BLCA
BRCA
CHOL
COAD
ESCA
GBM
HNSC
KICH
KIRC
KIRP
LIHC
LUAD
LUSC
PRAD
READ
STAD
THCA
UCEC
Cancer type

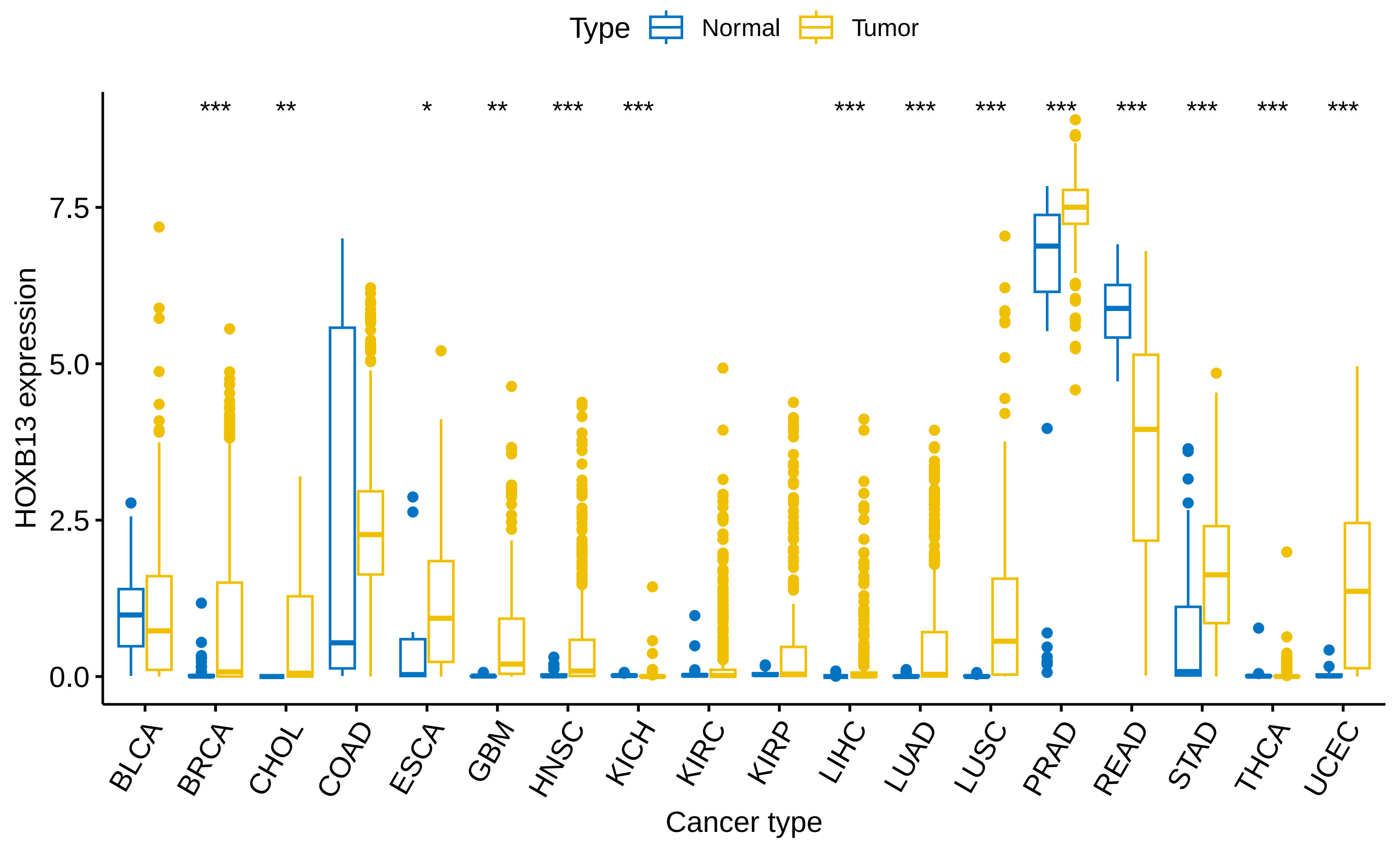
Type
Normal
Tumor
HOXB13 expression
7.5
5.0
2.5
0.0
*** ** * ** *** *** *** *** *** *** *** *** *** ***
BLCA
BRCA
CHOL
COAD
ESCA
GBM
HNSC
KICH
KIRC
KIRP
LIHC
LUAD
LUSC
PRAD
READ
STAD
THCA
UCEC
Cancer type

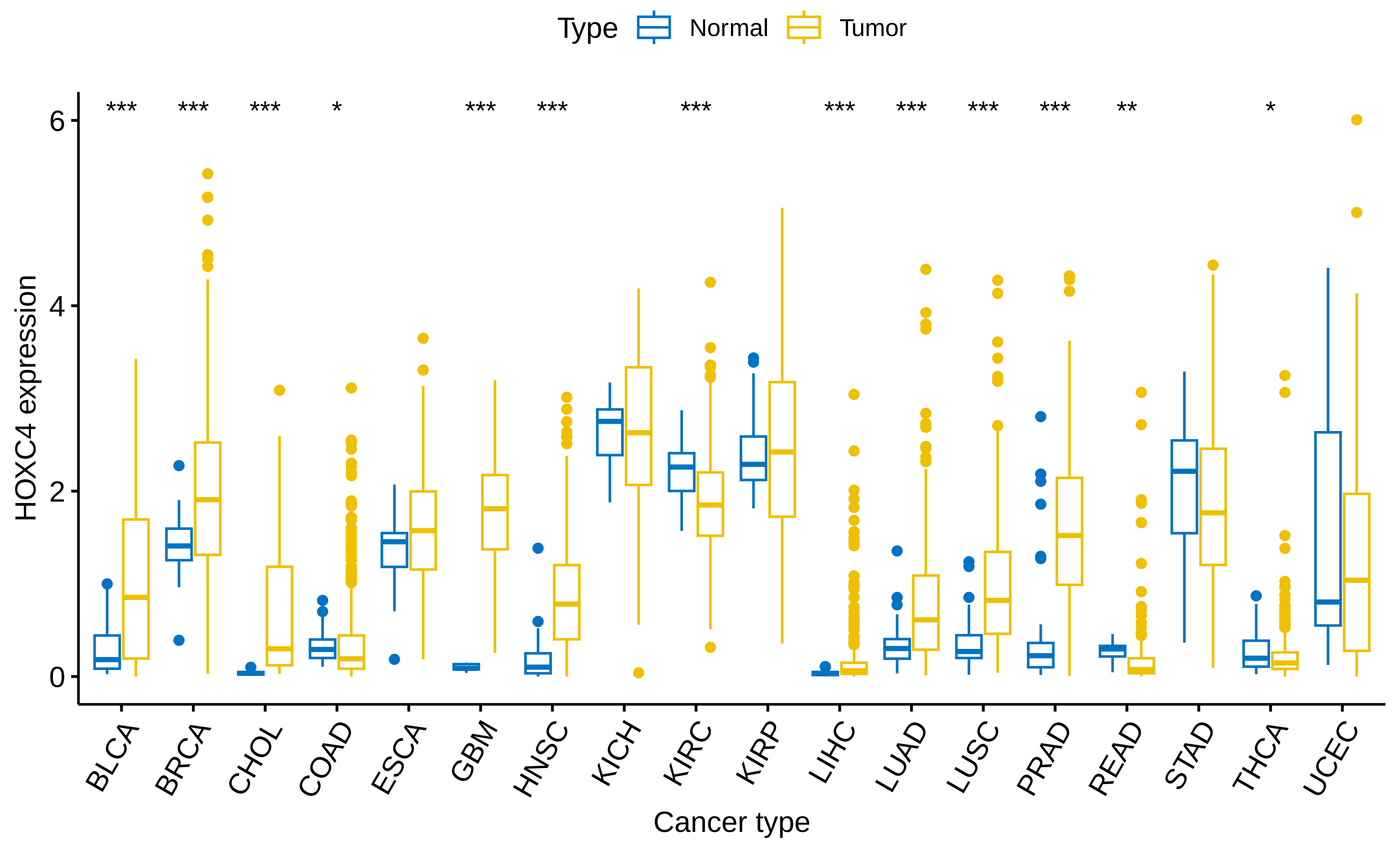

Type
Normal
Tumor
HOXC4 expression
0
2
4
6
*** *** *** * *** *** *** *** *** *** *** ** *
BLCA
BRCA
CHOL
COAD
ESCA
GBM
HNSC
KICH
KIRC
KIRP
LIHC
LUAD
LUSC
PRAD
READ
STAD
THCA
UCEC
Cancer type

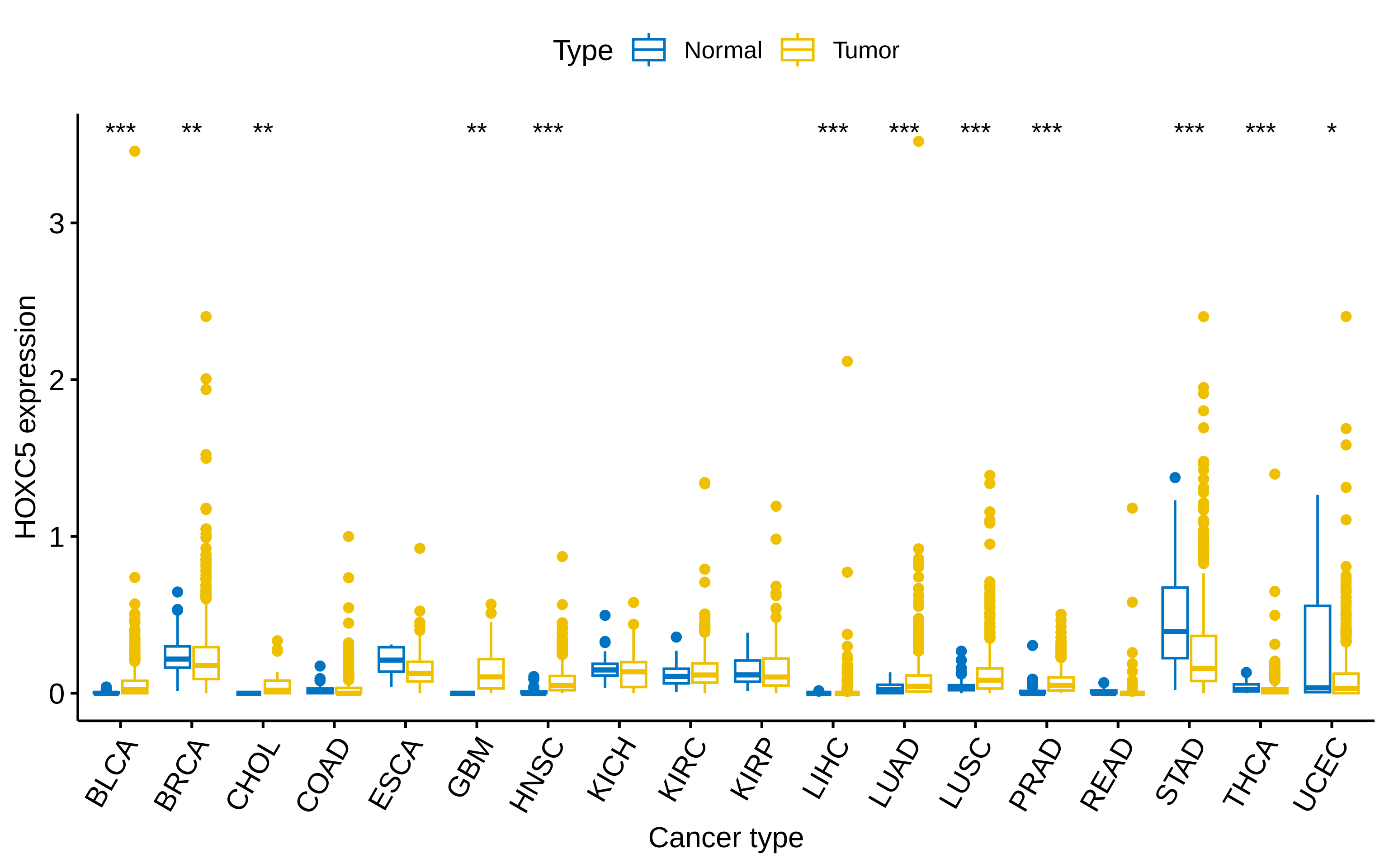
Type
Normal
Tumor
HOXC5 expression
0
1
2
3
***
**
**
**
***
***
***
***
***
***
***
*
BLCA
BRCA
CHOL
COAD
ESCA
GBM
HNSC
KICH
KIRC
KIRP
LIHC
LUAD
LUSC
PRAD
READ
STAD
THCA
UCEC
Cancer type

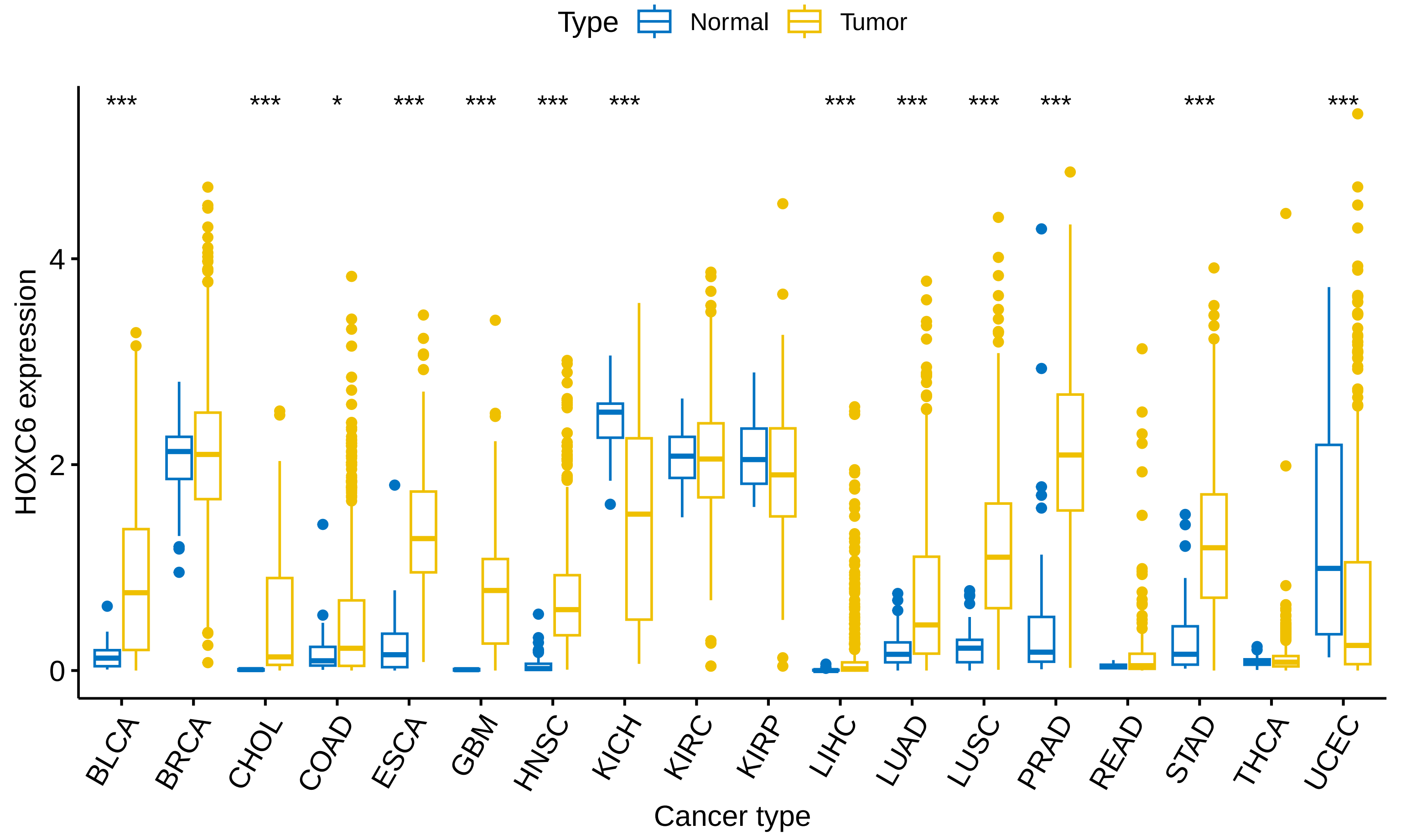

Type
Normal
Tumor
*** *** * *** *** *** *** *** *** *** *** *** ***
HOXC6 expression
0
2
4
BLCA
BRCA
CHOL
COAD
ESCA
GBM
HNSC
KICH
KIRC
KIRP
LIHC
LUAD
LUSC
PRAD
READ
STAD
THCA
UCEC
Cancer type

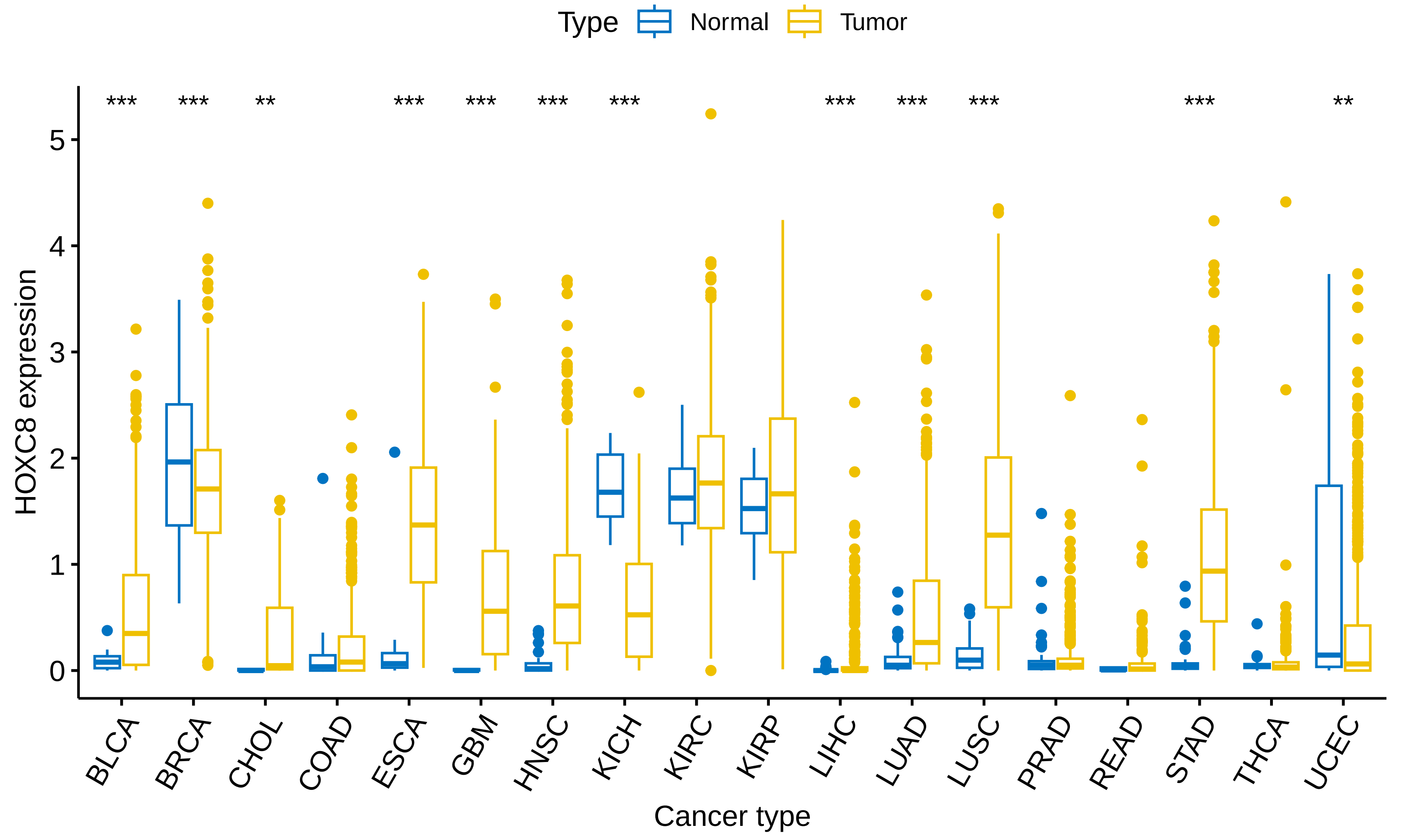

Type
Normal
Tumor
HOXC8 expression
0
1
2
3
4
5
***
***
**
***
***
***
***
***
***
***
***
**
BLCA
BRCA
CHOL
COAD
ESCA
GBM
HNSC
KICH
KIRC
KIRP
LIHC
LUAD
LUSC
PRAD
READ
STAD
THCA
UCEC
Cancer type

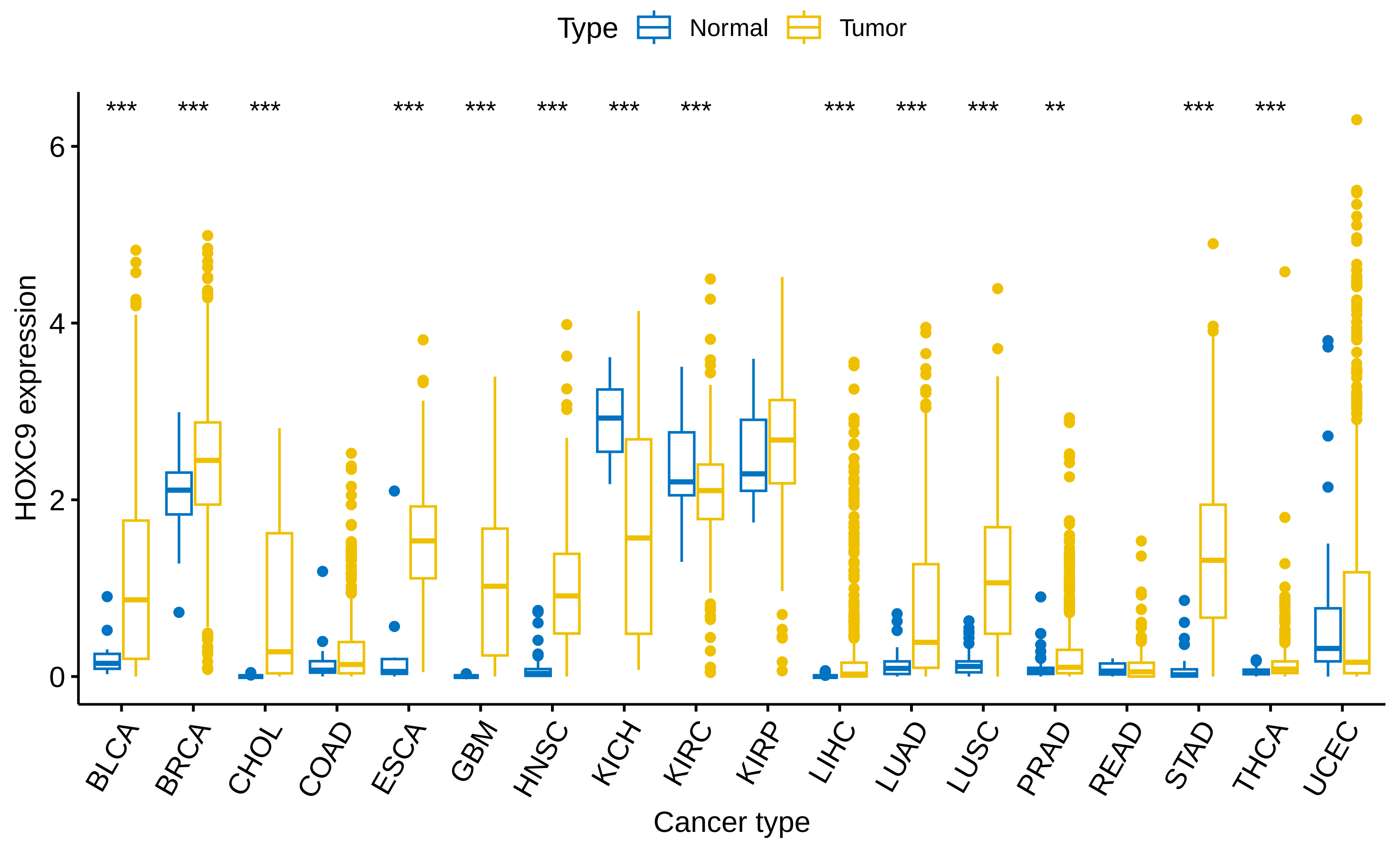

Type
Normal
Tumor
*** *** *** *** *** *** *** *** *** *** *** ** *** ***
HOXC9 expression
0
2
4
6
BLCA
BRCA
CHOL
COAD
ESCA
GBM
HNSC
KICH
KIRC
KIRP
LIHC
LUAD
LUSC
PRAD
READ
STAD
THCA
UCEC
Cancer type

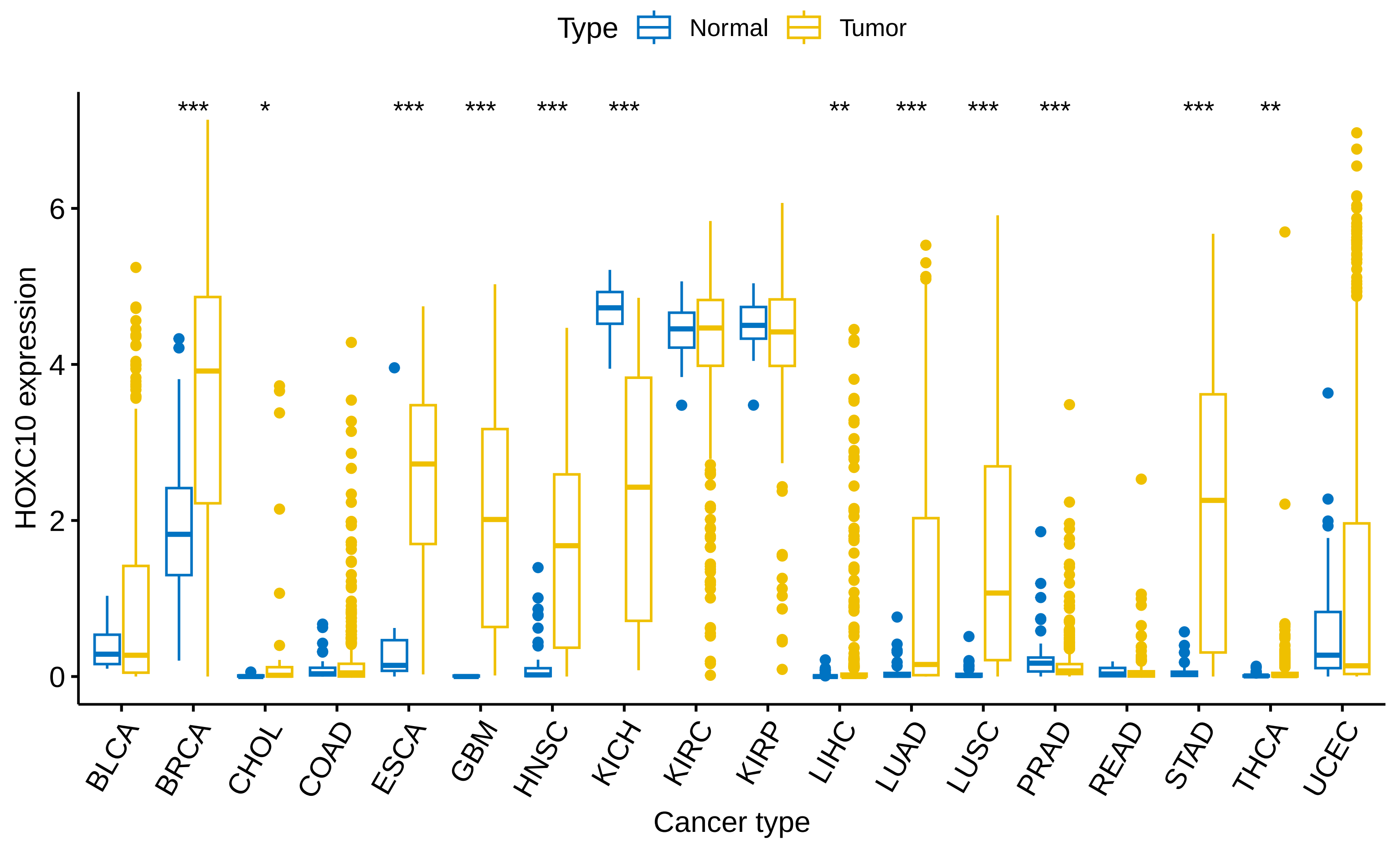

Type
Normal
Tumor
HOXC10 expression
0
2
4
6
***
*
***
***
***
***
**
***
***
***
***
**
BLCA
BRCA
CHOL
COAD
ESCA
GBM
HNSC
KICH
KIRC
KIRP
LIHC
LUAD
LUSC
PRAD
READ
STAD
THCA
UCEC
Cancer type

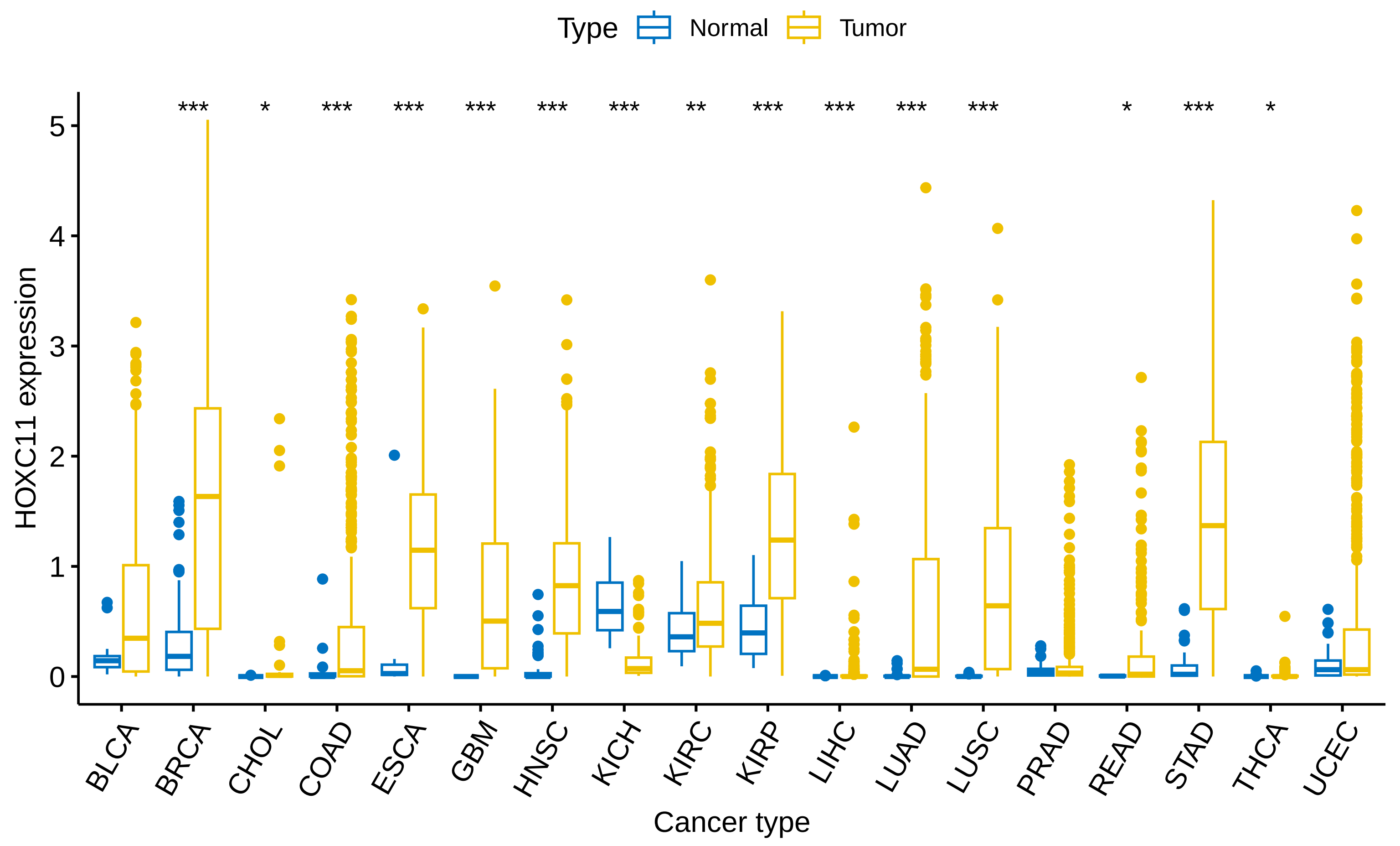
Type
Normal
Tumor
HOXC11 expression
5
4
3
2
1
0
***
*
***
***
***
***
***
**
***
***
***
***
*
***
*
BLCA
BRCA
CHOL
COAD
ESCA
GBM
HNSC
KICH
KIRC
KIRP
LIHC
LUAD
LUSC
PRAD
READ
STAD
THCA
UCEC
Cancer type

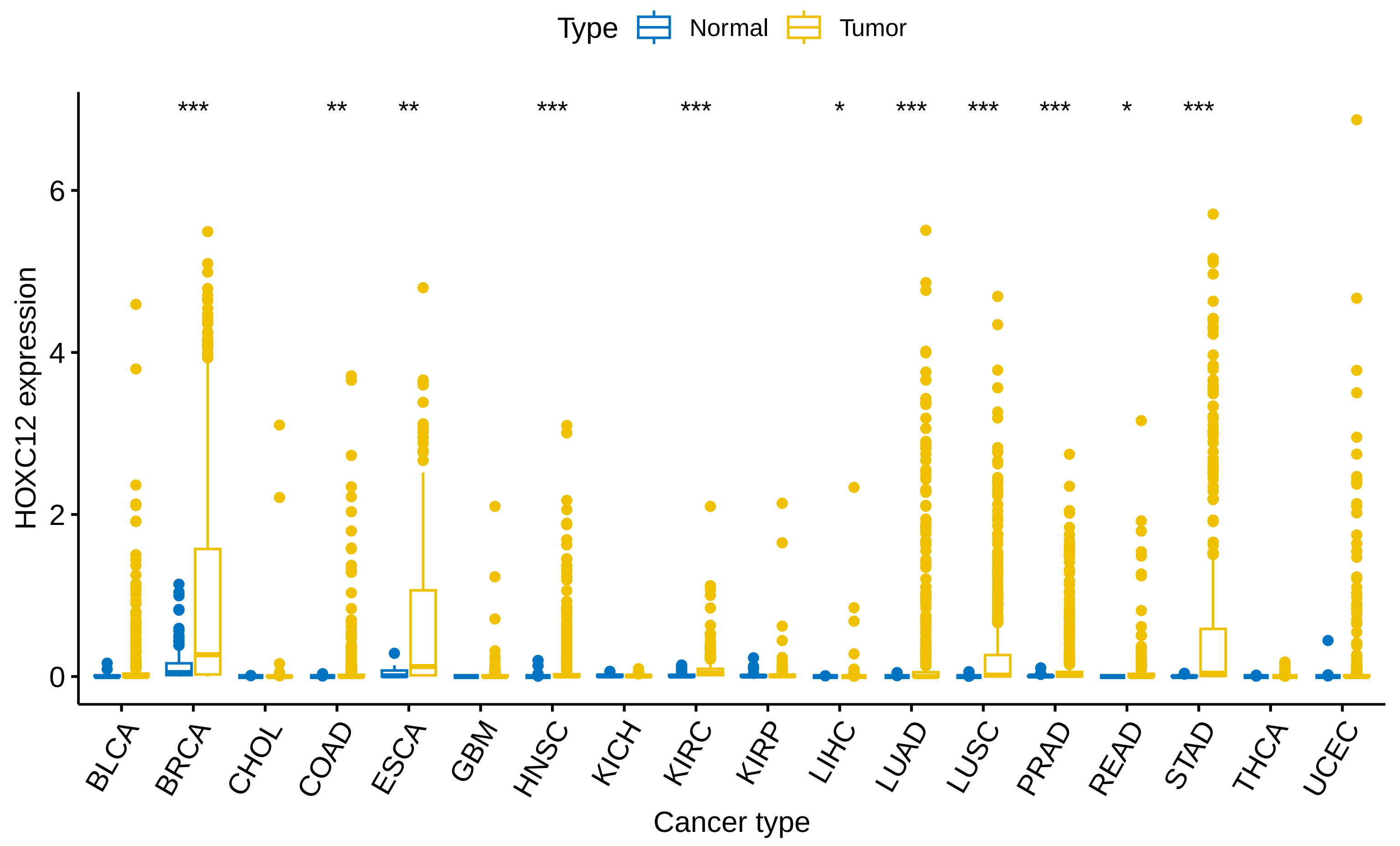

Type
Normal
Tumor
HOXC12 expression
0
2
4
6
***
**
**
***
***
*
***
***
***
*
***
BLCA
BRCA
CHOL
COAD
ESCA
GBM
HNSC
KICH
KIRC
KIRP
LIHC
LUAD
LUSC
PRAD
READ
STAD
THCA
UCEC
Cancer type

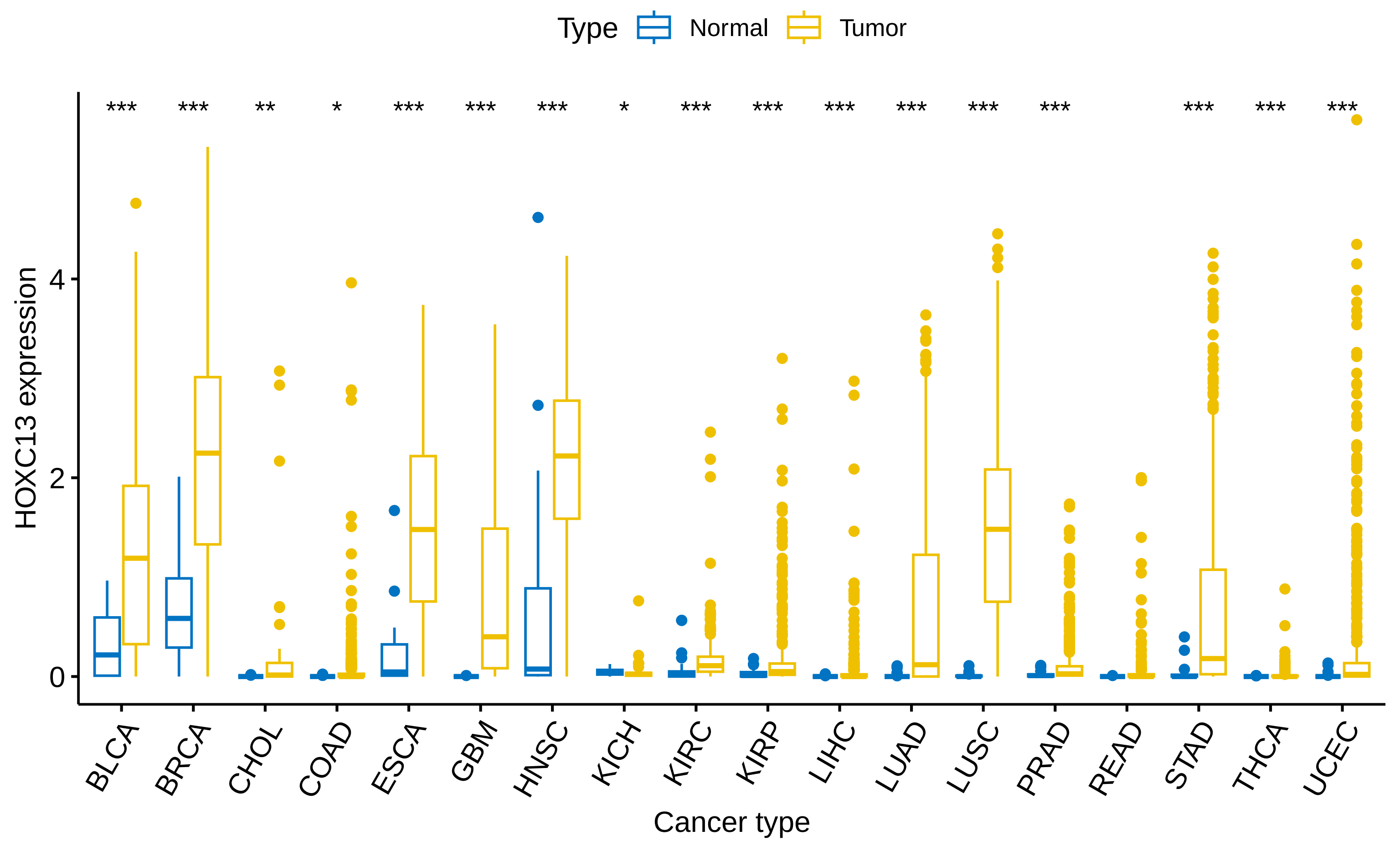

Type
Normal
Tumor
HOXC13 expression
0
2
4
*** *** ** * *** *** *** * *** *** *** *** *** *** *** *** ***
BLCA
BRCA
CHOL
COAD
ESCA
GBM
HNSC
KICH
KIRC
KIRP
LIHC
LUAD
LUSC
PRAD
READ
STAD
THCA
UCEC
Cancer type

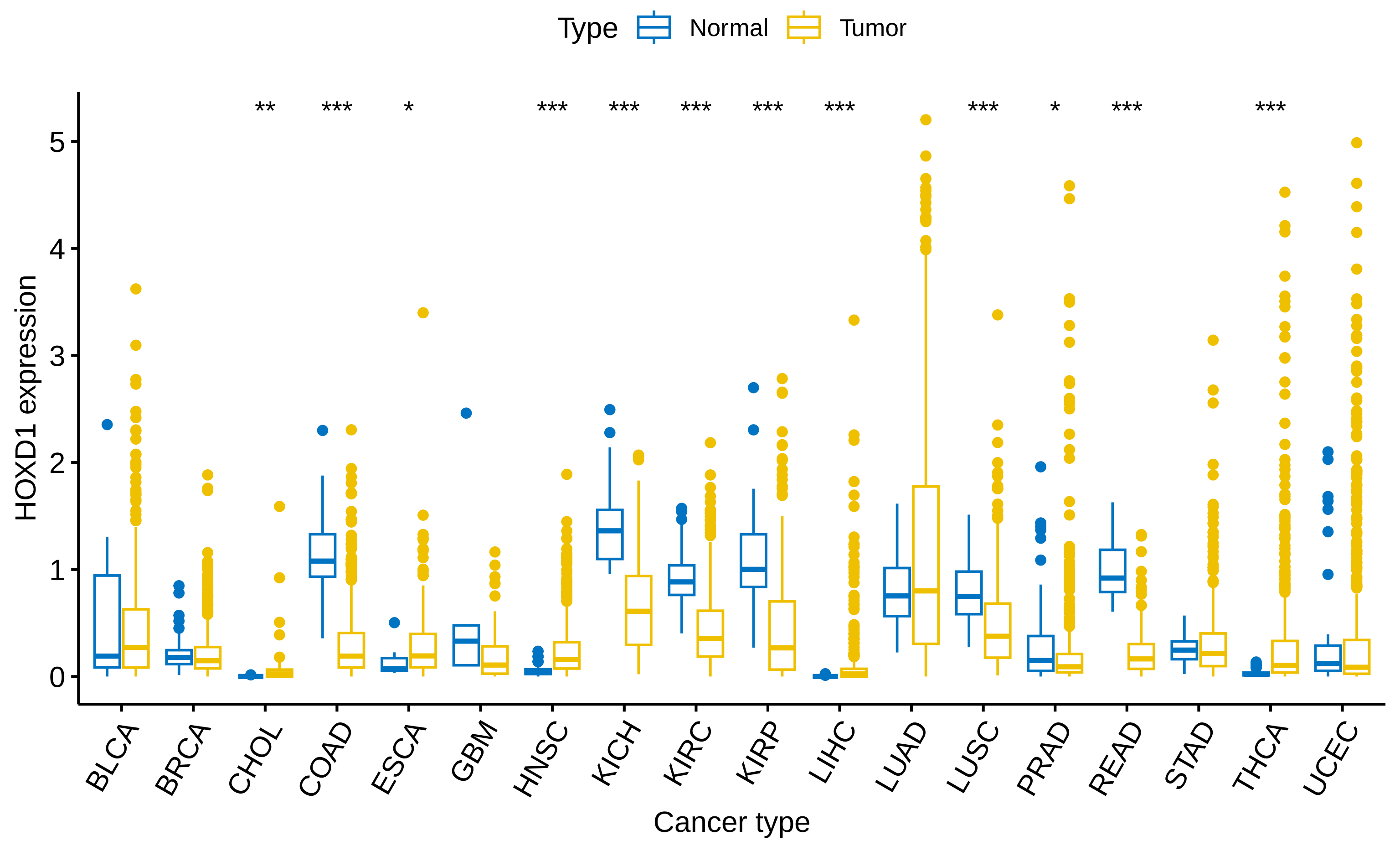

Type
Normal
Tumor
HOXD1 expression
0
1
2
3
4
5
**
***
*
***
***
***
***
***
***
*
***
***
BLCA
BRCA
CHOL
COAD
ESCA
GBM
HNSC
KICH
KIRC
KIRP
LIHC
LUAD
LUSC
PRAD
READ
STAD
THCA
UCEC
Cancer type

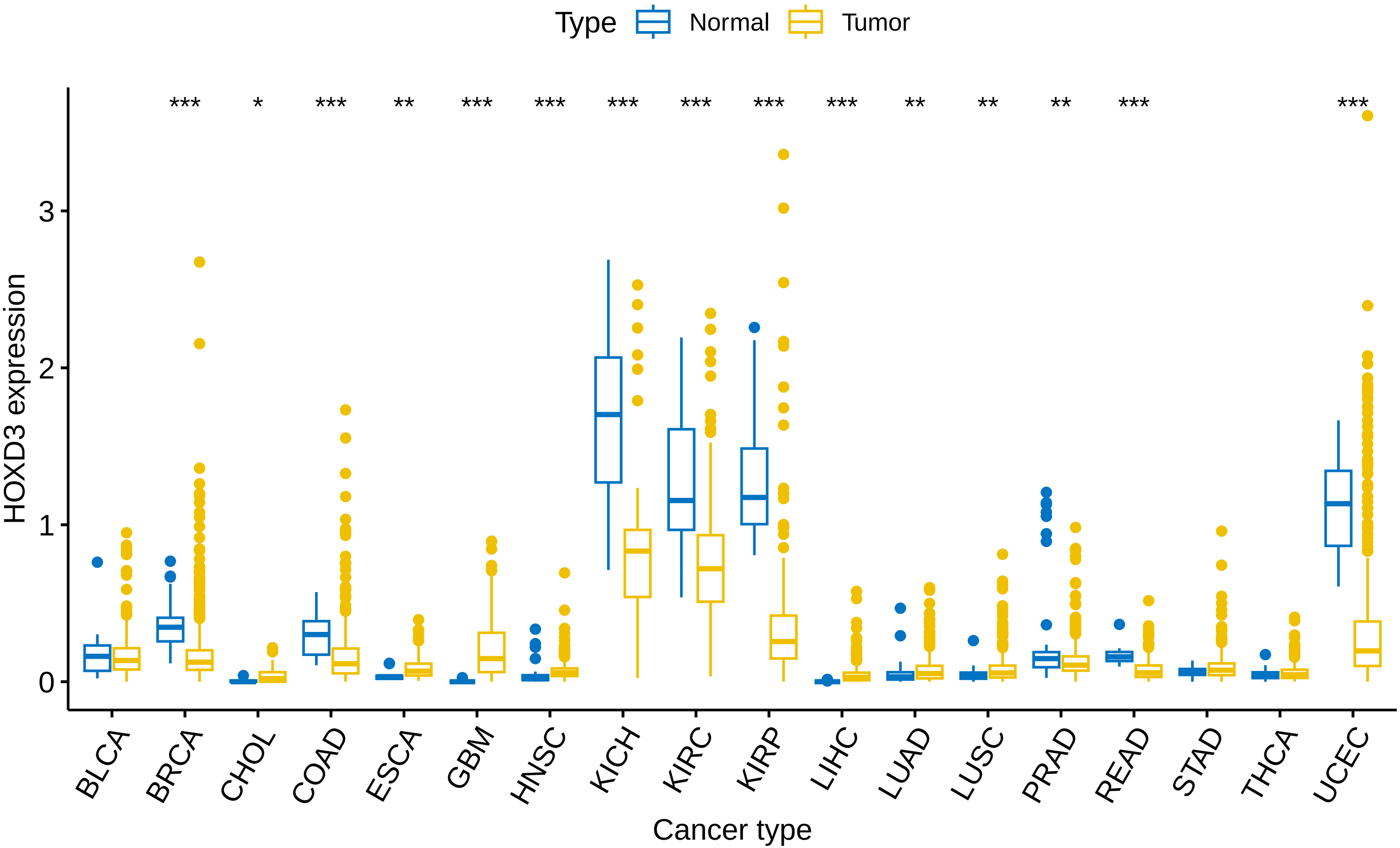

Type
Normal
Tumor
HOXD3 expression
0
1
2
3
*** * *** ** *** *** *** *** *** *** ** ** ** *** ***
BLCA
BRCA
CHOL
COAD
ESCA
GBM
HNSC
KICH
KIRC
KIRP
LIHC
LUAD
LUSC
PRAD
READ
STAD
THCA
UCEC
Cancer type

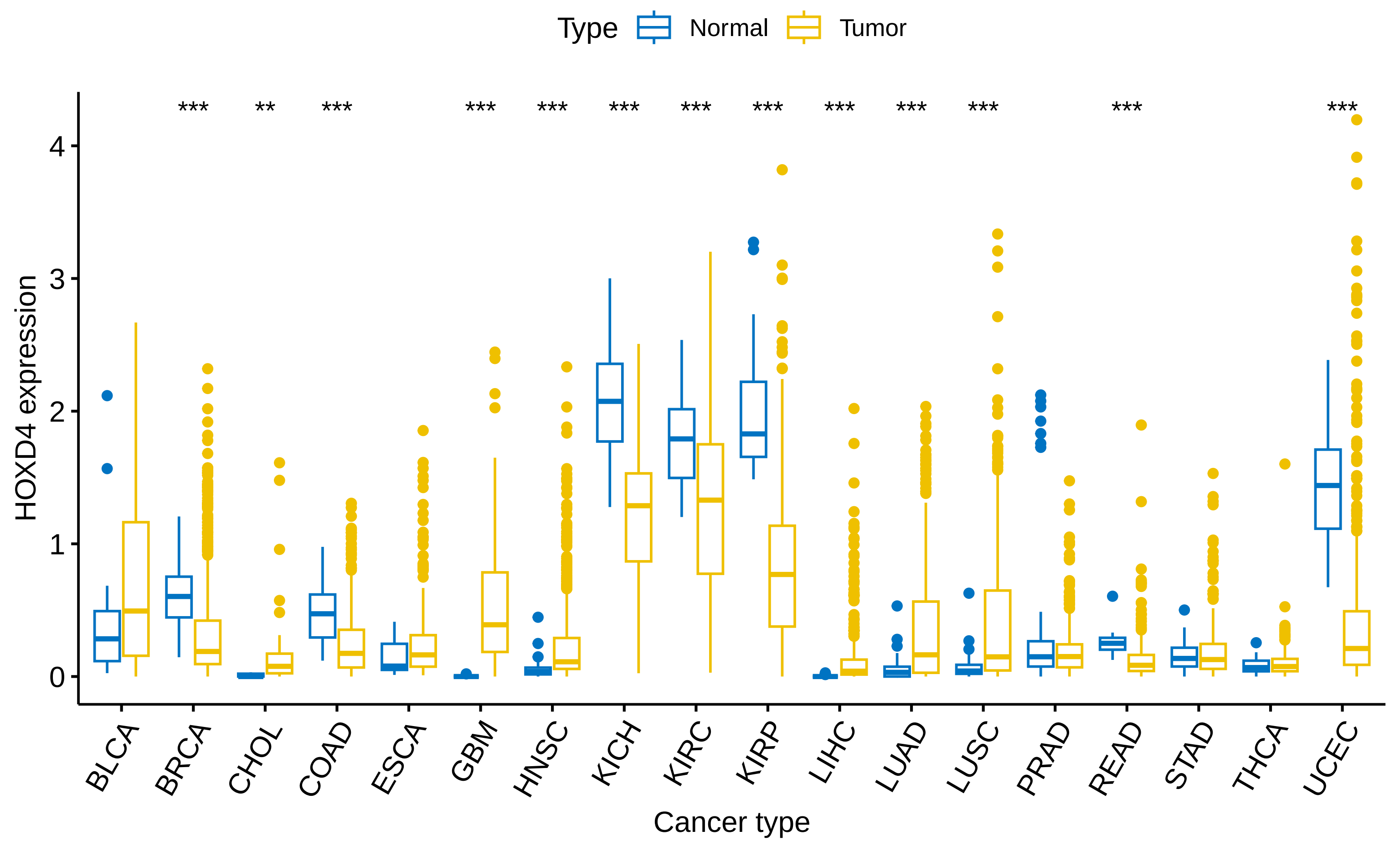

Type
Normal
Tumor
HOXD4 expression
0
1
2
3
4
***
**
***
***
***
***
***
***
***
***
***
***
***
BLCA
BRCA
CHOL
COAD
ESCA
GBM
HNSC
KICH
KIRC
KIRP
LIHC
LUAD
LUSC
PRAD
READ
STAD
THCA
UCEC
Cancer type

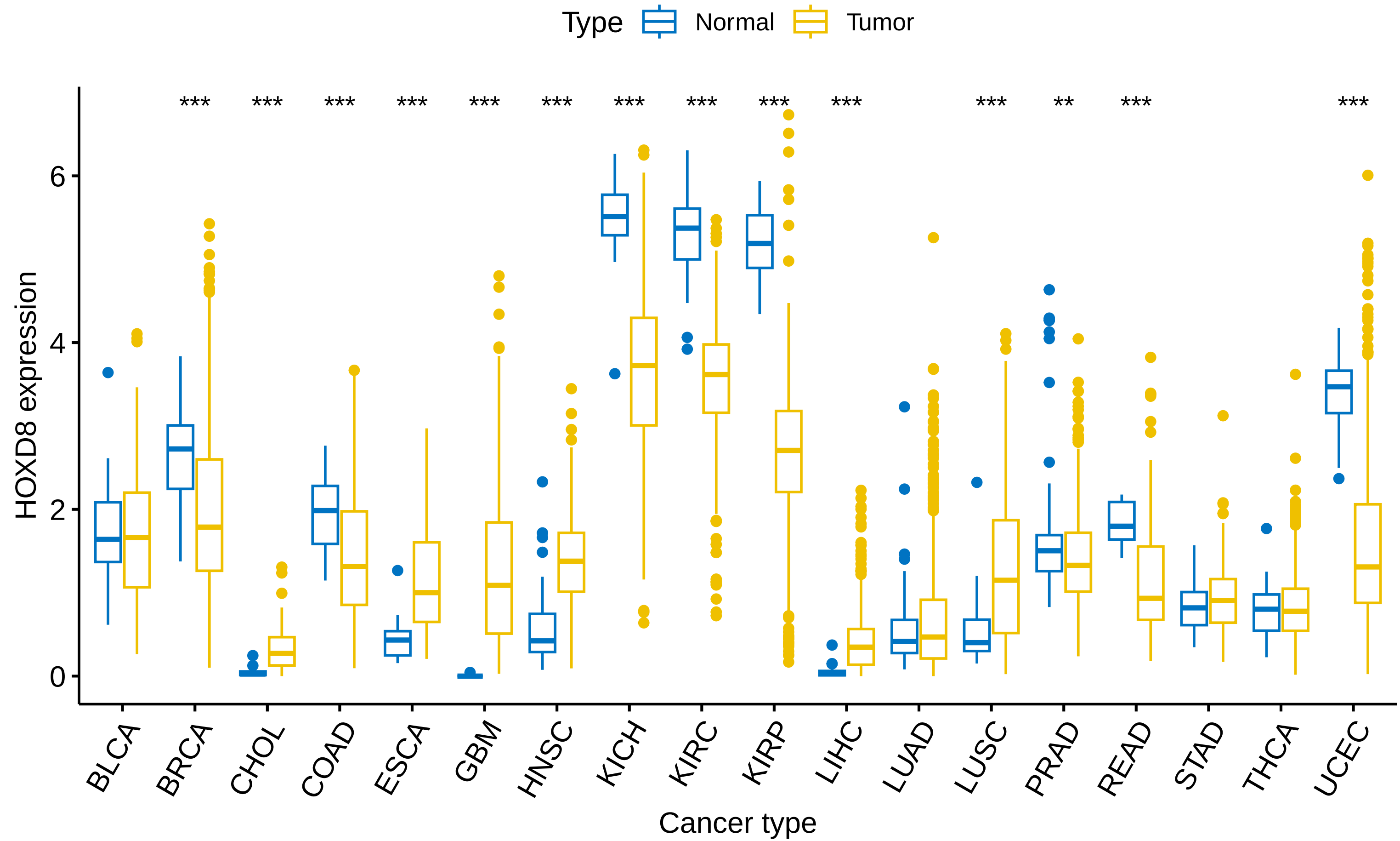

Type
Normal
Tumor
***
***
***
***
***
***
***
***
***
***
***
**
***
***
HOXD8 expression
6
4
2
0
BLCA
BRCA
CHOL
COAD
ESCA
GBM
HNSC
KICH
KIRC
KIRP
LIHC
LUAD
LUSC
PRAD
READ
STAD
THCA
UCEC
Cancer type

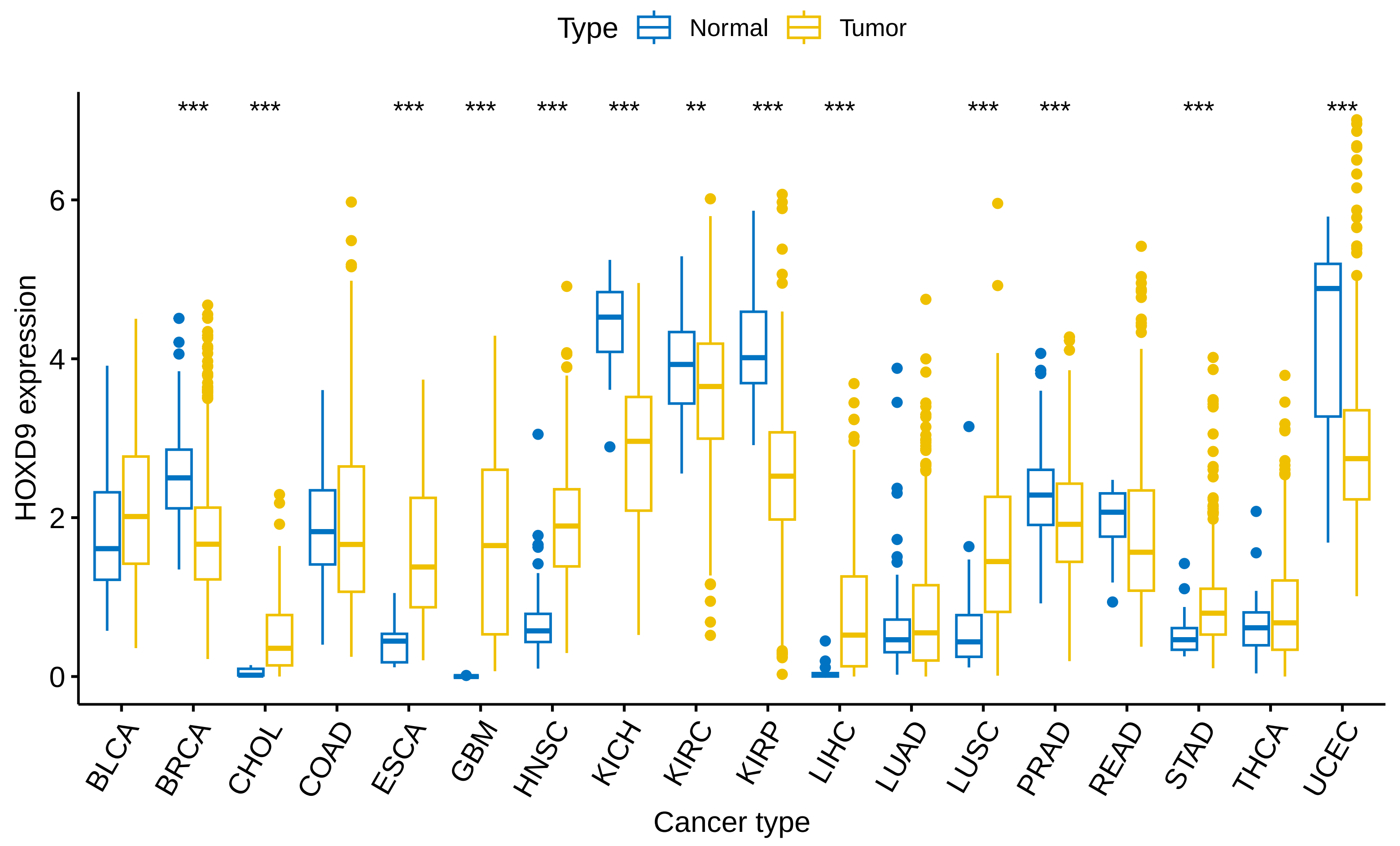
Type
Normal
Tumor
HOXD9 expression
0
2
4
6
***
***
***
***
***
***
**
***
***
***
***
***
***
BLCA
BRCA
CHOL
COAD
ESCA
GBM
HNSC
KICH
KIRC
KIRP
LIHC
LUAD
LUSC
PRAD
READ
STAD
THCA
UCEC
Cancer type

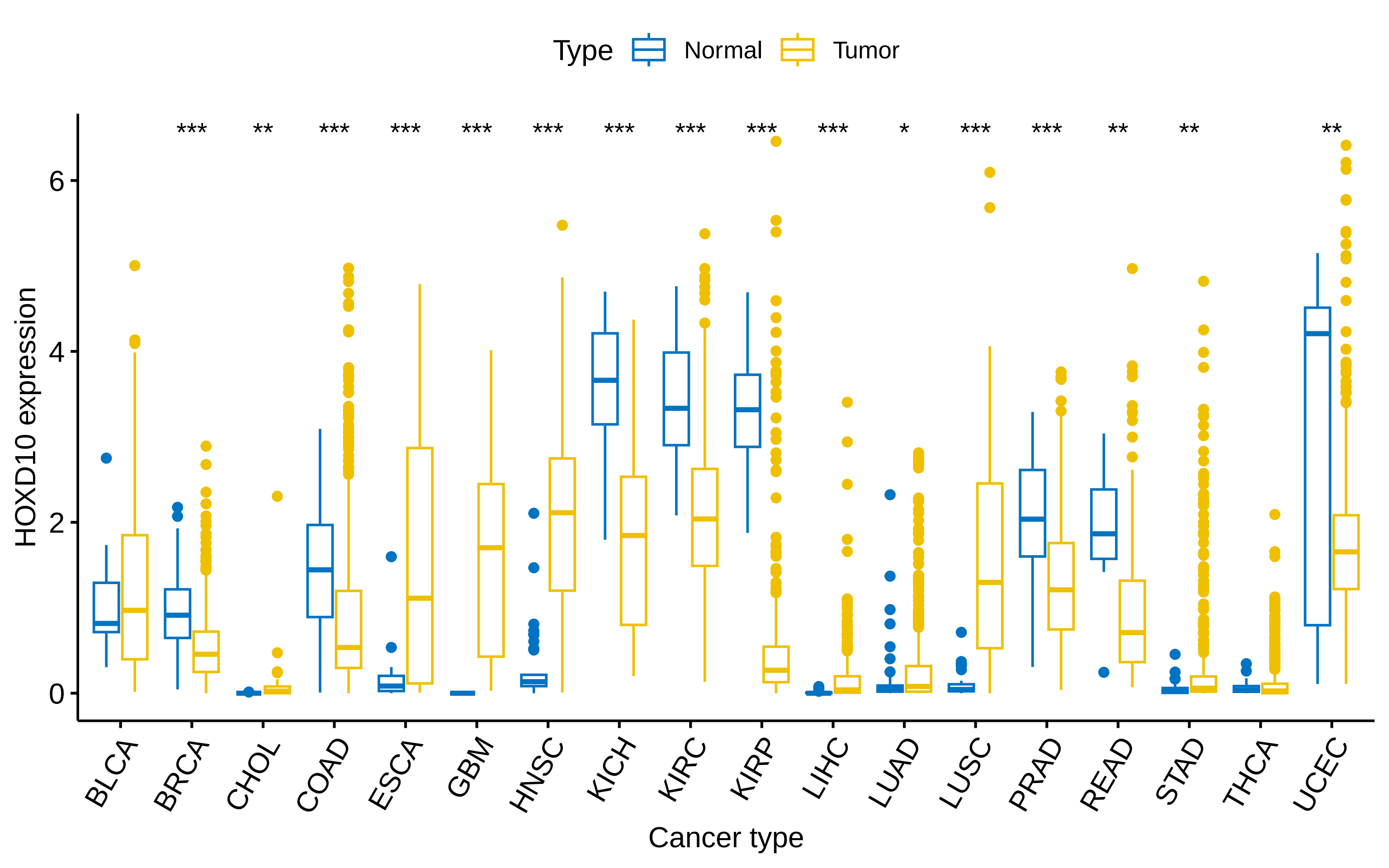

Type
Normal
Tumor
HOXD10 expression
0
2
4
6
*** ** *** *** *** *** *** *** *** *** * *** *** ** ** **
BLCA
BRCA
CHOL
COAD
ESCA
GBM
HNSC
KICH
KIRC
KIRP
LIHC
LUAD
LUSC
PRAD
READ
STAD
THCA
UCEC
Cancer type

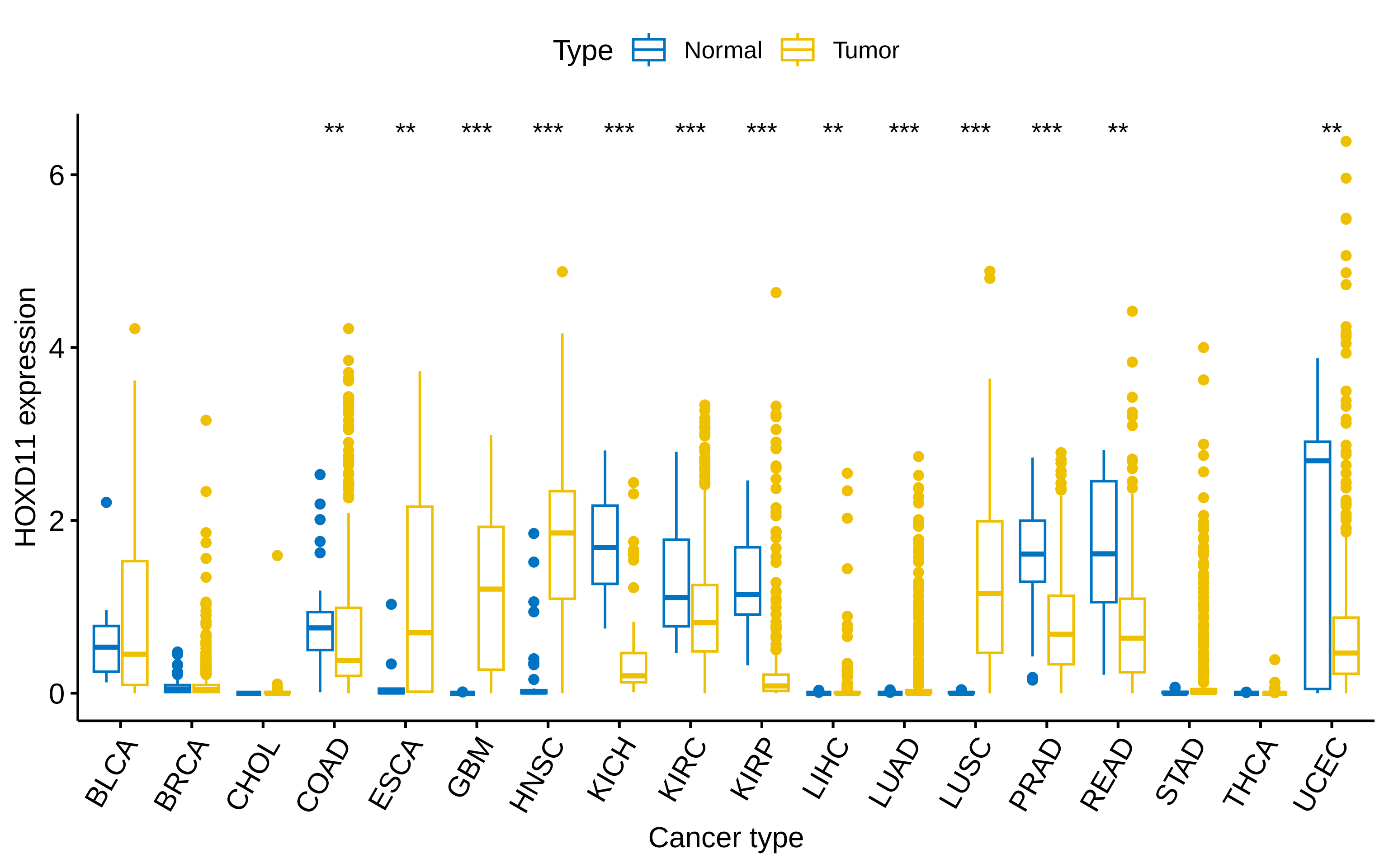

Type
Normal
Tumor
HOXD11 expression
0
2
4
6
** ** *** *** *** *** *** ** *** *** *** ** **
BLCA
BRCA
CHOL
COAD
ESCA
GBM
HNSC
KICH
KIRC
KIRP
LIHC
LUAD
LUSC
PRAD
READ
STAD
THCA
UCEC
Cancer type

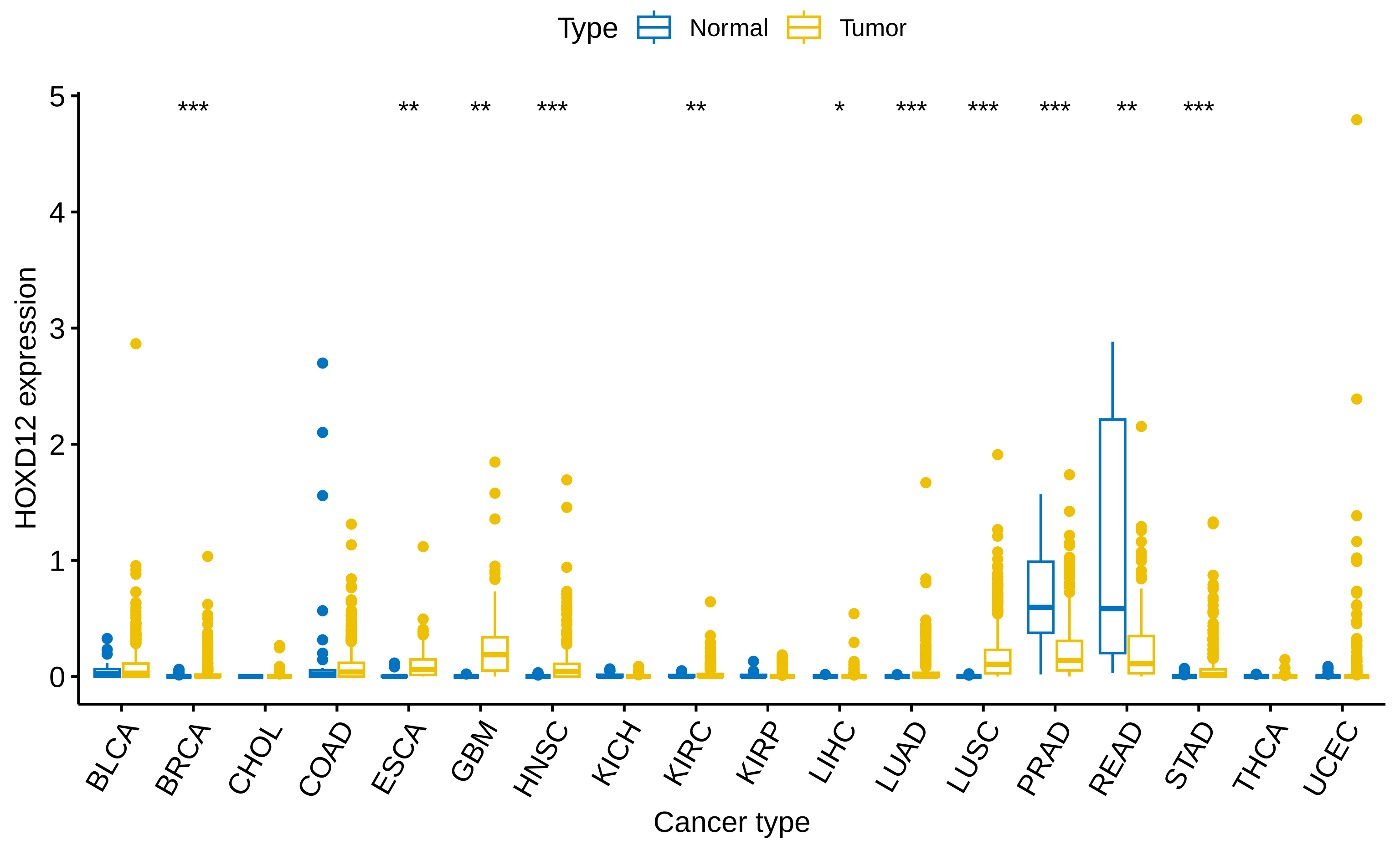

Type
Normal
Tumor
HOXD12 expression
0
1
2
3
4
5
***
**
**
***
**
*
***
***
***
**
***
BLCA
BRCA
CHOL
COAD
ESCA
GBM
HNSC
KICH
KIRC
KIRP
LIHC
LUAD
LUSC
PRAD
READ
STAD
THCA
UCEC
Cancer type

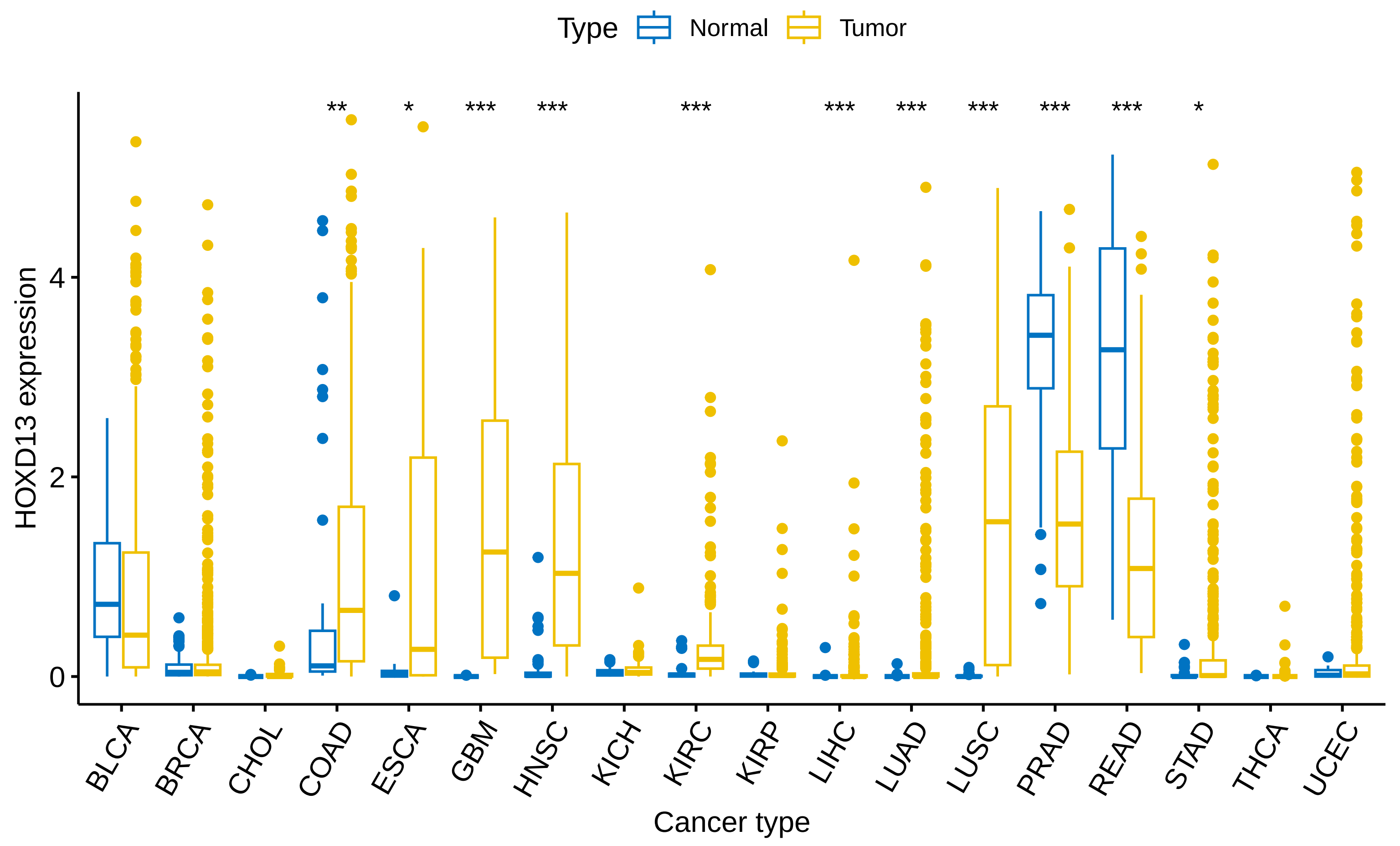
Type
Normal
Tumor
HOXD13 expression
0
2
4
**
*
***
***
***
***
***
***
***
***
*
BLCA
BRCA
CHOL
COAD
ESCA
GBM
HNSC
KICH
KIRC
KIRP
LIHC
LUAD
LUSC
PRAD
READ
STAD
THCA
UCEC
Cancer type
