# Supplementary material for: A pan-cancer analysis of homeobox family: expression characteristics and latent significance in prognosis and immune microenvironment
Source: Front Oncol. 2025 Feb 6;15:1521652. doi: 10.3389/fonc.2025.1521652 (PMC11840236; doi:10.3389/fonc.2025.1521652)
Supplement: Supplementary Figure 1 — Mutations of each HOX gene in 32 cancers (TCGA, PanCancer Atlas) by cBioportal. [file DataSheet1.zip › Suppl.files/S3_File.pdf]

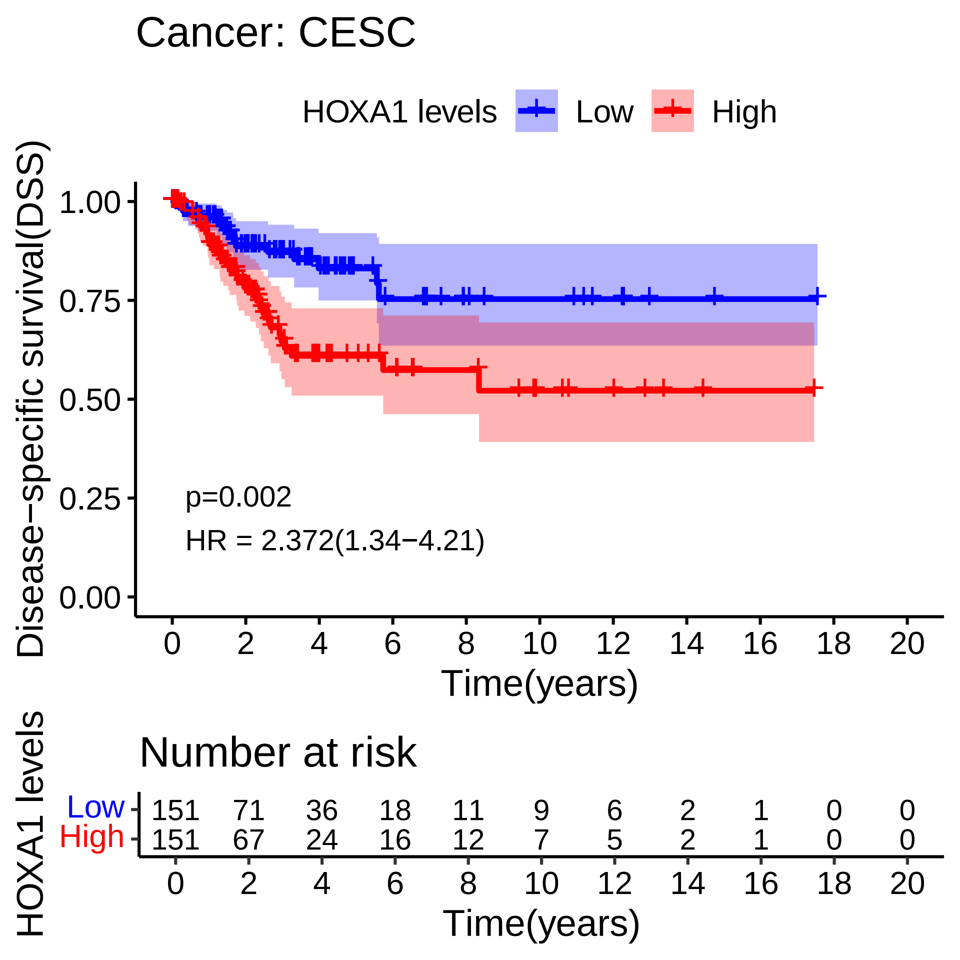

Cancer: CESC
HOXA1 levels
Low
High
Disease−specific survival(DSS)
1.00
0.75
0.50
0.25
0.00
p=0.002
HR = 2.372(1.34−4.21)
0 2 4 6 8 10 12 14 16 18 20
Time(years)
Number at risk
HOXA1 levels
Low 151 71 36 18 11 9 6 2 1 0 0
High 151 67 24 16 12 7 5 2 1 0 0
0 2 4 6 8 10 12 14 16 18 20
Time(years)

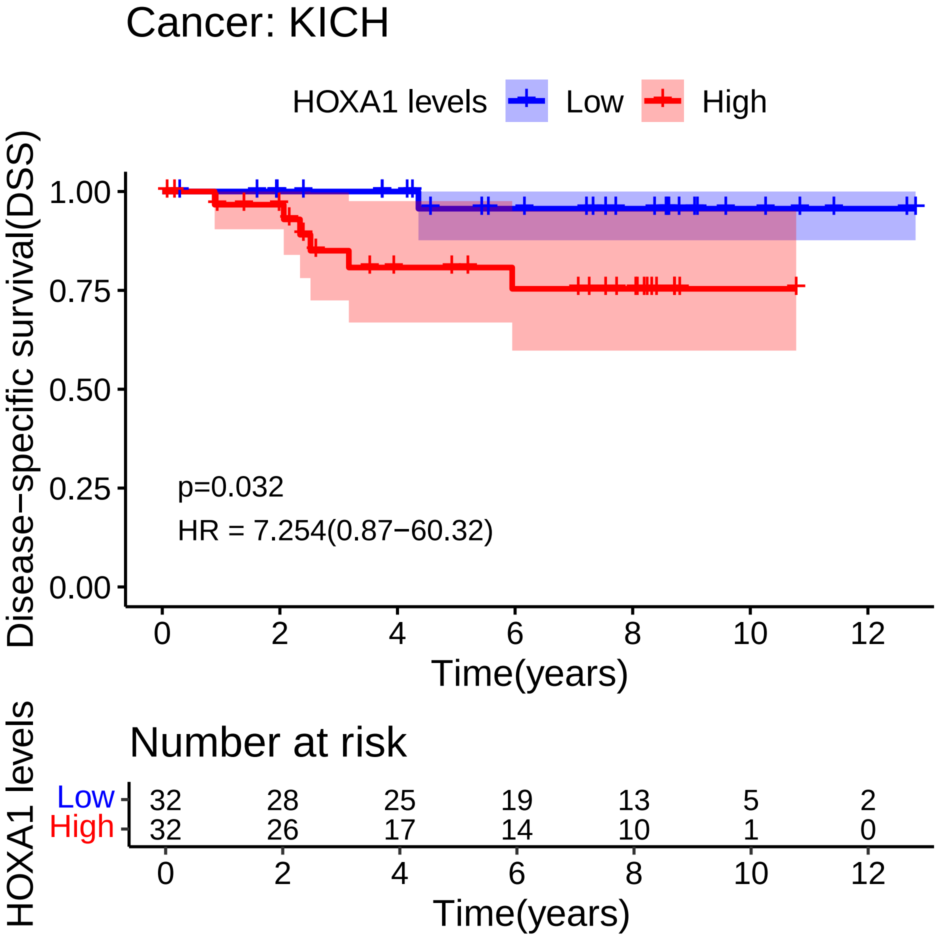

Cancer: KICH
HOXA1 levels
Low
High
Disease−specific survival(DSS)
1.00
0.75
0.50
0.25
0.00
p=0.032
HR = 7.254(0.87−60.32)
0
2
4
6
8
10
12
Time(years)
Number at risk
HOXA1 levels
Low 32 28 25 19 13 5 2
High 32 26 17 14 10 1 0
Time(years)

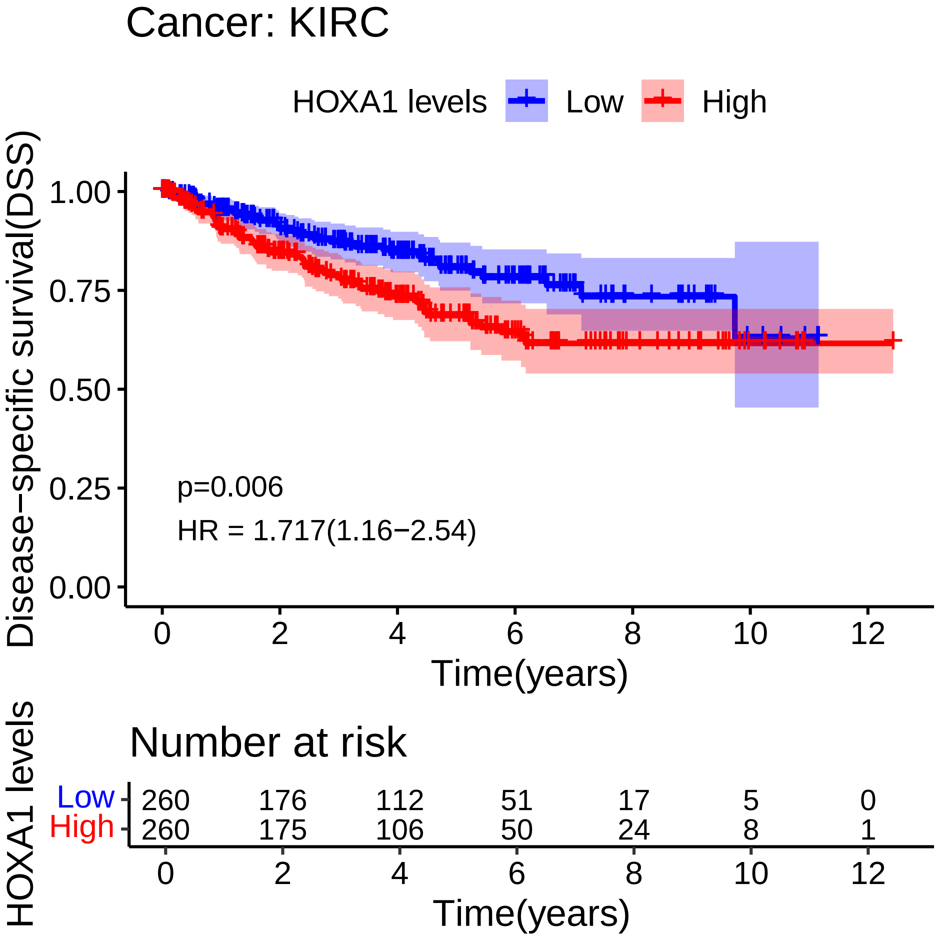

Cancer: KIRC
HOXA1 levels
Low
High
Disease−specific survival(DSS)
1.00
0.75
0.50
0.25
0.00
p=0.006
HR = 1.717(1.16−2.54)
0
2
4
6
8
10
12
Time(years)
Number at risk
HOXA1 levels
Low 260 176 112 51 17 5 0
High 260 175 106 50 24 8 1
0
2
4
6
8
10
12
Time(years)

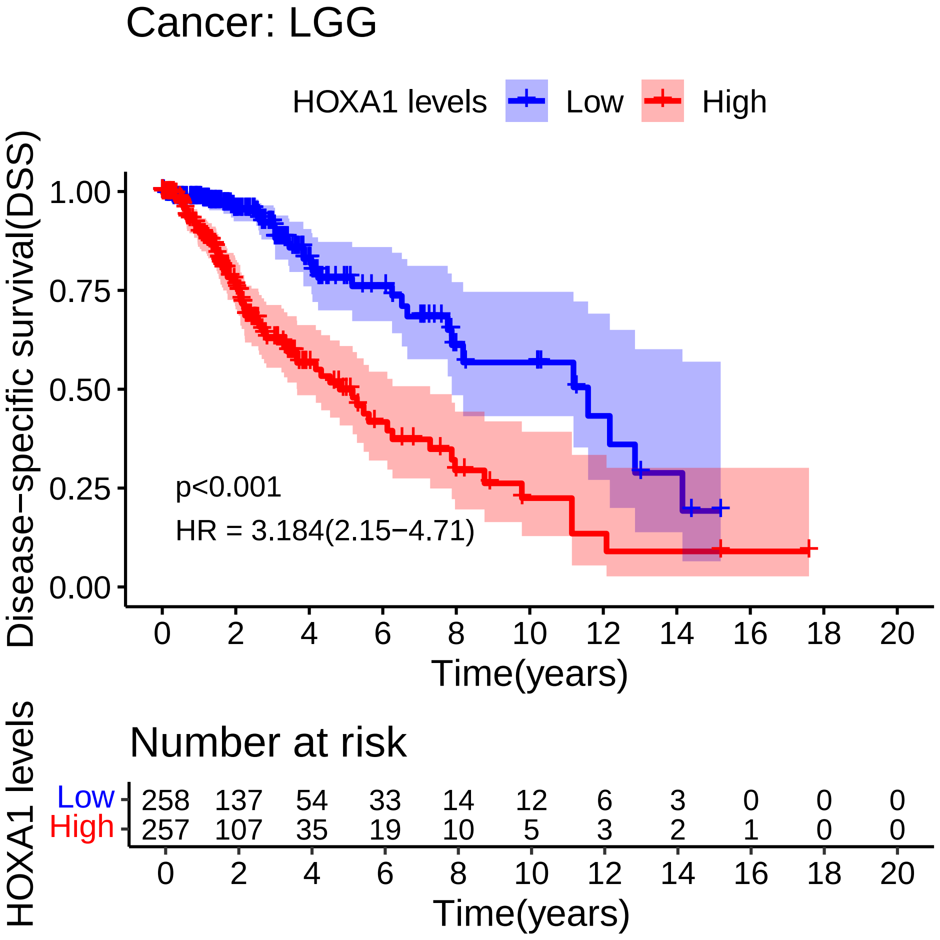
Cancer: LGG
HOXA1 levels
Low
High
Disease−specific survival(DSS)
1.00
0.75
0.50
0.25
0.00
p<0.001
HR = 3.184(2.15−4.71)
0
2
4
6
8
10
12
14
16
18
20
Time(years)
Number at risk
HOXA1 levels
Low 258 137 54 33 14 12 6 3 0 0 0
High 257 107 35 19 10 5 3 2 1 0 0
0
2
4
6
8
10
12
14
16
18
20
Time(years)

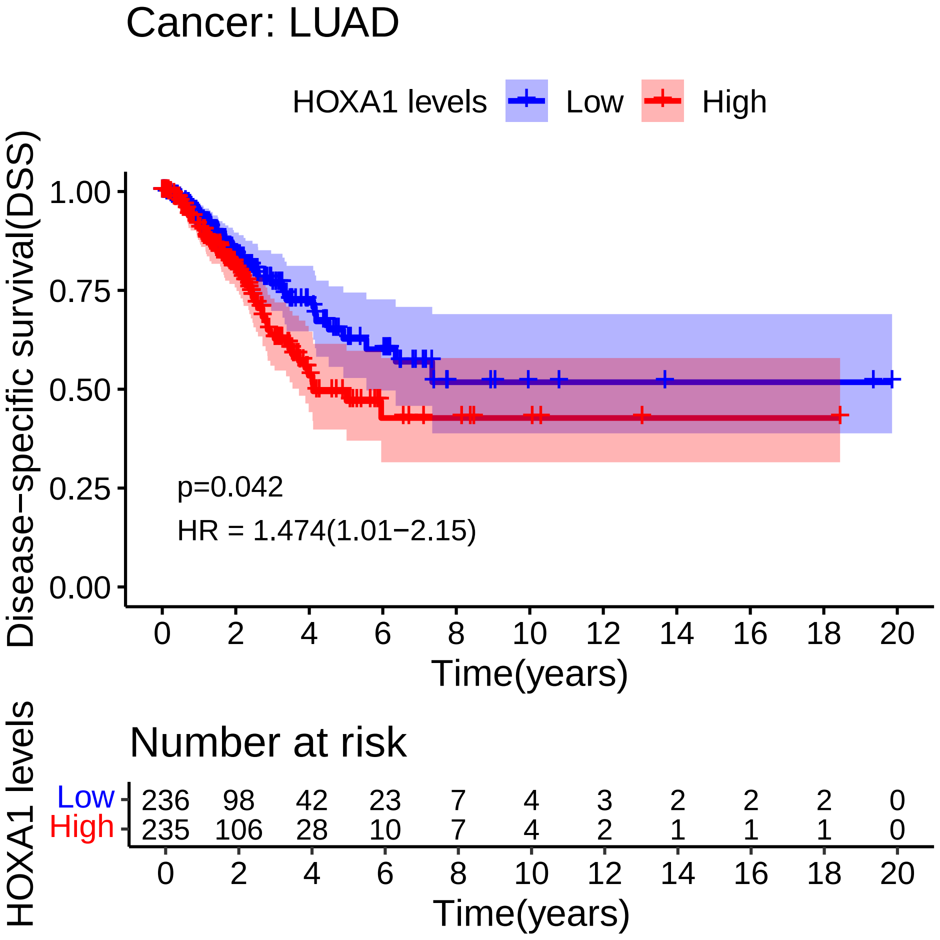
Cancer: LUAD
HOXA1 levels
Low
High
Disease−specific survival(DSS)
1.00
0.75
0.50
0.25
0.00
p=0.042
HR = 1.474(1.01−2.15)
0
2
4
6
8
10
12
14
16
18
20
Time(years)
Number at risk
HOXA1 levels
Low 236 98 42 23 7 4 3 2 2 2 0
High 235 106 28 10 7 4 2 1 1 1 0
0
2
4
6
8
10
12
14
16
18
20
Time(years)

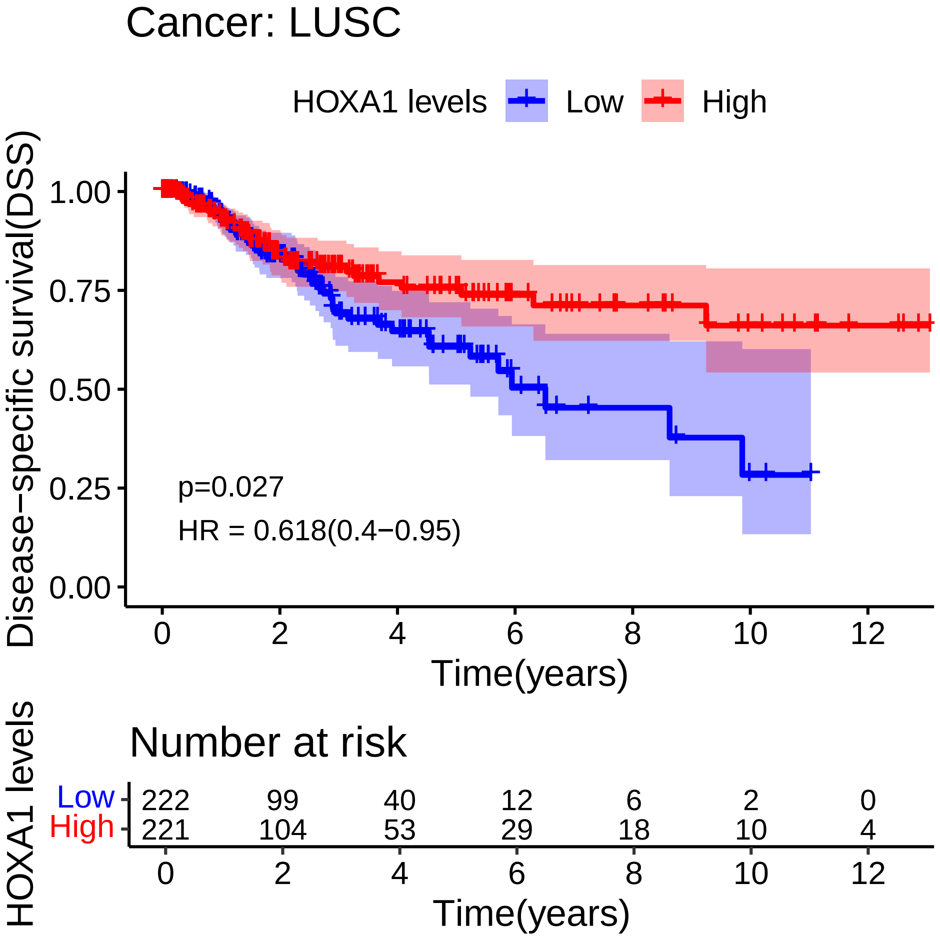

Cancer: LUSC
HOXA1 levels
Low
High
Disease−specific survival(DSS)
1.00
0.75
0.50
0.25
0.00
p=0.027
HR = 0.618(0.4−0.95)
0
2
4
6
8
10
12
Time(years)
Number at risk
HOXA1 levels
Low 222 99 40 12 6 2 0
High 221 104 53 29 18 10 4
0
2
4
6
8
10
12
Time(years)

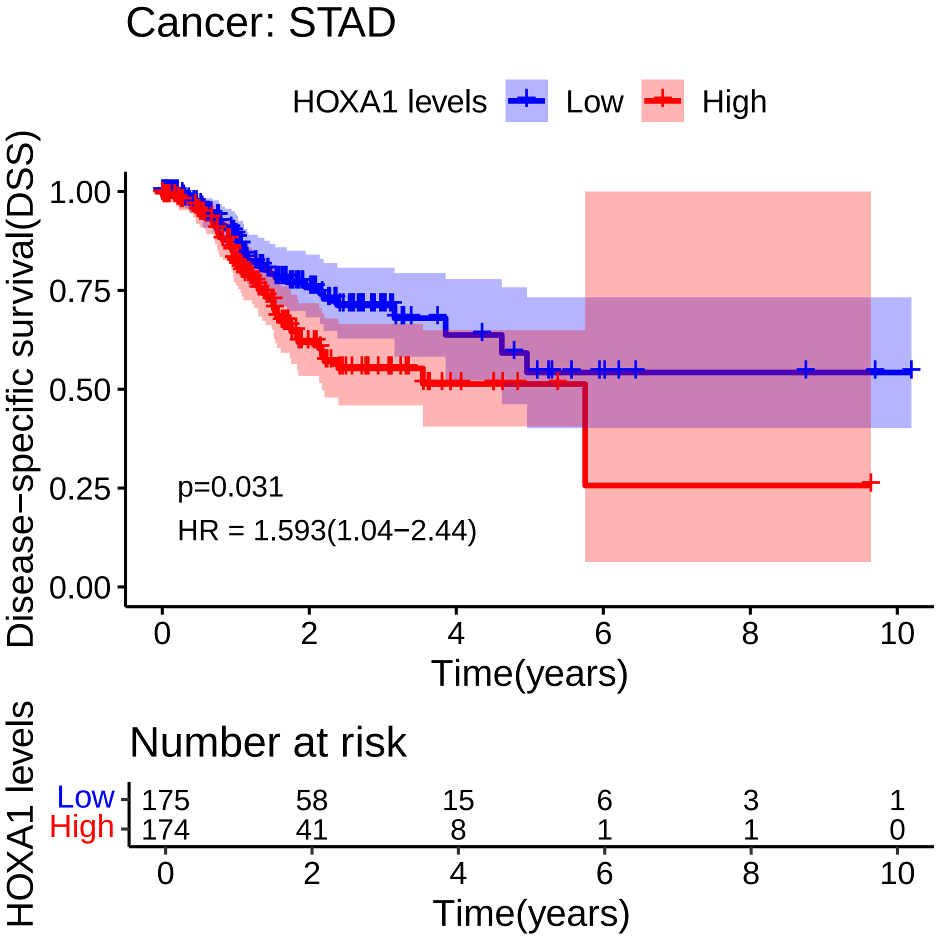
Cancer: STAD
HOXA1 levels
Low
High
Disease−specific survival(DSS)
1.00
0.75
0.50
0.25
0.00
p=0.031
HR = 1.593(1.04−2.44)
0
2
4
6
8
10
Time(years)
Number at risk
HOXA1 levels
Low
High
175 58 15 6 3 1
174 41 8 1 1 0
0
2
4
6
8
10
Time(years)

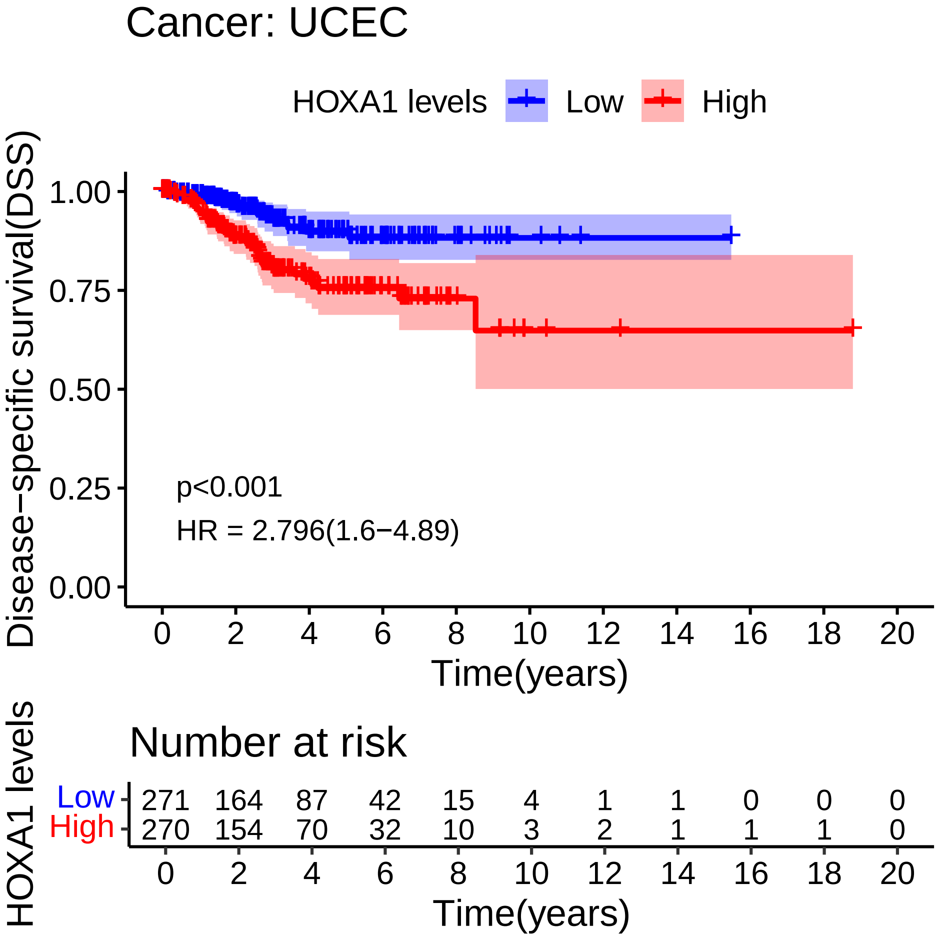

Cancer: UCEC
HOXA1 levels
Low
High
Disease−specific survival(DSS)
1.00
0.75
0.50
0.25
0.00
p<0.001
HR = 2.796(1.6−4.89)
0
2
4
6
8
10
12
14
16
18
20
Time(years)
Number at risk
HOXA1 levels
Low 271 164 87 42 15 4 1 1 0 0 0
High 270 154 70 32 10 3 2 1 1 1 0
Time(years)

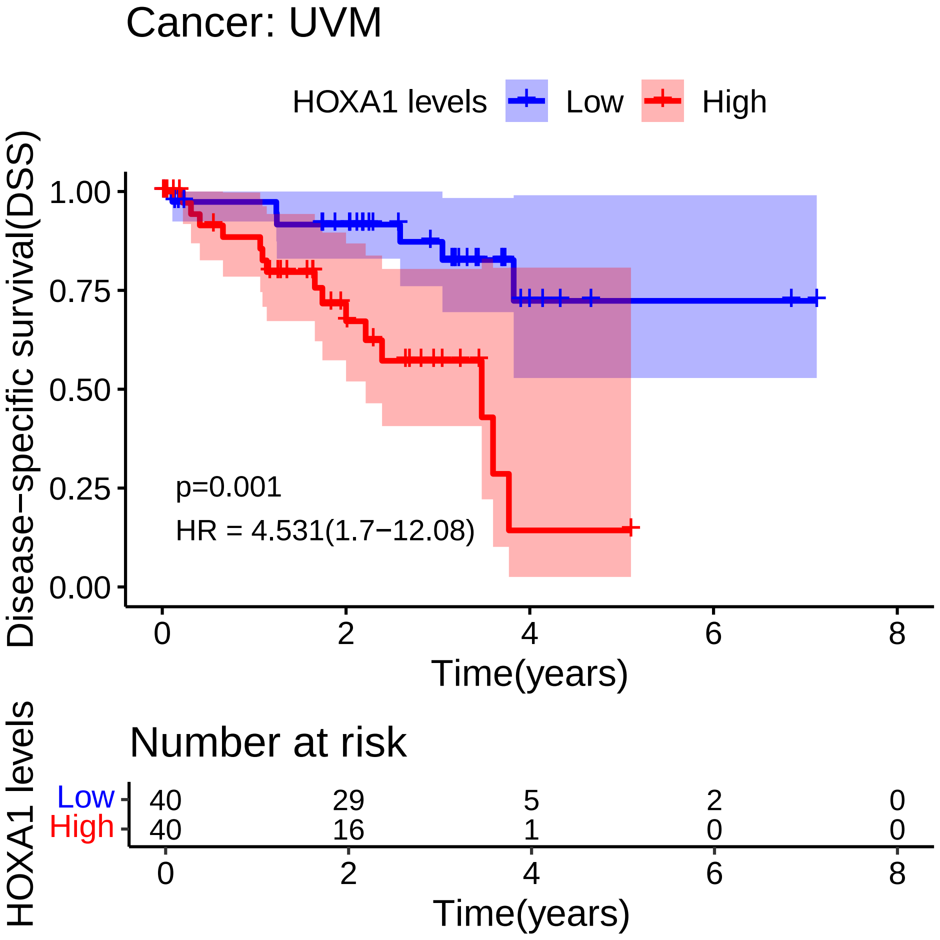
Cancer: UVM
HOXA1 levels
Low
High
Disease−specific survival(DSS)
1.00
0.75
0.50
0.25
0.00
p=0.001
HR = 4.531(1.7−12.08)
0
2
4
6
8
Time(years)
Number at risk
HOXA1 levels
Low 40 29 5 2 0
High 40 16 1 0 0
0
2
4
6
8
Time(years)

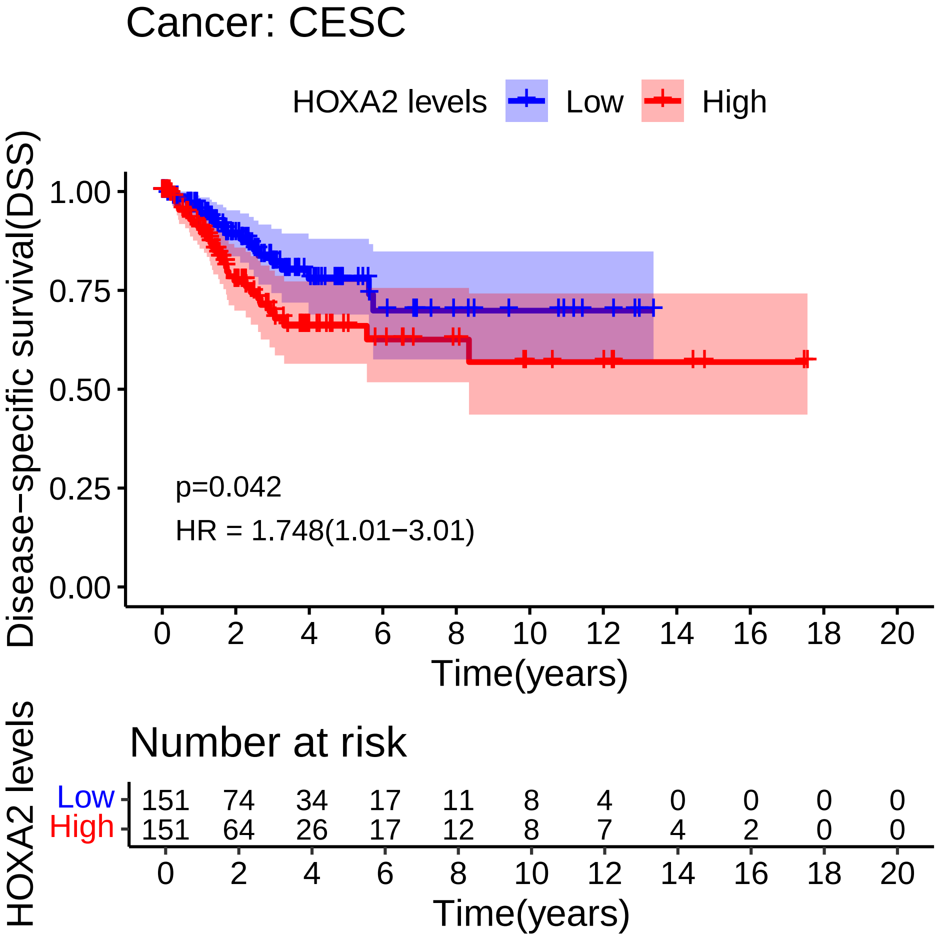

Cancer: CESC
HOXA2 levels
Low
High
Disease−specific survival(DSS)
1.00
0.75
0.50
0.25
0.00
p=0.042
HR = 1.748(1.01−3.01)
0
2
4
6
8
10
12
14
16
18
20
Time(years)
Number at risk
HOXA2 levels
Low 151 74 34 17 11 8 4 0 0 0 0
High 151 64 26 17 12 8 7 4 2 0 0
0
2
4
6
8
10
12
14
16
18
20
Time(years)

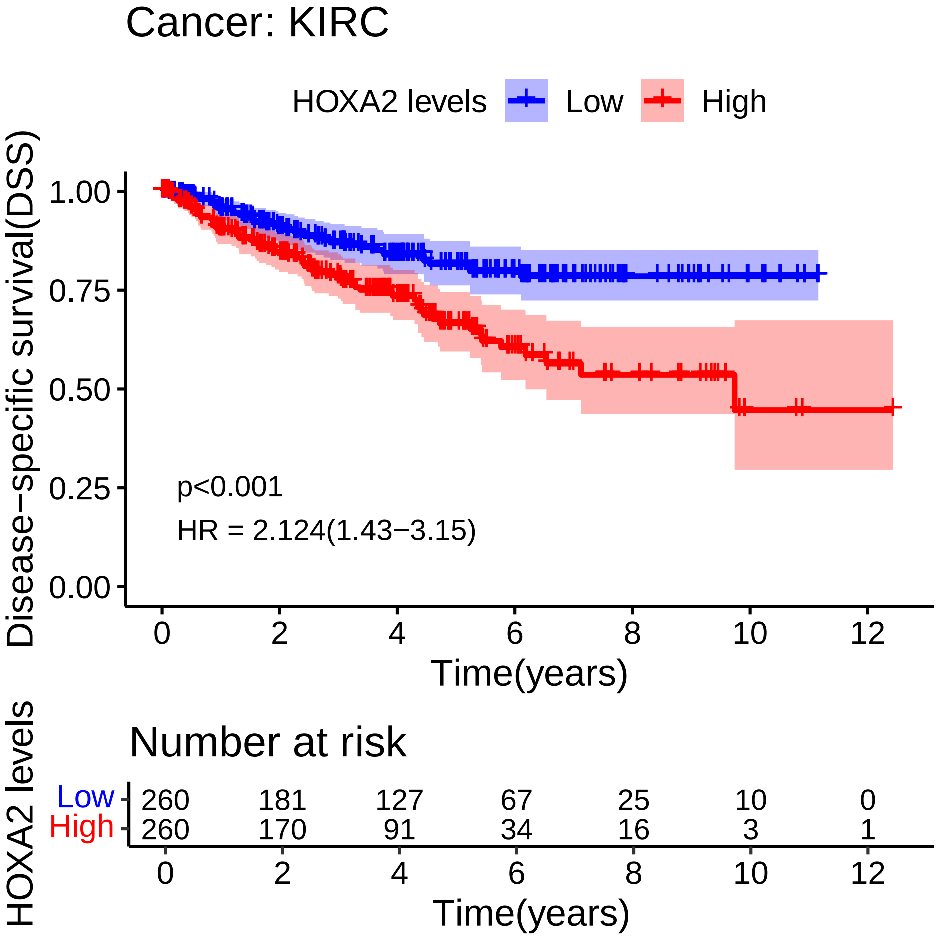

Cancer: KIRC
HOXA2 levels
Low
High
Disease−specific survival(DSS)
1.00
0.75
0.50
0.25
0.00
p<0.001
HR = 2.124(1.43−3.15)
0
2
4
6
8
10
12
Time(years)
Number at risk
HOXA2 levels
Low 260 181 127 67 25 10 0
High 260 170 91 34 16 3 1
Time(years)

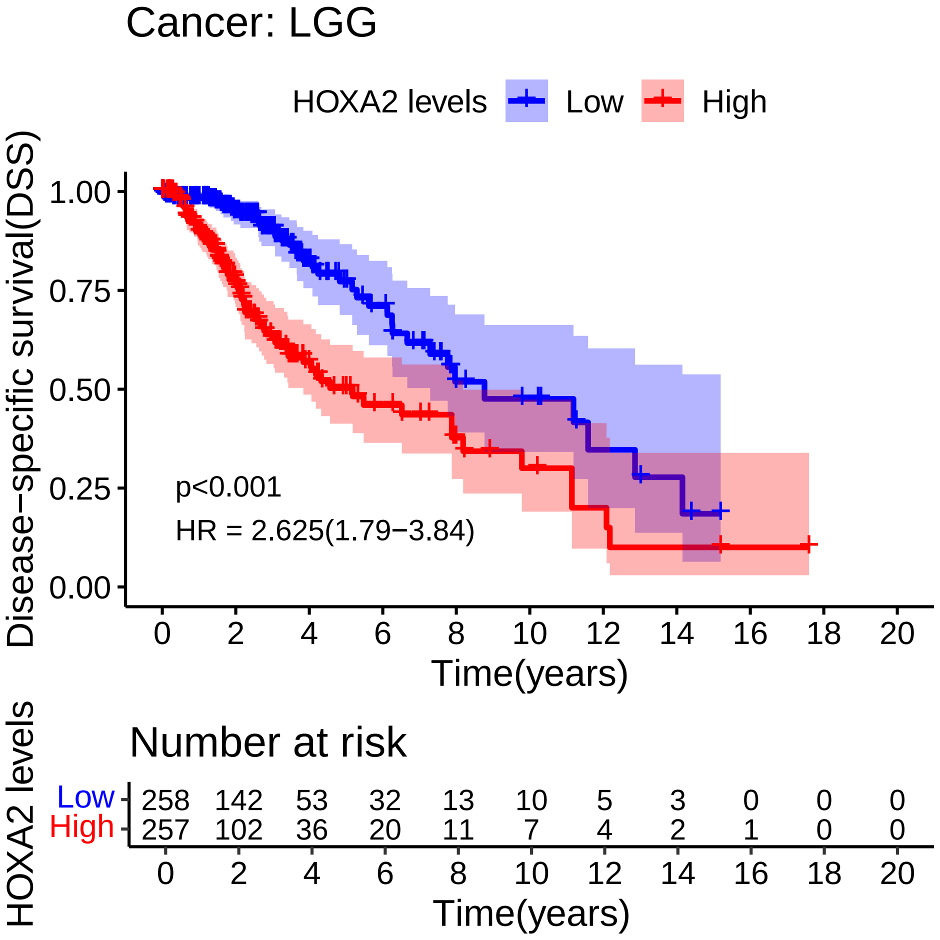

Cancer: LGG
HOXA2 levels
Low
High
Disease−specific survival(DSS)
1.00
0.75
0.50
0.25
0.00
p<0.001
HR = 2.625(1.79−3.84)
0
2
4
6
8
10
12
14
16
18
20
Time(years)
Number at risk
HOXA2 levels
Low
High
258 142 53 32 13 10 5 3 0 0 0
257 102 36 20 11 7 4 2 1 0 0
0
2
4
6
8
10
12
14
16
18
20
Time(years)

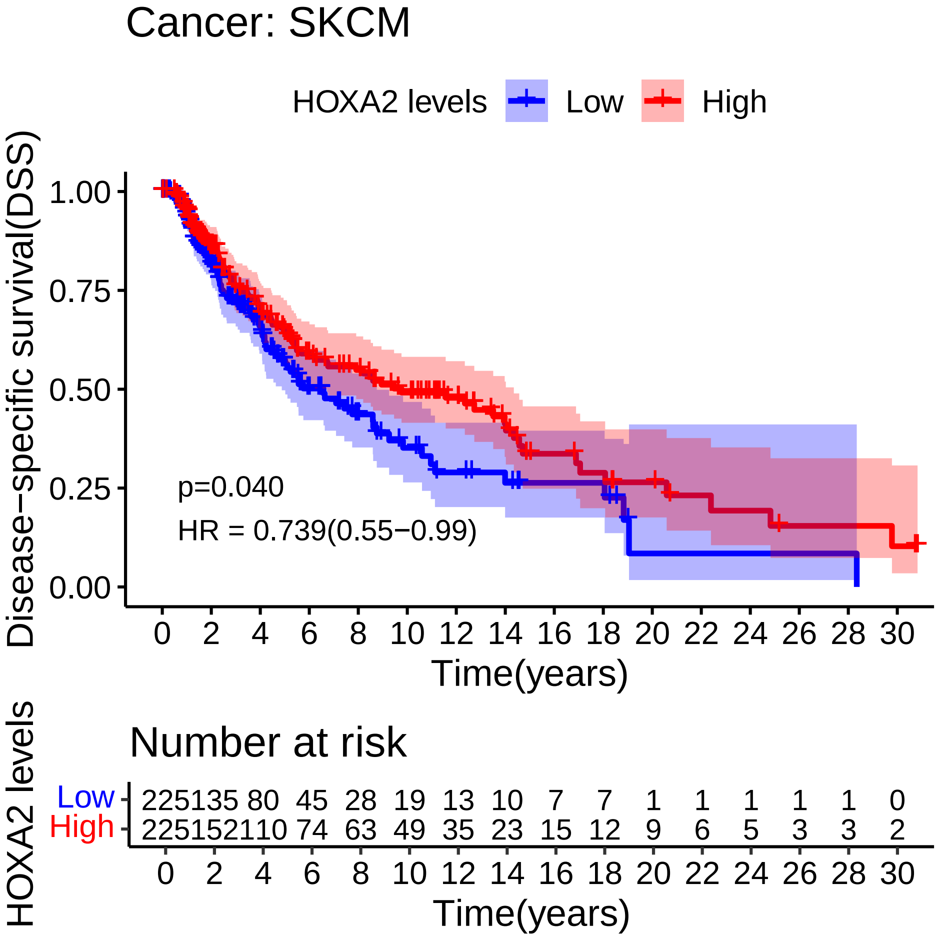

Cancer: SKCM
HOXA2 levels
Low
High
Disease−specific survival(DSS)
1.00
0.75
0.50
0.25
0.00
p=0.040
HR = 0.739(0.55−0.99)
0 2 4 6 8 10 12 14 16 18 20 22 24 26 28 30
Time(years)
Number at risk
HOXA2 levels
Low 225 135 80 45 28 19 13 10 7 7 1 1 1 1 1 0
High 225 152 110 74 63 49 35 23 15 12 9 6 5 3 3 2
0 2 4 6 8 10 12 14 16 18 20 22 24 26 28 30
Time(years)

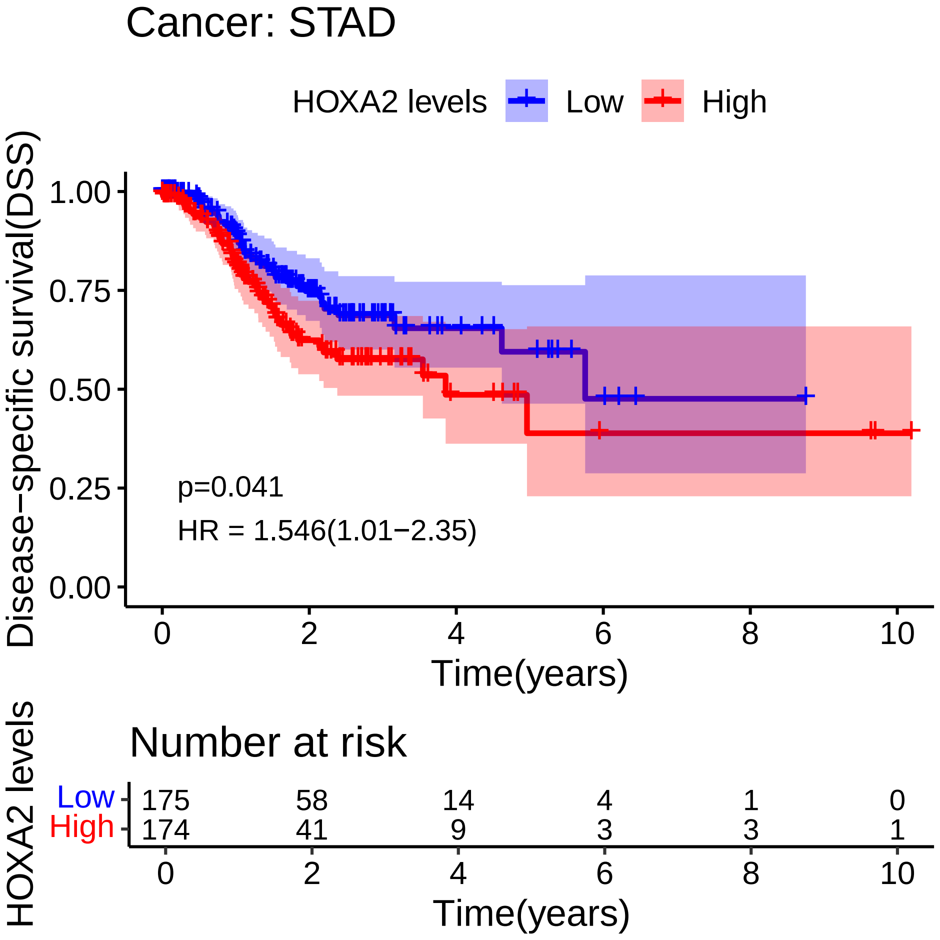

Cancer: STAD
HOXA2 levels
Low
High
Disease−specific survival(DSS)
1.00
0.75
0.50
0.25
0.00
p=0.041
HR = 1.546(1.01−2.35)
0
2
4
6
8
10
Time(years)
Number at risk
HOXA2 levels
Low 175 58 14 4 1 0
High 174 41 9 3 3 1
Time(years)

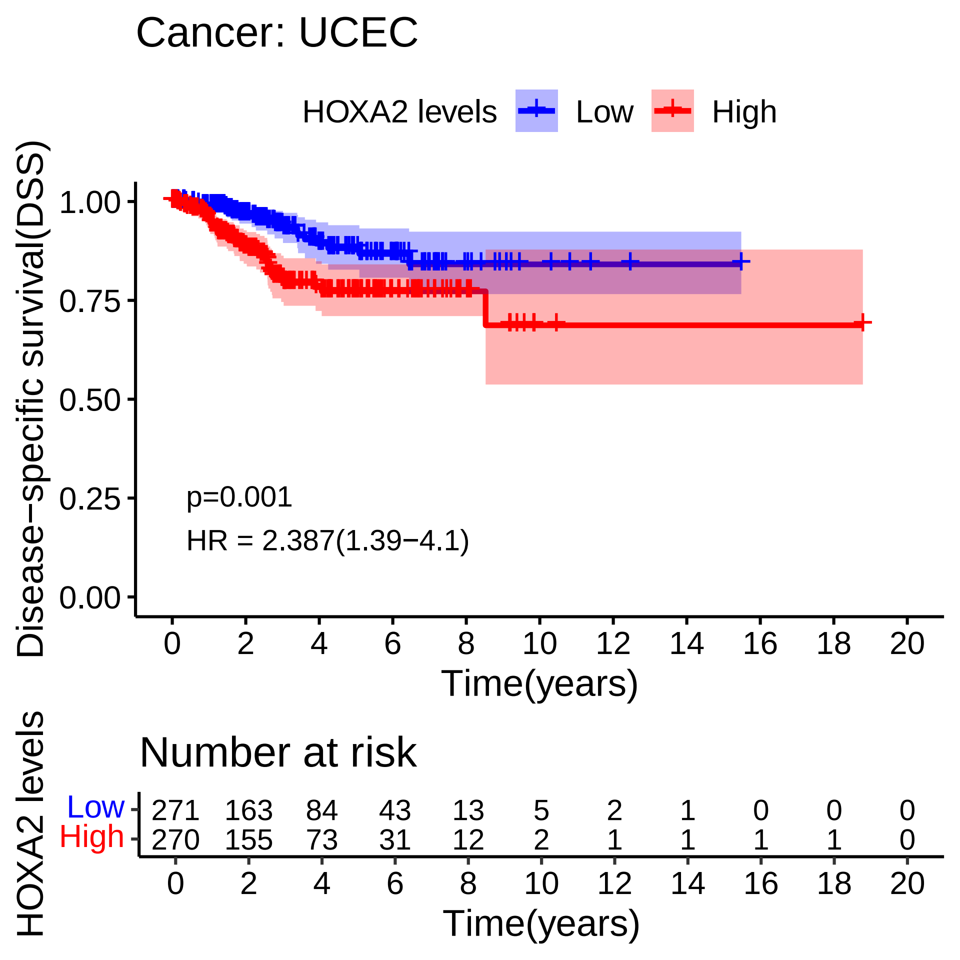

Cancer: UCEC
HOXA2 levels
Low
High
Disease−specific survival(DSS)
1.00
0.75
0.50
0.25
0.00
p=0.001
HR = 2.387(1.39−4.1)
0
2
4
6
8
10
12
14
16
18
20
Time(years)
Number at risk
HOXA2 levels
Low
High
271 163 84 43 13 5 2 1 0 0 0
270 155 73 31 12 2 1 1 1 1 0
0
2
4
6
8
10
12
14
16
18
20
Time(years)

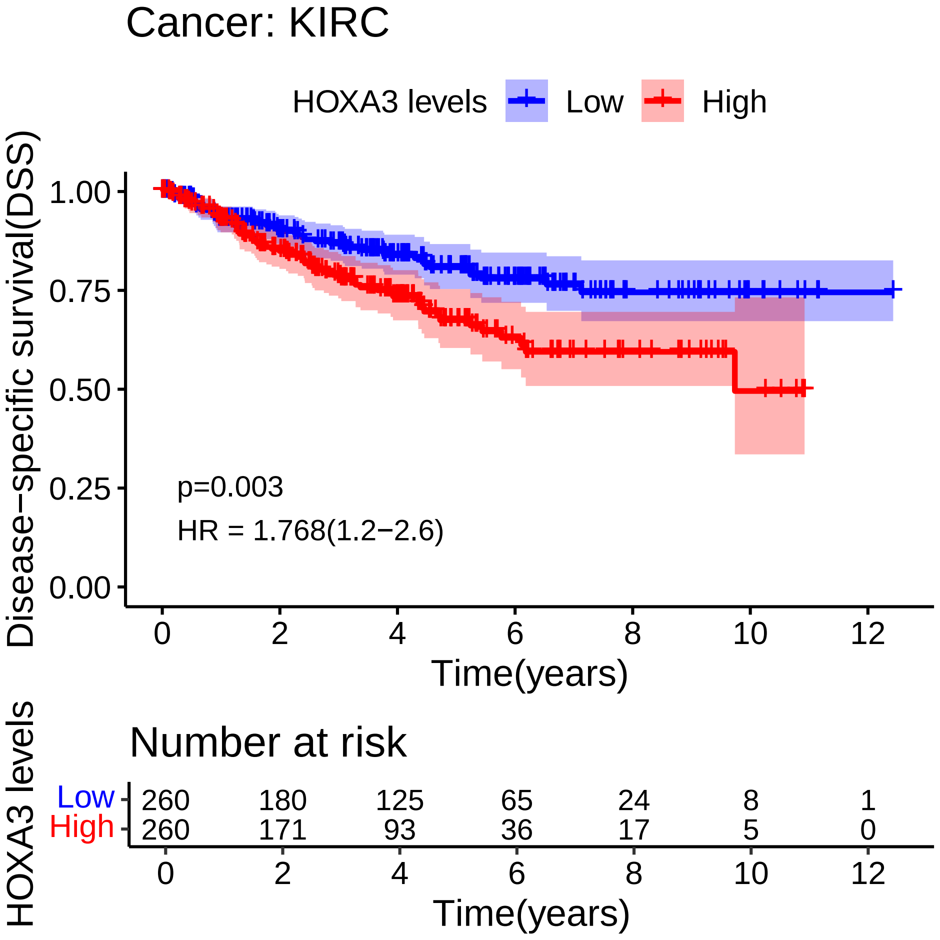

Cancer: KIRC
HOXA3 levels
Low
High
Disease−specific survival(DSS)
1.00
0.75
0.50
0.25
0.00
p=0.003
HR = 1.768(1.2−2.6)
0
2
4
6
8
10
12
Time(years)
Number at risk
HOXA3 levels
Low 260 180 125 65 24 8 1
High 260 171 93 36 17 5 0
0
2
4
6
8
10
12
Time(years)

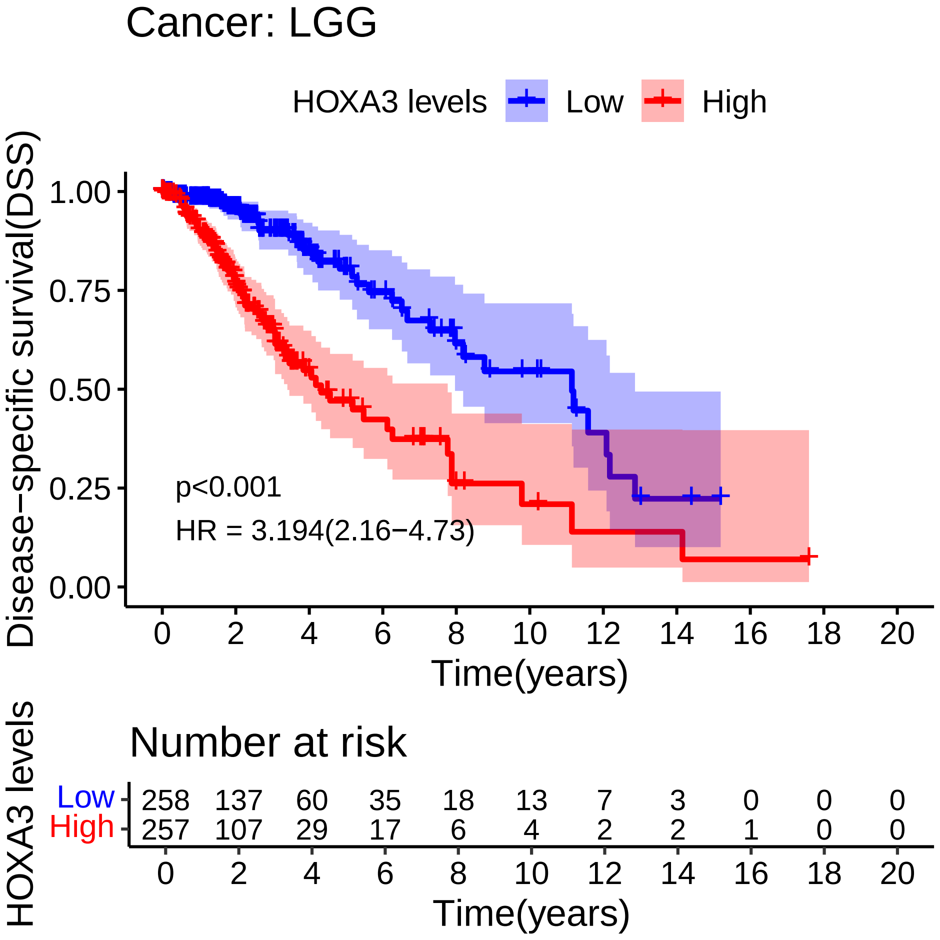

Cancer: LGG
HOXA3 levels
Low
High
Disease−specific survival(DSS)
1.00
0.75
0.50
0.25
0.00
p<0.001
HR = 3.194(2.16−4.73)
0 2 4 6 8 10 12 14 16 18 20
Time(years)
Number at risk
HOXA3 levels
Low 258 137 60 35 18 13 7 3 0 0 0
High 257 107 29 17 6 4 2 2 1 0 0
0 2 4 6 8 10 12 14 16 18 20
Time(years)

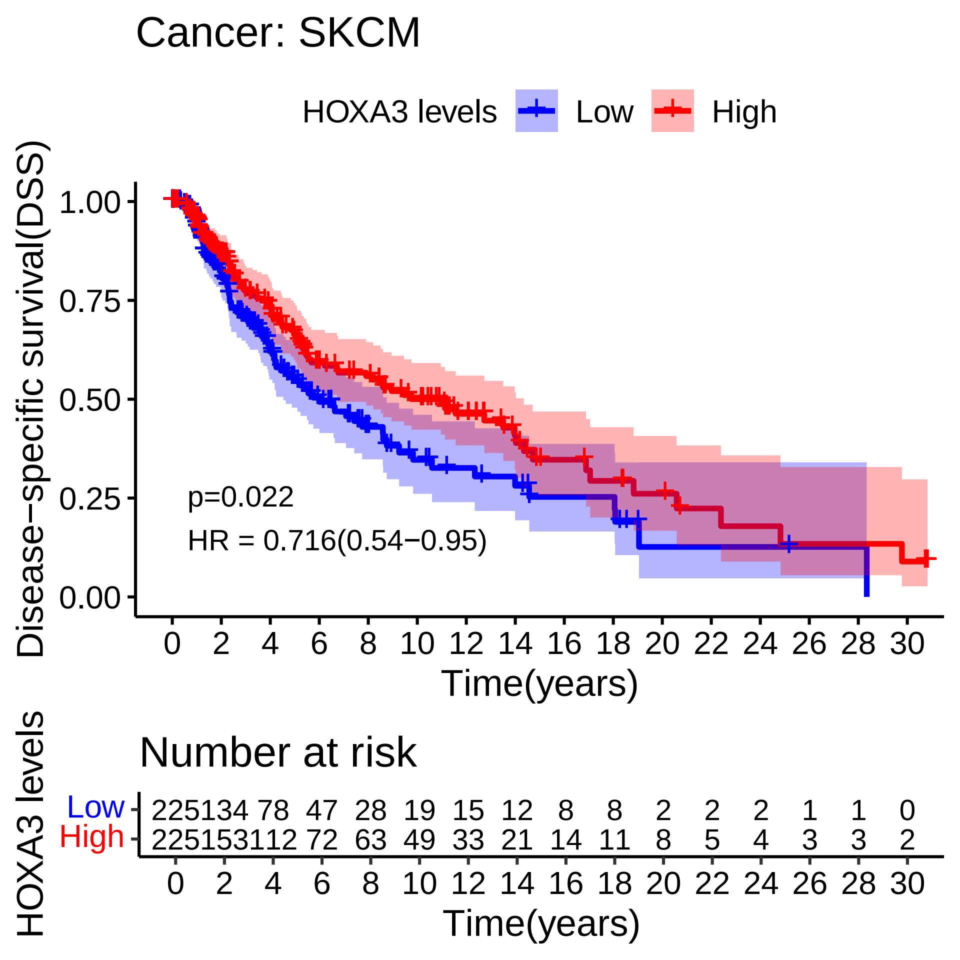
Cancer: SKCM
HOXA3 levels
Low
High
Disease−specific survival(DSS)
1.00
0.75
0.50
0.25
0.00
p=0.022
HR = 0.716(0.54−0.95)
0 2 4 6 8 10 12 14 16 18 20 22 24 26 28 30
Time(years)
Number at risk
HOXA3 levels
Low 225 134 78 47 28 19 15 12 8 8 2 2 2 1 1 0
High 225 153 112 72 63 49 33 21 14 11 8 5 4 3 3 2
0 2 4 6 8 10 12 14 16 18 20 22 24 26 28 30
Time(years)

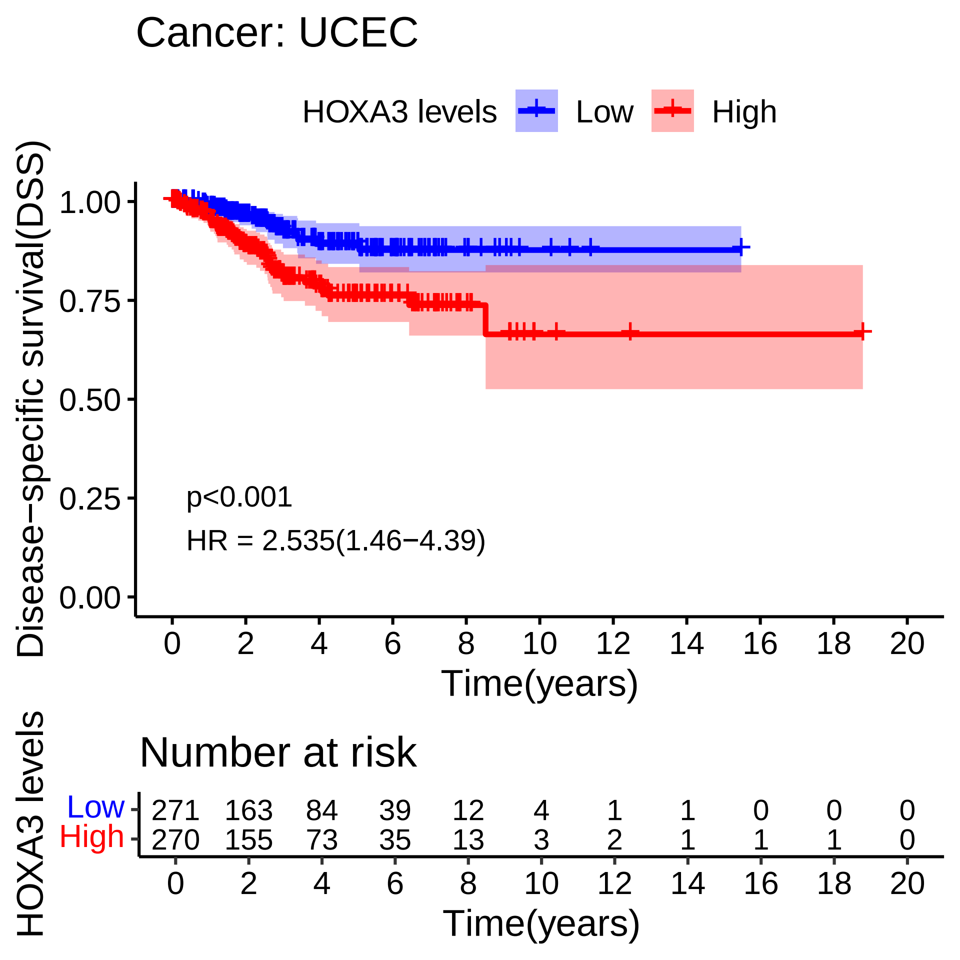
Cancer: UCEC
HOXA3 levels
Low
High
Disease−specific survival(DSS)
1.00
0.75
0.50
0.25
0.00
p<0.001
HR = 2.535(1.46−4.39)
0 2 4 6 8 10 12 14 16 18 20
Time(years)
Number at risk
HOXA3 levels
Low 271 163 84 39 12 4 1 1 0 0 0
High 270 155 73 35 13 3 2 1 1 1 0
0 2 4 6 8 10 12 14 16 18 20
Time(years)

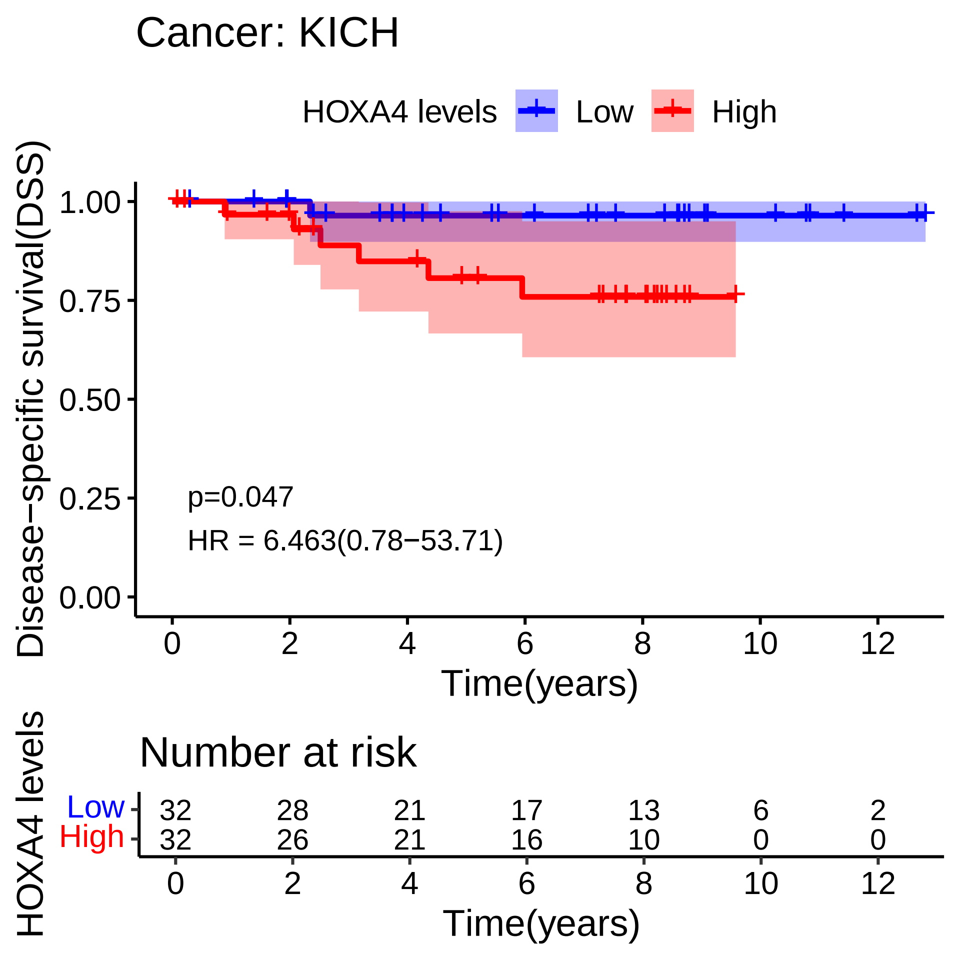
Cancer: KICH
HOXA4 levels
Low
High
Disease−specific survival(DSS)
1.00
0.75
0.50
0.25
0.00
p=0.047
HR = 6.463(0.78−53.71)
0
2
4
6
8
10
12
Time(years)
Number at risk
HOXA4 levels
Low
High
32 28 21 17 13 6 2
32 26 21 16 10 0 0
Time(years)

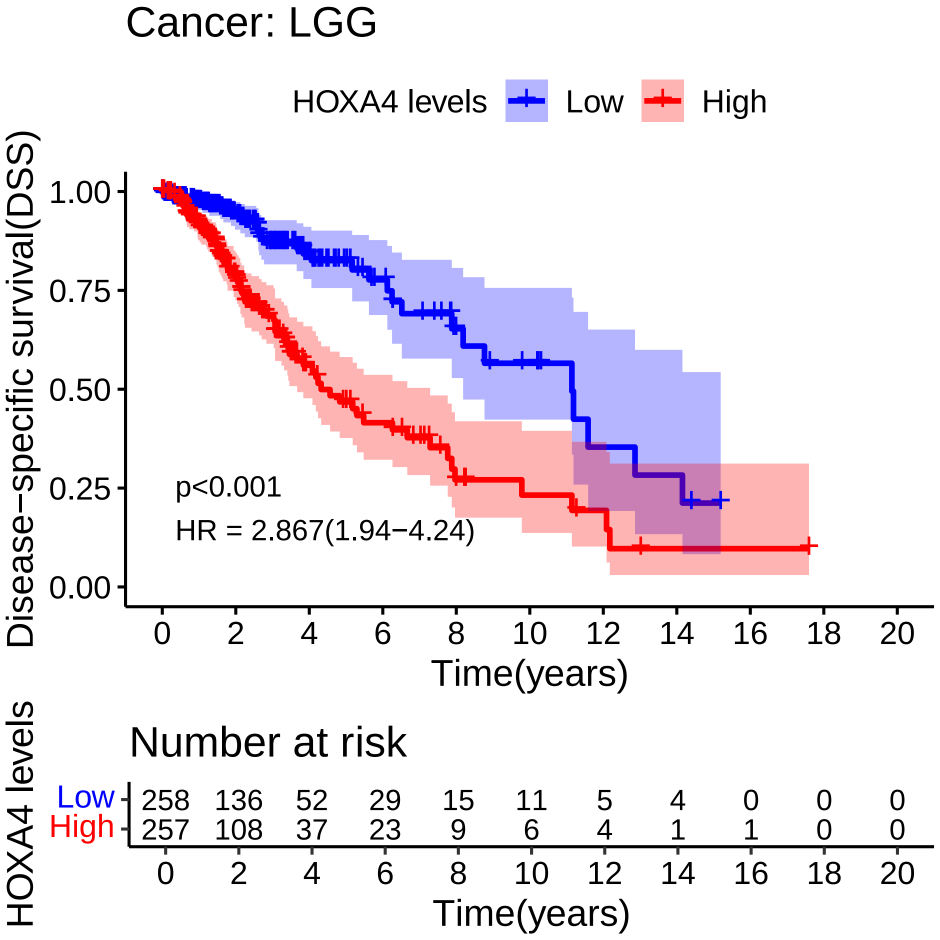

Cancer: LGG
HOXA4 levels
Low
High
Disease−specific survival(DSS)
1.00
0.75
0.50
0.25
0.00
p<0.001
HR = 2.867(1.94−4.24)
0
2
4
6
8
10
12
14
16
18
20
Time(years)
Number at risk
HOXA4 levels
Low 258 136 52 29 15 11 5 4 0 0 0
High 257 108 37 23 9 6 4 1 1 0 0
Time(years)

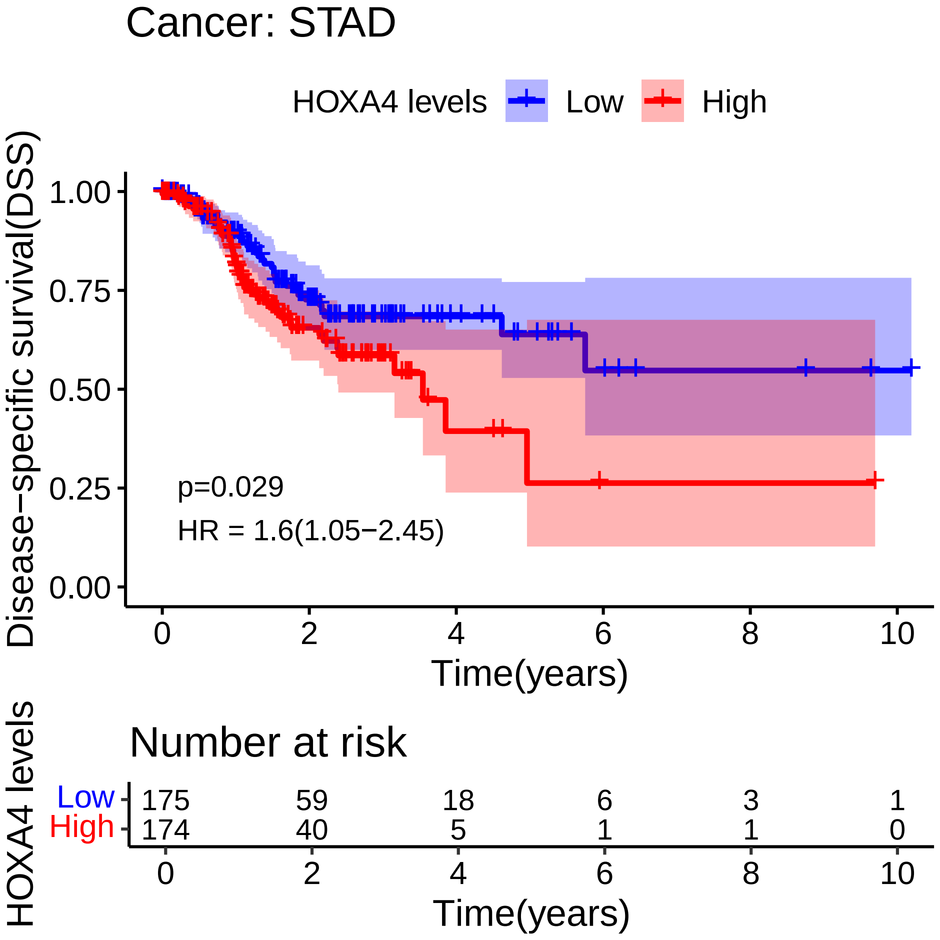

Cancer: STAD
HOXA4 levels
Low
High
Disease−specific survival(DSS)
1.00
0.75
0.50
0.25
0.00
p=0.029
HR = 1.6(1.05−2.45)
0
2
4
6
8
10
Time(years)
Number at risk
HOXA4 levels
Low 175 59 18 6 3 1
High 174 40 5 1 1 0
0
2
4
6
8
10
Time(years)

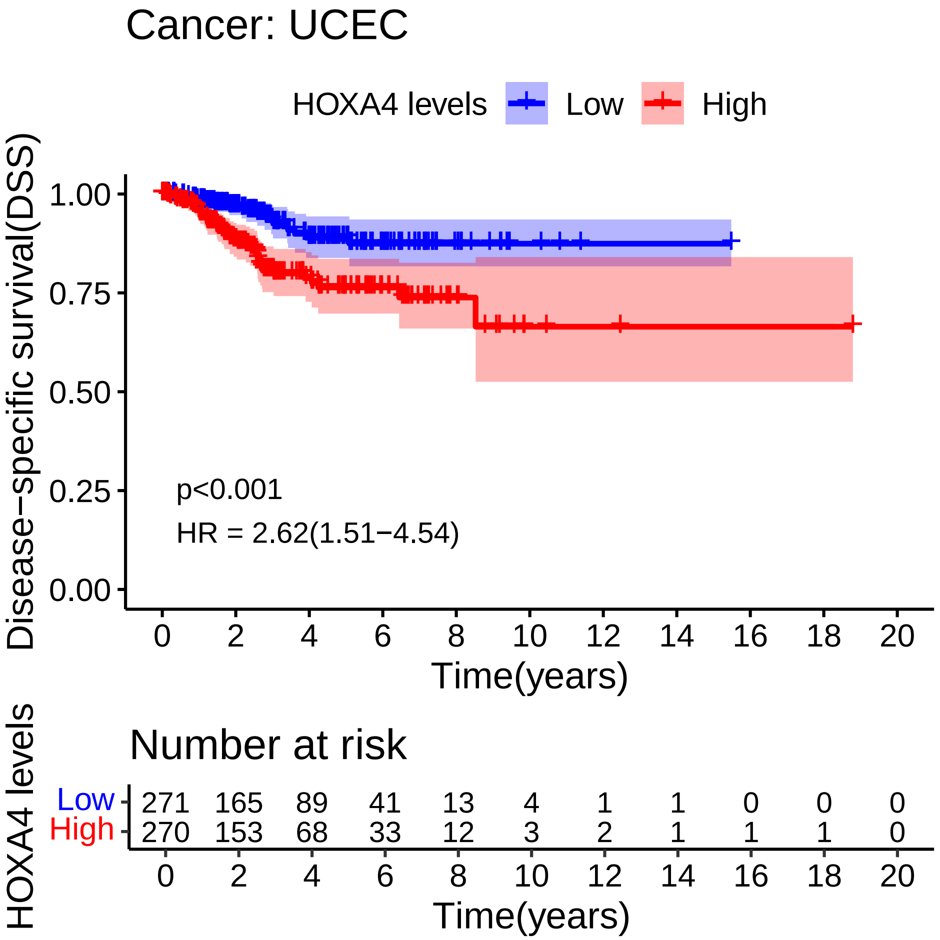
Cancer: UCEC
HOXA4 levels
Low
High
Disease−specific survival(DSS)
1.00
0.75
0.50
0.25
0.00
p<0.001
HR = 2.62(1.51−4.54)
0
2
4
6
8
10
12
14
16
18
20
Time(years)
Number at risk
HOXA4 levels
Low 271 165 89 41 13 4 1 1 0 0 0
High 270 153 68 33 12 3 2 1 1 1 0
Time(years)

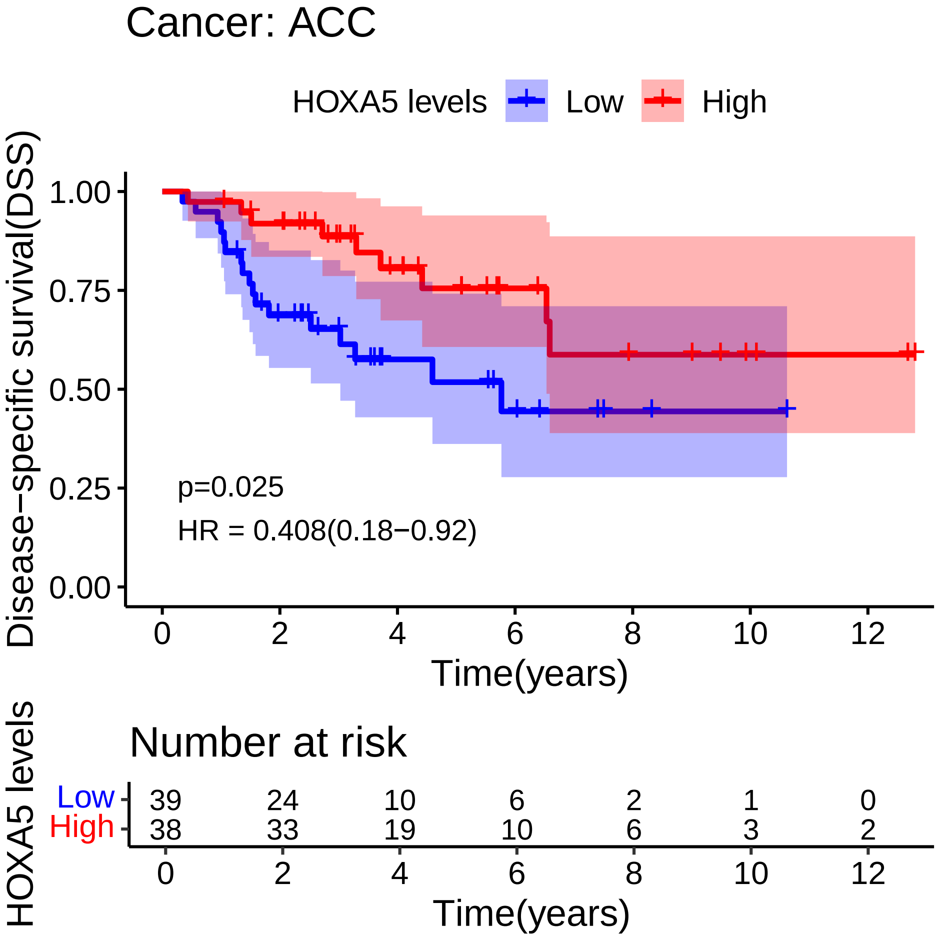
Cancer: ACC
HOXA5 levels
Low
High
Disease−specific survival(DSS)
1.00
0.75
0.50
0.25
0.00
p=0.025
HR = 0.408(0.18−0.92)
0
2
4
6
8
10
12
Time(years)
Number at risk
HOXA5 levels
Low
High
39 24 10 6 2 1 0
38 33 19 10 6 3 2
0
2
4
6
8
10
12
Time(years)

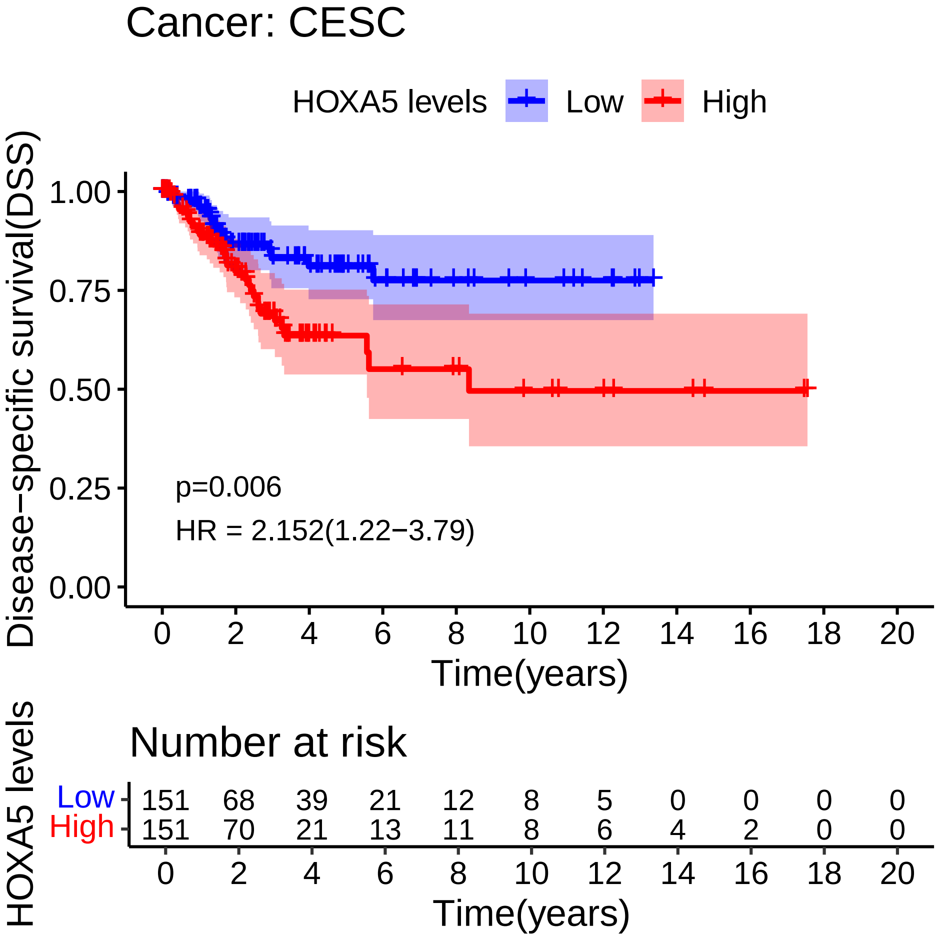

Cancer: CESC
HOXA5 levels
Low
High
Disease−specific survival(DSS)
1.00
0.75
0.50
0.25
0.00
p=0.006
HR = 2.152(1.22−3.79)
0
2
4
6
8
10
12
14
16
18
20
Time(years)
Number at risk
HOXA5 levels
Low
High
151 68 39 21 12 8 5 0 0 0 0
151 70 21 13 11 8 6 4 2 0 0
0
2
4
6
8
10
12
14
16
18
20
Time(years)

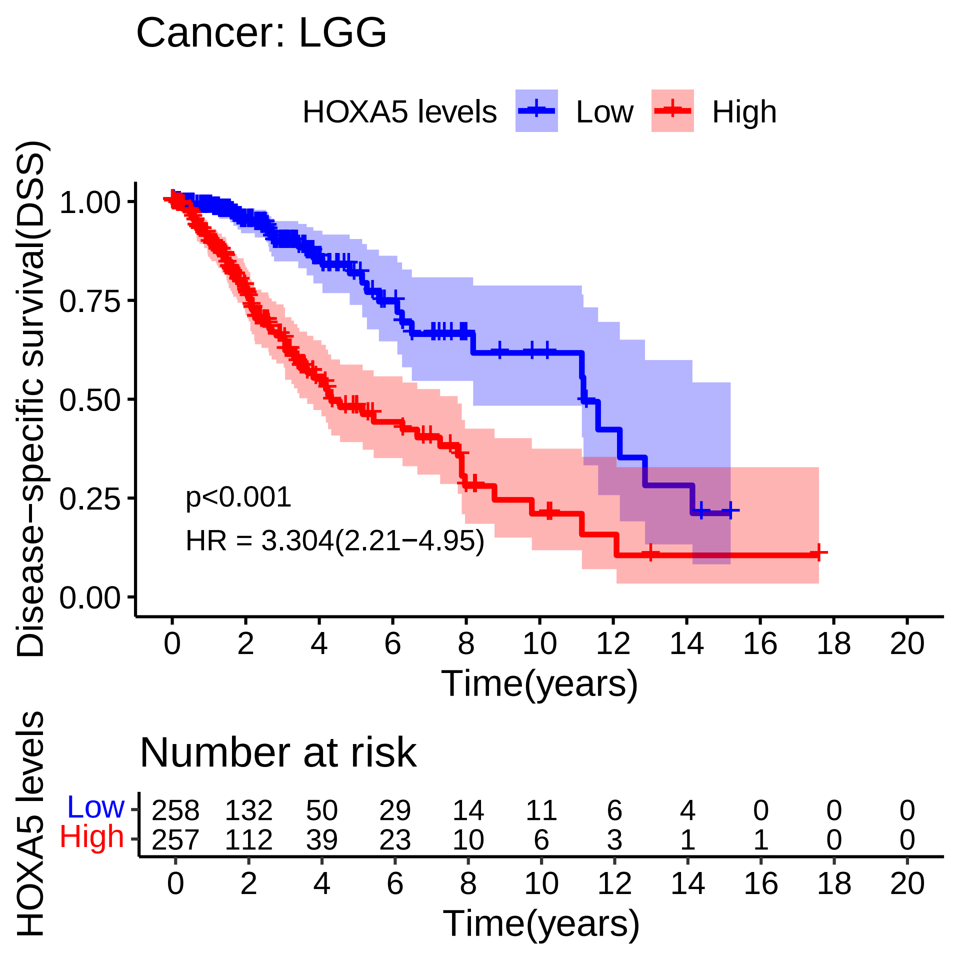
Cancer: LGG
HOXA5 levels
Low
High
Disease−specific survival(DSS)
1.00
0.75
0.50
0.25
0.00
p<0.001
HR = 3.304(2.21−4.95)
0 2 4 6 8 10 12 14 16 18 20
Time(years)
Number at risk
HOXA5 levels
Low 258 132 50 29 14 11 6 4 0 0 0
High 257 112 39 23 10 6 3 1 1 0 0
0 2 4 6 8 10 12 14 16 18 20
Time(years)

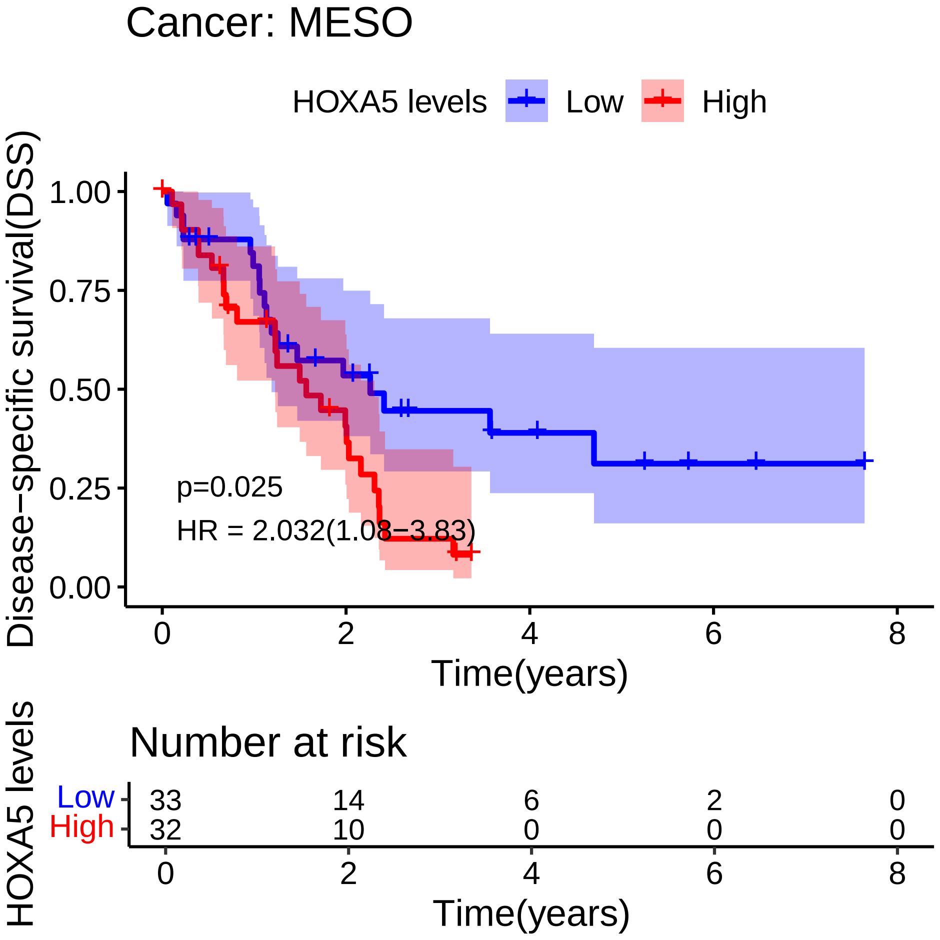
Cancer: MESO
HOXA5 levels
Low
High
Disease−specific survival(DSS)
1.00
0.75
0.50
0.25
0.00
p=0.025
HR = 2.032(1.08−3.83)
0
2
4
6
8
Time(years)
Number at risk
HOXA5 levels
Low 33 14 6 2 0
High 32 10 0 0 0
Time(years)

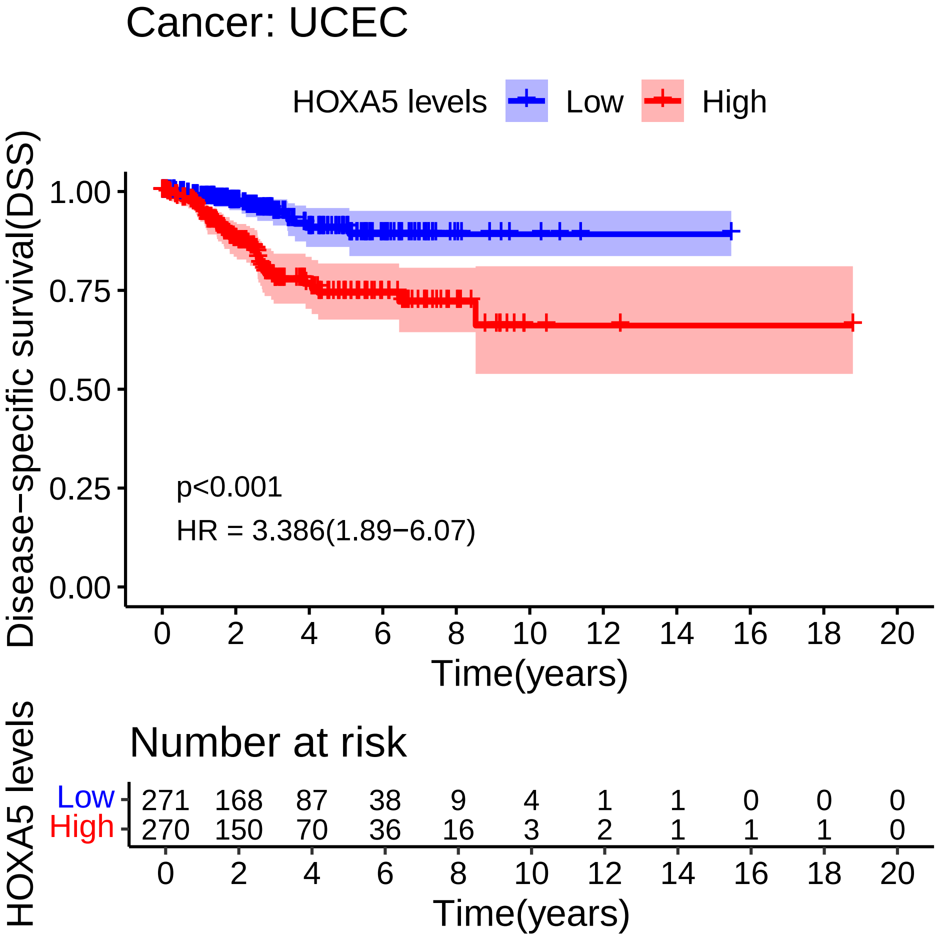

Cancer: UCEC
HOXA5 levels
Low
High
Disease−specific survival(DSS)
1.00
0.75
0.50
0.25
0.00
p<0.001
HR = 3.386(1.89−6.07)
0 2 4 6 8 10 12 14 16 18 20
Time(years)
Number at risk
HOXA5 levels
Low 271 168 87 38 9 4 1 1 0 0 0
High 270 150 70 36 16 3 2 1 1 1 0
0 2 4 6 8 10 12 14 16 18 20
Time(years)

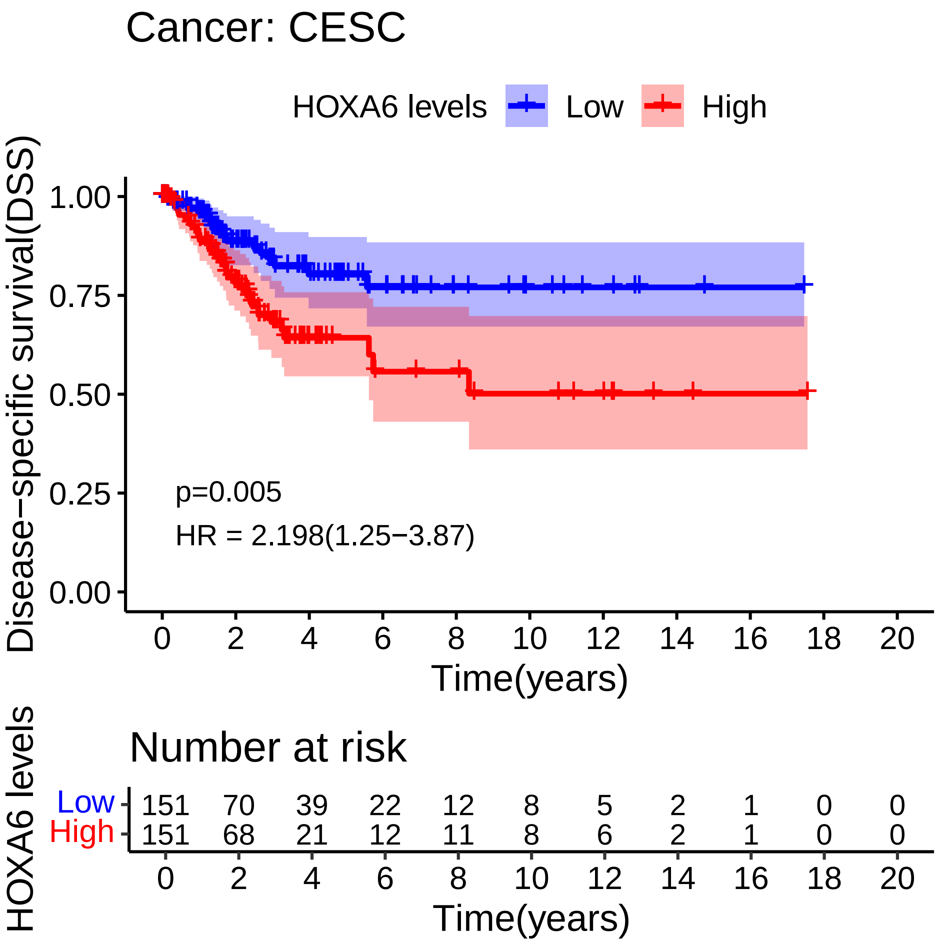
Cancer: CESC
HOXA6 levels
Low
High
Disease−specific survival(DSS)
1.00
0.75
0.50
0.25
0.00
p=0.005
HR = 2.198(1.25−3.87)
0
2
4
6
8
10
12
14
16
18
20
Time(years)
Number at risk
HOXA6 levels
Low
High
151 70 39 22 12 8 5 2 1 0 0
151 68 21 12 11 8 6 2 1 0 0
Time(years)

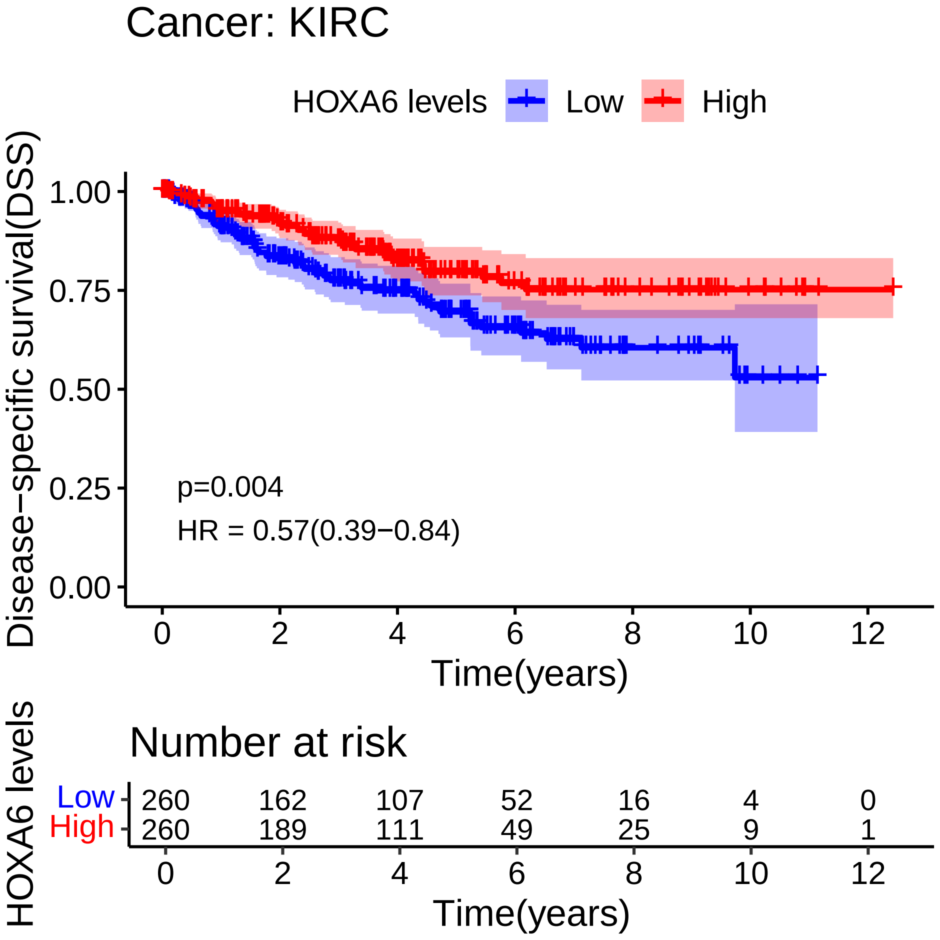
Cancer: KIRC
HOXA6 levels
Low
High
Disease−specific survival(DSS)
1.00
0.75
0.50
0.25
0.00
p=0.004
HR = 0.57(0.39−0.84)
0
2
4
6
8
10
12
Time(years)
Number at risk
HOXA6 levels
Low 260 162 107 52 16 4 0
High 260 189 111 49 25 9 1
0
2
4
6
8
10
12
Time(years)

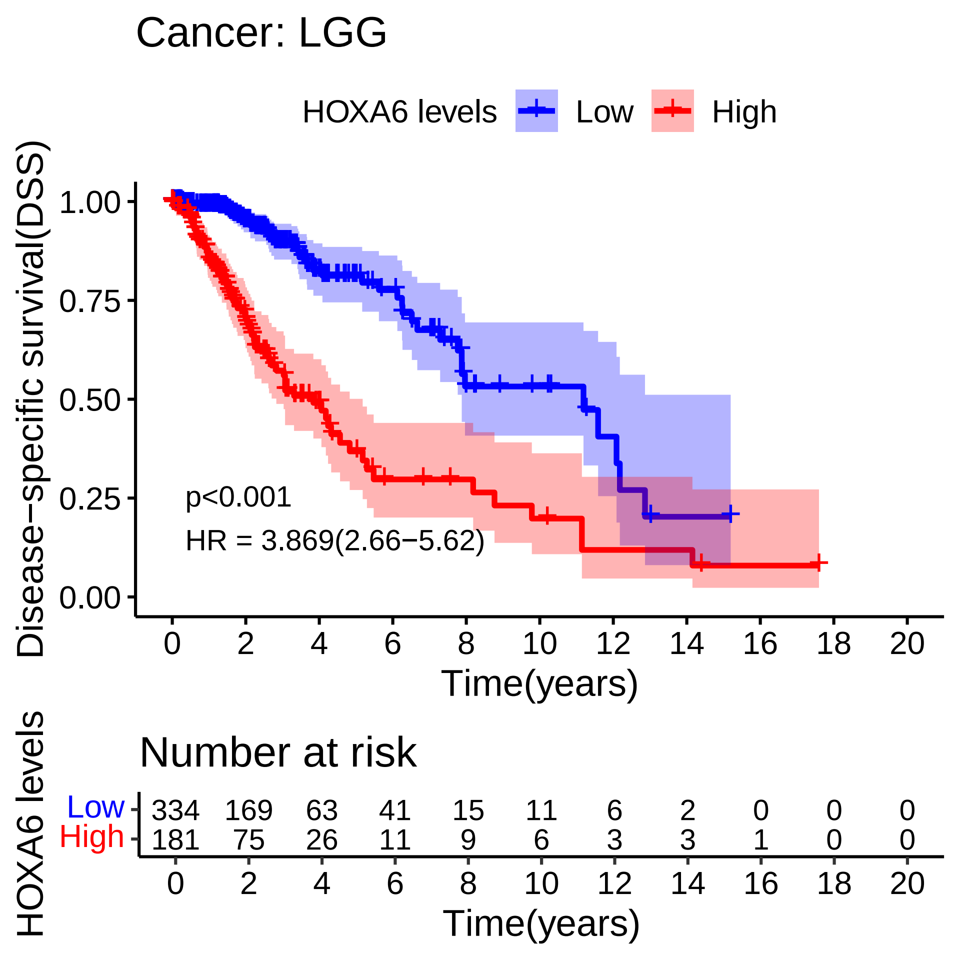

Cancer: LGG
HOXA6 levels
Low
High
Disease−specific survival(DSS)
1.00
0.75
0.50
0.25
0.00
p<0.001
HR = 3.869(2.66−5.62)
0 2 4 6 8 10 12 14 16 18 20
Time(years)
Number at risk
HOXA6 levels
Low 334 169 63 41 15 11 6 2 0 0 0
High 181 75 26 11 9 6 3 3 1 0 0
0 2 4 6 8 10 12 14 16 18 20
Time(years)

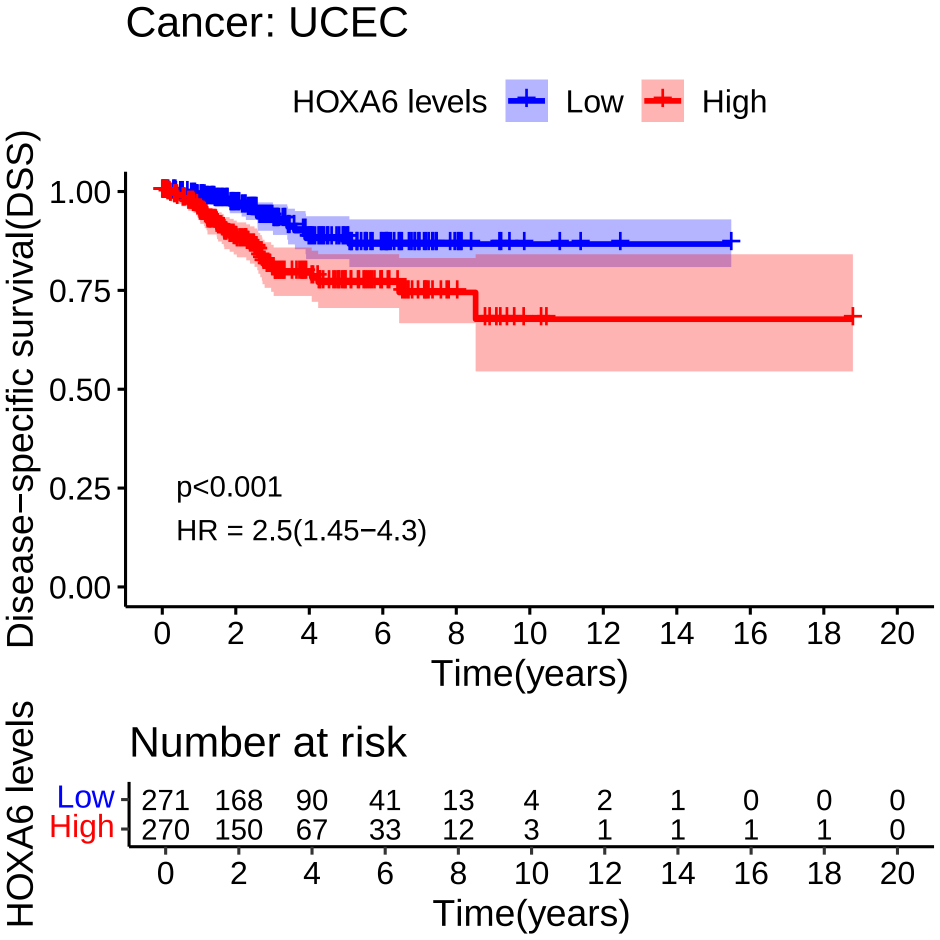
Cancer: UCEC
HOXA6 levels
Low
High
Disease−specific survival(DSS)
1.00
0.75
0.50
0.25
0.00
p<0.001
HR = 2.5(1.45−4.3)
0
2
4
6
8
10
12
14
16
18
20
Time(years)
Number at risk
HOXA6 levels
Low 271 168 90 41 13 4 2 1 0 0 0
High 270 150 67 33 12 3 1 1 1 1 0
Time(years)

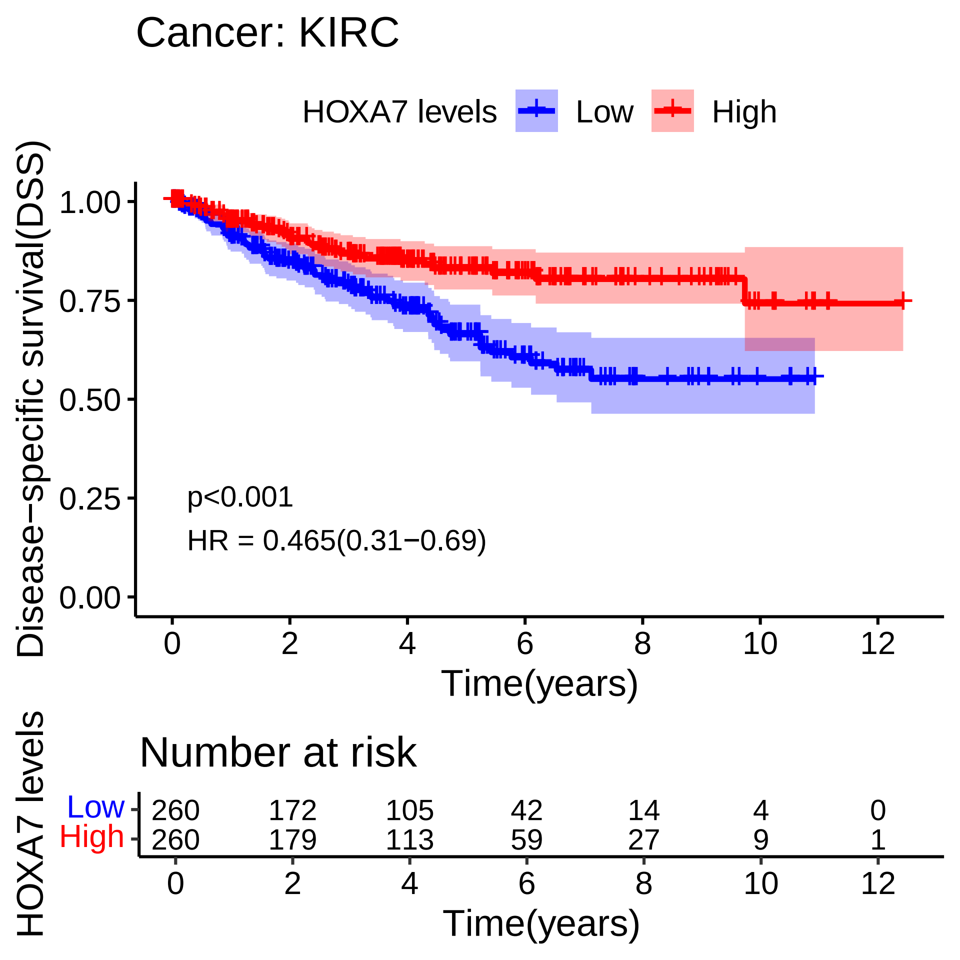
Cancer: KIRC
HOXA7 levels
Low
High
Disease−specific survival(DSS)
1.00
0.75
0.50
0.25
0.00
p<0.001
HR = 0.465(0.31−0.69)
0
2
4
6
8
10
12
Time(years)
Number at risk
HOXA7 levels
Low 260 172 105 42 14 4 0
High 260 179 113 59 27 9 1
Time(years)

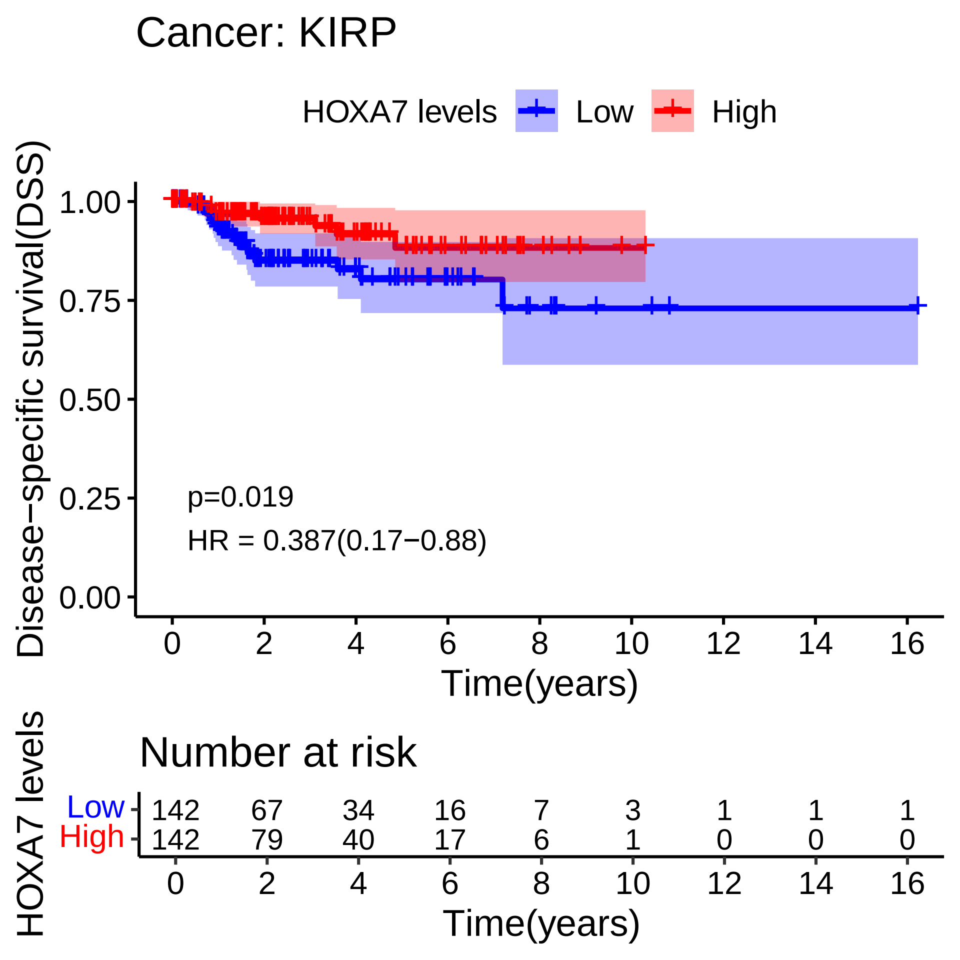

Cancer: KIRP
HOXA7 levels
Low
High
Disease−specific survival(DSS)
1.00
0.75
0.50
0.25
0.00
p=0.019
HR = 0.387(0.17−0.88)
0
2
4
6
8
10
12
14
16
Time(years)
Number at risk
HOXA7 levels
Low 142 67 34 16 7 3 1 1 1
High 142 79 40 17 6 1 0 0 0
0
2
4
6
8
10
12
14
16
Time(years)

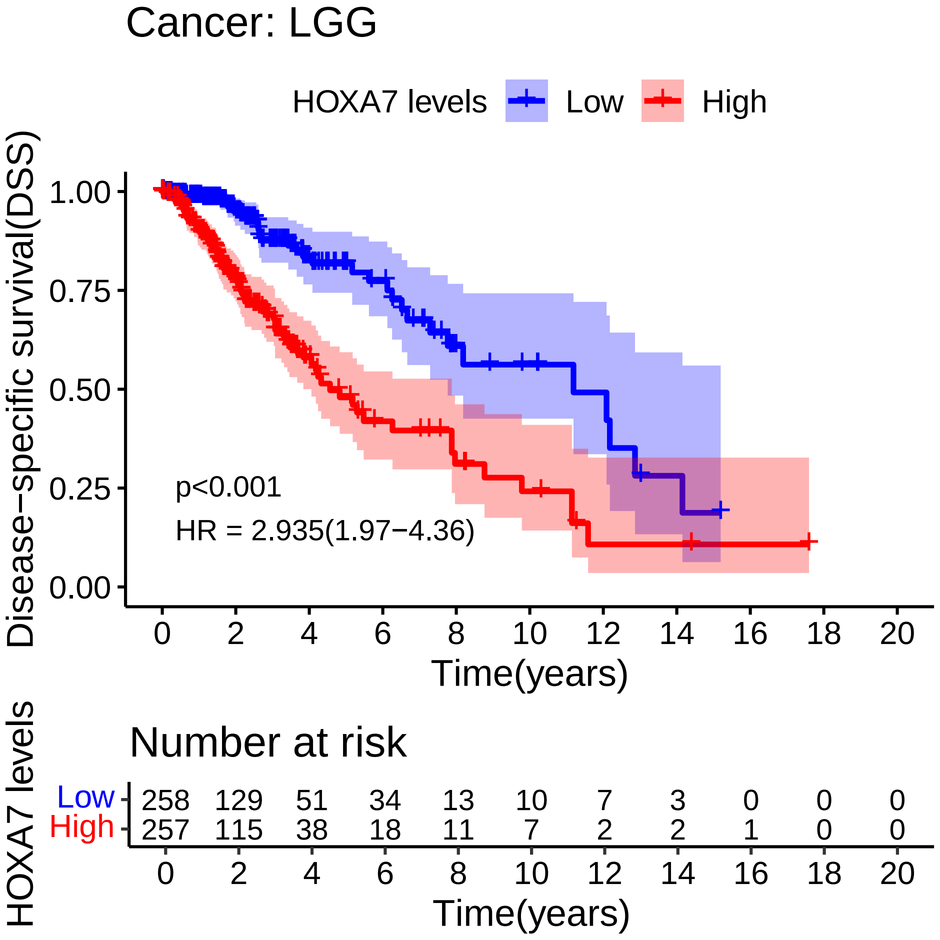
Cancer: LGG
HOXA7 levels
Low
High
Disease−specific survival(DSS)
1.00
0.75
0.50
0.25
0.00
p<0.001
HR = 2.935(1.97−4.36)
0 2 4 6 8 10 12 14 16 18 20
Time(years)
Number at risk
HOXA7 levels
Low 258 129 51 34 13 10 7 3 0 0 0
High 257 115 38 18 11 7 2 2 1 0 0
0 2 4 6 8 10 12 14 16 18 20
Time(years)

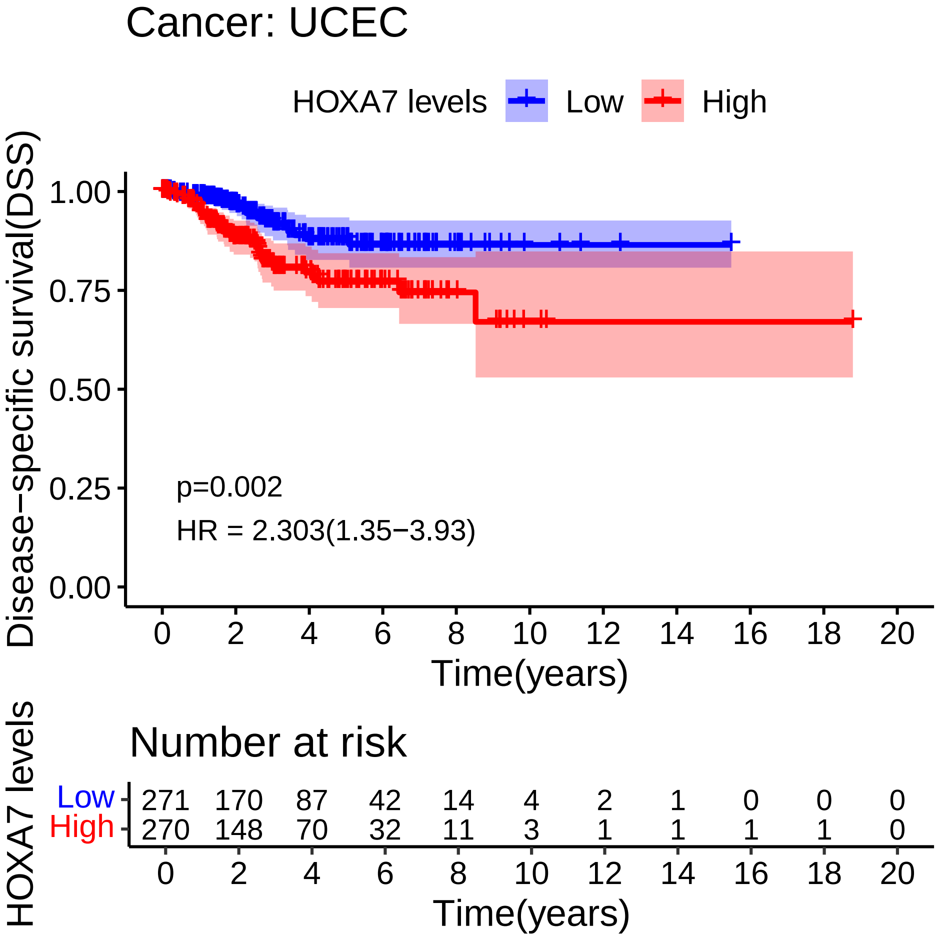

Cancer: UCEC
HOXA7 levels
Low
High
Disease−specific survival(DSS)
1.00
0.75
0.50
0.25
0.00
p=0.002
HR = 2.303(1.35−3.93)
0
2
4
6
8
10
12
14
16
18
20
Time(years)
Number at risk
HOXA7 levels
Low 271 170 87 42 14 4 2 1 0 0 0
High 270 148 70 32 11 3 1 1 1 1 0
Time(years)

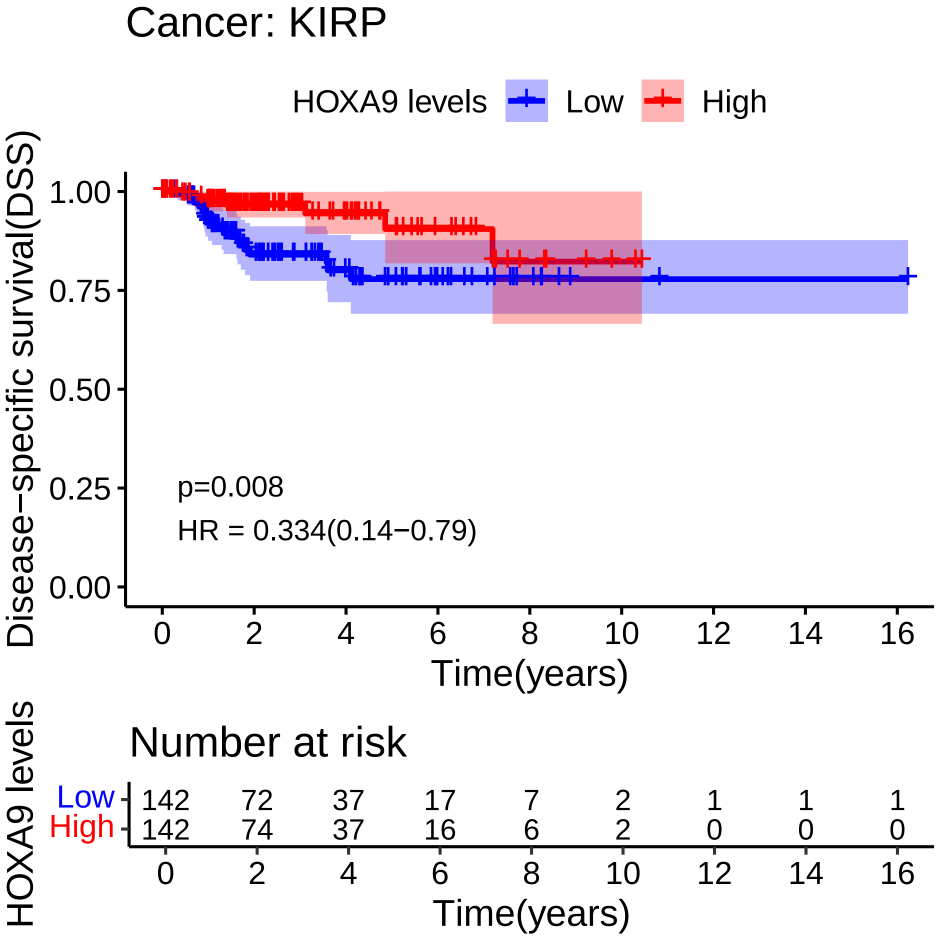

Cancer: KIRP
HOXA9 levels
Low
High
Disease−specific survival(DSS)
1.00
0.75
0.50
0.25
0.00
p=0.008
HR = 0.334(0.14−0.79)
0
2
4
6
8
10
12
14
16
Time(years)
Number at risk
HOXA9 levels
Low 142 72 37 17 7 2 1 1 1
High 142 74 37 16 6 2 0 0 0
Time(years)

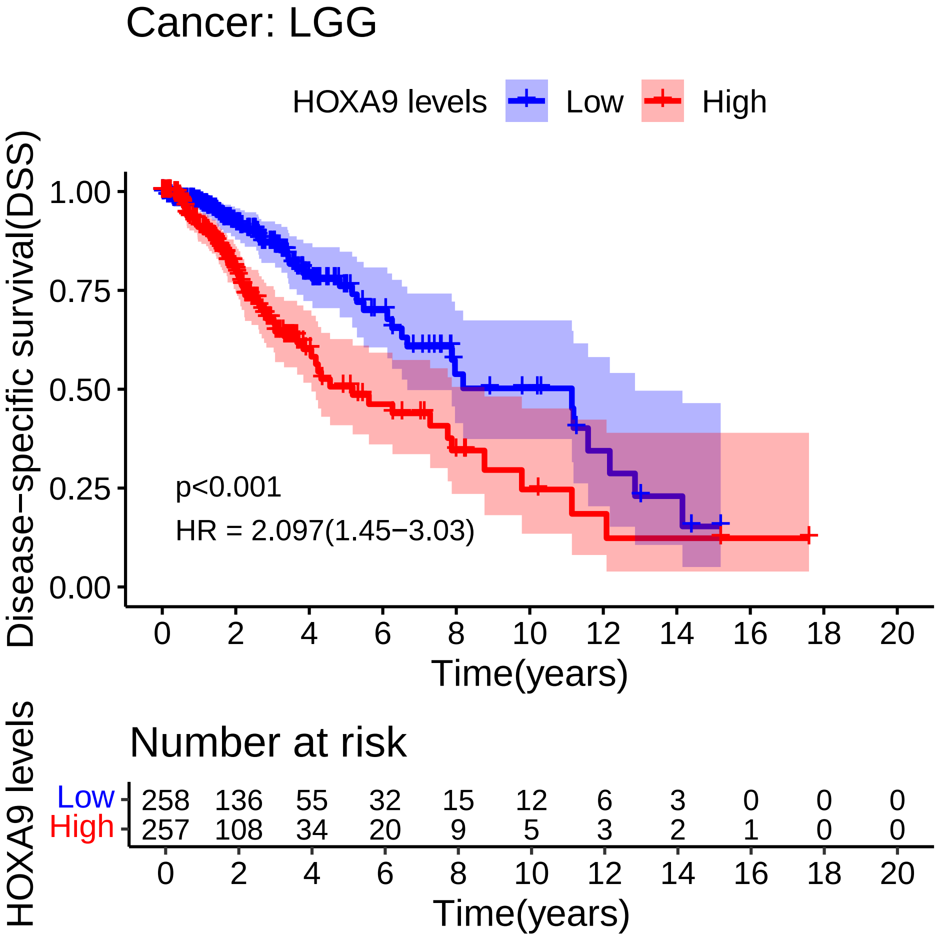
Cancer: LGG
HOXA9 levels
Low
High
Disease−specific survival(DSS)
1.00
0.75
0.50
0.25
0.00
p<0.001
HR = 2.097(1.45−3.03)
0
2
4
6
8
10
12
14
16
18
20
Time(years)
Number at risk
HOXA9 levels
Low
High
258 136 55 32 15 12 6 3 0 0 0
257 108 34 20 9 5 3 2 1 0 0
0
2
4
6
8
10
12
14
16
18
20
Time(years)

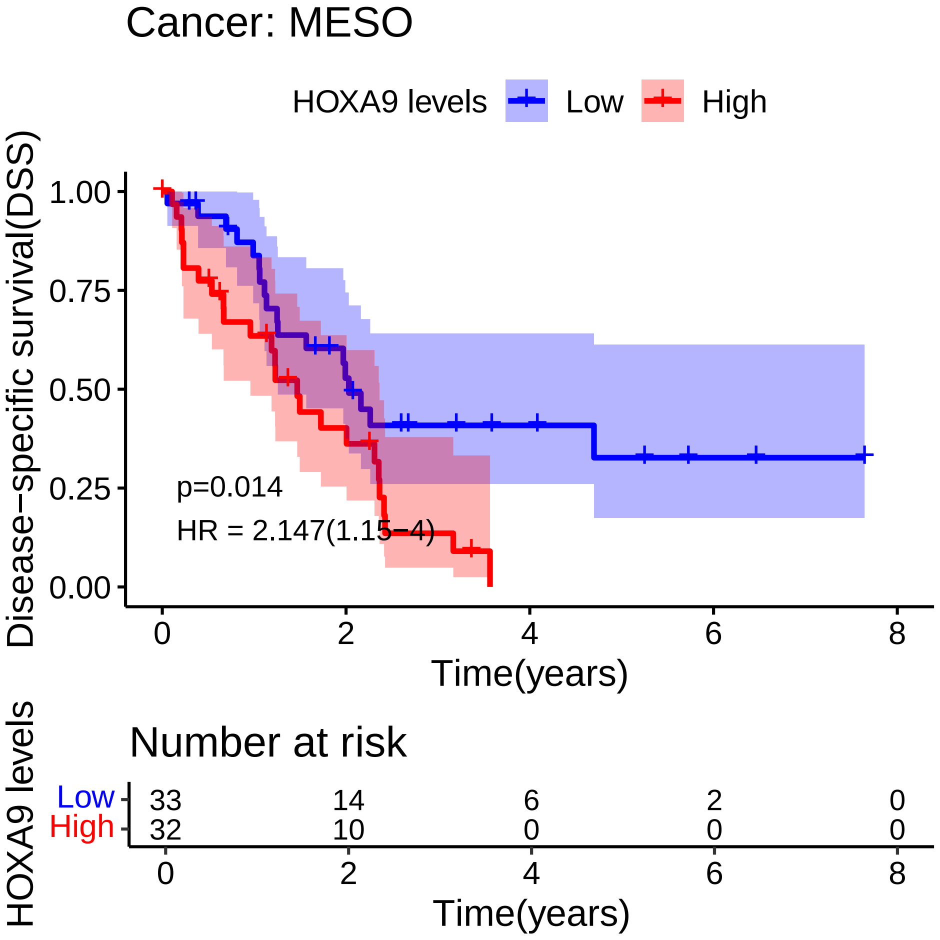

Cancer: MESO
HOXA9 levels
Low
High
Disease−specific survival(DSS)
1.00
0.75
0.50
0.25
0.00
p=0.014
HR = 2.147(1.15−4)
0
2
4
6
8
Time(years)
Number at risk
HOXA9 levels
Low 33 14 6 2 0
High 32 10 0 0 0
0
2
4
6
8
Time(years)

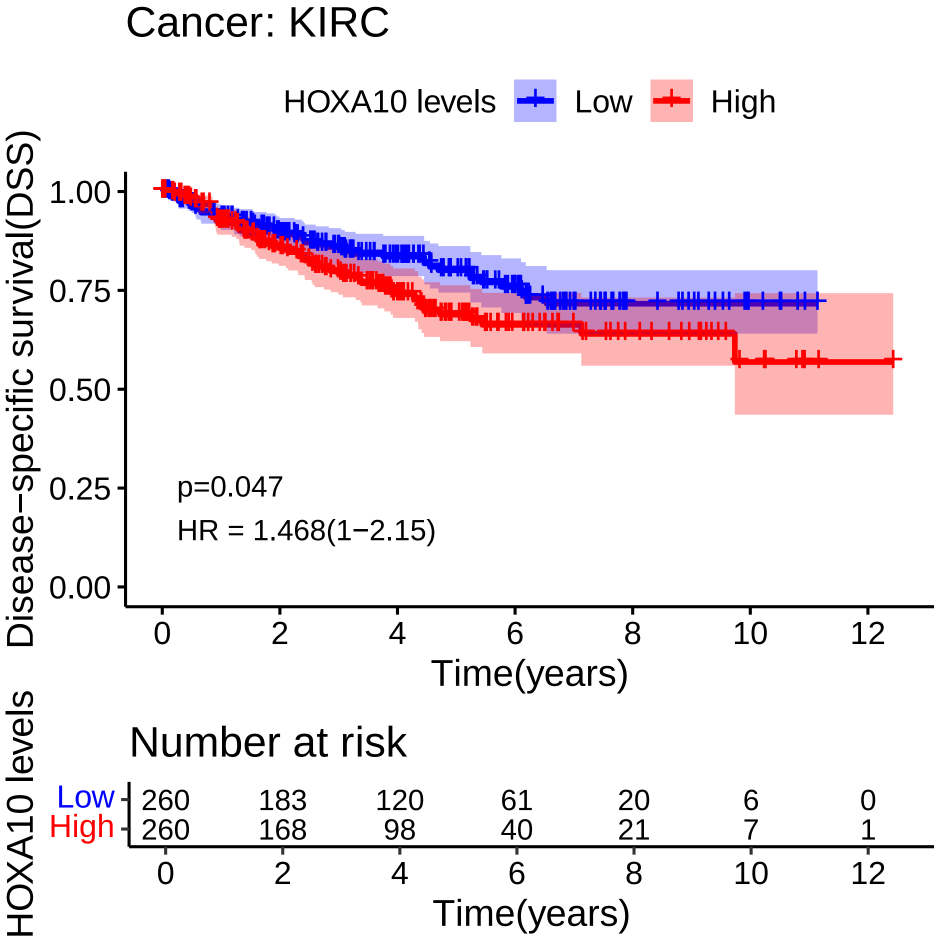

Cancer: KIRC
HOXA10 levels
Low
High
Disease−specific survival(DSS)
1.00
0.75
0.50
0.25
0.00
p=0.047
HR = 1.468(1−2.15)
0
2
4
6
8
10
12
Time(years)
Number at risk
HOXA10 levels
Low 260 183 120 61 20 6 0
High 260 168 98 40 21 7 1
0
2
4
6
8
10
12
Time(years)

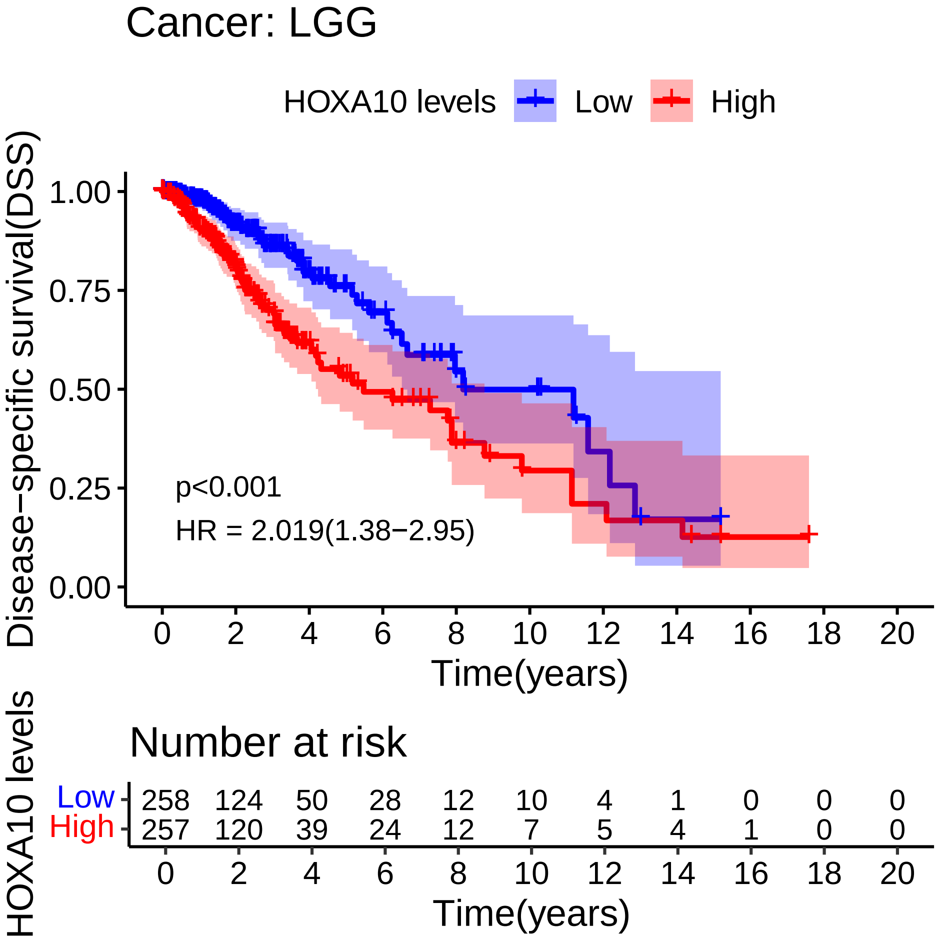
Cancer: LGG
HOXA10 levels
Low
High
Disease−specific survival(DSS)
1.00
0.75
0.50
0.25
0.00
p<0.001
HR = 2.019(1.38−2.95)
0 2 4 6 8 10 12 14 16 18 20
Time(years)
Number at risk
HOXA10 levels
Low 258 124 50 28 12 10 4 1 0 0 0
High 257 120 39 24 12 7 5 4 1 0 0
0 2 4 6 8 10 12 14 16 18 20
Time(years)

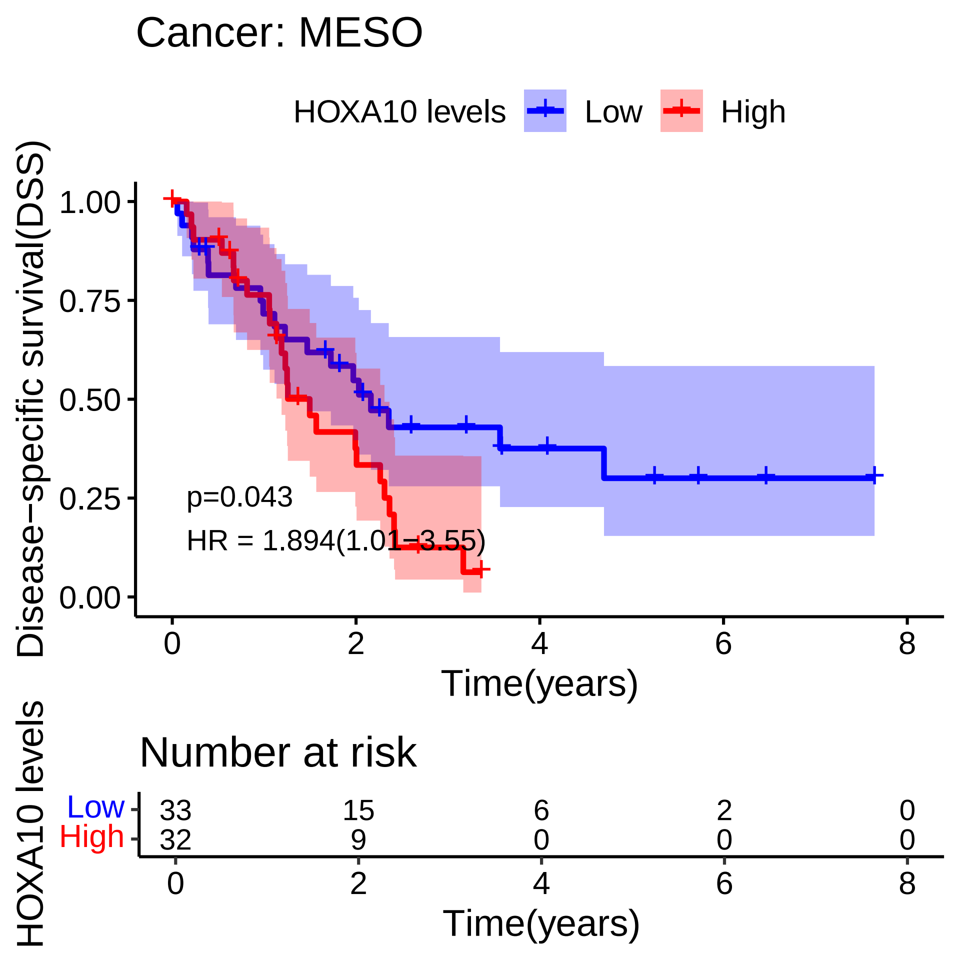

Cancer: MESO
HOXA10 levels
Low
High
Disease−specific survival(DSS)
1.00
0.75
0.50
0.25
0.00
p=0.043
HR = 1.894(1.01−3.55)
0
2
4
6
8
Time(years)
Number at risk
HOXA10 levels
Low 33 15 6 2 0
High 32 9 0 0 0
0
2
4
6
8
Time(years)

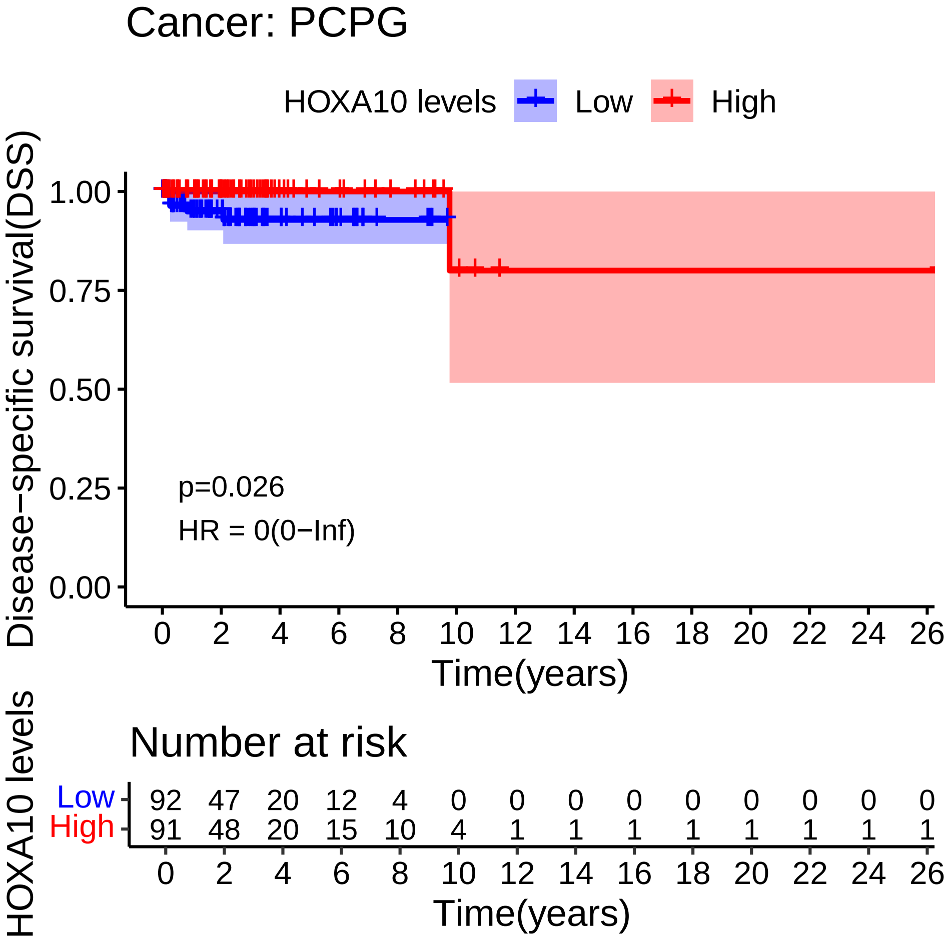

Cancer: PCPG
HOXA10 levels
Low
High
Disease−specific survival(DSS)
1.00
0.75
0.50
0.25
0.00
p=0.026
HR = 0(0−Inf)
0 2 4 6 8 10 12 14 16 18 20 22 24 26
Time(years)
Number at risk
HOXA10 levels
Low 92 47 20 12 4 0 0 0 0 0 0 0 0 0
High 91 48 20 15 10 4 1 1 1 1 1 1 1 1
0 2 4 6 8 10 12 14 16 18 20 22 24 26
Time(years)

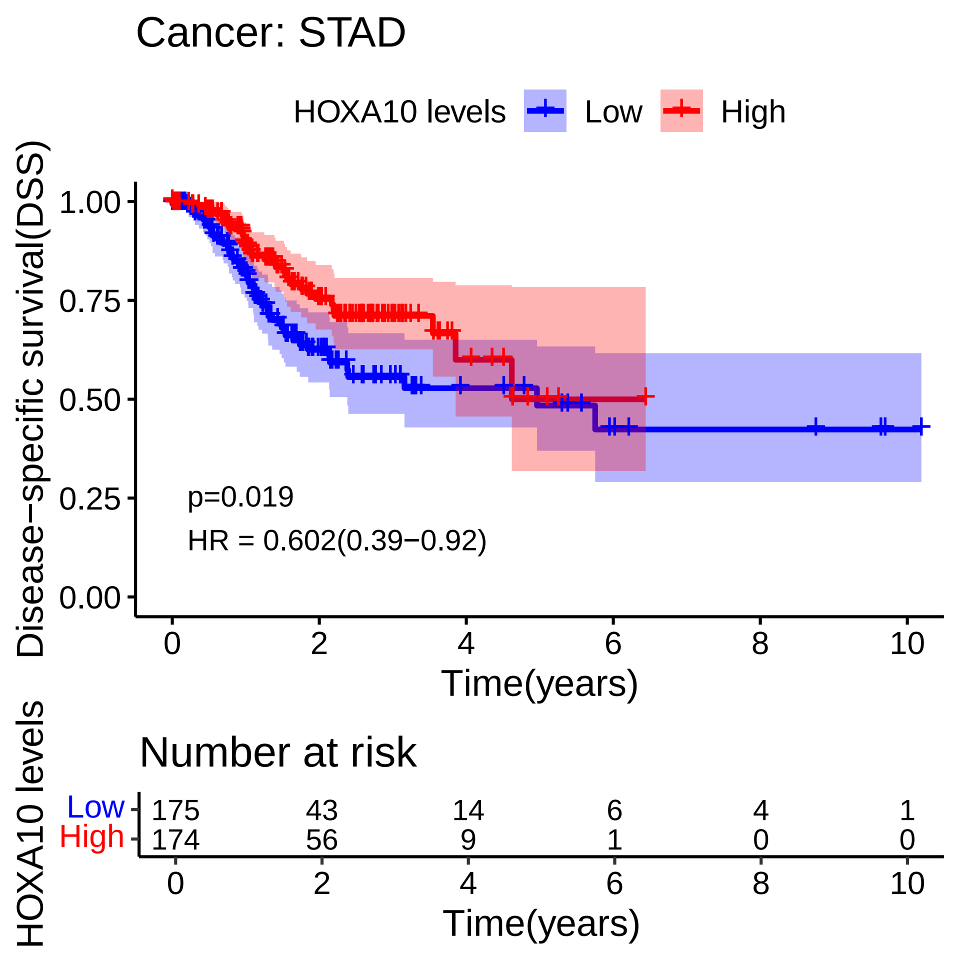

Cancer: STAD
HOXA10 levels
Low
High
Disease−specific survival(DSS)
1.00
0.75
0.50
0.25
0.00
p=0.019
HR = 0.602(0.39−0.92)
0
2
4
6
8
10
Time(years)
Number at risk
HOXA10 levels
Low 175 43 14 6 4 1
High 174 56 9 1 0 0
0
2
4
6
8
10
Time(years)

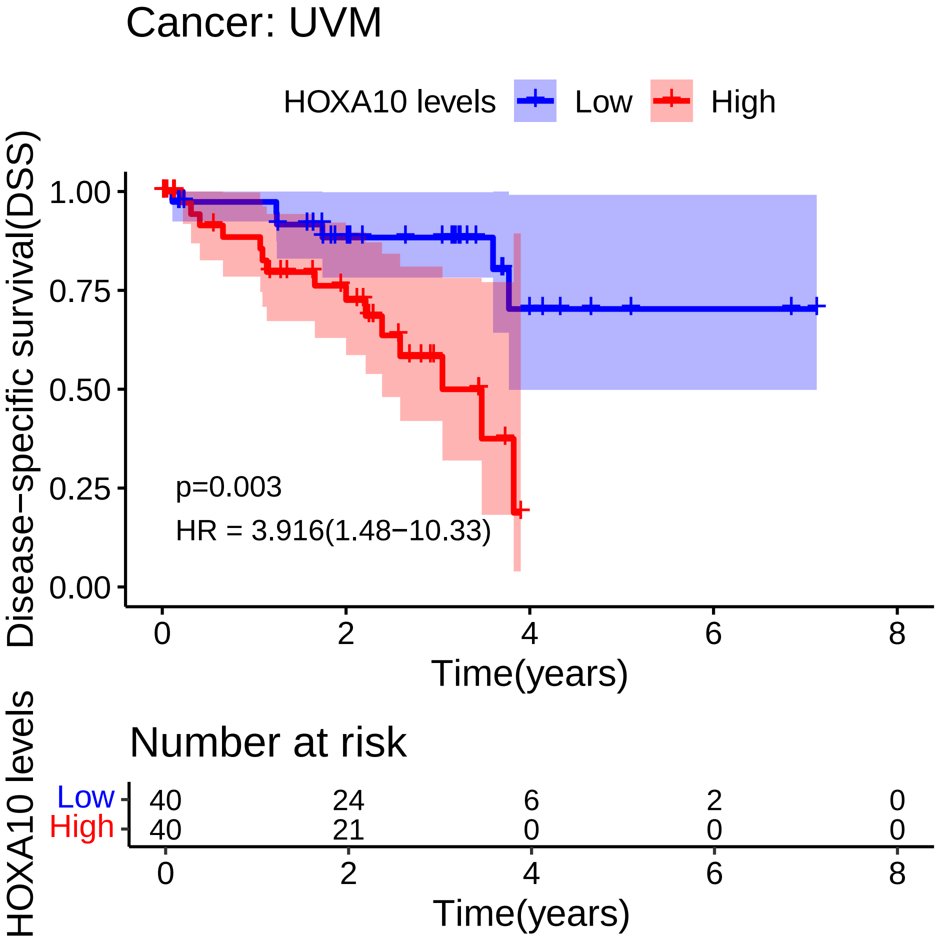

Cancer: UVM
HOXA10 levels
Low
High
Disease−specific survival(DSS)
1.00
0.75
0.50
0.25
0.00
p=0.003
HR = 3.916(1.48−10.33)
0
2
4
6
8
Time(years)
Number at risk
HOXA10 levels
Low 40 24 6 2 0
High 40 21 0 0 0
Time(years)

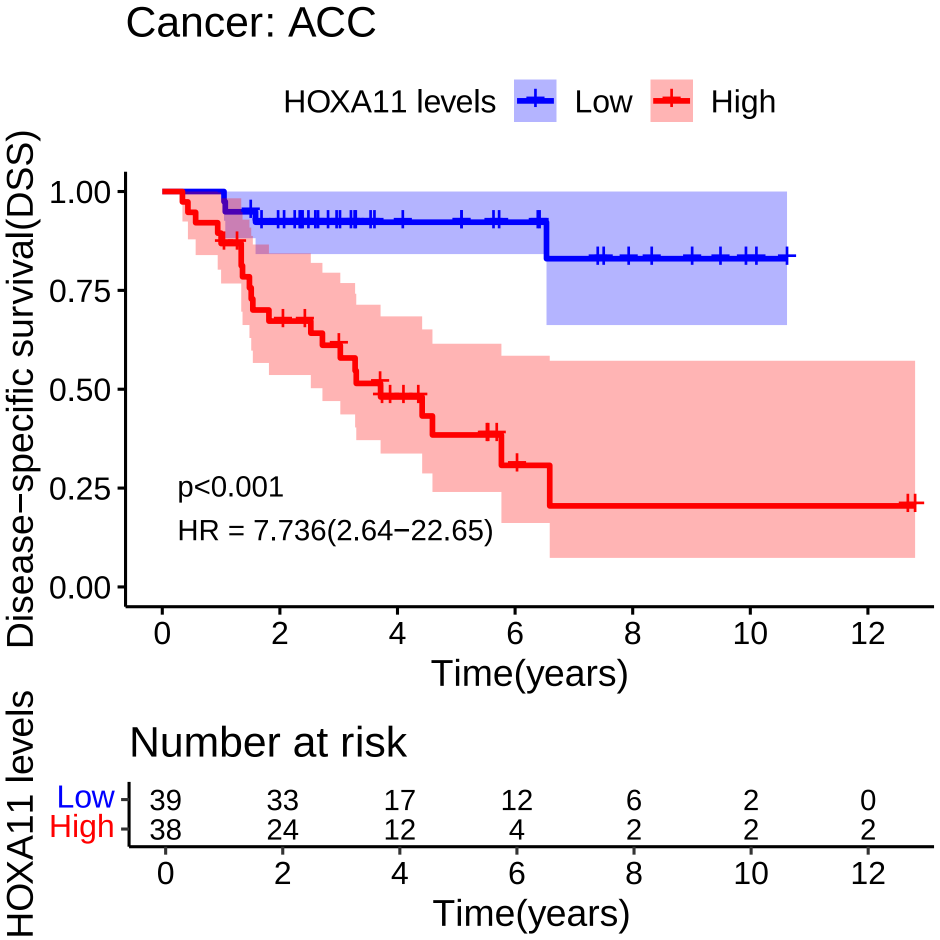

Cancer: ACC
HOXA11 levels
Low
High
Disease−specific survival(DSS)
1.00
0.75
0.50
0.25
0.00
p<0.001
HR = 7.736(2.64−22.65)
0
2
4
6
8
10
12
Time(years)
Number at risk
HOXA11 levels
Low 39 33 17 12 6 2 0
High 38 24 12 4 2 2 2
Time(years)

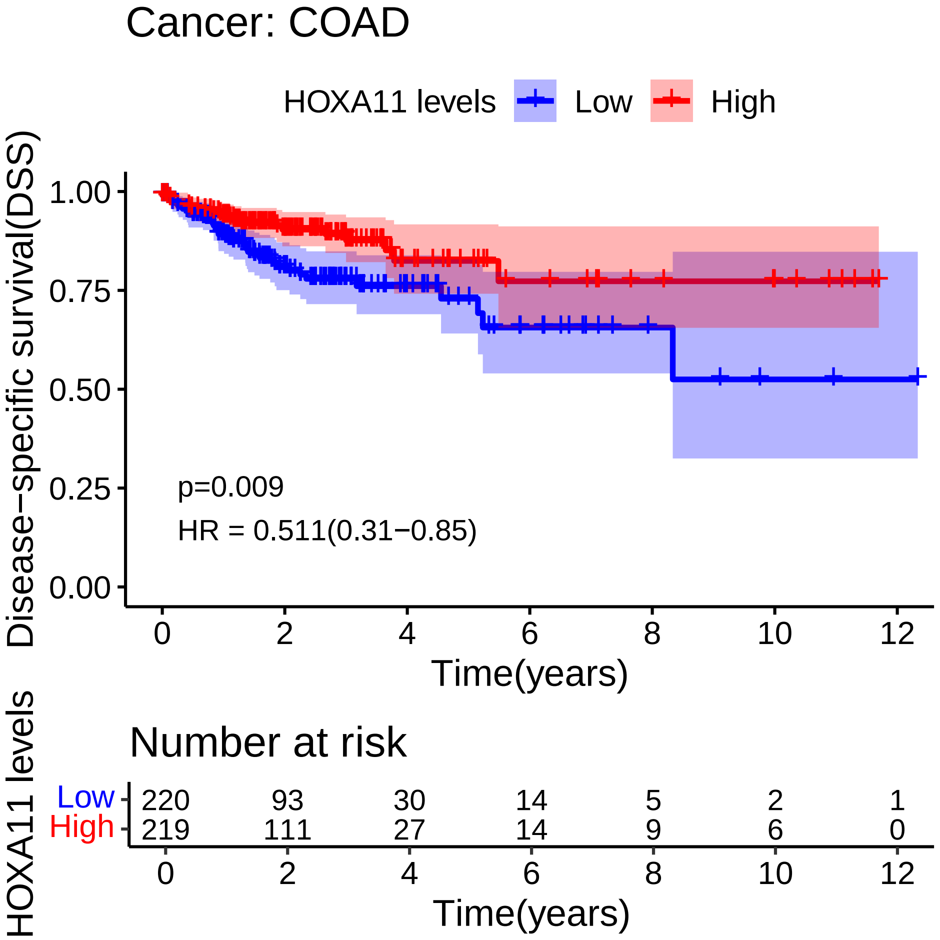

Cancer: COAD
HOXA11 levels
Low
High
Disease−specific survival(DSS)
1.00
0.75
0.50
0.25
0.00
p=0.009
HR = 0.511(0.31−0.85)
0
2
4
6
8
10
12
Time(years)
Number at risk
HOXA11 levels
Low
High
220 93 30 14 5 2 1
219 111 27 14 9 6 0
Time(years)

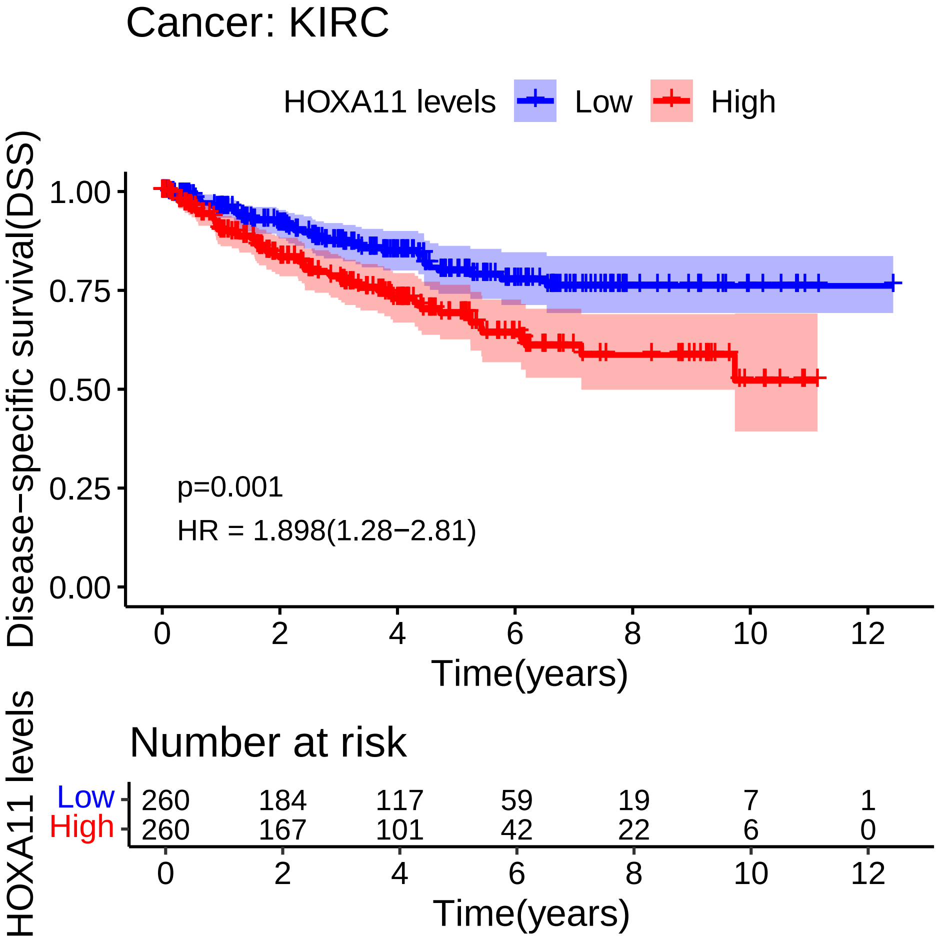

Cancer: KIRC
HOXA11 levels
Low
High
Disease−specific survival(DSS)
1.00
0.75
0.50
0.25
0.00
p=0.001
HR = 1.898(1.28−2.81)
0
2
4
6
8
10
12
Time(years)
Number at risk
HOXA11 levels
Low 260 184 117 59 19 7 1
High 260 167 101 42 22 6 0
0
2
4
6
8
10
12
Time(years)

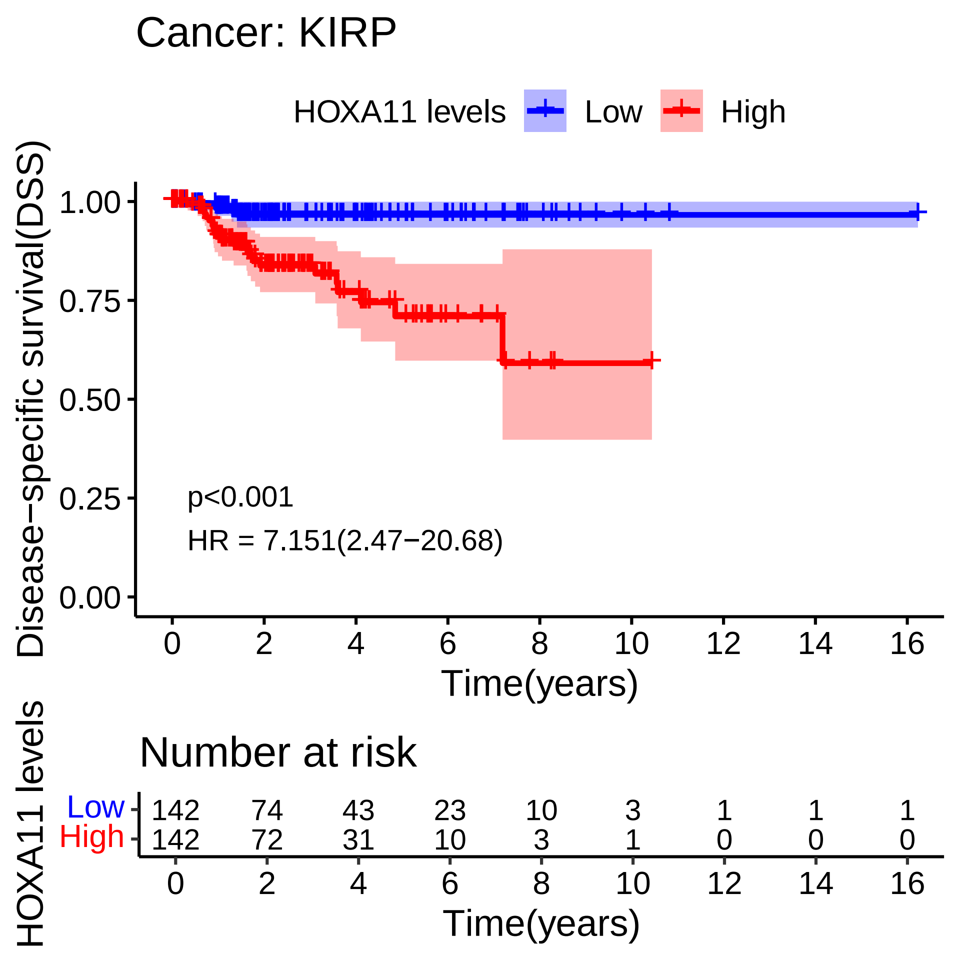

Cancer: KIRP
HOXA11 levels
Low
High
Disease−specific survival(DSS)
1.00
0.75
0.50
0.25
0.00
p<0.001
HR = 7.151(2.47−20.68)
0
2
4
6
8
10
12
14
16
Time(years)
Number at risk
HOXA11 levels
Low 142 74 43 23 10 3 1 1 1
High 142 72 31 10 3 1 0 0 0
0
2
4
6
8
10
12
14
16
Time(years)

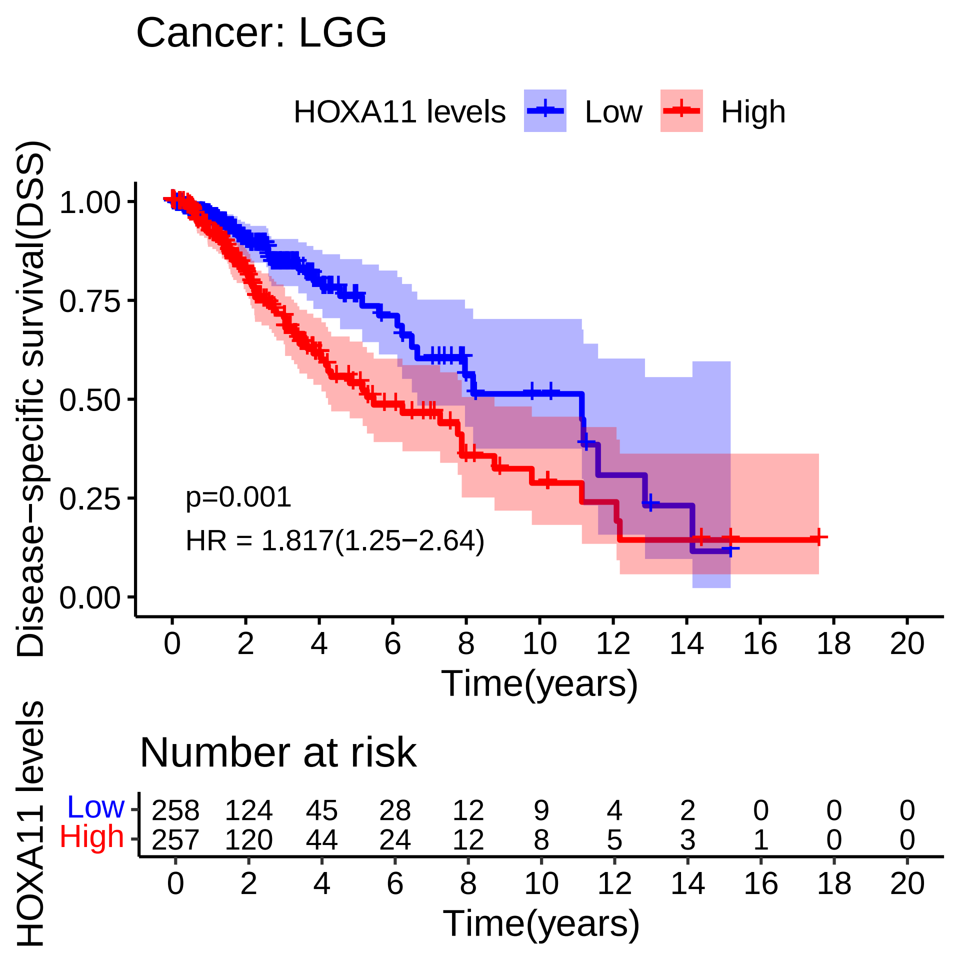

Cancer: LGG
HOXA11 levels
Low
High
Disease−specific survival(DSS)
1.00
0.75
0.50
0.25
0.00
p=0.001
HR = 1.817(1.25−2.64)
0 2 4 6 8 10 12 14 16 18 20
Time(years)
Number at risk
HOXA11 levels
Low 258 124 45 28 12 9 4 2 0 0 0
High 257 120 44 24 12 8 5 3 1 0 0
0 2 4 6 8 10 12 14 16 18 20
Time(years)

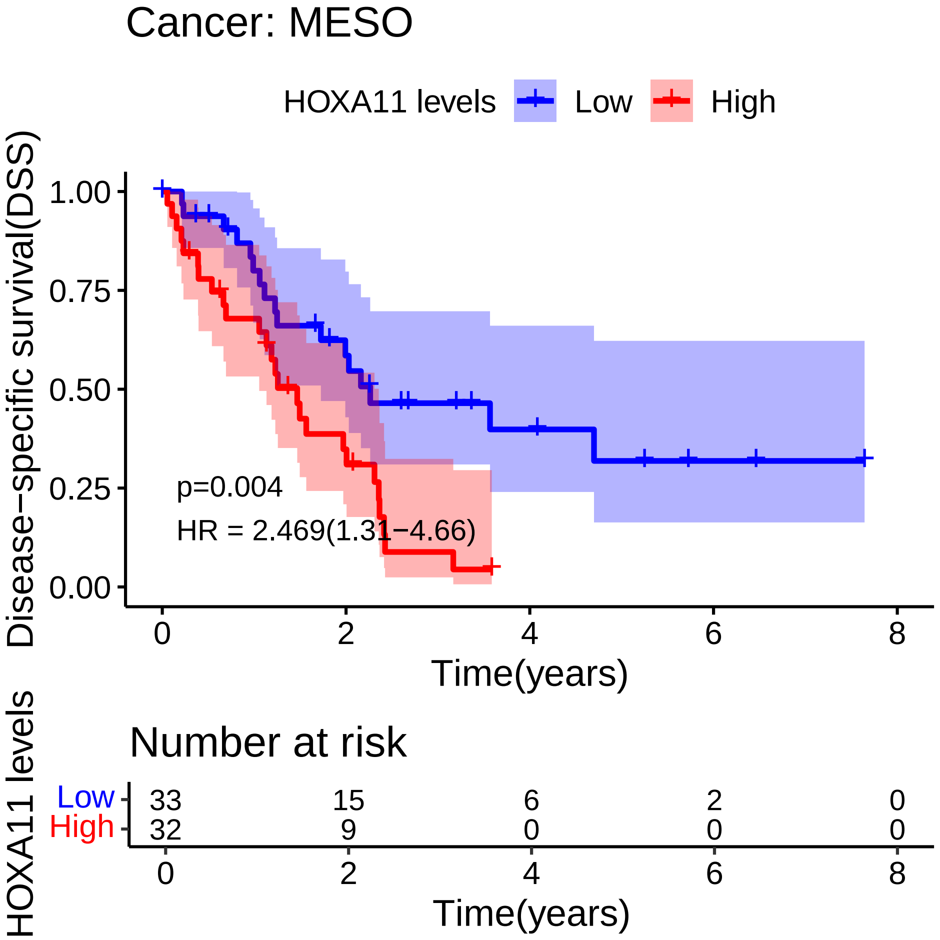

Cancer: MESO
HOXA11 levels
Low
High
Disease−specific survival(DSS)
1.00
0.75
0.50
0.25
0.00
p=0.004
HR = 2.469(1.31−4.66)
0
2
4
6
8
Time(years)
Number at risk
HOXA11 levels
Low
High
33
15
6
2
0
32
9
0
0
0
0
2
4
6
8
Time(years)

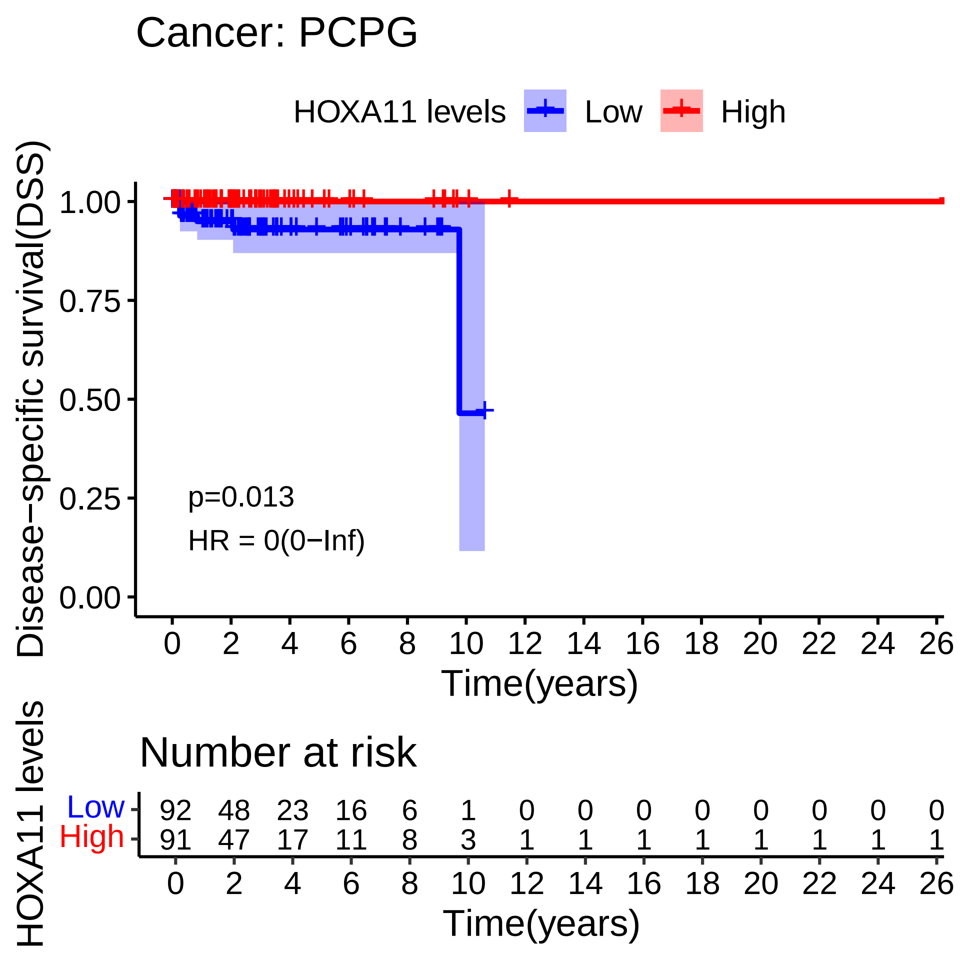

Cancer: PCPG
HOXA11 levels
Low
High
Disease−specific survival(DSS)
1.00
0.75
0.50
0.25
0.00
p=0.013
HR = 0(0−Inf)
0 2 4 6 8 10 12 14 16 18 20 22 24 26
Time(years)
Number at risk
HOXA11 levels
Low 92 48 23 16 6 1 0 0 0 0 0 0 0 0
High 91 47 17 11 8 3 1 1 1 1 1 1 1 1
0 2 4 6 8 10 12 14 16 18 20 22 24 26
Time(years)

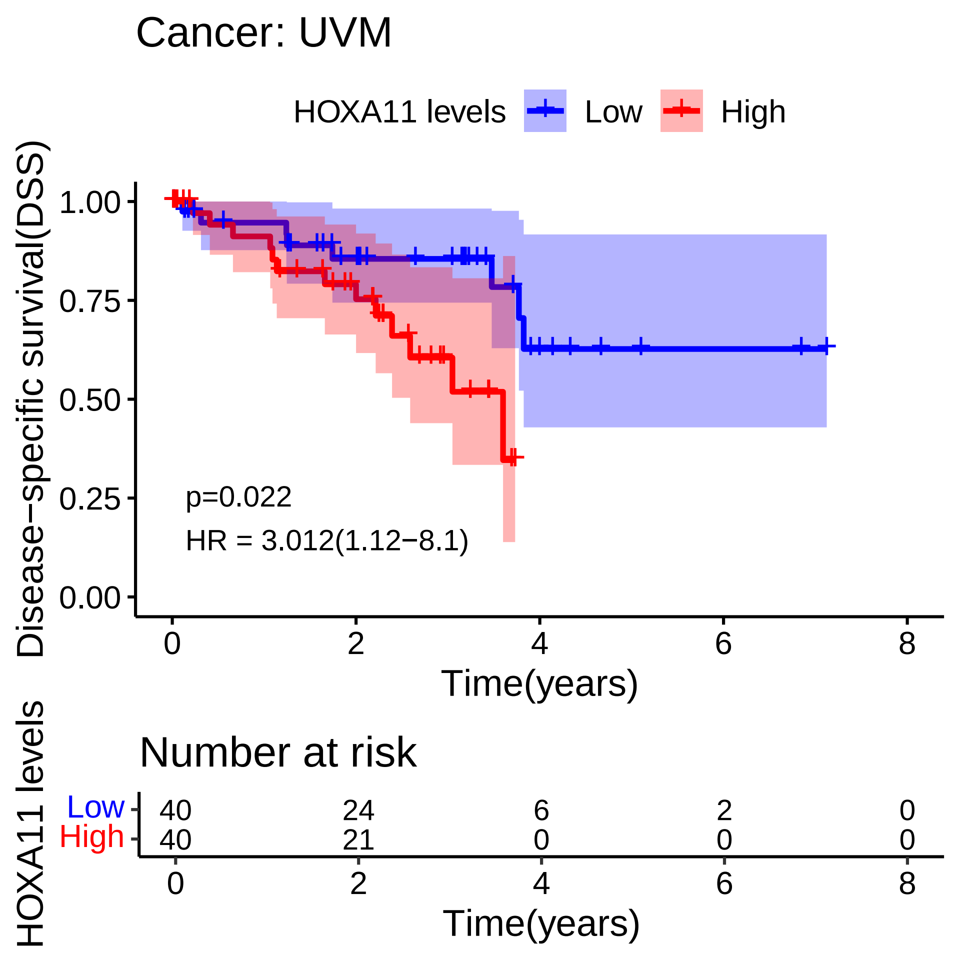
Cancer: UVM
HOXA11 levels
Low
High
Disease−specific survival(DSS)
1.00
0.75
0.50
0.25
0.00
p=0.022
HR = 3.012(1.12−8.1)
0
2
4
6
8
Time(years)
Number at risk
HOXA11 levels
Low 40 24 6 2 0
High 40 21 0 0 0
0
2
4
6
8
Time(years)

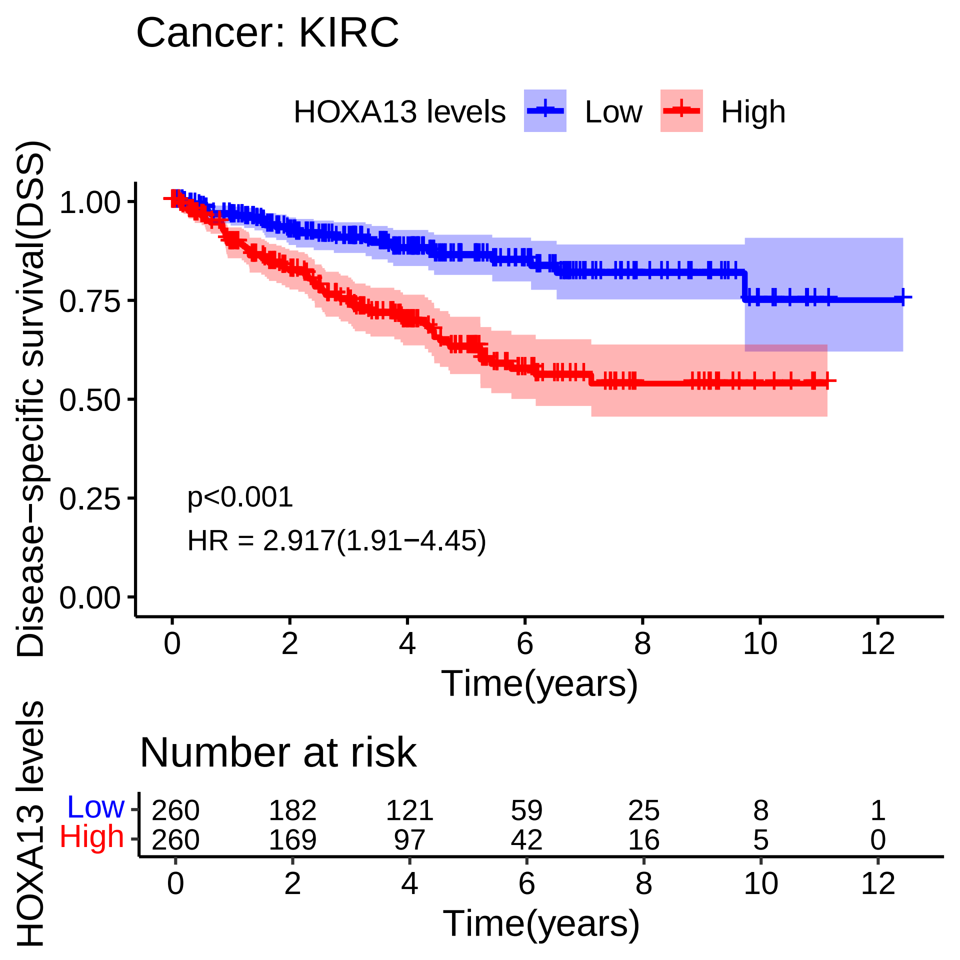
Cancer: KIRC
HOXA13 levels
Low
High
Disease−specific survival(DSS)
1.00
0.75
0.50
0.25
0.00
p<0.001
HR = 2.917(1.91−4.45)
0
2
4
6
8
10
12
Time(years)
Number at risk
HOXA13 levels
Low 260 182 121 59 25 8 1
High 260 169 97 42 16 5 0
Time(years)

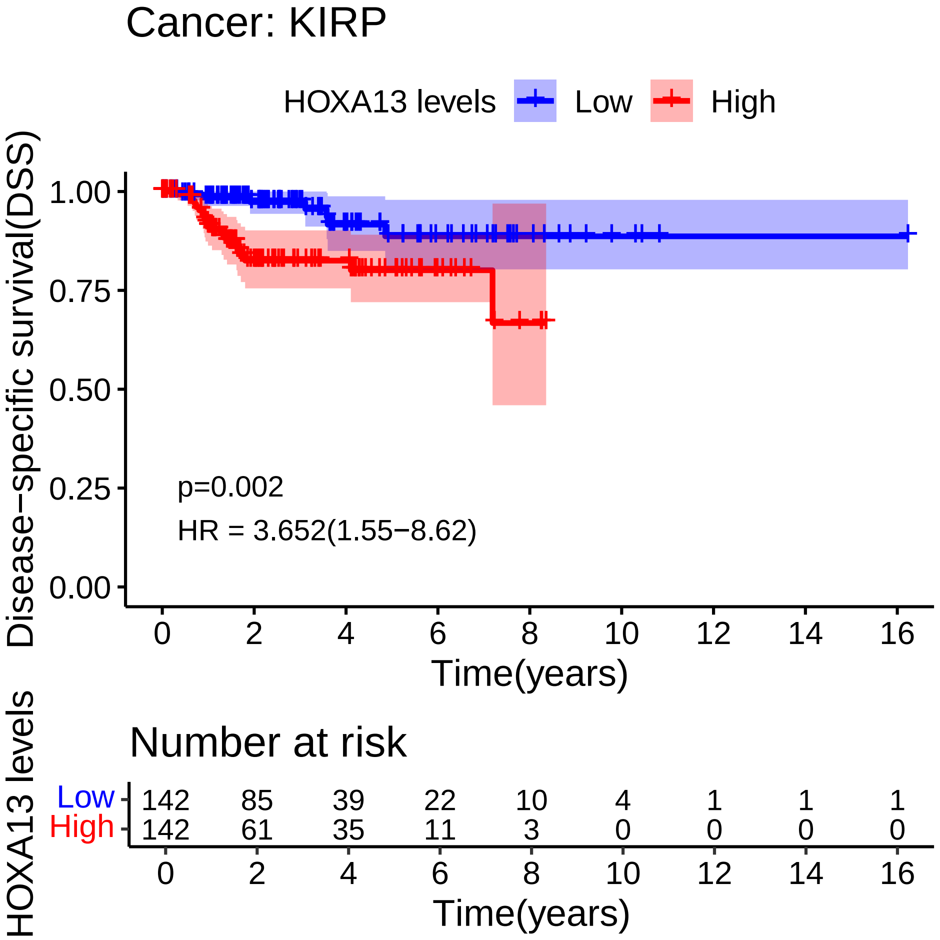
Cancer: KIRP
HOXA13 levels
Low
High
Disease−specific survival(DSS)
1.00
0.75
0.50
0.25
0.00
p=0.002
HR = 3.652(1.55−8.62)
0
2
4
6
8
10
12
14
16
Time(years)
Number at risk
HOXA13 levels
Low 142 85 39 22 10 4 1 1 1
High 142 61 35 11 3 0 0 0 0
0
2
4
6
8
10
12
14
16
Time(years)

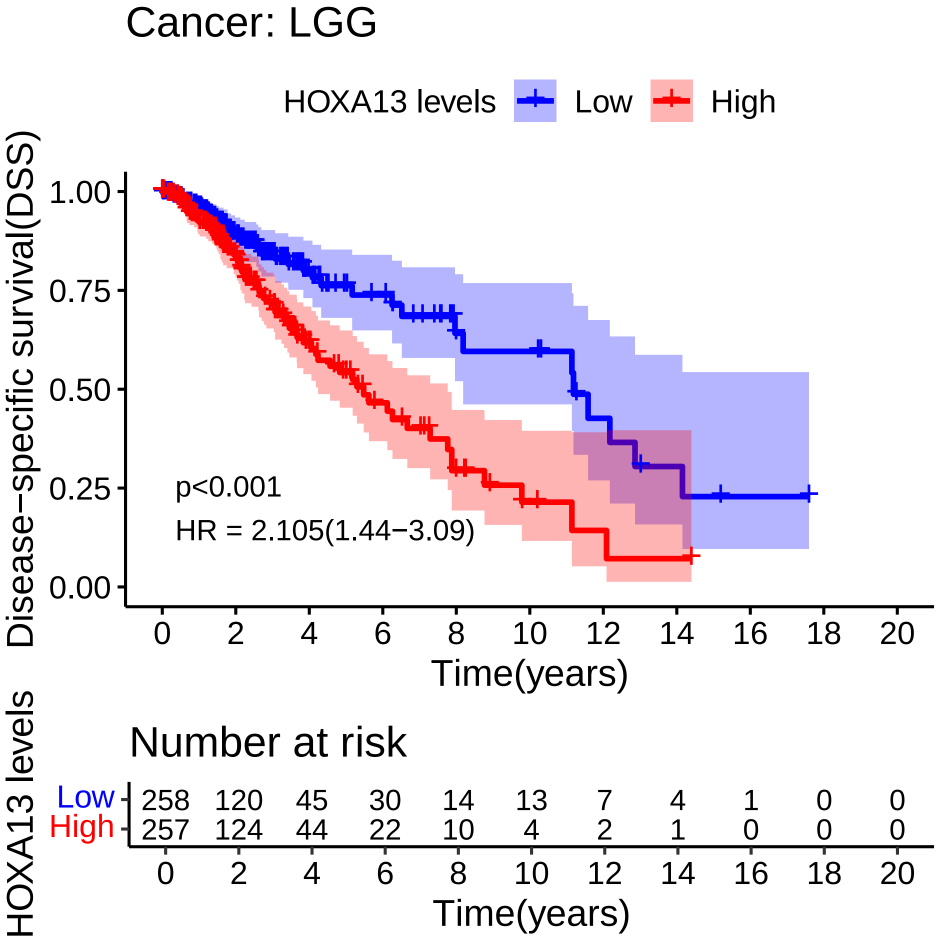
Cancer: LGG
HOXA13 levels
Low
High
Disease−specific survival(DSS)
1.00
0.75
0.50
0.25
0.00
p<0.001
HR = 2.105(1.44−3.09)
0
2
4
6
8
10
12
14
16
18
20
Time(years)
Number at risk
HOXA13 levels
Low
High
258 120 45 30 14 13 7 4 1 0 0
257 124 44 22 10 4 2 1 0 0 0
0
2
4
6
8
10
12
14
16
18
20
Time(years)

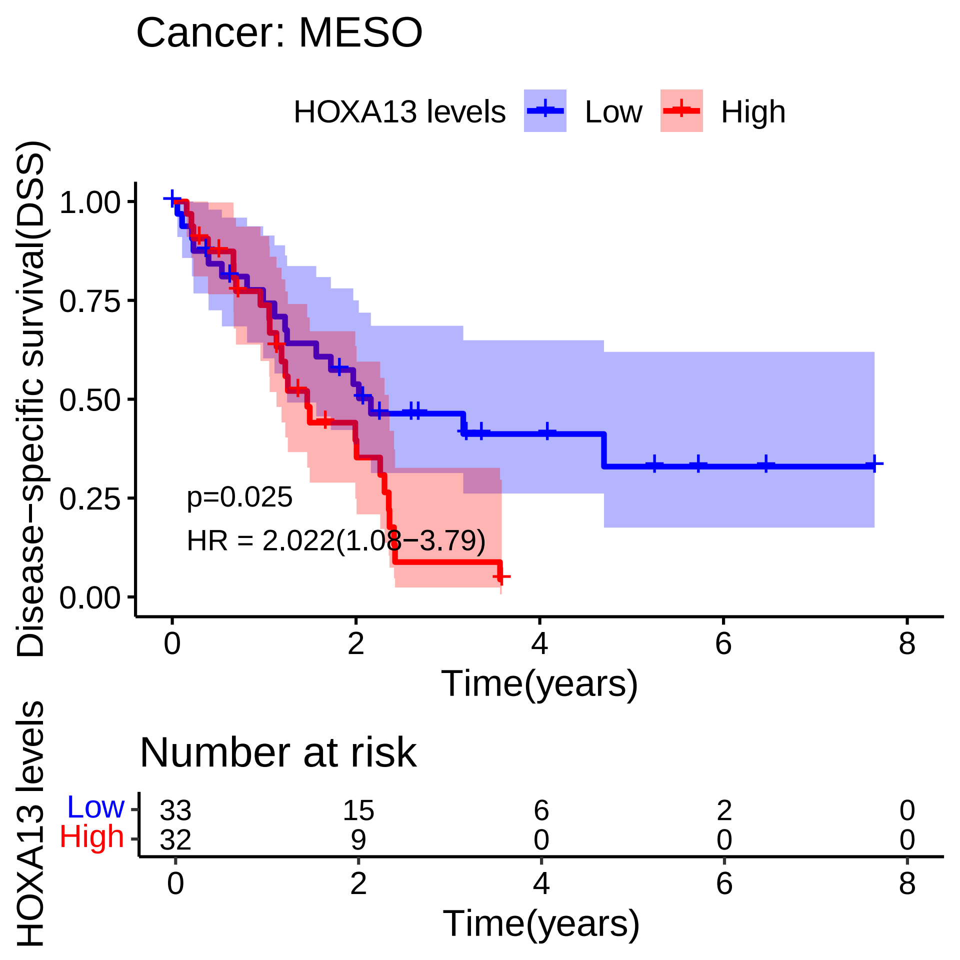

Cancer: MESO
HOXA13 levels
Low
High
Disease−specific survival(DSS)
1.00
0.75
0.50
0.25
0.00
p=0.025
HR = 2.022(1.08−3.79)
0
2
4
6
8
Time(years)
Number at risk
HOXA13 levels
Low
High
33
15
6
2
0
32
9
0
0
0
0
2
4
6
8
Time(years)

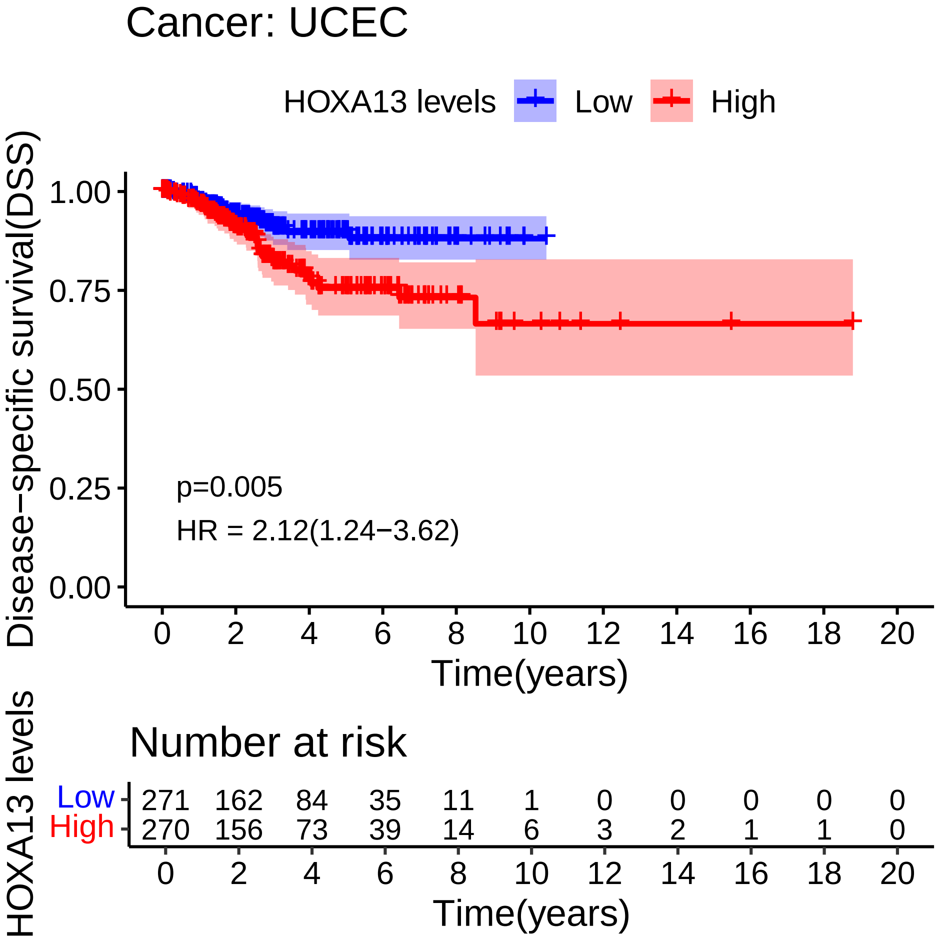

Cancer: UCEC
HOXA13 levels
Low
High
Disease−specific survival(DSS)
1.00
0.75
0.50
0.25
0.00
p=0.005
HR = 2.12(1.24−3.62)
0
2
4
6
8
10
12
14
16
18
20
Time(years)
Number at risk
HOXA13 levels
Low 271 162 84 35 11 1 0 0 0 0 0
High 270 156 73 39 14 6 3 2 1 1 0
0
2
4
6
8
10
12
14
16
18
20
Time(years)

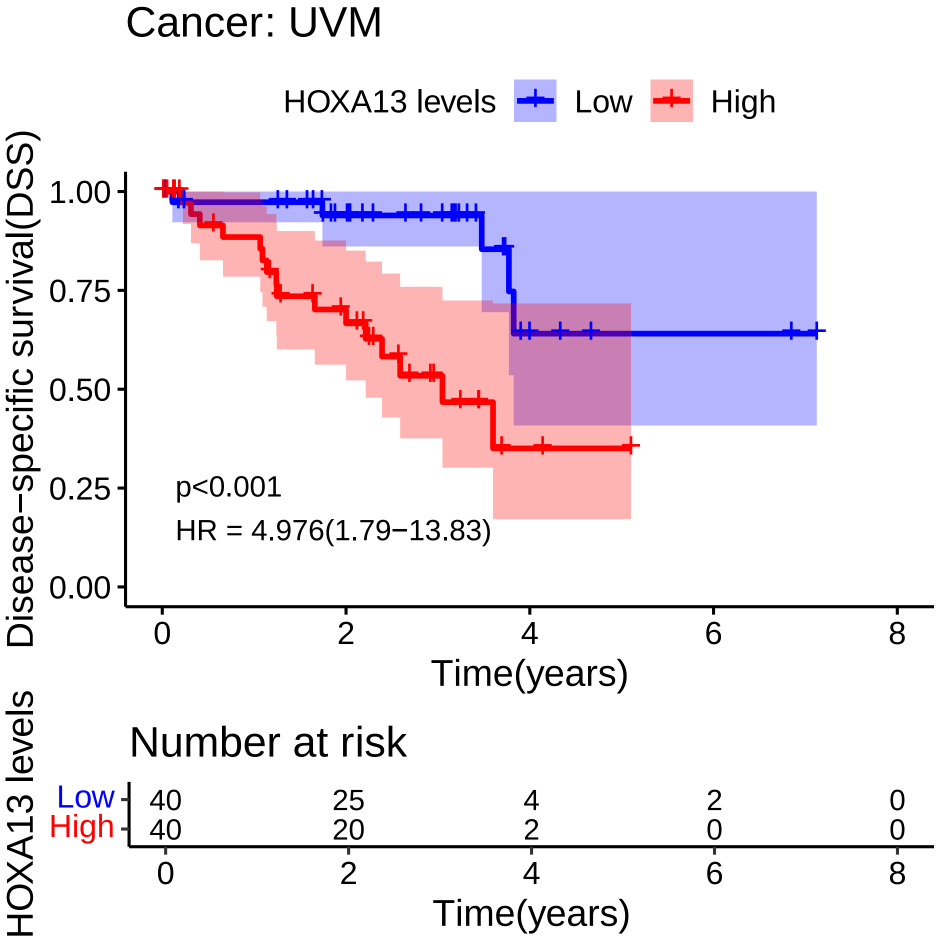

Cancer: UVM
HOXA13 levels
Low
High
Disease−specific survival(DSS)
1.00
0.75
0.50
0.25
0.00
p<0.001
HR = 4.976(1.79−13.83)
0
2
4
6
8
Time(years)
Number at risk
HOXA13 levels
Low
High
40
25
4
2
0
40
20
2
0
0
0
2
4
6
8
Time(years)

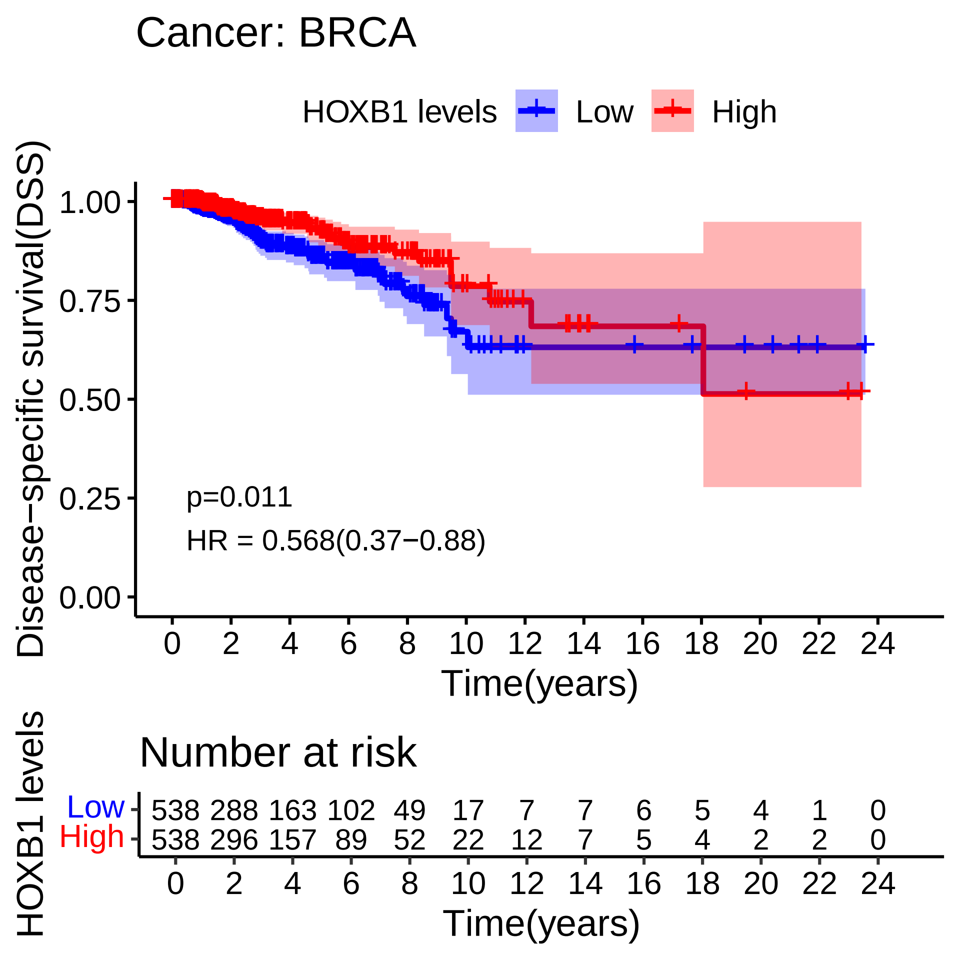

Cancer: BRCA
HOXB1 levels
Low
High
Disease−specific survival(DSS)
1.00
0.75
0.50
0.25
0.00
p=0.011
HR = 0.568(0.37−0.88)
0 2 4 6 8 10 12 14 16 18 20 22 24
Time(years)
Number at risk
HOXB1 levels
Low 538 288 163 102 49 17 7 7 6 5 4 1 0
High 538 296 157 89 52 22 12 7 5 4 2 2 0
0 2 4 6 8 10 12 14 16 18 20 22 24
Time(years)

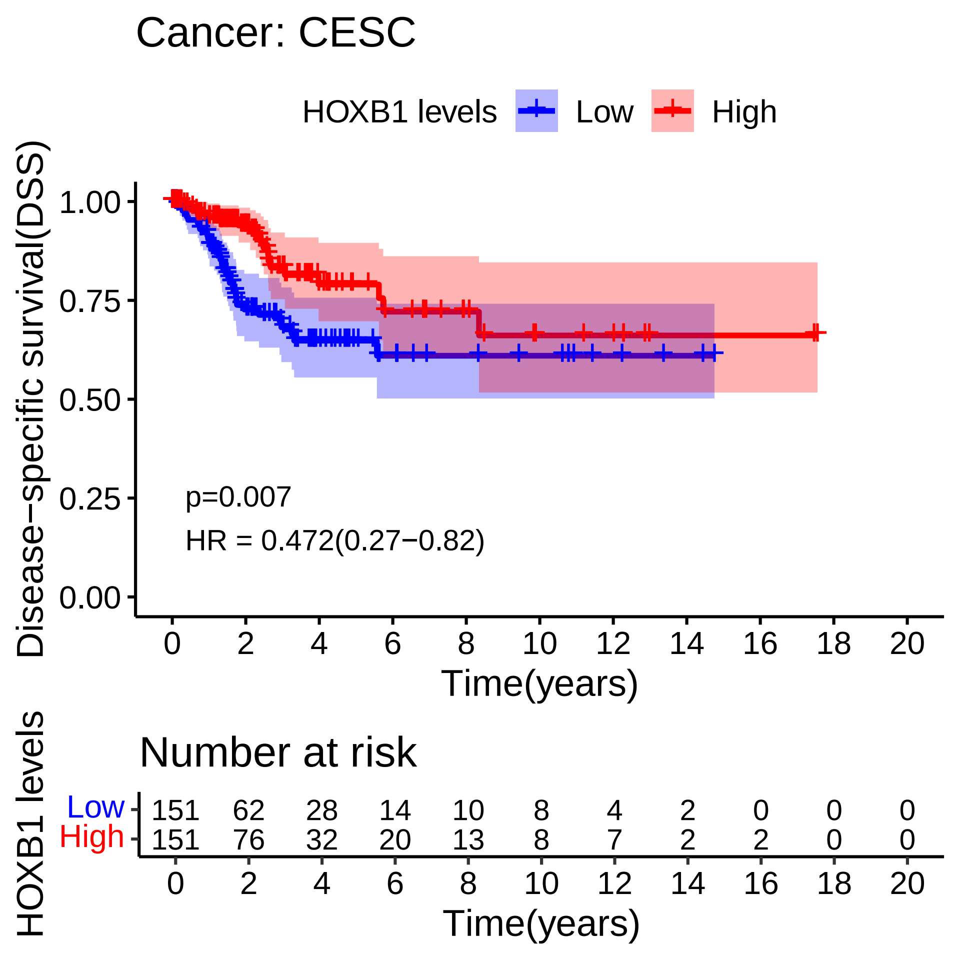

Cancer: CESC
HOXB1 levels
Low
High
Disease−specific survival(DSS)
1.00
0.75
0.50
0.25
0.00
p=0.007
HR = 0.472(0.27−0.82)
0 2 4 6 8 10 12 14 16 18 20
Time(years)
Number at risk
HOXB1 levels
Low 151 62 28 14 10 8 4 2 0 0 0
High 151 76 32 20 13 8 7 2 2 0 0
0 2 4 6 8 10 12 14 16 18 20
Time(years)

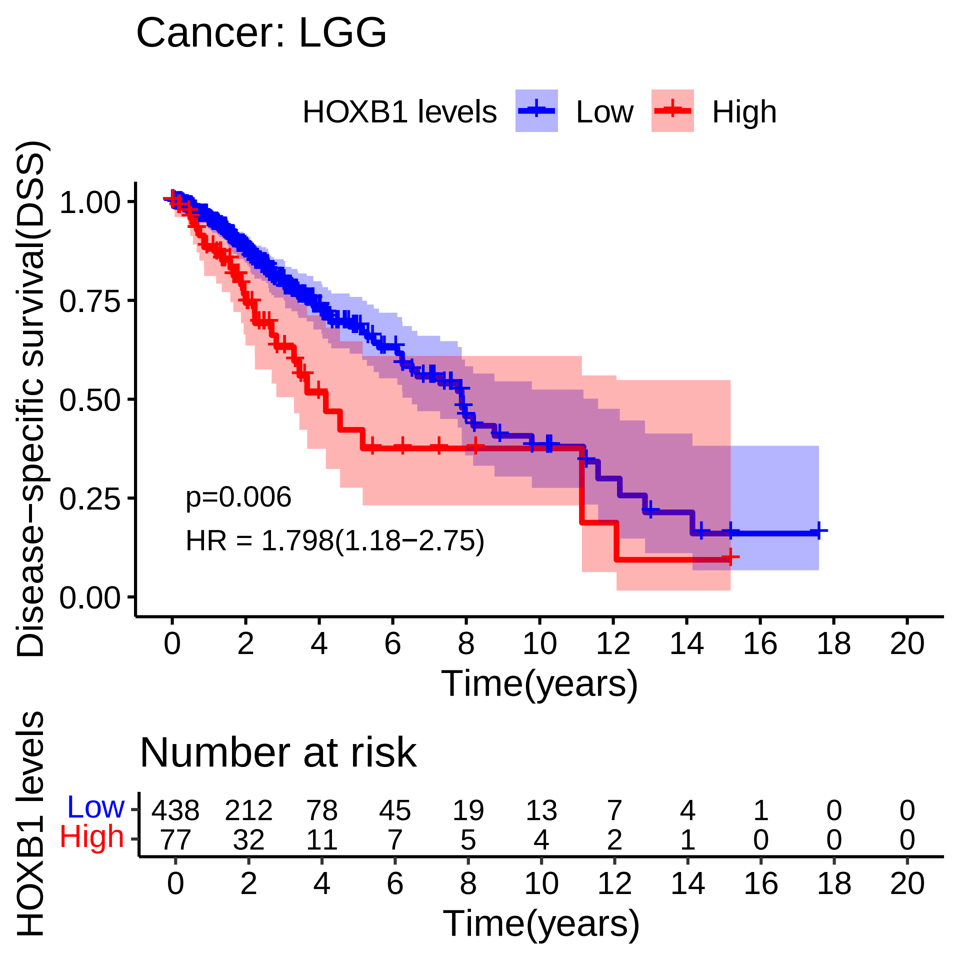

Cancer: LGG
HOXB1 levels
Low
High
Disease−specific survival(DSS)
1.00
0.75
0.50
0.25
0.00
p=0.006
HR = 1.798(1.18−2.75)
0
2
4
6
8
10
12
14
16
18
20
Time(years)
Number at risk
HOXB1 levels
Low
High
438 212 78 45 19 13 7 4 1 0 0
77 32 11 7 5 4 2 1 0 0 0
0
2
4
6
8
10
12
14
16
18
20
Time(years)

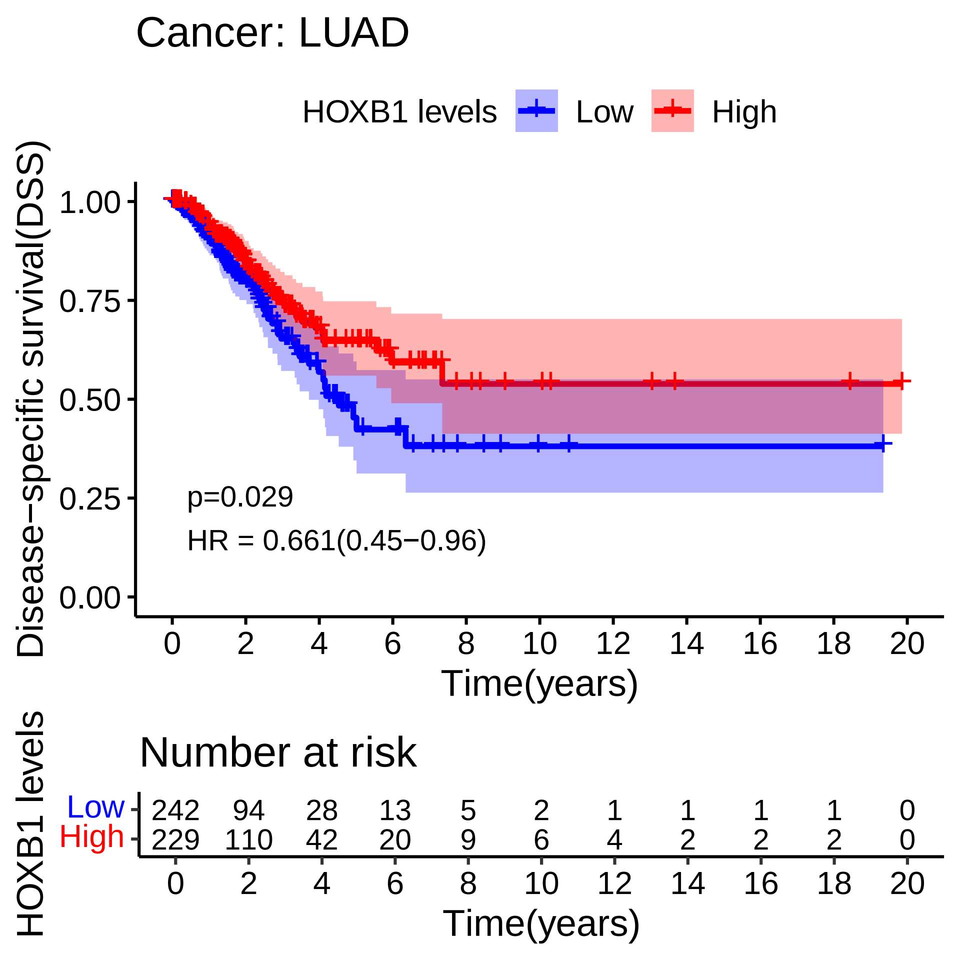

Cancer: LUAD
HOXB1 levels
Low
High
Disease−specific survival(DSS)
1.00
0.75
0.50
0.25
0.00
p=0.029
HR = 0.661(0.45−0.96)
0
2
4
6
8
10
12
14
16
18
20
Time(years)
Number at risk
HOXB1 levels
Low
High
242 94 28 13 5 2 1 1 1 1 0
229 110 42 20 9 6 4 2 2 2 0
0
2
4
6
8
10
12
14
16
18
20
Time(years)

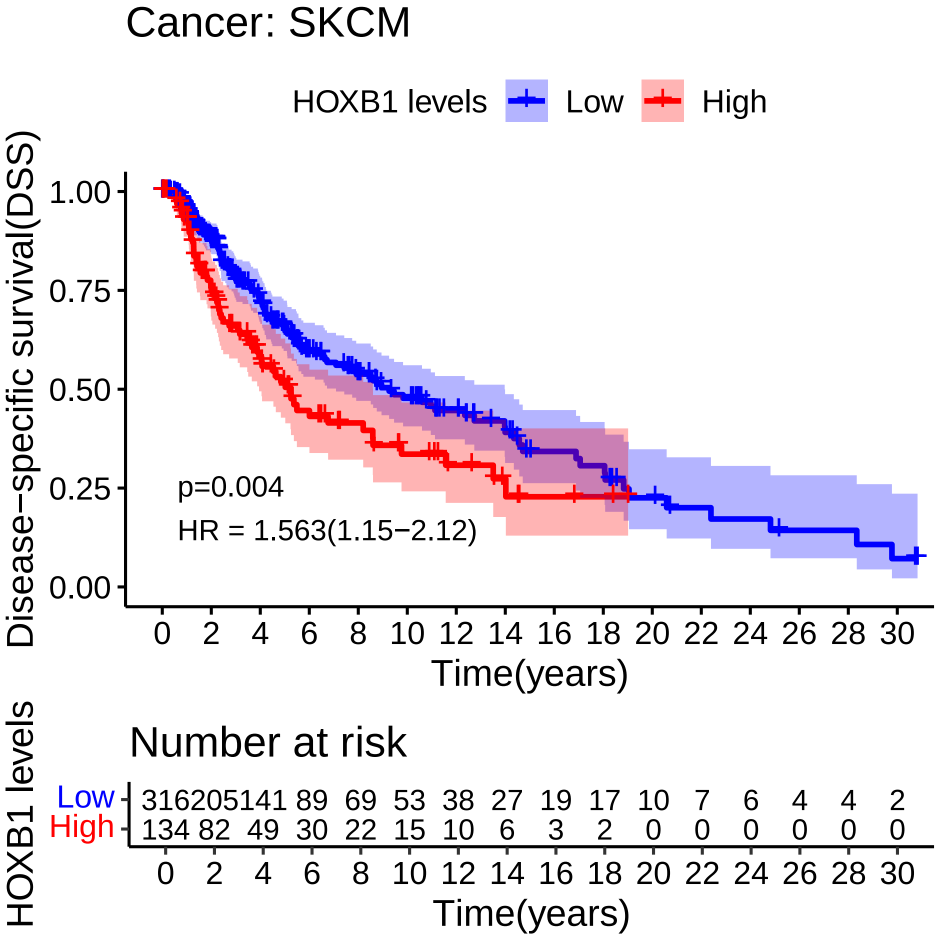

Cancer: SKCM
HOXB1 levels
Low
High
Disease−specific survival(DSS)
1.00
0.75
0.50
0.25
0.00
p=0.004
HR = 1.563(1.15−2.12)
0 2 4 6 8 10 12 14 16 18 20 22 24 26 28 30
Time(years)
Number at risk
HOXB1 levels
Low 316 205 141 89 69 53 38 27 19 17 10 7 6 4 4 2
High 134 82 49 30 22 15 10 6 3 2 0 0 0 0 0 0
0 2 4 6 8 10 12 14 16 18 20 22 24 26 28 30
Time(years)

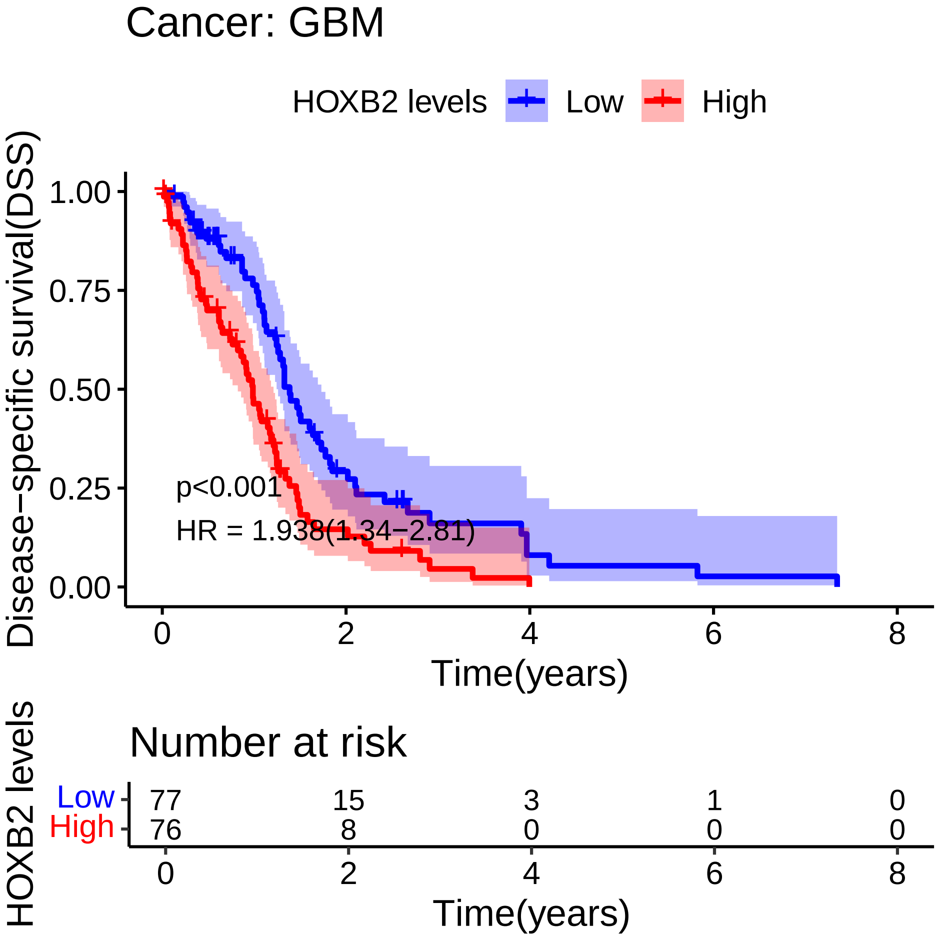
Cancer: GBM
HOXB2 levels
Low
High
Disease−specific survival(DSS)
1.00
0.75
0.50
0.25
0.00
p<0.001
HR = 1.938(1.34−2.81)
0
2
4
6
8
Time(years)
Number at risk
HOXB2 levels
Low
High
77
15
3
1
0
76
8
0
0
0
0
2
4
6
8
Time(years)

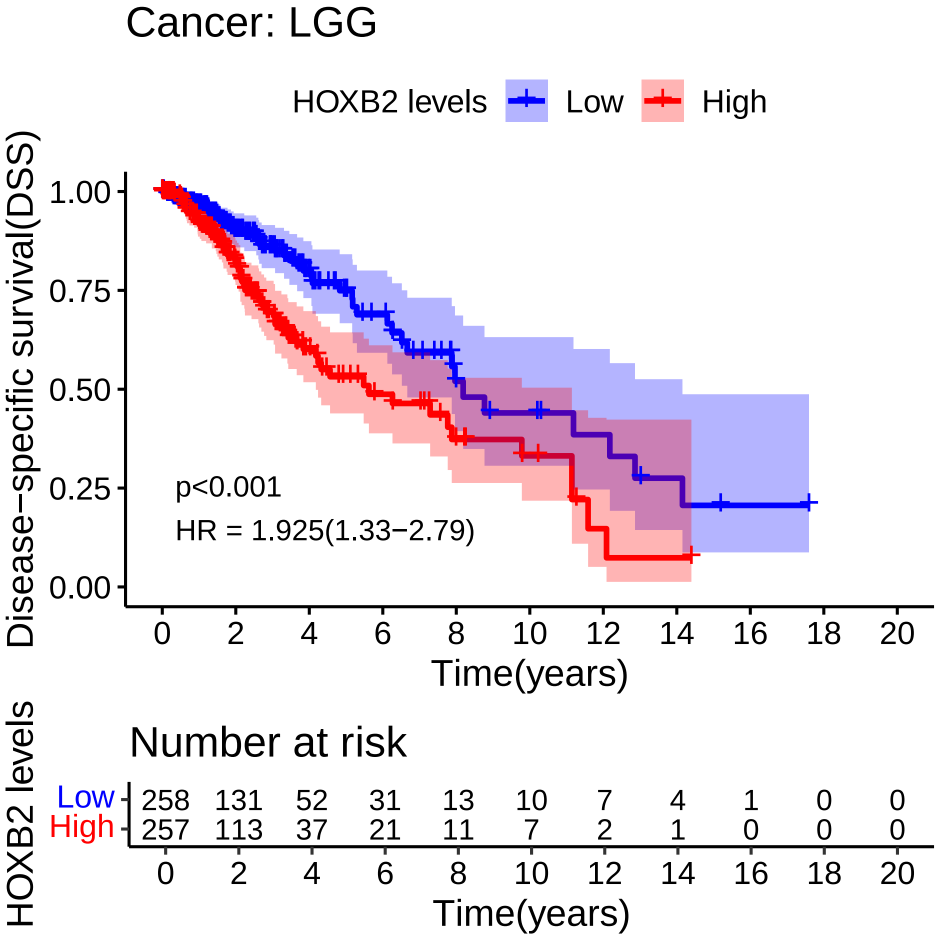

Cancer: LGG
HOXB2 levels
Low
High
Disease−specific survival(DSS)
1.00
0.75
0.50
0.25
0.00
p<0.001
HR = 1.925(1.33−2.79)
0 2 4 6 8 10 12 14 16 18 20
Time(years)
Number at risk
HOXB2 levels
Low 258 131 52 31 13 10 7 4 1 0 0
High 257 113 37 21 11 7 2 1 0 0 0
0 2 4 6 8 10 12 14 16 18 20
Time(years)

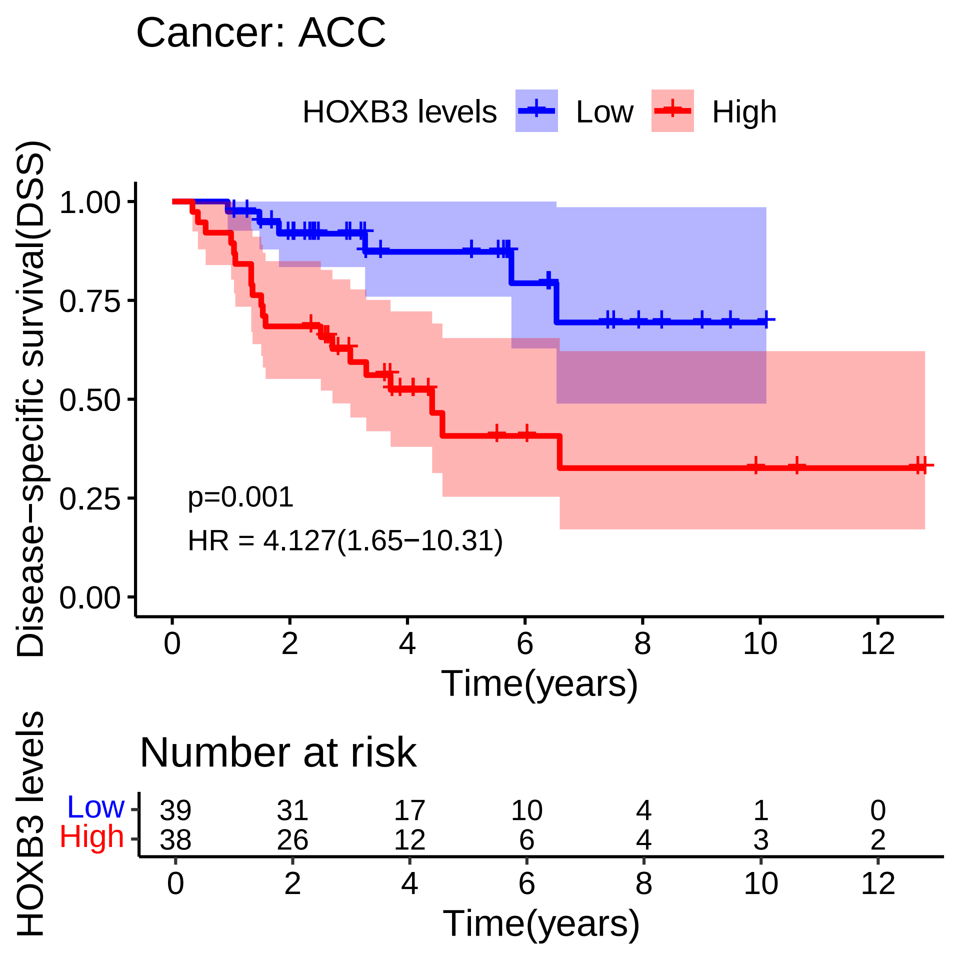

Cancer: ACC
HOXB3 levels
Low
High
Disease−specific survival(DSS)
1.00
0.75
0.50
0.25
0.00
p=0.001
HR = 4.127(1.65−10.31)
0
2
4
6
8
10
12
Time(years)
Number at risk
HOXB3 levels
Low 39 31 17 10 4 1 0
High 38 26 12 6 4 3 2
Time(years)

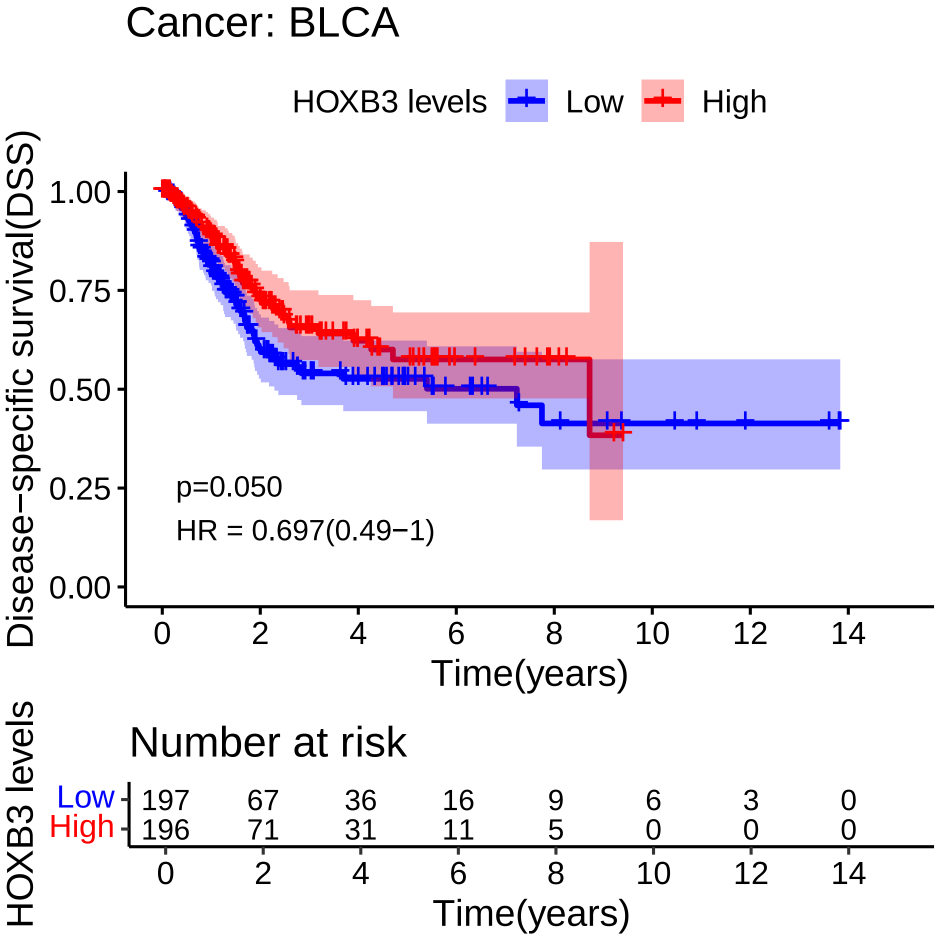

Cancer: BLCA
HOXB3 levels
Low
High
Disease−specific survival(DSS)
1.00
0.75
0.50
0.25
0.00
p=0.050
HR = 0.697(0.49−1)
0
2
4
6
8
10
12
14
Time(years)
Number at risk
HOXB3 levels
Low 197 67 36 16 9 6 3 0
High 196 71 31 11 5 0 0 0
Time(years)

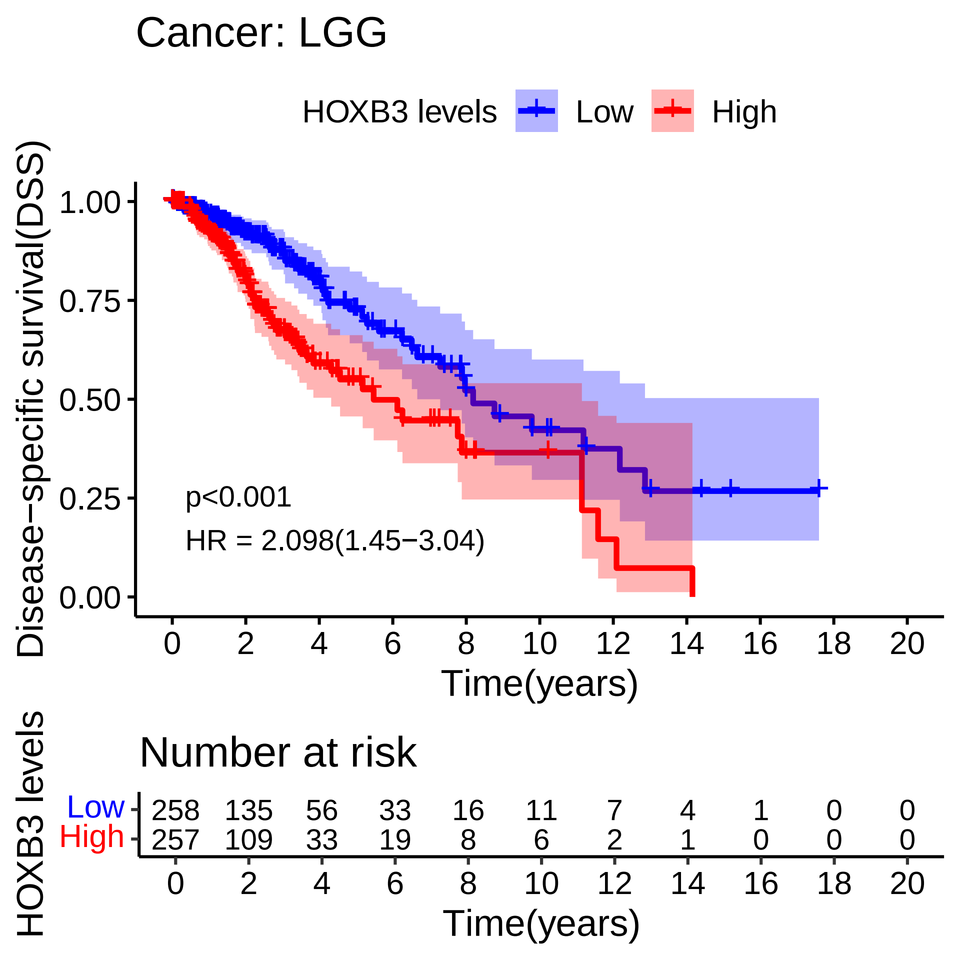
Cancer: LGG
HOXB3 levels
Low
High
Disease−specific survival(DSS)
1.00
0.75
0.50
0.25
0.00
p<0.001
HR = 2.098(1.45−3.04)
0
2
4
6
8
10
12
14
16
18
20
Time(years)
Number at risk
HOXB3 levels
Low 258 135 56 33 16 11 7 4 1 0 0
High 257 109 33 19 8 6 2 1 0 0 0
Time(years)

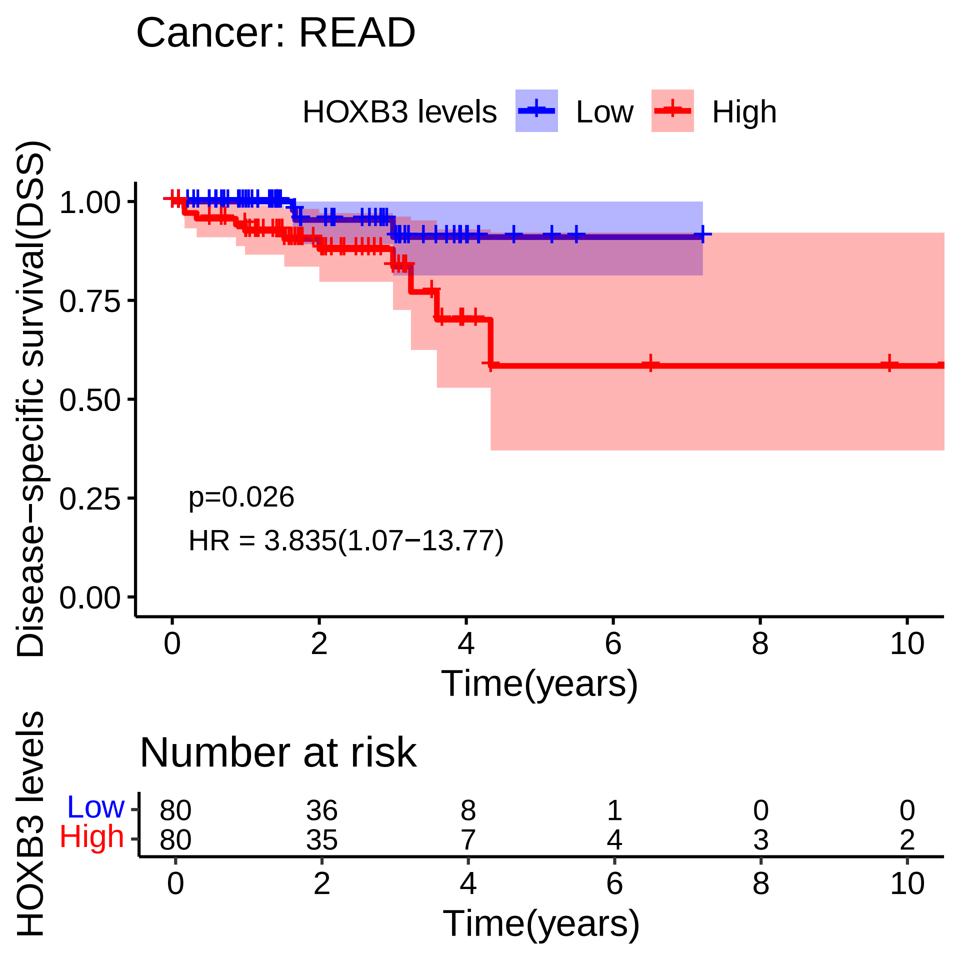

Cancer: READ
HOXB3 levels
Low
High
Disease−specific survival(DSS)
1.00
0.75
0.50
0.25
0.00
p=0.026
HR = 3.835(1.07−13.77)
0
2
4
6
8
10
Time(years)
Number at risk
HOXB3 levels
Low 80 36 8 1 0 0
High 80 35 7 4 3 2
0
2
4
6
8
10
Time(years)

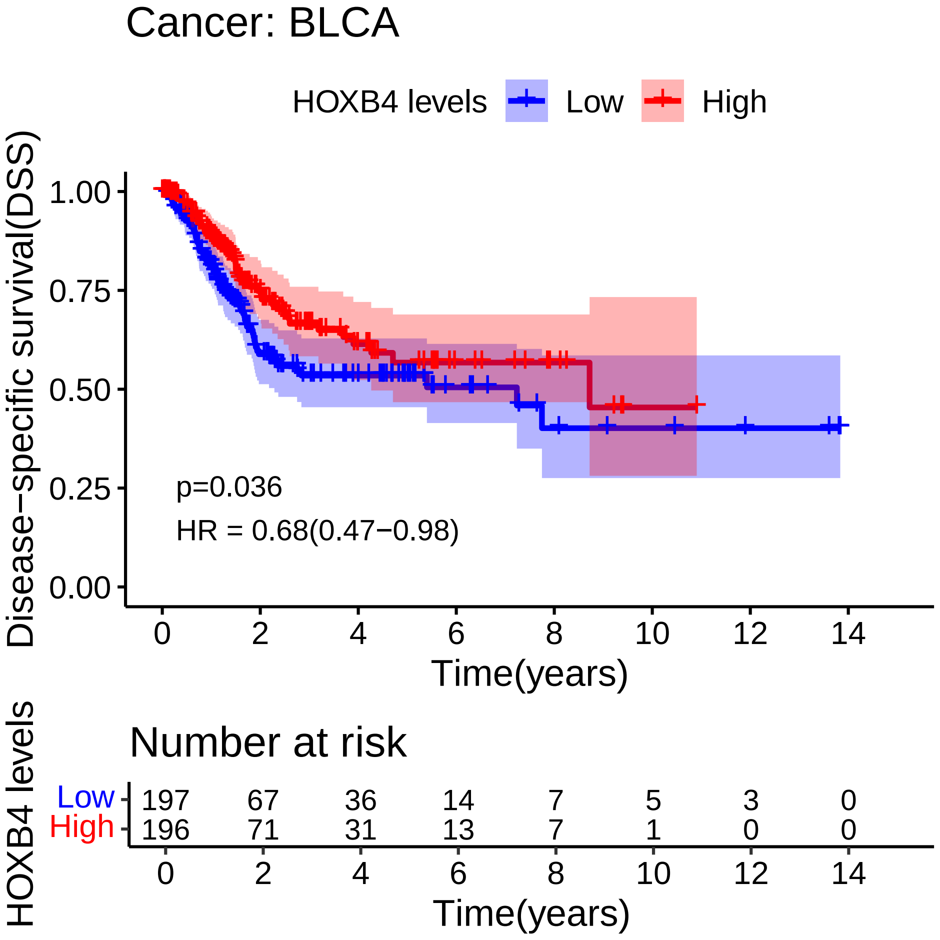

Cancer: BLCA
HOXB4 levels
Low
High
Disease−specific survival(DSS)
1.00
0.75
0.50
0.25
0.00
p=0.036
HR = 0.68(0.47−0.98)
0
2
4
6
8
10
12
14
Time(years)
Number at risk
HOXB4 levels
Low 197 67 36 14 7 5 3 0
High 196 71 31 13 7 1 0 0
Time(years)

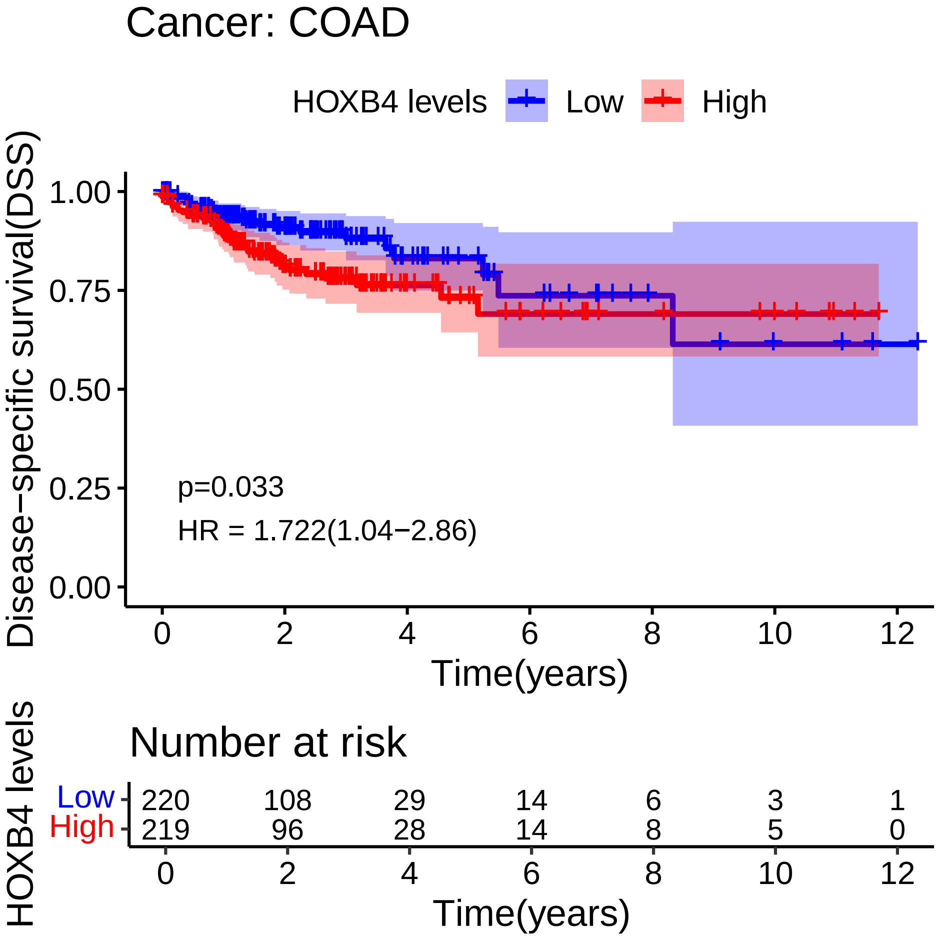
Cancer: COAD
HOXB4 levels
Low
High
Disease−specific survival(DSS)
1.00
0.75
0.50
0.25
0.00
p=0.033
HR = 1.722(1.04−2.86)
0
2
4
6
8
10
12
Time(years)
Number at risk
HOXB4 levels
Low 220 108 29 14 6 3 1
High 219 96 28 14 8 5 0
0
2
4
6
8
10
12
Time(years)

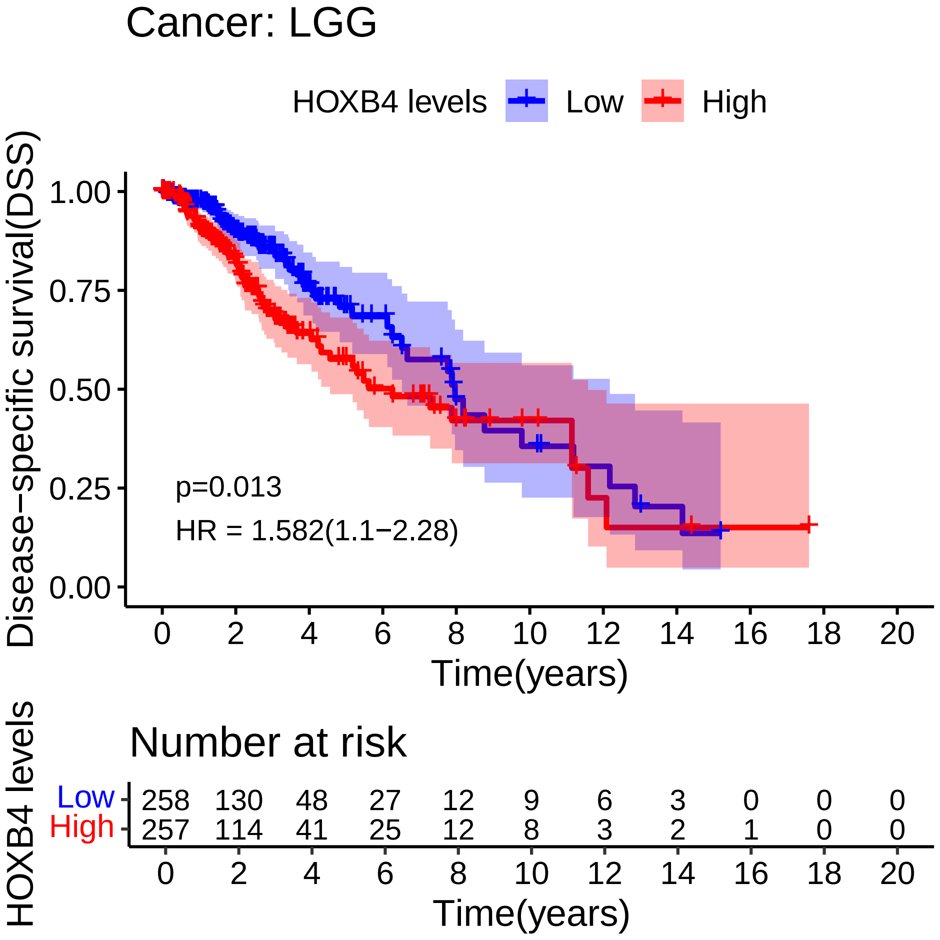

Cancer: LGG
HOXB4 levels
Low
High
Disease−specific survival(DSS)
1.00
0.75
0.50
0.25
0.00
p=0.013
HR = 1.582(1.1−2.28)
0 2 4 6 8 10 12 14 16 18 20
Time(years)
Number at risk
HOXB4 levels
Low 258 130 48 27 12 9 6 3 0 0 0
High 257 114 41 25 12 8 3 2 1 0 0
0 2 4 6 8 10 12 14 16 18 20
Time(years)

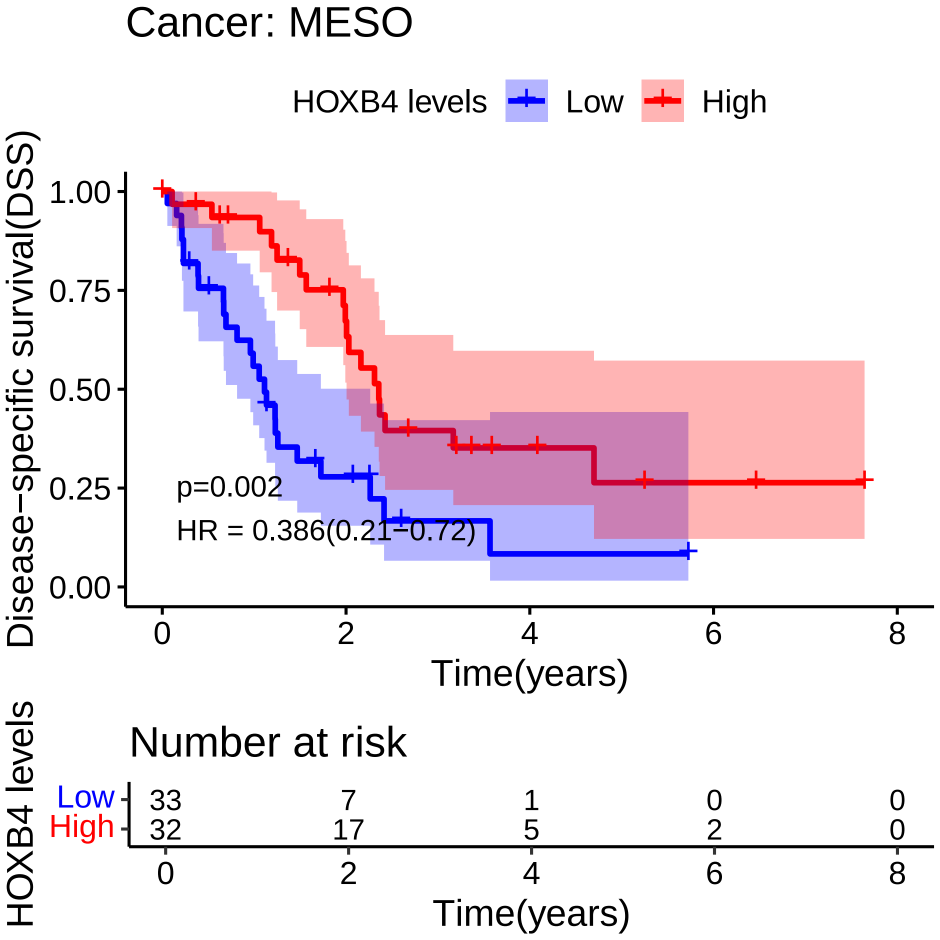
Cancer: MESO
HOXB4 levels
Low
High
Disease−specific survival(DSS)
1.00
0.75
0.50
0.25
0.00
p=0.002
HR = 0.386(0.21−0.72)
0
2
4
6
8
Time(years)
Number at risk
HOXB4 levels
Low
High
33
7
1
0
0
32
17
5
2
0
0
2
4
6
8
Time(years)

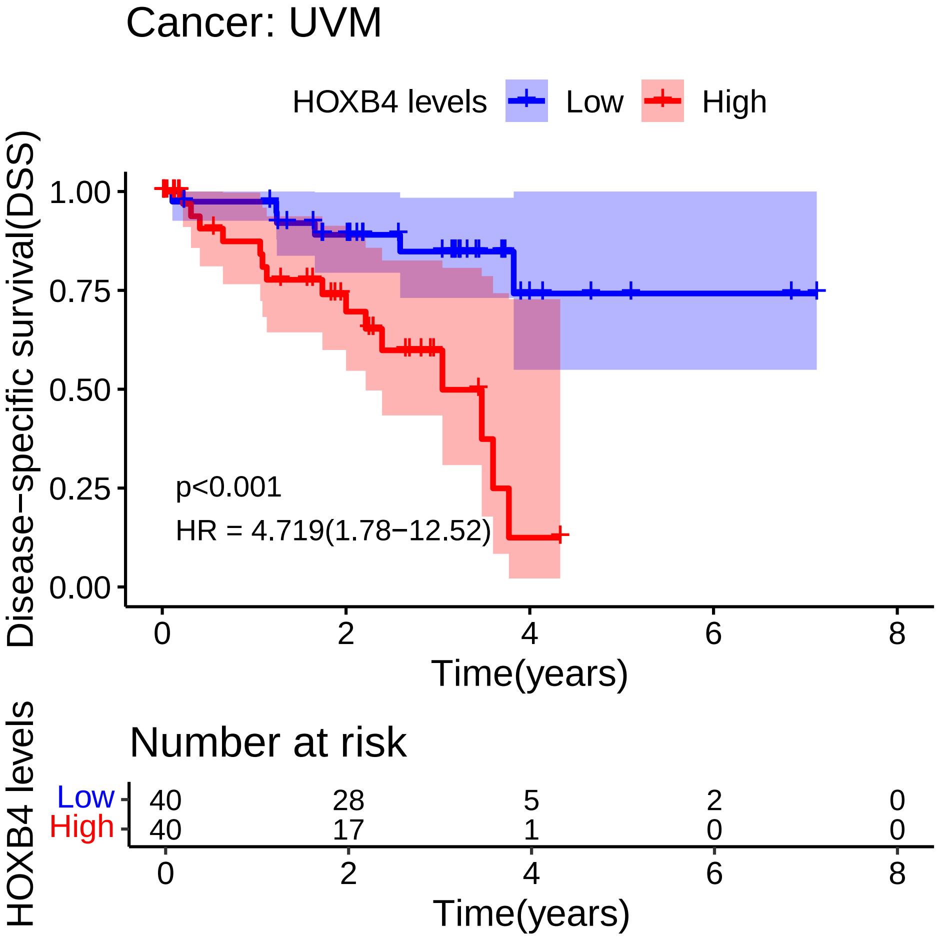

Cancer: UVM
HOXB4 levels
Low
High
Disease−specific survival(DSS)
1.00
0.75
0.50
0.25
0.00
p<0.001
HR = 4.719(1.78−12.52)
0
2
4
6
8
Time(years)
Number at risk
HOXB4 levels
Low
High
40 28 5 2 0
40 17 1 0 0
0
2
4
6
8
Time(years)

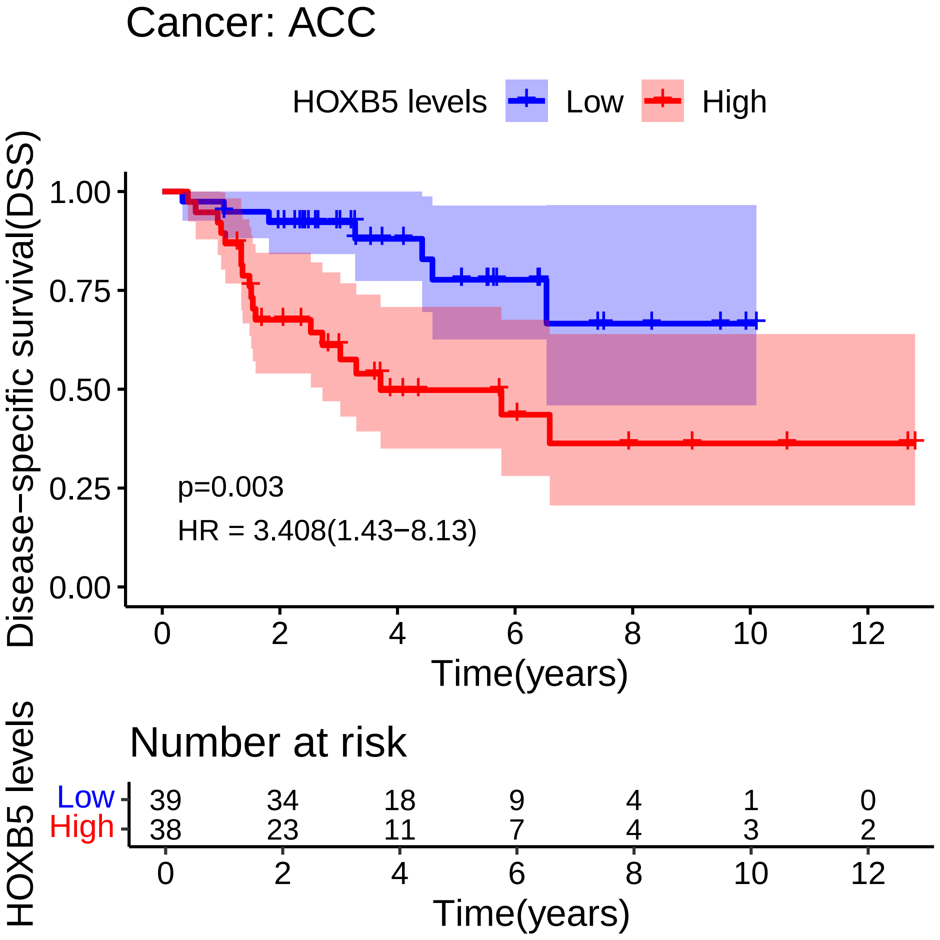

Cancer: ACC
HOXB5 levels
Low
High
Disease−specific survival(DSS)
1.00
0.75
0.50
0.25
0.00
p=0.003
HR = 3.408(1.43−8.13)
0
2
4
6
8
10
12
Time(years)
Number at risk
HOXB5 levels
Low 39 34 18 9 4 1 0
High 38 23 11 7 4 3 2
Time(years)

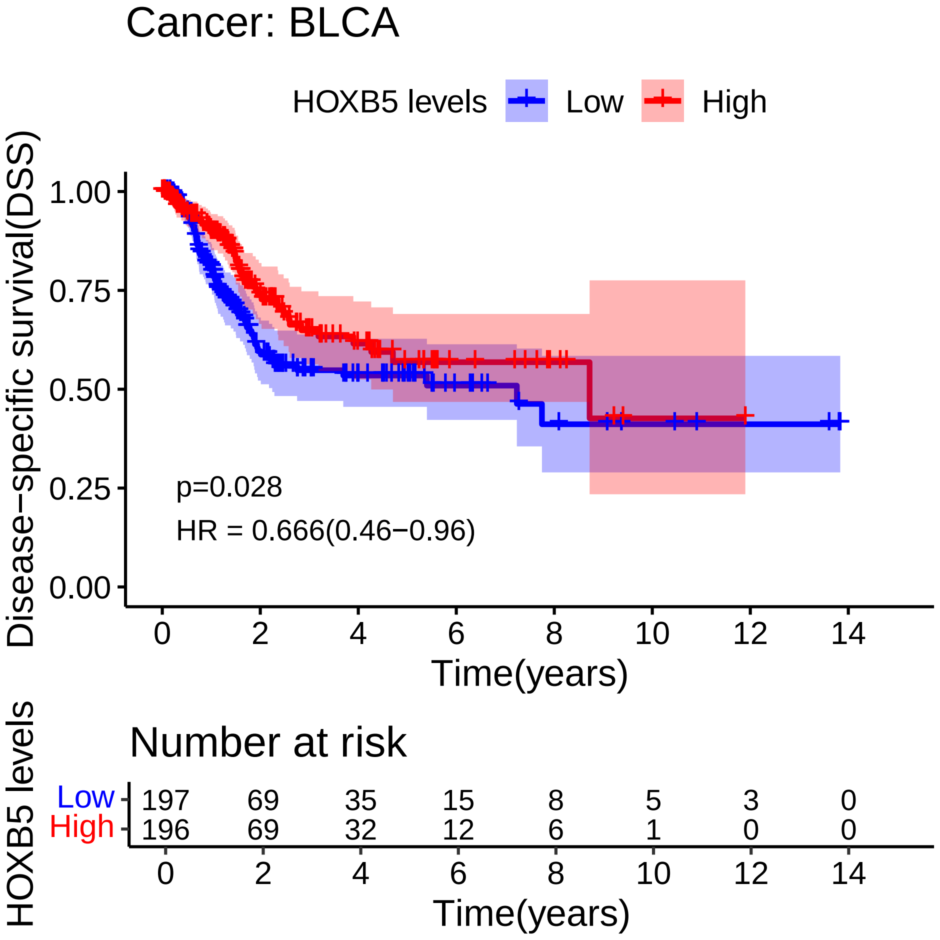

Cancer: BLCA
HOXB5 levels
Low
High
Disease−specific survival(DSS)
1.00
0.75
0.50
0.25
0.00
p=0.028
HR = 0.666(0.46−0.96)
0 2 4 6 8 10 12 14
Time(years)
Number at risk
HOXB5 levels
Low 197 69 35 15 8 5 3 0
High 196 69 32 12 6 1 0 0
0 2 4 6 8 10 12 14
Time(years)

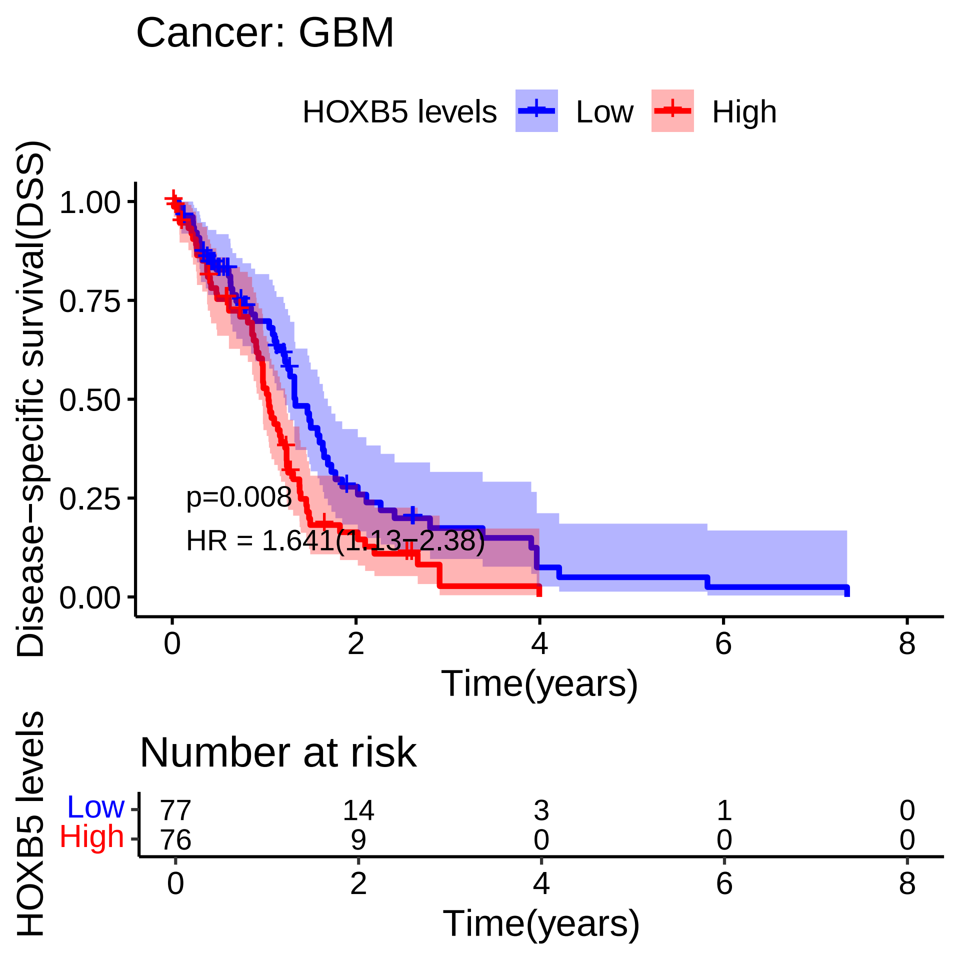

Cancer: GBM
HOXB5 levels
Low
High
Disease−specific survival(DSS)
1.00
0.75
0.50
0.25
0.00
p=0.008
HR = 1.641(1.13−2.38)
0
2
4
6
8
Time(years)
Number at risk
HOXB5 levels
Low
High
77
14
3
1
0
76
9
0
0
0
0
2
4
6
8
Time(years)

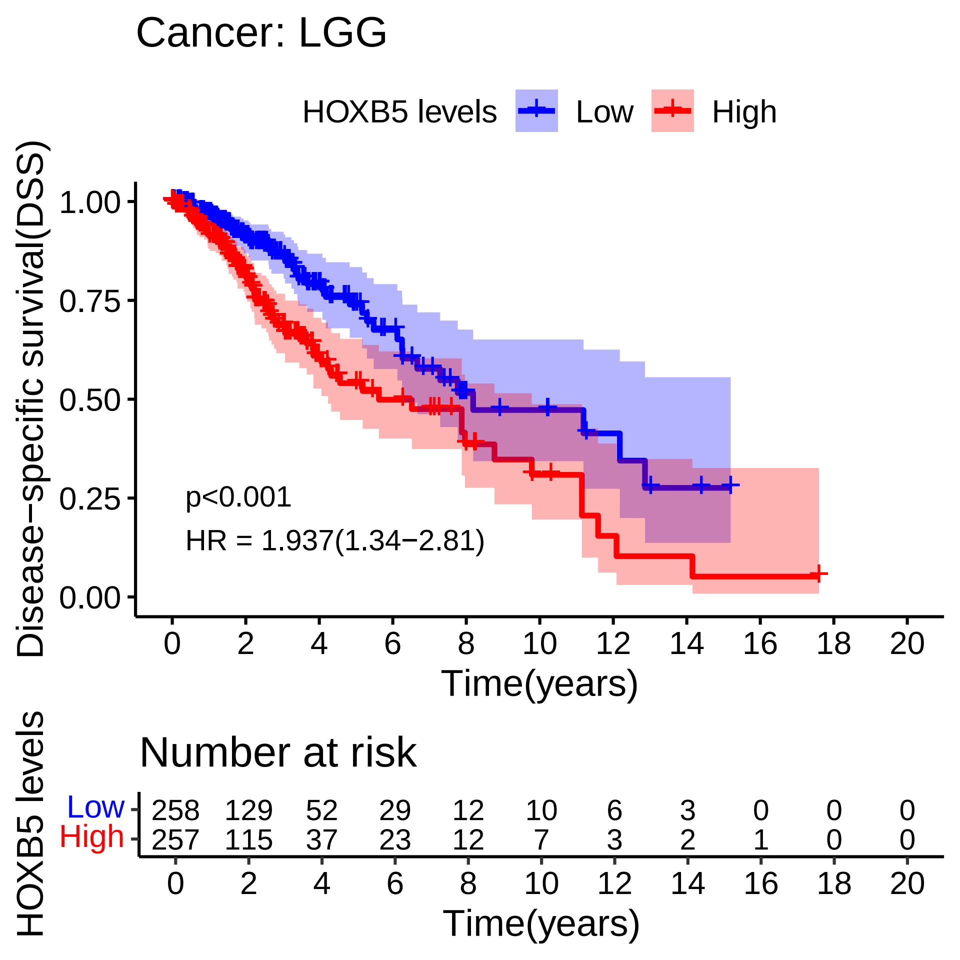

Cancer: LGG
HOXB5 levels
Low
High
Disease−specific survival(DSS)
1.00
0.75
0.50
0.25
0.00
p<0.001
HR = 1.937(1.34−2.81)
0
2
4
6
8
10
12
14
16
18
20
Time(years)
Number at risk
HOXB5 levels
Low 258 129 52 29 12 10 6 3 0 0 0
High 257 115 37 23 12 7 3 2 1 0 0
0
2
4
6
8
10
12
14
16
18
20
Time(years)

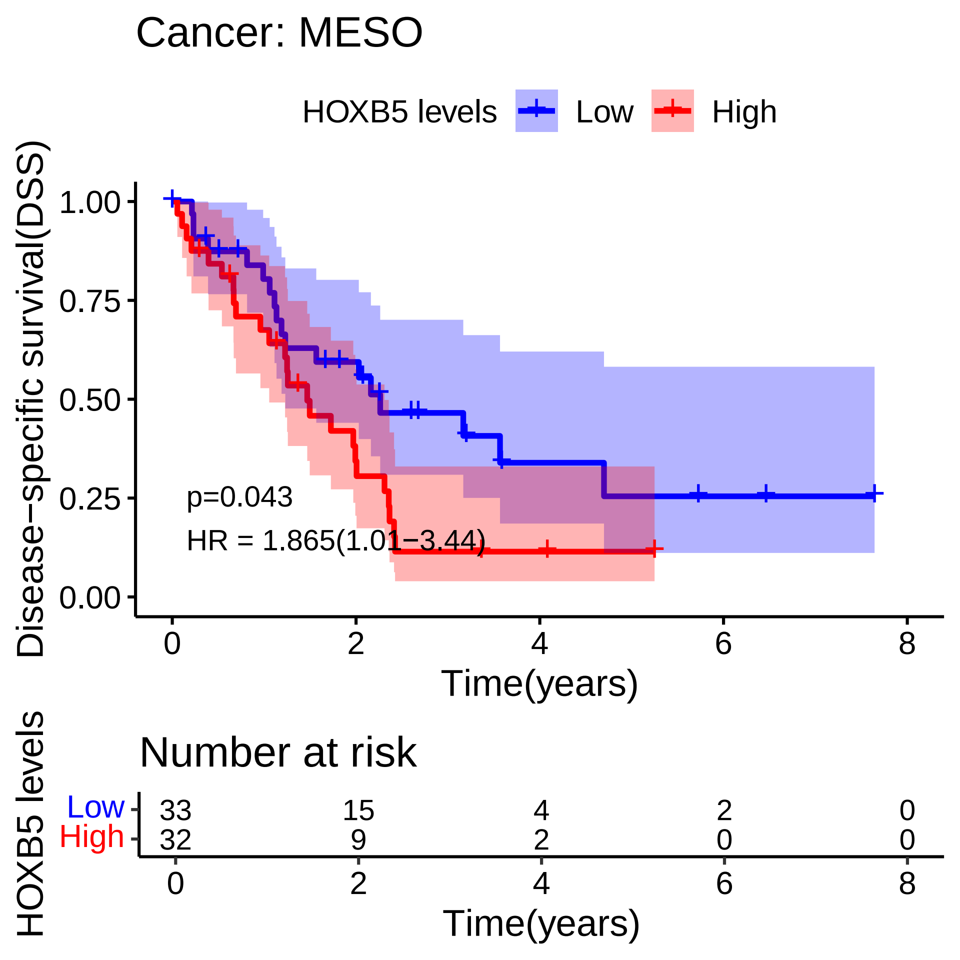
Cancer: MESO
HOXB5 levels
Low
High
Disease−specific survival(DSS)
1.00
0.75
0.50
0.25
0.00
p=0.043
HR = 1.865(1.01−3.44)
0
2
4
6
8
Time(years)
Number at risk
HOXB5 levels
Low 33 15 4 2 0
High 32 9 2 0 0
0
2
4
6
8
Time(years)

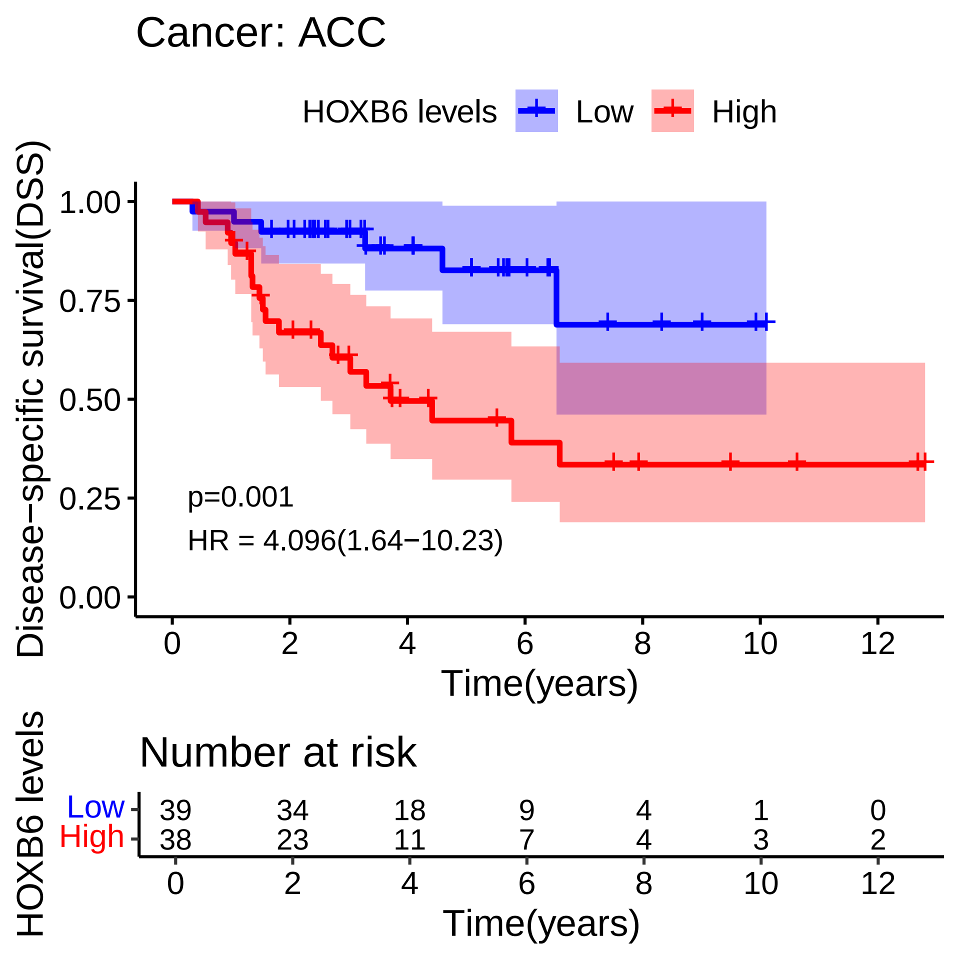

Cancer: ACC
HOXB6 levels
Low
High
Disease−specific survival(DSS)
1.00
0.75
0.50
0.25
0.00
p=0.001
HR = 4.096(1.64−10.23)
0
2
4
6
8
10
12
Time(years)
Number at risk
HOXB6 levels
Low 39 34 18 9 4 1 0
High 38 23 11 7 4 3 2
Time(years)

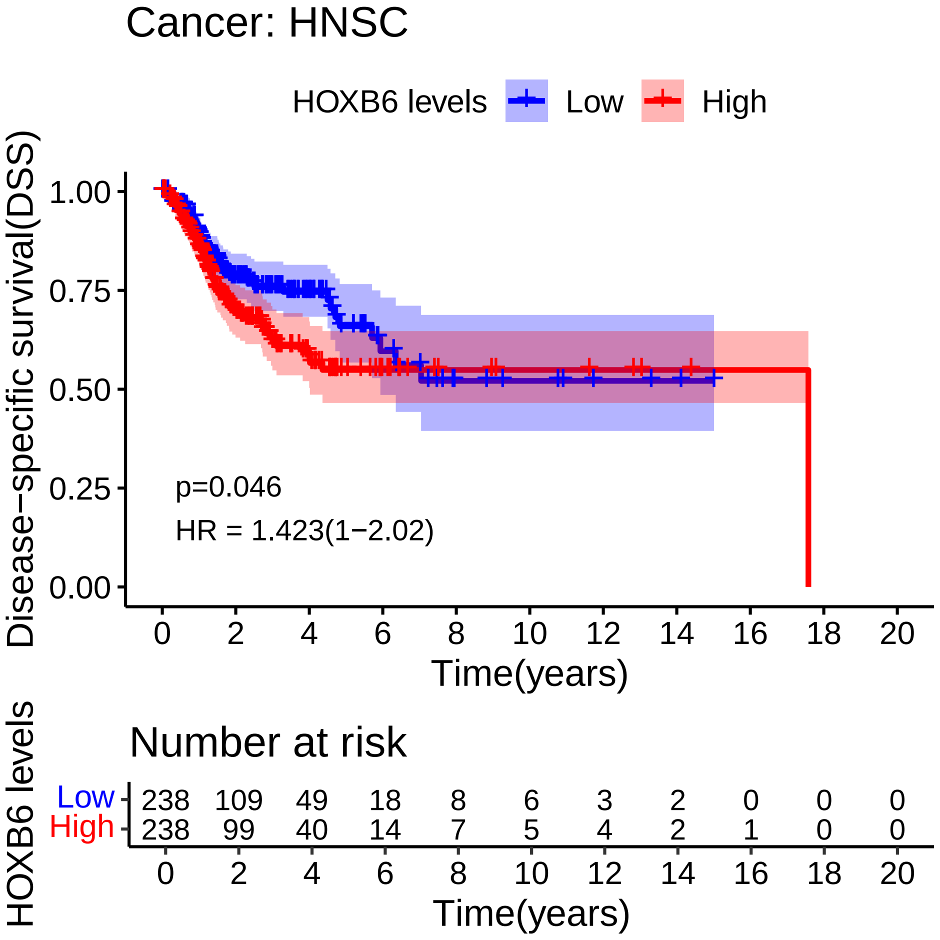

Cancer: HNSC
HOXB6 levels
Low
High
Disease−specific survival(DSS)
1.00
0.75
0.50
0.25
0.00
p=0.046
HR = 1.423(1−2.02)
0
2
4
6
8
10
12
14
16
18
20
Time(years)
Number at risk
HOXB6 levels
Low 238 109 49 18 8 6 3 2 0 0 0
High 238 99 40 14 7 5 4 2 1 0 0
0
2
4
6
8
10
12
14
16
18
20
Time(years)

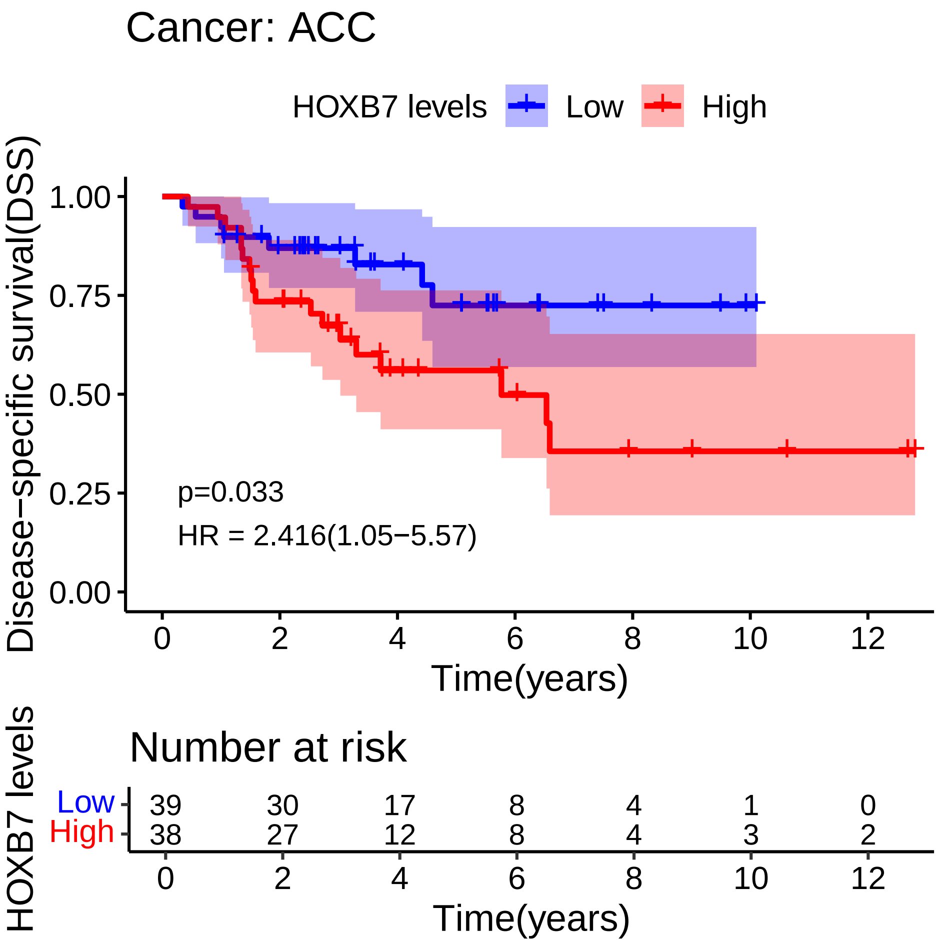

Cancer: ACC
HOXB7 levels
Low
High
Disease−specific survival(DSS)
1.00
0.75
0.50
0.25
0.00
p=0.033
HR = 2.416(1.05−5.57)
0
2
4
6
8
10
12
Time(years)
Number at risk
HOXB7 levels
Low 39 30 17 8 4 1 0
High 38 27 12 8 4 3 2
0
2
4
6
8
10
12
Time(years)

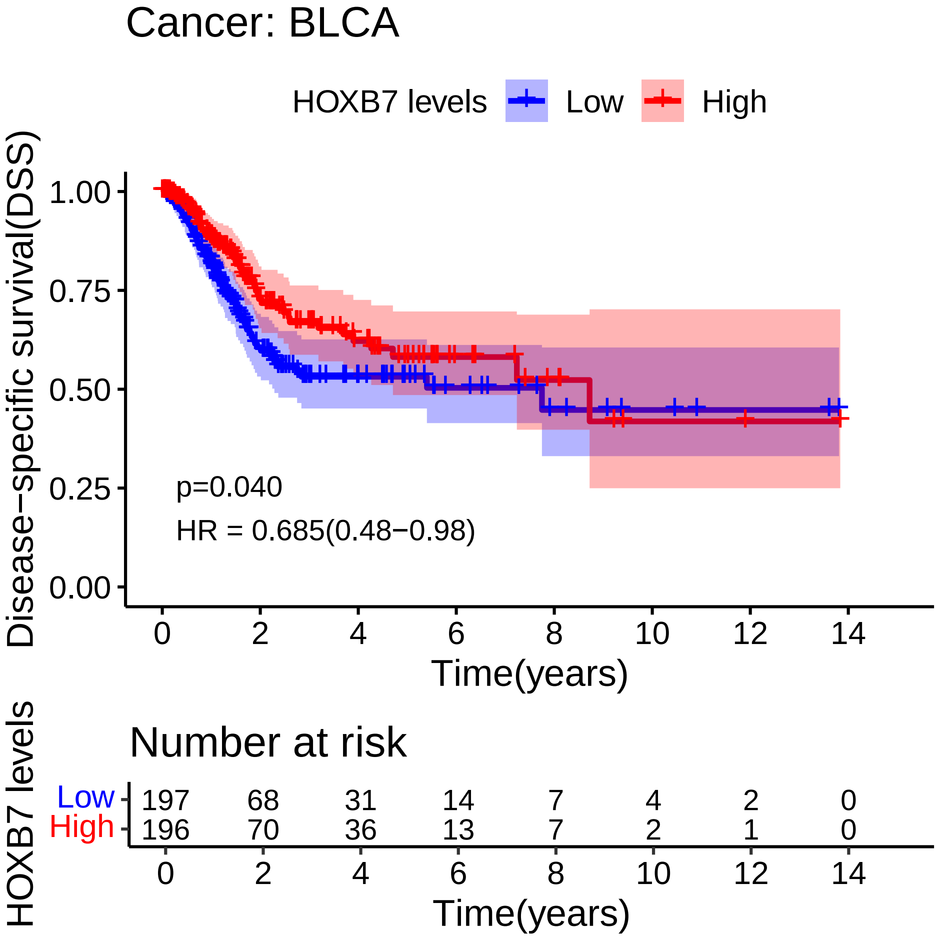
Cancer: BLCA
HOXB7 levels
Low
High
Disease−specific survival(DSS)
1.00
0.75
0.50
0.25
0.00
p=0.040
HR = 0.685(0.48−0.98)
0
2
4
6
8
10
12
14
Time(years)
Number at risk
HOXB7 levels
Low 197 68 31 14 7 4 2 0
High 196 70 36 13 7 2 1 0
0
2
4
6
8
10
12
14
Time(years)

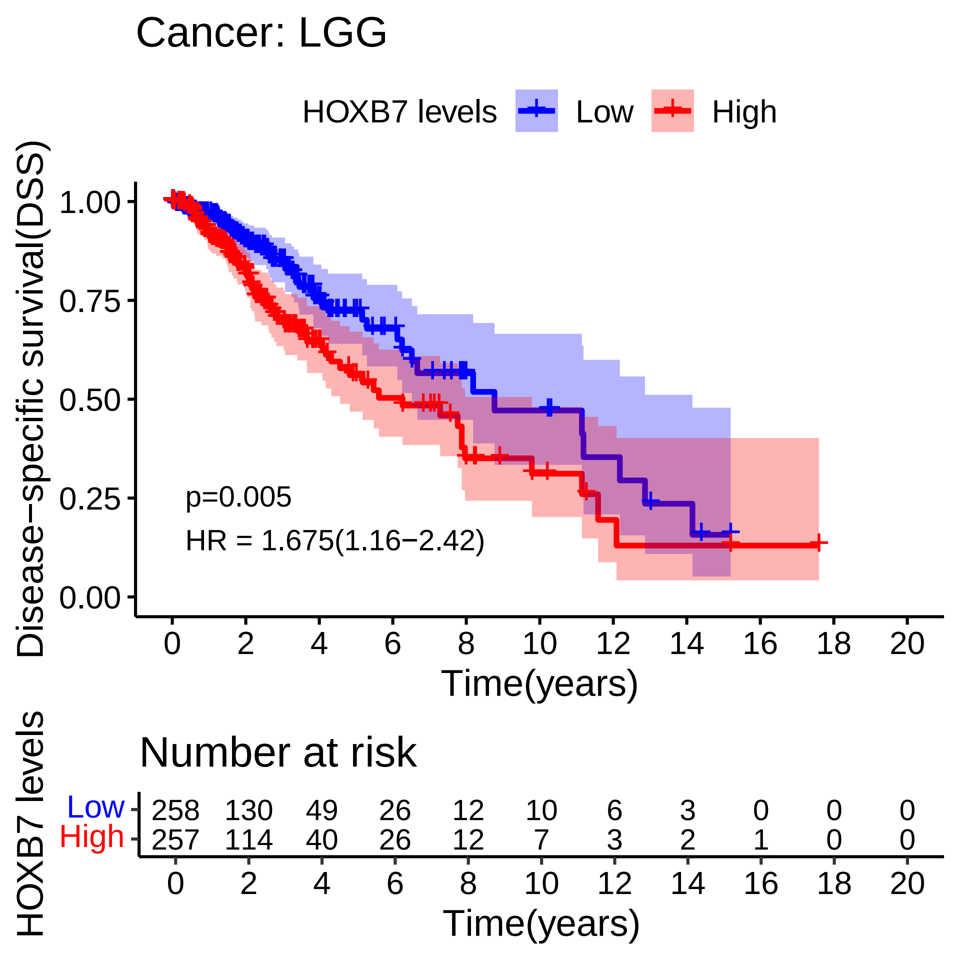

Cancer: LGG
HOXB7 levels
Low
High
Disease−specific survival(DSS)
1.00
0.75
0.50
0.25
0.00
p=0.005
HR = 1.675(1.16−2.42)
0 2 4 6 8 10 12 14 16 18 20
Time(years)
Number at risk
HOXB7 levels
Low 258 130 49 26 12 10 6 3 0 0 0
High 257 114 40 26 12 7 3 2 1 0 0
0 2 4 6 8 10 12 14 16 18 20
Time(years)

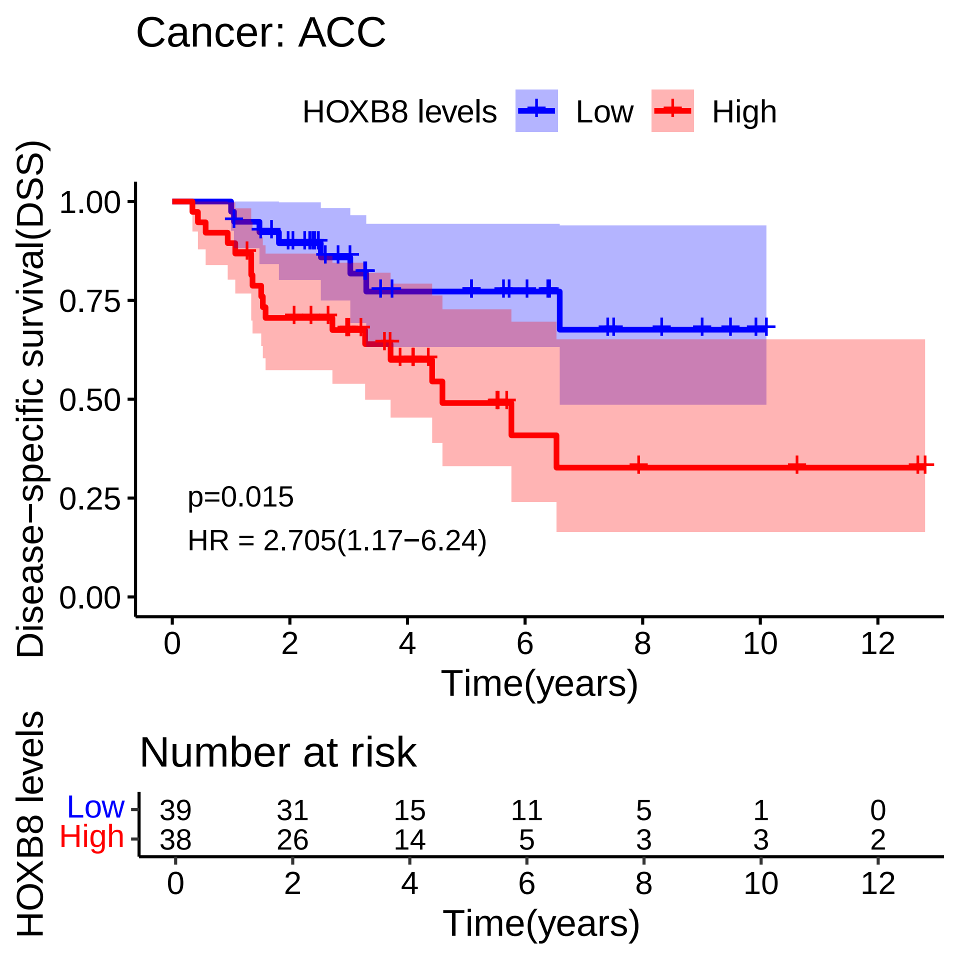
Cancer: ACC
HOXB8 levels
Low
High
Disease−specific survival(DSS)
1.00
0.75
0.50
0.25
0.00
p=0.015
HR = 2.705(1.17−6.24)
0
2
4
6
8
10
12
Time(years)
Number at risk
HOXB8 levels
Low 39 31 15 11 5 1 0
High 38 26 14 5 3 3 2
0
2
4
6
8
10
12
Time(years)

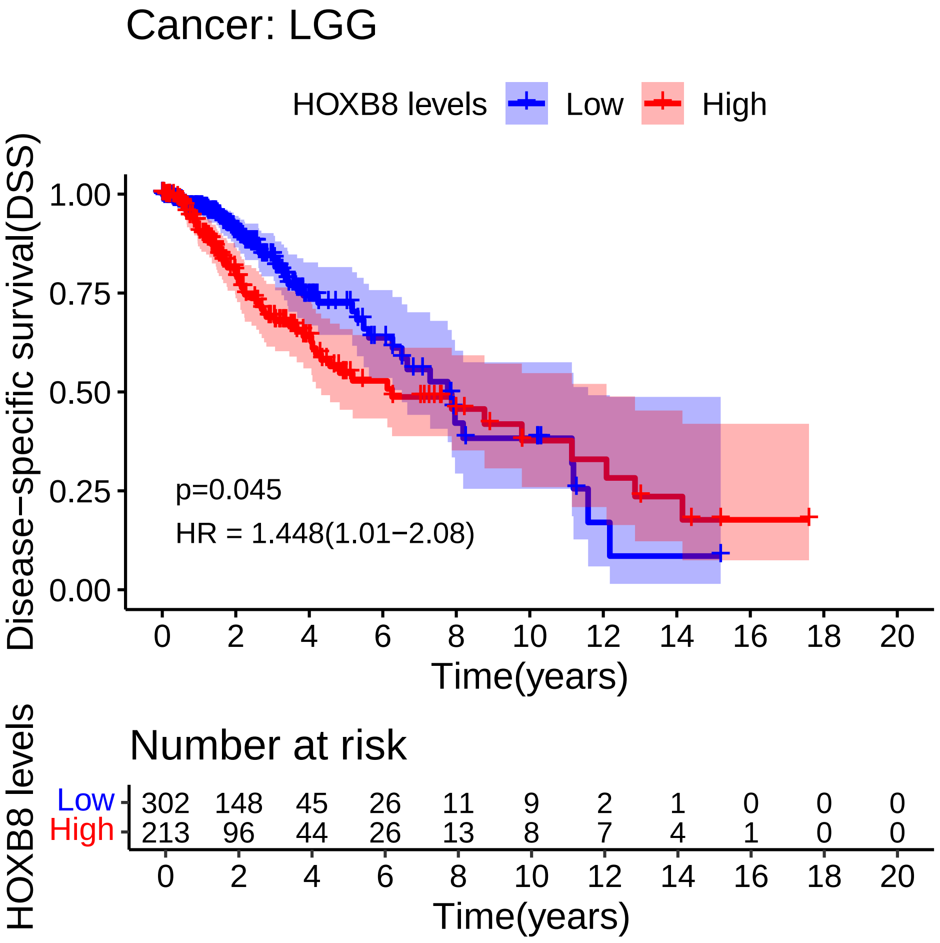
Cancer: LGG
HOXB8 levels
Low
High
Disease−specific survival(DSS)
1.00
0.75
0.50
0.25
0.00
p=0.045
HR = 1.448(1.01−2.08)
0
2
4
6
8
10
12
14
16
18
20
Time(years)
Number at risk
HOXB8 levels
Low 302 148 45 26 11 9 2 1 0 0 0
High 213 96 44 26 13 8 7 4 1 0 0
0
2
4
6
8
10
12
14
16
18
20
Time(years)

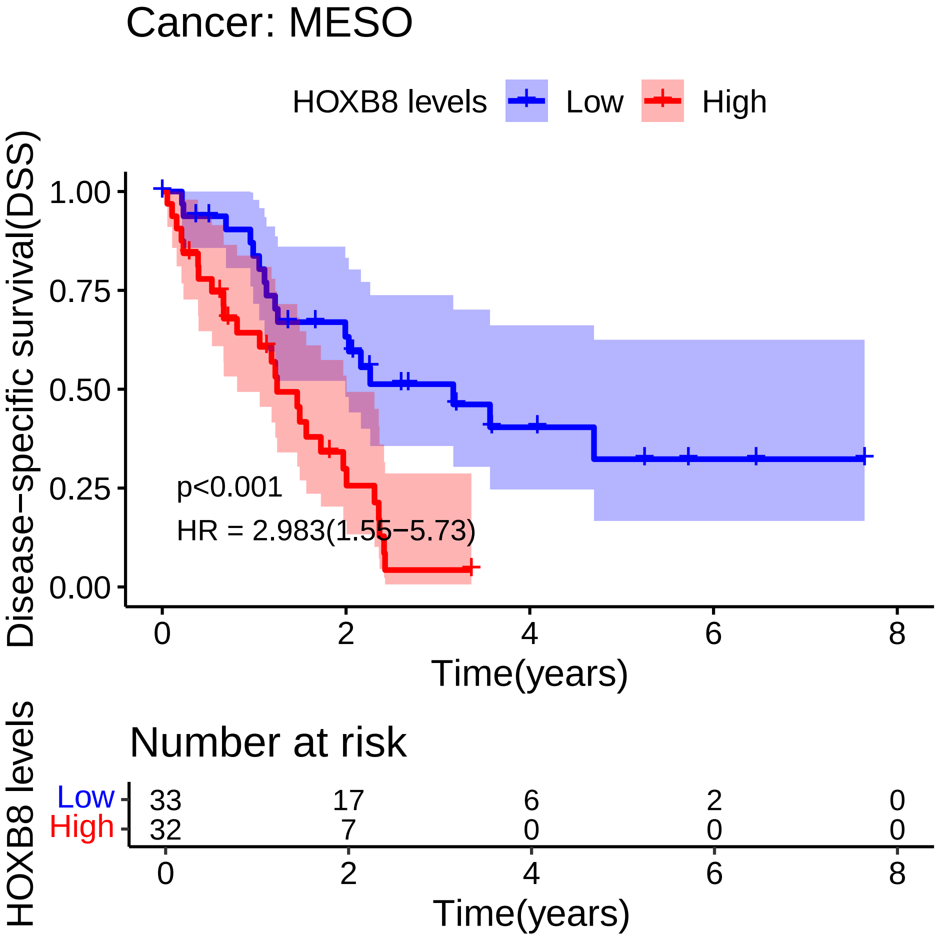

Cancer: MESO
HOXB8 levels
Low
High
Disease−specific survival(DSS)
1.00
0.75
0.50
0.25
0.00
p<0.001
HR = 2.983(1.55−5.73)
0
2
4
6
8
Time(years)
Number at risk
HOXB8 levels
Low
High
33
17
6
2
0
32
7
0
0
0
0
2
4
6
8
Time(years)

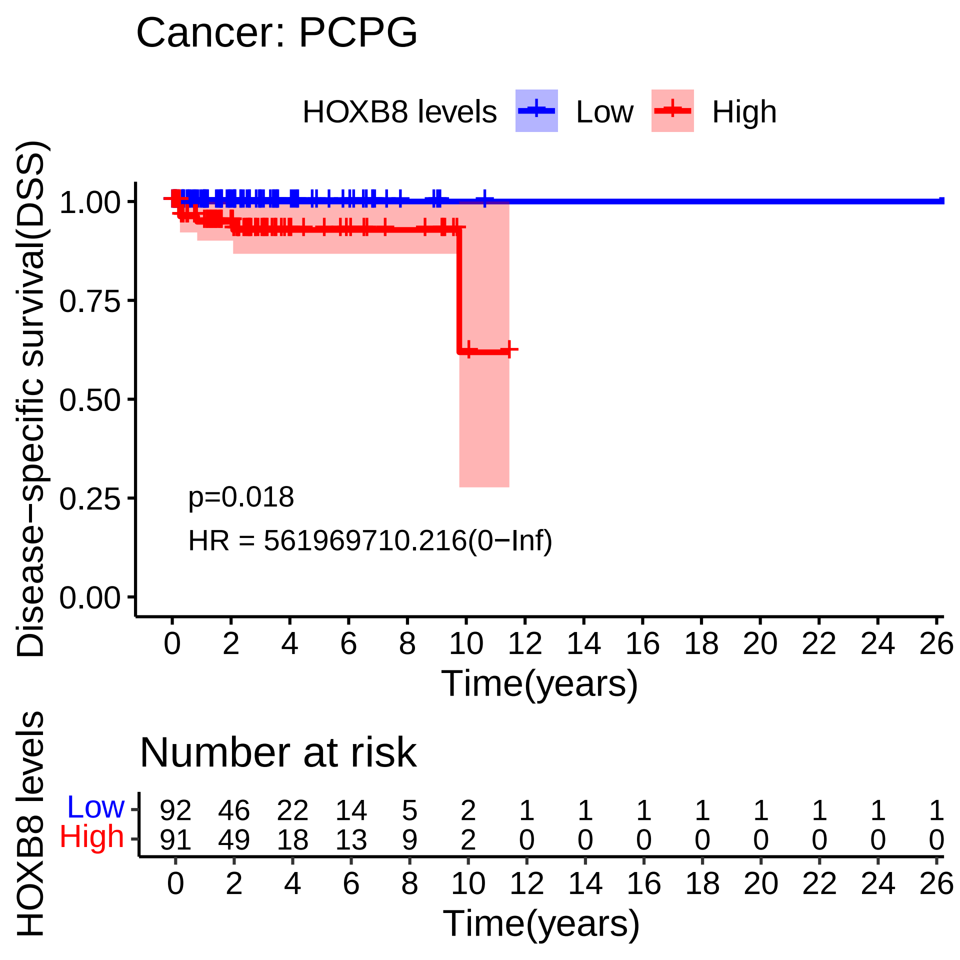

Cancer: PCPG
HOXB8 levels
Low
High
Disease−specific survival(DSS)
1.00
0.75
0.50
0.25
0.00
p=0.018
HR = 561969710.216(0−Inf)
0 2 4 6 8 10 12 14 16 18 20 22 24 26
Time(years)
Number at risk
HOXB8 levels
Low 92 46 22 14 5 2 1 1 1 1 1 1 1 1
High 91 49 18 13 9 2 0 0 0 0 0 0 0 0
0 2 4 6 8 10 12 14 16 18 20 22 24 26
Time(years)

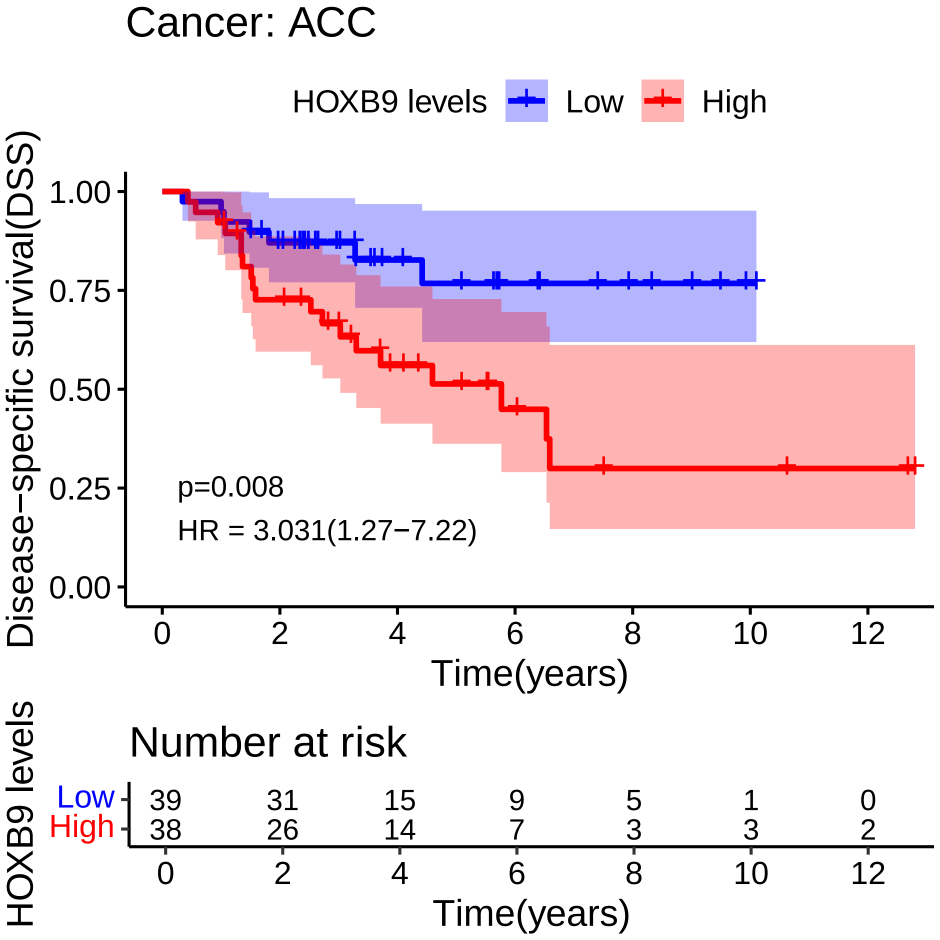

Cancer: ACC
HOXB9 levels
Low
High
Disease−specific survival(DSS)
1.00
0.75
0.50
0.25
0.00
p=0.008
HR = 3.031(1.27−7.22)
0
2
4
6
8
10
12
Time(years)
Number at risk
HOXB9 levels
Low 39 31 15 9 5 1 0
High 38 26 14 7 3 3 2
Time(years)

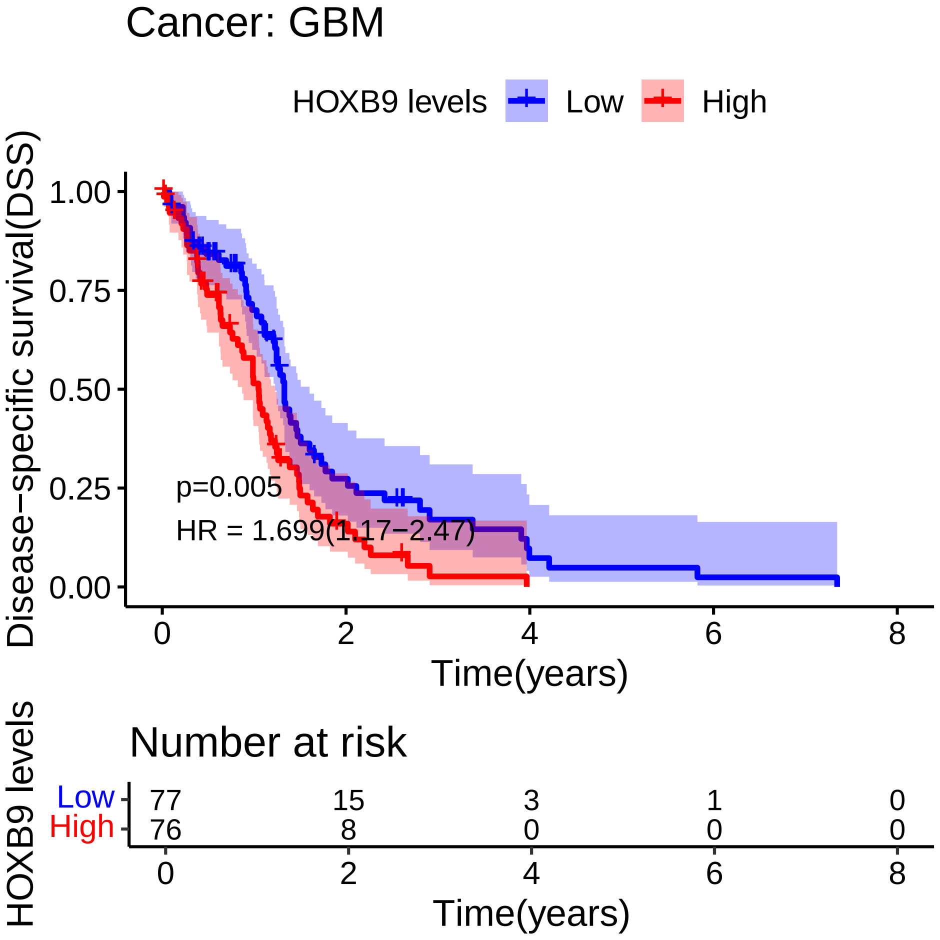

Cancer: GBM
HOXB9 levels
Low
High
Disease−specific survival(DSS)
1.00
0.75
0.50
0.25
0.00
p=0.005
HR = 1.699(1.17−2.47)
0
2
4
6
8
Time(years)
Number at risk
HOXB9 levels
Low 77 15 3 1 0
High 76 8 0 0 0
0
2
4
6
8
Time(years)

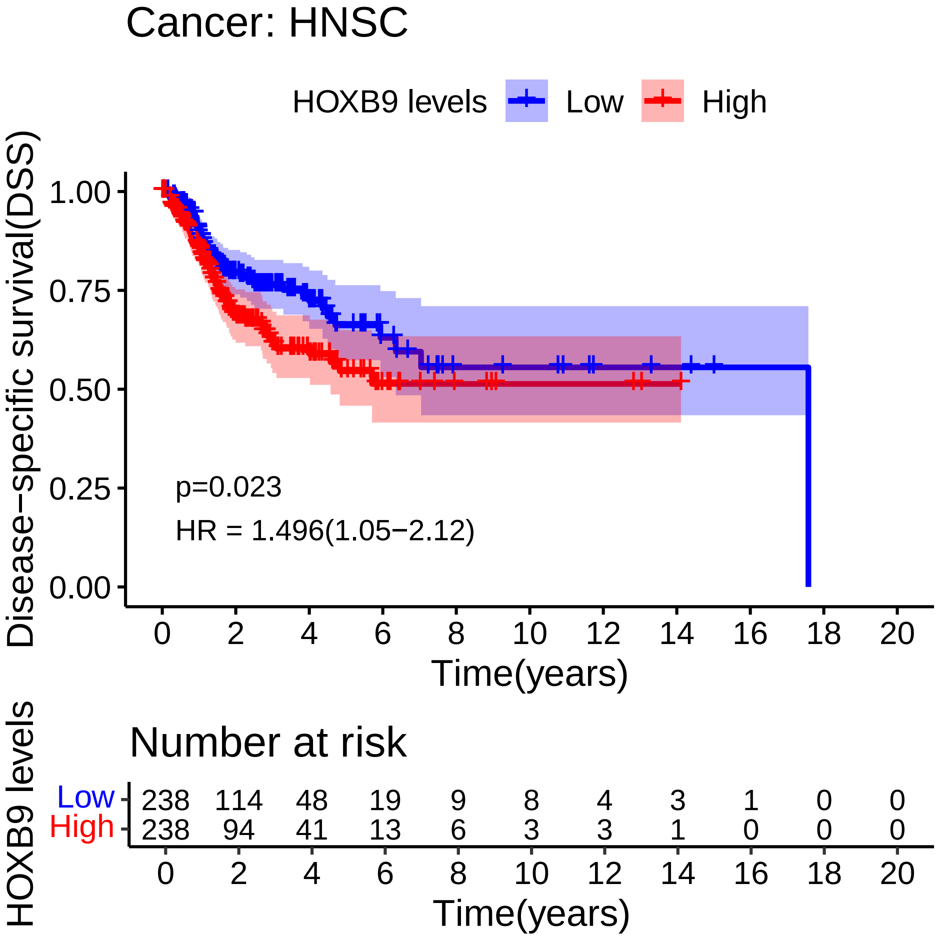

Cancer: HNSC
HOXB9 levels
Low
High
Disease−specific survival(DSS)
1.00
0.75
0.50
0.25
0.00
p=0.023
HR = 1.496(1.05−2.12)
0 2 4 6 8 10 12 14 16 18 20
Time(years)
Number at risk
HOXB9 levels
Low 238 114 48 19 9 8 4 3 1 0 0
High 238 94 41 13 6 3 3 1 0 0 0
0 2 4 6 8 10 12 14 16 18 20
Time(years)

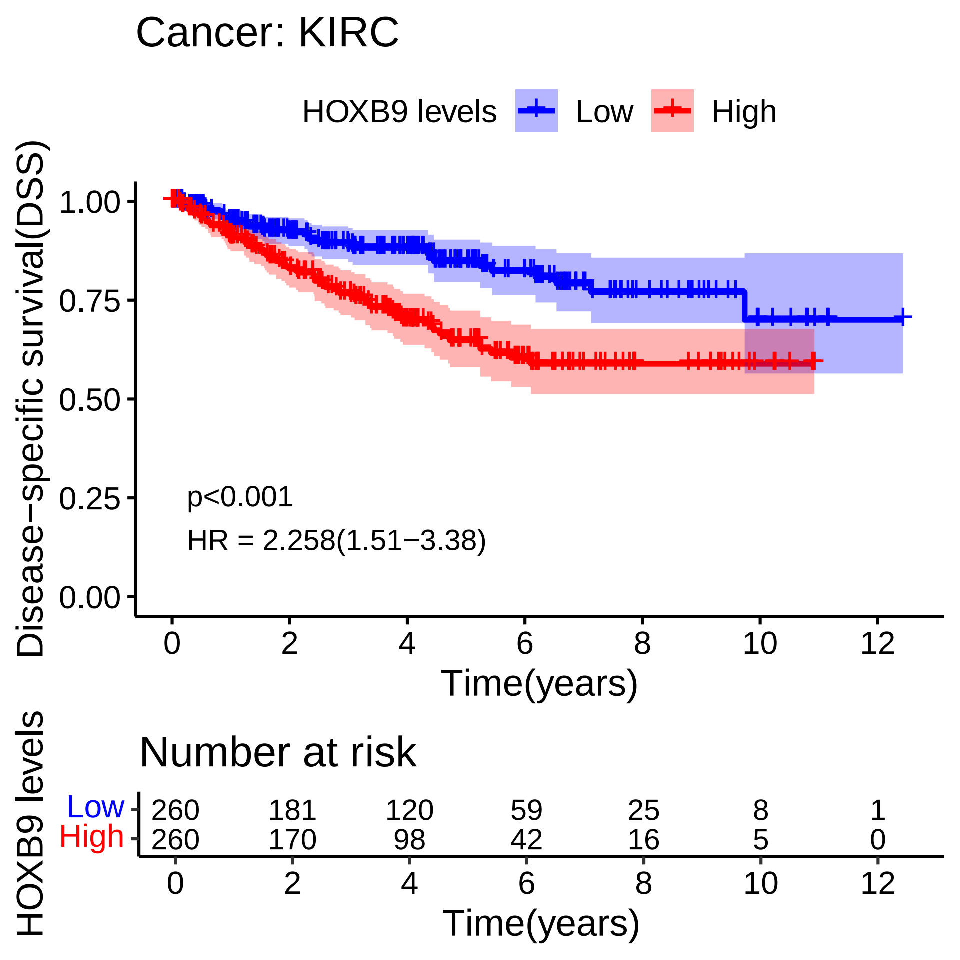

Cancer: KIRC
HOXB9 levels
Low
High
Disease−specific survival(DSS)
1.00
0.75
0.50
0.25
0.00
p<0.001
HR = 2.258(1.51−3.38)
0
2
4
6
8
10
12
Time(years)
Number at risk
HOXB9 levels
Low 260 181 120 59 25 8 1
High 260 170 98 42 16 5 0
0
2
4
6
8
10
12
Time(years)

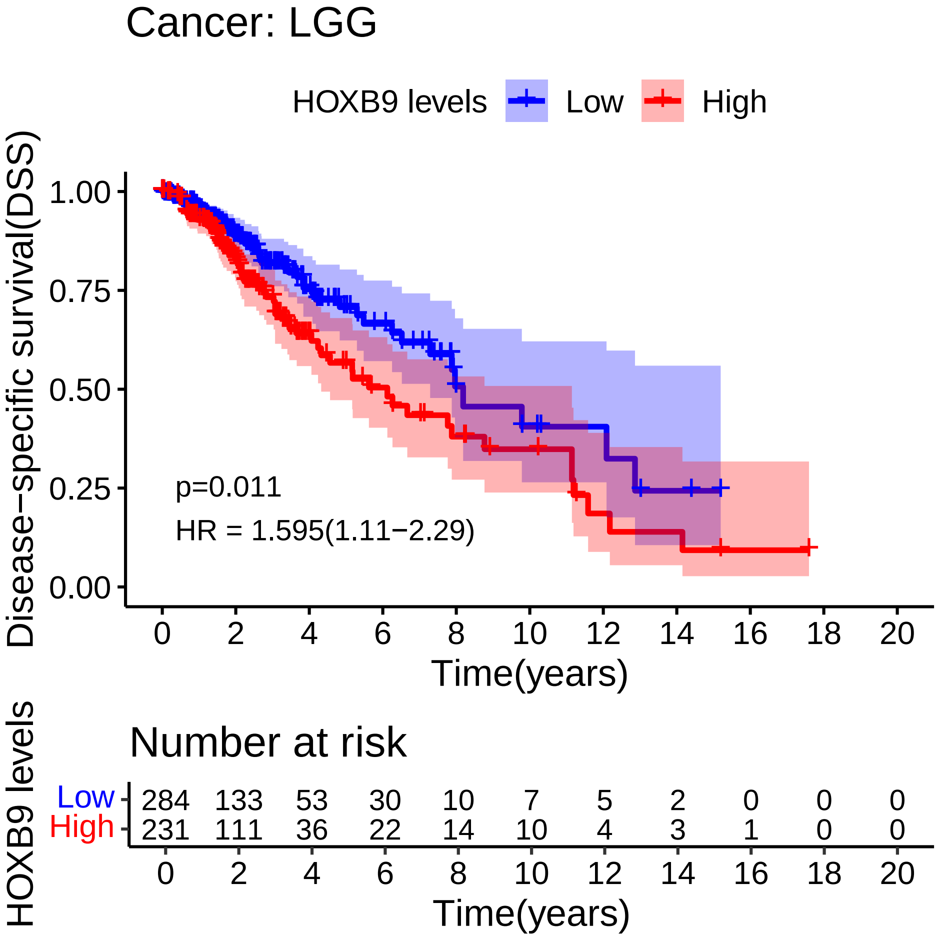

Cancer: LGG
HOXB9 levels
Low
High
Disease−specific survival(DSS)
1.00
0.75
0.50
0.25
0.00
p=0.011
HR = 1.595(1.11−2.29)
0 2 4 6 8 10 12 14 16 18 20
Time(years)
Number at risk
HOXB9 levels
Low 284 133 53 30 10 7 5 2 0 0 0
High 231 111 36 22 14 10 4 3 1 0 0
0 2 4 6 8 10 12 14 16 18 20
Time(years)

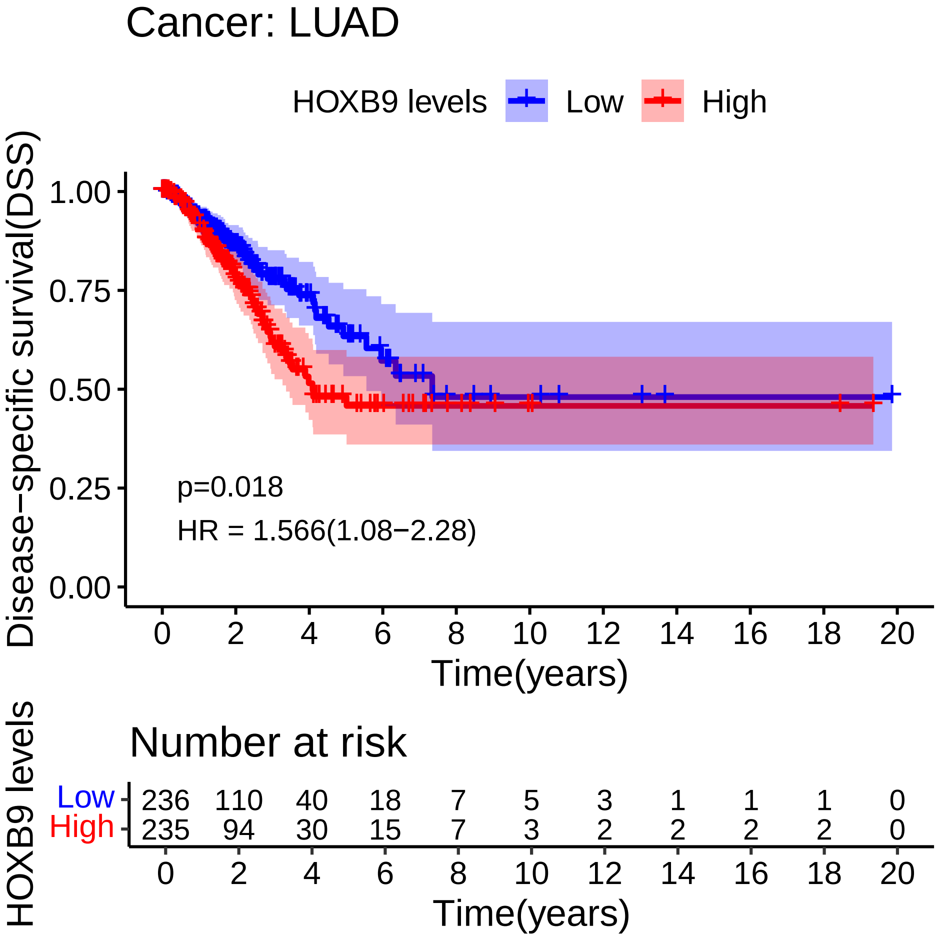

Cancer: LUAD
HOXB9 levels
Low
High
Disease−specific survival(DSS)
1.00
0.75
0.50
0.25
0.00
p=0.018
HR = 1.566(1.08−2.28)
0 2 4 6 8 10 12 14 16 18 20
Time(years)
Number at risk
HOXB9 levels
Low 236 110 40 18 7 5 3 1 1 1 0
High 235 94 30 15 7 3 2 2 2 2 0
0 2 4 6 8 10 12 14 16 18 20
Time(years)

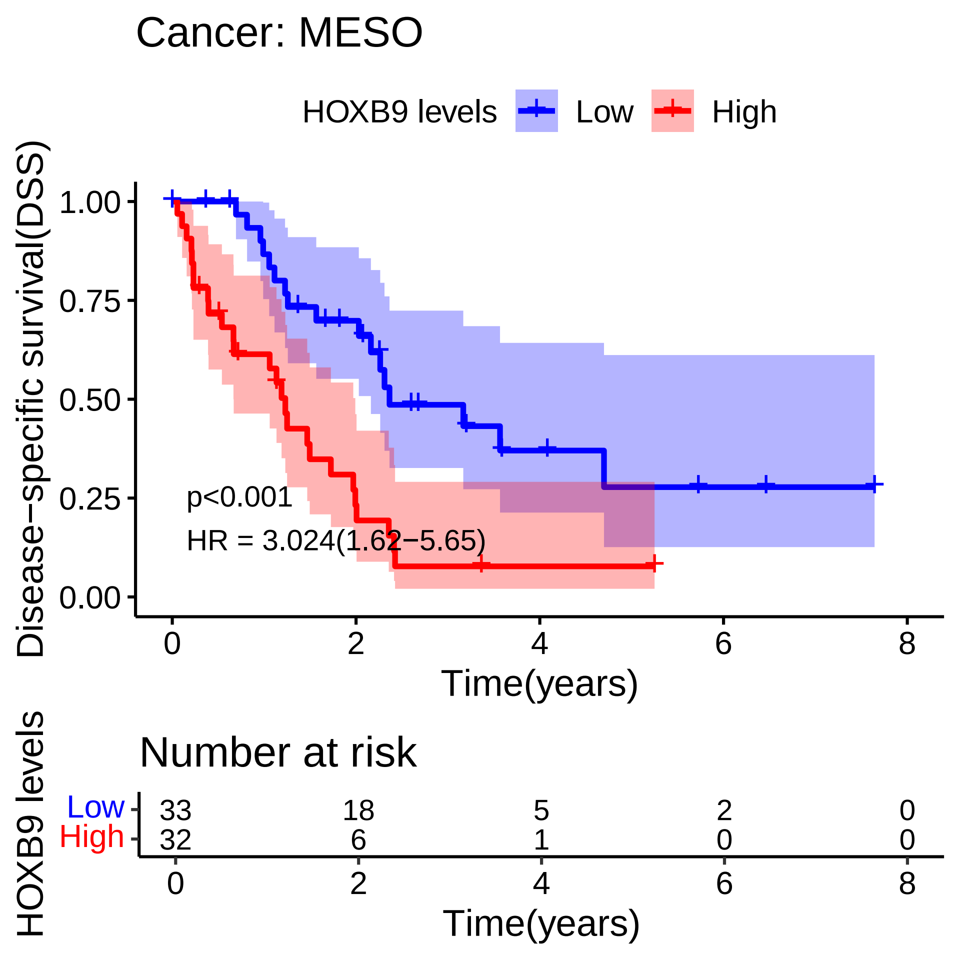

Cancer: MESO
HOXB9 levels
Low
High
Disease−specific survival(DSS)
1.00
0.75
0.50
0.25
0.00
p<0.001
HR = 3.024(1.62−5.65)
0
2
4
6
8
Time(years)
Number at risk
HOXB9 levels
Low 33 18 5 2 0
High 32 6 1 0 0
0
2
4
6
8
Time(years)

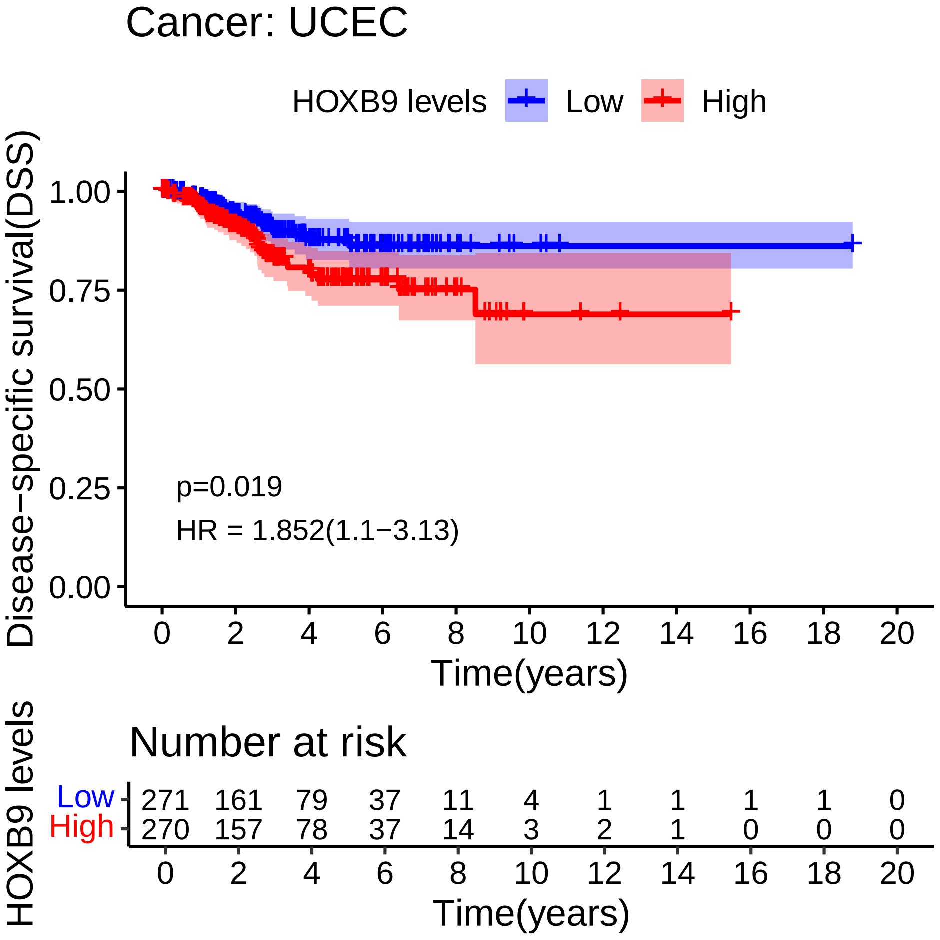

Cancer: UCEC
HOXB9 levels
Low
High
Disease−specific survival(DSS)
1.00
0.75
0.50
0.25
0.00
p=0.019
HR = 1.852(1.1−3.13)
0 2 4 6 8 10 12 14 16 18 20
Time(years)
Number at risk
HOXB9 levels
Low 271 161 79 37 11 4 1 1 1 1 0
High 270 157 78 37 14 3 2 1 0 0 0
0 2 4 6 8 10 12 14 16 18 20
Time(years)

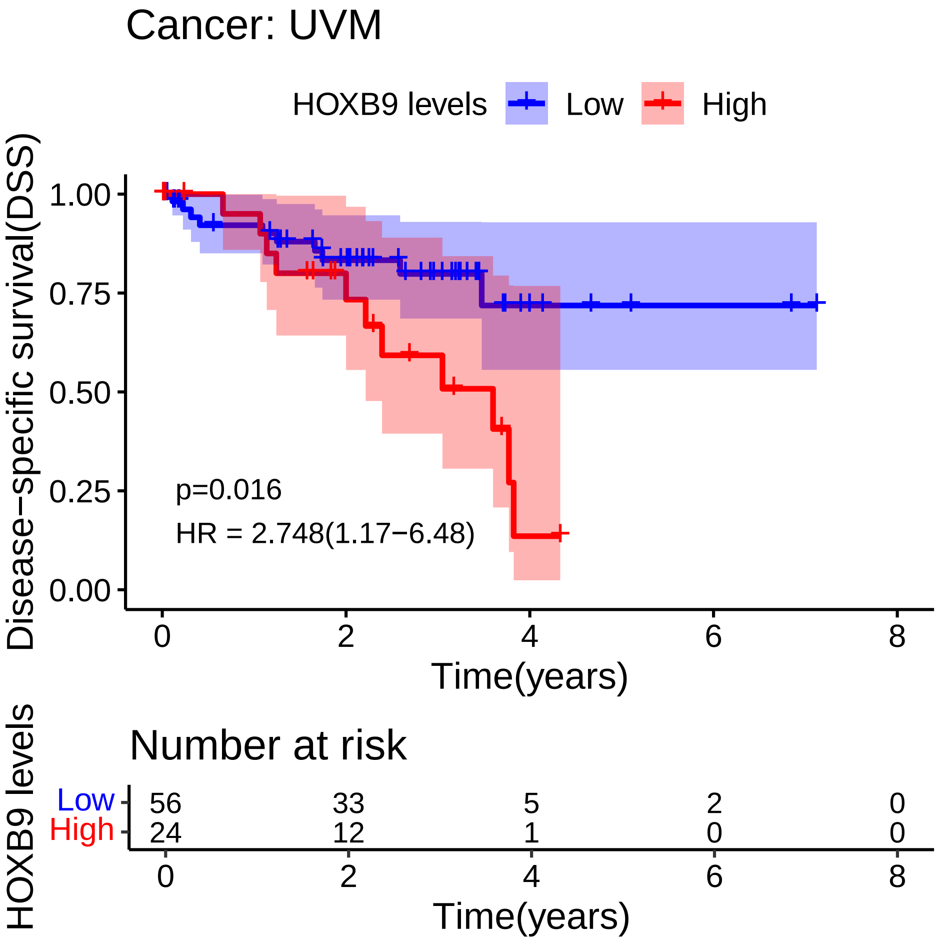

Cancer: UVM
HOXB9 levels
Low
High
Disease−specific survival(DSS)
1.00
0.75
0.50
0.25
0.00
p=0.016
HR = 2.748(1.17−6.48)
0
2
4
6
8
Time(years)
Number at risk
HOXB9 levels
Low 56 33 5 2 0
High 24 12 1 0 0
Time(years)

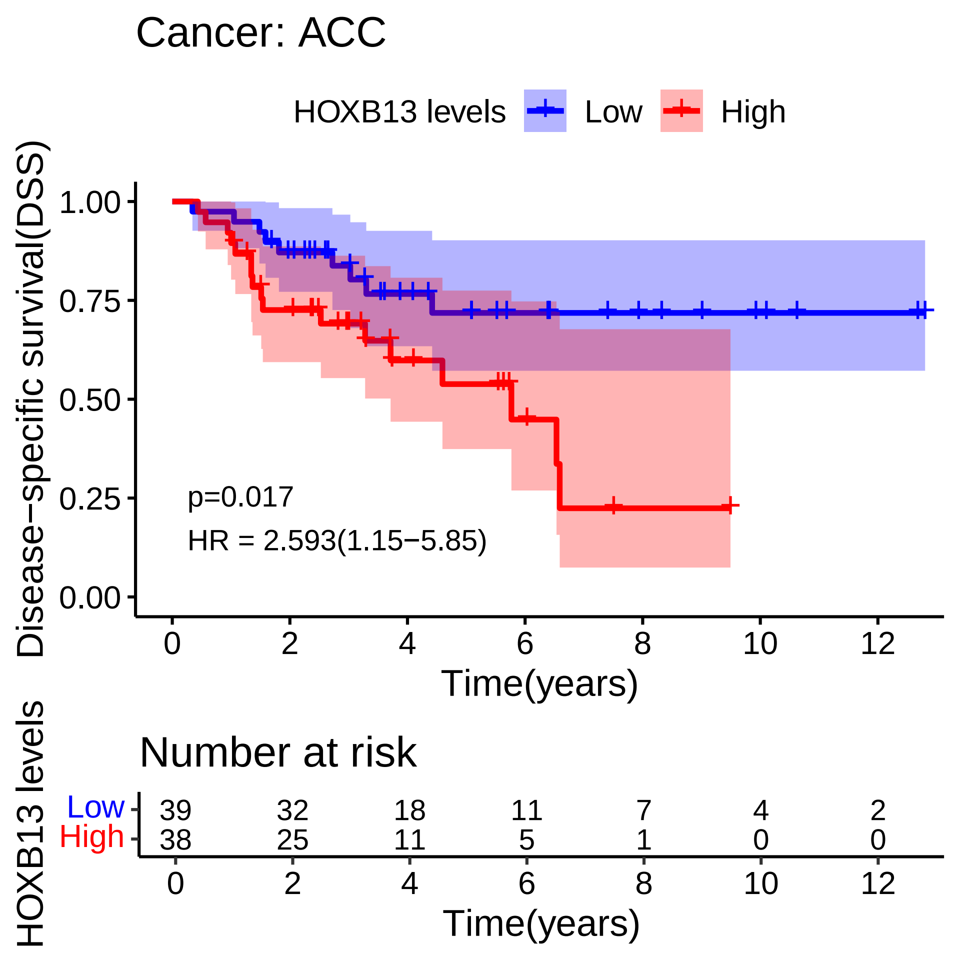

Cancer: ACC
HOXB13 levels
Low
High
Disease−specific survival(DSS)
1.00
0.75
0.50
0.25
0.00
p=0.017
HR = 2.593(1.15−5.85)
0
2
4
6
8
10
12
Time(years)
Number at risk
HOXB13 levels
Low
High
39 32 18 11 7 4 2
38 25 11 5 1 0 0
0
2
4
6
8
10
12
Time(years)

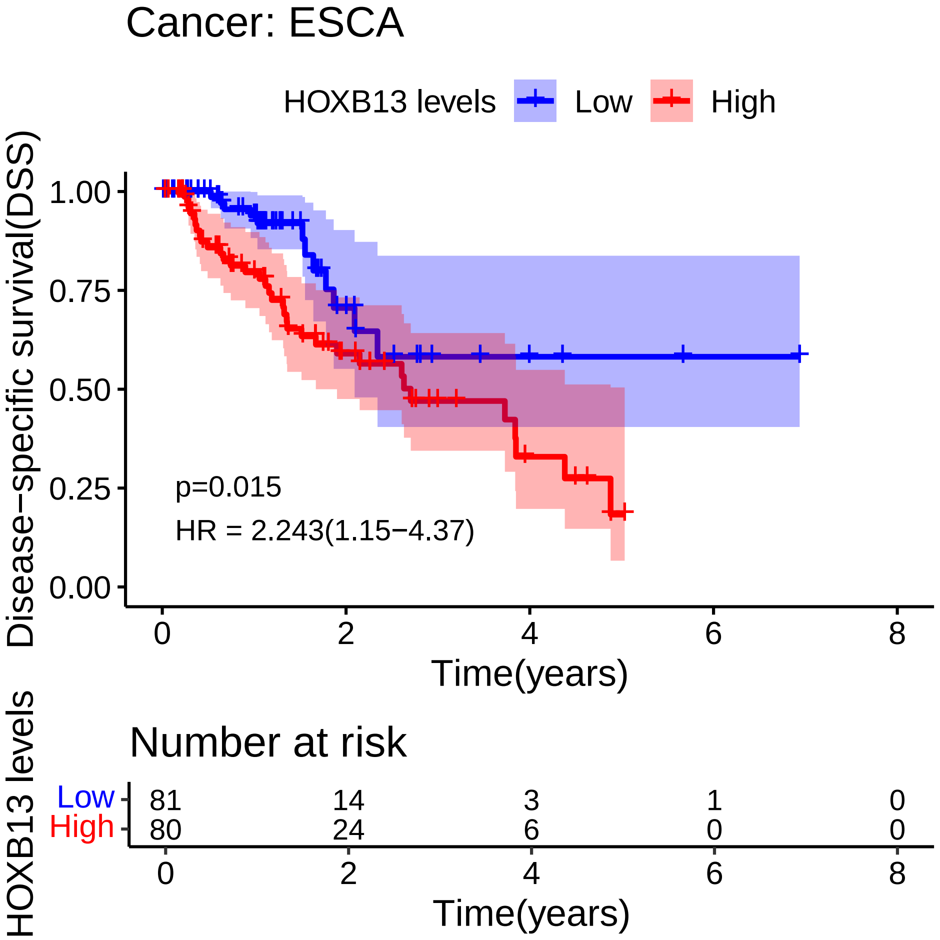
Cancer: ESCA
HOXB13 levels
Low
High
Disease−specific survival(DSS)
1.00
0.75
0.50
0.25
0.00
p=0.015
HR = 2.243(1.15−4.37)
0
2
4
6
8
Time(years)
Number at risk
HOXB13 levels
Low
High
81
14
3
1
0
80
24
6
0
0
0
2
4
6
8
Time(years)

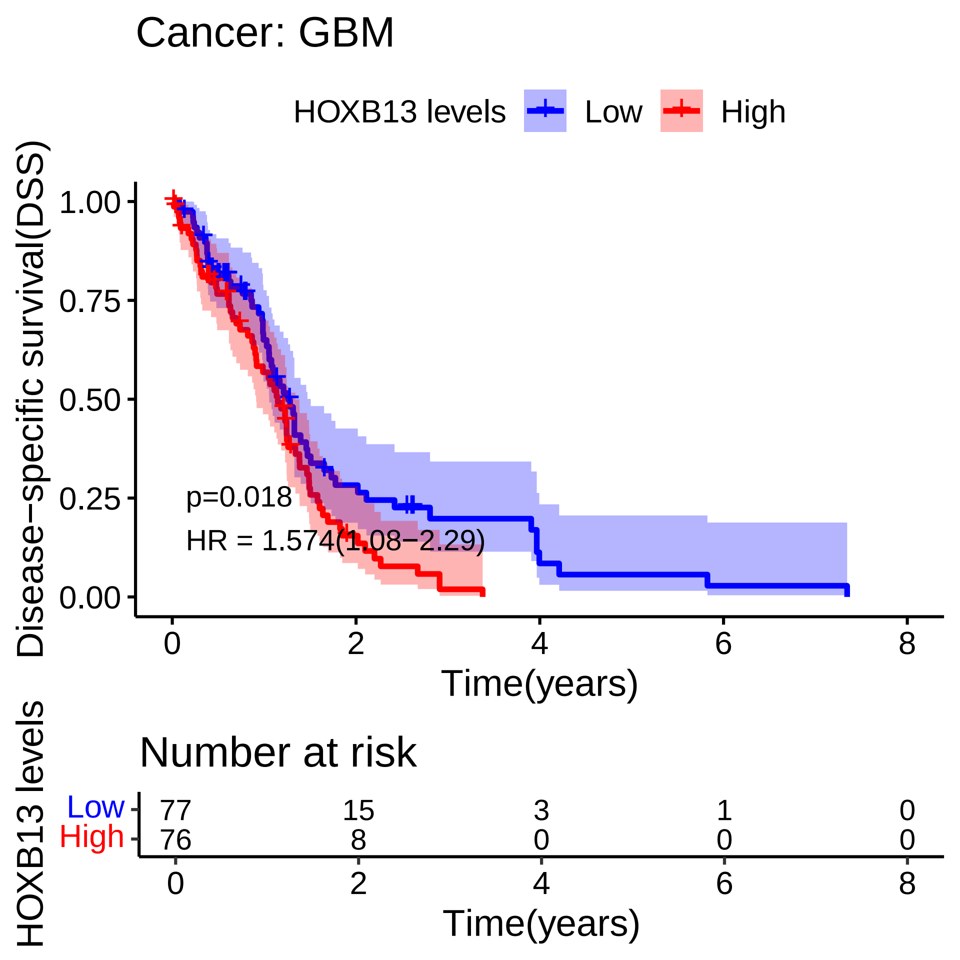

Cancer: GBM
HOXB13 levels
Low
High
Disease−specific survival(DSS)
1.00
0.75
0.50
0.25
0.00
p=0.018
HR = 1.574(1.08−2.29)
0
2
4
6
8
Time(years)
Number at risk
HOXB13 levels
Low 77 15 3 1 0
High 76 8 0 0 0
0
2
4
6
8
Time(years)

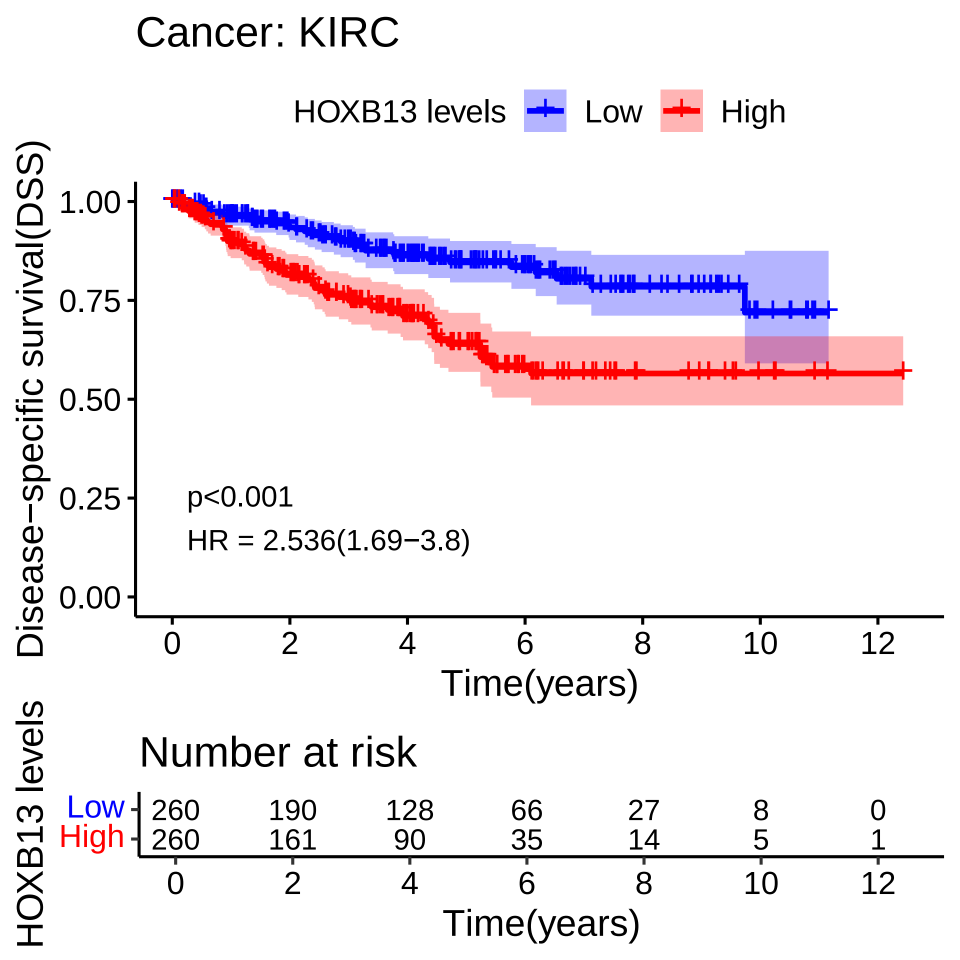

Cancer: KIRC
HOXB13 levels
Low
High
Disease−specific survival(DSS)
1.00
0.75
0.50
0.25
0.00
p<0.001
HR = 2.536(1.69−3.8)
0
2
4
6
8
10
12
Time(years)
Number at risk
HOXB13 levels
Low 260 190 128 66 27 8 0
High 260 161 90 35 14 5 1
Time(years)

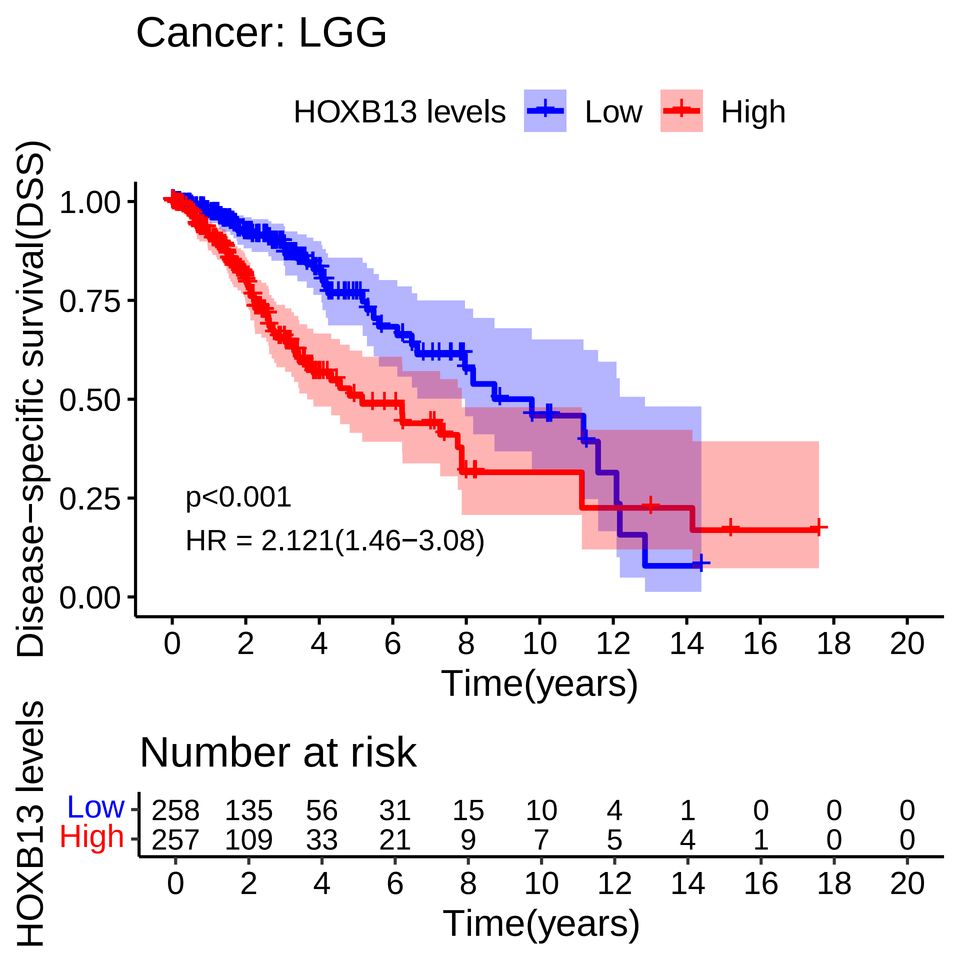

Cancer: LGG
HOXB13 levels
Low
High
Disease−specific survival(DSS)
1.00
0.75
0.50
0.25
0.00
p<0.001
HR = 2.121(1.46−3.08)
0 2 4 6 8 10 12 14 16 18 20
Time(years)
Number at risk
HOXB13 levels
Low 258 135 56 31 15 10 4 1 0 0 0
High 257 109 33 21 9 7 5 4 1 0 0
0 2 4 6 8 10 12 14 16 18 20
Time(years)

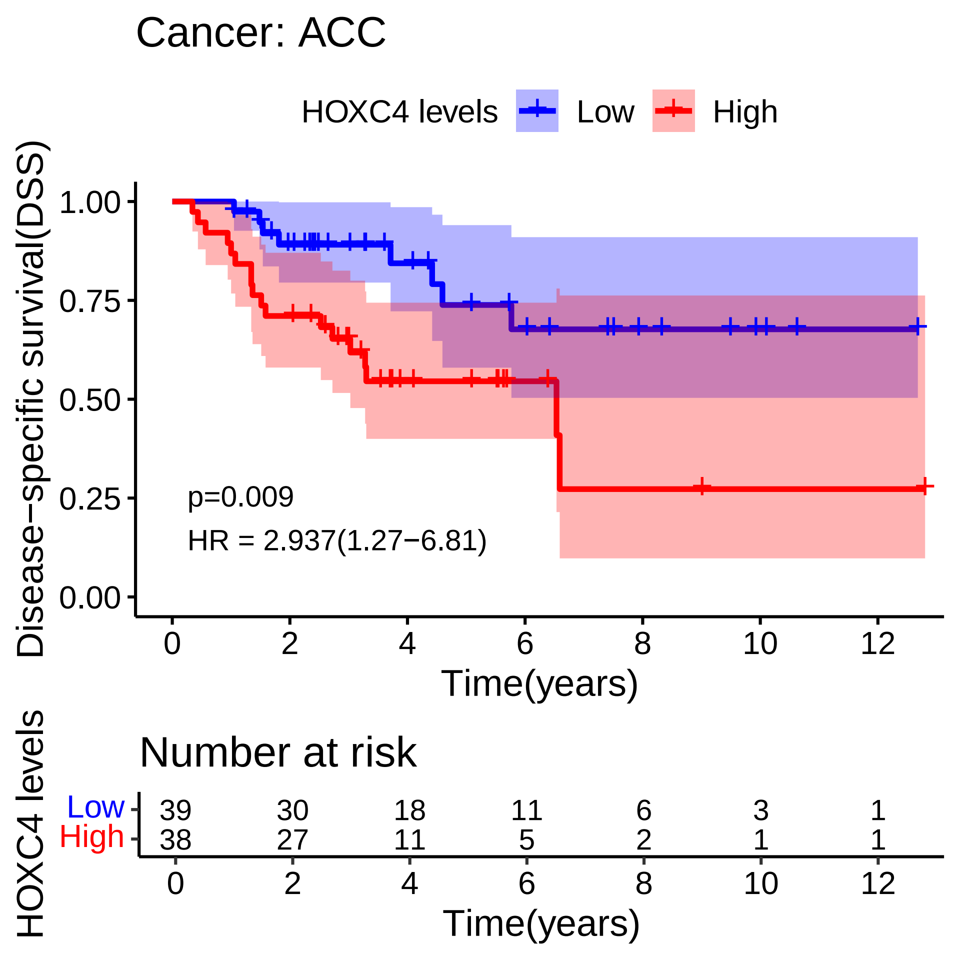

Cancer: ACC
HOXC4 levels
Low
High
Disease−specific survival(DSS)
1.00
0.75
0.50
0.25
0.00
p=0.009
HR = 2.937(1.27−6.81)
0
2
4
6
8
10
12
Time(years)
Number at risk
HOXC4 levels
Low 39 30 18 11 6 3 1
High 38 27 11 5 2 1 1
Time(years)

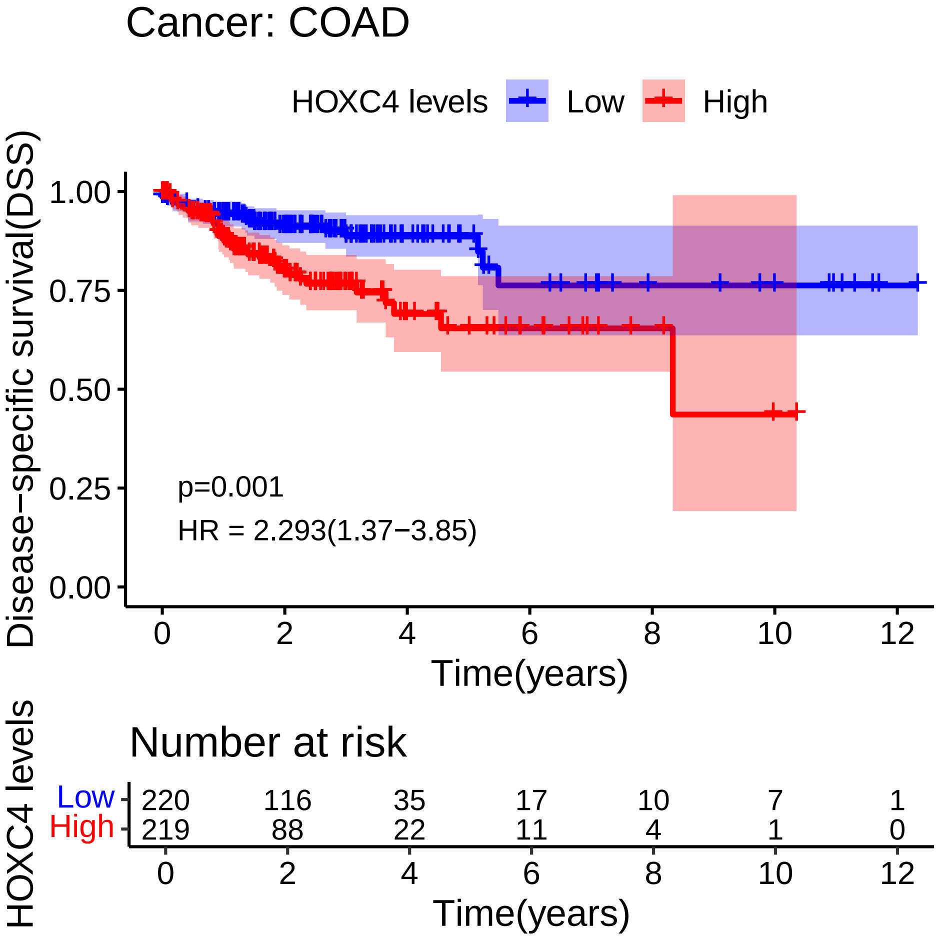

Cancer: COAD
HOXC4 levels
Low
High
Disease−specific survival(DSS)
1.00
0.75
0.50
0.25
0.00
p=0.001
HR = 2.293(1.37−3.85)
0
2
4
6
8
10
12
Time(years)
Number at risk
HOXC4 levels
Low 220 116 35 17 10 7 1
High 219 88 22 11 4 1 0
0
2
4
6
8
10
12
Time(years)

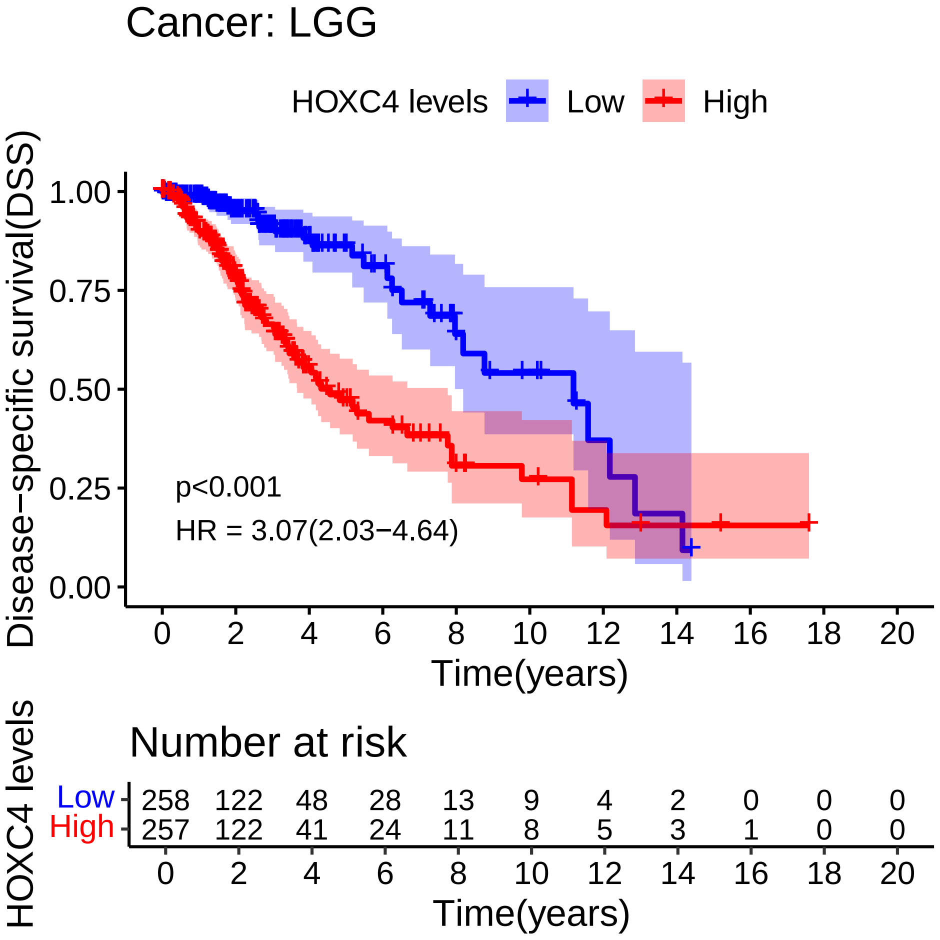

Cancer: LGG
HOXC4 levels
Low
High
Disease−specific survival(DSS)
1.00
0.75
0.50
0.25
0.00
p<0.001
HR = 3.07(2.03−4.64)
0 2 4 6 8 10 12 14 16 18 20
Time(years)
Number at risk
HOXC4 levels
Low 258 122 48 28 13 9 4 2 0 0 0
High 257 122 41 24 11 8 5 3 1 0 0
0 2 4 6 8 10 12 14 16 18 20
Time(years)

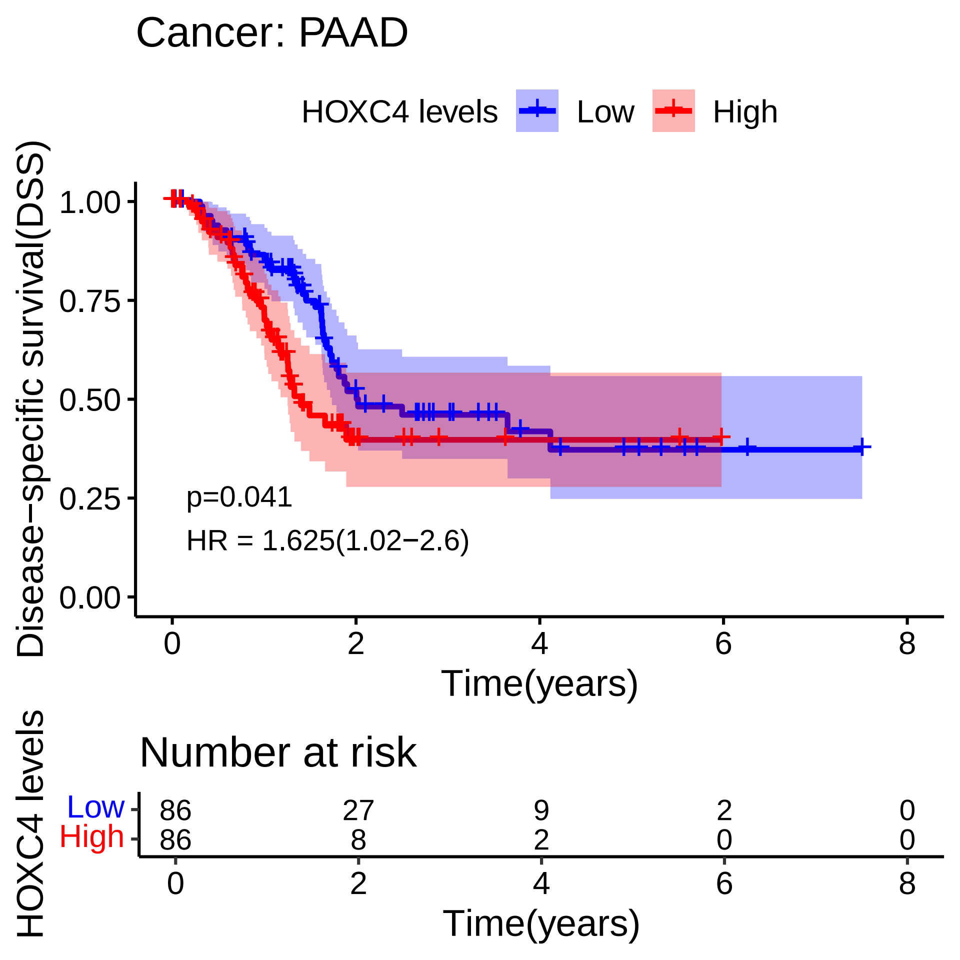
Cancer: PAAD
HOXC4 levels
Low
High
Disease−specific survival(DSS)
1.00
0.75
0.50
0.25
0.00
p=0.041
HR = 1.625(1.02−2.6)
0
2
4
6
8
Time(years)
Number at risk
HOXC4 levels
Low
High
86 27 9 2 0
86 8 2 0 0
0
2
4
6
8
Time(years)

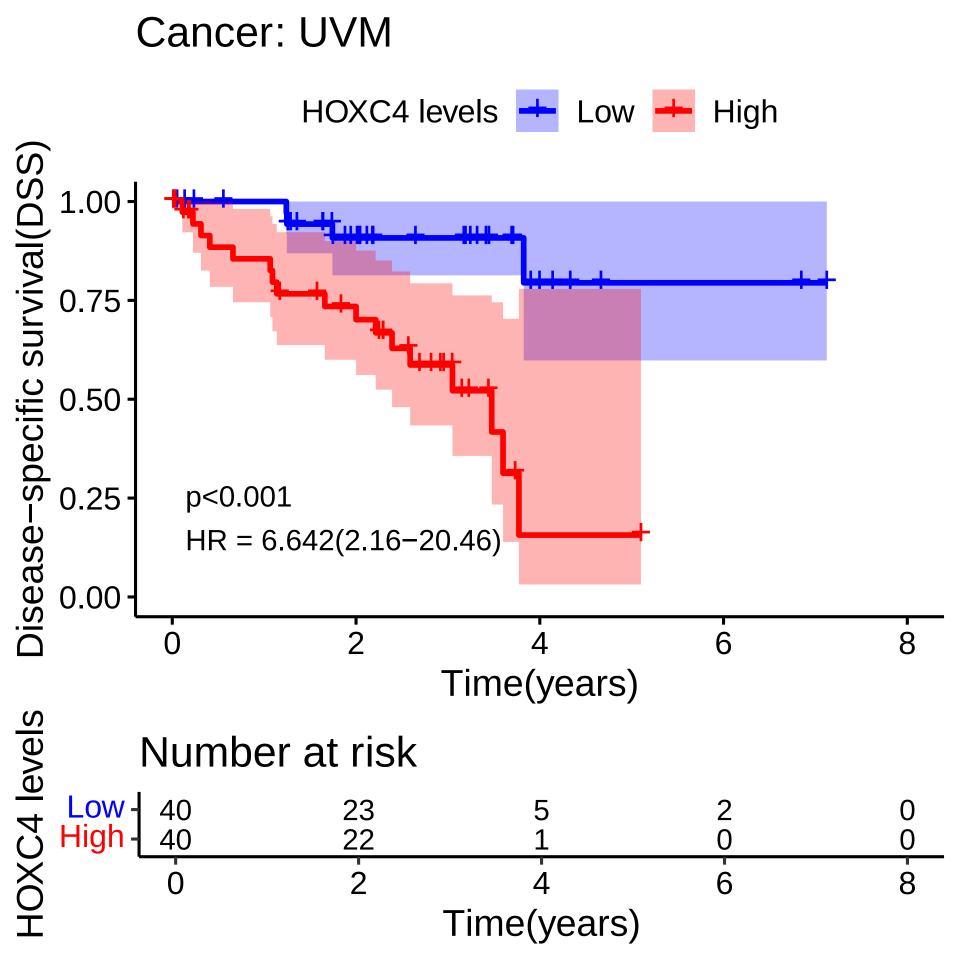

Cancer: UVM
HOXC4 levels
Low
High
Disease−specific survival(DSS)
1.00
0.75
0.50
0.25
0.00
p<0.001
HR = 6.642(2.16−20.46)
0
2
4
6
8
Time(years)
Number at risk
HOXC4 levels
Low
High
40 23 5 2 0
40 22 1 0 0
0
2
4
6
8
Time(years)

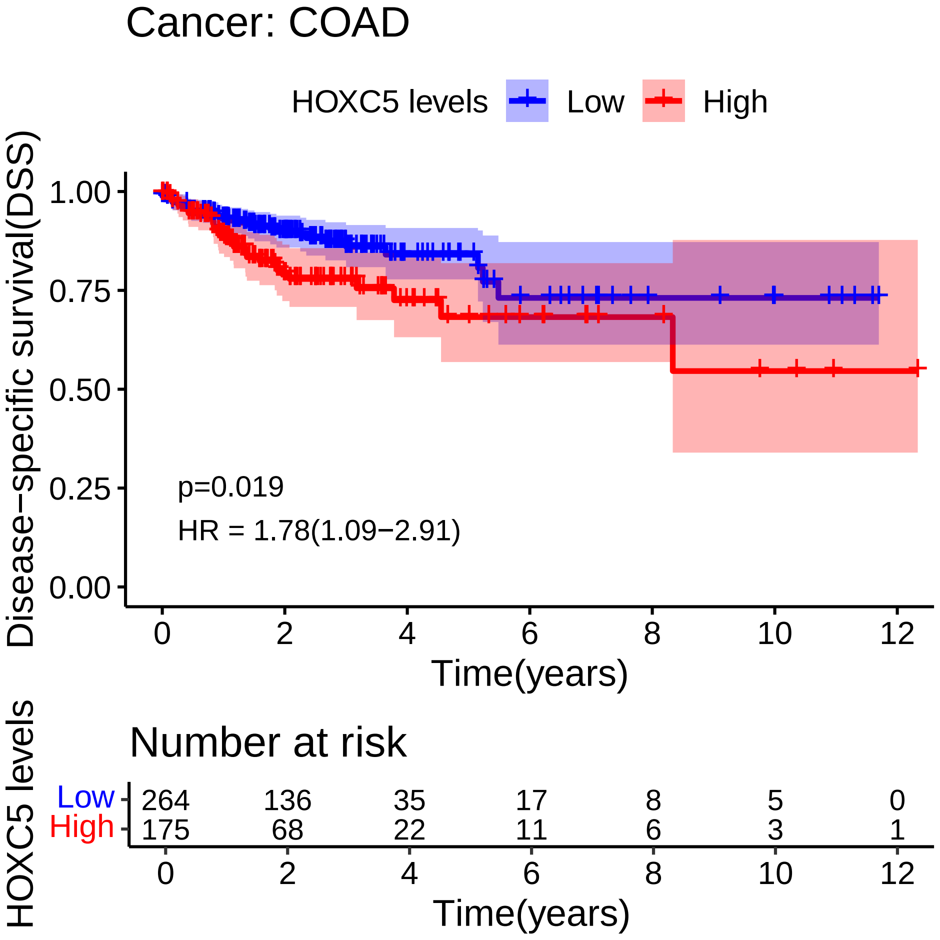

Cancer: COAD
HOXC5 levels
Low
High
Disease−specific survival(DSS)
1.00
0.75
0.50
0.25
0.00
p=0.019
HR = 1.78(1.09−2.91)
0
2
4
6
8
10
12
Time(years)
Number at risk
HOXC5 levels
Low 264 136 35 17 8 5 0
High 175 68 22 11 6 3 1
Time(years)

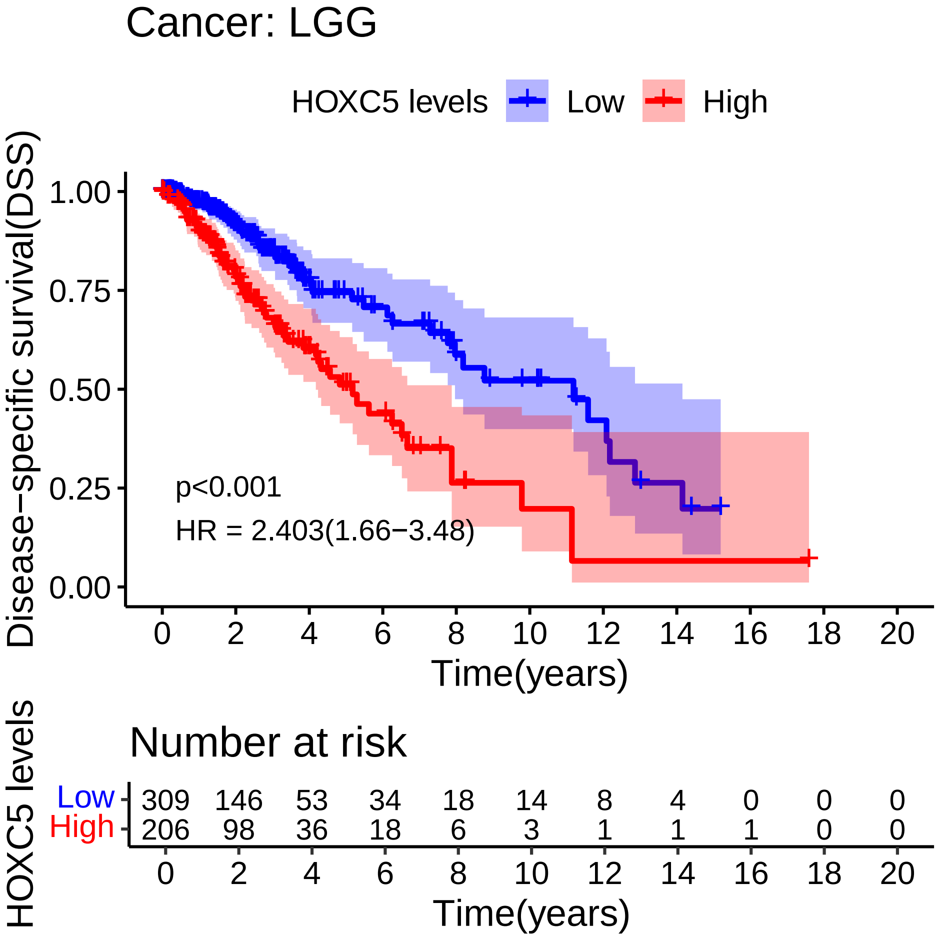

Cancer: LGG
HOXC5 levels
Low
High
Disease−specific survival(DSS)
1.00
0.75
0.50
0.25
0.00
p<0.001
HR = 2.403(1.66−3.48)
0 2 4 6 8 10 12 14 16 18 20
Time(years)
Number at risk
HOXC5 levels
Low 309 146 53 34 18 14 8 4 0 0 0
High 206 98 36 18 6 3 1 1 1 0 0
0 2 4 6 8 10 12 14 16 18 20
Time(years)

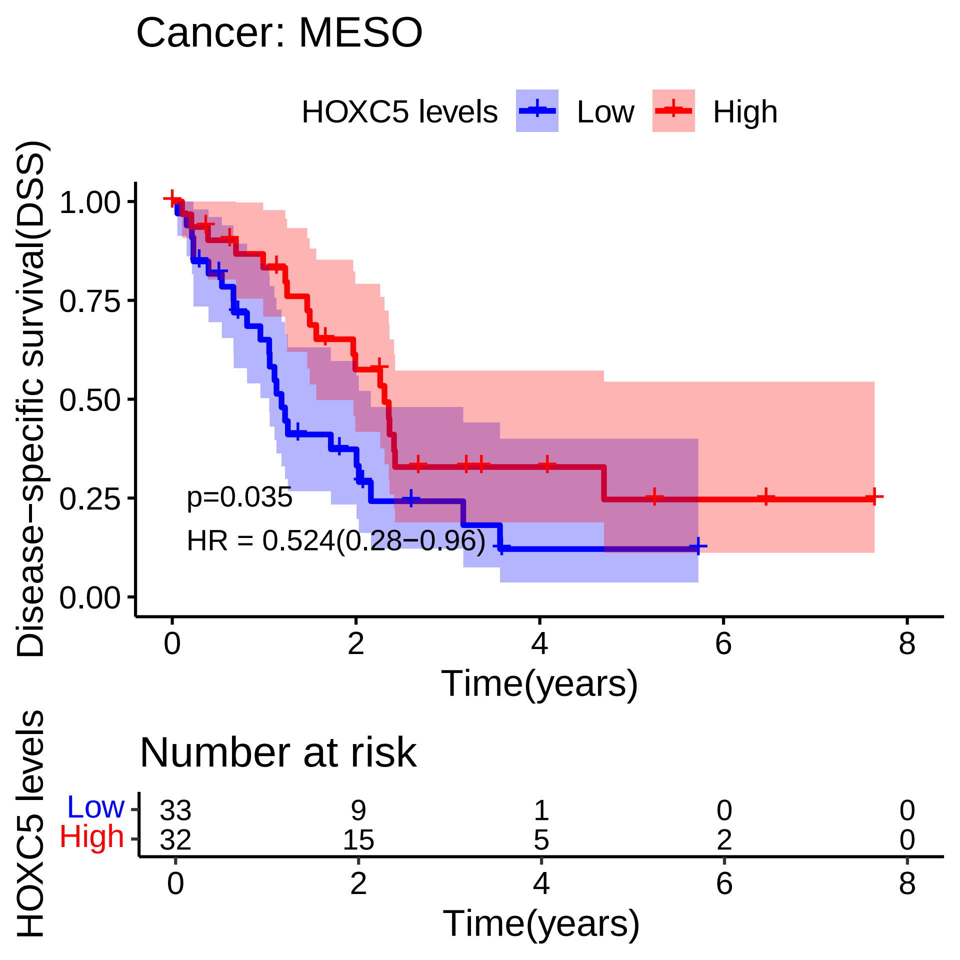

Cancer: MESO
HOXC5 levels
Low
High
Disease−specific survival(DSS)
1.00
0.75
0.50
0.25
0.00
p=0.035
HR = 0.524(0.28−0.96)
0
2
4
6
8
Time(years)
Number at risk
HOXC5 levels
Low 33 9 1 0 0
High 32 15 5 2 0
0
2
4
6
8
Time(years)

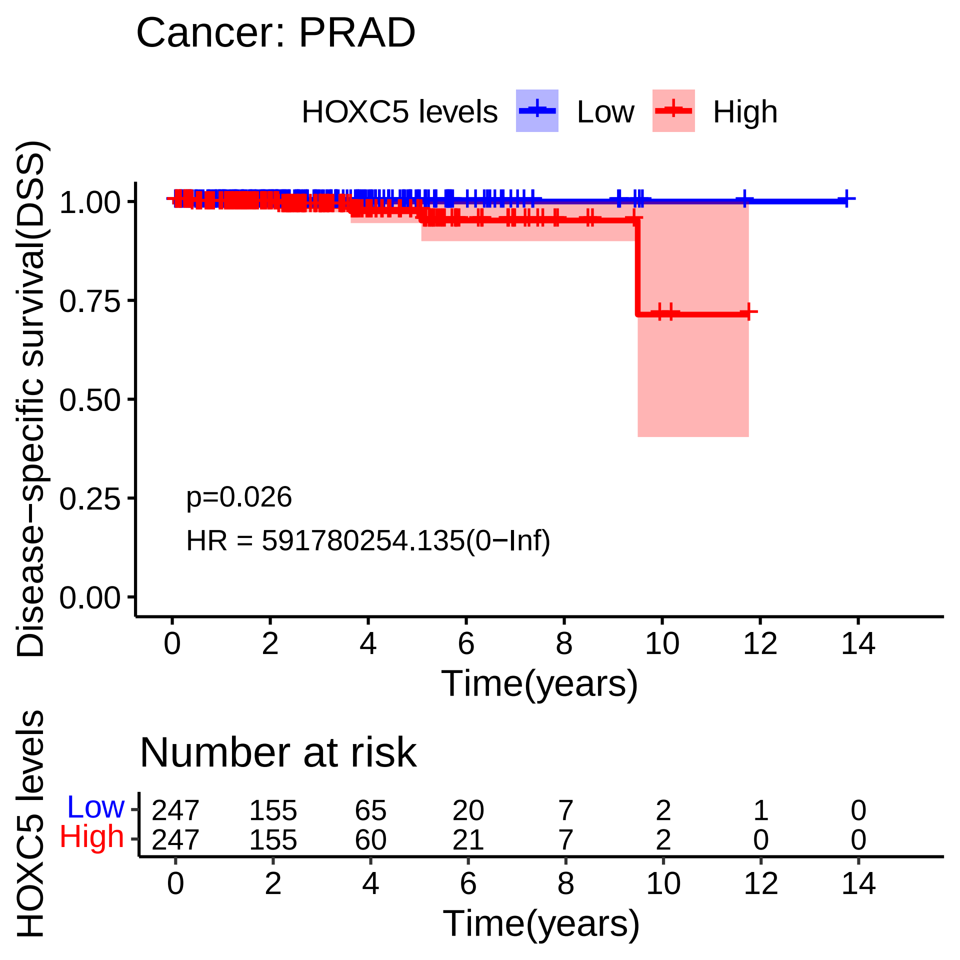

Cancer: PRAD
HOXC5 levels
Low
High
Disease−specific survival(DSS)
1.00
0.75
0.50
0.25
0.00
p=0.026
HR = 591780254.135(0−Inf)
0
2
4
6
8
10
12
14
Time(years)
Number at risk
HOXC5 levels
Low 247 155 65 20 7 2 1 0
High 247 155 60 21 7 2 0 0
Time(years)

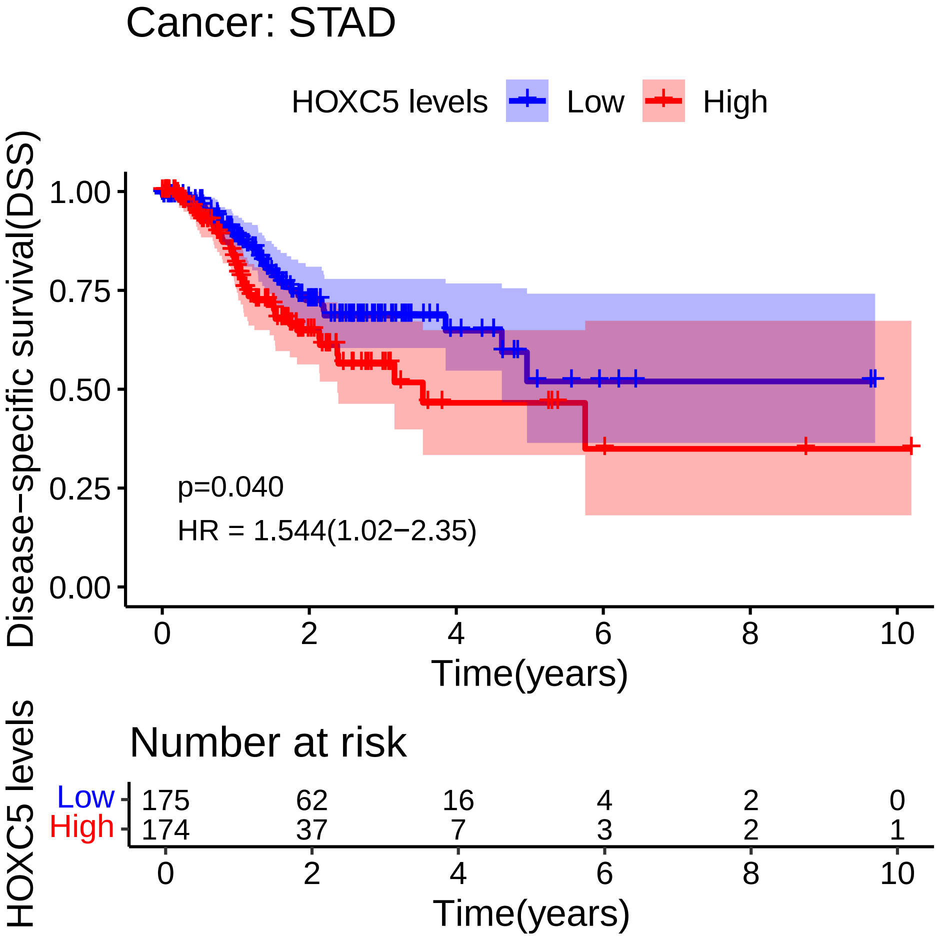

Cancer: STAD
HOXC5 levels
Low
High
Disease−specific survival(DSS)
1.00
0.75
0.50
0.25
0.00
p=0.040
HR = 1.544(1.02−2.35)
0
2
4
6
8
10
Time(years)
Number at risk
HOXC5 levels
Low
High
175 62 16 4 2 0
174 37 7 3 2 1
Time(years)

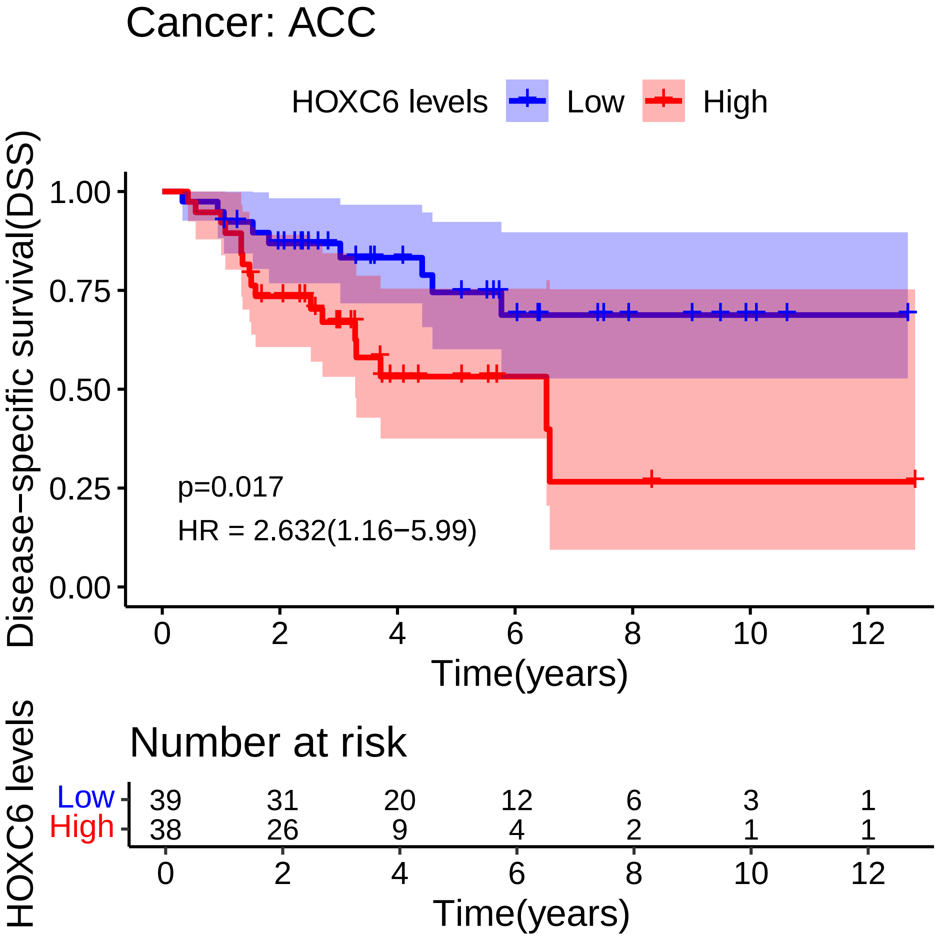

Cancer: ACC
HOXC6 levels
Low
High
Disease−specific survival(DSS)
1.00
0.75
0.50
0.25
0.00
p=0.017
HR = 2.632(1.16−5.99)
0
2
4
6
8
10
12
Time(years)
Number at risk
HOXC6 levels
Low 39 31 20 12 6 3 1
High 38 26 9 4 2 1 1
0
2
4
6
8
10
12
Time(years)

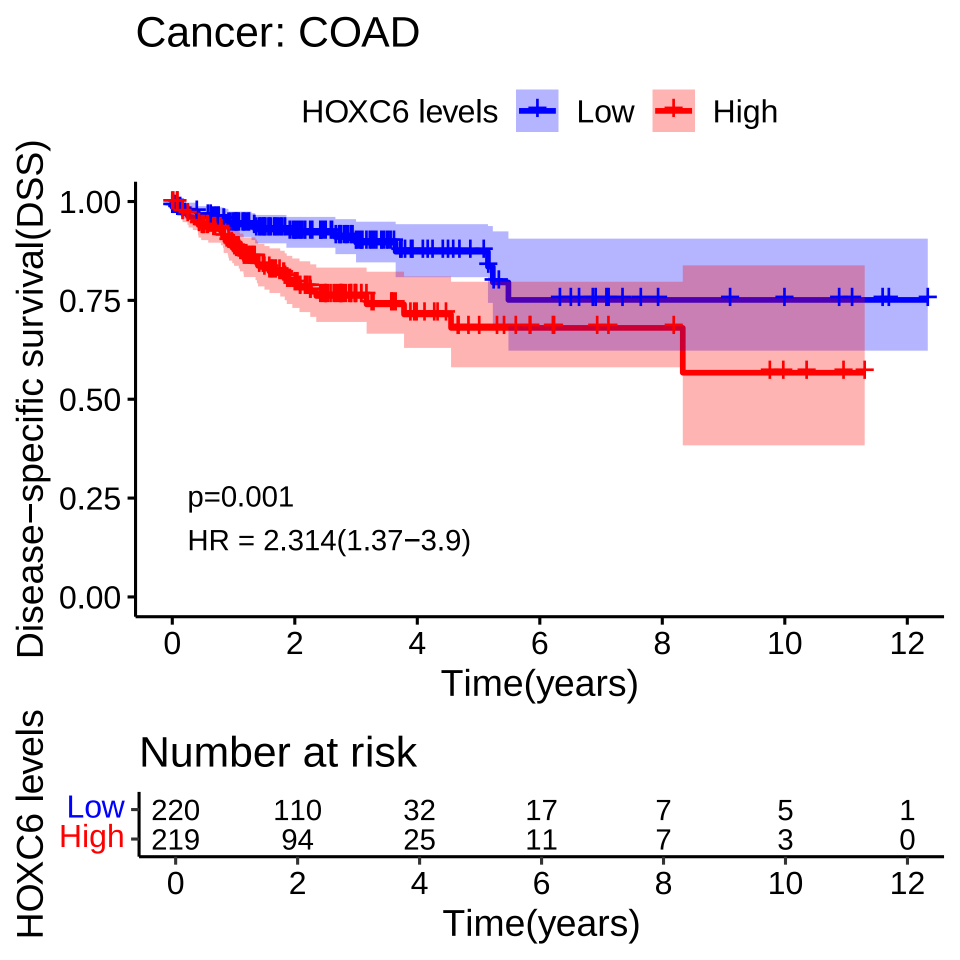
Cancer: COAD
HOXC6 levels
Low
High
Disease−specific survival(DSS)
1.00
0.75
0.50
0.25
0.00
p=0.001
HR = 2.314(1.37−3.9)
0
2
4
6
8
10
12
Time(years)
Number at risk
HOXC6 levels
Low 220 110 32 17 7 5 1
High 219 94 25 11 7 3 0
Time(years)

Cancer: GBM

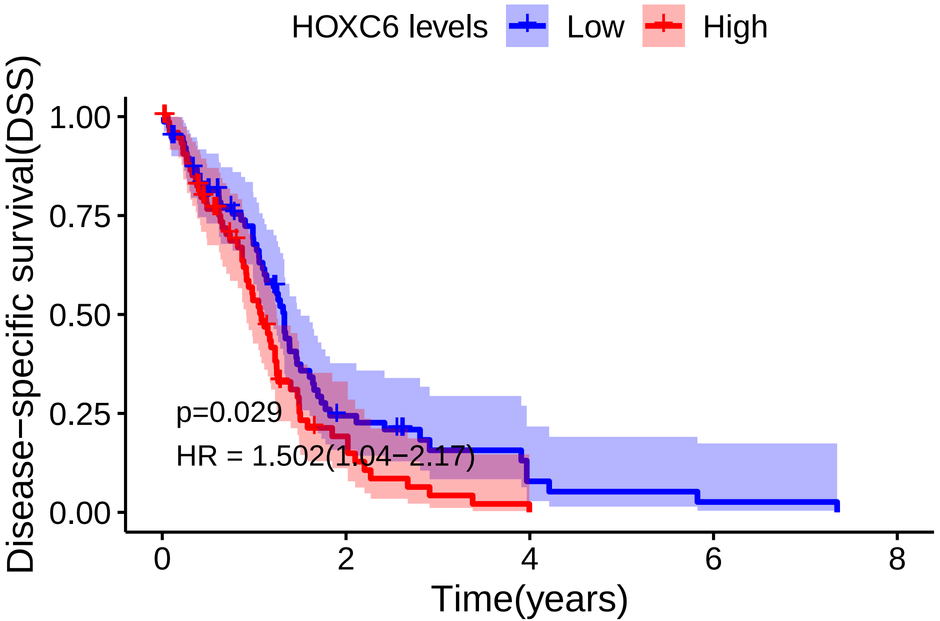

Number at risk

HOXC6 levels	0	2	4	6	8
Low	77	14	3	1	0
High	76	9	0	0	0

Time(years)

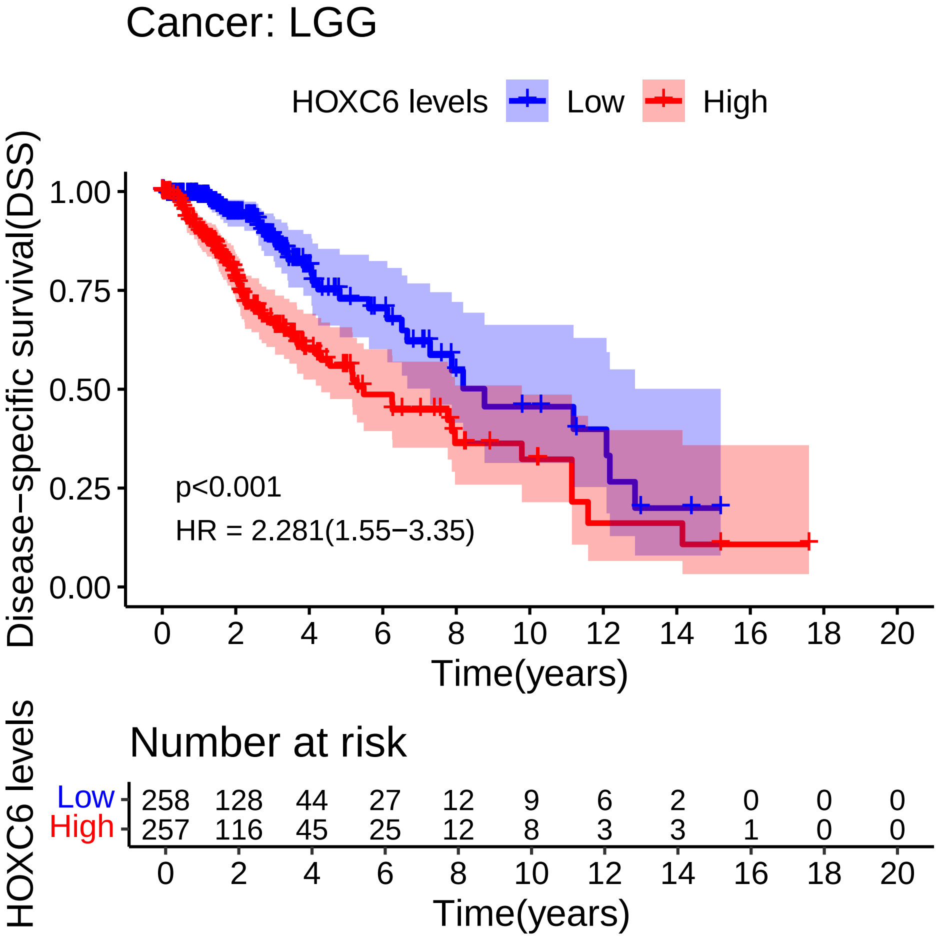

Cancer: LGG
HOXC6 levels
Low
High
Disease−specific survival(DSS)
1.00
0.75
0.50
0.25
0.00
p<0.001
HR = 2.281(1.55−3.35)
0 2 4 6 8 10 12 14 16 18 20
Time(years)
Number at risk
HOXC6 levels
Low 258 128 44 27 12 9 6 2 0 0 0
High 257 116 45 25 12 8 3 3 1 0 0
0 2 4 6 8 10 12 14 16 18 20
Time(years)

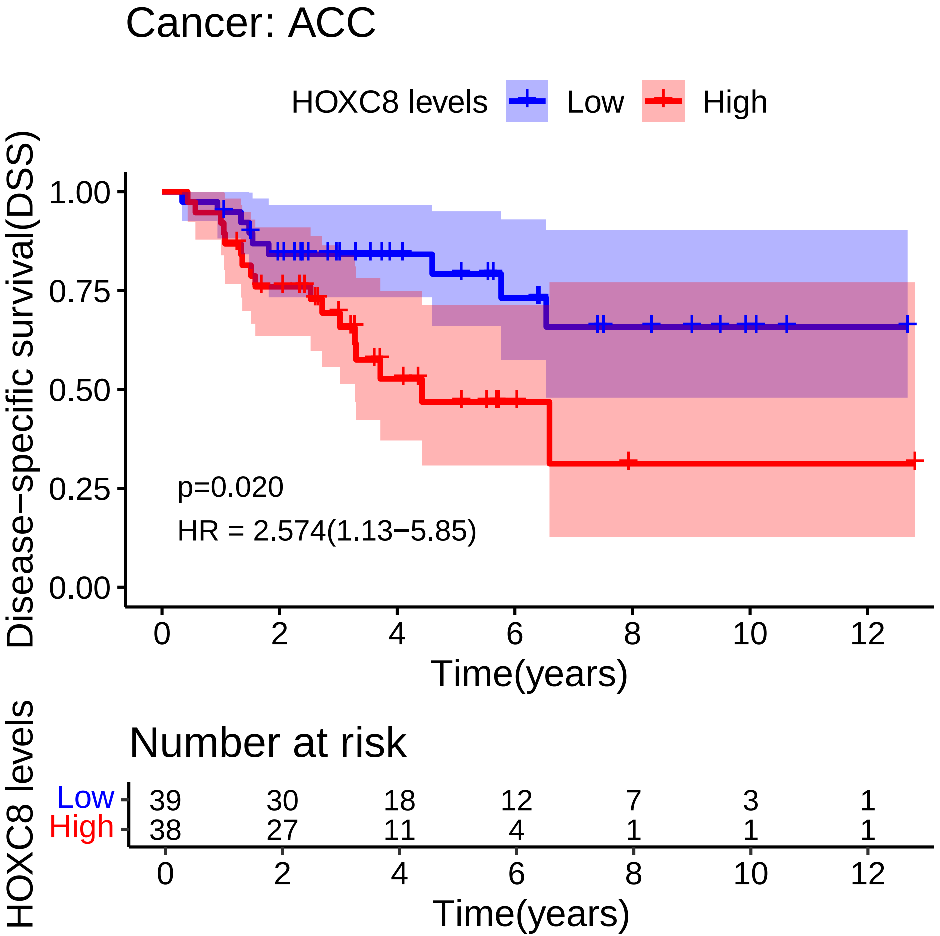

Cancer: ACC
HOXC8 levels
Low
High
Disease−specific survival(DSS)
1.00
0.75
0.50
0.25
0.00
p=0.020
HR = 2.574(1.13−5.85)
0
2
4
6
8
10
12
Time(years)
Number at risk
HOXC8 levels
Low 39 30 18 12 7 3 1
High 38 27 11 4 1 1 1
Time(years)

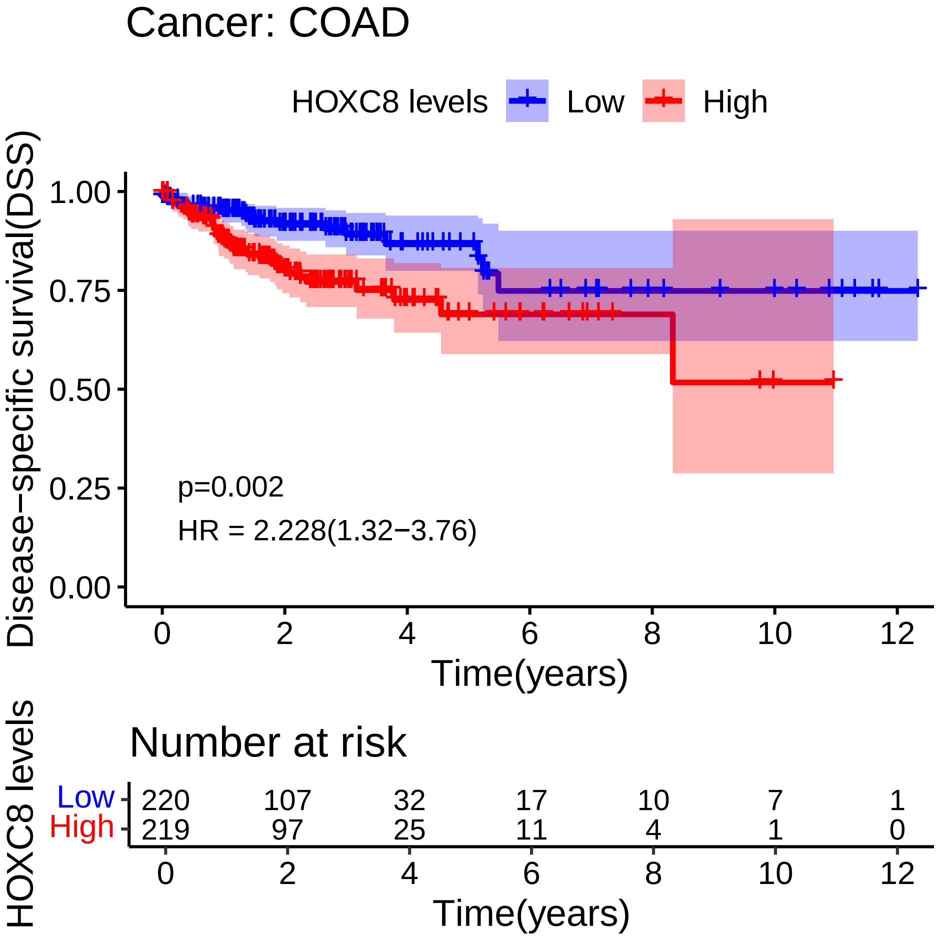
Cancer: COAD
HOXC8 levels
Low
High
Disease−specific survival(DSS)
1.00
0.75
0.50
0.25
0.00
p=0.002
HR = 2.228(1.32−3.76)
0
2
4
6
8
10
12
Time(years)
Number at risk
HOXC8 levels
Low 220 107 32 17 10 7 1
High 219 97 25 11 4 1 0
Time(years)

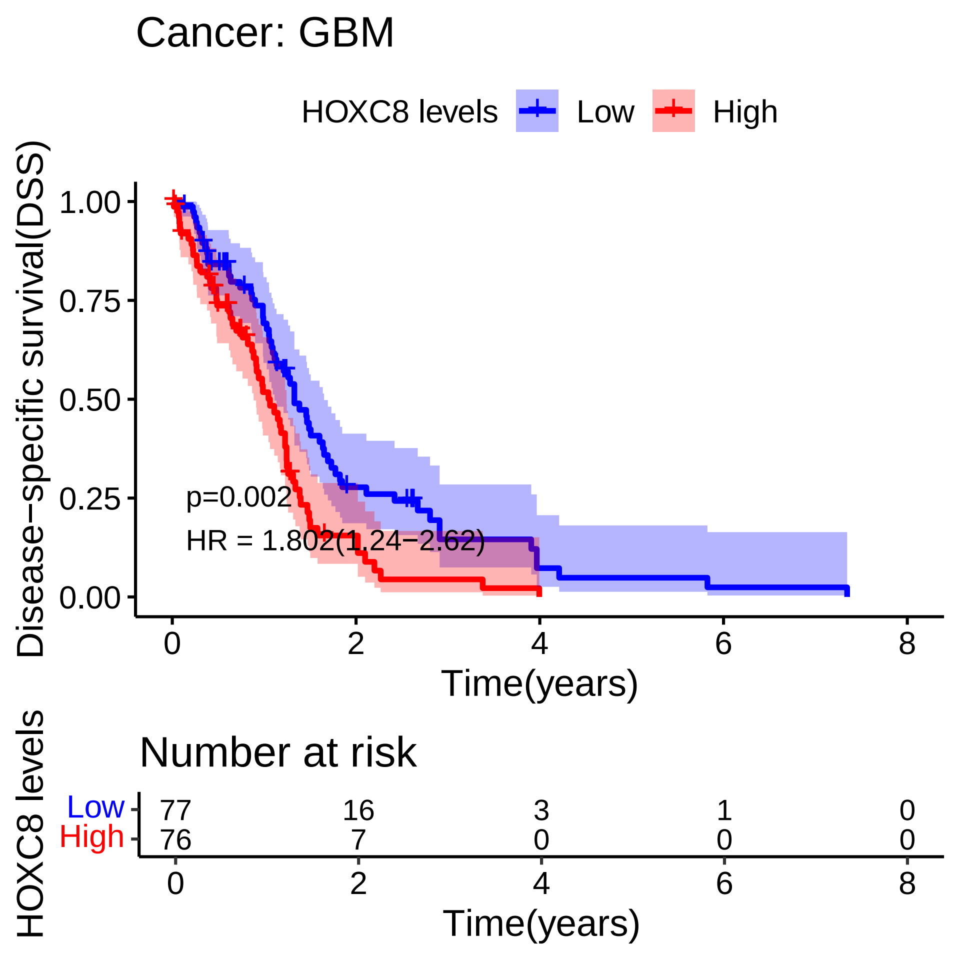
Cancer: GBM
HOXC8 levels
Low
High
Disease−specific survival(DSS)
1.00
0.75
0.50
0.25
0.00
p=0.002
HR = 1.802(1.24−2.62)
0
2
4
6
8
Time(years)
Number at risk
HOXC8 levels
Low
High
77
16
3
1
0
76
7
0
0
0
Time(years)

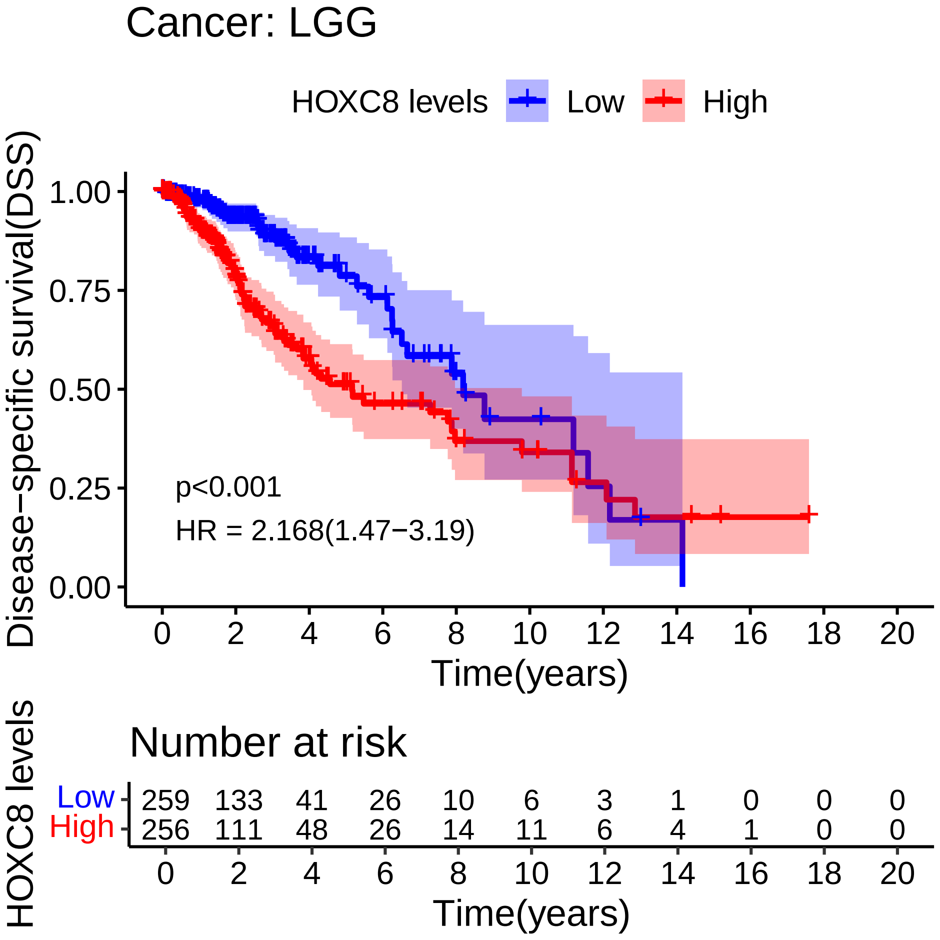
Cancer: LGG
HOXC8 levels
Low
High
Disease−specific survival(DSS)
1.00
0.75
0.50
0.25
0.00
p<0.001
HR = 2.168(1.47−3.19)
0
2
4
6
8
10
12
14
16
18
20
Time(years)
Number at risk
HOXC8 levels
Low 259 133 41 26 10 6 3 1 0 0 0
High 256 111 48 26 14 11 6 4 1 0 0
Time(years)

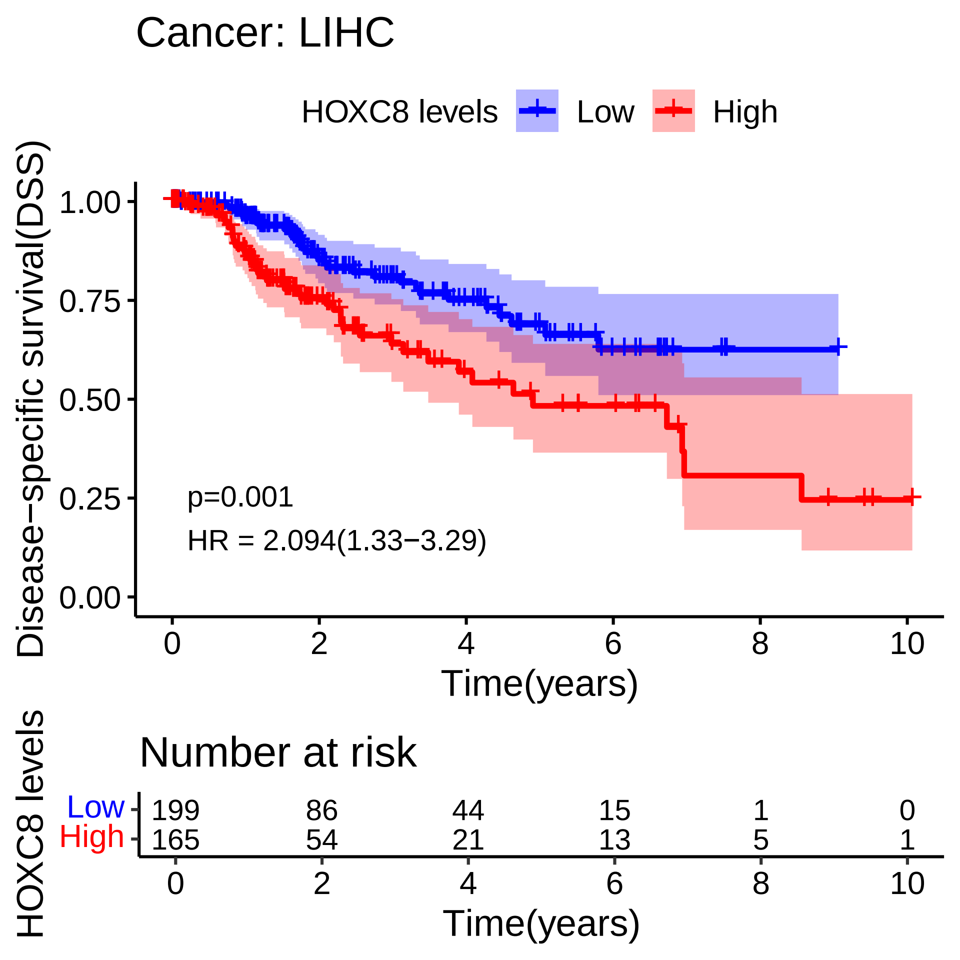
Cancer: LIHC
HOXC8 levels
Low
High
Disease−specific survival(DSS)
1.00
0.75
0.50
0.25
0.00
p=0.001
HR = 2.094(1.33−3.29)
0
2
4
6
8
10
Time(years)
Number at risk
HOXC8 levels
Low 199 86 44 15 1 0
High 165 54 21 13 5 1
Time(years)

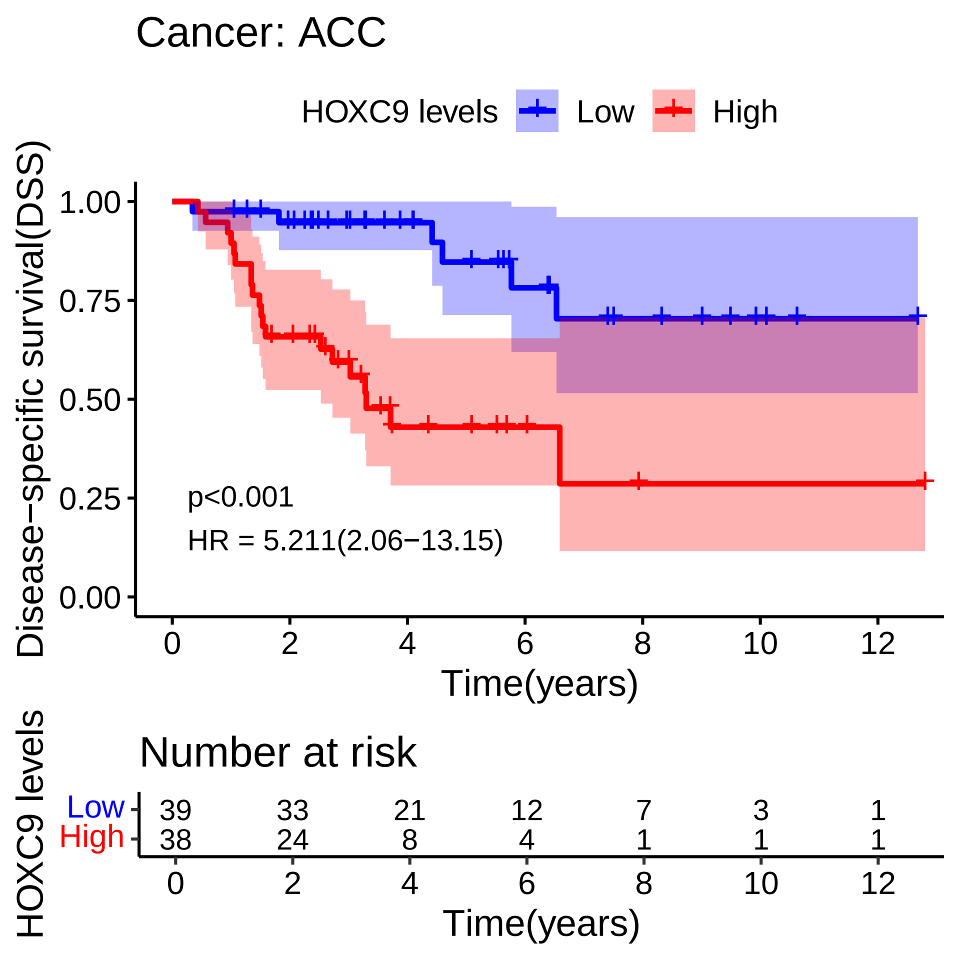

Cancer: ACC
HOXC9 levels
Low
High
Disease−specific survival(DSS)
1.00
0.75
0.50
0.25
0.00
p<0.001
HR = 5.211(2.06−13.15)
0
2
4
6
8
10
12
Time(years)
Number at risk
HOXC9 levels
Low
High
39 33 21 12 7 3 1
38 24 8 4 1 1 1
Time(years)

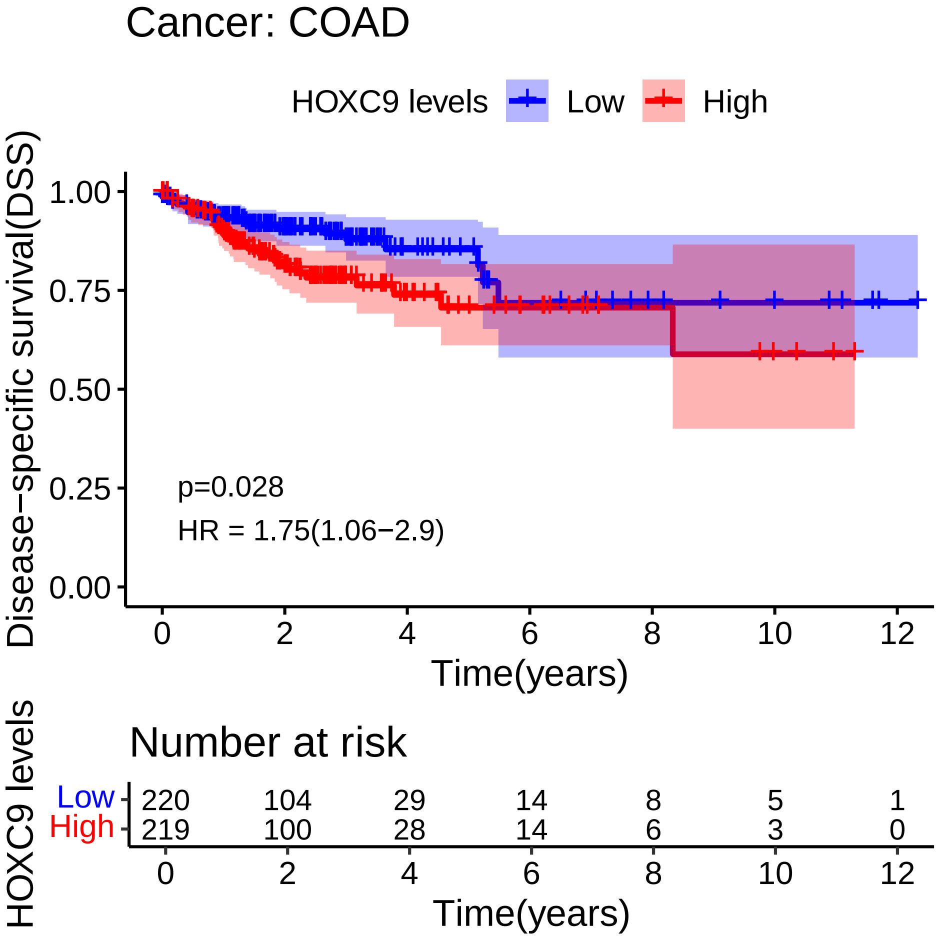

Cancer: COAD
HOXC9 levels
Low
High
Disease−specific survival(DSS)
1.00
0.75
0.50
0.25
0.00
p=0.028
HR = 1.75(1.06−2.9)
0
2
4
6
8
10
12
Time(years)
Number at risk
HOXC9 levels
Low
High
220 104 29 14 8 5 1
219 100 28 14 6 3 0
0
2
4
6
8
10
12
Time(years)

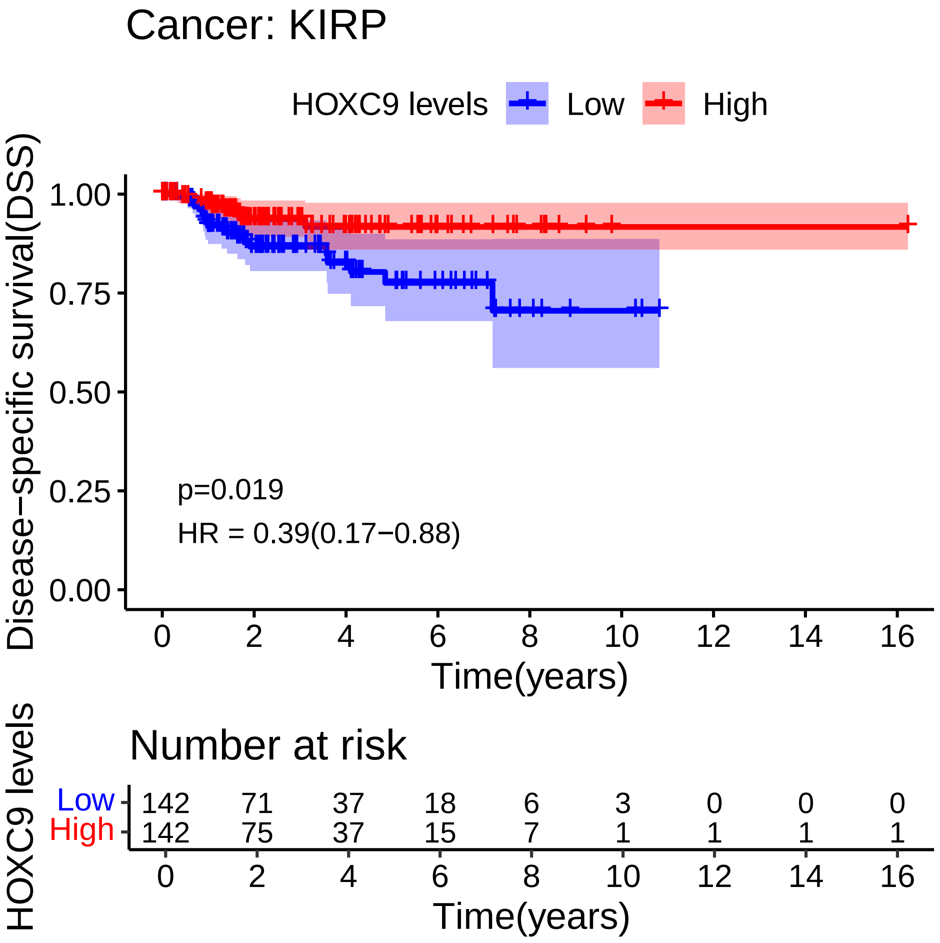
Cancer: KIRP
HOXC9 levels
Low
High
Disease−specific survival(DSS)
1.00
0.75
0.50
0.25
0.00
p=0.019
HR = 0.39(0.17−0.88)
0
2
4
6
8
10
12
14
16
Time(years)
Number at risk
HOXC9 levels
Low 142 71 37 18 6 3 0 0 0
High 142 75 37 15 7 1 1 1 1
0
2
4
6
8
10
12
14
16
Time(years)

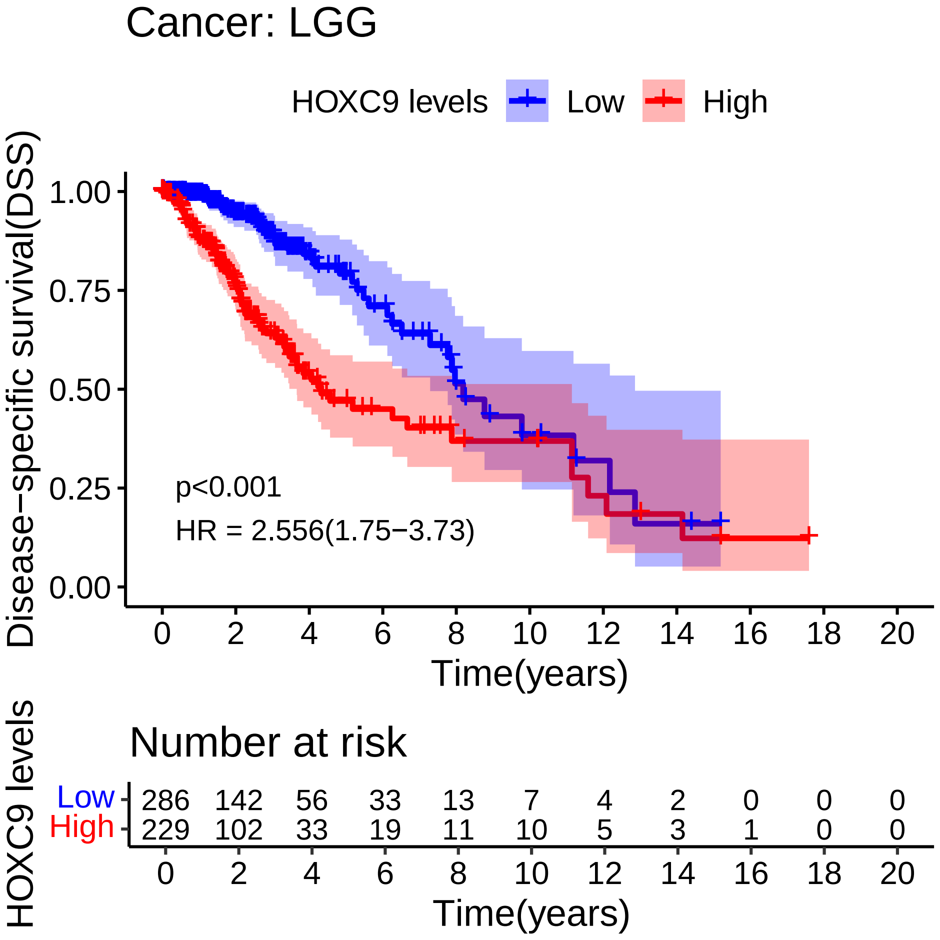

Cancer: LGG
HOXC9 levels
Low
High
Disease−specific survival(DSS)
1.00
0.75
0.50
0.25
0.00
p<0.001
HR = 2.556(1.75−3.73)
0 2 4 6 8 10 12 14 16 18 20
Time(years)
Number at risk
HOXC9 levels
Low 286 142 56 33 13 7 4 2 0 0 0
High 229 102 33 19 11 10 5 3 1 0 0
0 2 4 6 8 10 12 14 16 18 20
Time(years)

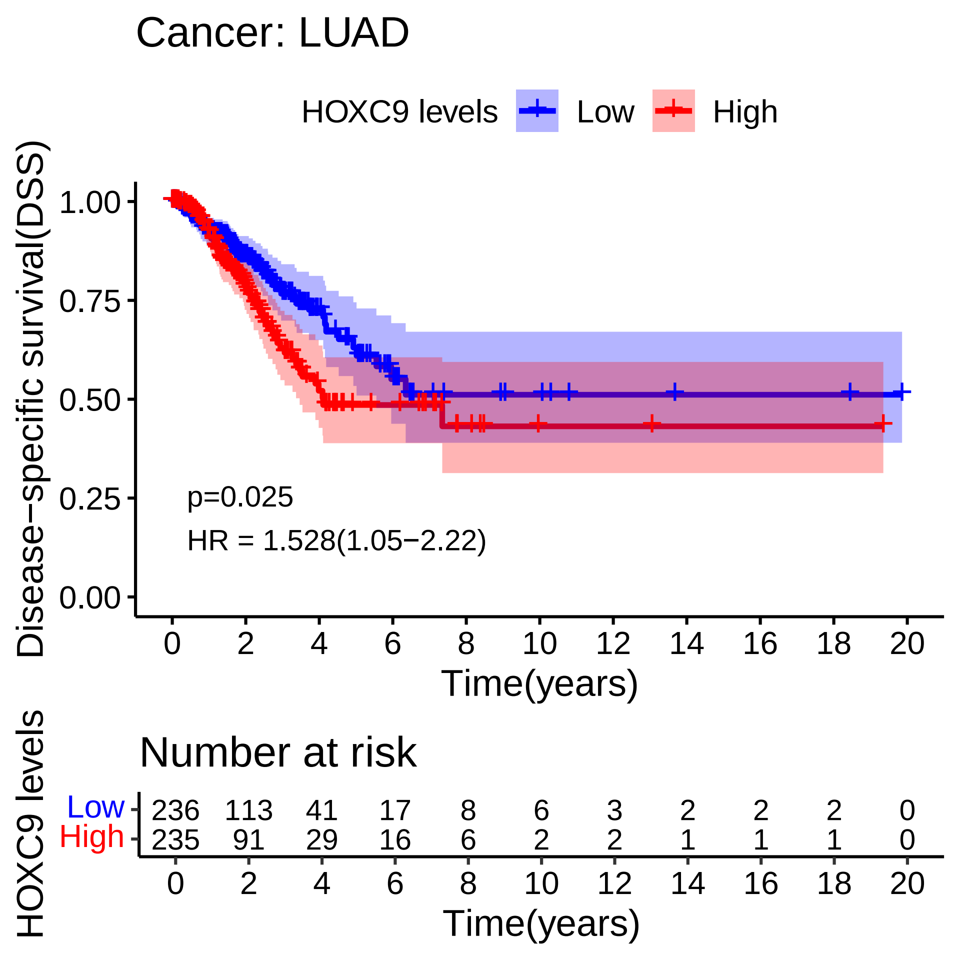
Cancer: LUAD
HOXC9 levels
Low
High
Disease−specific survival(DSS)
1.00
0.75
0.50
0.25
0.00
p=0.025
HR = 1.528(1.05−2.22)
0 2 4 6 8 10 12 14 16 18 20
Time(years)
Number at risk
HOXC9 levels
Low 236 113 41 17 8 6 3 2 2 2 0
High 235 91 29 16 6 2 2 1 1 1 0
0 2 4 6 8 10 12 14 16 18 20
Time(years)

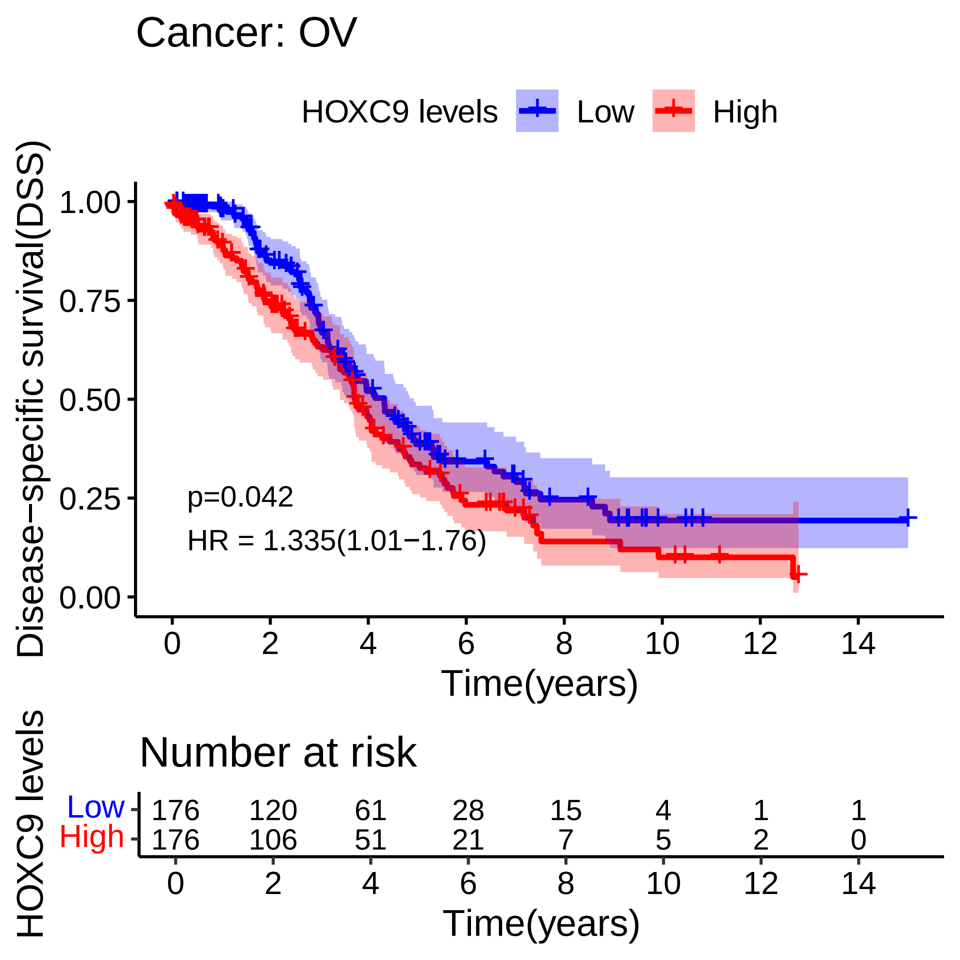
Cancer: OV
HOXC9 levels
Low
High
Disease−specific survival(DSS)
1.00
0.75
0.50
0.25
0.00
p=0.042
HR = 1.335(1.01−1.76)
0
2
4
6
8
10
12
14
Time(years)
Number at risk
HOXC9 levels
Low 176 120 61 28 15 4 1 1
High 176 106 51 21 7 5 2 0
Time(years)

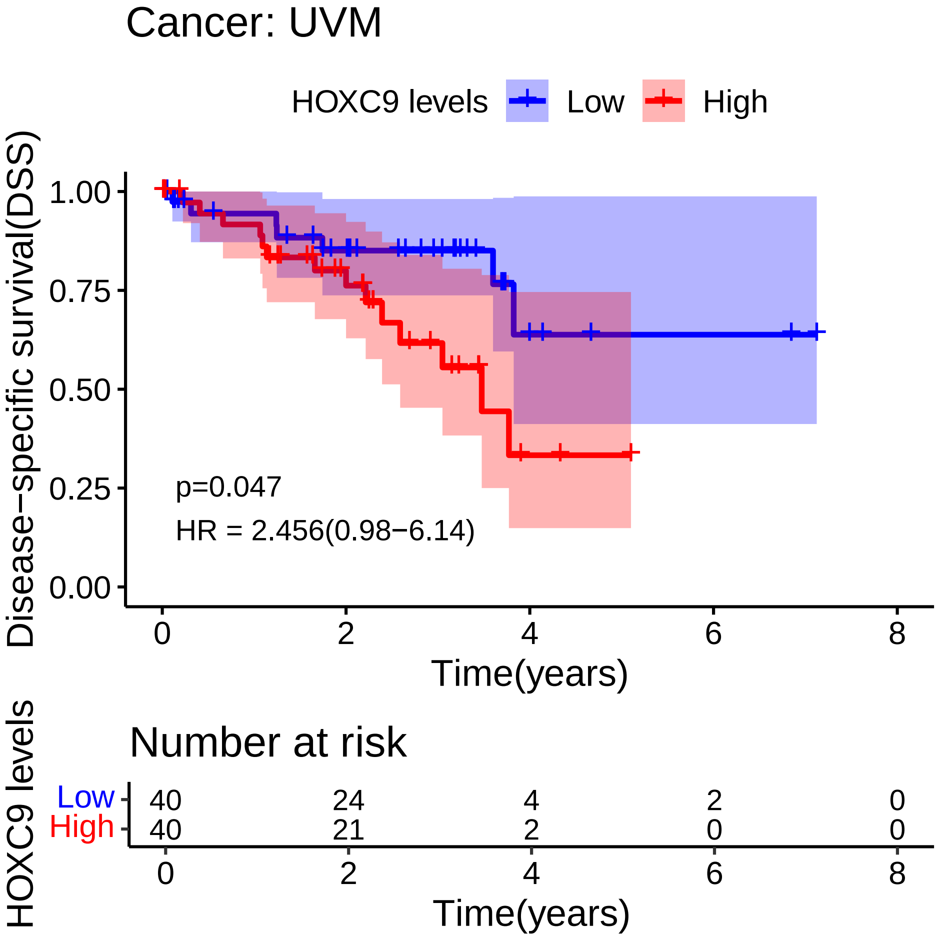

Cancer: UVM
HOXC9 levels
Low
High
Disease−specific survival(DSS)
1.00
0.75
0.50
0.25
0.00
p=0.047
HR = 2.456(0.98−6.14)
0
2
4
6
8
Time(years)
Number at risk
HOXC9 levels
Low 40 24 4 2 0
High 40 21 2 0 0
0
2
4
6
8
Time(years)

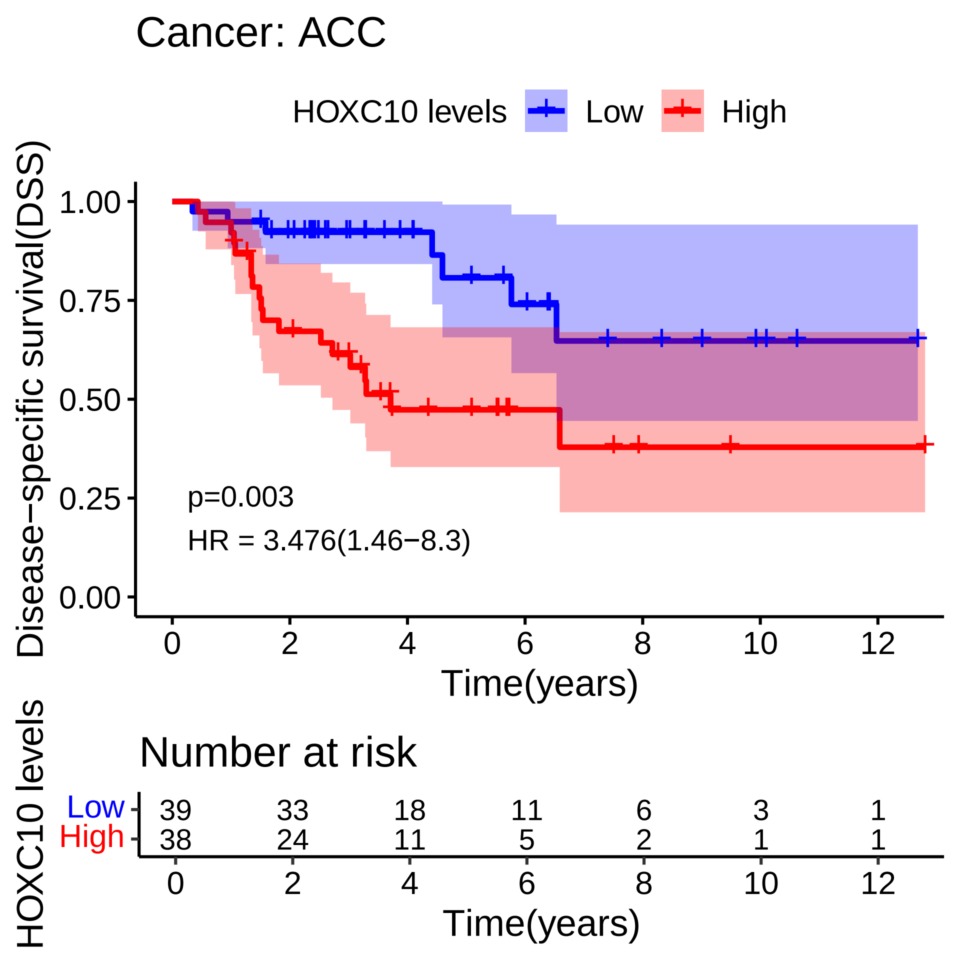

Cancer: ACC
HOXC10 levels
Low
High
Disease−specific survival(DSS)
1.00
0.75
0.50
0.25
0.00
p=0.003
HR = 3.476(1.46−8.3)
0
2
4
6
8
10
12
Time(years)
Number at risk
HOXC10 levels
Low 39 33 18 11 6 3 1
High 38 24 11 5 2 1 1
Time(years)

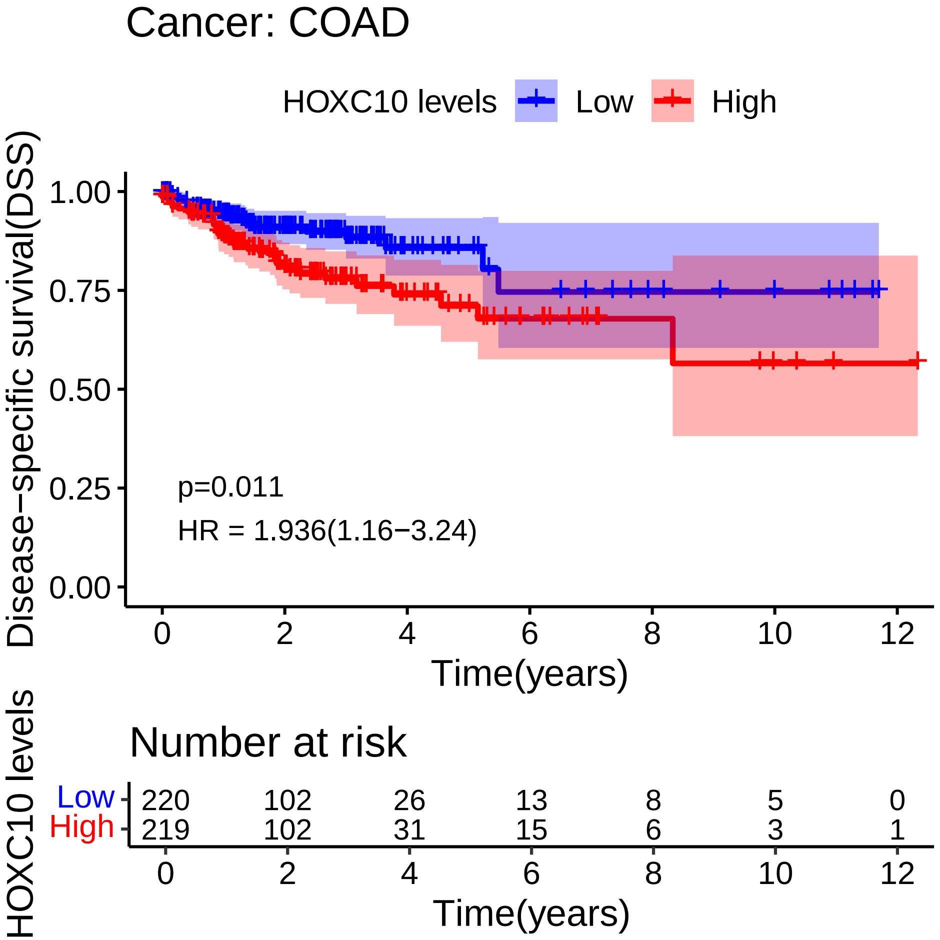

Cancer: COAD
HOXC10 levels
Low
High
Disease−specific survival(DSS)
1.00
0.75
0.50
0.25
0.00
p=0.011
HR = 1.936(1.16−3.24)
0
2
4
6
8
10
12
Time(years)
Number at risk
HOXC10 levels
Low 220 102 26 13 8 5 0
High 219 102 31 15 6 3 1
0
2
4
6
8
10
12
Time(years)

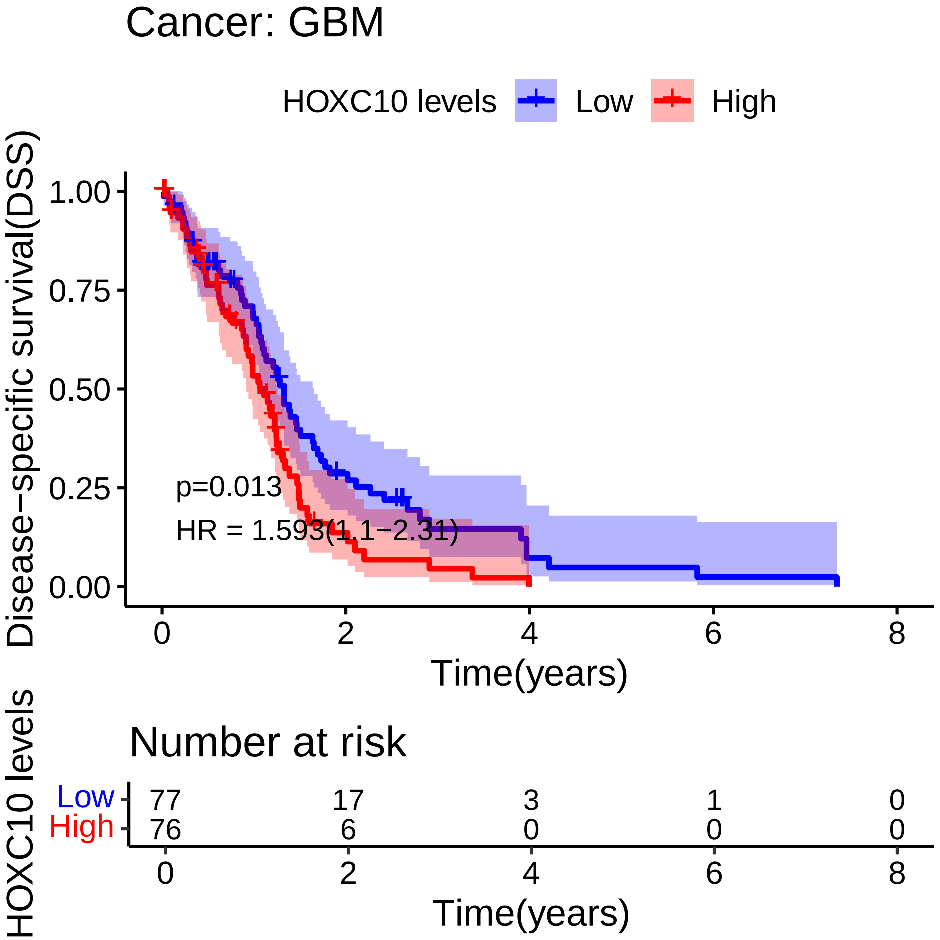
Cancer: GBM
HOXC10 levels
Low
High
Disease−specific survival(DSS)
1.00
0.75
0.50
0.25
0.00
p=0.013
HR = 1.593(1.1−2.31)
0
2
4
6
8
Time(years)
Number at risk
HOXC10 levels
Low
High
77
17
3
1
0
76
6
0
0
0
0
2
4
6
8
Time(years)

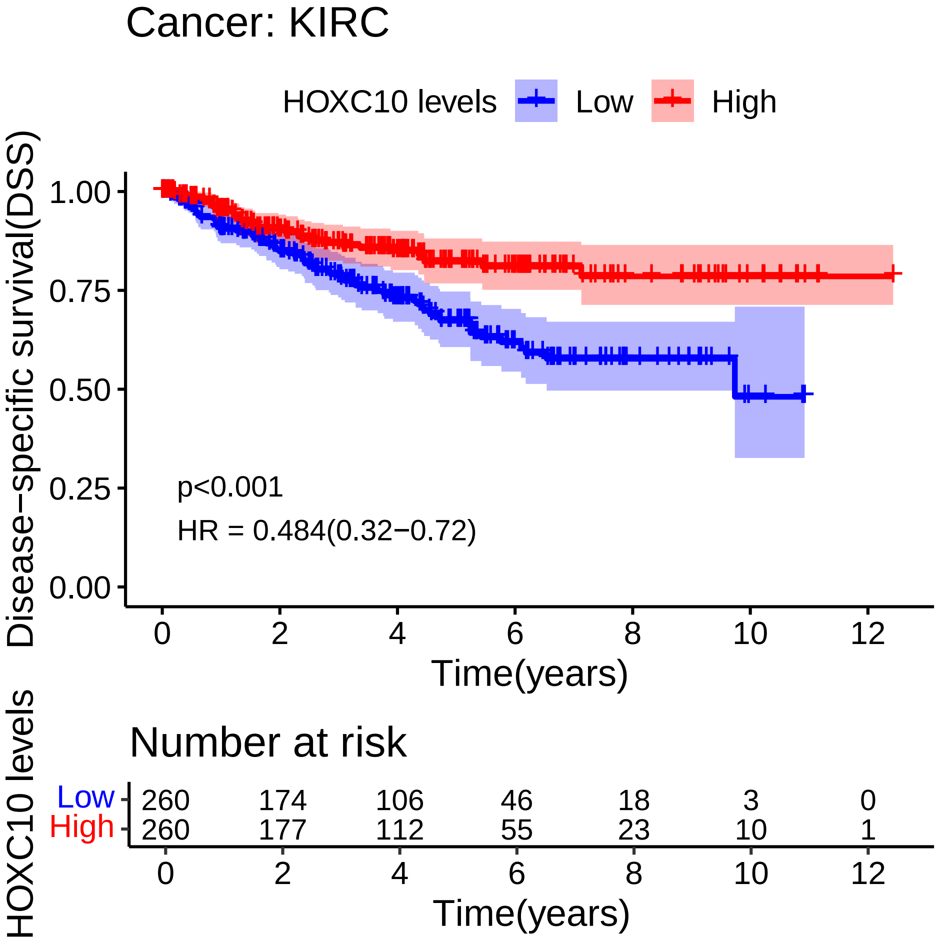

Cancer: KIRC
HOXC10 levels
Low
High
Disease−specific survival(DSS)
1.00
0.75
0.50
0.25
0.00
p<0.001
HR = 0.484(0.32−0.72)
0
2
4
6
8
10
12
Time(years)
Number at risk
HOXC10 levels
Low 260 174 106 46 18 3 0
High 260 177 112 55 23 10 1
0
2
4
6
8
10
12
Time(years)

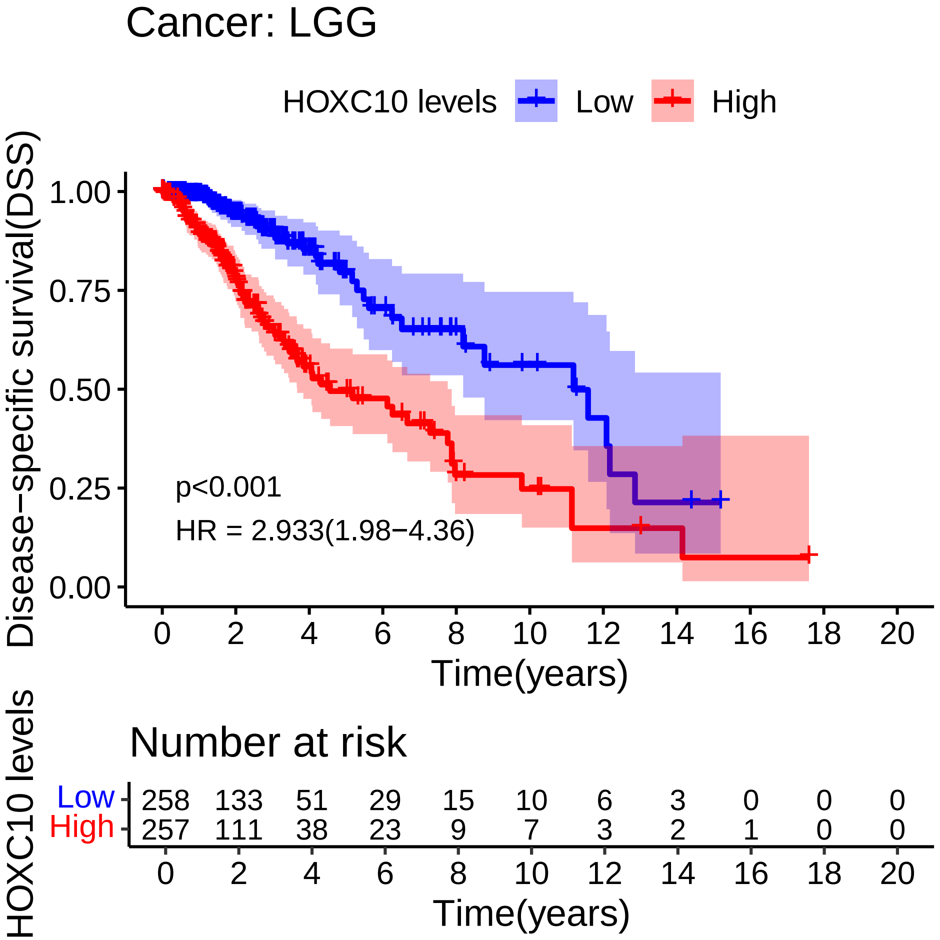
Cancer: LGG
HOXC10 levels
Low
High
Disease−specific survival(DSS)
1.00
0.75
0.50
0.25
0.00
p<0.001
HR = 2.933(1.98−4.36)
0 2 4 6 8 10 12 14 16 18 20
Time(years)
Number at risk
HOXC10 levels
Low 258 133 51 29 15 10 6 3 0 0 0
High 257 111 38 23 9 7 3 2 1 0 0
0 2 4 6 8 10 12 14 16 18 20
Time(years)

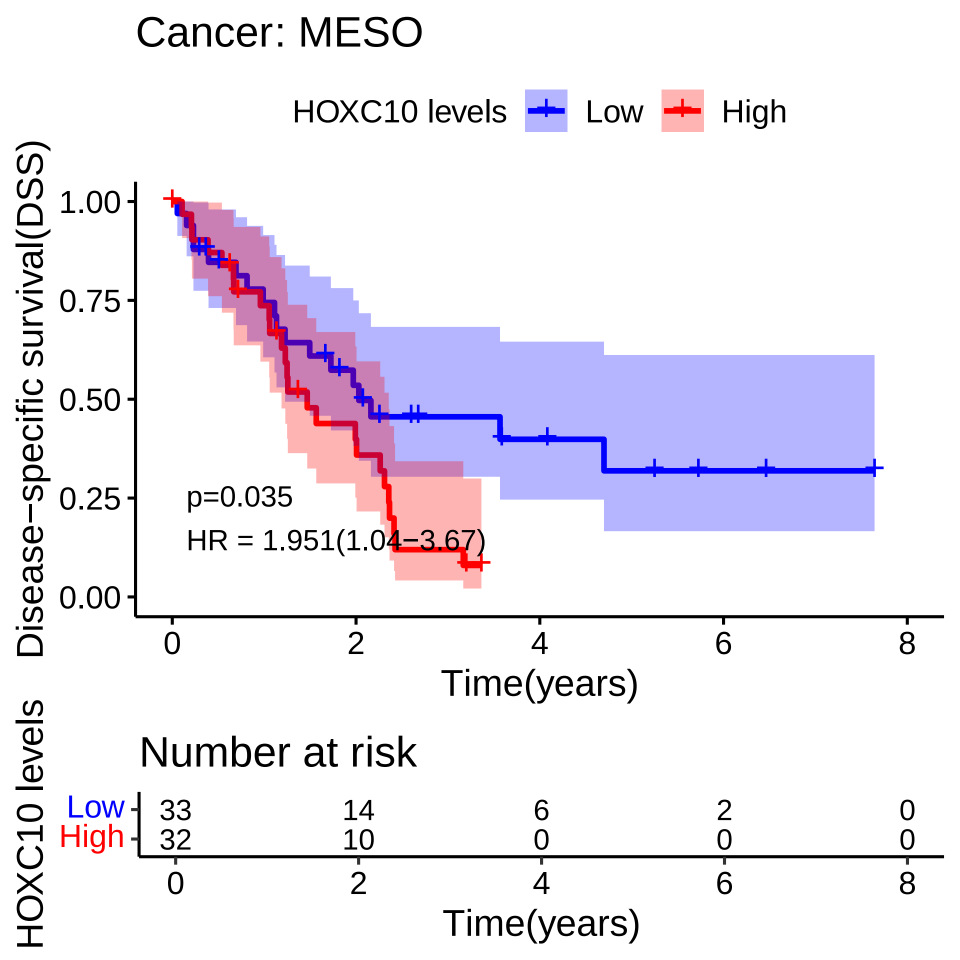

Cancer: MESO
HOXC10 levels
Low
High
Disease−specific survival(DSS)
1.00
0.75
0.50
0.25
0.00
p=0.035
HR = 1.951(1.04−3.67)
0
2
4
6
8
Time(years)
Number at risk
HOXC10 levels
Low 33 14 6 2 0
High 32 10 0 0 0
0
2
4
6
8
Time(years)

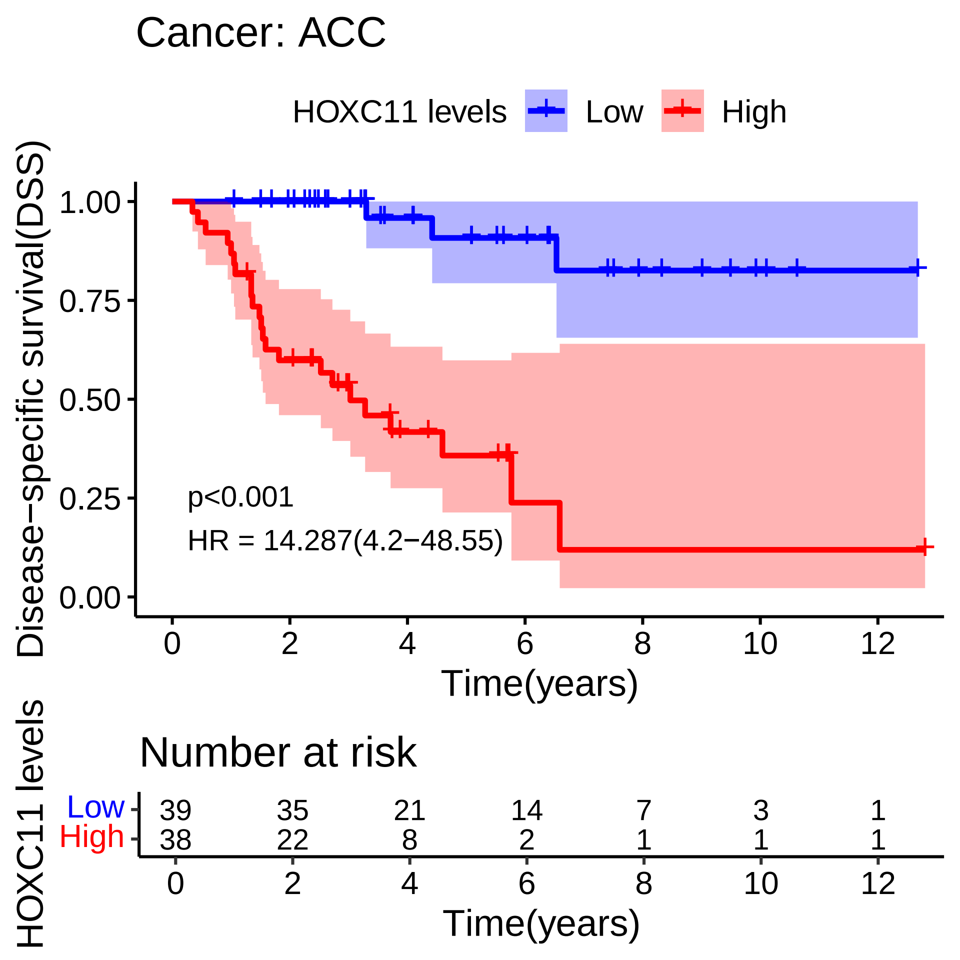

Cancer: ACC
HOXC11 levels
Low
High
Disease−specific survival(DSS)
1.00
0.75
0.50
0.25
0.00
p<0.001
HR = 14.287(4.2−48.55)
0
2
4
6
8
10
12
Time(years)
Number at risk
HOXC11 levels
Low 39 35 21 14 7 3 1
High 38 22 8 2 1 1 1
0
2
4
6
8
10
12
Time(years)

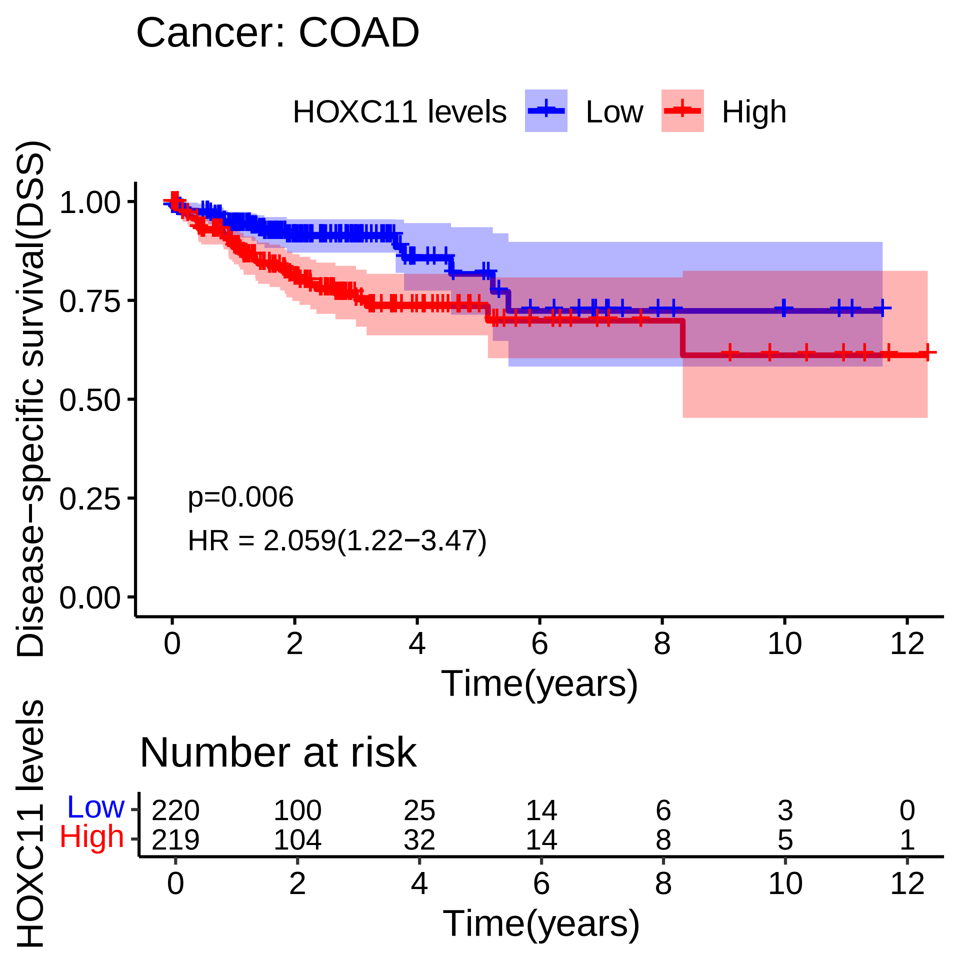
Cancer: COAD
HOXC11 levels
Low
High
Disease−specific survival(DSS)
1.00
0.75
0.50
0.25
0.00
p=0.006
HR = 2.059(1.22−3.47)
0
2
4
6
8
10
12
Time(years)
Number at risk
HOXC11 levels
Low 220 100 25 14 6 3 0
High 219 104 32 14 8 5 1
Time(years)

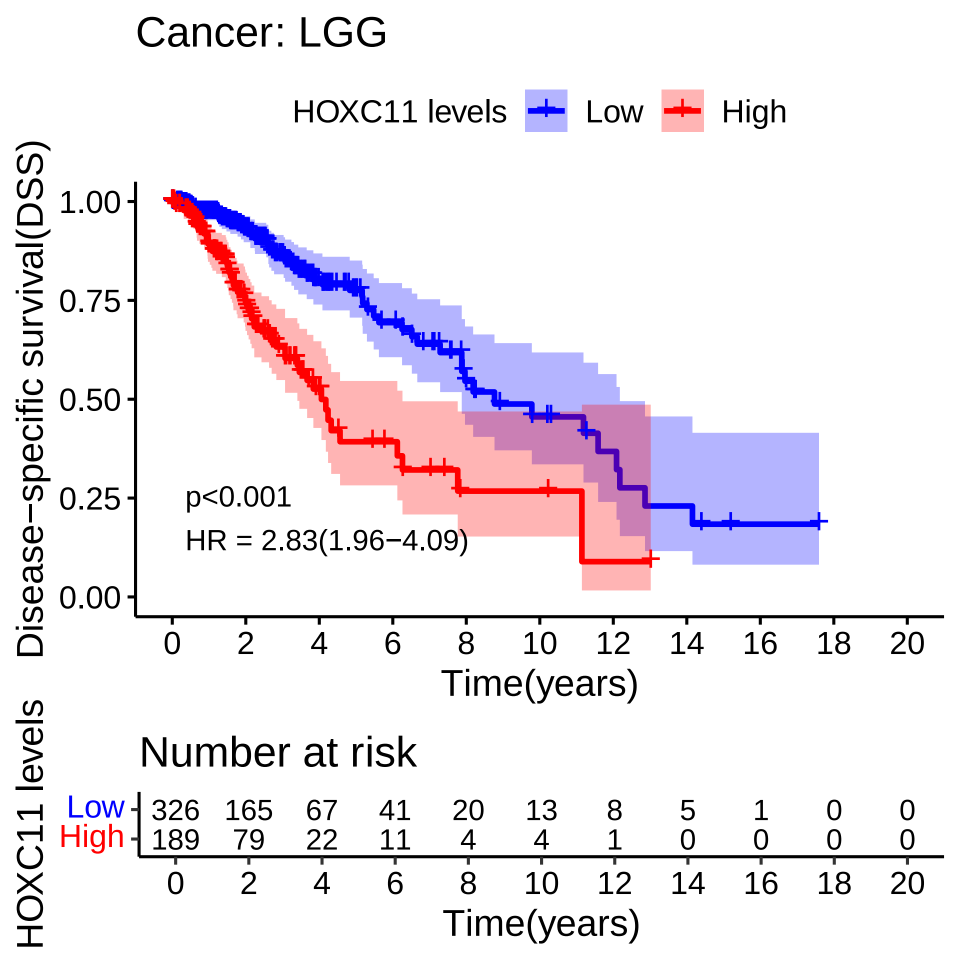

Cancer: LGG
HOXC11 levels
Low
High
Disease−specific survival(DSS)
1.00
0.75
0.50
0.25
0.00
p<0.001
HR = 2.83(1.96−4.09)
0 2 4 6 8 10 12 14 16 18 20
Time(years)
Number at risk
HOXC11 levels
Low 326 165 67 41 20 13 8 5 1 0 0
High 189 79 22 11 4 4 1 0 0 0 0
0 2 4 6 8 10 12 14 16 18 20
Time(years)

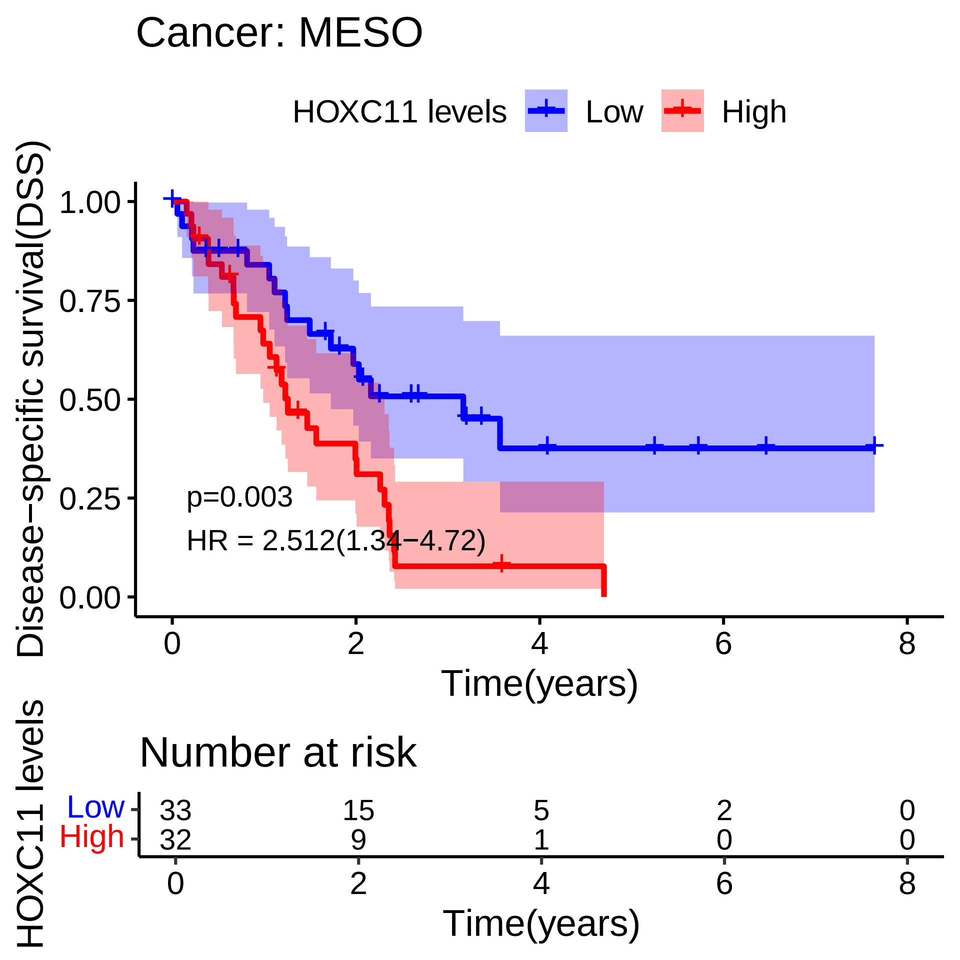

Cancer: MESO
HOXC11 levels
Low
High
Disease−specific survival(DSS)
1.00
0.75
0.50
0.25
0.00
p=0.003
HR = 2.512(1.34−4.72)
0
2
4
6
8
Time(years)
Number at risk
HOXC11 levels
Low
High
33
15
5
2
0
32
9
1
0
0
0
2
4
6
8
Time(years)

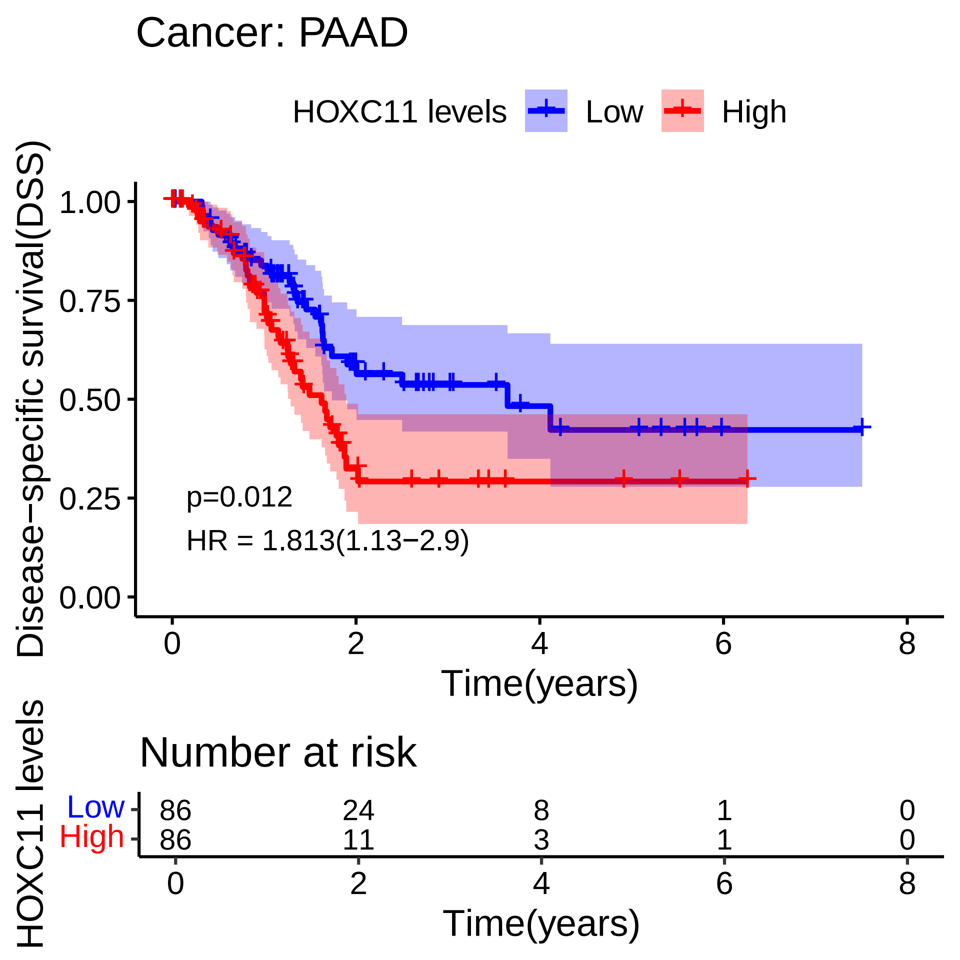

Cancer: PAAD
HOXC11 levels
Low
High
Disease−specific survival(DSS)
1.00
0.75
0.50
0.25
0.00
p=0.012
HR = 1.813(1.13−2.9)
0
2
4
6
8
Time(years)
Number at risk
HOXC11 levels
Low
High
86
24
8
1
0
86
11
3
1
0
0
2
4
6
8
Time(years)

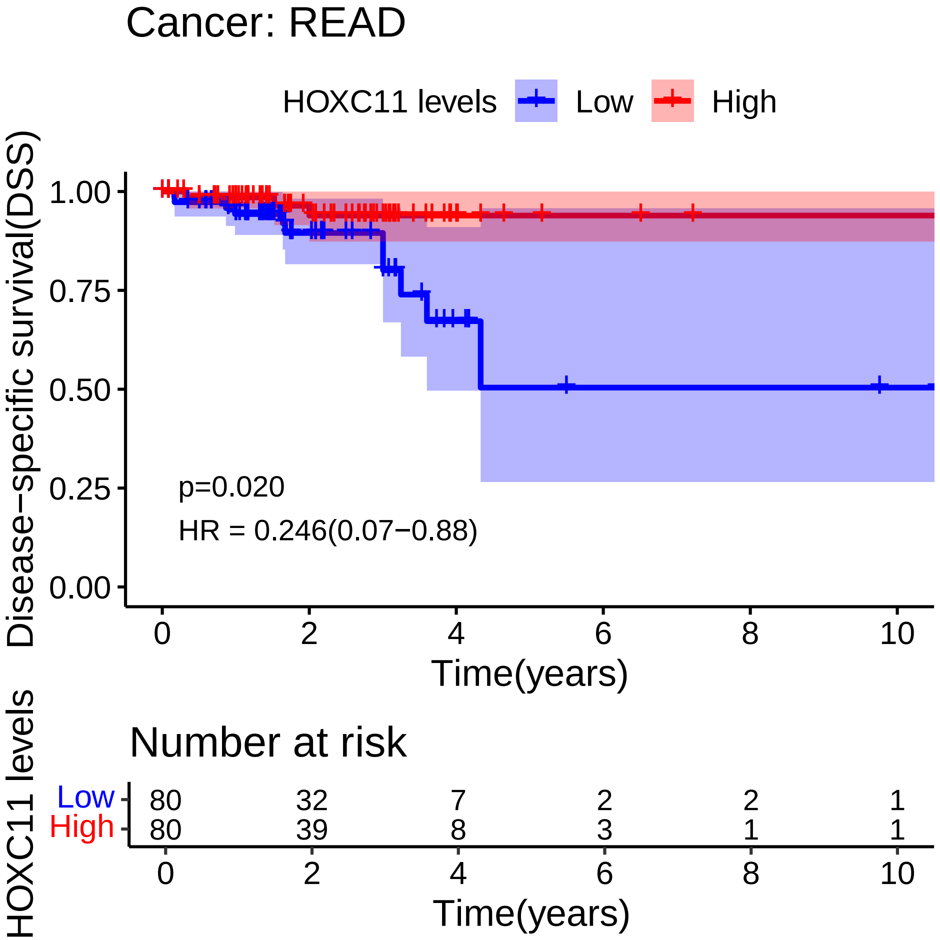

Cancer: READ
HOXC11 levels
Low
High
Disease−specific survival(DSS)
1.00
0.75
0.50
0.25
0.00
p=0.020
HR = 0.246(0.07−0.88)
0
2
4
6
8
10
Time(years)
Number at risk
HOXC11 levels
Low
High
80
32
7
2
2
1
80
39
8
3
1
1
0
2
4
6
8
10
Time(years)

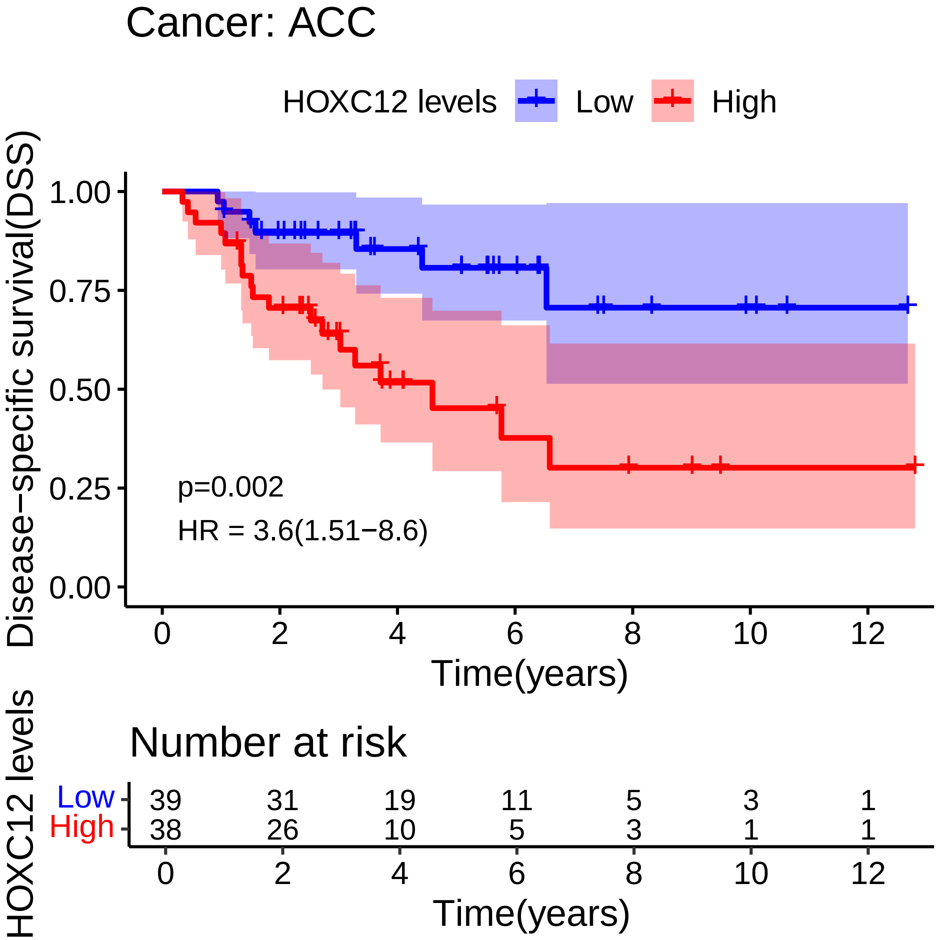

Cancer: ACC
HOXC12 levels
Low
High
Disease−specific survival(DSS)
1.00
0.75
0.50
0.25
0.00
p=0.002
HR = 3.6(1.51−8.6)
0
2
4
6
8
10
12
Time(years)
Number at risk
HOXC12 levels
Low 39 31 19 11 5 3 1
High 38 26 10 5 3 1 1
0
2
4
6
8
10
12
Time(years)

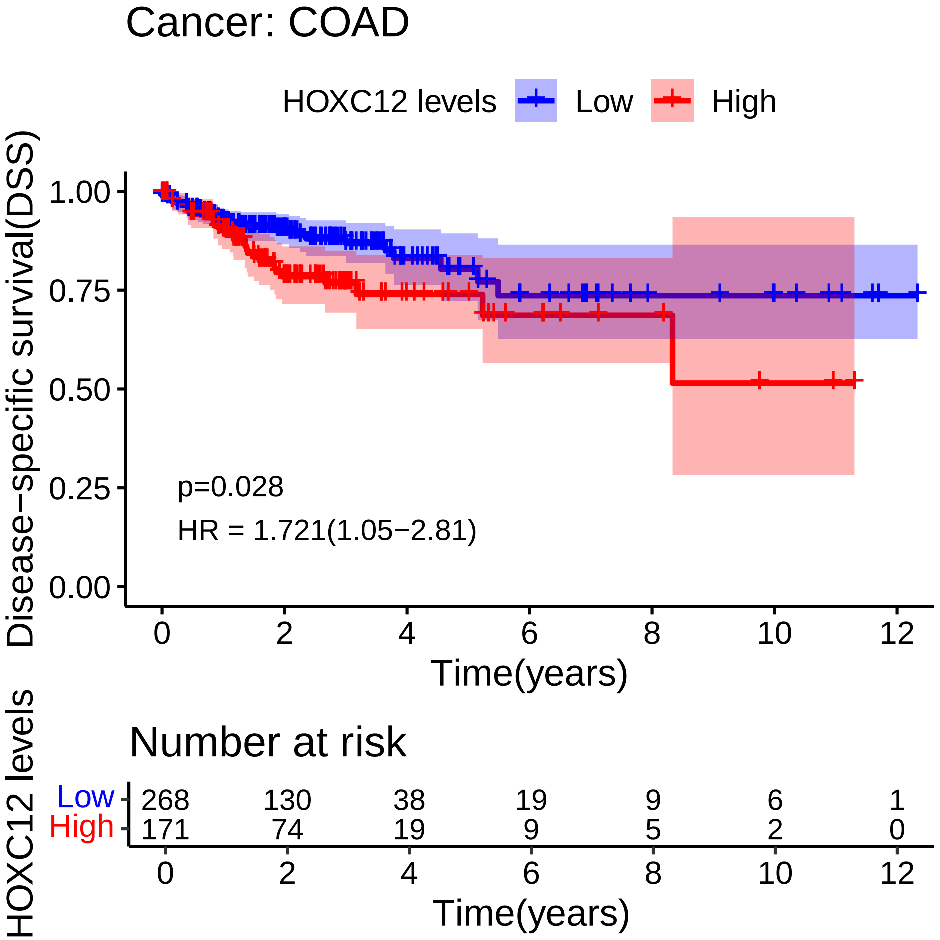

Cancer: COAD
HOXC12 levels
Low
High
Disease−specific survival(DSS)
1.00
0.75
0.50
0.25
0.00
p=0.028
HR = 1.721(1.05−2.81)
0
2
4
6
8
10
12
Time(years)
Number at risk
HOXC12 levels
Low 268 130 38 19 9 6 1
High 171 74 19 9 5 2 0
Time(years)

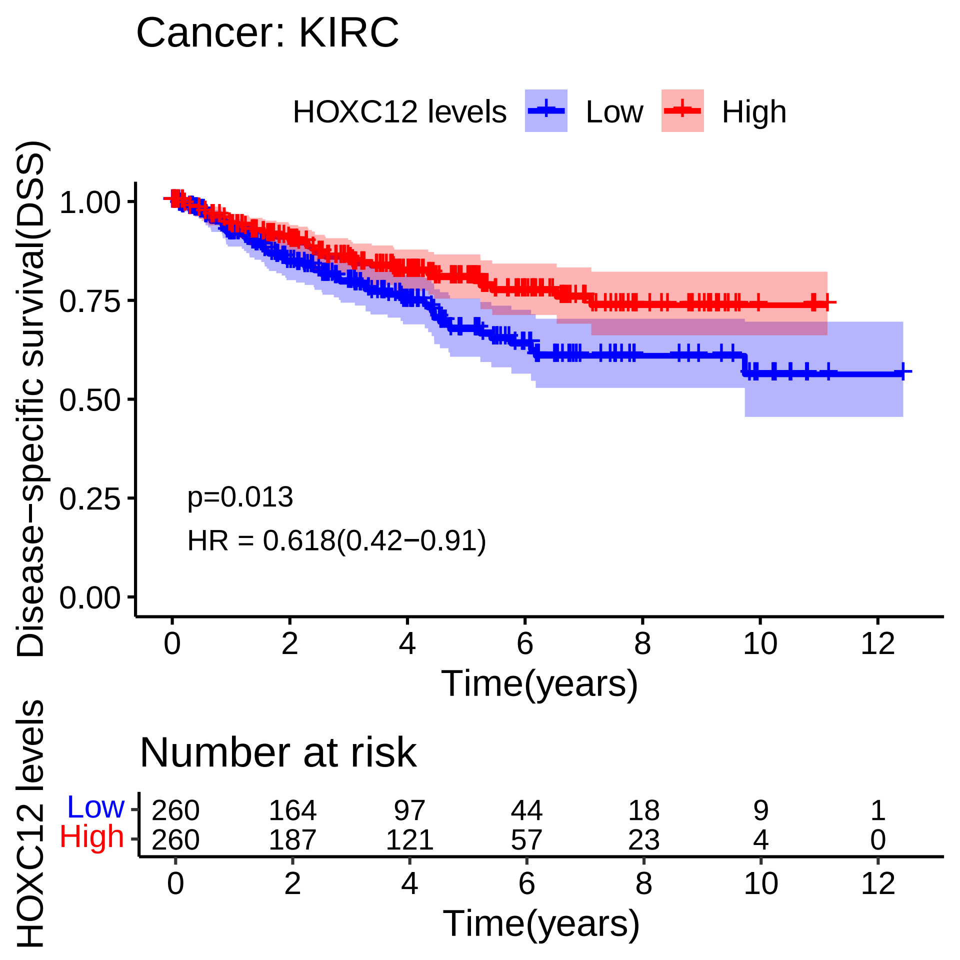

Cancer: KIRC
HOXC12 levels
Low
High
Disease−specific survival(DSS)
1.00
0.75
0.50
0.25
0.00
p=0.013
HR = 0.618(0.42−0.91)
0
2
4
6
8
10
12
Time(years)
Number at risk
HOXC12 levels
Low 260 164 97 44 18 9 1
High 260 187 121 57 23 4 0
0
2
4
6
8
10
12
Time(years)

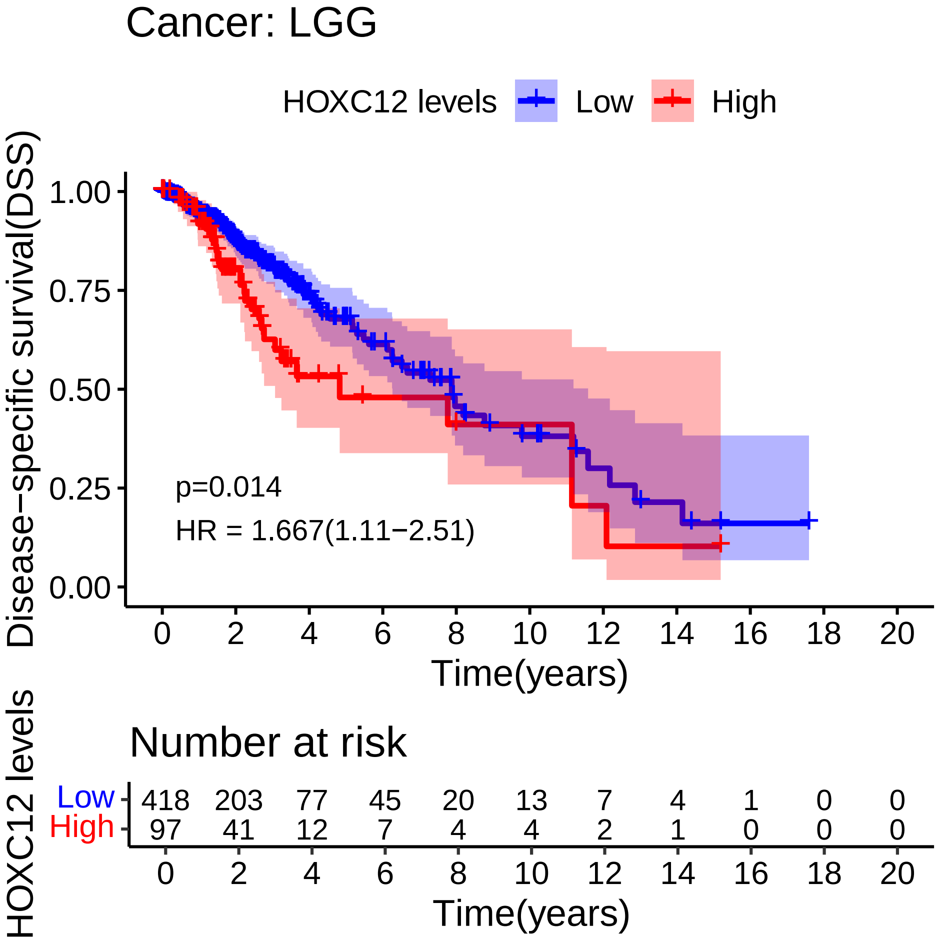

Cancer: LGG
HOXC12 levels
Low
High
Disease−specific survival(DSS)
1.00
0.75
0.50
0.25
0.00
p=0.014
HR = 1.667(1.11−2.51)
0
2
4
6
8
10
12
14
16
18
20
Time(years)
Number at risk
HOXC12 levels
Low
High
418 203 77 45 20 13 7 4 1 0 0
97 41 12 7 4 4 2 1 0 0 0
Time(years)

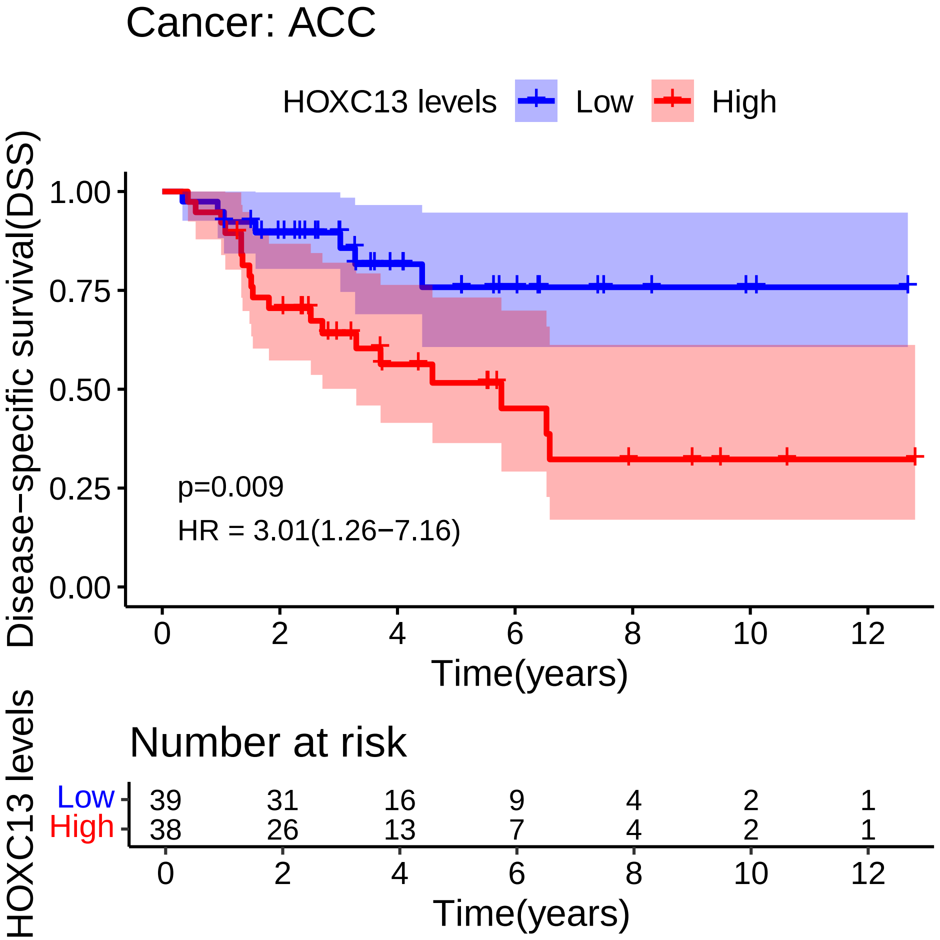

Cancer: ACC
HOXC13 levels
Low
High
Disease−specific survival(DSS)
1.00
0.75
0.50
0.25
0.00
p=0.009
HR = 3.01(1.26−7.16)
0
2
4
6
8
10
12
Time(years)
Number at risk
HOXC13 levels
Low 39 31 16 9 4 2 1
High 38 26 13 7 4 2 1
0
2
4
6
8
10
12
Time(years)

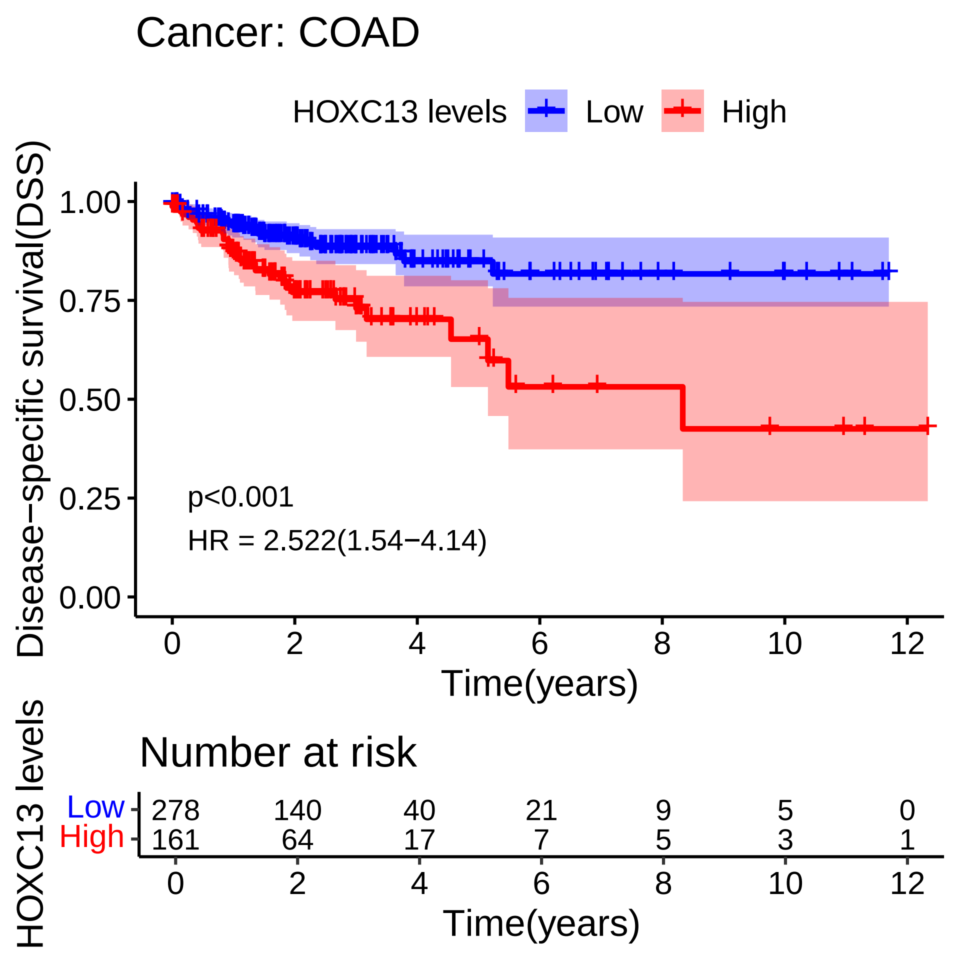

Cancer: COAD
HOXC13 levels
Low
High
Disease−specific survival(DSS)
1.00
0.75
0.50
0.25
0.00
p<0.001
HR = 2.522(1.54−4.14)
0
2
4
6
8
10
12
Time(years)
Number at risk
HOXC13 levels
Low 278 140 40 21 9 5 0
High 161 64 17 7 5 3 1
Time(years)

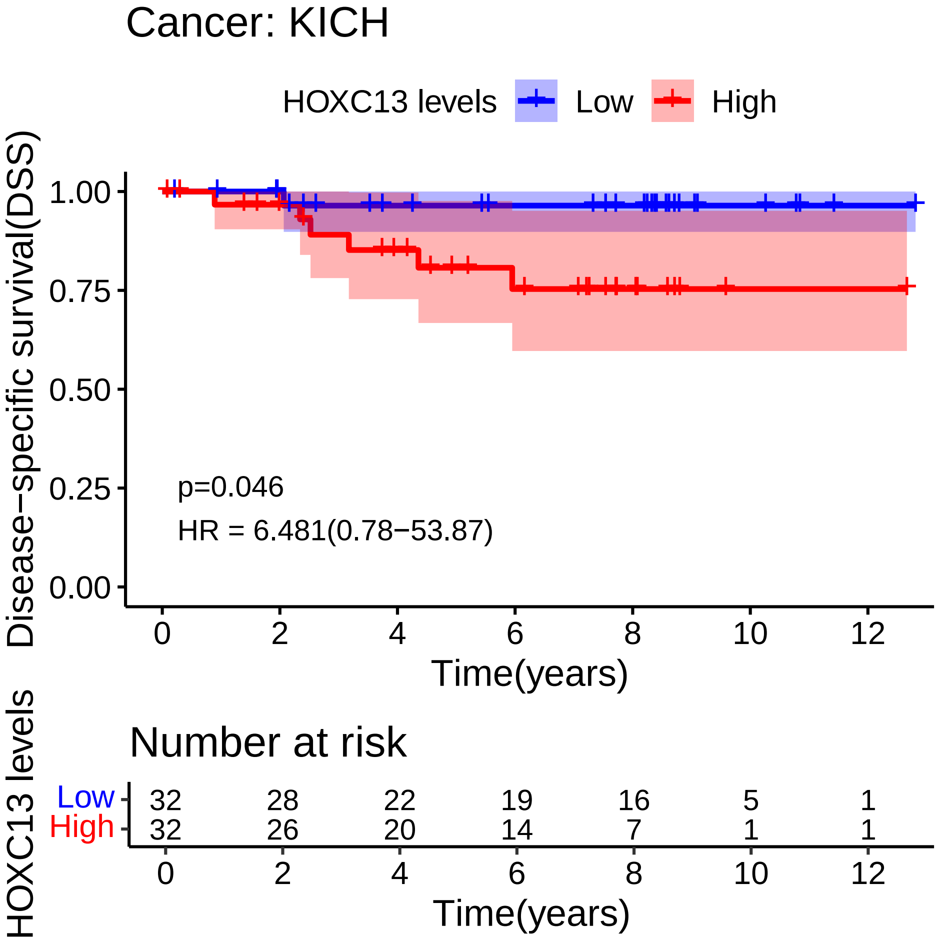

Cancer: KICH
HOXC13 levels
Low
High
Disease−specific survival(DSS)
1.00
0.75
0.50
0.25
0.00
p=0.046
HR = 6.481(0.78−53.87)
0
2
4
6
8
10
12
Time(years)
Number at risk
HOXC13 levels
Low
High
32 28 22 19 16 5 1
32 26 20 14 7 1 1
0
2
4
6
8
10
12
Time(years)

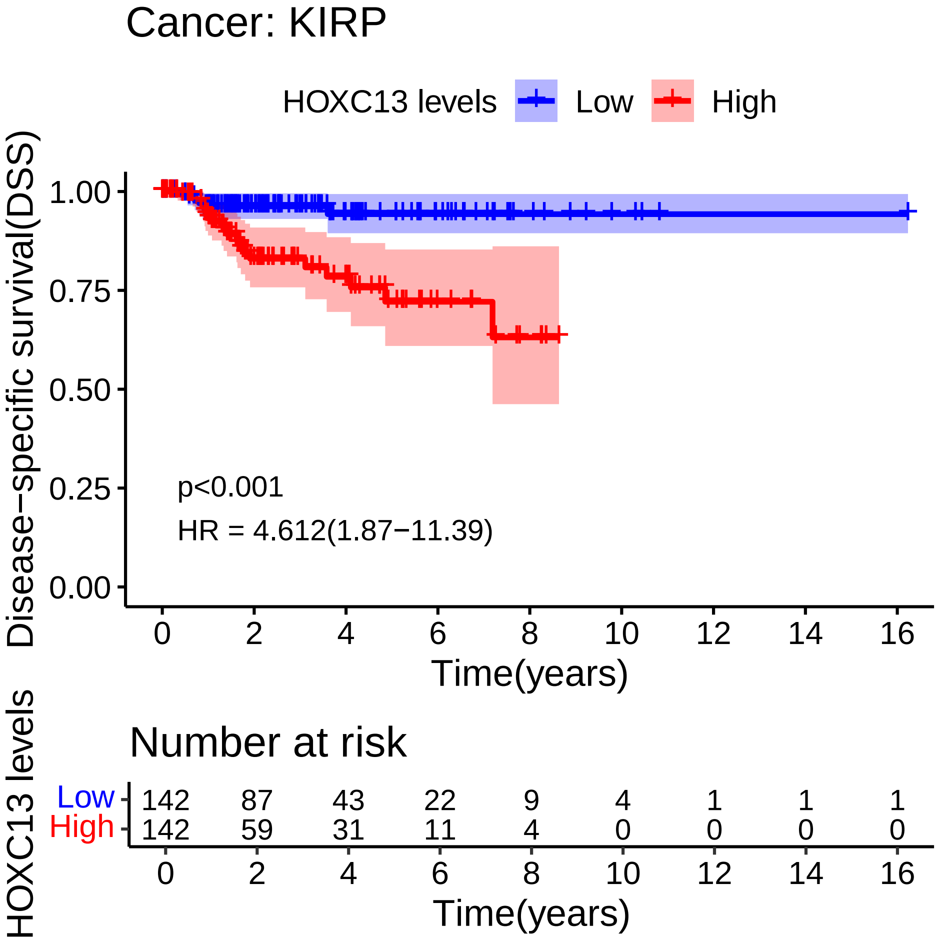
Cancer: KIRP
HOXC13 levels
Low
High
Disease−specific survival(DSS)
1.00
0.75
0.50
0.25
0.00
p<0.001
HR = 4.612(1.87−11.39)
0
2
4
6
8
10
12
14
16
Time(years)
Number at risk
HOXC13 levels
Low 142 87 43 22 9 4 1 1 1
High 142 59 31 11 4 0 0 0 0
Time(years)

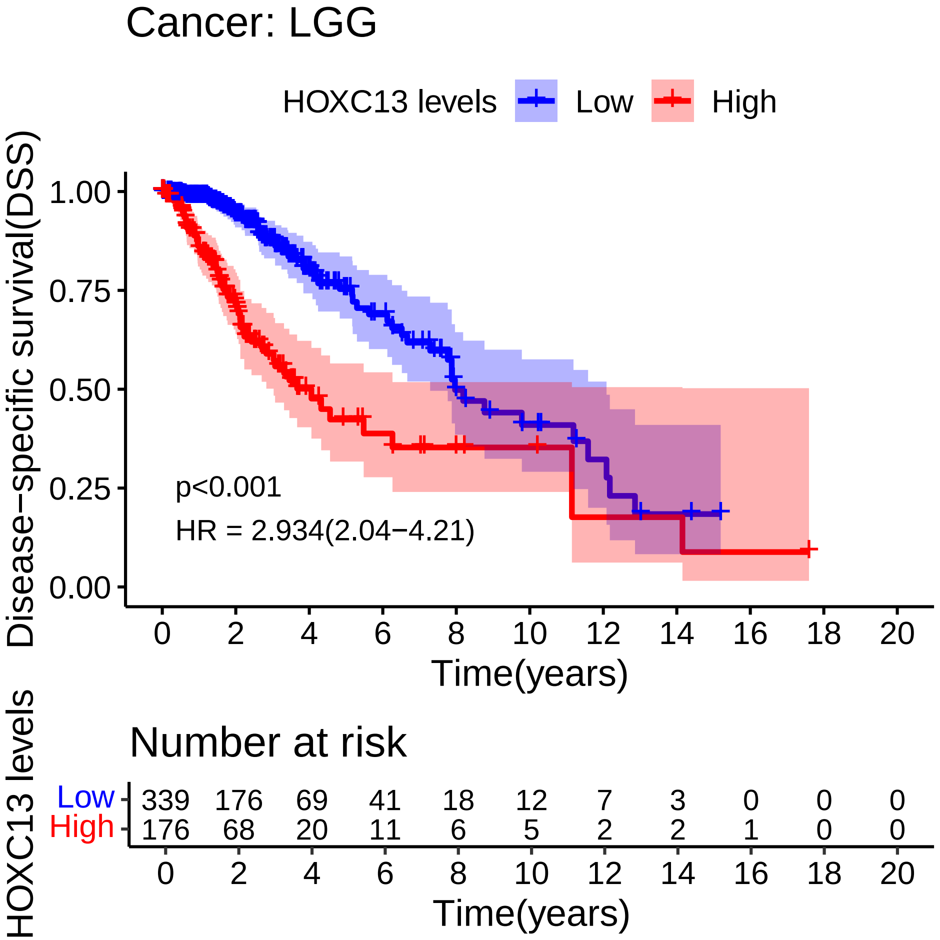
Cancer: LGG
HOXC13 levels
Low
High
Disease−specific survival(DSS)
1.00
0.75
0.50
0.25
0.00
p<0.001
HR = 2.934(2.04−4.21)
0 2 4 6 8 10 12 14 16 18 20
Time(years)
Number at risk
HOXC13 levels
Low 339 176 69 41 18 12 7 3 0 0 0
High 176 68 20 11 6 5 2 2 1 0 0
0 2 4 6 8 10 12 14 16 18 20
Time(years)

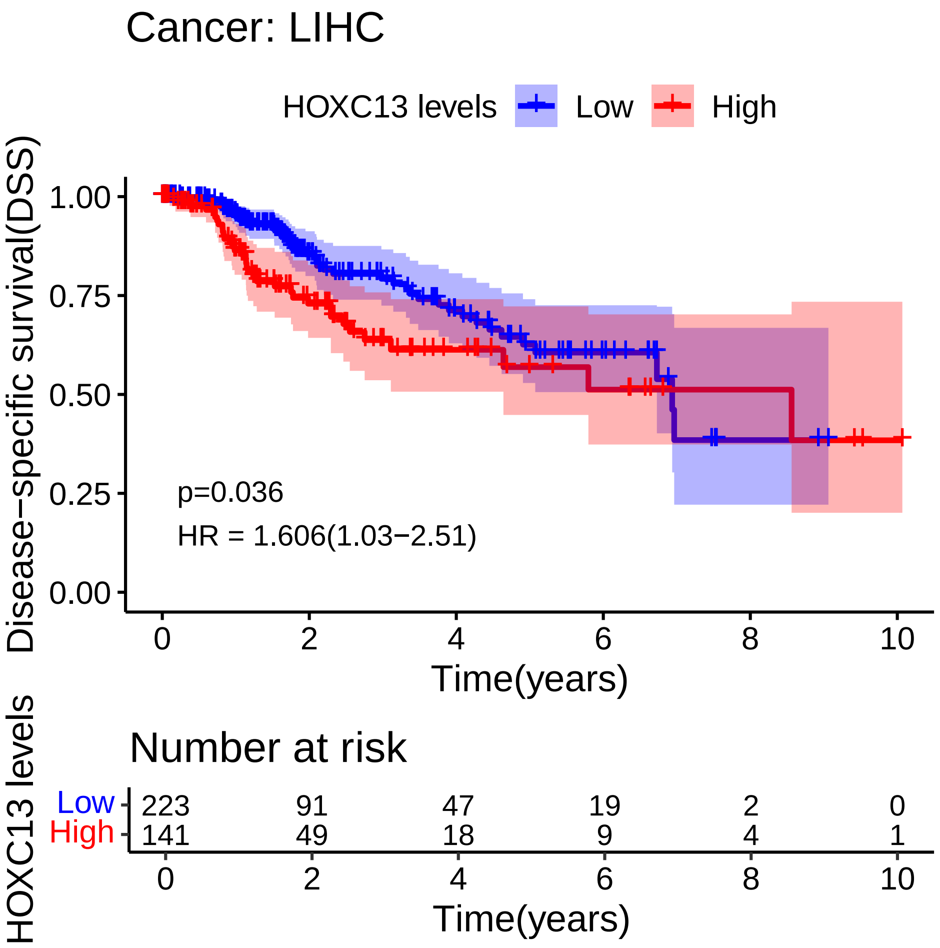

Cancer: LIHC
HOXC13 levels
Low
High
Disease−specific survival(DSS)
1.00
0.75
0.50
0.25
0.00
p=0.036
HR = 1.606(1.03−2.51)
0
2
4
6
8
10
Time(years)
Number at risk
HOXC13 levels
Low 223 91 47 19 2 0
High 141 49 18 9 4 1
0
2
4
6
8
10
Time(years)

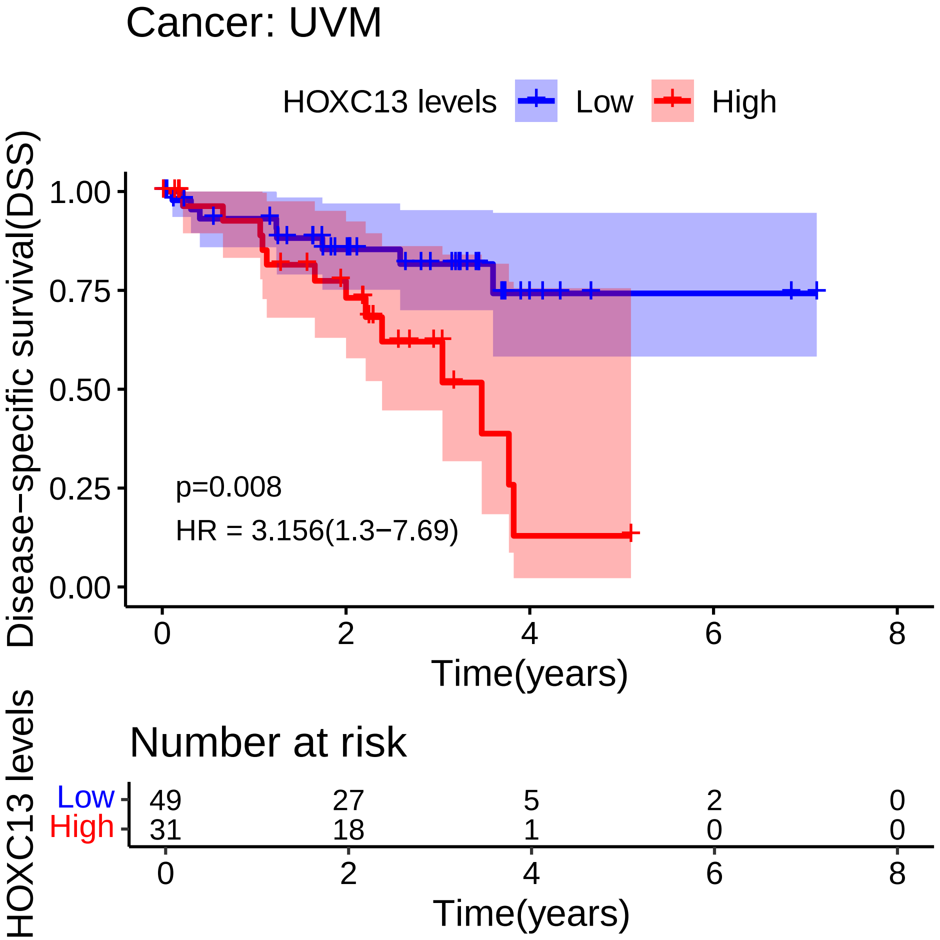

Cancer: UVM
HOXC13 levels
Low
High
Disease−specific survival(DSS)
1.00
0.75
0.50
0.25
0.00
p=0.008
HR = 3.156(1.3−7.69)
0
2
4
6
8
Time(years)
Number at risk
HOXC13 levels
Low 49 27 5 2 0
High 31 18 1 0 0
0
2
4
6
8
Time(years)

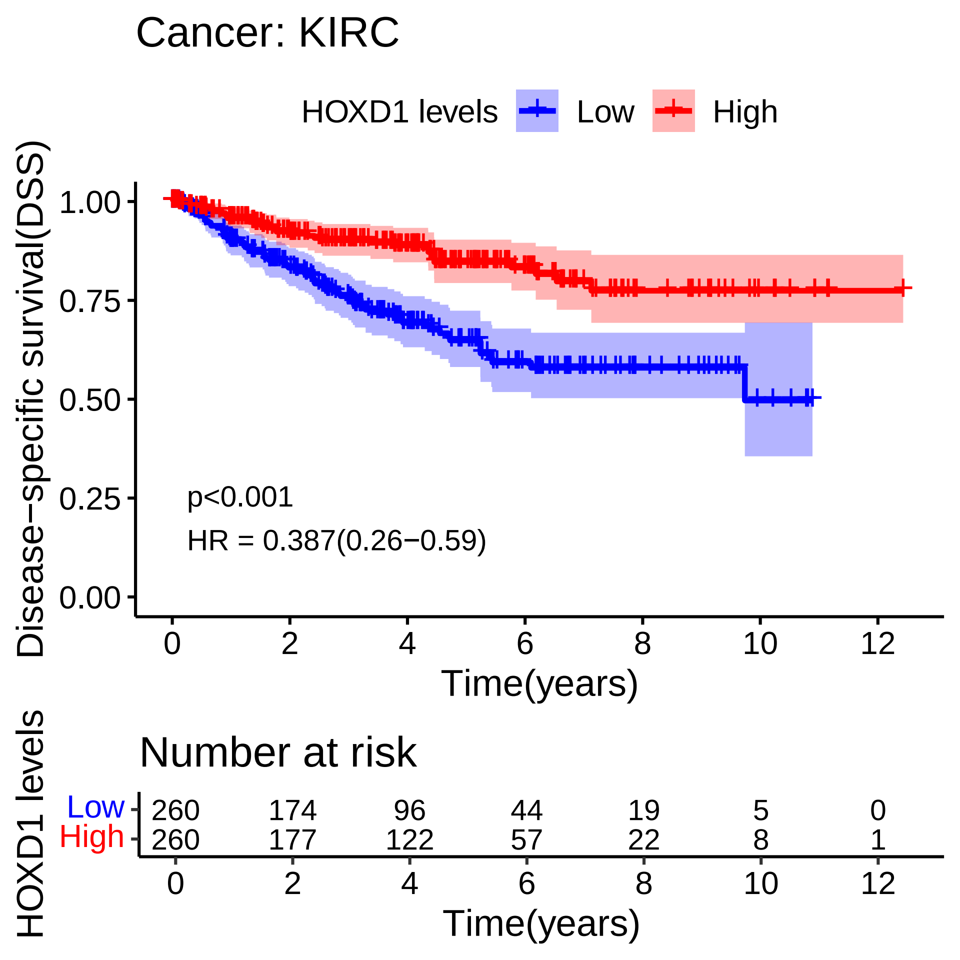

Cancer: KIRC
HOXD1 levels
Low
High
Disease−specific survival(DSS)
1.00
0.75
0.50
0.25
0.00
p<0.001
HR = 0.387(0.26−0.59)
0
2
4
6
8
10
12
Time(years)
Number at risk
HOXD1 levels
Low 260 174 96 44 19 5 0
High 260 177 122 57 22 8 1
Time(years)

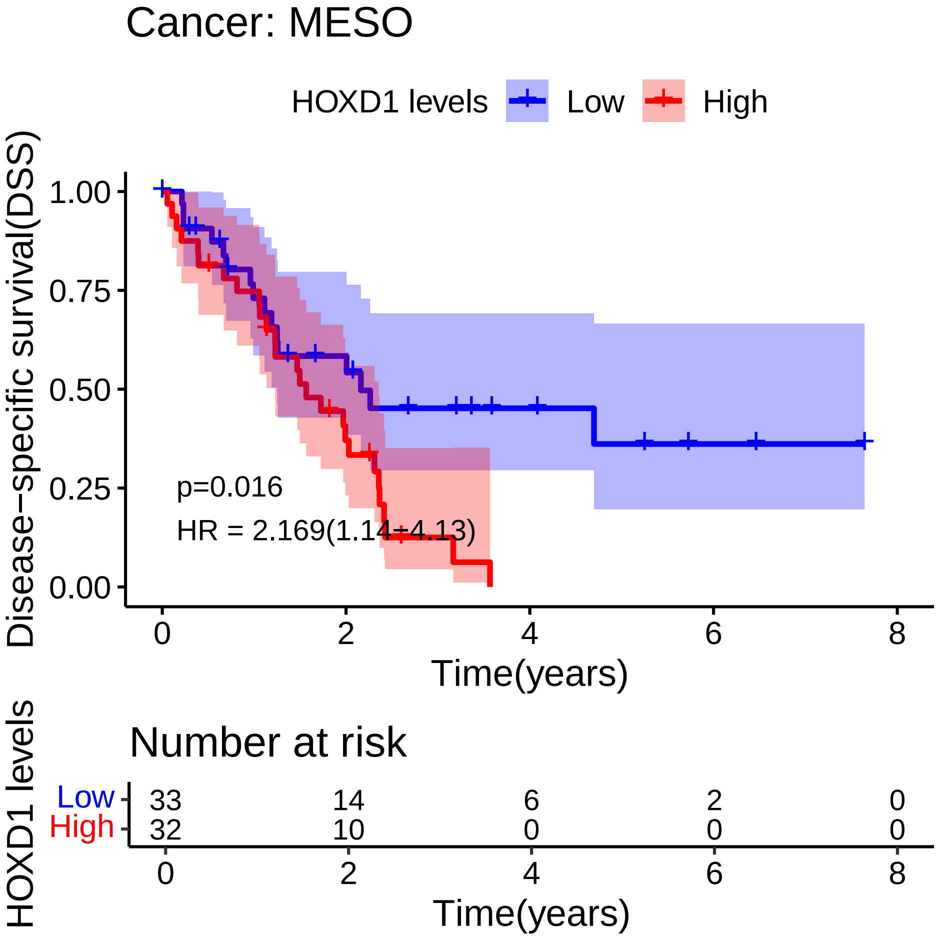

Cancer: MESO
HOXD1 levels
Low
High
Disease−specific survival(DSS)
1.00
0.75
0.50
0.25
0.00
p=0.016
HR = 2.169(1.14−4.13)
0
2
4
6
8
Time(years)
Number at risk
HOXD1 levels
Low
High
33
14
6
2
0
32
10
0
0
0
0
2
4
6
8
Time(years)

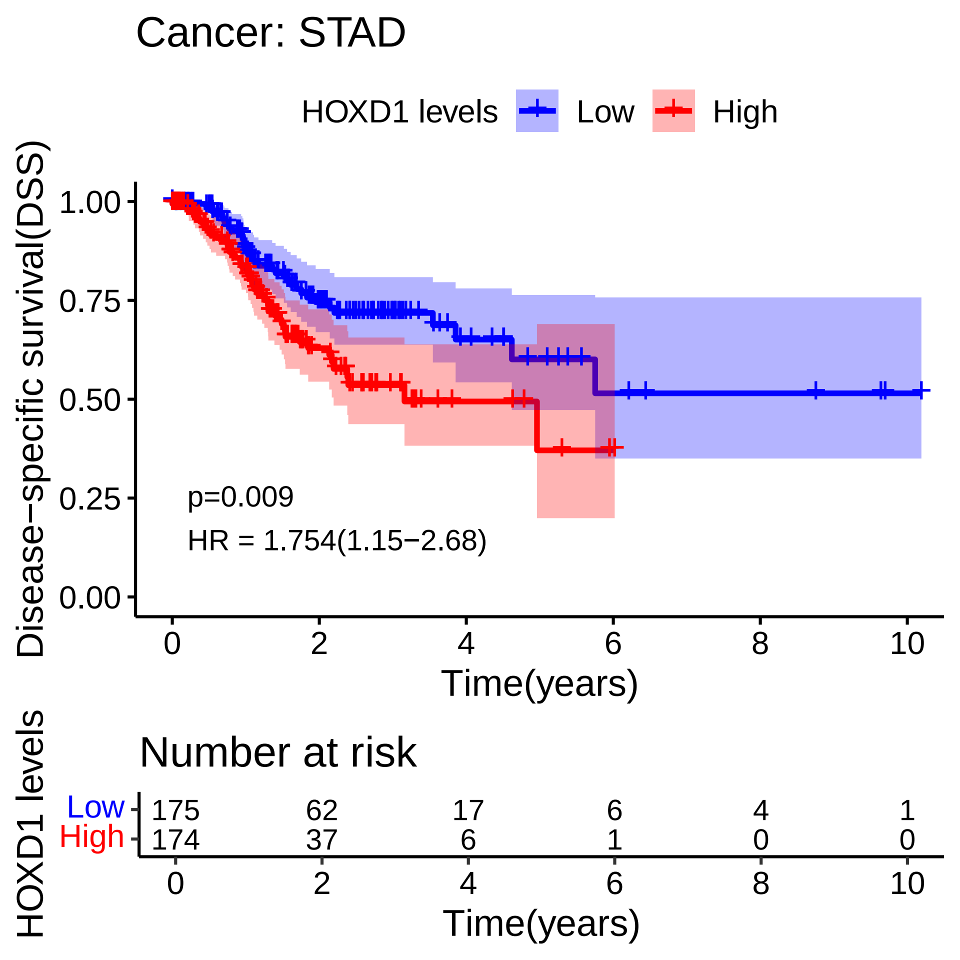

Cancer: STAD
HOXD1 levels
Low
High
Disease−specific survival(DSS)
1.00
0.75
0.50
0.25
0.00
p=0.009
HR = 1.754(1.15−2.68)
0
2
4
6
8
10
Time(years)
Number at risk
HOXD1 levels
Low 175 62 17 6 4 1
High 174 37 6 1 0 0
Time(years)

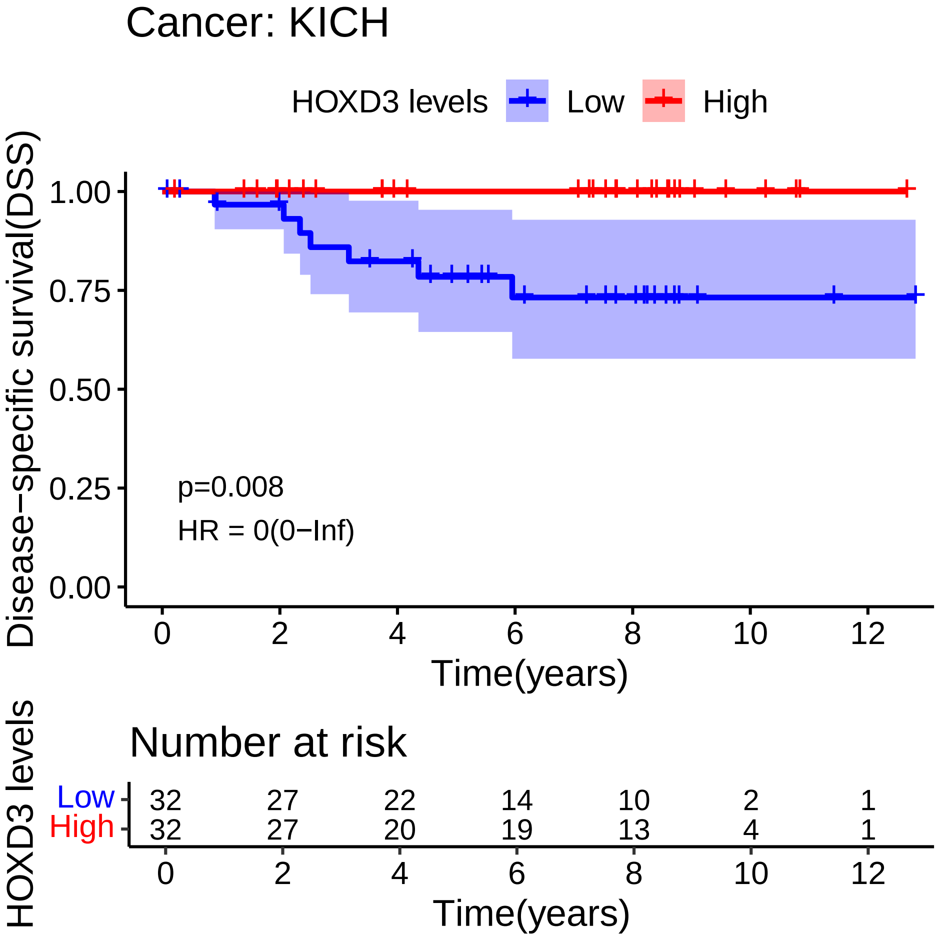

Cancer: KICH
HOXD3 levels
Low
High
Disease−specific survival(DSS)
1.00
0.75
0.50
0.25
0.00
p=0.008
HR = 0(0−Inf)
0
2
4
6
8
10
12
Time(years)
Number at risk
HOXD3 levels
Low
High
32 27 22 14 10 2 1
32 27 20 19 13 4 1
Time(years)

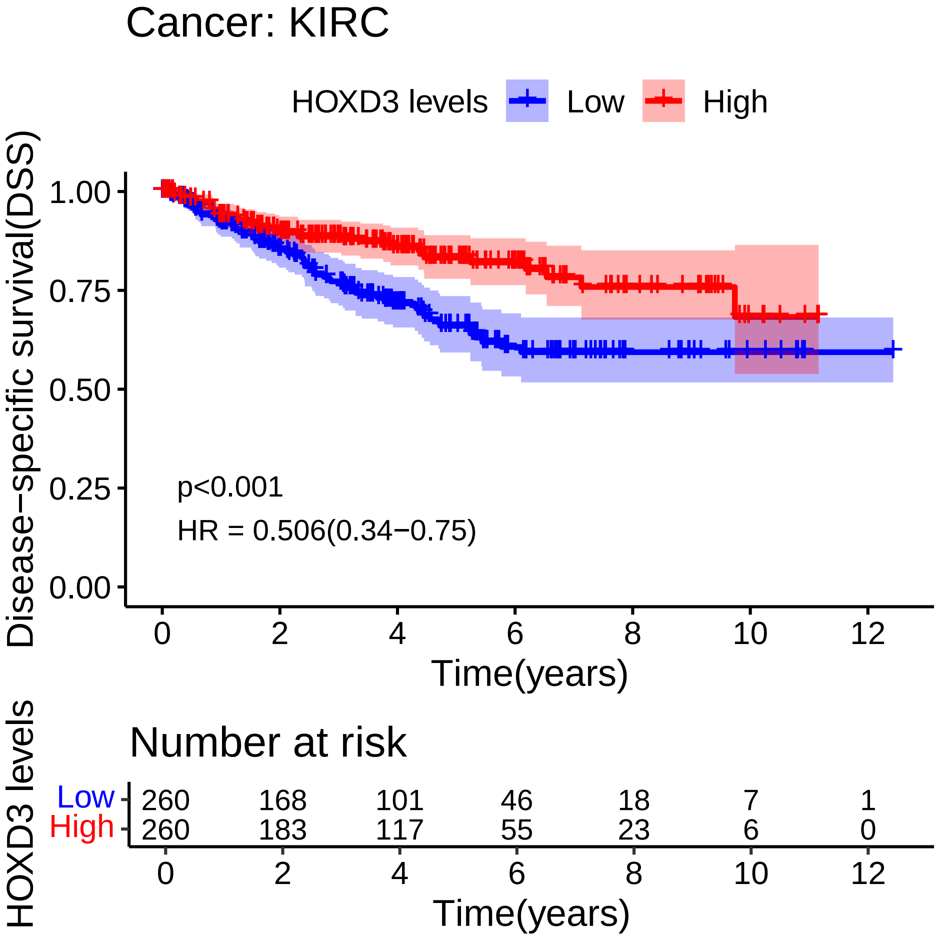

Cancer: KIRC
HOXD3 levels
Low
High
Disease−specific survival(DSS)
1.00
0.75
0.50
0.25
0.00
p<0.001
HR = 0.506(0.34−0.75)
0
2
4
6
8
10
12
Time(years)
Number at risk
HOXD3 levels
Low 260 168 101 46 18 7 1
High 260 183 117 55 23 6 0
Time(years)

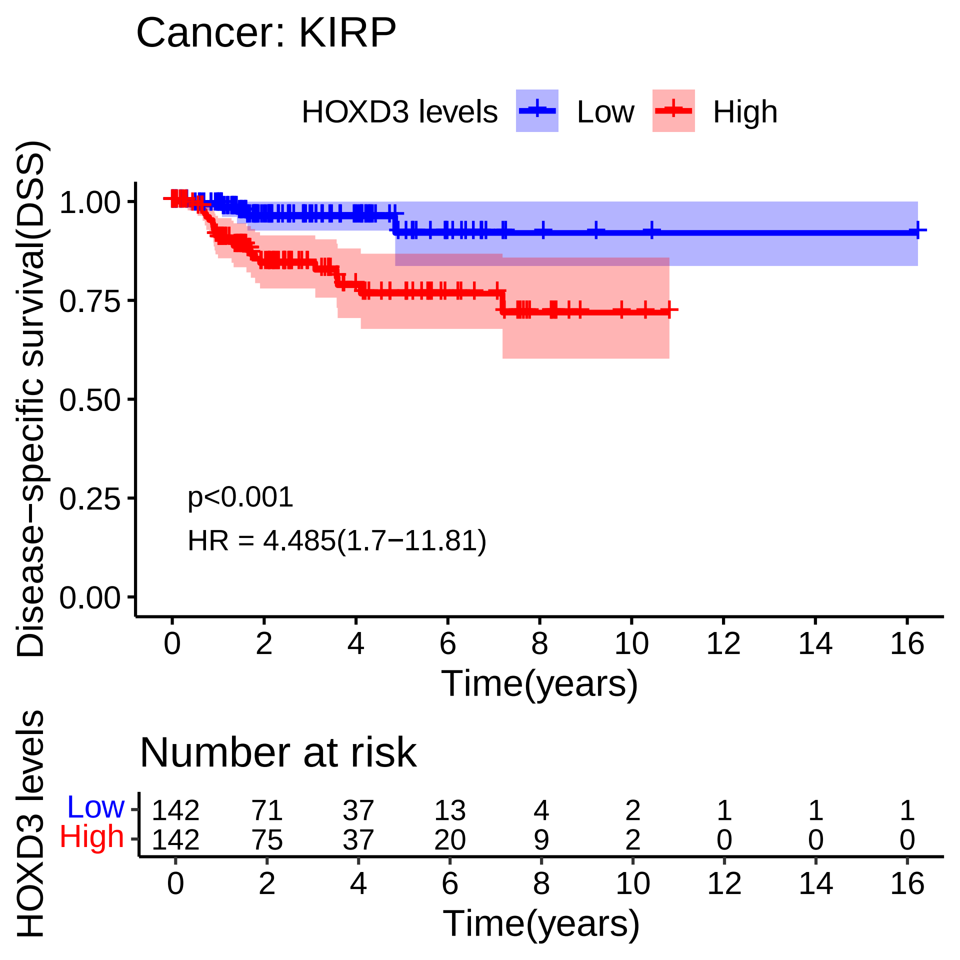

Cancer: KIRP
HOXD3 levels
Low
High
Disease−specific survival(DSS)
1.00
0.75
0.50
0.25
0.00
p<0.001
HR = 4.485(1.7−11.81)
0
2
4
6
8
10
12
14
16
Time(years)
Number at risk
HOXD3 levels
Low
High
142 71 37 13 4 2 1 1 1
142 75 37 20 9 2 0 0 0
0
2
4
6
8
10
12
14
16
Time(years)

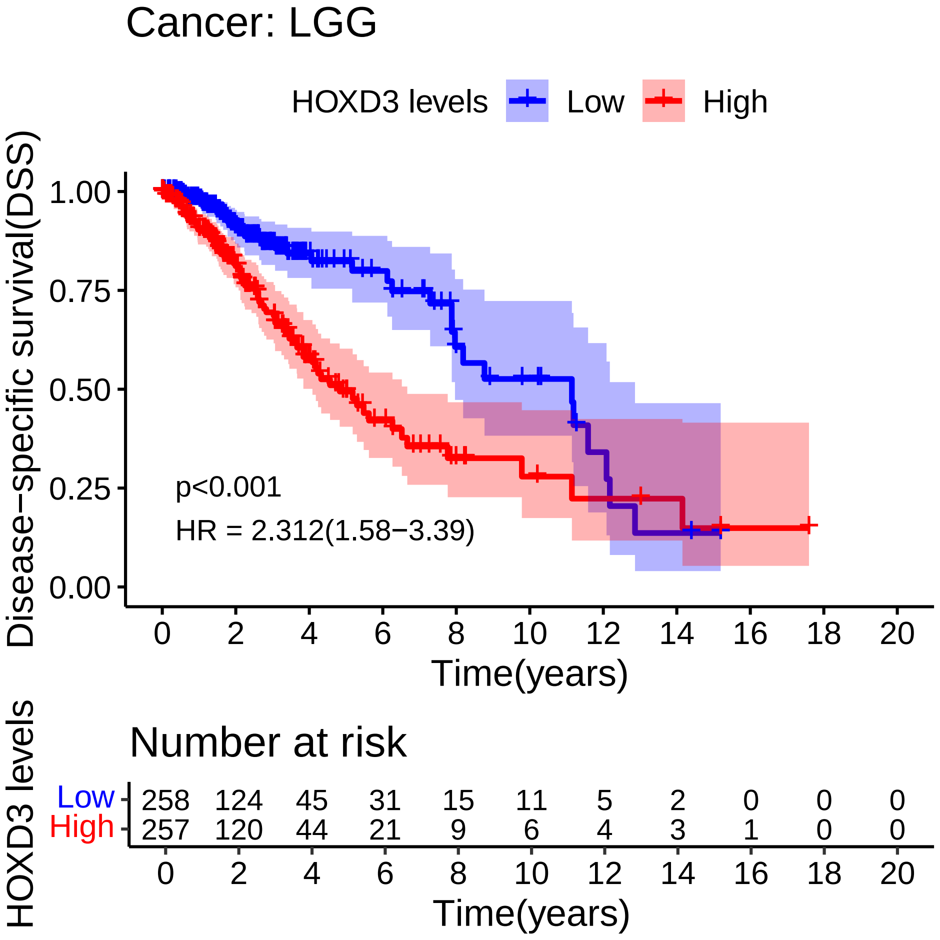
Cancer: LGG
HOXD3 levels
Low
High
Disease−specific survival(DSS)
1.00
0.75
0.50
0.25
0.00
p<0.001
HR = 2.312(1.58−3.39)
0
2
4
6
8
10
12
14
16
18
20
Time(years)
Number at risk
HOXD3 levels
Low 258 124 45 31 15 11 5 2 0 0 0
High 257 120 44 21 9 6 4 3 1 0 0
Time(years)

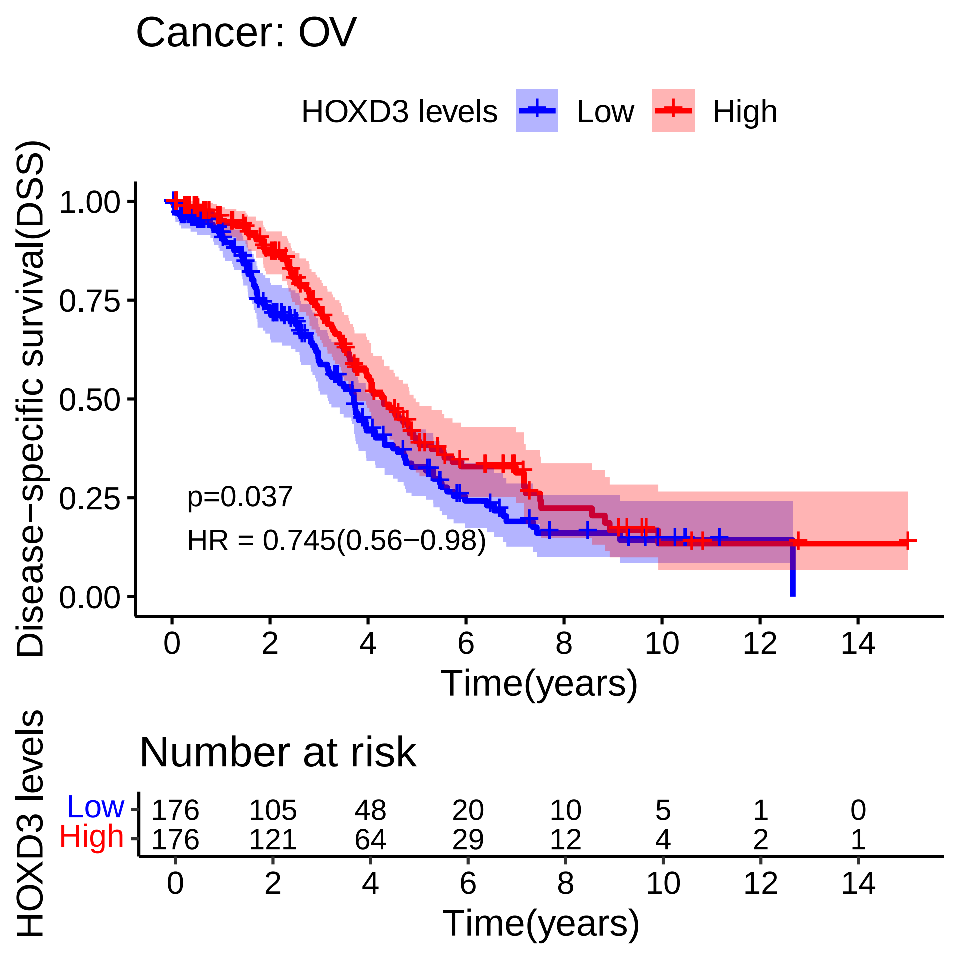

Cancer: OV
HOXD3 levels
Low
High
1.00
0.75
0.50
0.25
0.00
Disease−specific survival(DSS)
p=0.037
HR = 0.745(0.56−0.98)
0
2
4
6
8
10
12
14
Time(years)
Number at risk
HOXD3 levels
Low 176 105 48 20 10 5 1 0
High 176 121 64 29 12 4 2 1
0
2
4
6
8
10
12
14
Time(years)

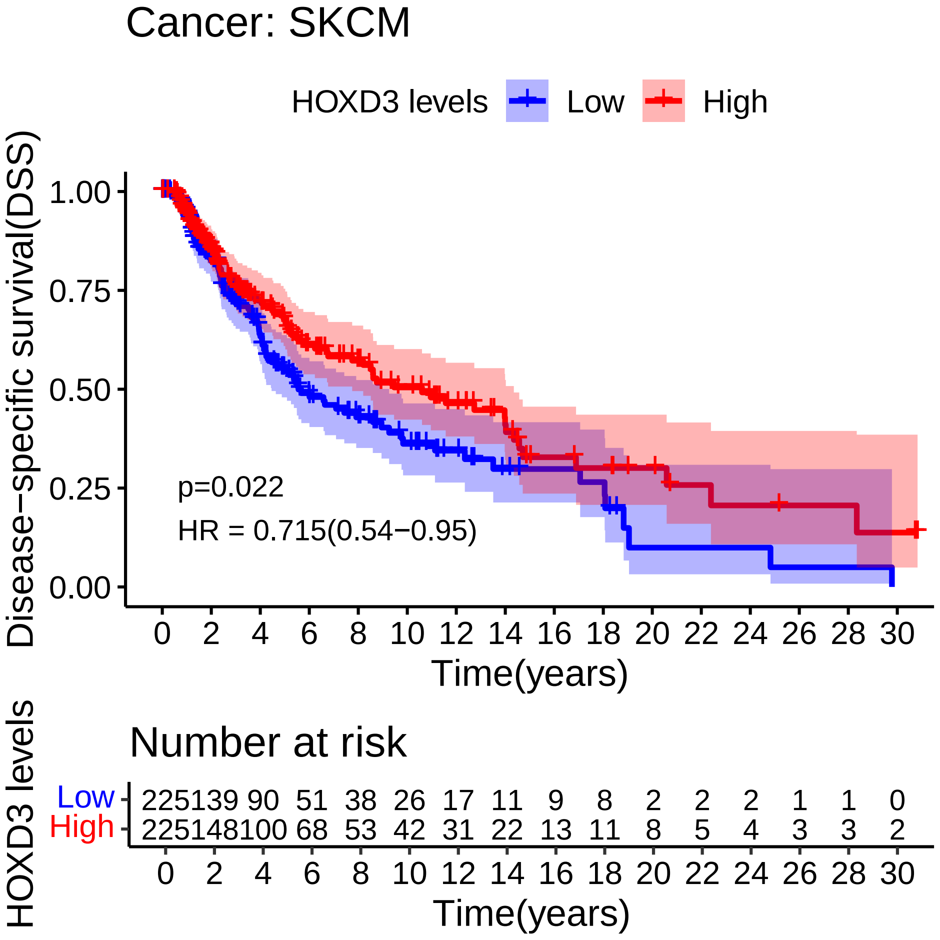
Cancer: SKCM
HOXD3 levels
Low
High
Disease−specific survival(DSS)
1.00
0.75
0.50
0.25
0.00
p=0.022
HR = 0.715(0.54−0.95)
0 2 4 6 8 10 12 14 16 18 20 22 24 26 28 30
Time(years)
Number at risk
HOXD3 levels
Low 225 139 90 51 38 26 17 11 9 8 2 2 2 1 1 0
High 225 148 100 68 53 42 31 22 13 11 8 5 4 3 3 2
0 2 4 6 8 10 12 14 16 18 20 22 24 26 28 30
Time(years)

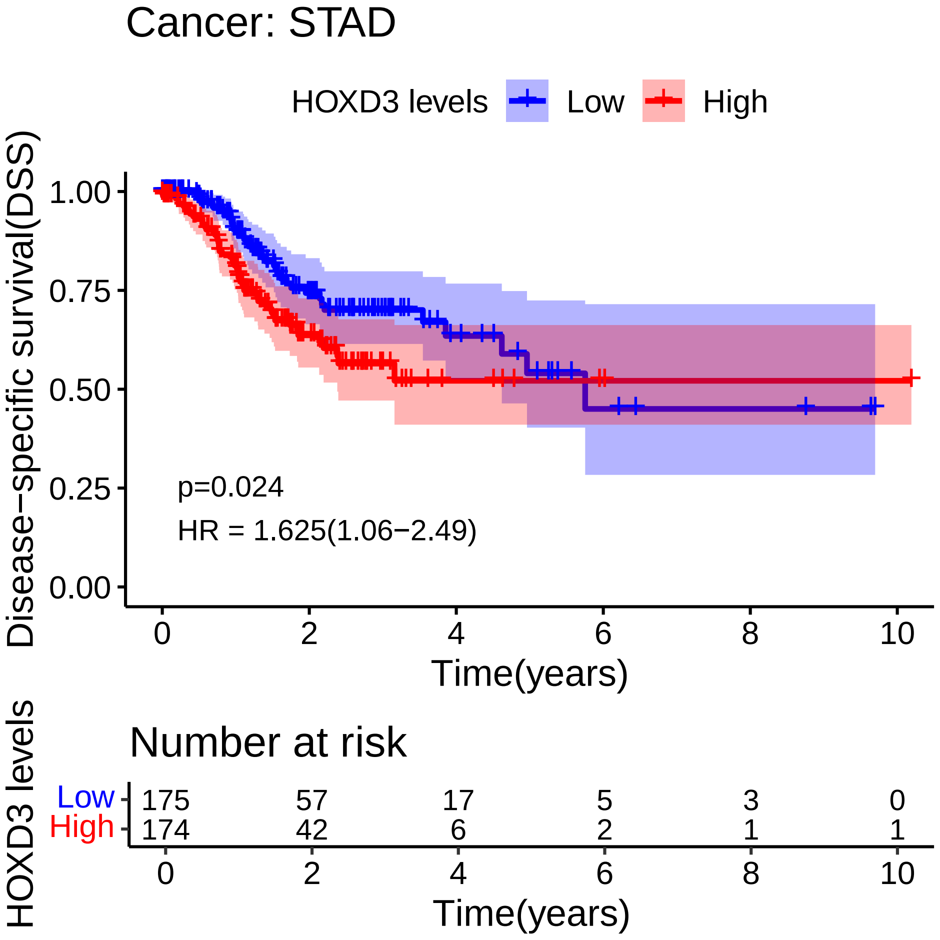
Cancer: STAD
HOXD3 levels
Low
High
Disease−specific survival(DSS)
1.00
0.75
0.50
0.25
0.00
p=0.024
HR = 1.625(1.06−2.49)
0
2
4
6
8
10
Time(years)
Number at risk
HOXD3 levels
Low 175 57 17 5 3 0
High 174 42 6 2 1 1
0
2
4
6
8
10
Time(years)

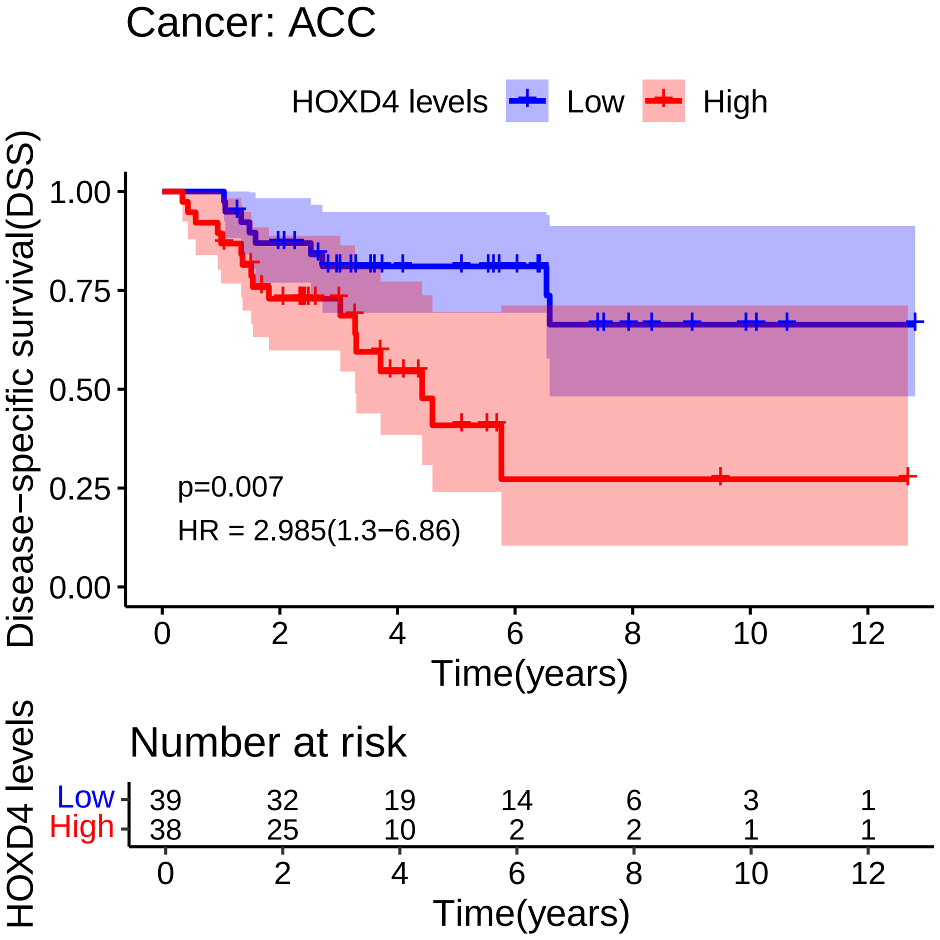
Cancer: ACC
HOXD4 levels
Low
High
Disease−specific survival(DSS)
1.00
0.75
0.50
0.25
0.00
p=0.007
HR = 2.985(1.3−6.86)
0
2
4
6
8
10
12
Time(years)
Number at risk
HOXD4 levels
Low 39 32 19 14 6 3 1
High 38 25 10 2 2 1 1
0
2
4
6
8
10
12
Time(years)

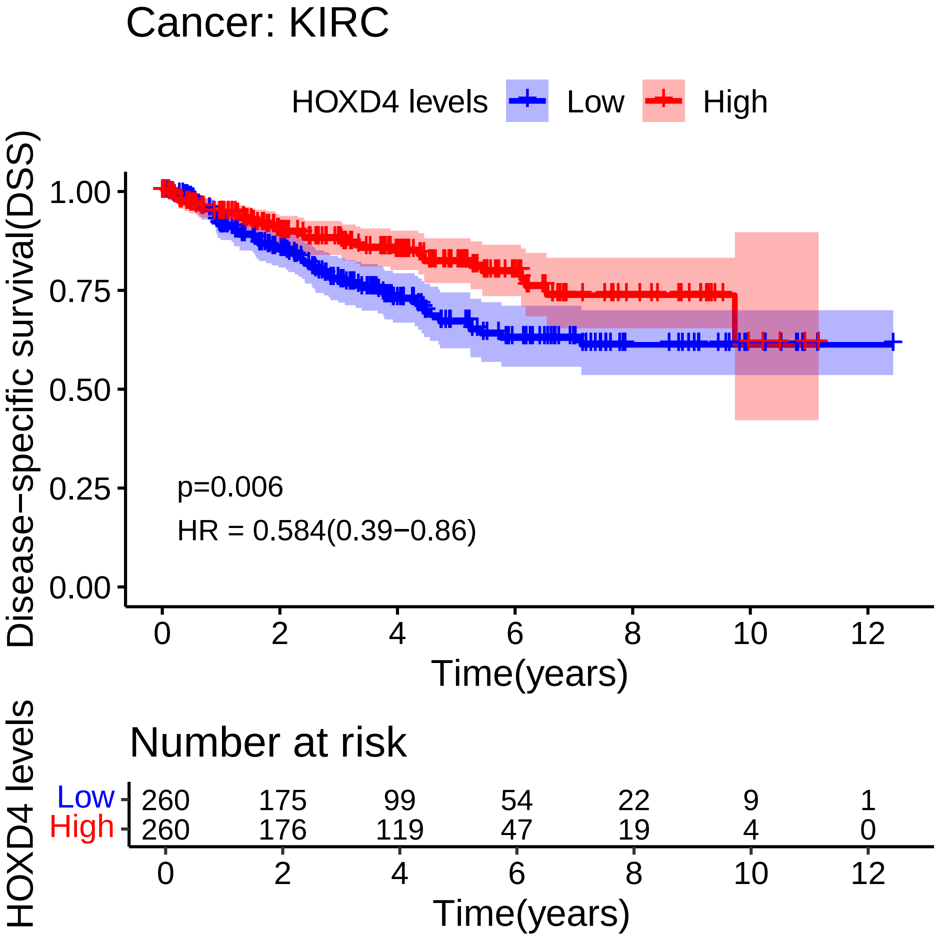

Cancer: KIRC
HOXD4 levels
Low
High
Disease−specific survival(DSS)
1.00
0.75
0.50
0.25
0.00
p=0.006
HR = 0.584(0.39−0.86)
0
2
4
6
8
10
12
Time(years)
Number at risk
HOXD4 levels
Low 260 175 99 54 22 9 1
High 260 176 119 47 19 4 0
0
2
4
6
8
10
12
Time(years)

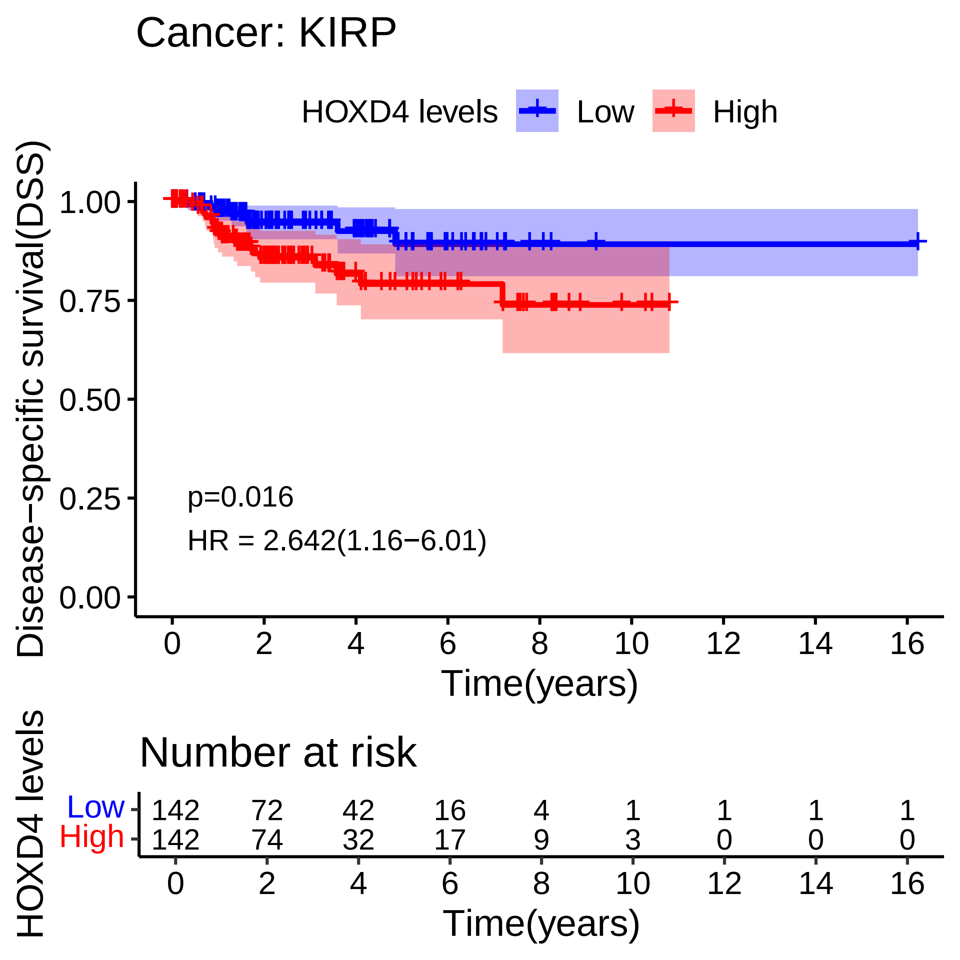

Cancer: KIRP
HOXD4 levels
Low
High
Disease−specific survival(DSS)
1.00
0.75
0.50
0.25
0.00
p=0.016
HR = 2.642(1.16−6.01)
0
2
4
6
8
10
12
14
16
Time(years)
Number at risk
HOXD4 levels
Low 142 72 42 16 4 1 1 1 1
High 142 74 32 17 9 3 0 0 0
Time(years)

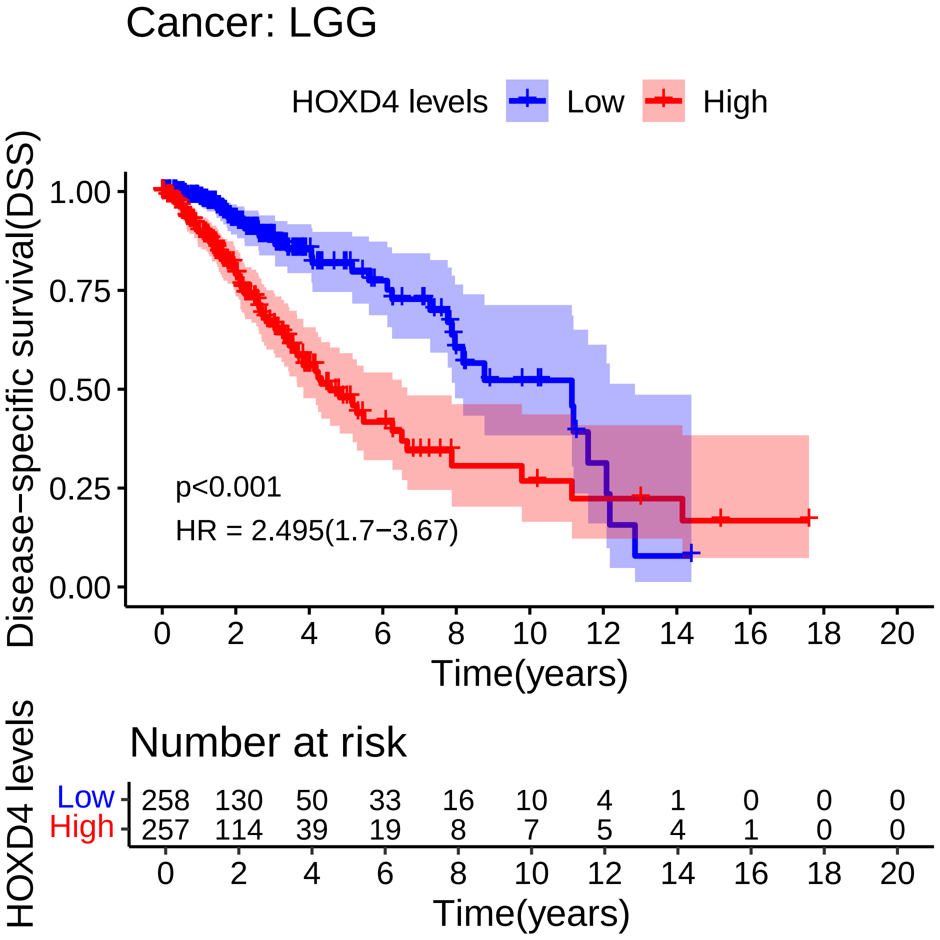

Cancer: LGG
HOXD4 levels
Low
High
Disease−specific survival(DSS)
1.00
0.75
0.50
0.25
0.00
p<0.001
HR = 2.495(1.7−3.67)
0 2 4 6 8 10 12 14 16 18 20
Time(years)
Number at risk
HOXD4 levels
Low 258 130 50 33 16 10 4 1 0 0 0
High 257 114 39 19 8 7 5 4 1 0 0
0 2 4 6 8 10 12 14 16 18 20
Time(years)

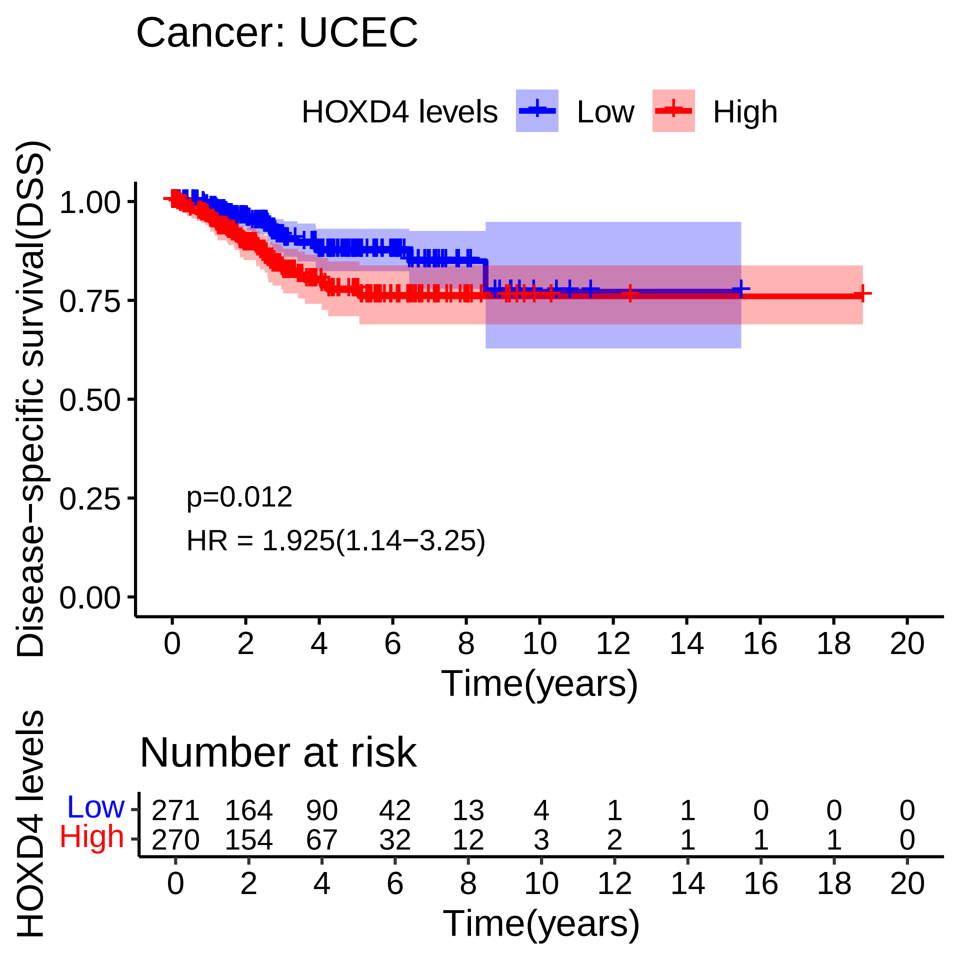

Cancer: UCEC
HOXD4 levels
Low
High
Disease−specific survival(DSS)
1.00
0.75
0.50
0.25
0.00
p=0.012
HR = 1.925(1.14−3.25)
0 2 4 6 8 10 12 14 16 18 20
Time(years)
Number at risk
HOXD4 levels
Low 271 164 90 42 13 4 1 1 0 0 0
High 270 154 67 32 12 3 2 1 1 1 0
0 2 4 6 8 10 12 14 16 18 20
Time(years)

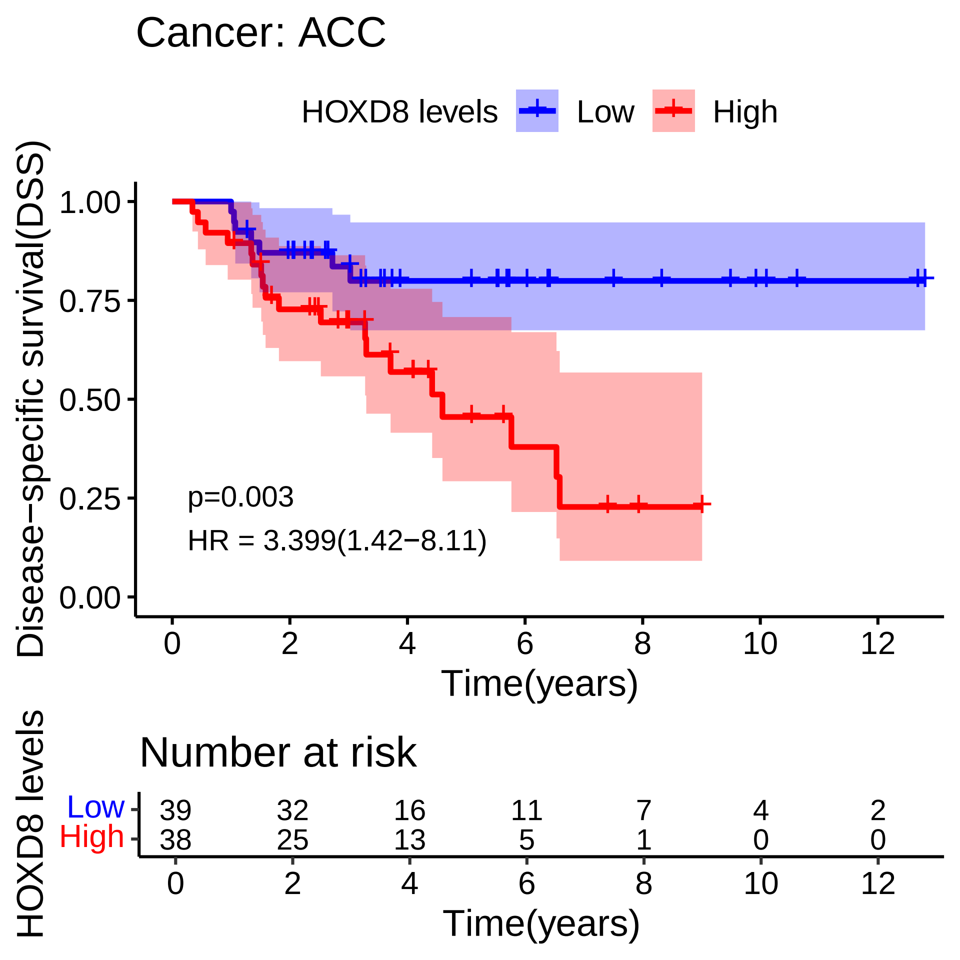

Cancer: ACC
HOXD8 levels
Low
High
Disease−specific survival(DSS)
1.00
0.75
0.50
0.25
0.00
p=0.003
HR = 3.399(1.42−8.11)
0
2
4
6
8
10
12
Time(years)
Number at risk
HOXD8 levels
Low 39 32 16 11 7 4 2
High 38 25 13 5 1 0 0
Time(years)

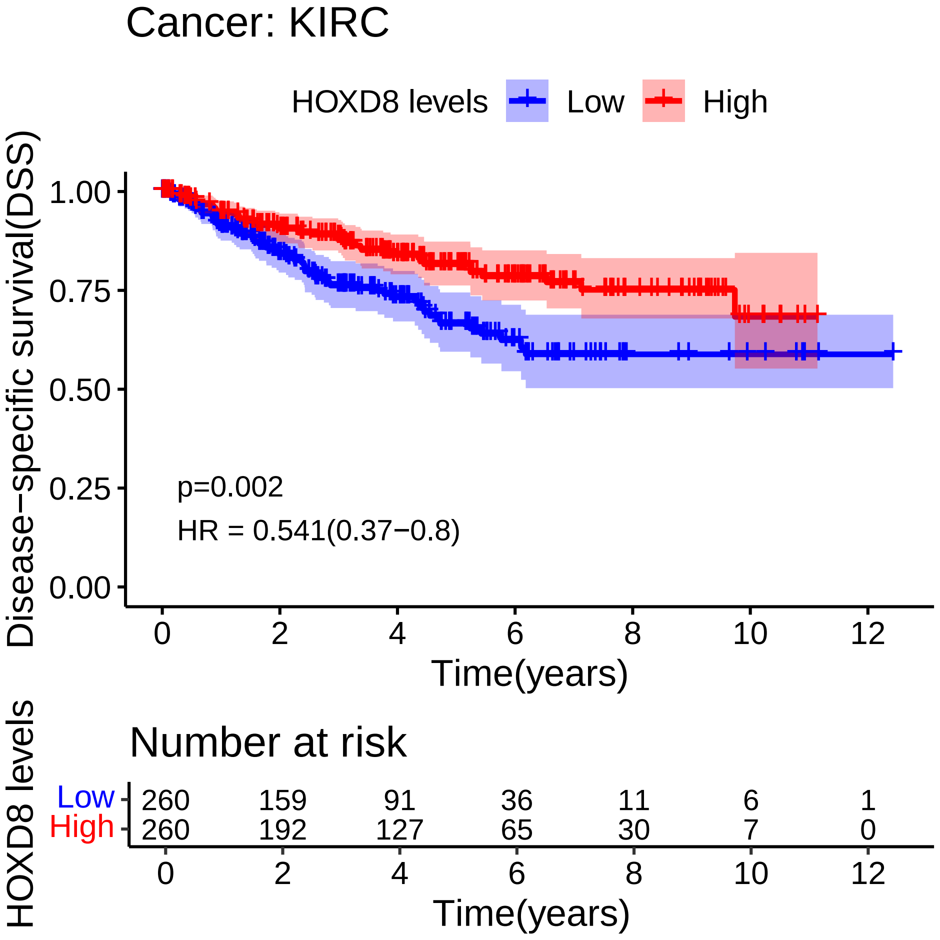

Cancer: KIRC
HOXD8 levels
Low
High
Disease−specific survival(DSS)
1.00
0.75
0.50
0.25
0.00
p=0.002
HR = 0.541(0.37−0.8)
0
2
4
6
8
10
12
Time(years)
Number at risk
HOXD8 levels
Low
High
260 159 91 36 11 6 1
260 192 127 65 30 7 0
0
2
4
6
8
10
12
Time(years)

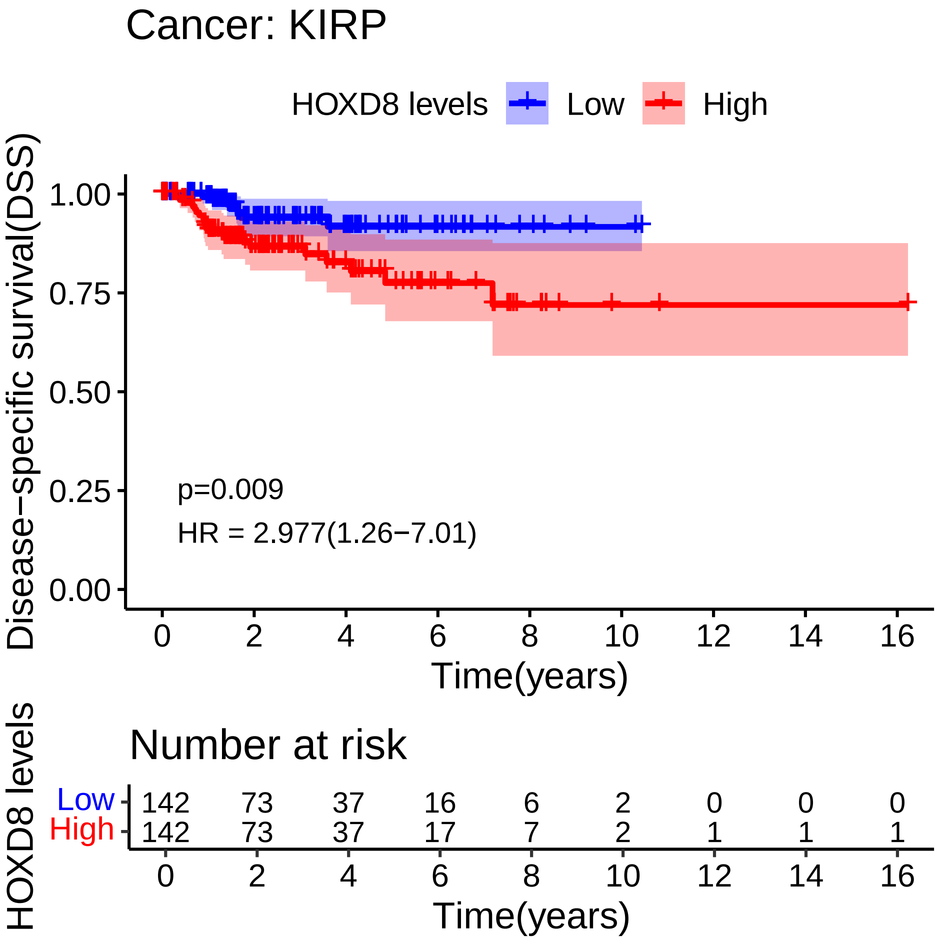

Cancer: KIRP
HOXD8 levels
Low
High
Disease−specific survival(DSS)
1.00
0.75
0.50
0.25
0.00
p=0.009
HR = 2.977(1.26−7.01)
0
2
4
6
8
10
12
14
16
Time(years)
Number at risk
HOXD8 levels
Low 142 73 37 16 6 2 0 0 0
High 142 73 37 17 7 2 1 1 1
0
2
4
6
8
10
12
14
16
Time(years)

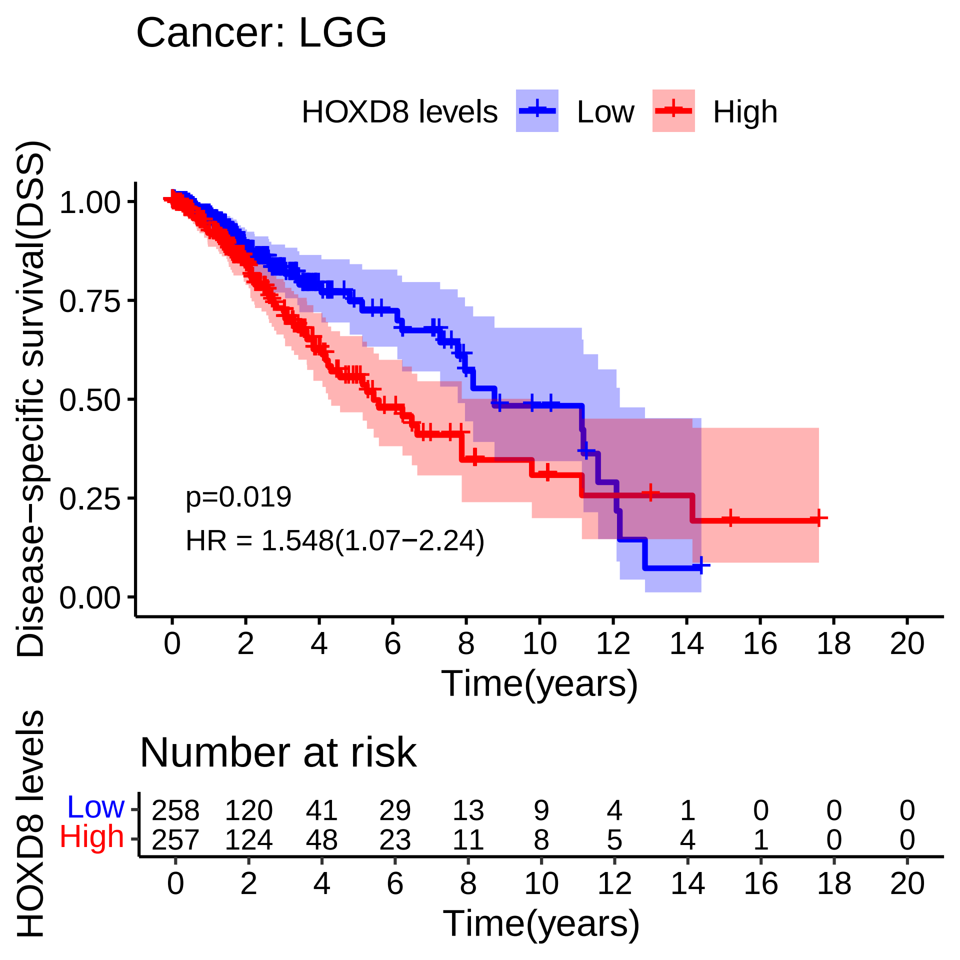

Cancer: LGG
HOXD8 levels
Low
High
Disease−specific survival(DSS)
1.00
0.75
0.50
0.25
0.00
p=0.019
HR = 1.548(1.07−2.24)
0
2
4
6
8
10
12
14
16
18
20
Time(years)
Number at risk
HOXD8 levels
Low 258 120 41 29 13 9 4 1 0 0 0
High 257 124 48 23 11 8 5 4 1 0 0
Time(years)

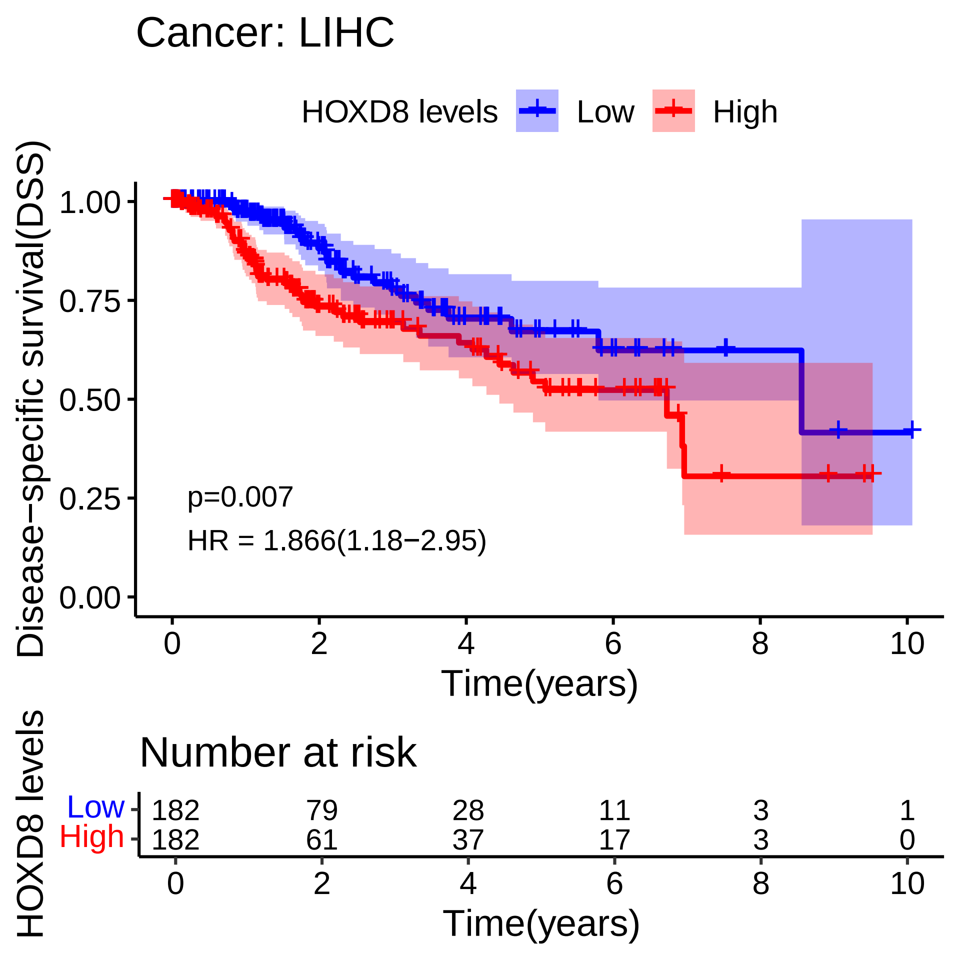

Cancer: LIHC
HOXD8 levels
Low
High
Disease−specific survival(DSS)
1.00
0.75
0.50
0.25
0.00
p=0.007
HR = 1.866(1.18−2.95)
0
2
4
6
8
10
Time(years)
Number at risk
HOXD8 levels
Low 182 79 28 11 3 1
High 182 61 37 17 3 0
0
2
4
6
8
10
Time(years)

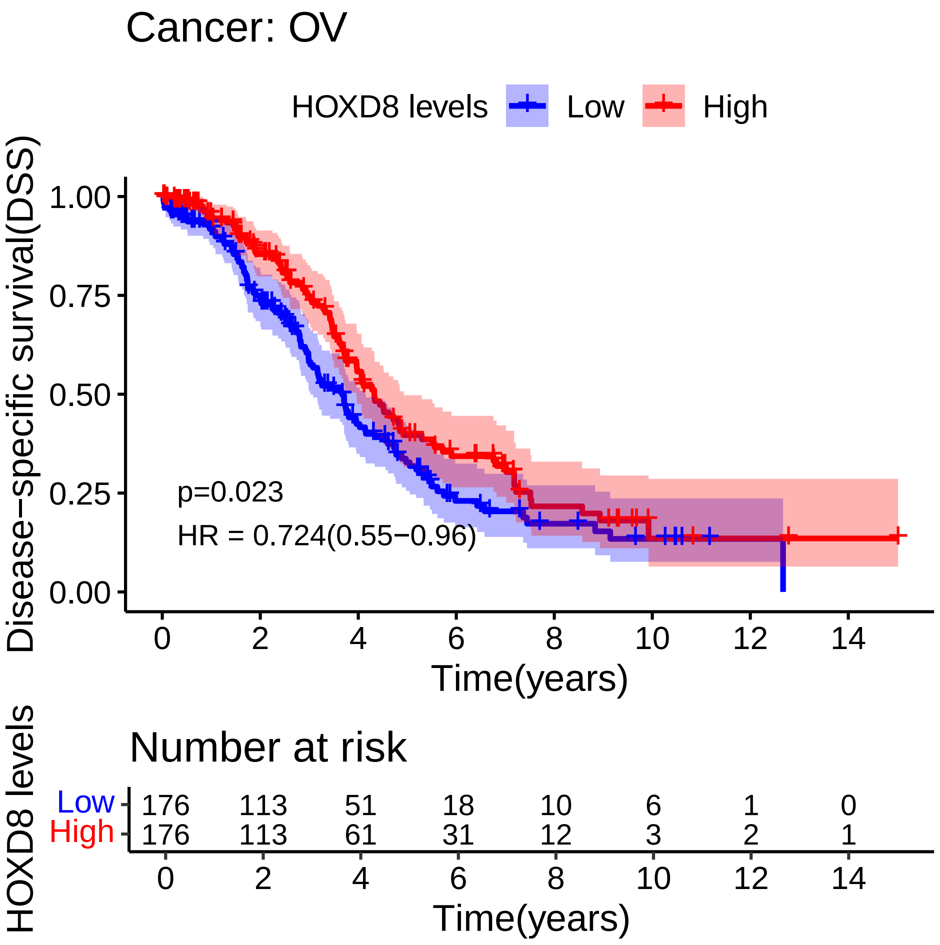

Cancer: OV
HOXD8 levels
Low
High
Disease−specific survival(DSS)
1.00
0.75
0.50
0.25
0.00
p=0.023
HR = 0.724(0.55−0.96)
0
2
4
6
8
10
12
14
Time(years)
Number at risk
HOXD8 levels
Low 176 113 51 18 10 6 1 0
High 176 113 61 31 12 3 2 1
0
2
4
6
8
10
12
14
Time(years)

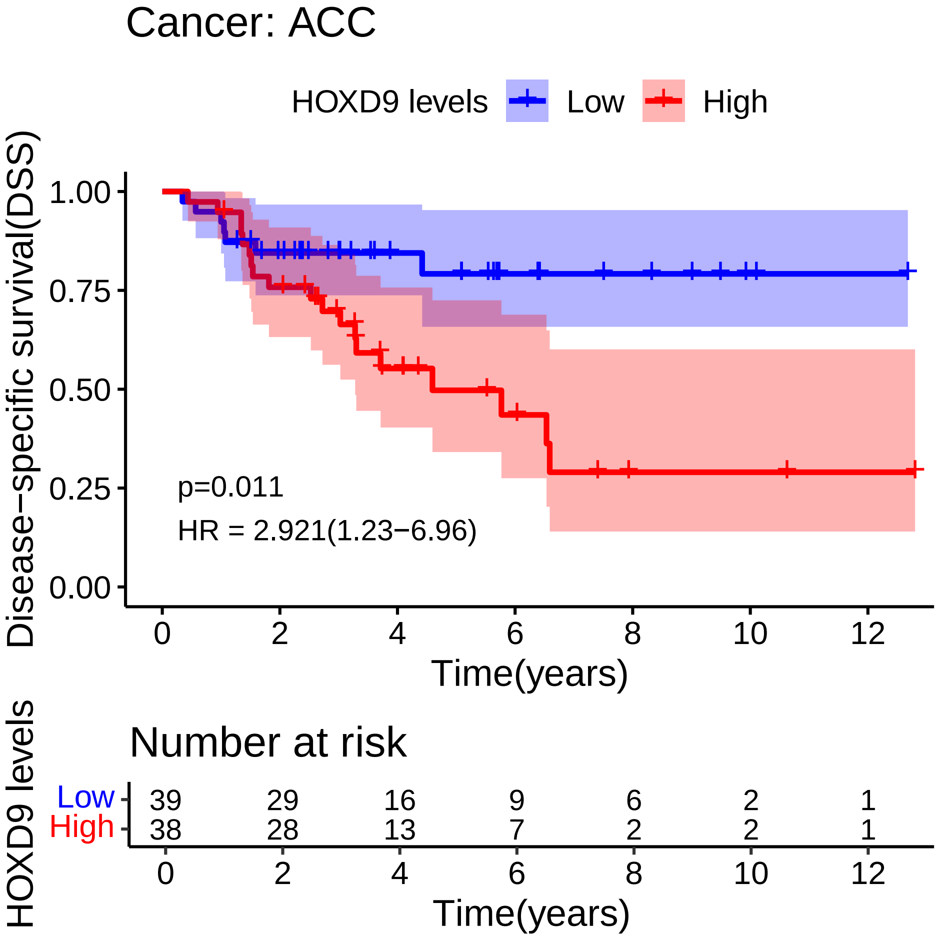

Cancer: ACC
HOXD9 levels
Low
High
Disease−specific survival(DSS)
1.00
0.75
0.50
0.25
0.00
p=0.011
HR = 2.921(1.23−6.96)
0
2
4
6
8
10
12
Time(years)
Number at risk
HOXD9 levels
Low 39 29 16 9 6 2 1
High 38 28 13 7 2 2 1
0
2
4
6
8
10
12
Time(years)

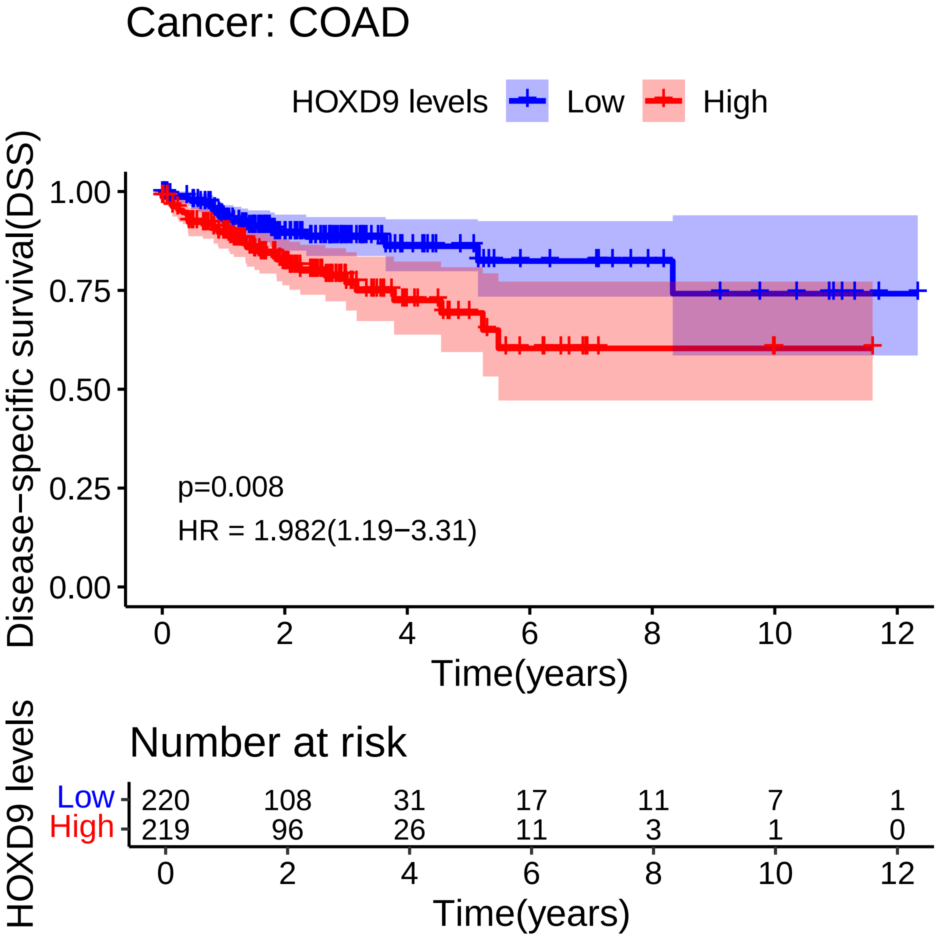

Cancer: COAD
HOXD9 levels
Low
High
Disease−specific survival(DSS)
1.00
0.75
0.50
0.25
0.00
p=0.008
HR = 1.982(1.19−3.31)
0
2
4
6
8
10
12
Time(years)
Number at risk
HOXD9 levels
Low 220 108 31 17 11 7 1
High 219 96 26 11 3 1 0
Time(years)

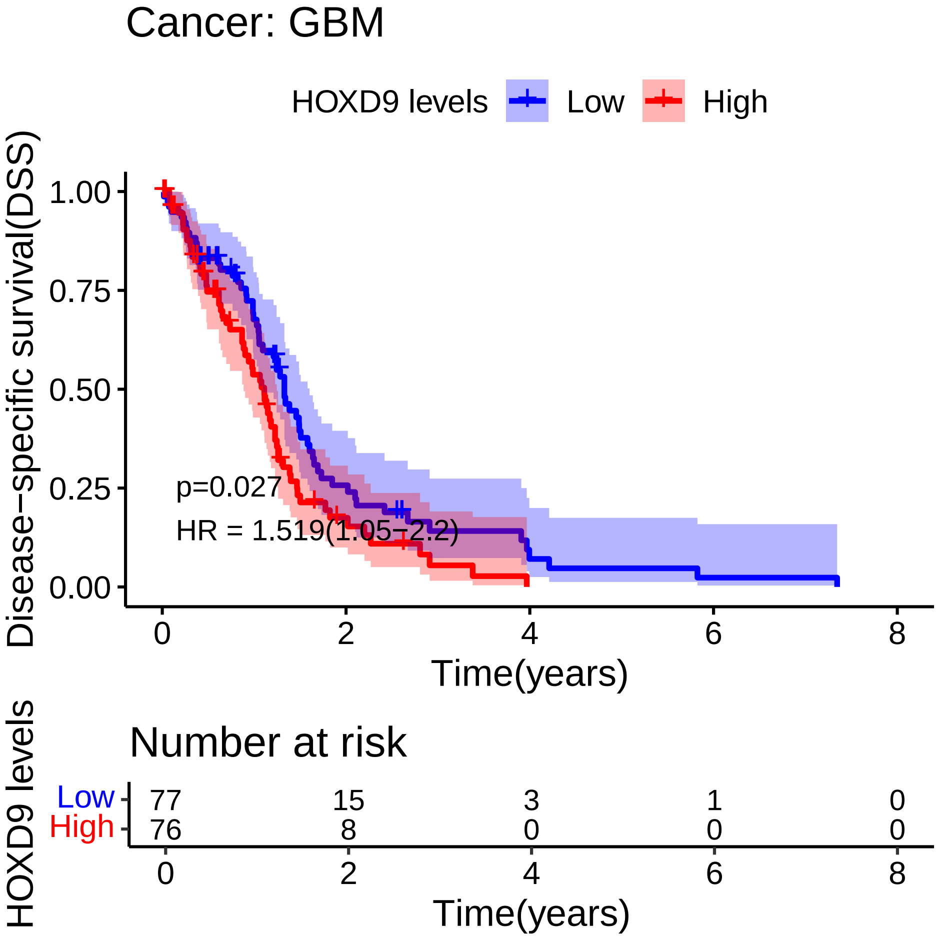

Cancer: GBM
HOXD9 levels
Low
High
Disease−specific survival(DSS)
1.00
0.75
0.50
0.25
0.00
p=0.027
HR = 1.519(1.05−2.2)
0
2
4
6
8
Time(years)
Number at risk
HOXD9 levels
Low
High
77
15
3
1
0
76
8
0
0
0
0
2
4
6
8
Time(years)

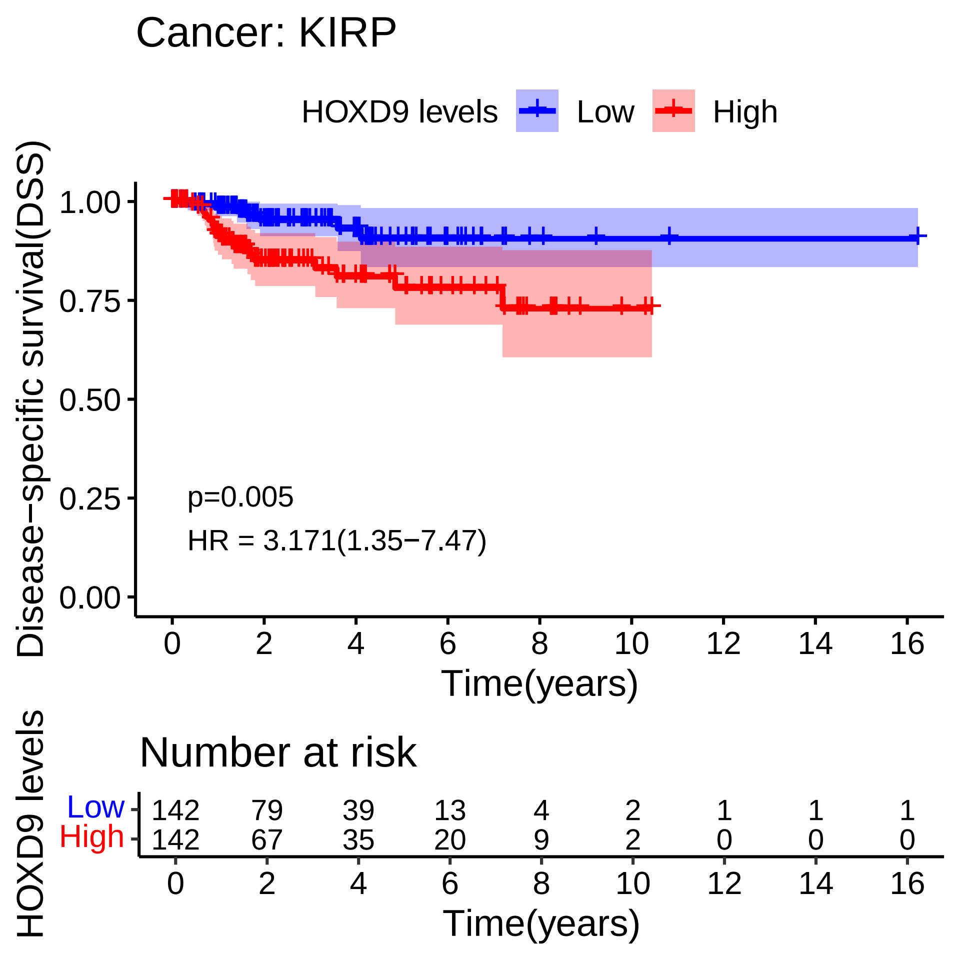

Cancer: KIRP
HOXD9 levels
Low
High
Disease−specific survival(DSS)
1.00
0.75
0.50
0.25
0.00
p=0.005
HR = 3.171(1.35−7.47)
0
2
4
6
8
10
12
14
16
Time(years)
Number at risk
HOXD9 levels
Low
142
79
39
13
4
2
1
1
1
High
142
67
35
20
9
2
0
0
0
Time(years)

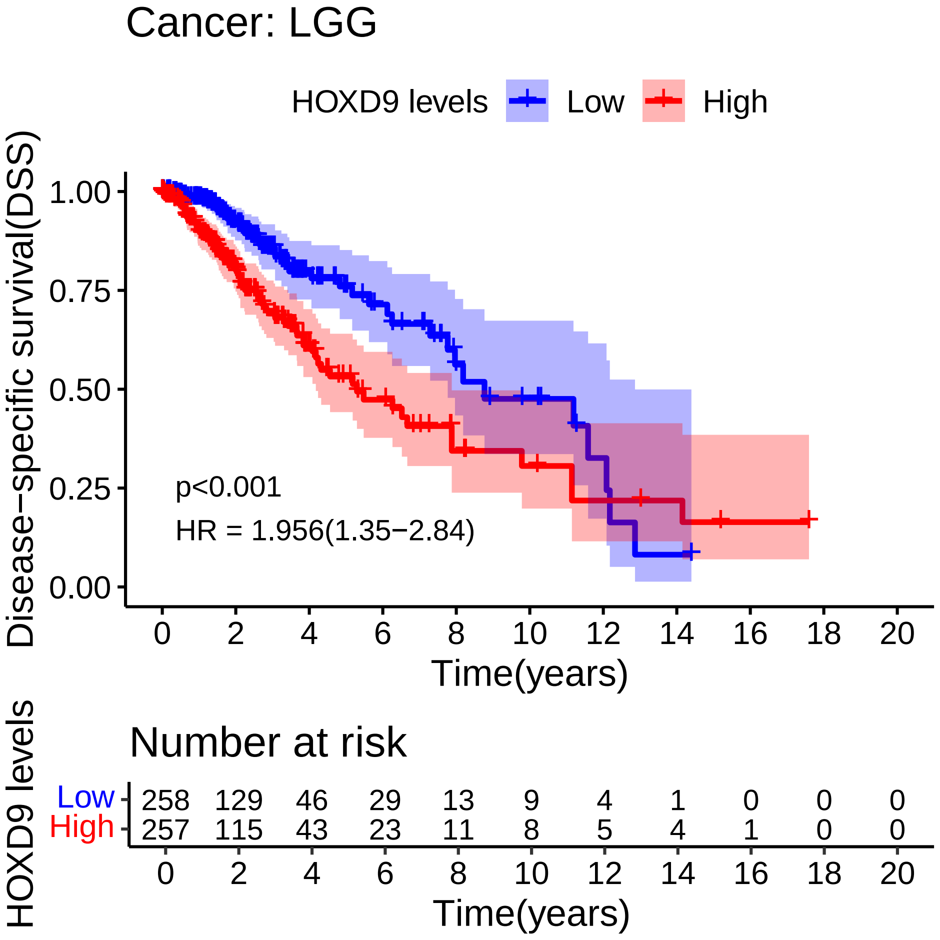
Cancer: LGG
HOXD9 levels
Low
High
Disease−specific survival(DSS)
1.00
0.75
0.50
0.25
0.00
p<0.001
HR = 1.956(1.35−2.84)
0 2 4 6 8 10 12 14 16 18 20
Time(years)
Number at risk
HOXD9 levels
Low 258 129 46 29 13 9 4 1 0 0 0
High 257 115 43 23 11 8 5 4 1 0 0
0 2 4 6 8 10 12 14 16 18 20
Time(years)

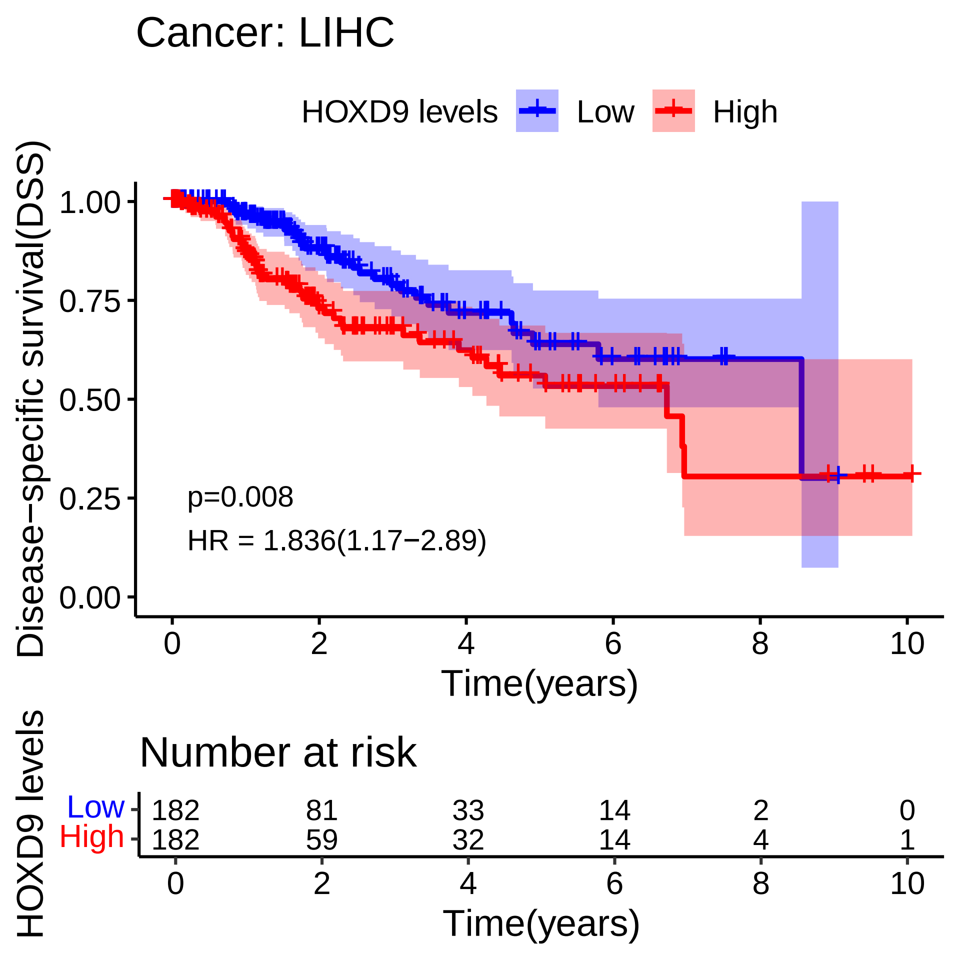
Cancer: LIHC
HOXD9 levels
Low
High
Disease−specific survival(DSS)
1.00
0.75
0.50
0.25
0.00
p=0.008
HR = 1.836(1.17−2.89)
0
2
4
6
8
10
Time(years)
Number at risk
HOXD9 levels
Low
High
182 81 33 14 2 0
182 59 32 14 4 1
Time(years)

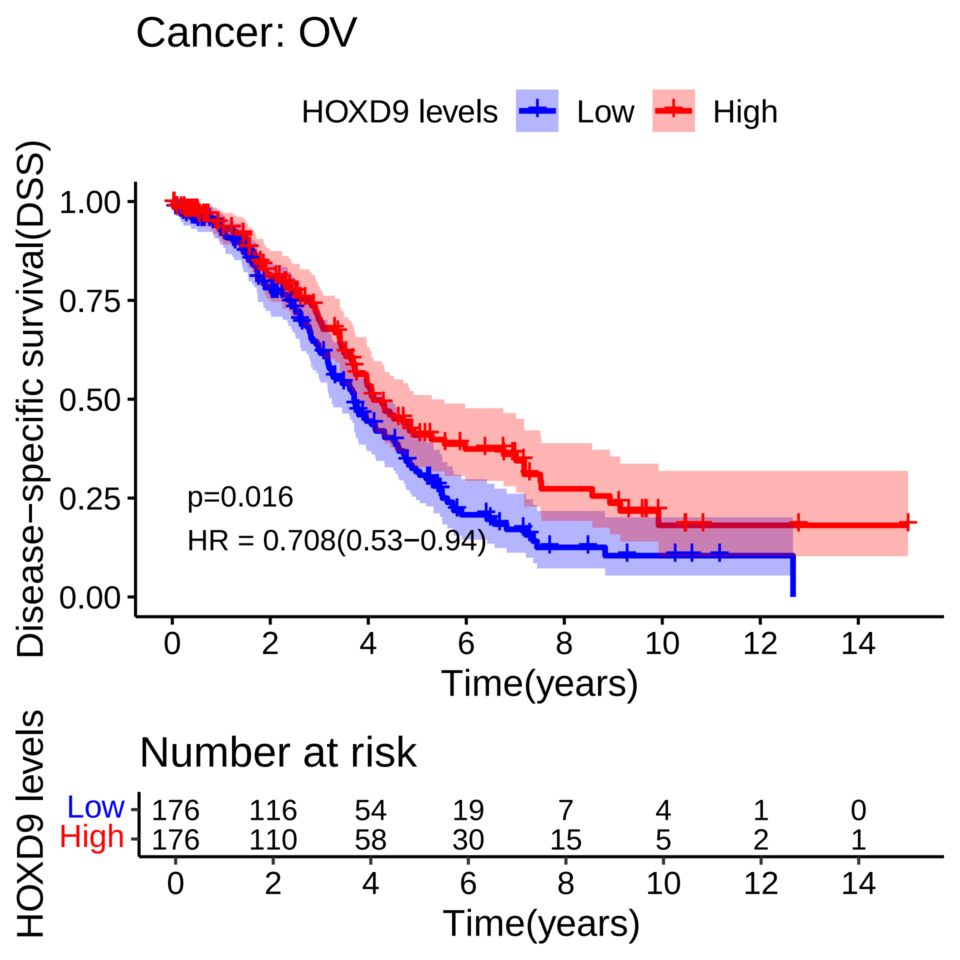

Cancer: OV
HOXD9 levels
Low
High
Disease−specific survival(DSS)
1.00
0.75
0.50
0.25
0.00
p=0.016
HR = 0.708(0.53−0.94)
0
2
4
6
8
10
12
14
Time(years)
Number at risk
HOXD9 levels
Low 176 116 54 19 7 4 1 0
High 176 110 58 30 15 5 2 1
0
2
4
6
8
10
12
14
Time(years)

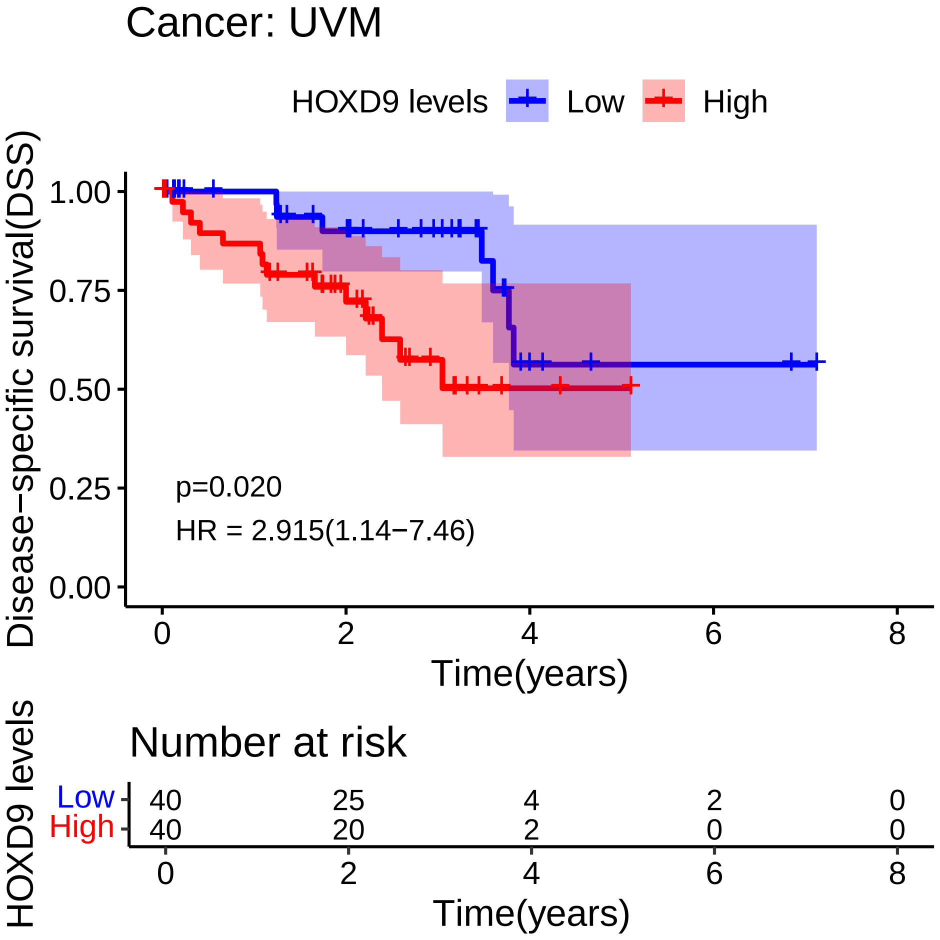

Cancer: UVM
HOXD9 levels
Low
High
Disease−specific survival(DSS)
1.00
0.75
0.50
0.25
0.00
p=0.020
HR = 2.915(1.14−7.46)
0
2
4
6
8
Time(years)
Number at risk
HOXD9 levels
Low 40 25 4 2 0
High 40 20 2 0 0
0
2
4
6
8
Time(years)

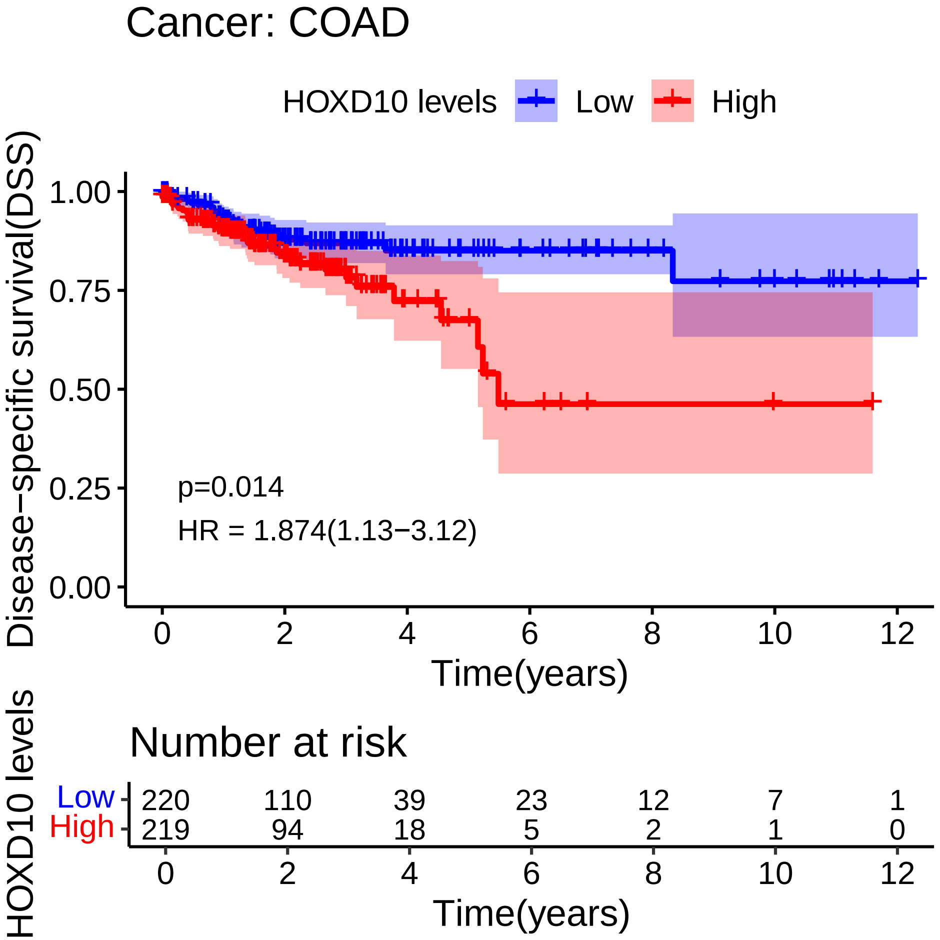

Cancer: COAD
HOXD10 levels
Low
High
Disease−specific survival(DSS)
1.00
0.75
0.50
0.25
0.00
p=0.014
HR = 1.874(1.13−3.12)
0
2
4
6
8
10
12
Time(years)
Number at risk
HOXD10 levels
Low
High
220 110 39 23 12 7 1
219 94 18 5 2 1 0
Time(years)

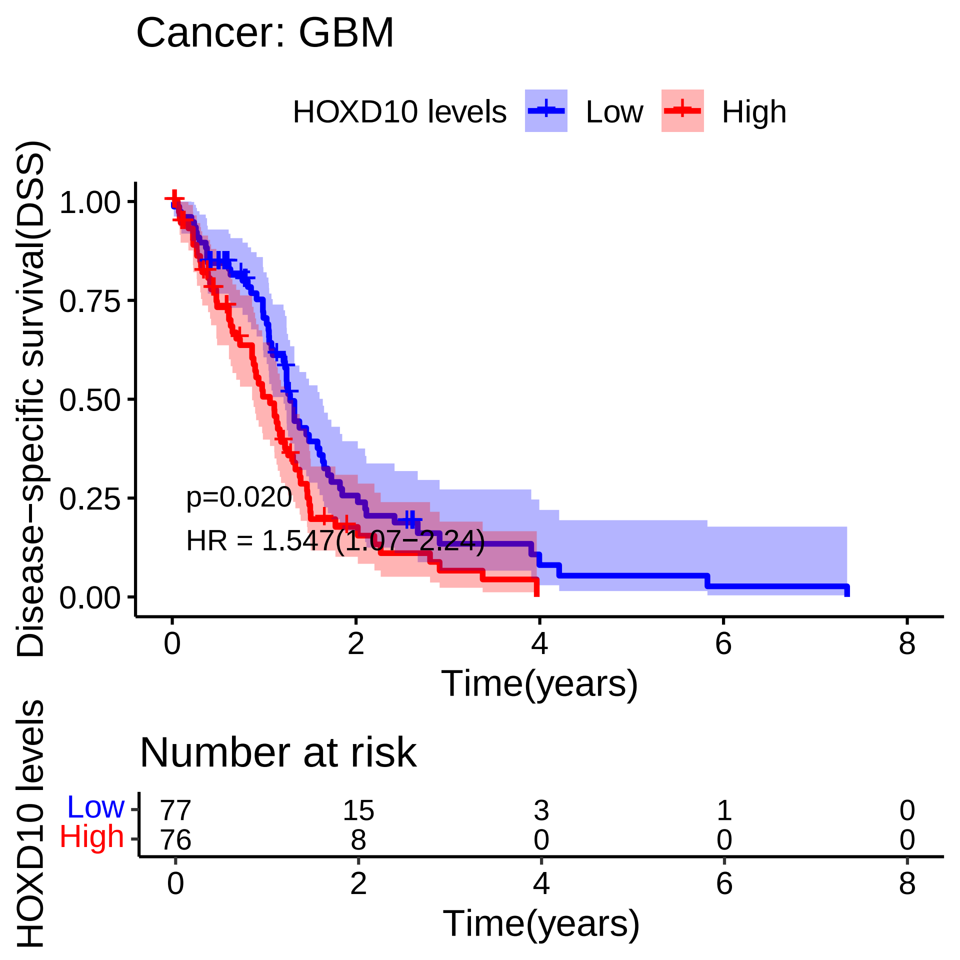

Cancer: GBM
HOXD10 levels
Low
High
Disease−specific survival(DSS)
1.00
0.75
0.50
0.25
0.00
p=0.020
HR = 1.547(1.07−2.24)
0
2
4
6
8
Time(years)
Number at risk
HOXD10 levels
Low
High
77
15
3
1
0
76
8
0
0
0
0
2
4
6
8
Time(years)

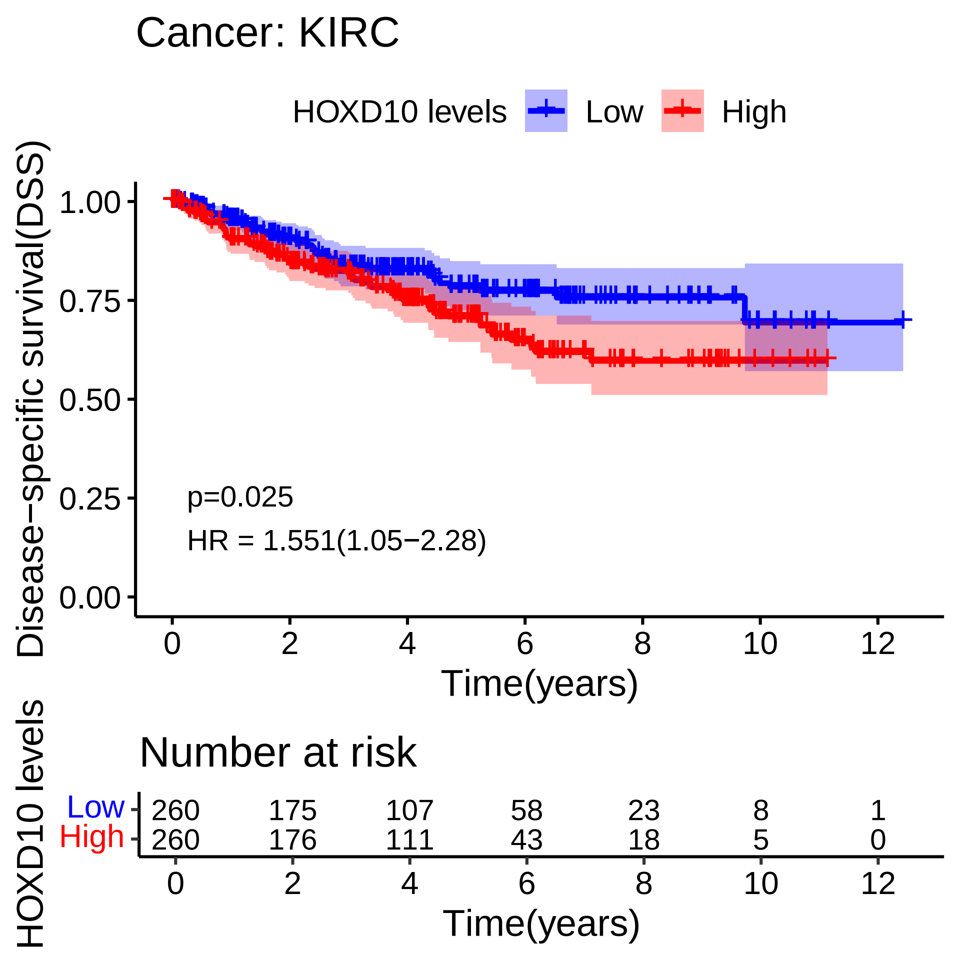

Cancer: KIRC
HOXD10 levels
Low
High
Disease−specific survival(DSS)
1.00
0.75
0.50
0.25
0.00
p=0.025
HR = 1.551(1.05−2.28)
0
2
4
6
8
10
12
Time(years)
Number at risk
HOXD10 levels
Low 260 175 107 58 23 8 1
High 260 176 111 43 18 5 0
Time(years)

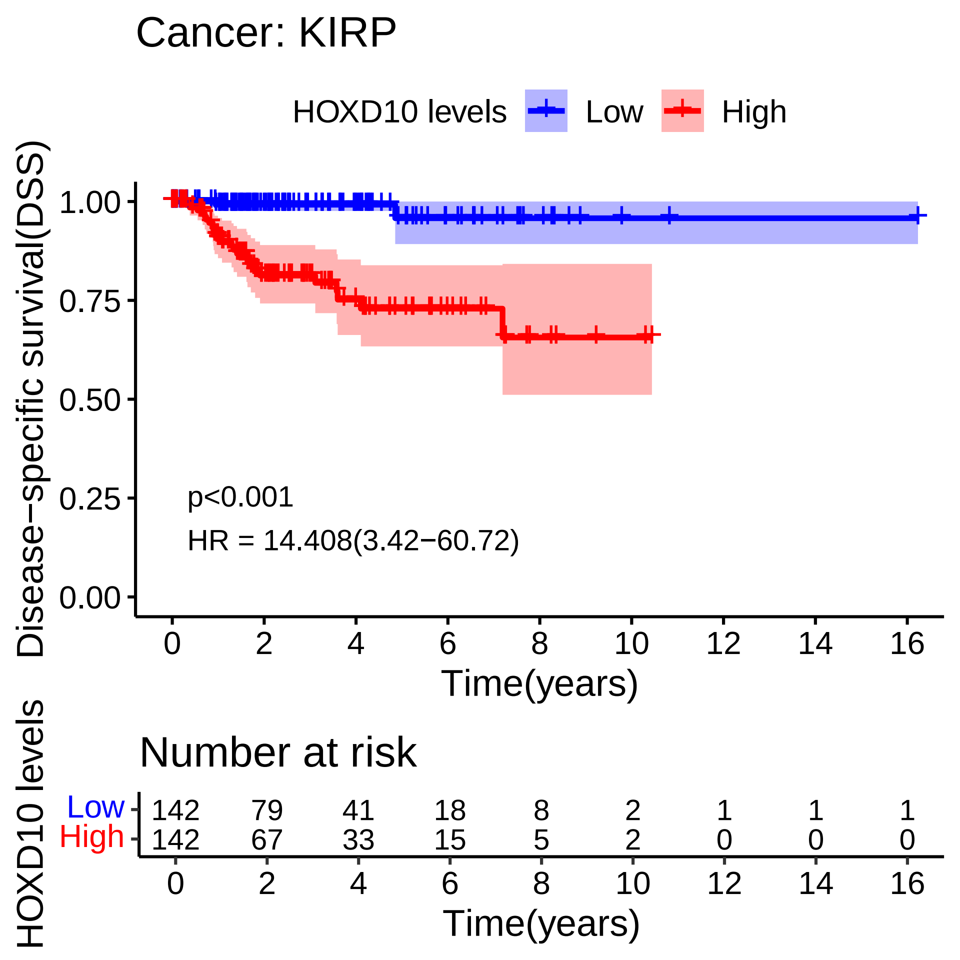
Cancer: KIRP
HOXD10 levels
Low
High
Disease−specific survival(DSS)
1.00
0.75
0.50
0.25
0.00
p<0.001
HR = 14.408(3.42−60.72)
0
2
4
6
8
10
12
14
16
Time(years)
Number at risk
HOXD10 levels
Low 142 79 41 18 8 2 1 1 1
High 142 67 33 15 5 2 0 0 0
0
2
4
6
8
10
12
14
16
Time(years)

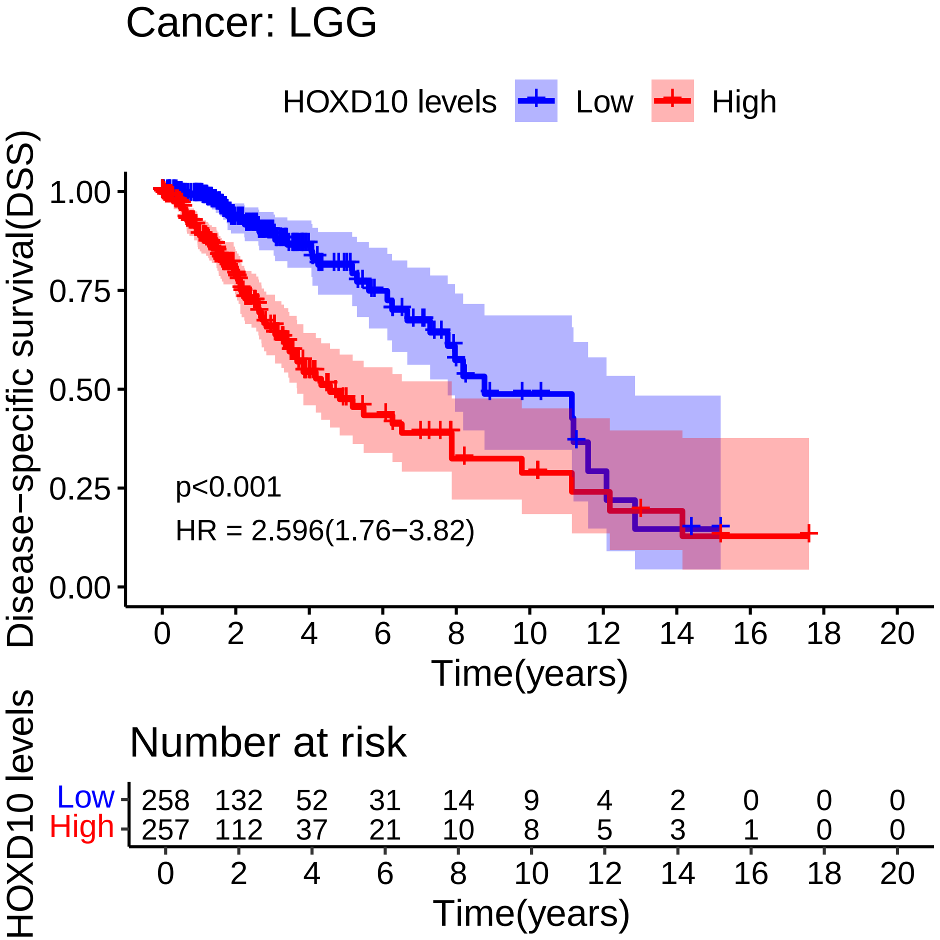

Cancer: LGG
HOXD10 levels
Low
High
Disease−specific survival(DSS)
1.00
0.75
0.50
0.25
0.00
p<0.001
HR = 2.596(1.76−3.82)
0
2
4
6
8
10
12
14
16
18
20
Time(years)
Number at risk
HOXD10 levels
Low 258 132 52 31 14 9 4 2 0 0 0
High 257 112 37 21 10 8 5 3 1 0 0
0
2
4
6
8
10
12
14
16
18
20
Time(years)

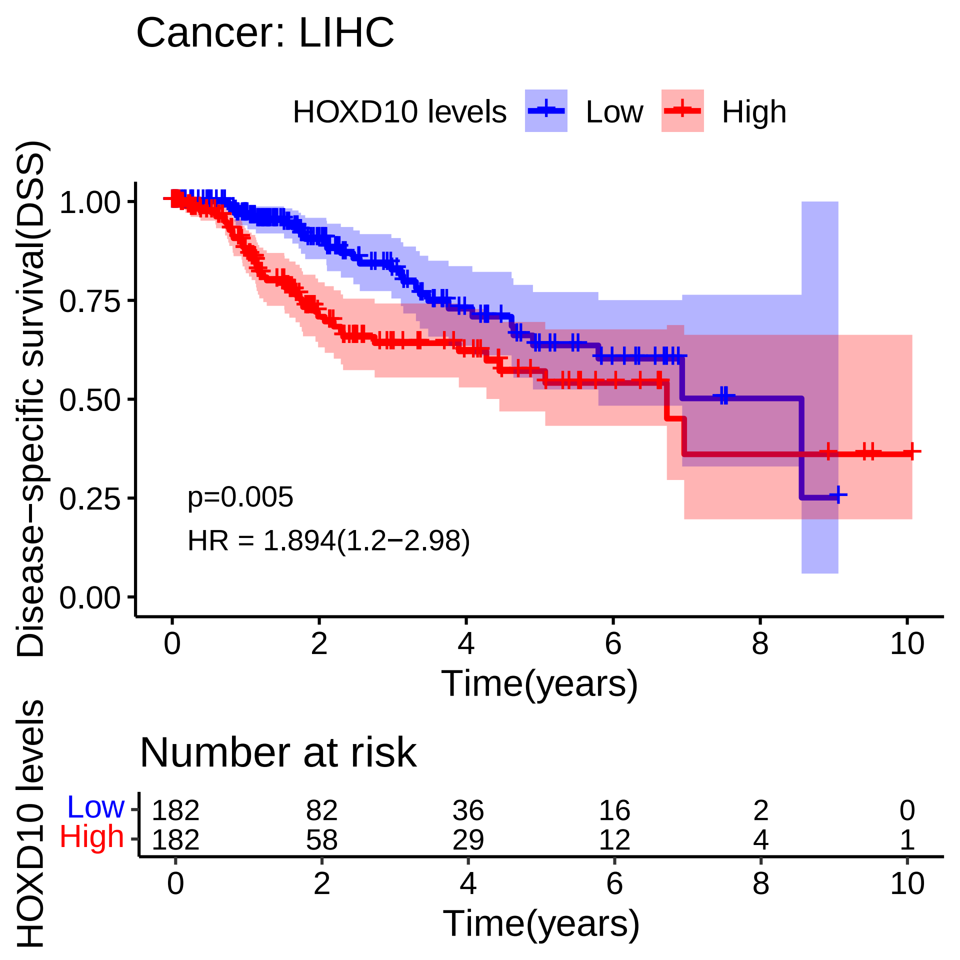

Cancer: LIHC
HOXD10 levels
Low
High
Disease−specific survival(DSS)
1.00
0.75
0.50
0.25
0.00
p=0.005
HR = 1.894(1.2−2.98)
0
2
4
6
8
10
Time(years)
Number at risk
HOXD10 levels
Low 182 82 36 16 2 0
High 182 58 29 12 4 1
Time(years)

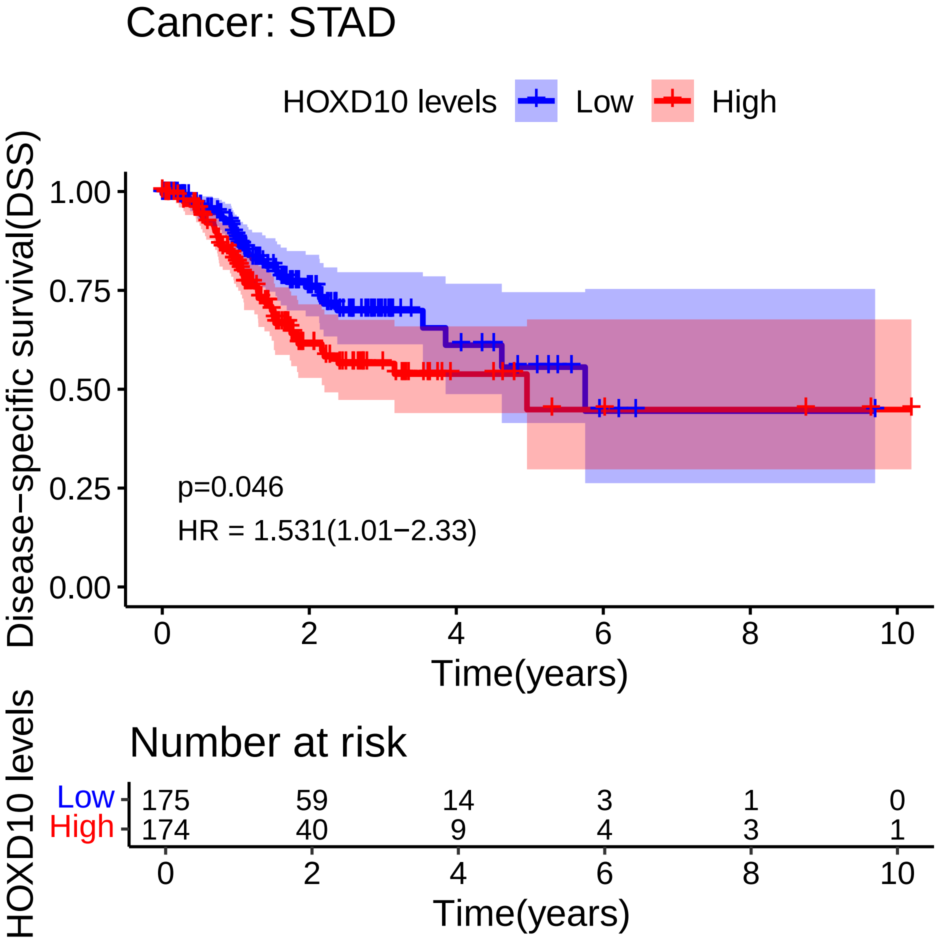

Cancer: STAD
HOXD10 levels
Low
High
Disease−specific survival(DSS)
1.00
0.75
0.50
0.25
0.00
p=0.046
HR = 1.531(1.01−2.33)
0
2
4
6
8
10
Time(years)
Number at risk
HOXD10 levels
Low 175 59 14 3 1 0
High 174 40 9 4 3 1
Time(years)

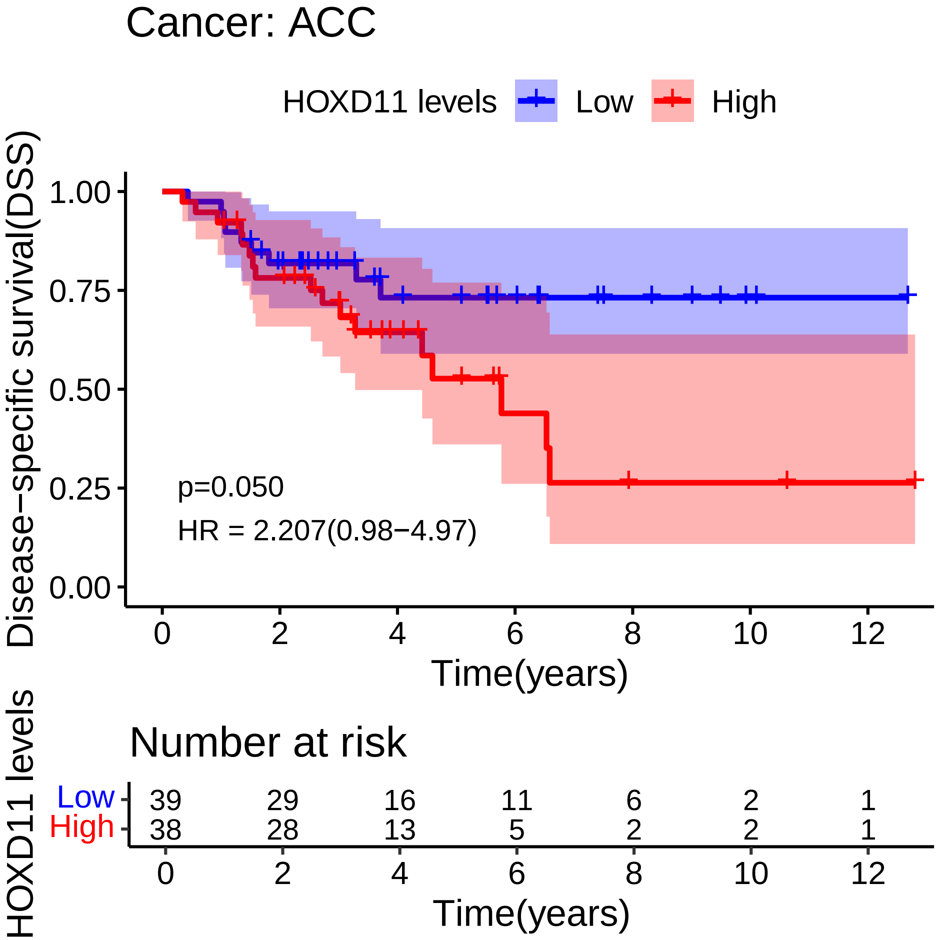

Cancer: ACC
HOXD11 levels
Low
High
Disease−specific survival(DSS)
1.00
0.75
0.50
0.25
0.00
p=0.050
HR = 2.207(0.98−4.97)
0
2
4
6
8
10
12
Time(years)
Number at risk
HOXD11 levels
Low 39 29 16 11 6 2 1
High 38 28 13 5 2 2 1
Time(years)

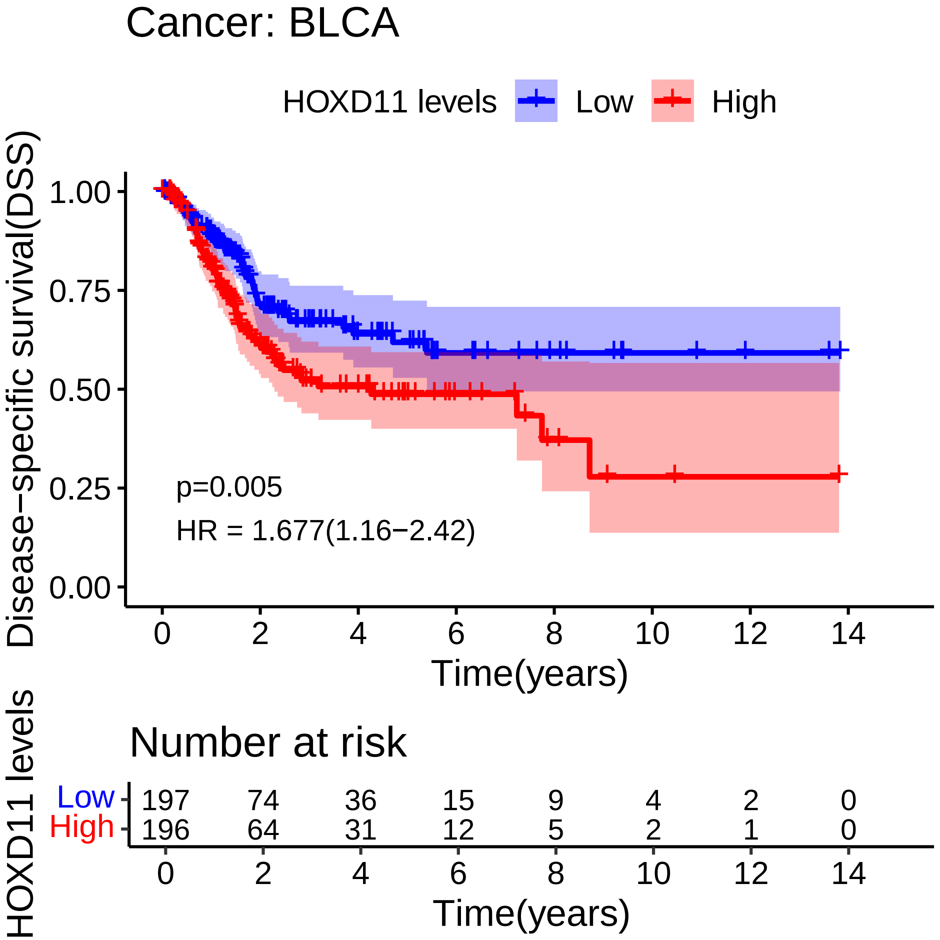

Cancer: BLCA
HOXD11 levels
Low
High
Disease−specific survival(DSS)
1.00
0.75
0.50
0.25
0.00
p=0.005
HR = 1.677(1.16−2.42)
0
2
4
6
8
10
12
14
Time(years)
Number at risk
HOXD11 levels
Low 197 74 36 15 9 4 2 0
High 196 64 31 12 5 2 1 0
Time(years)

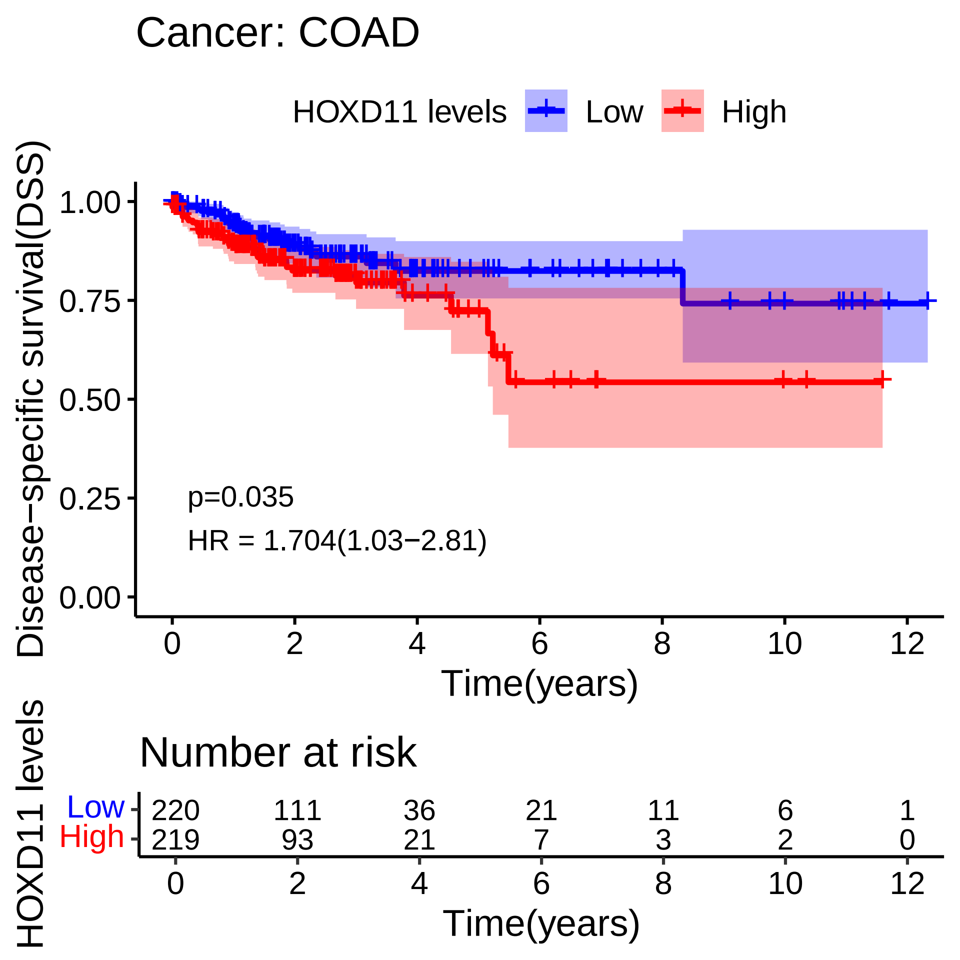
Cancer: COAD
HOXD11 levels
Low
High
Disease−specific survival(DSS)
1.00
0.75
0.50
0.25
0.00
p=0.035
HR = 1.704(1.03−2.81)
0
2
4
6
8
10
12
Time(years)
Number at risk
HOXD11 levels
Low
High
220 111 36 21 11 6 1
219 93 21 7 3 2 0
0
2
4
6
8
10
12
Time(years)

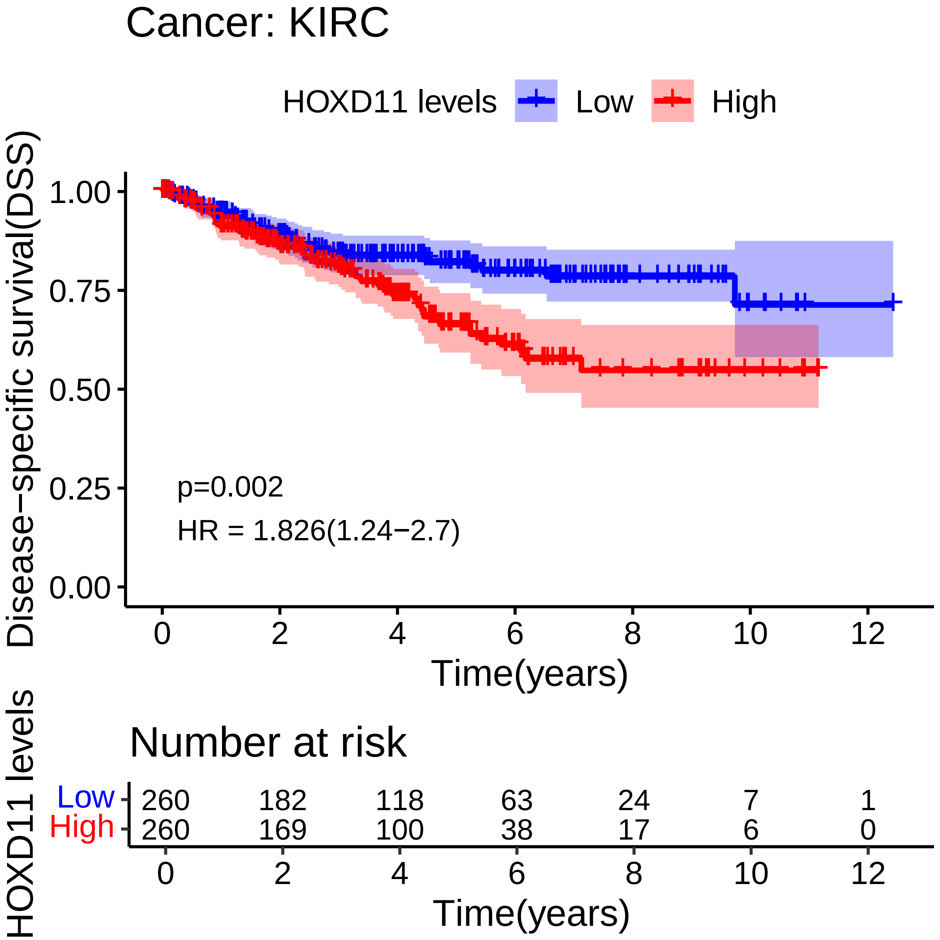

Cancer: KIRC
HOXD11 levels
Low
High
Disease−specific survival(DSS)
1.00
0.75
0.50
0.25
0.00
p=0.002
HR = 1.826(1.24−2.7)
0
2
4
6
8
10
12
Time(years)
Number at risk
HOXD11 levels
Low 260 182 118 63 24 7 1
High 260 169 100 38 17 6 0
Time(years)

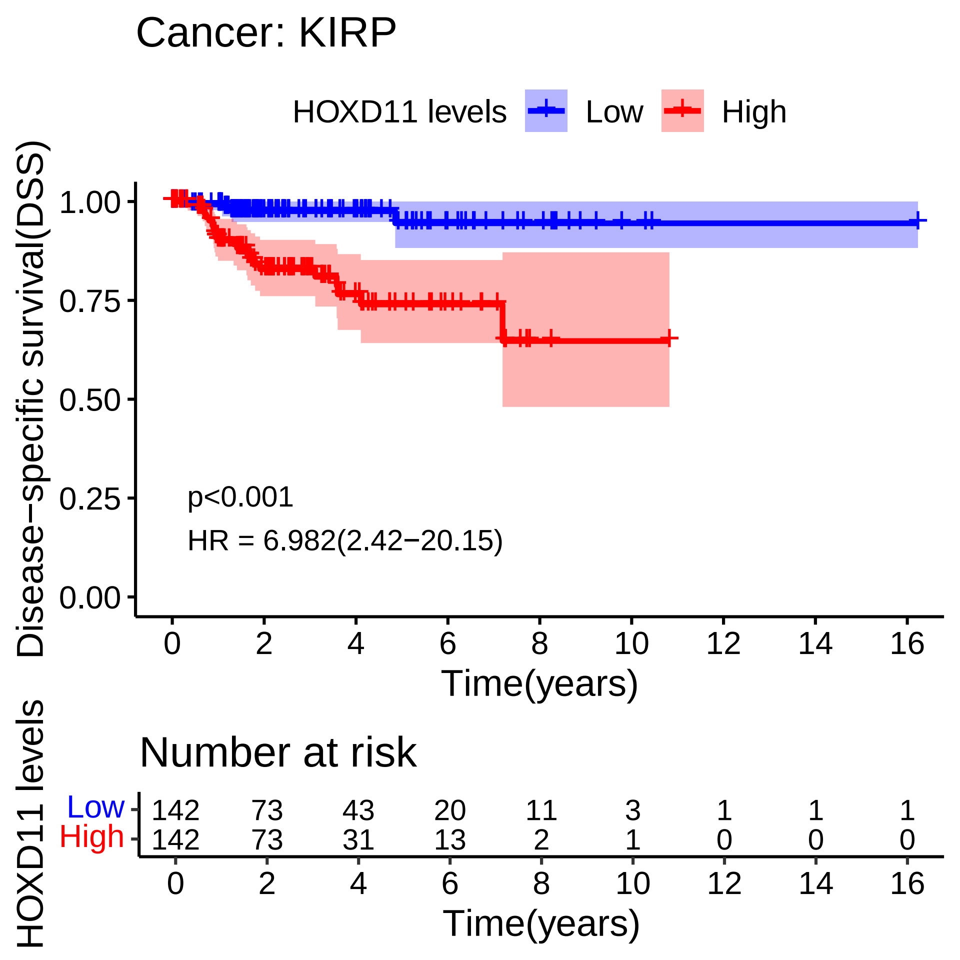
Cancer: KIRP
HOXD11 levels
Low
High
Disease−specific survival(DSS)
1.00
0.75
0.50
0.25
0.00
p<0.001
HR = 6.982(2.42−20.15)
0
2
4
6
8
10
12
14
16
Time(years)
Number at risk
HOXD11 levels
Low 142 73 43 20 11 3 1 1 1
High 142 73 31 13 2 1 0 0 0
Time(years)

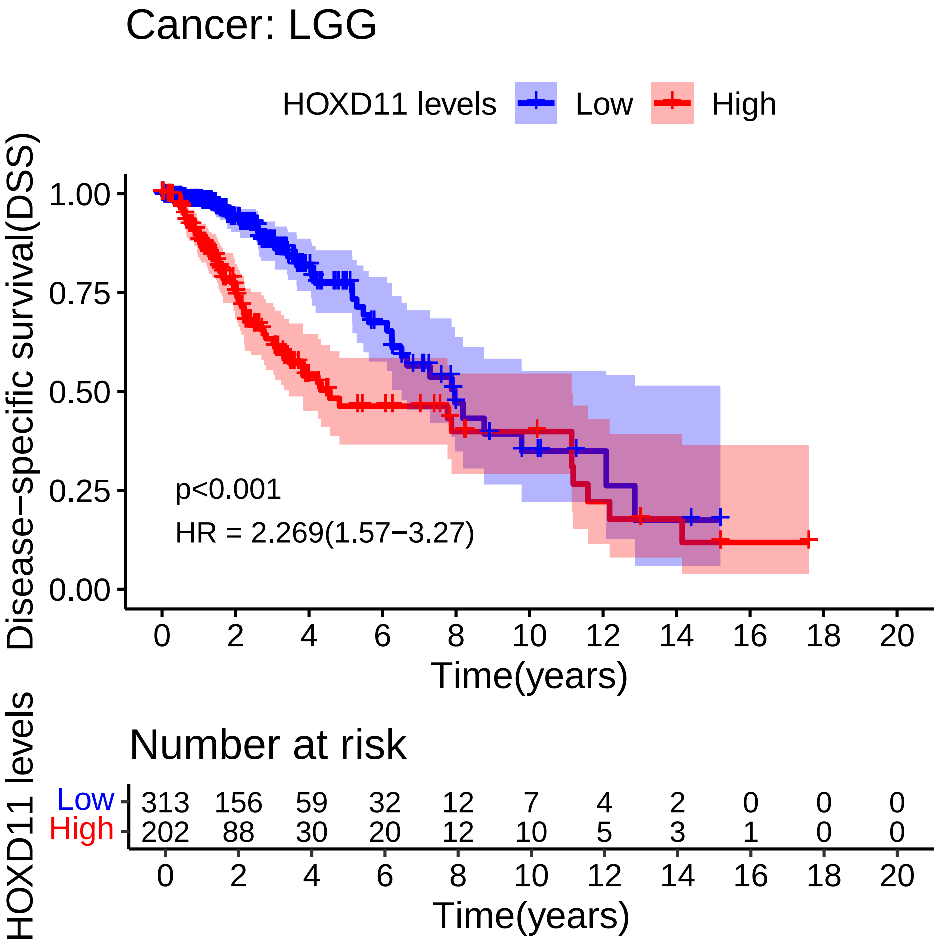
Cancer: LGG
HOXD11 levels
Low
High
Disease−specific survival(DSS)
1.00
0.75
0.50
0.25
0.00
p<0.001
HR = 2.269(1.57−3.27)
0 2 4 6 8 10 12 14 16 18 20
Time(years)
Number at risk
HOXD11 levels
Low 313 156 59 32 12 7 4 2 0 0 0
High 202 88 30 20 12 10 5 3 1 0 0
0 2 4 6 8 10 12 14 16 18 20
Time(years)

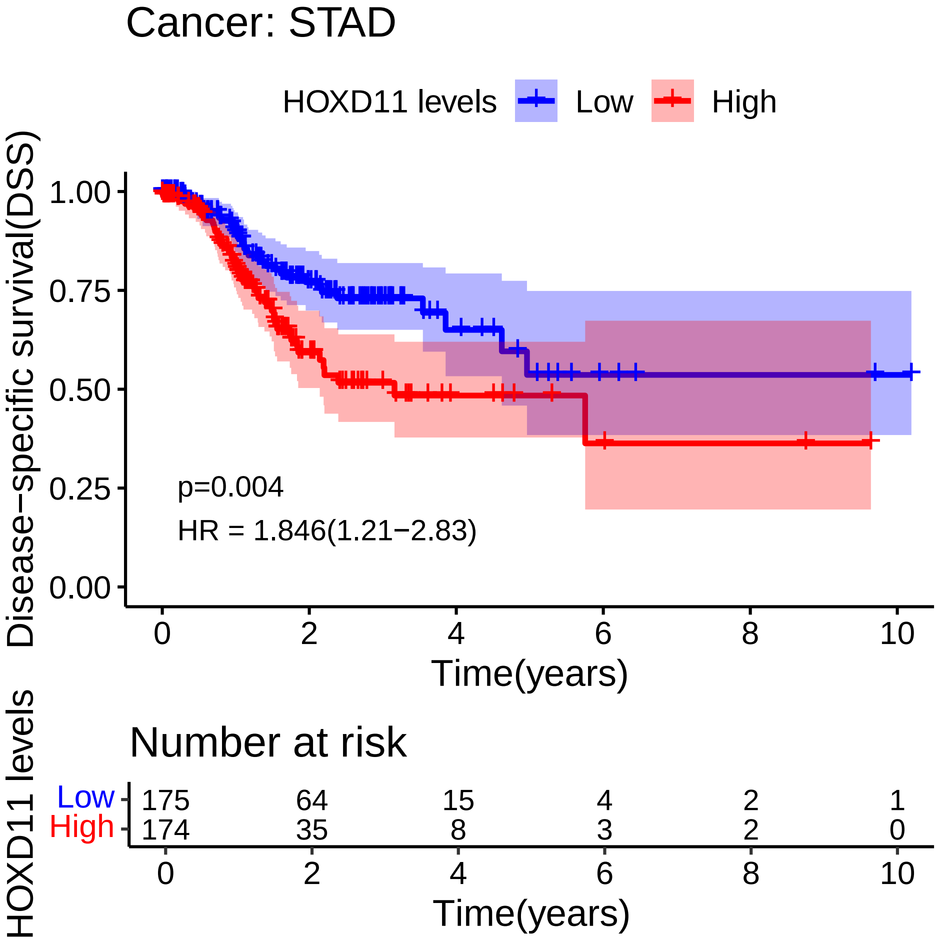

Cancer: STAD
HOXD11 levels
Low
High
Disease−specific survival(DSS)
1.00
0.75
0.50
0.25
0.00
p=0.004
HR = 1.846(1.21−2.83)
0
2
4
6
8
10
Time(years)
Number at risk
HOXD11 levels
Low
High
175 64 15 4 2 1
174 35 8 3 2 0
Time(years)

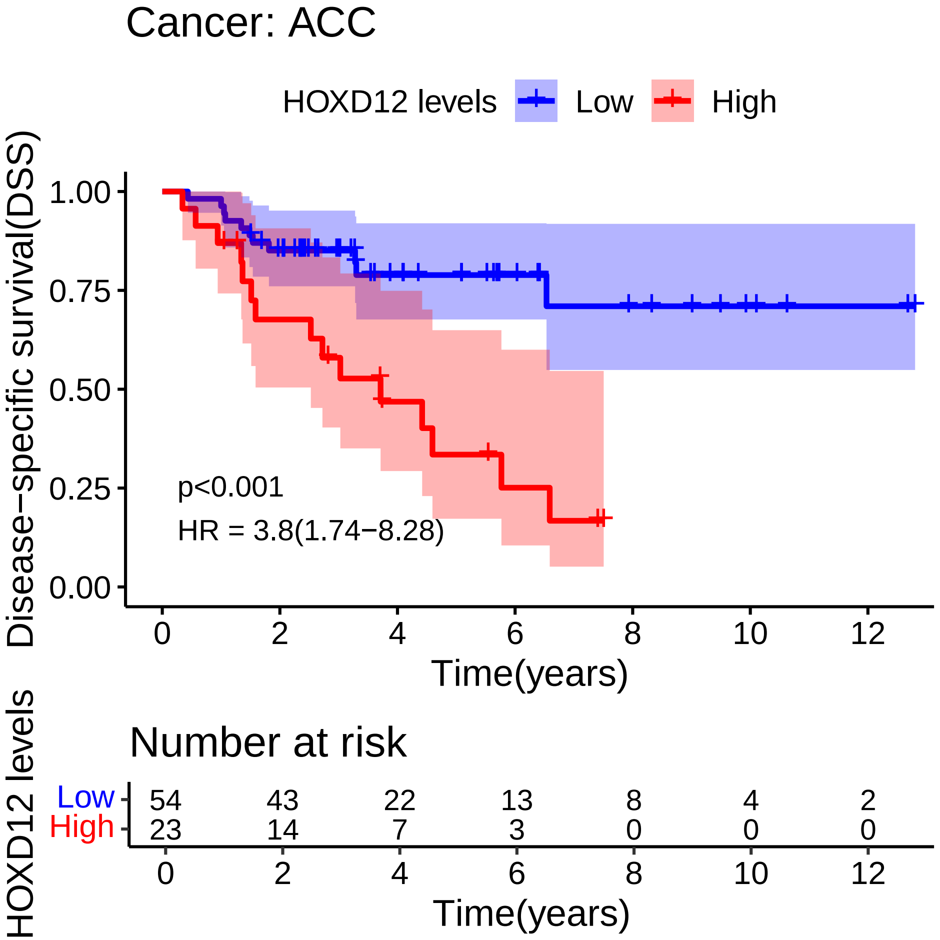
Cancer: ACC
HOXD12 levels
Low
High
Disease−specific survival(DSS)
1.00
0.75
0.50
0.25
0.00
p<0.001
HR = 3.8(1.74−8.28)
0
2
4
6
8
10
12
Time(years)
Number at risk
HOXD12 levels
Low 54 43 22 13 8 4 2
High 23 14 7 3 0 0 0
0
2
4
6
8
10
12
Time(years)

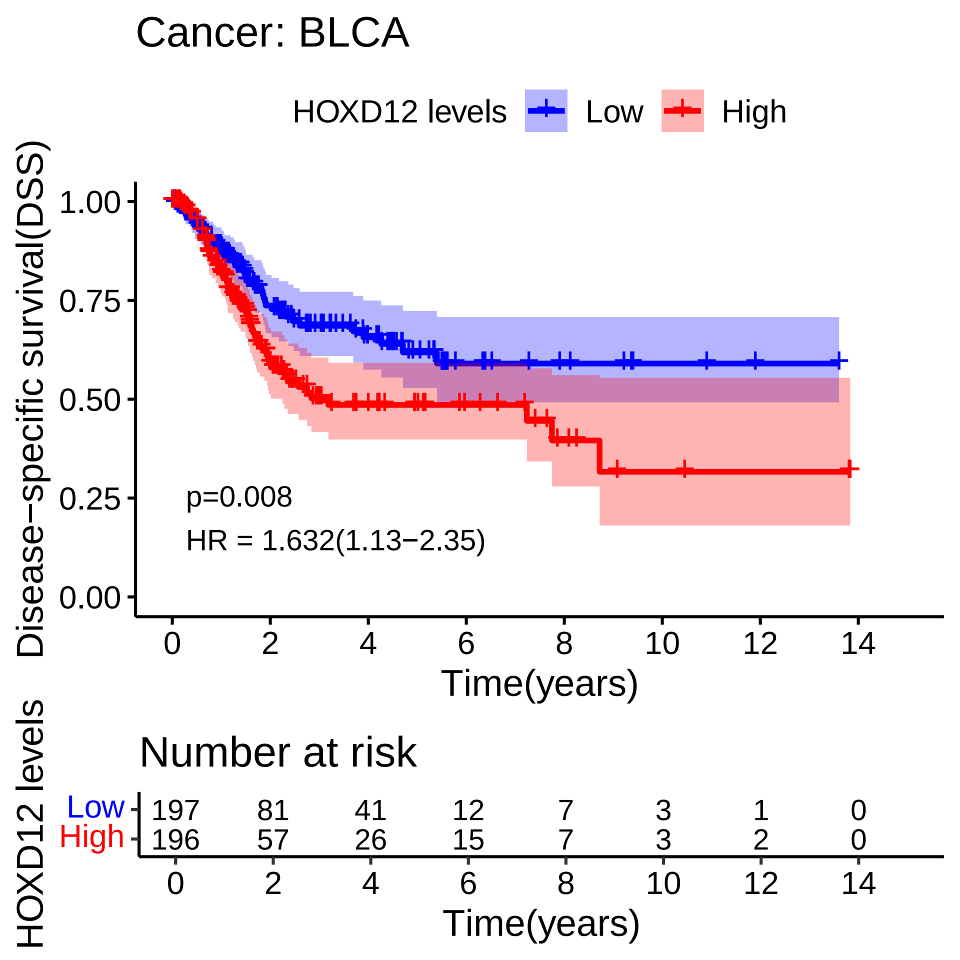

Cancer: BLCA
HOXD12 levels
Low
High
Disease−specific survival(DSS)
1.00
0.75
0.50
0.25
0.00
p=0.008
HR = 1.632(1.13−2.35)
0
2
4
6
8
10
12
14
Time(years)
Number at risk
HOXD12 levels
Low 197 81 41 12 7 3 1 0
High 196 57 26 15 7 3 2 0
Time(years)

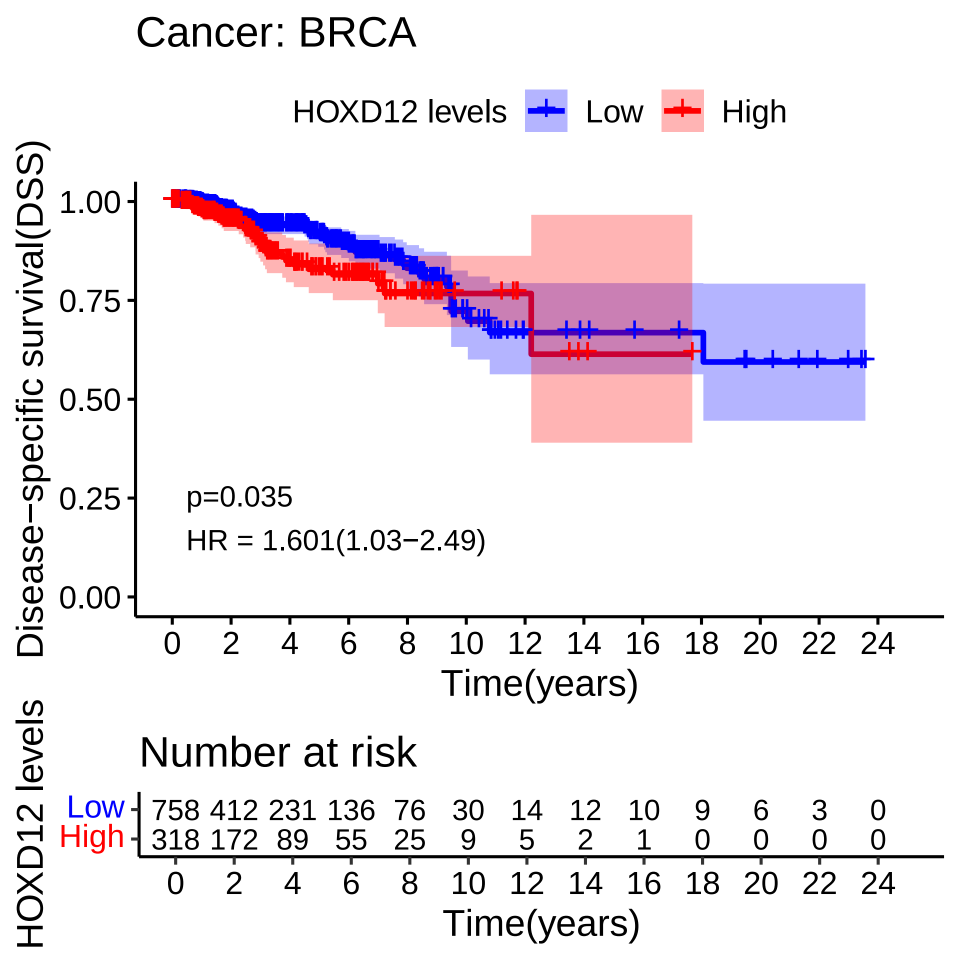
Cancer: BRCA
HOXD12 levels
Low
High
Disease−specific survival(DSS)
1.00
0.75
0.50
0.25
0.00
p=0.035
HR = 1.601(1.03−2.49)
0
2
4
6
8
10
12
14
16
18
20
22
24
Time(years)
Number at risk
HOXD12 levels
Low 758 412 231 136 76 30 14 12 10 9 6 3 0
High 318 172 89 55 25 9 5 2 1 0 0 0 0
Time(years)

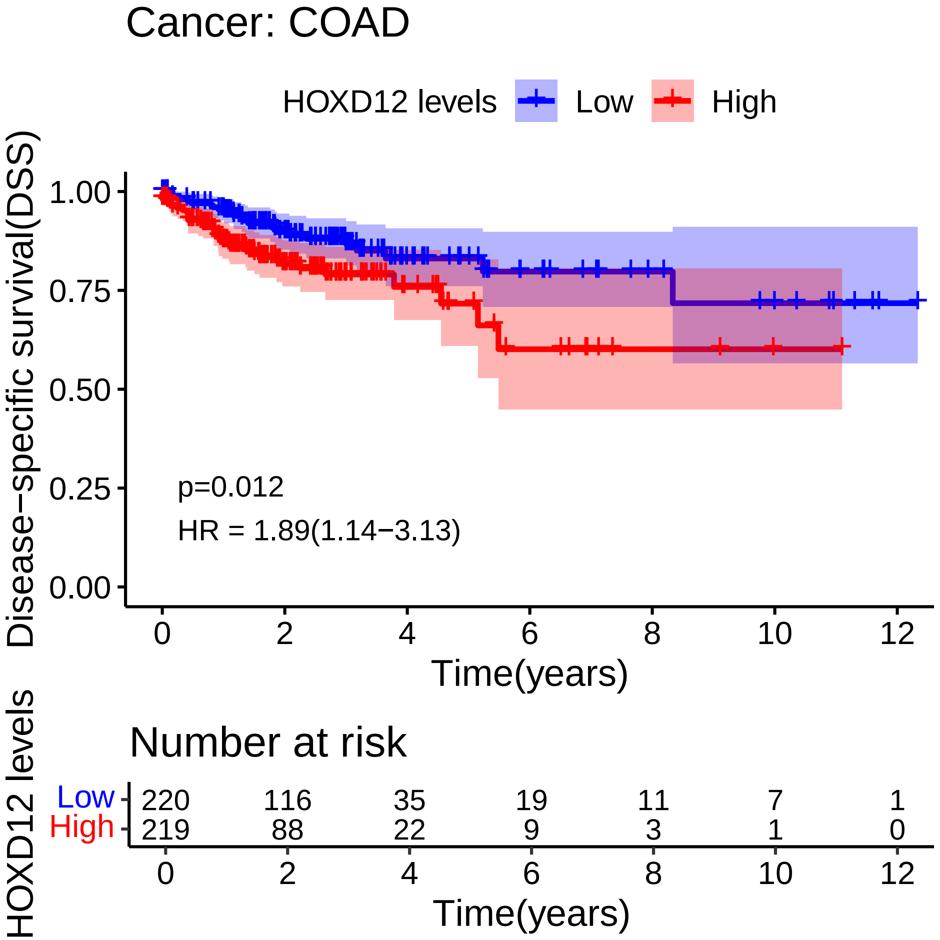
Cancer: COAD
HOXD12 levels
Low
High
Disease−specific survival(DSS)
1.00
0.75
0.50
0.25
0.00
p=0.012
HR = 1.89(1.14−3.13)
0
2
4
6
8
10
12
Time(years)
Number at risk
HOXD12 levels
Low
High
220 116 35 19 11 7 1
219 88 22 9 3 1 0
Time(years)

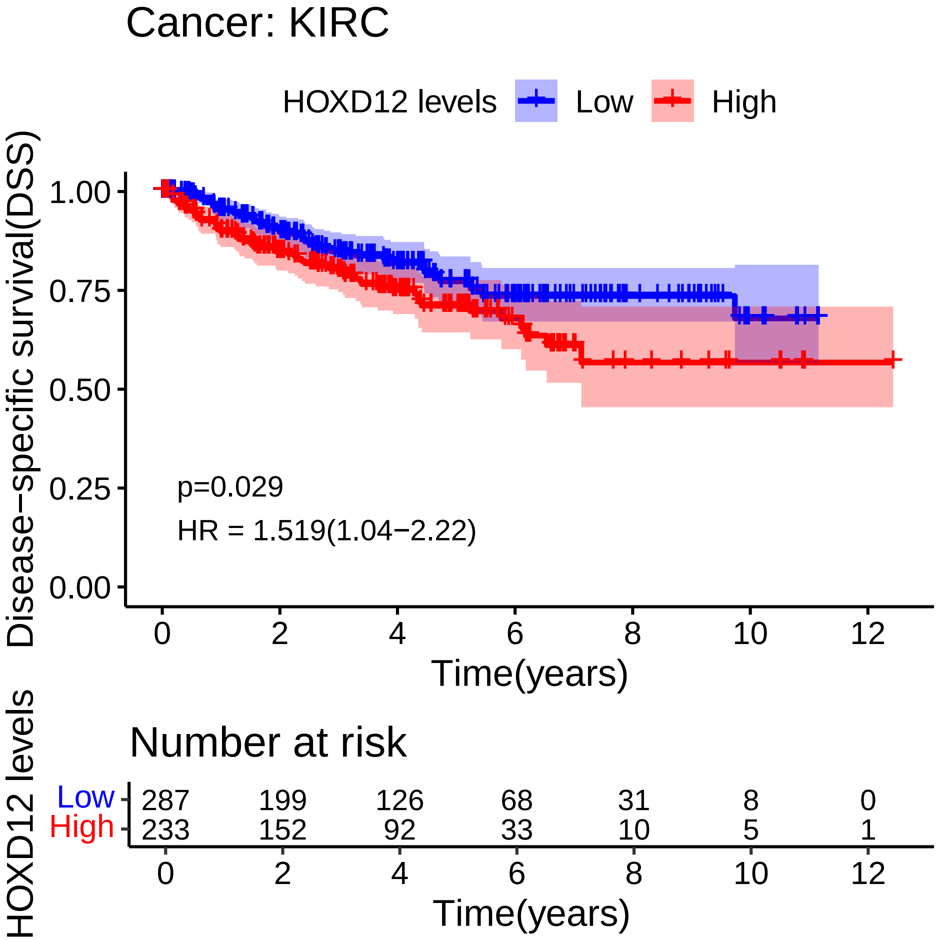

Cancer: KIRC
HOXD12 levels
Low
High
Disease−specific survival(DSS)
1.00
0.75
0.50
0.25
0.00
p=0.029
HR = 1.519(1.04−2.22)
0
2
4
6
8
10
12
Time(years)
Number at risk
HOXD12 levels
Low 287 199 126 68 31 8 0
High 233 152 92 33 10 5 1
Time(years)

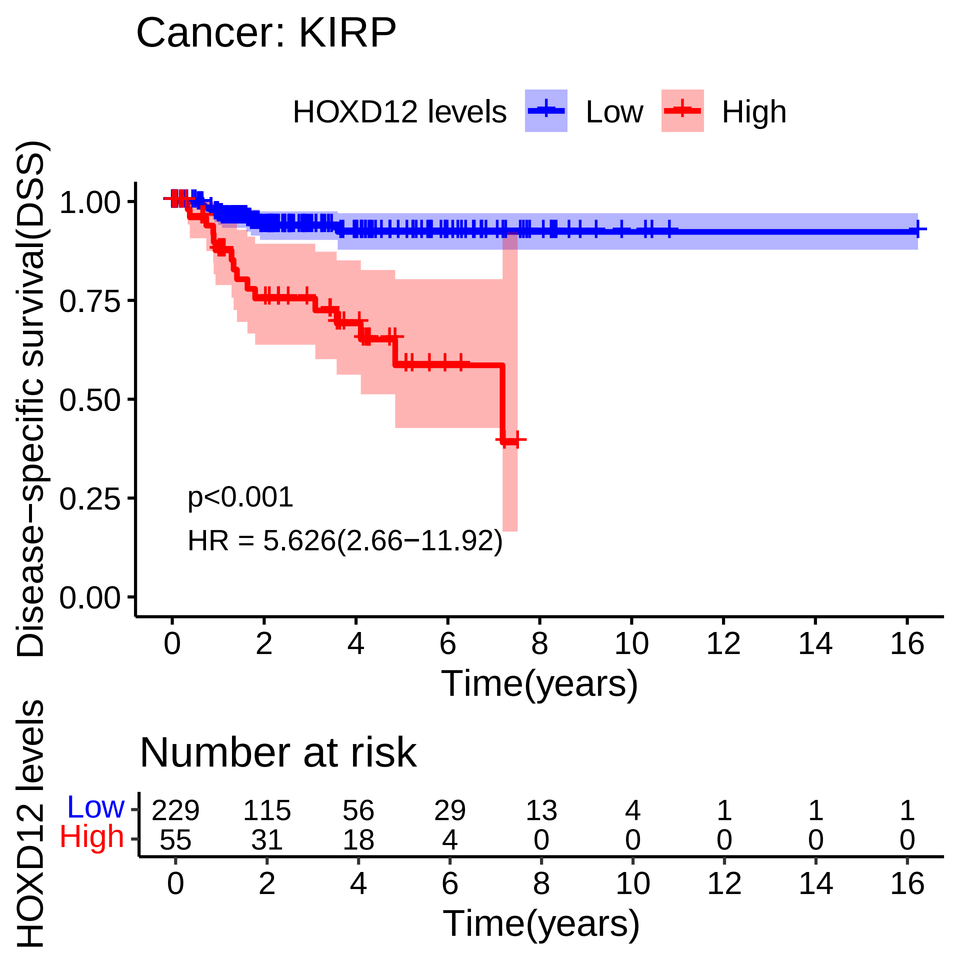

Cancer: KIRP
HOXD12 levels
Low
High
Disease−specific survival(DSS)
1.00
0.75
0.50
0.25
0.00
p<0.001
HR = 5.626(2.66−11.92)
0 2 4 6 8 10 12 14 16
Time(years)
Number at risk
HOXD12 levels
Low 229 115 56 29 13 4 1 1 1
High 55 31 18 4 0 0 0 0 0
0 2 4 6 8 10 12 14 16
Time(years)

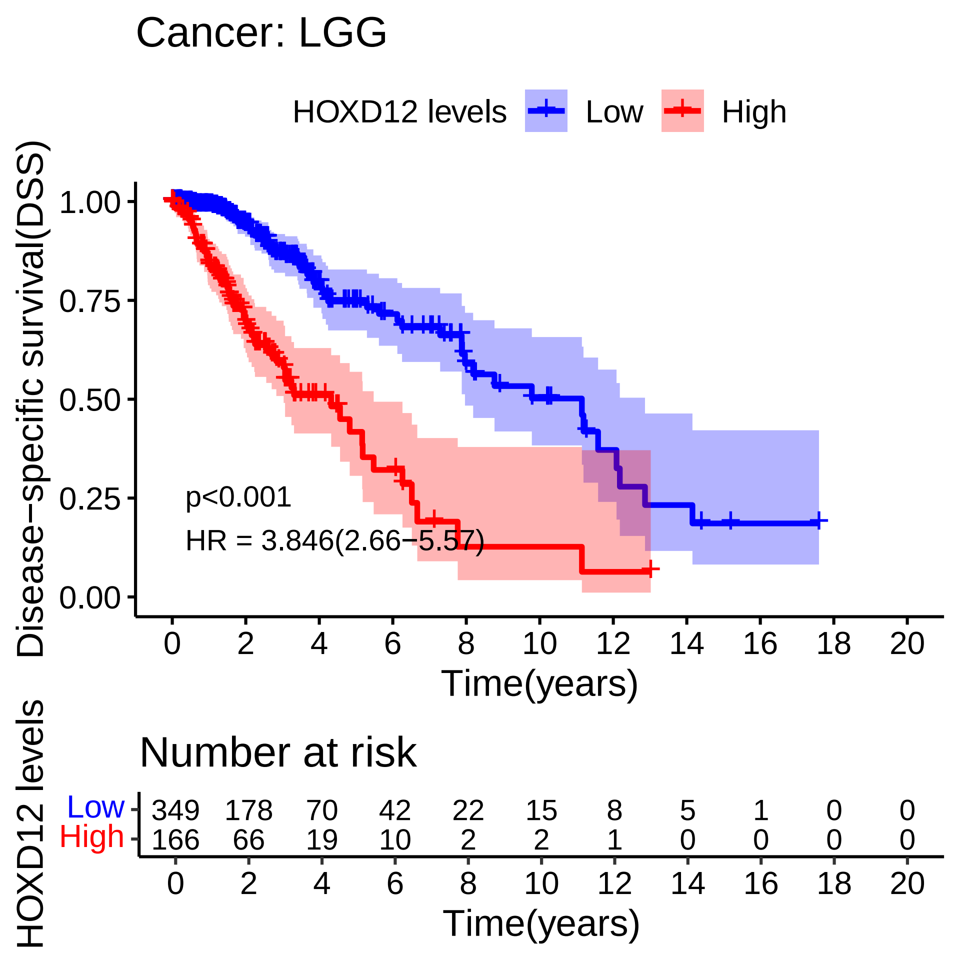
Cancer: LGG
HOXD12 levels
Low
High
Disease−specific survival(DSS)
1.00
0.75
0.50
0.25
0.00
p<0.001
HR = 3.846(2.66−5.57)
0 2 4 6 8 10 12 14 16 18 20
Time(years)
Number at risk
HOXD12 levels
Low 349 178 70 42 22 15 8 5 1 0 0
High 166 66 19 10 2 2 1 0 0 0 0
0 2 4 6 8 10 12 14 16 18 20
Time(years)

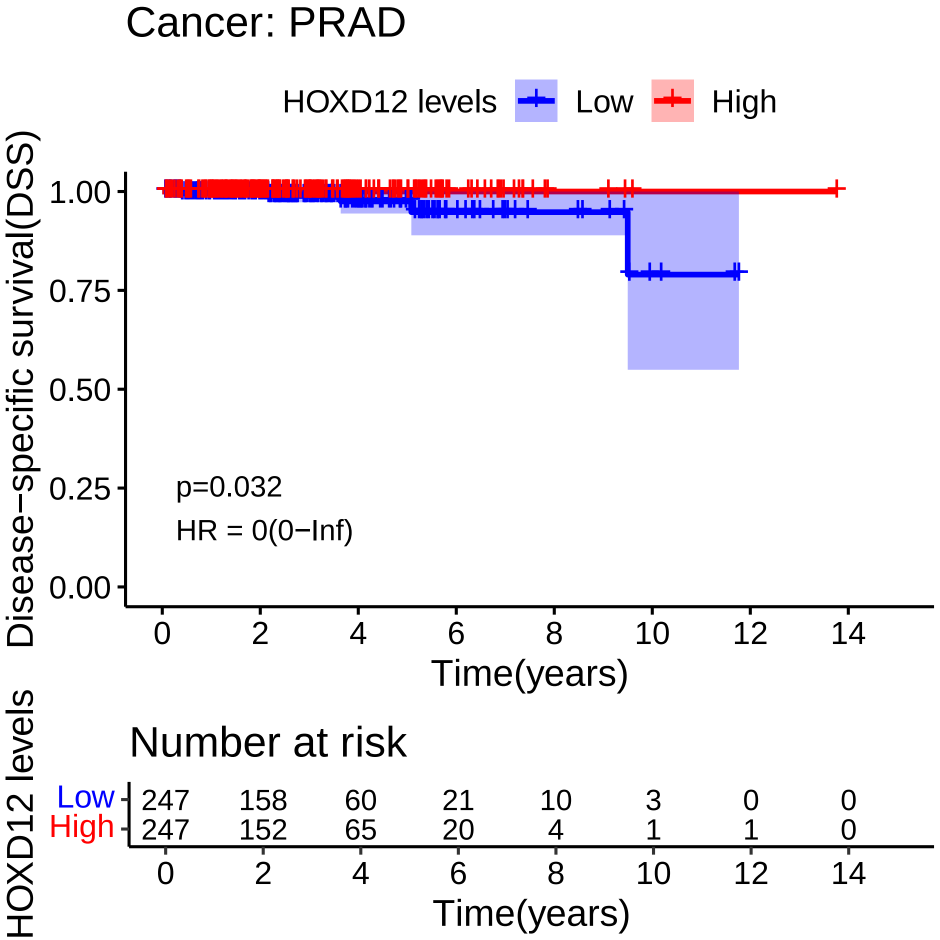
Cancer: PRAD
HOXD12 levels
Low
High
Disease−specific survival(DSS)
1.00
0.75
0.50
0.25
0.00
p=0.032
HR = 0(0−Inf)
0
2
4
6
8
10
12
14
Time(years)
Number at risk
HOXD12 levels
Low 247 158 60 21 10 3 0 0
High 247 152 65 20 4 1 1 0
Time(years)

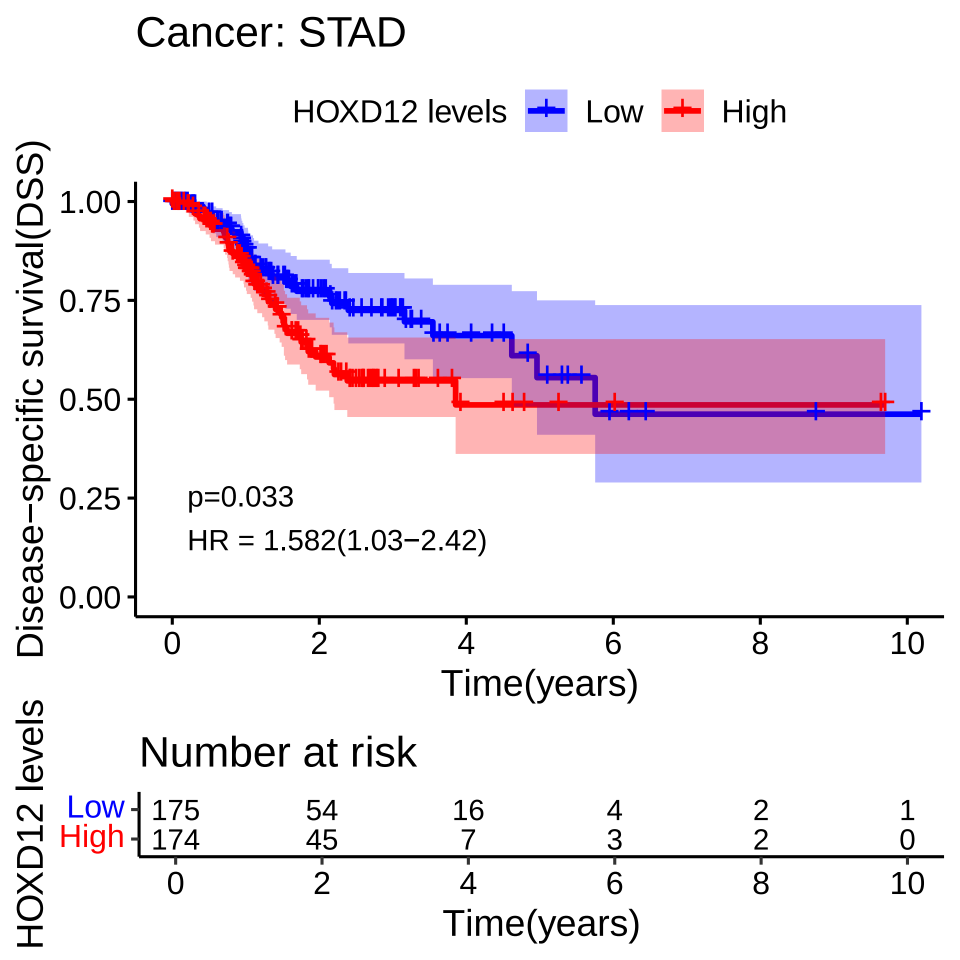

Cancer: STAD
HOXD12 levels
Low
High
Disease−specific survival(DSS)
1.00
0.75
0.50
0.25
0.00
p=0.033
HR = 1.582(1.03−2.42)
0
2
4
6
8
10
Time(years)
Number at risk
HOXD12 levels
Low 175 54 16 4 2 1
High 174 45 7 3 2 0
0
2
4
6
8
10
Time(years)

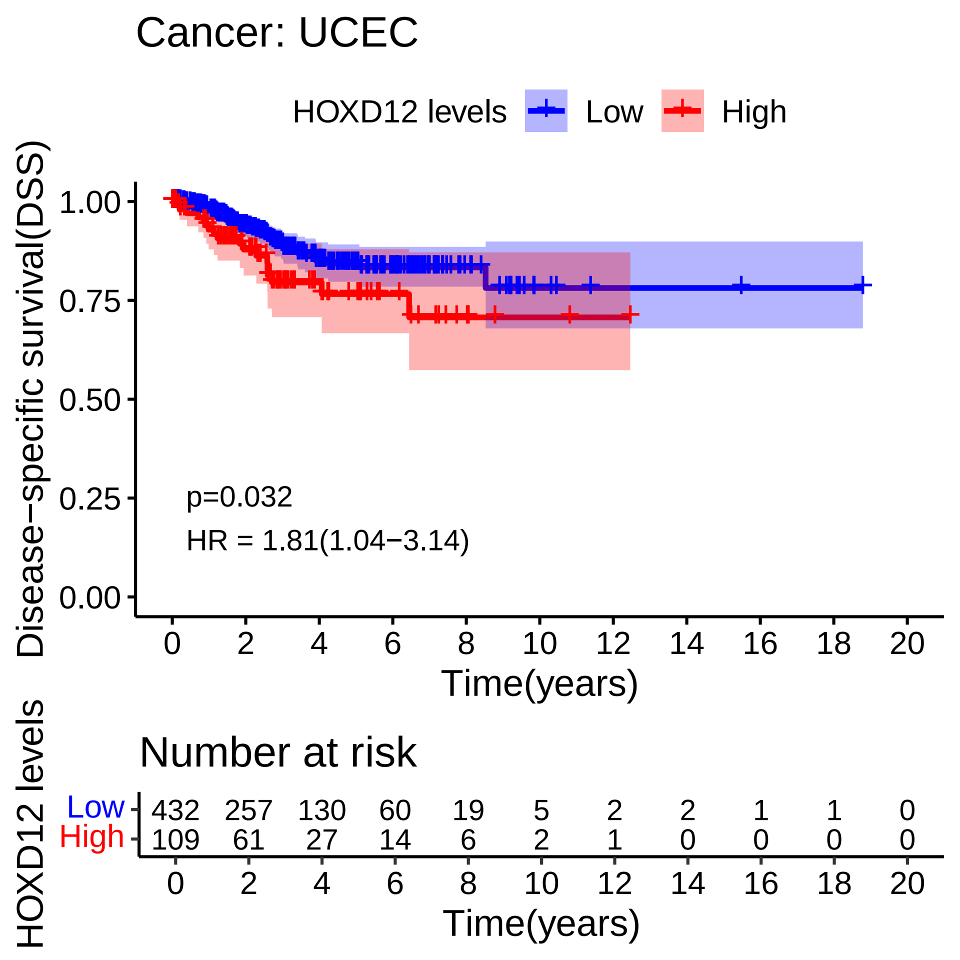
Cancer: UCEC
HOXD12 levels
Low
High
Disease−specific survival(DSS)
1.00
0.75
0.50
0.25
0.00
p=0.032
HR = 1.81(1.04−3.14)
0 2 4 6 8 10 12 14 16 18 20
Time(years)
Number at risk
HOXD12 levels
Low 432 257 130 60 19 5 2 2 1 1 0
High 109 61 27 14 6 2 1 0 0 0 0
0 2 4 6 8 10 12 14 16 18 20
Time(years)

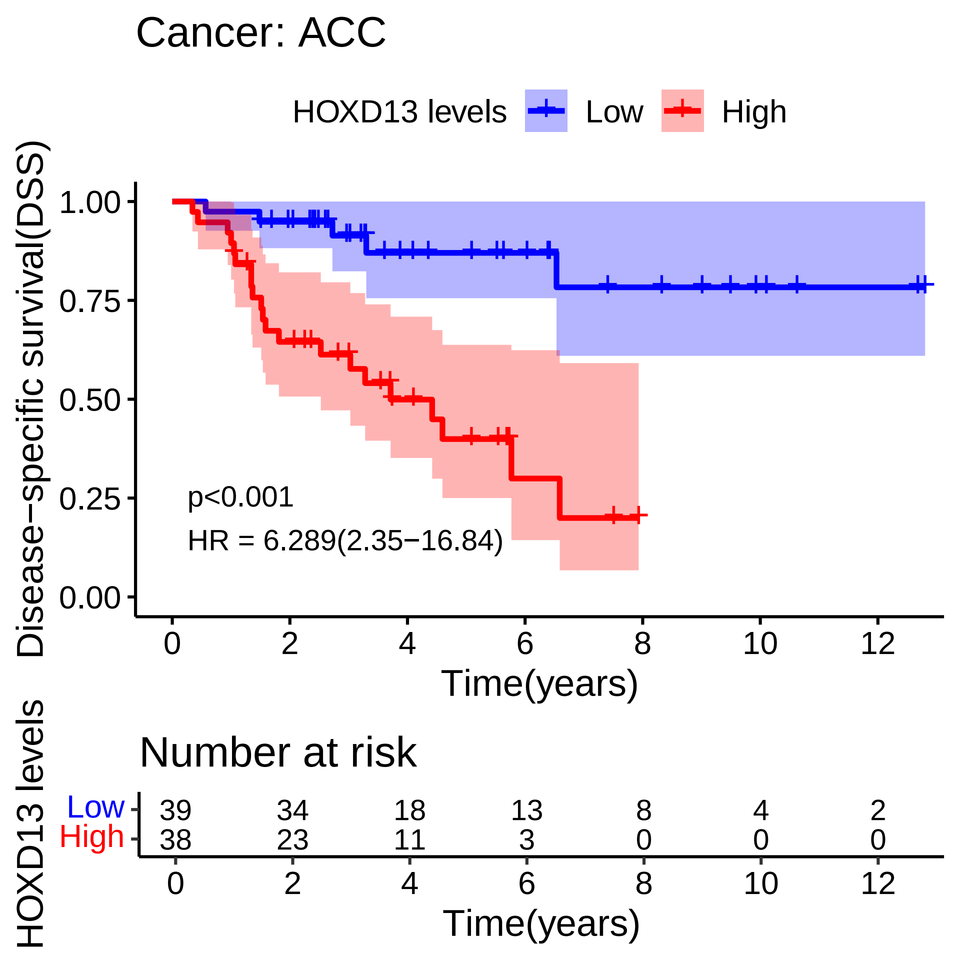
Cancer: ACC
HOXD13 levels
Low
High
Disease−specific survival(DSS)
1.00
0.75
0.50
0.25
0.00
p<0.001
HR = 6.289(2.35−16.84)
0
2
4
6
8
10
12
Time(years)
Number at risk
HOXD13 levels
Low 39 34 18 13 8 4 2
High 38 23 11 3 0 0 0
0
2
4
6
8
10
12
Time(years)

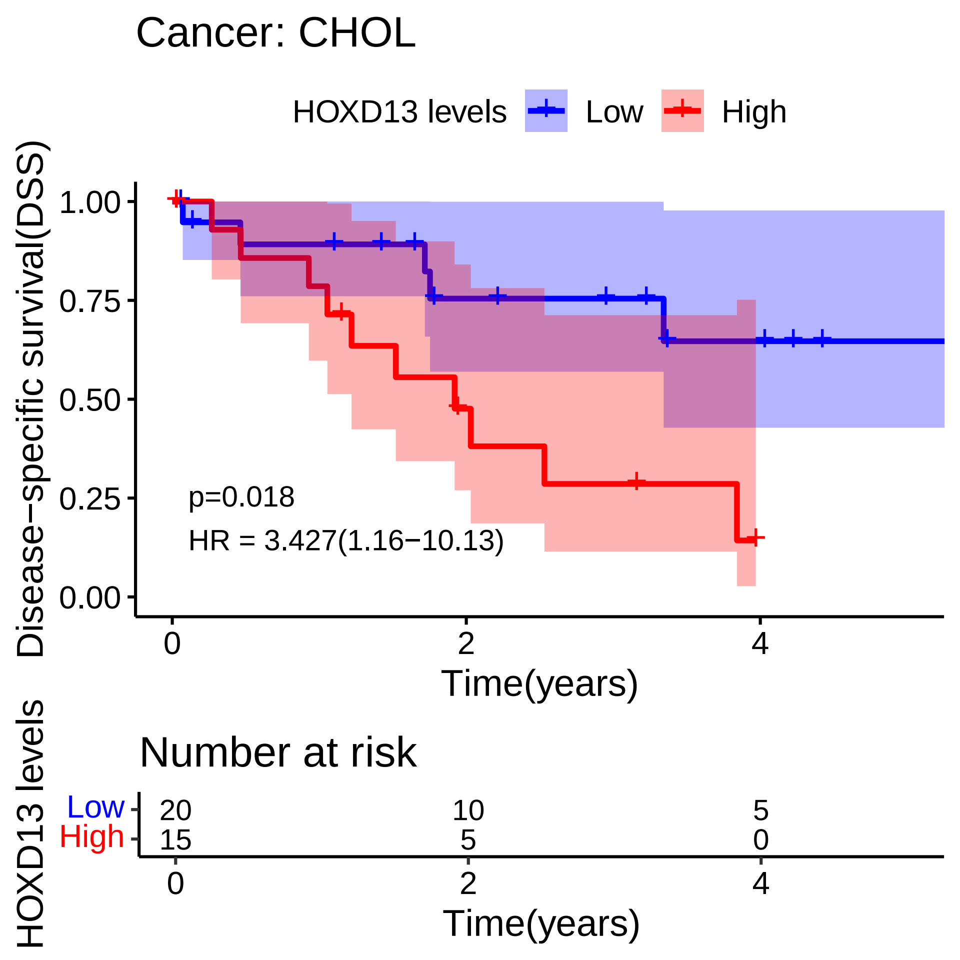

Cancer: CHOL
HOXD13 levels
Low
High
Disease−specific survival(DSS)
1.00
0.75
0.50
0.25
0.00
p=0.018
HR = 3.427(1.16−10.13)
0
2
4
Time(years)
Number at risk
HOXD13 levels
Low
High
20
10
5
15
5
0
0
2
4
Time(years)

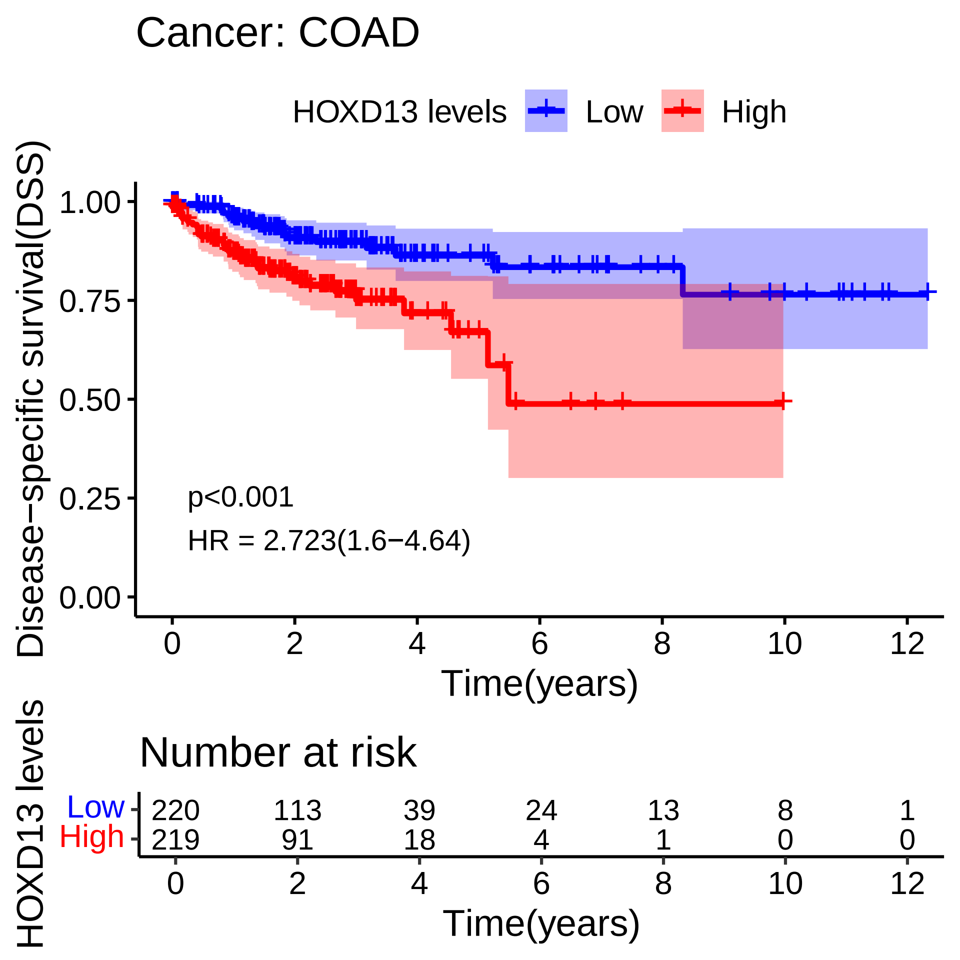

Cancer: COAD
HOXD13 levels
Low
High
Disease−specific survival(DSS)
1.00
0.75
0.50
0.25
0.00
p<0.001
HR = 2.723(1.6−4.64)
0
2
4
6
8
10
12
Time(years)
Number at risk
HOXD13 levels
Low 220 113 39 24 13 8 1
High 219 91 18 4 1 0 0
Time(years)

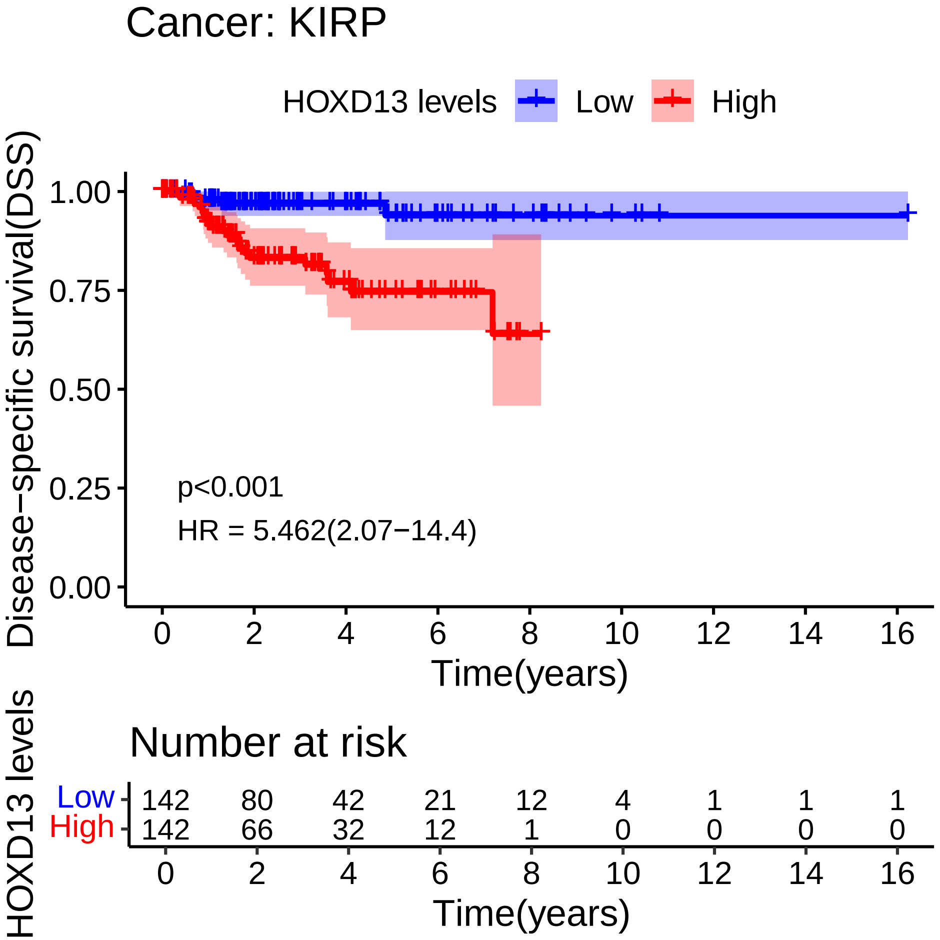
Cancer: KIRP
HOXD13 levels
Low
High
Disease−specific survival(DSS)
1.00
0.75
0.50
0.25
0.00
p<0.001
HR = 5.462(2.07−14.4)
0
2
4
6
8
10
12
14
16
Time(years)
Number at risk
HOXD13 levels
Low
High
142 80 42 21 12 4 1 1 1
142 66 32 12 1 0 0 0 0
0
2
4
6
8
10
12
14
16
Time(years)

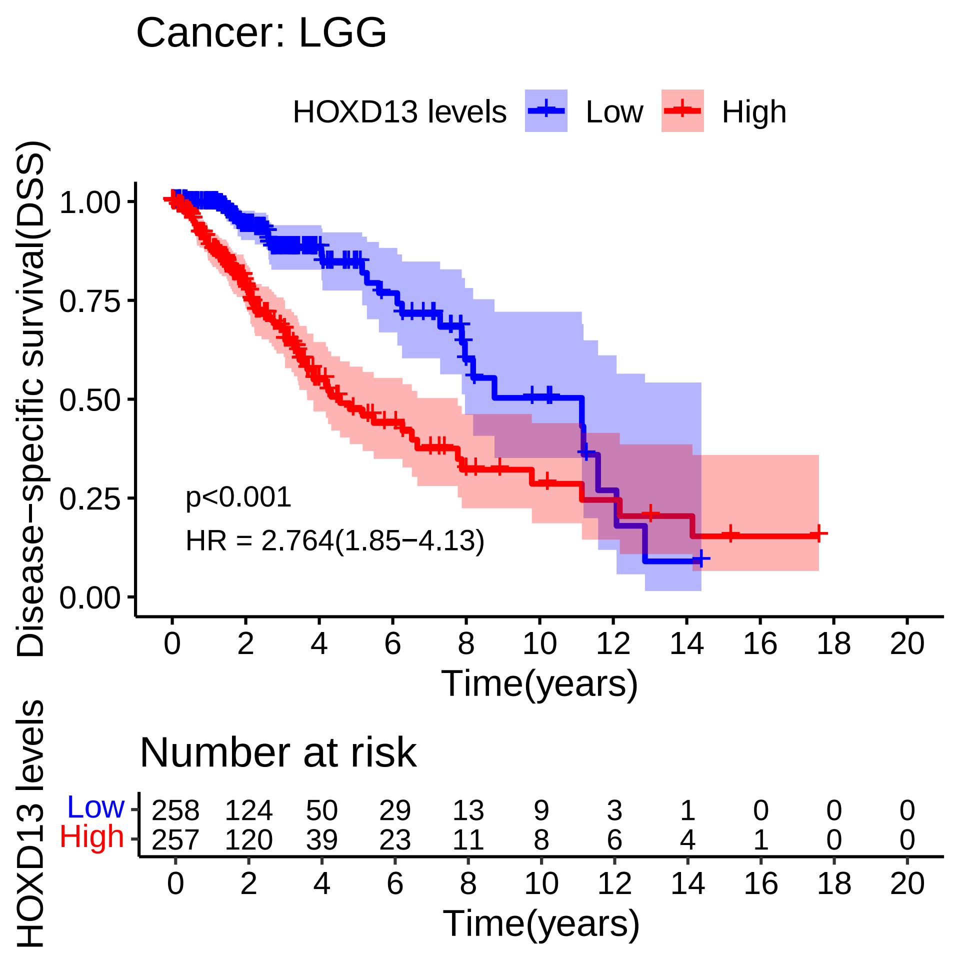

Cancer: LGG
HOXD13 levels
Low
High
Disease−specific survival(DSS)
1.00
0.75
0.50
0.25
0.00
p<0.001
HR = 2.764(1.85−4.13)
0 2 4 6 8 10 12 14 16 18 20
Time(years)
Number at risk
HOXD13 levels
Low 258 124 50 29 13 9 3 1 0 0 0
High 257 120 39 23 11 8 6 4 1 0 0
0 2 4 6 8 10 12 14 16 18 20
Time(years)

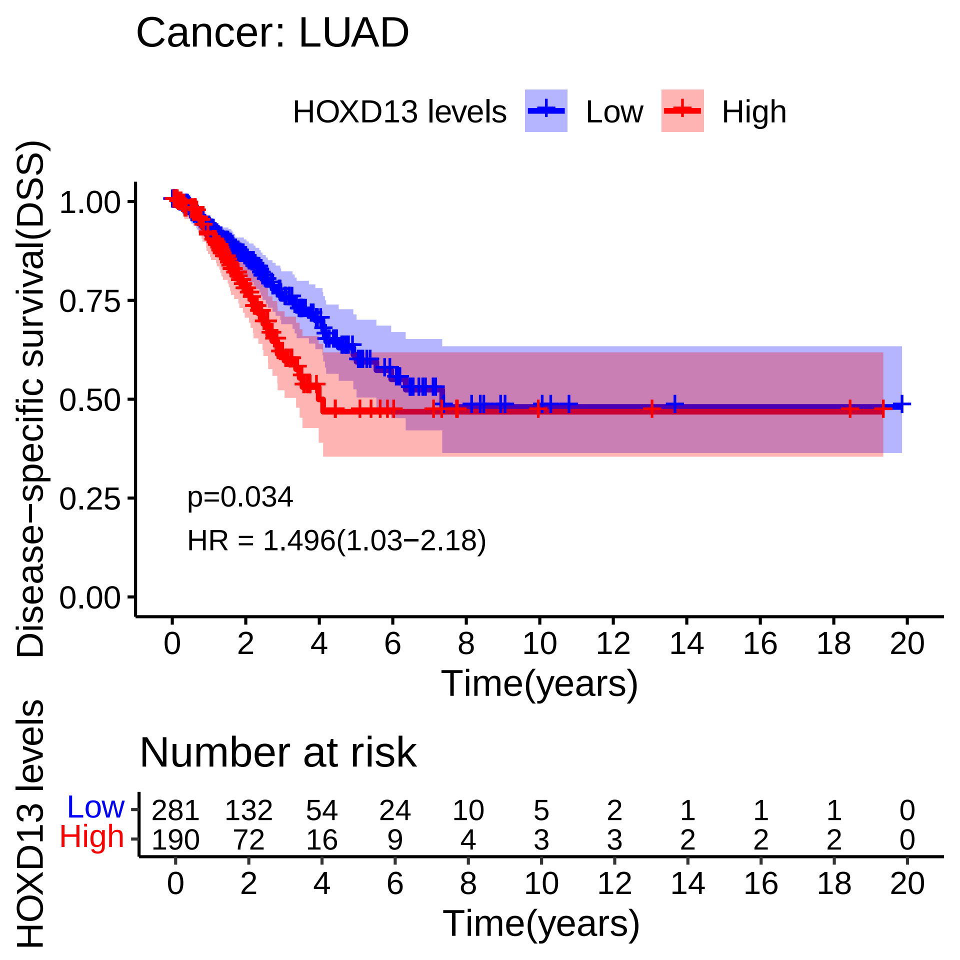

Cancer: LUAD
HOXD13 levels
Low
High
Disease−specific survival(DSS)
1.00
0.75
0.50
0.25
0.00
p=0.034
HR = 1.496(1.03−2.18)
0
2
4
6
8
10
12
14
16
18
20
Time(years)
Number at risk
HOXD13 levels
Low 281 132 54 24 10 5 2 1 1 1 0
High 190 72 16 9 4 3 3 2 2 2 0
Time(years)

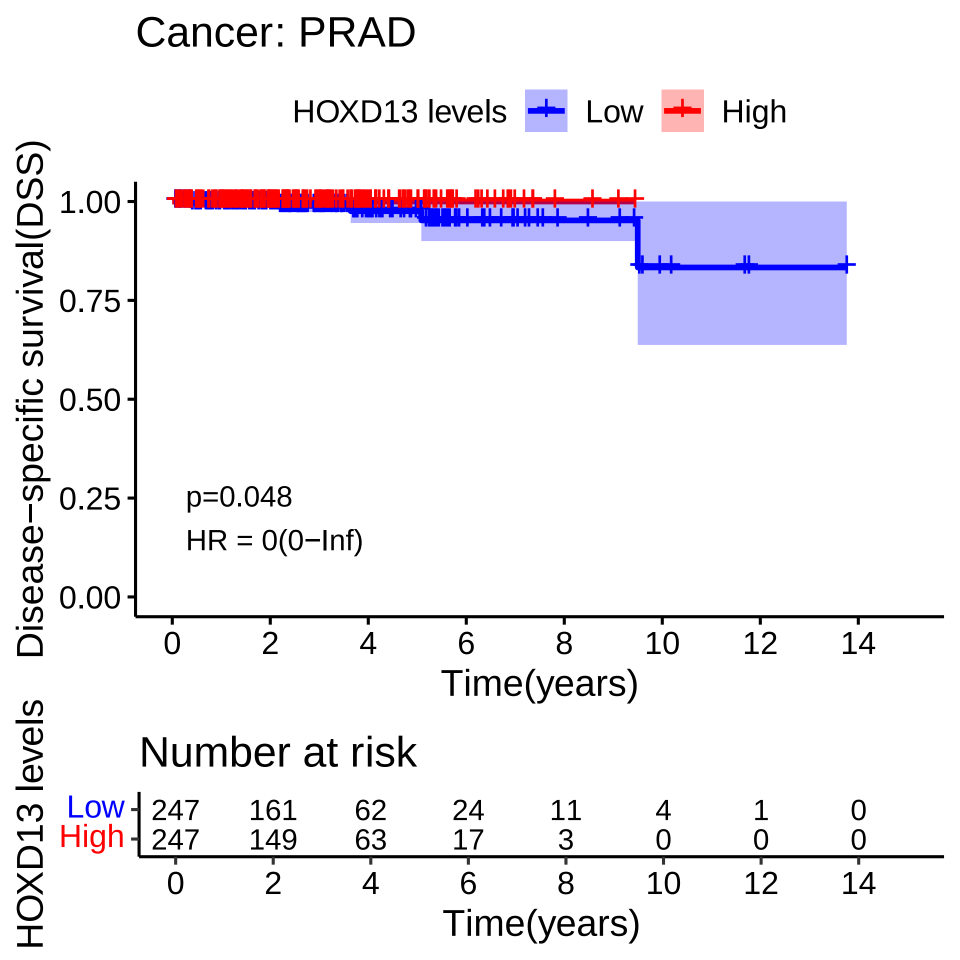
Cancer: PRAD
HOXD13 levels
Low
High
Disease−specific survival(DSS)
1.00
0.75
0.50
0.25
0.00
p=0.048
HR = 0(0−Inf)
0
2
4
6
8
10
12
14
Time(years)
Number at risk
HOXD13 levels
Low 247 161 62 24 11 4 1 0
High 247 149 63 17 3 0 0 0
Time(years)

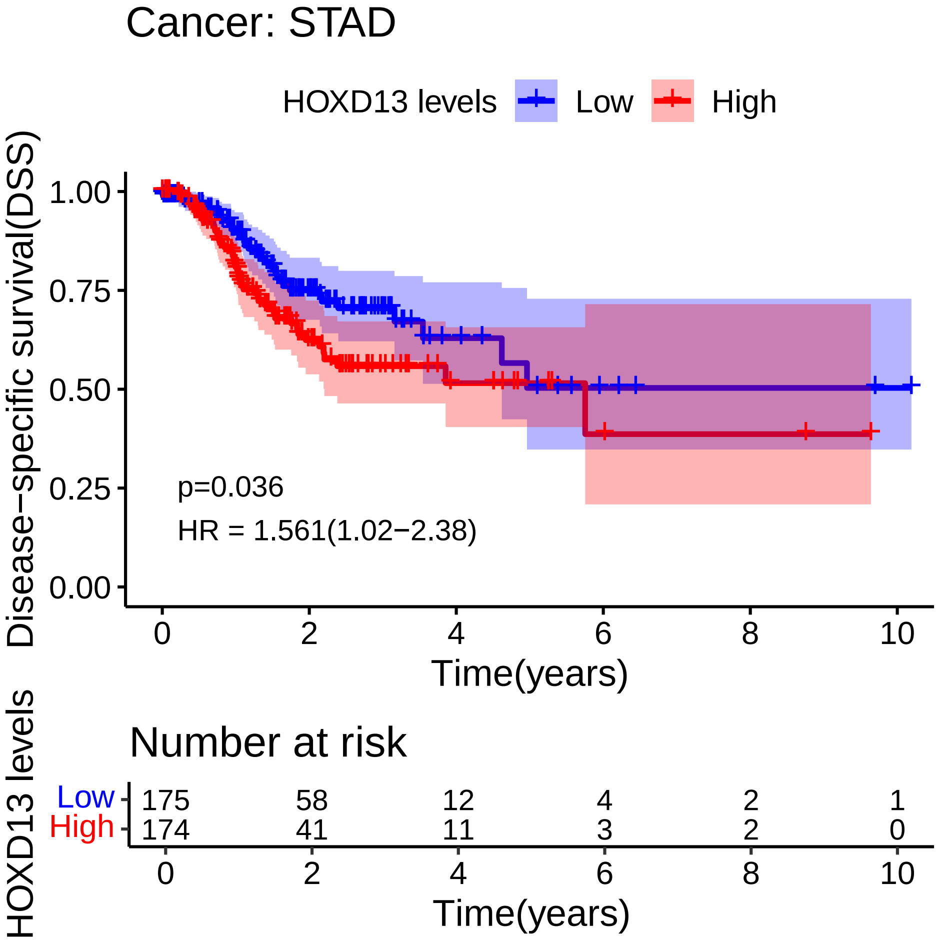

Cancer: STAD
HOXD13 levels
Low
High
Disease−specific survival(DSS)
1.00
0.75
0.50
0.25
0.00
p=0.036
HR = 1.561(1.02−2.38)
0
2
4
6
8
10
Time(years)
Number at risk
HOXD13 levels
Low 175 58 12 4 2 1
High 174 41 11 3 2 0
Time(years)

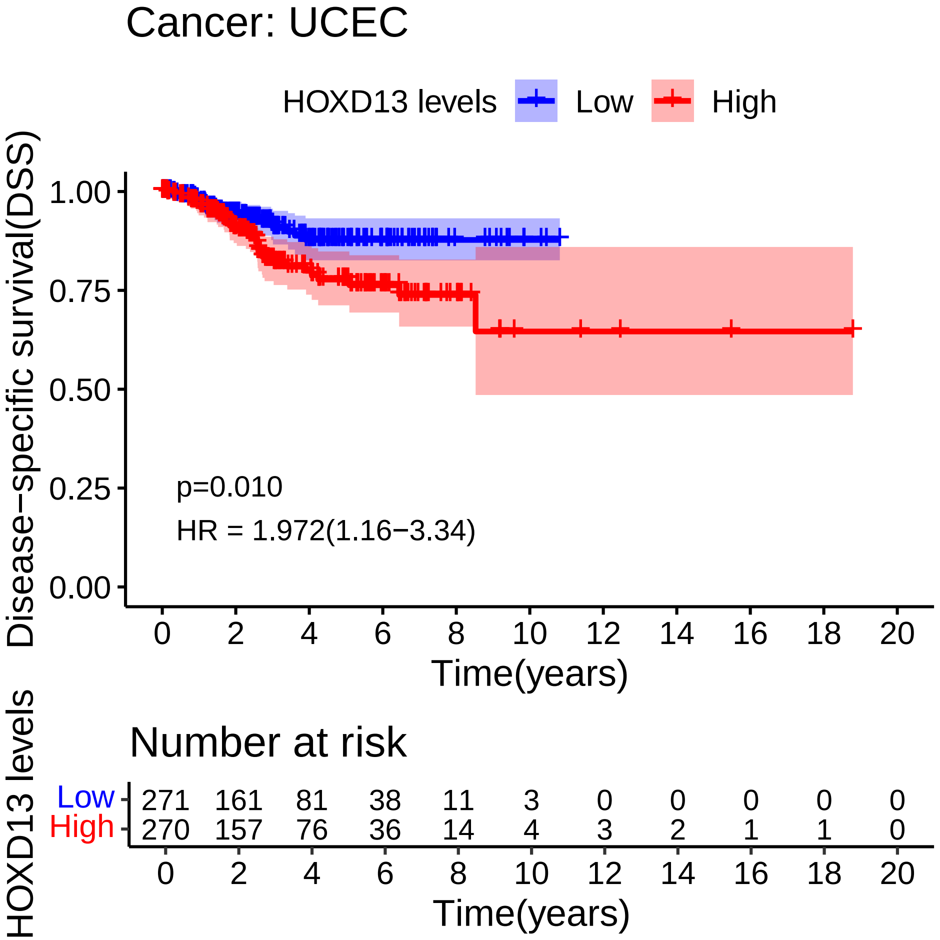

Cancer: UCEC
HOXD13 levels
Low
High
Disease−specific survival(DSS)
1.00
0.75
0.50
0.25
0.00
p=0.010
HR = 1.972(1.16−3.34)
0
2
4
6
8
10
12
14
16
18
20
Time(years)
Number at risk
HOXD13 levels
Low 271 161 81 38 11 3 0 0 0 0 0
High 270 157 76 36 14 4 3 2 1 1 0
Time(years)

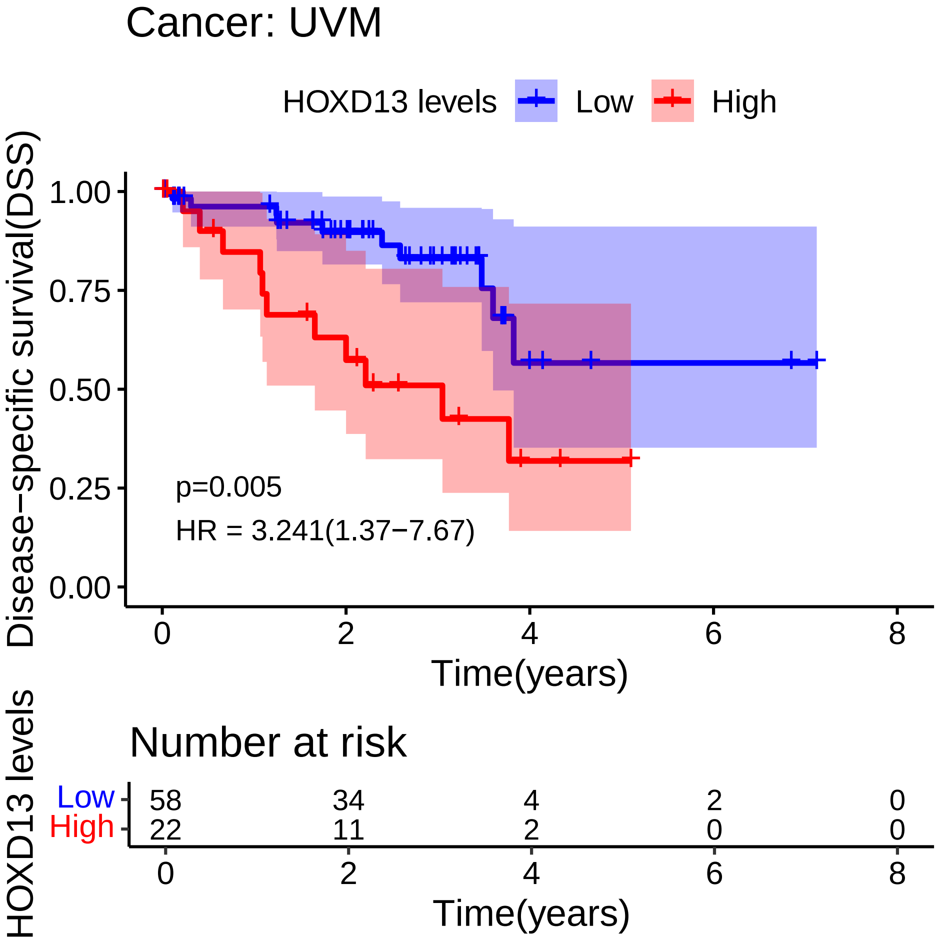

Cancer: UVM
HOXD13 levels
Low
High
Disease−specific survival(DSS)
1.00
0.75
0.50
0.25
0.00
p=0.005
HR = 3.241(1.37−7.67)
0
2
4
6
8
Time(years)
Number at risk
HOXD13 levels
Low 58 34 4 2 0
High 22 11 2 0 0
0
2
4
6
8
Time(years)
