# Supplementary material for: A pan-cancer analysis of homeobox family: expression characteristics and latent significance in prognosis and immune microenvironment
Source: Front Oncol. 2025 Feb 6;15:1521652. doi: 10.3389/fonc.2025.1521652 (PMC11840236; doi:10.3389/fonc.2025.1521652)
Supplement: Supplementary Figure 1 — Mutations of each HOX gene in 32 cancers (TCGA, PanCancer Atlas) by cBioportal. [file DataSheet1.zip › Suppl.files/S4_File.pdf]

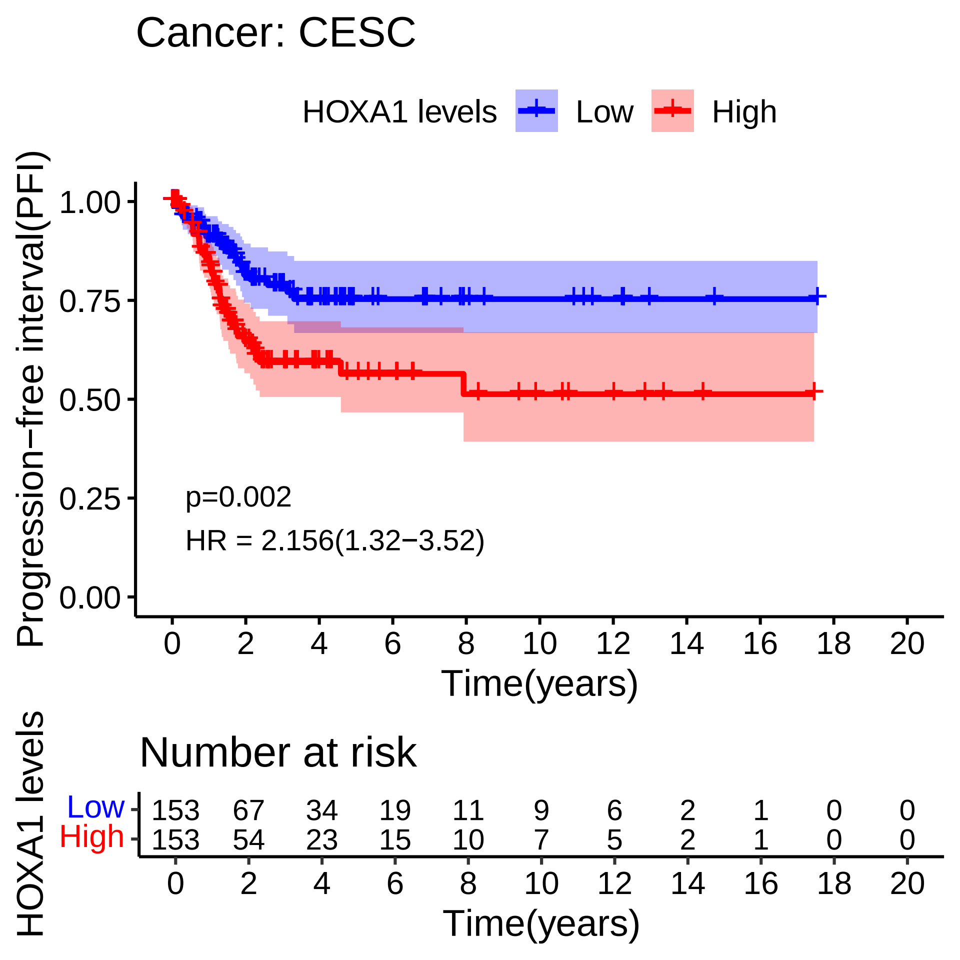
Cancer: CESC
HOXA1 levels
Low
High
Progression−free interval(PFI)
1.00
0.75
0.50
0.25
0.00
p=0.002
HR = 2.156(1.32−3.52)
0 2 4 6 8 10 12 14 16 18 20
Time(years)
Number at risk
HOXA1 levels
Low 153 67 34 19 11 9 6 2 1 0 0
High 153 54 23 15 10 7 5 2 1 0 0
0 2 4 6 8 10 12 14 16 18 20
Time(years)

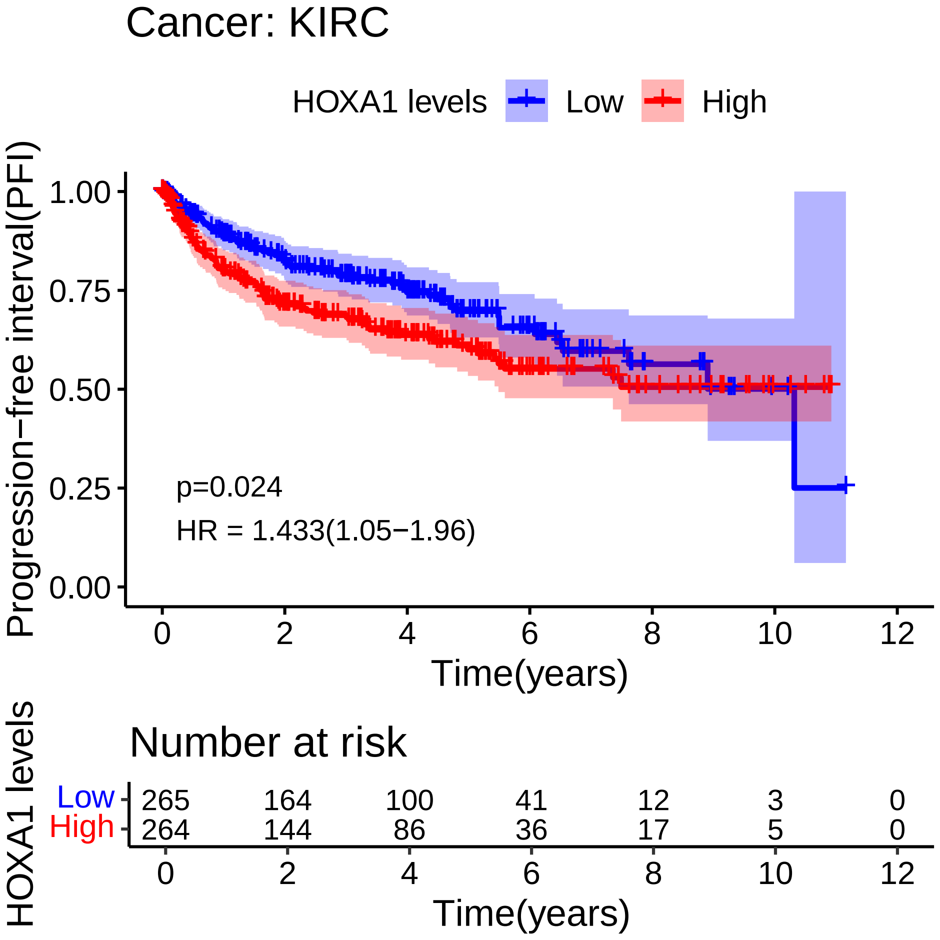
Cancer: KIRC
HOXA1 levels
Low
High
Progression−free interval(PFI)
1.00
0.75
0.50
0.25
0.00
p=0.024
HR = 1.433(1.05−1.96)
0
2
4
6
8
10
12
Time(years)
Number at risk
HOXA1 levels
Low 265 164 100 41 12 3 0
High 264 144 86 36 17 5 0
0
2
4
6
8
10
12
Time(years)

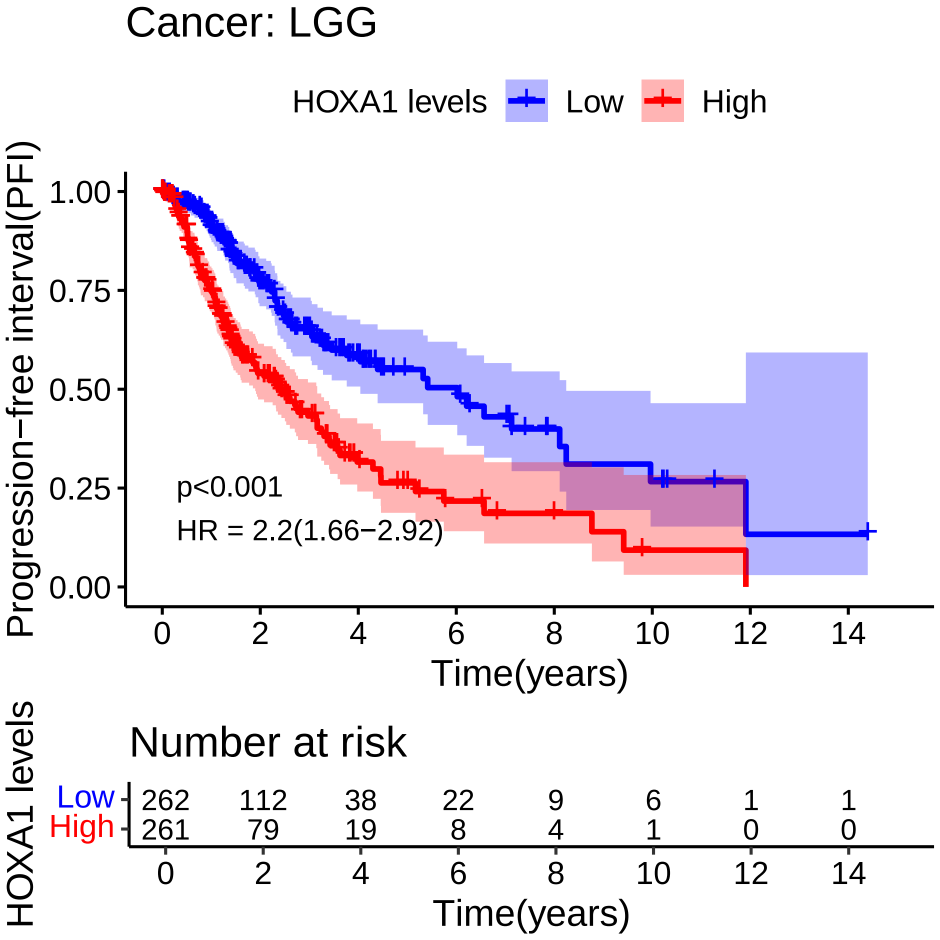
Cancer: LGG
HOXA1 levels
Low
High
Progression−free interval(PFI)
1.00
0.75
0.50
0.25
0.00
p<0.001
HR = 2.2(1.66−2.92)
0
2
4
6
8
10
12
14
Time(years)
Number at risk
HOXA1 levels
Low 262 112 38 22 9 6 1 1
High 261 79 19 8 4 1 0 0
Time(years)

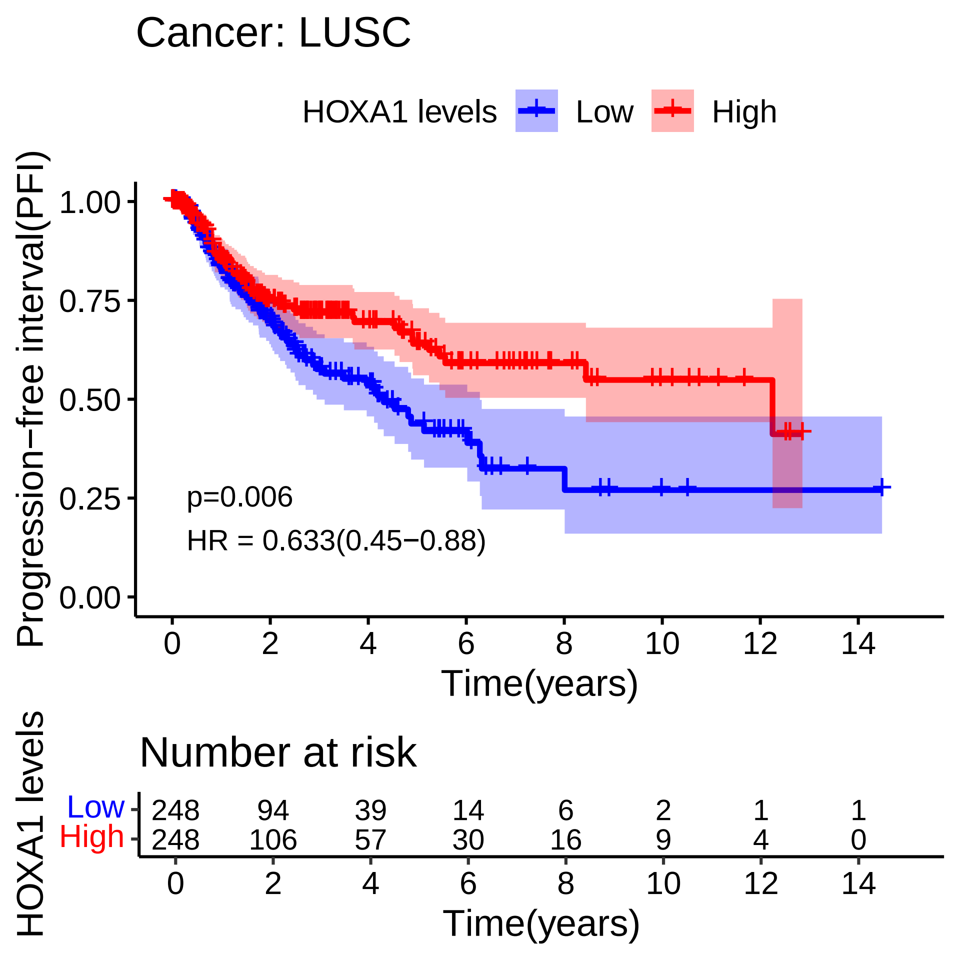
Cancer: LUSC
HOXA1 levels
Low
High
Progression−free interval(PFI)
1.00
0.75
0.50
0.25
0.00
p=0.006
HR = 0.633(0.45−0.88)
0
2
4
6
8
10
12
14
Time(years)
Number at risk
HOXA1 levels
Low 248 94 39 14 6 2 1 1
High 248 106 57 30 16 9 4 0
0
2
4
6
8
10
12
14
Time(years)

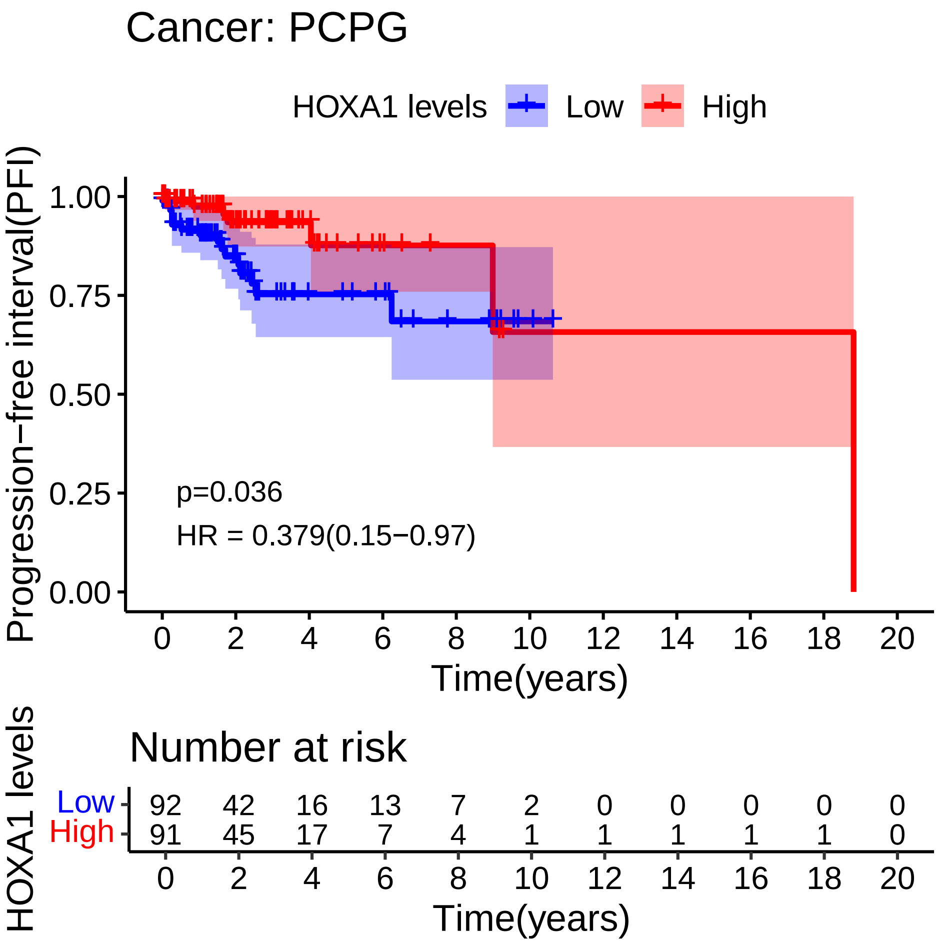
Cancer: PCPG
HOXA1 levels
Low
High
Progression−free interval(PFI)
1.00
0.75
0.50
0.25
0.00
p=0.036
HR = 0.379(0.15−0.97)
0
2
4
6
8
10
12
14
16
18
20
Time(years)
Number at risk
HOXA1 levels
Low 92 42 16 13 7 2 0 0 0 0 0
High 91 45 17 7 4 1 1 1 1 1 0
Time(years)

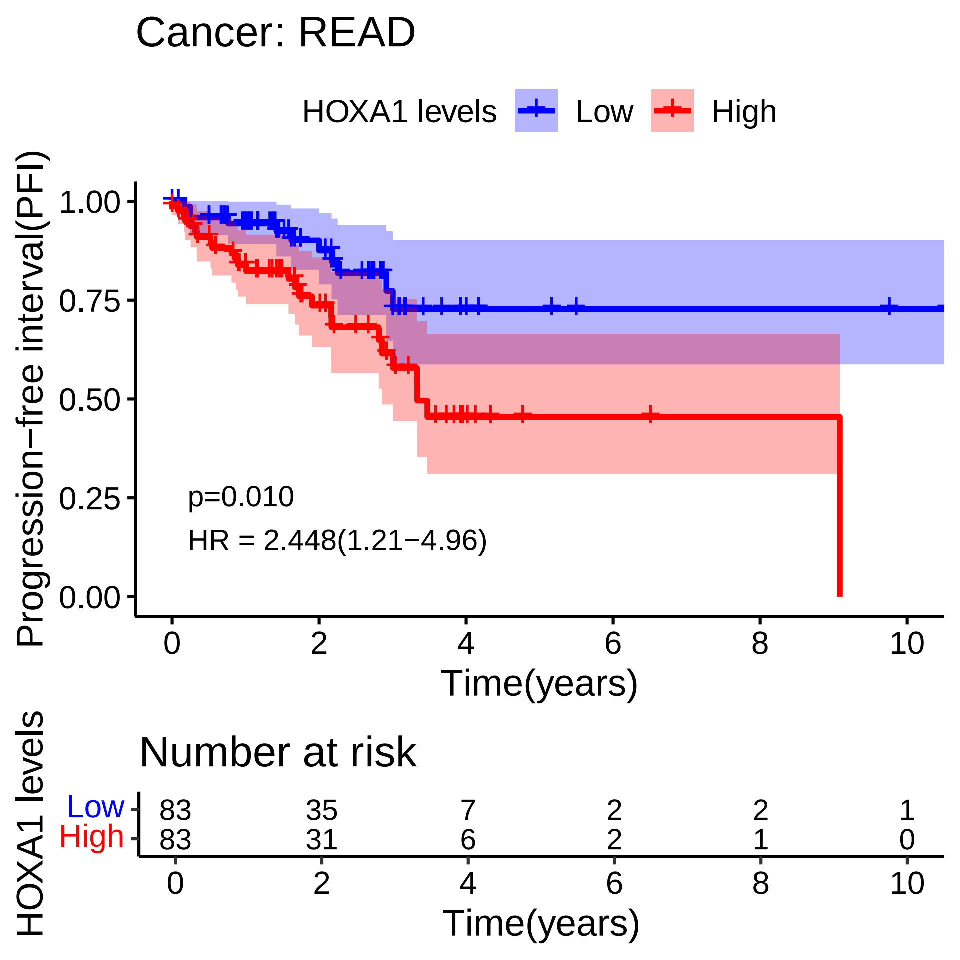

Cancer: READ
HOXA1 levels
Low
High
Progression−free interval(PFI)
1.00
0.75
0.50
0.25
0.00
p=0.010
HR = 2.448(1.21−4.96)
0
2
4
6
8
10
Time(years)
Number at risk
HOXA1 levels
Low 83 35 7 2 2 1
High 83 31 6 2 1 0
0
2
4
6
8
10
Time(years)

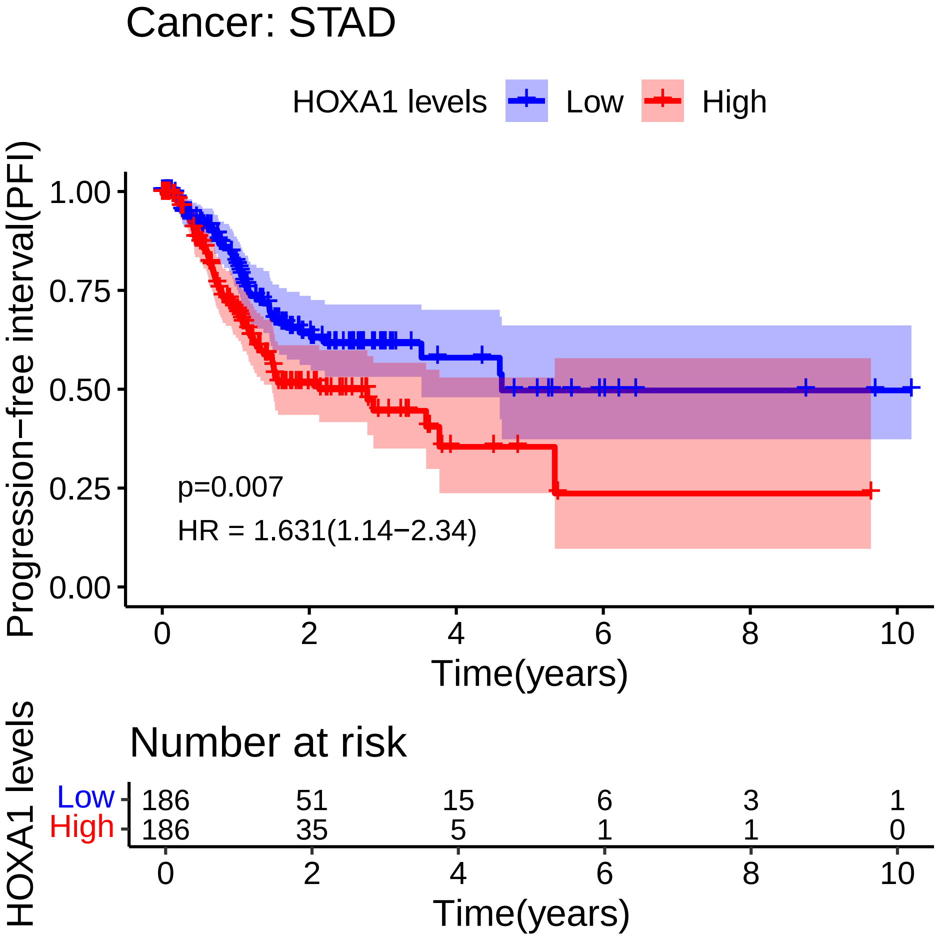

Cancer: STAD
HOXA1 levels
Low
High
Progression−free interval(PFI)
1.00
0.75
0.50
0.25
0.00
p=0.007
HR = 1.631(1.14−2.34)
0
2
4
6
8
10
Time(years)
Number at risk
HOXA1 levels
Low 186 51 15 6 3 1
High 186 35 5 1 1 0
Time(years)

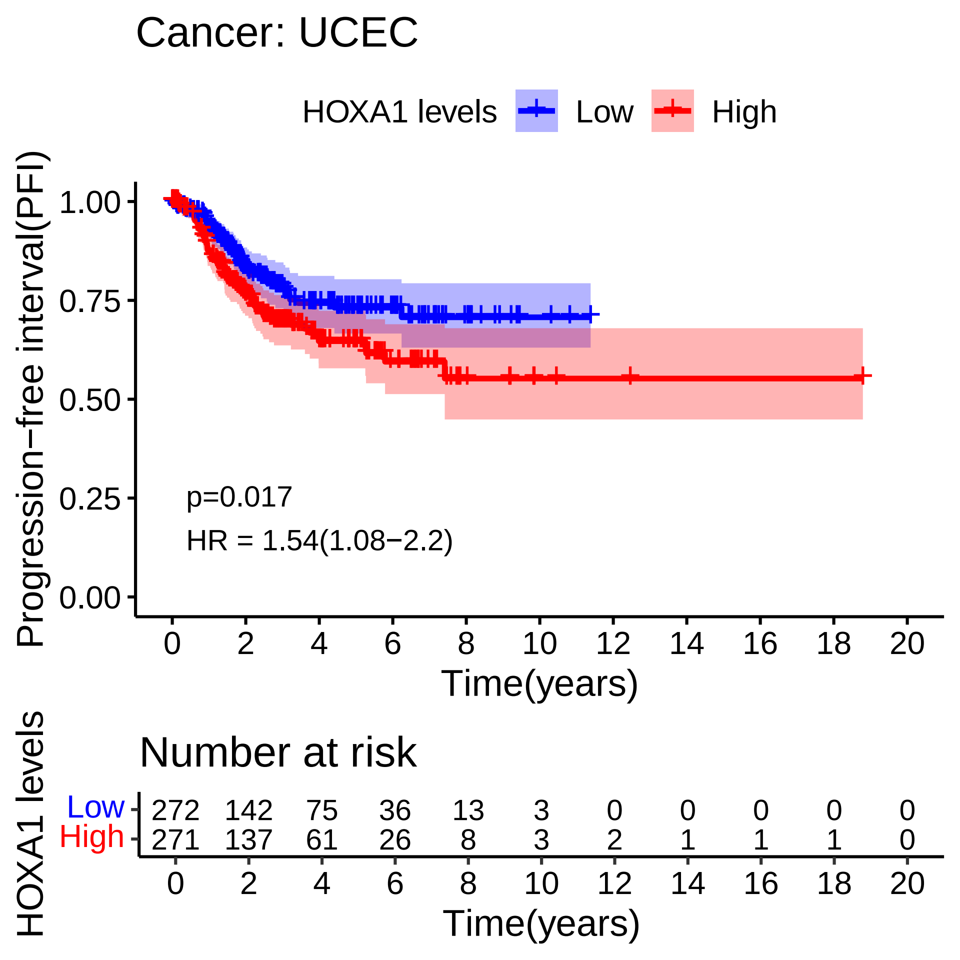
Cancer: UCEC
HOXA1 levels
Low
High
Progression−free interval(PFI)
1.00
0.75
0.50
0.25
0.00
p=0.017
HR = 1.54(1.08−2.2)
0
2
4
6
8
10
12
14
16
18
20
Time(years)
Number at risk
HOXA1 levels
Low
High
272 142 75 36 13 3 0 0 0 0 0
271 137 61 26 8 3 2 1 1 1 0
0
2
4
6
8
10
12
14
16
18
20
Time(years)

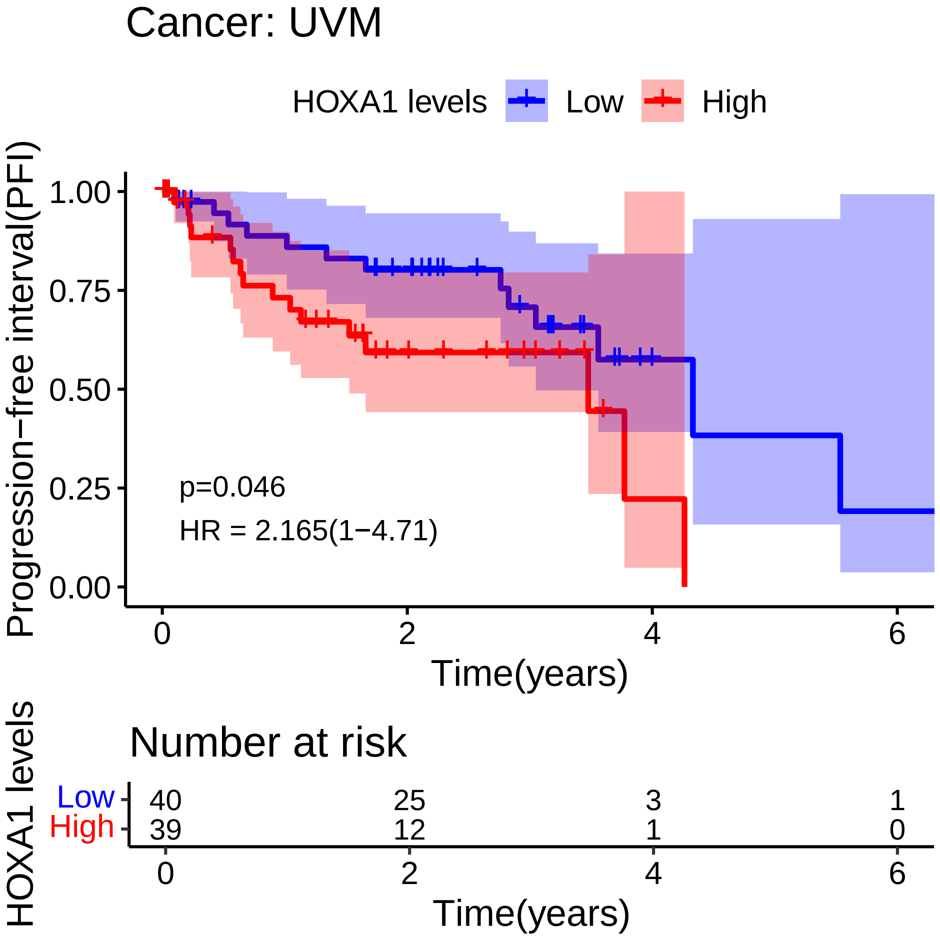

Cancer: UVM
HOXA1 levels
Low
High
Progression−free interval(PFI)
1.00
0.75
0.50
0.25
0.00
p=0.046
HR = 2.165(1−4.71)
0
2
4
6
Time(years)
Number at risk
HOXA1 levels
Low 40 25 3 1
High 39 12 1 0
0
2
4
6
Time(years)

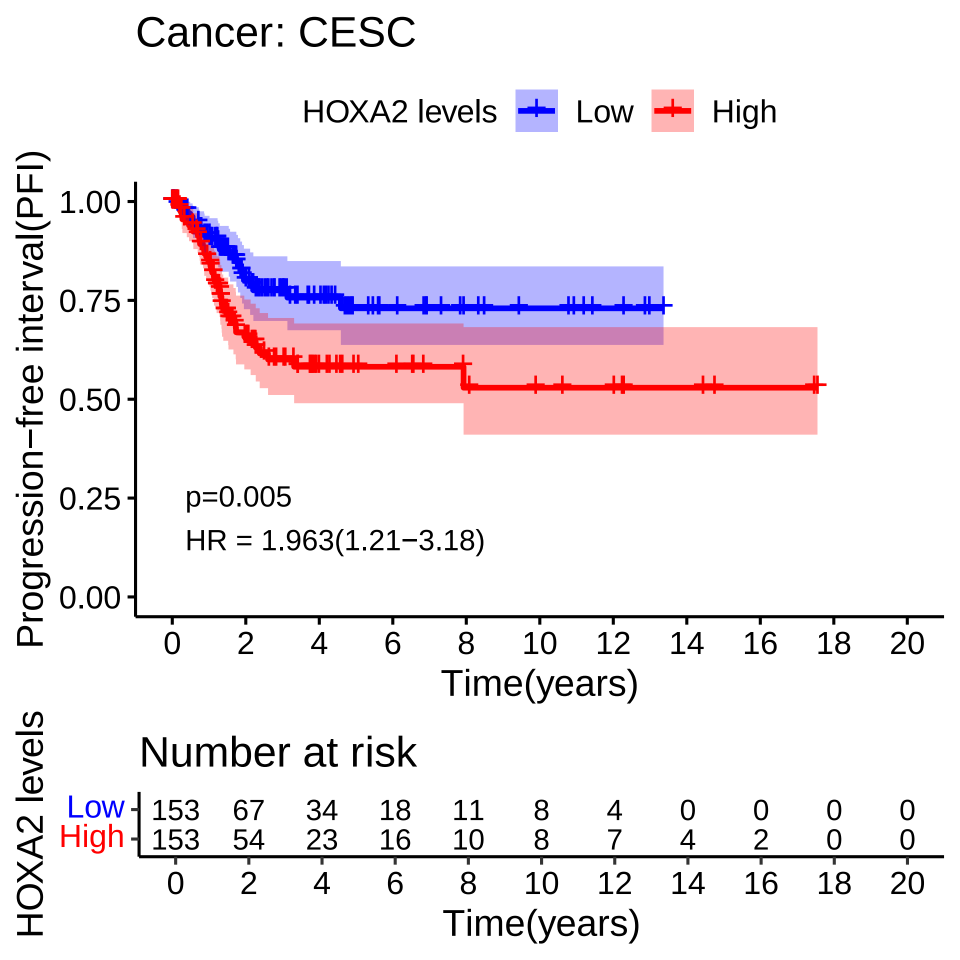

Cancer: CESC
HOXA2 levels
Low
High
Progression−free interval(PFI)
1.00
0.75
0.50
0.25
0.00
p=0.005
HR = 1.963(1.21−3.18)
0 2 4 6 8 10 12 14 16 18 20
Time(years)
Number at risk
HOXA2 levels
Low 153 67 34 18 11 8 4 0 0 0 0
High 153 54 23 16 10 8 7 4 2 0 0
0 2 4 6 8 10 12 14 16 18 20
Time(years)

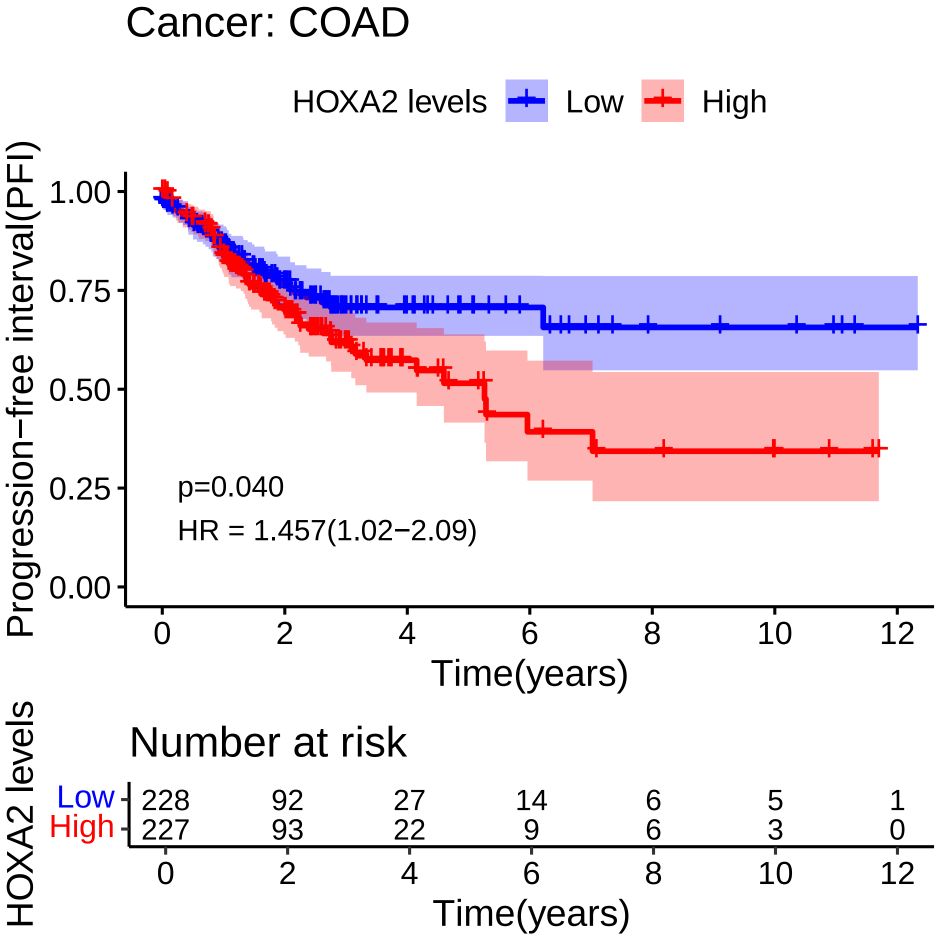

Cancer: COAD
HOXA2 levels
Low
High
Progression−free interval(PFI)
1.00
0.75
0.50
0.25
0.00
p=0.040
HR = 1.457(1.02−2.09)
0
2
4
6
8
10
12
Time(years)
Number at risk
HOXA2 levels
Low
High
228 92 27 14 6 5 1
227 93 22 9 6 3 0
0
2
4
6
8
10
12
Time(years)

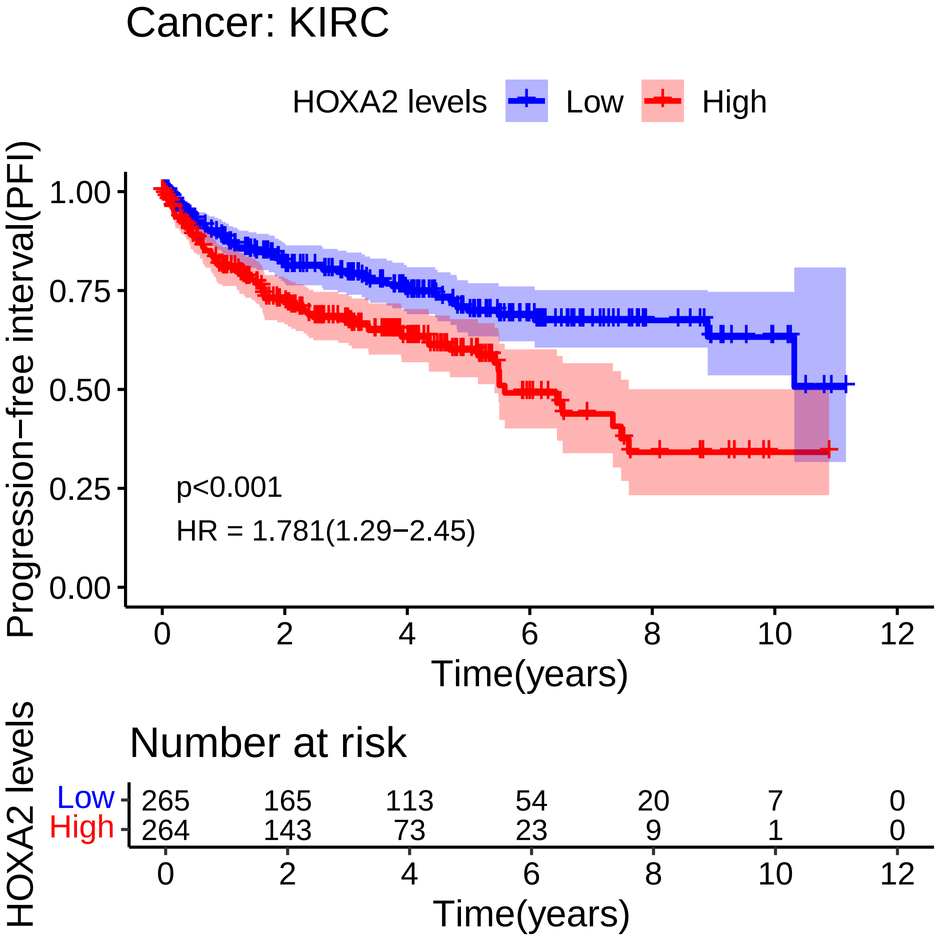
Cancer: KIRC
HOXA2 levels
Low
High
Progression−free interval(PFI)
1.00
0.75
0.50
0.25
0.00
p<0.001
HR = 1.781(1.29−2.45)
0
2
4
6
8
10
12
Time(years)
Number at risk
HOXA2 levels
Low
High
265 165 113 54 20 7 0
264 143 73 23 9 1 0
0
2
4
6
8
10
12
Time(years)

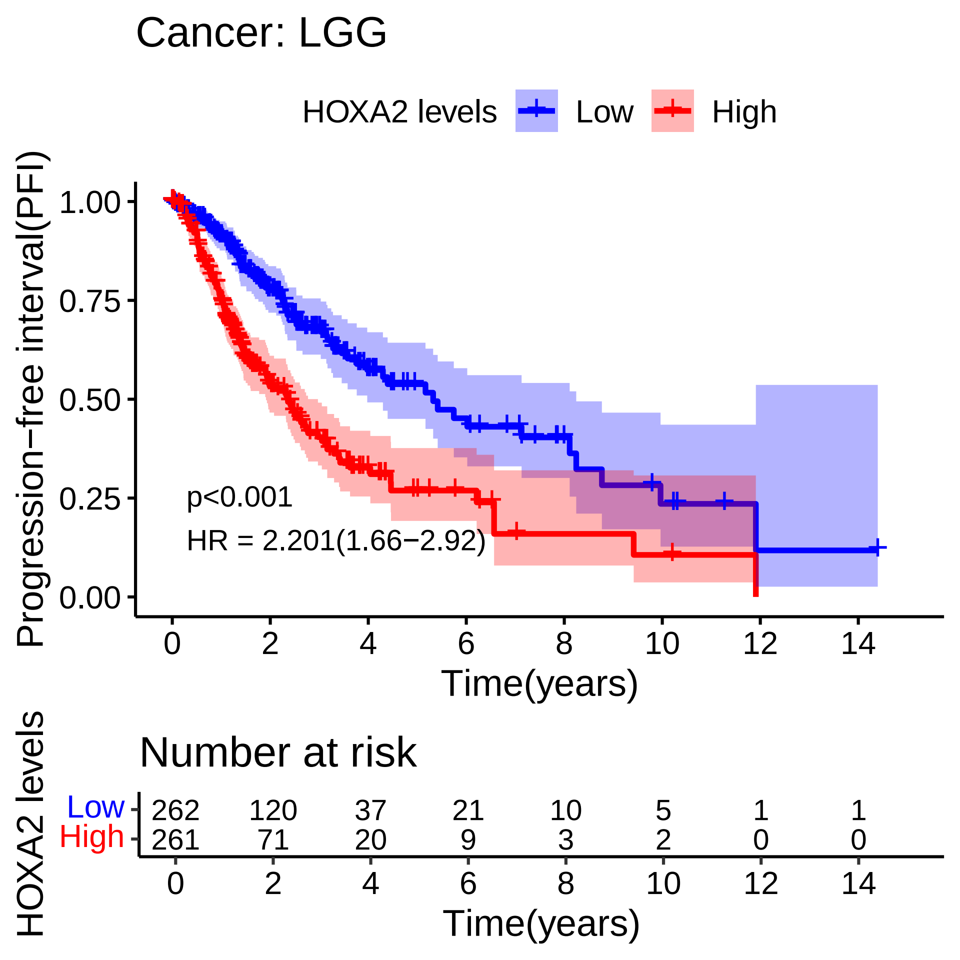
Cancer: LGG
HOXA2 levels
Low
High
Progression−free interval(PFI)
1.00
0.75
0.50
0.25
0.00
p<0.001
HR = 2.201(1.66−2.92)
0
2
4
6
8
10
12
14
Time(years)
Number at risk
HOXA2 levels
Low 262 120 37 21 10 5 1 1
High 261 71 20 9 3 2 0 0
0
2
4
6
8
10
12
14
Time(years)

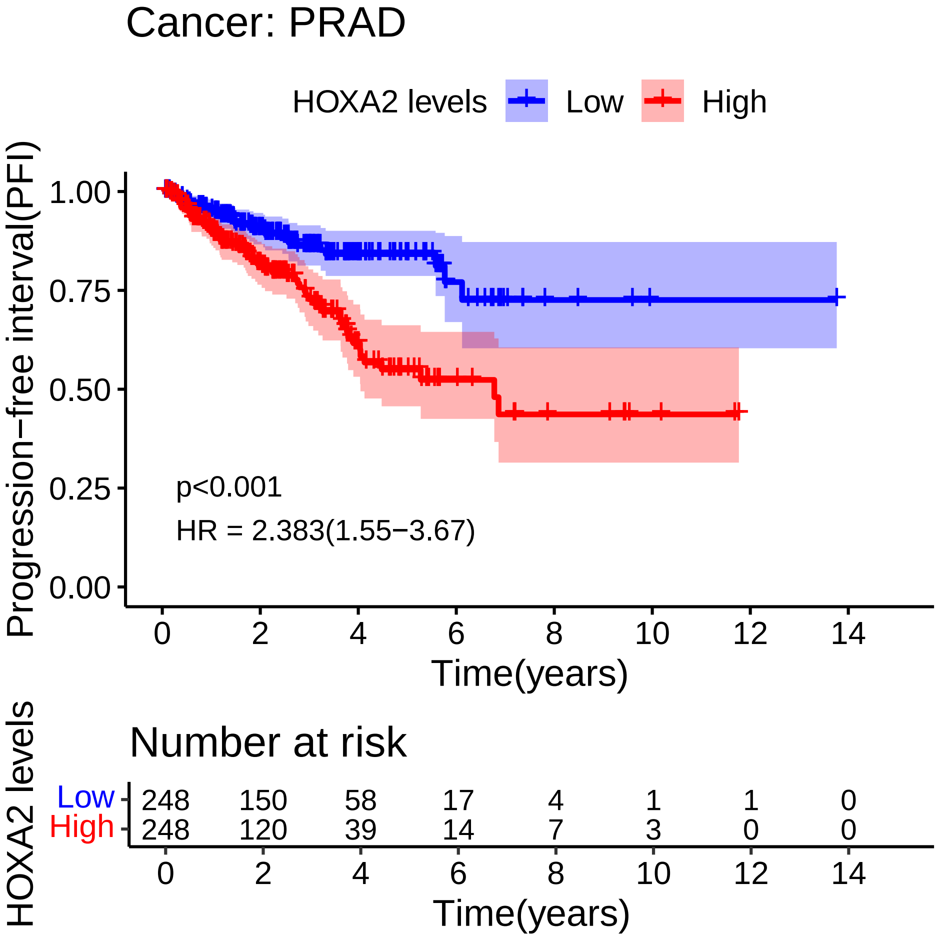

Cancer: PRAD
HOXA2 levels
Low
High
Progression−free interval(PFI)
1.00
0.75
0.50
0.25
0.00
p<0.001
HR = 2.383(1.55−3.67)
0
2
4
6
8
10
12
14
Time(years)
Number at risk
HOXA2 levels
Low 248 150 58 17 4 1 1 0
High 248 120 39 14 7 3 0 0
0
2
4
6
8
10
12
14
Time(years)

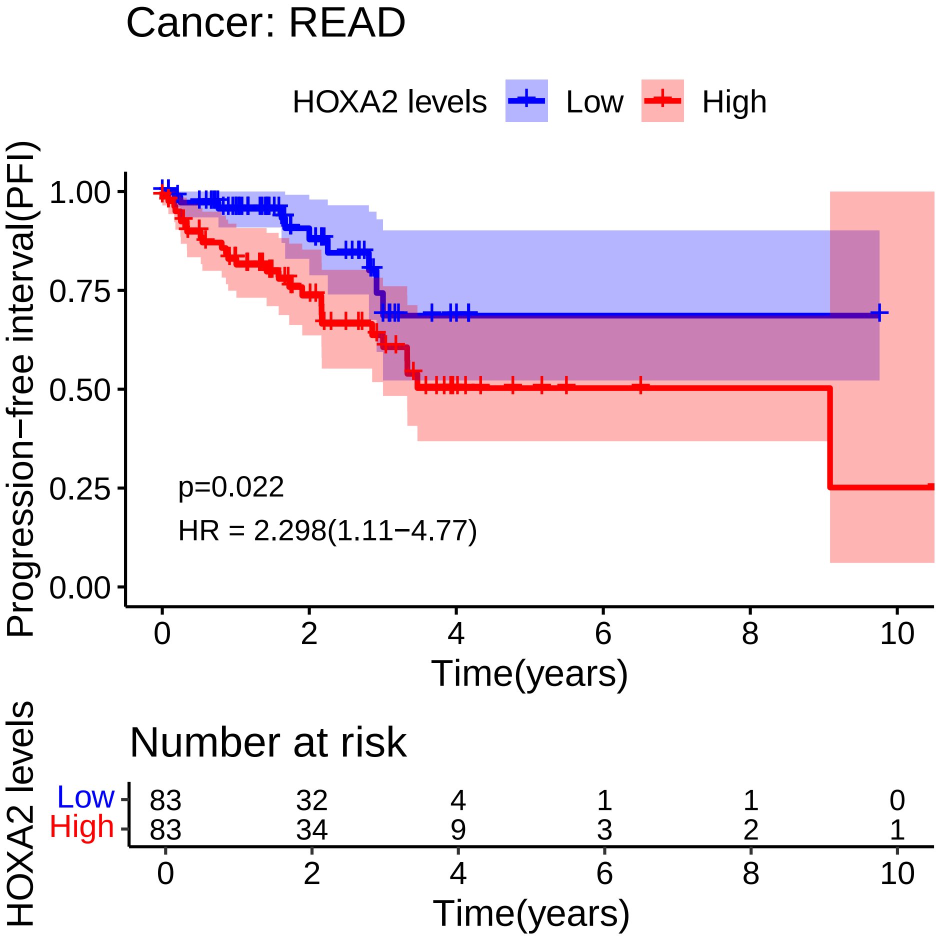

Cancer: READ
HOXA2 levels
Low
High
Progression−free interval(PFI)
1.00
0.75
0.50
0.25
0.00
p=0.022
HR = 2.298(1.11−4.77)
0
2
4
6
8
10
Time(years)
Number at risk
HOXA2 levels
Low 83 32 4 1 1 0
High 83 34 9 3 2 1
Time(years)

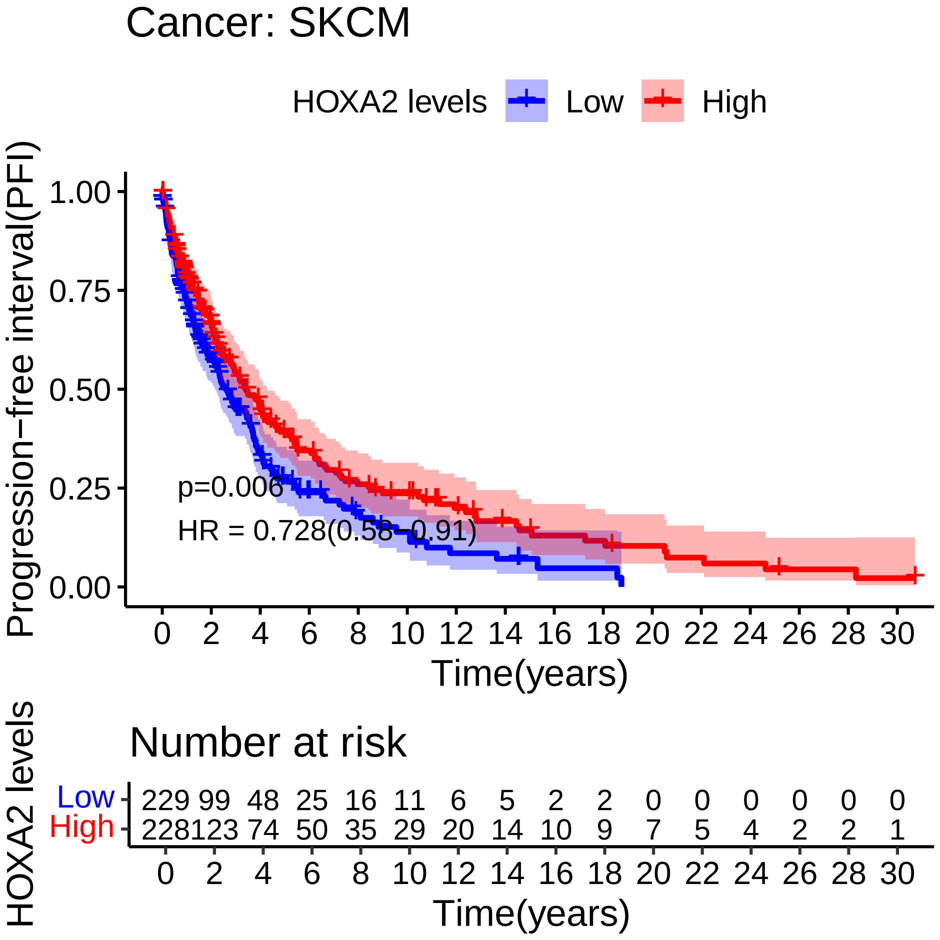

Cancer: SKCM
HOXA2 levels
Low
High
Progression−free interval(PFI)
1.00
0.75
0.50
0.25
0.00
p=0.006
HR = 0.728(0.58−0.91)
0 2 4 6 8 10 12 14 16 18 20 22 24 26 28 30
Time(years)
Number at risk
HOXA2 levels
Low 229 99 48 25 16 11 6 5 2 2 0 0 0 0 0 0
High 228 123 74 50 35 29 20 14 10 9 7 5 4 2 2 1
0 2 4 6 8 10 12 14 16 18 20 22 24 26 28 30
Time(years)

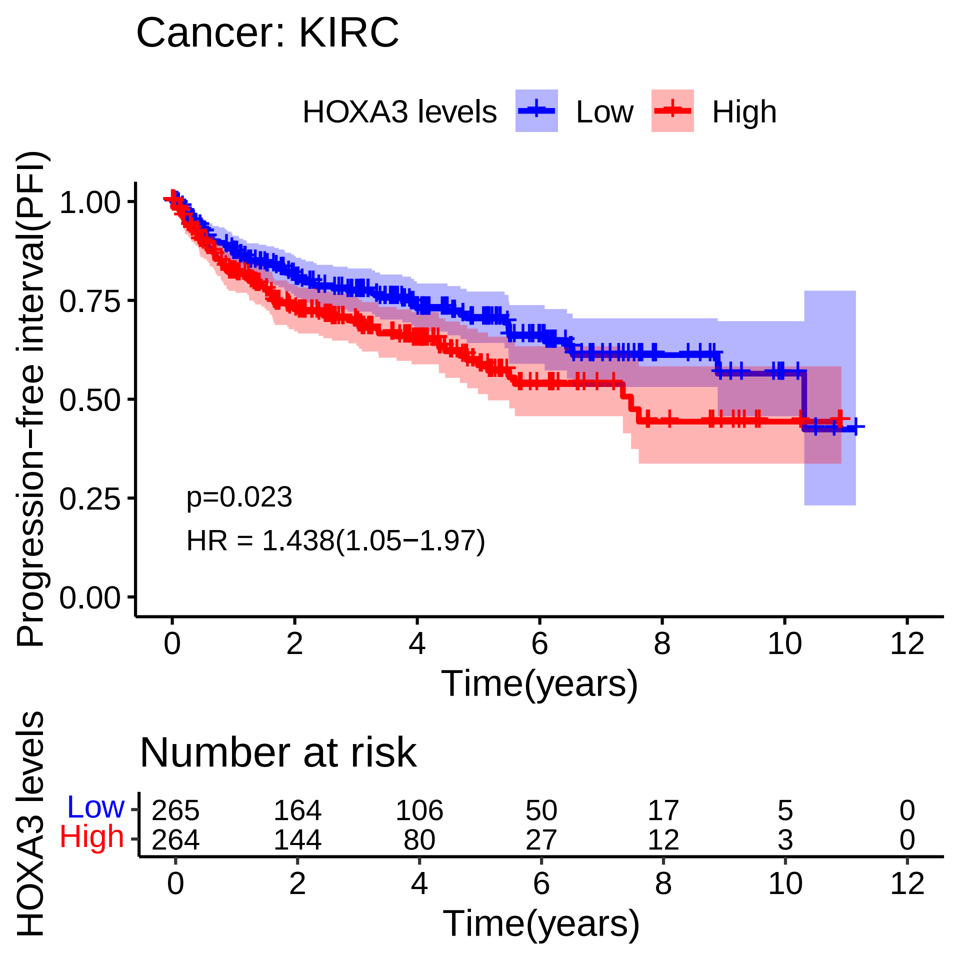

Cancer: KIRC
HOXA3 levels
Low
High
Progression−free interval(PFI)
1.00
0.75
0.50
0.25
0.00
p=0.023
HR = 1.438(1.05−1.97)
0
2
4
6
8
10
12
Time(years)
Number at risk
HOXA3 levels
Low
High
265 164 106 50 17 5 0
264 144 80 27 12 3 0
0
2
4
6
8
10
12
Time(years)

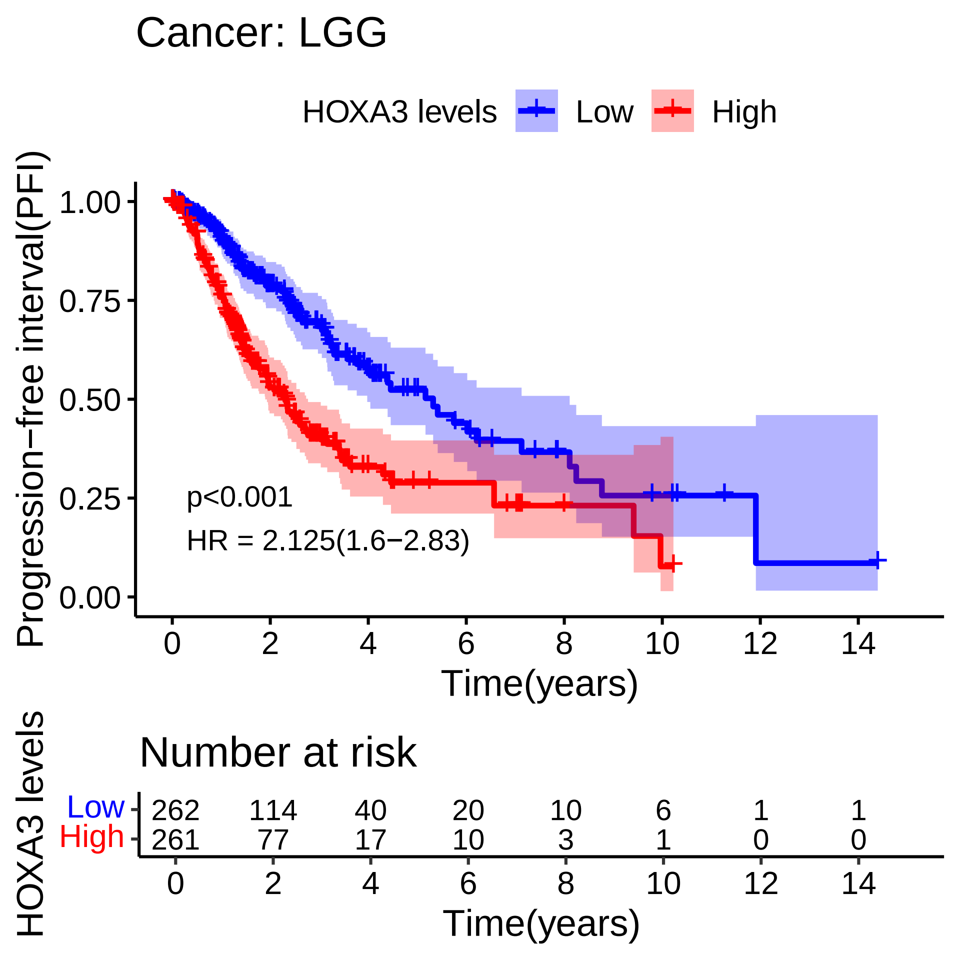

Cancer: LGG
HOXA3 levels
Low
High
Progression−free interval(PFI)
1.00
0.75
0.50
0.25
0.00
p<0.001
HR = 2.125(1.6−2.83)
0
2
4
6
8
10
12
14
Time(years)
Number at risk
HOXA3 levels
Low 262 114 40 20 10 6 1 1
High 261 77 17 10 3 1 0 0
0
2
4
6
8
10
12
14
Time(years)

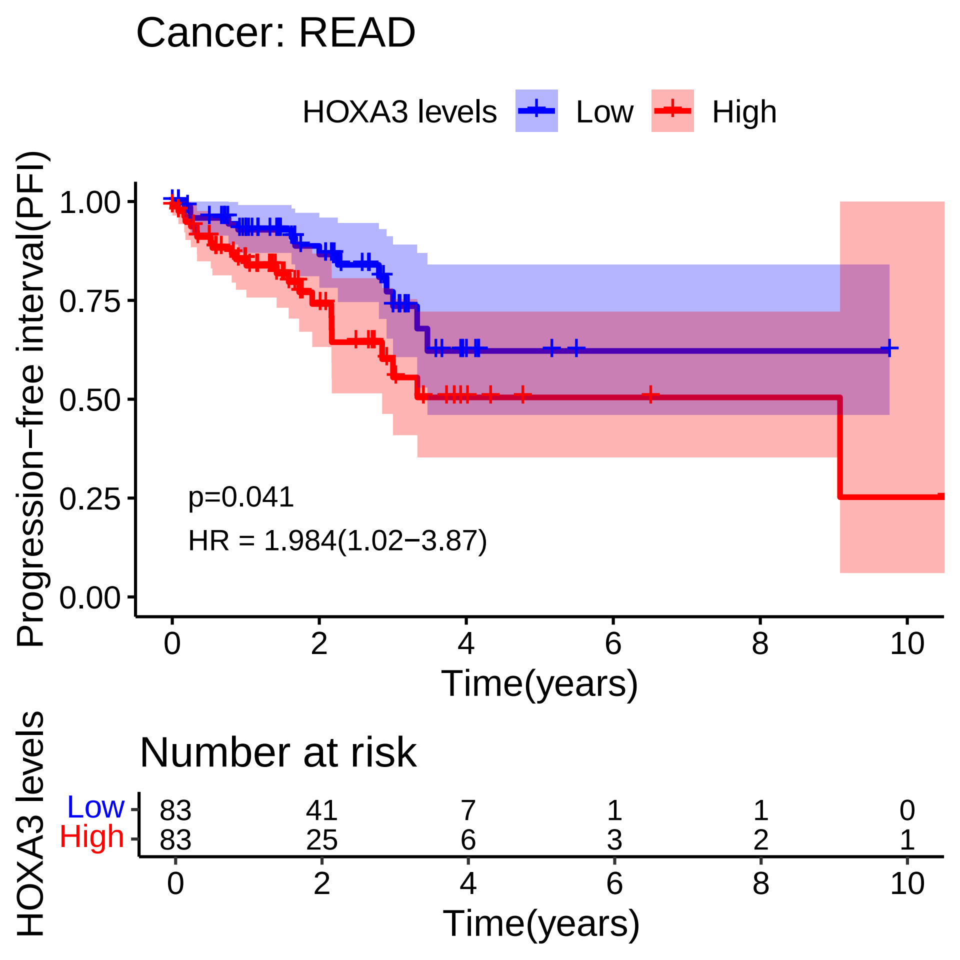

Cancer: READ
HOXA3 levels
Low
High
Progression−free interval(PFI)
1.00
0.75
0.50
0.25
0.00
p=0.041
HR = 1.984(1.02−3.87)
0
2
4
6
8
10
Time(years)
Number at risk
HOXA3 levels
Low 83 41 7 1 1 0
High 83 25 6 3 2 1
0
2
4
6
8
10
Time(years)

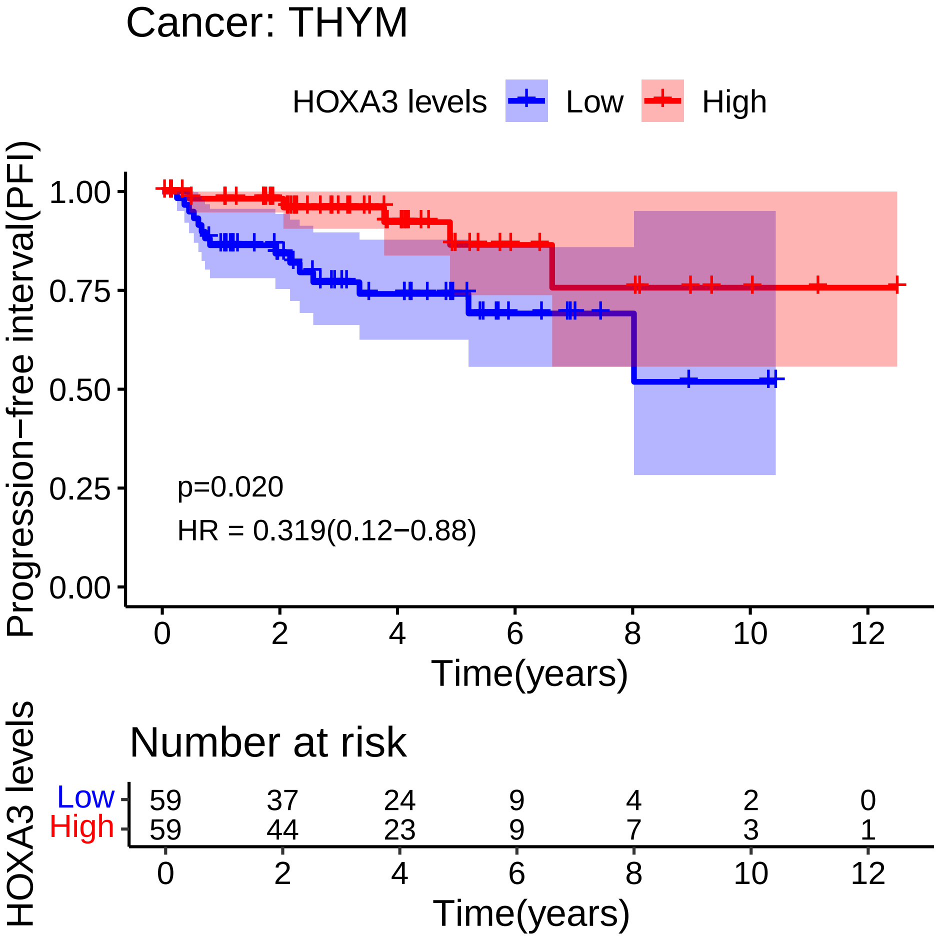

Cancer: THYM
HOXA3 levels
Low
High
Progression−free interval(PFI)
1.00
0.75
0.50
0.25
0.00
p=0.020
HR = 0.319(0.12−0.88)
0
2
4
6
8
10
12
Time(years)
Number at risk
HOXA3 levels
Low 59 37 24 9 4 2 0
High 59 44 23 9 7 3 1
0
2
4
6
8
10
12
Time(years)

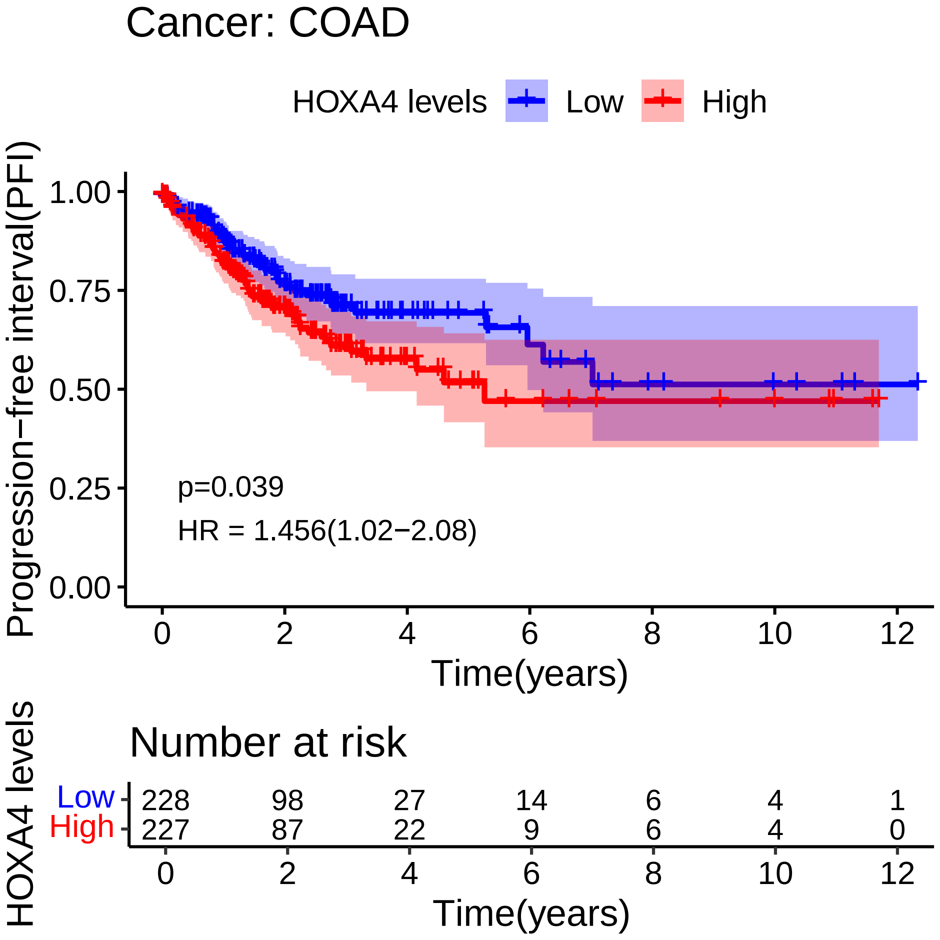
Cancer: COAD
HOXA4 levels
Low
High
Progression−free interval(PFI)
1.00
0.75
0.50
0.25
0.00
p=0.039
HR = 1.456(1.02−2.08)
0
2
4
6
8
10
12
Time(years)
Number at risk
HOXA4 levels
Low 228 98 27 14 6 4 1
High 227 87 22 9 6 4 0
Time(years)

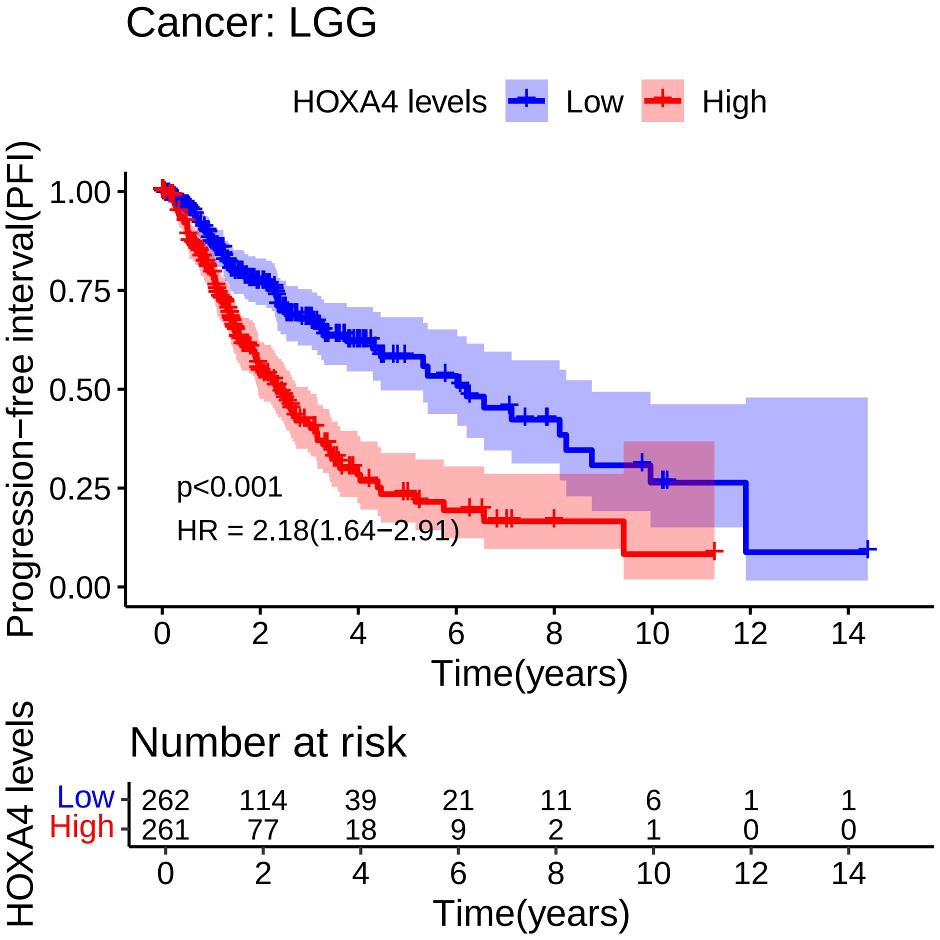
Cancer: LGG
HOXA4 levels
Low
High
Progression−free interval(PFI)
1.00
0.75
0.50
0.25
0.00
p<0.001
HR = 2.18(1.64−2.91)
0
2
4
6
8
10
12
14
Time(years)
Number at risk
HOXA4 levels
Low 262 114 39 21 11 6 1 1
High 261 77 18 9 2 1 0 0
Time(years)

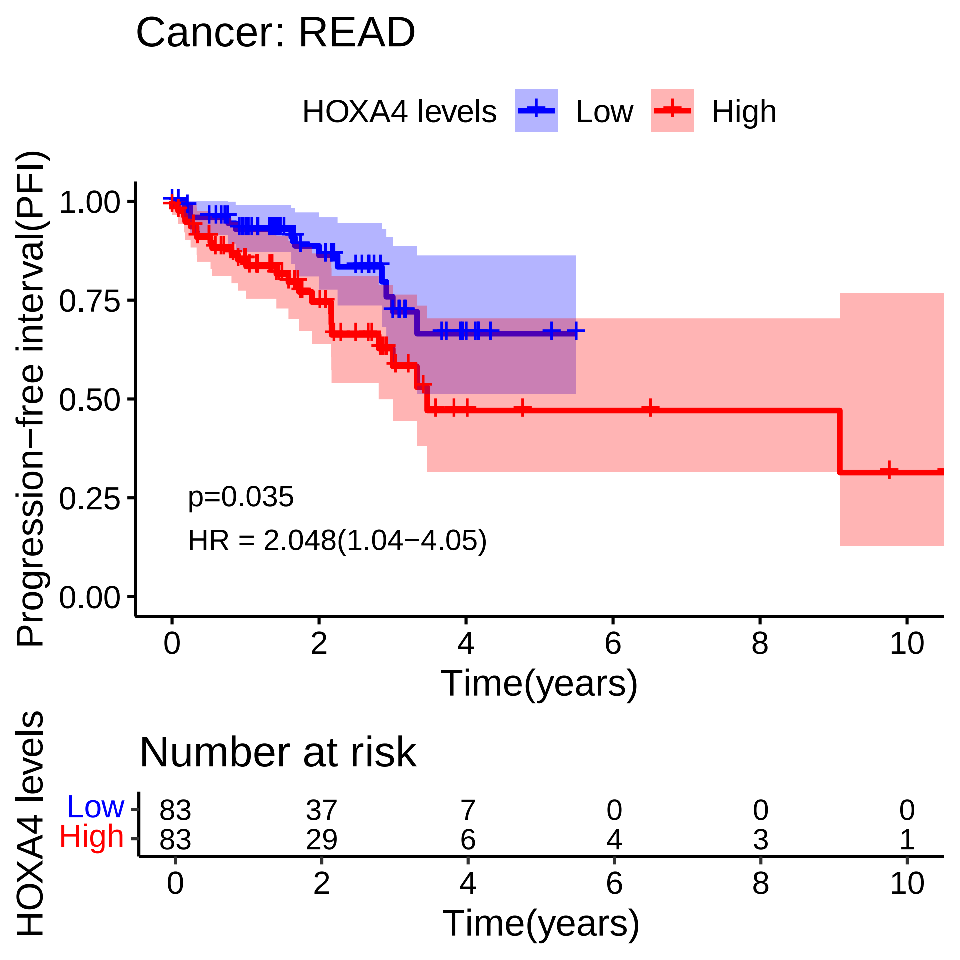
Cancer: READ
HOXA4 levels
Low
High
Progression−free interval(PFI)
1.00
0.75
0.50
0.25
0.00
p=0.035
HR = 2.048(1.04−4.05)
0
2
4
6
8
10
Time(years)
Number at risk
HOXA4 levels
Low
High
83 37 7 0 0 0
83 29 6 4 3 1
Time(years)

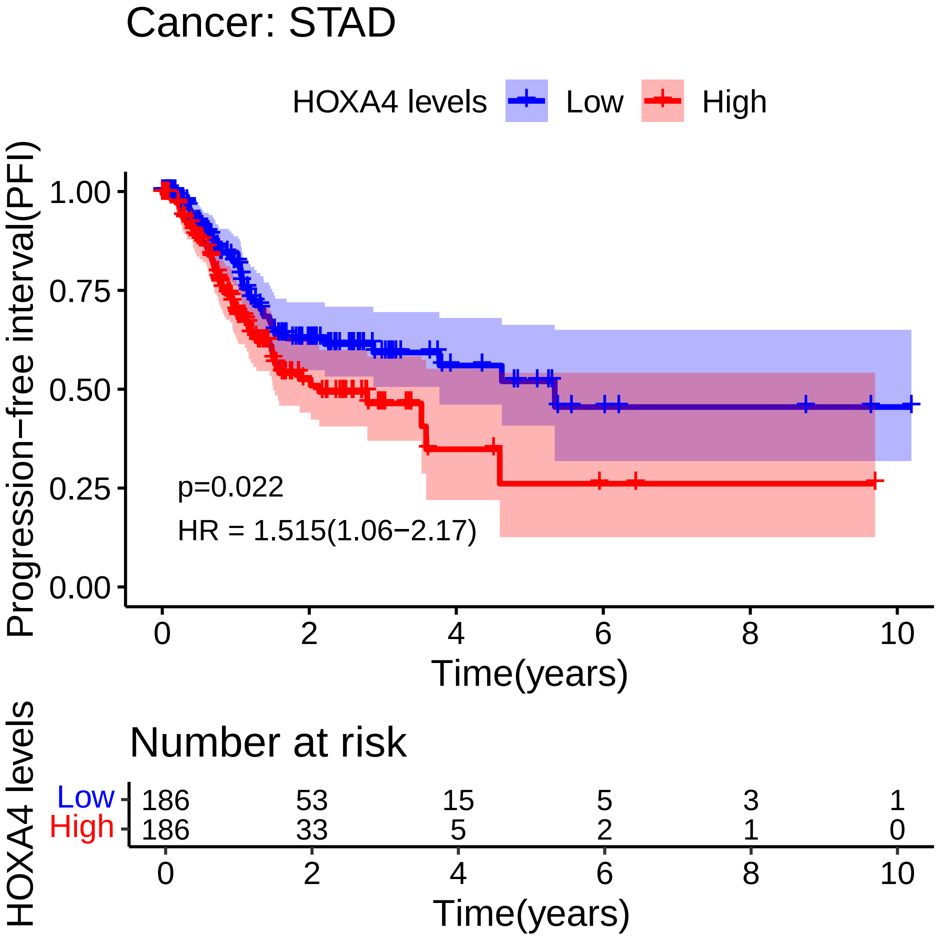

Cancer: STAD
HOXA4 levels
Low
High
Progression−free interval(PFI)
1.00
0.75
0.50
0.25
0.00
p=0.022
HR = 1.515(1.06−2.17)
0
2
4
6
8
10
Time(years)
Number at risk
HOXA4 levels
Low 186 53 15 5 3 1
High 186 33 5 2 1 0
0
2
4
6
8
10
Time(years)

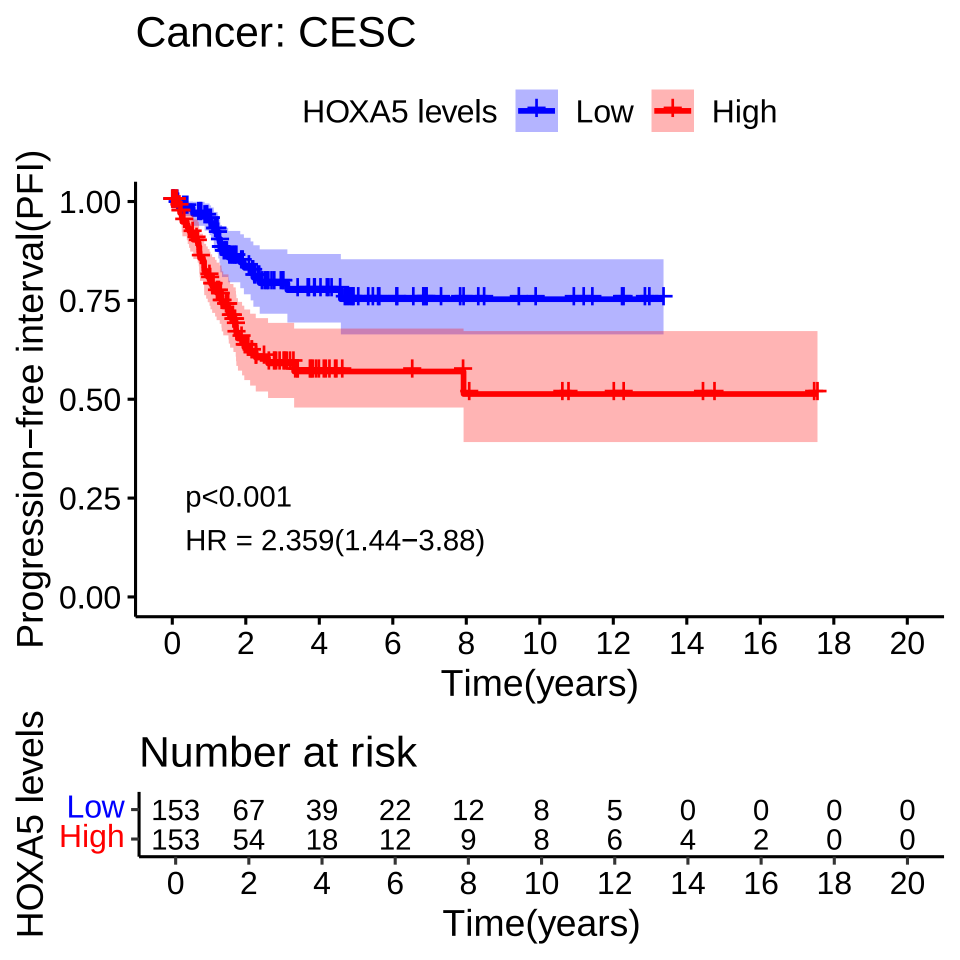

Cancer: CESC
HOXA5 levels
Low
High
Progression−free interval(PFI)
1.00
0.75
0.50
0.25
0.00
p<0.001
HR = 2.359(1.44−3.88)
0
2
4
6
8
10
12
14
16
18
20
Time(years)
Number at risk
HOXA5 levels
Low 153 67 39 22 12 8 5 0 0 0 0
High 153 54 18 12 9 8 6 4 2 0 0
Time(years)

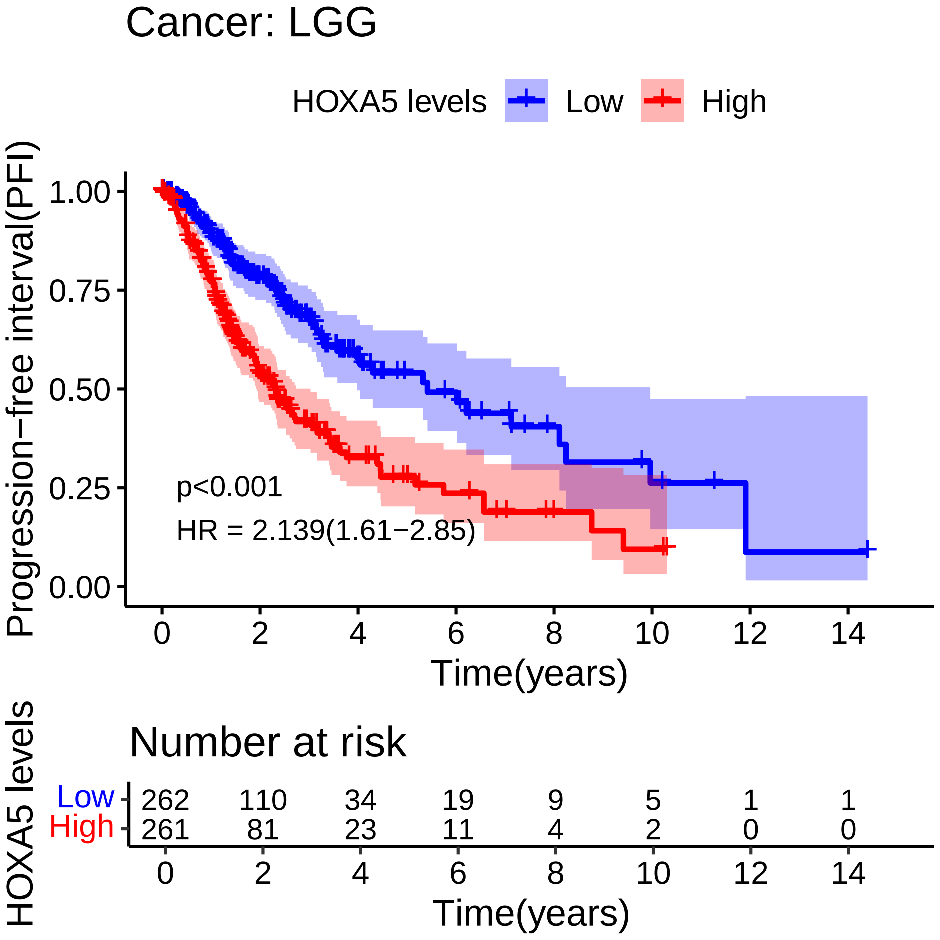
Cancer: LGG
HOXA5 levels
Low
High
Progression−free interval(PFI)
1.00
0.75
0.50
0.25
0.00
p<0.001
HR = 2.139(1.61−2.85)
0
2
4
6
8
10
12
14
Time(years)
Number at risk
HOXA5 levels
Low 262 110 34 19 9 5 1 1
High 261 81 23 11 4 2 0 0
0
2
4
6
8
10
12
14
Time(years)

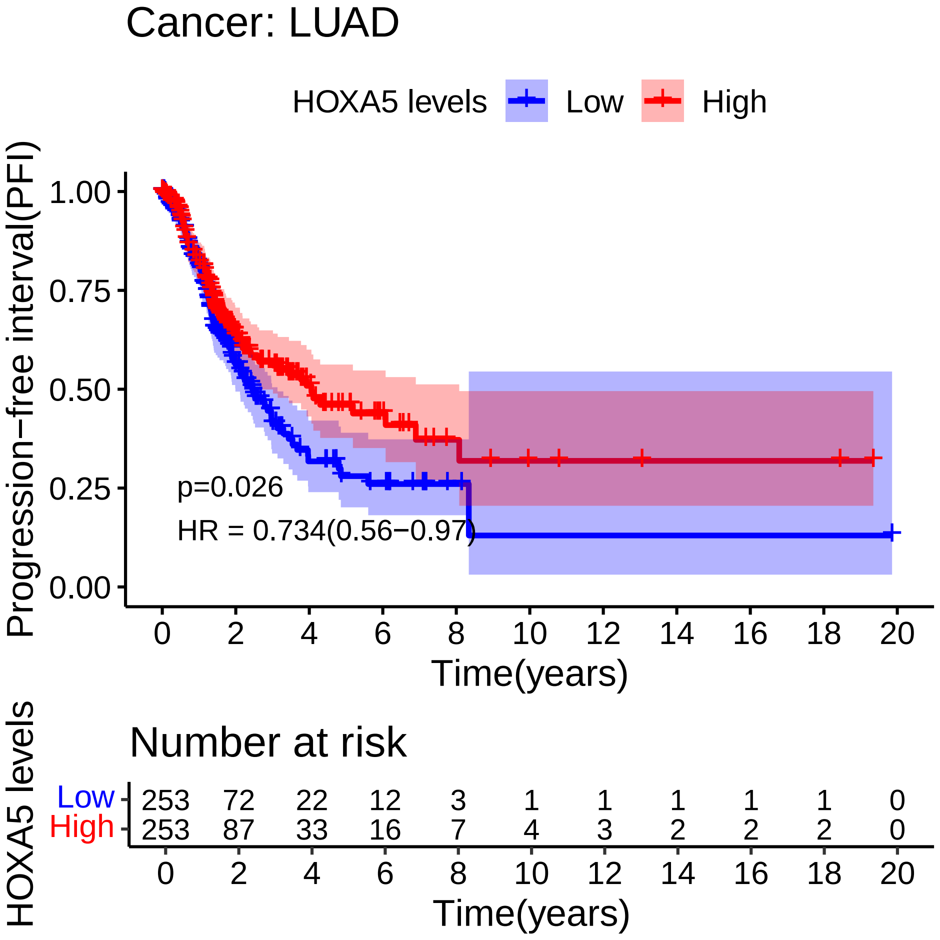
Cancer: LUAD
HOXA5 levels
Low
High
Progression−free interval(PFI)
1.00
0.75
0.50
0.25
0.00
p=0.026
HR = 0.734(0.56−0.97)
0
2
4
6
8
10
12
14
16
18
20
Time(years)
Number at risk
HOXA5 levels
Low 253 72 22 12 3 1 1 1 1 1 0
High 253 87 33 16 7 4 3 2 2 2 0
0
2
4
6
8
10
12
14
16
18
20
Time(years)

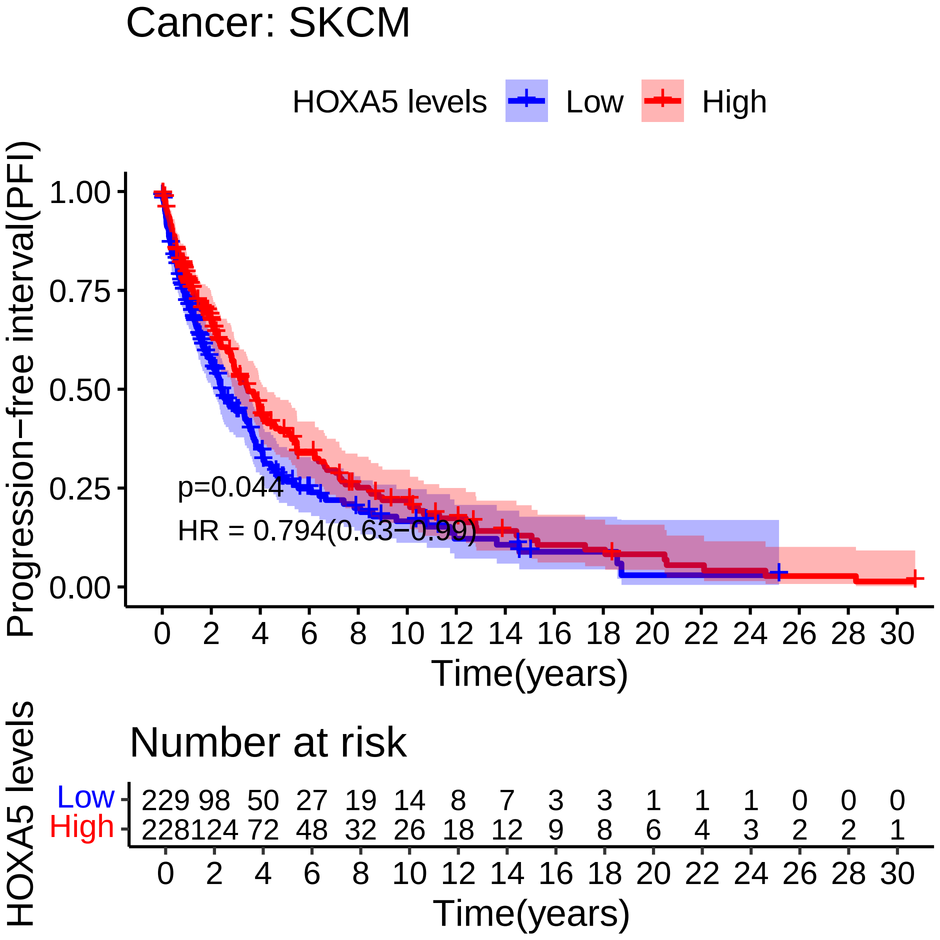

Cancer: SKCM
HOXA5 levels
Low
High
Progression−free interval(PFI)
1.00
0.75
0.50
0.25
0.00
p=0.044
HR = 0.794(0.63−0.99)
0 2 4 6 8 10 12 14 16 18 20 22 24 26 28 30
Time(years)
Number at risk
HOXA5 levels
Low 229 98 50 27 19 14 8 7 3 3 1 1 1 0 0 0
High 228 124 72 48 32 26 18 12 9 8 6 4 3 2 2 1
0 2 4 6 8 10 12 14 16 18 20 22 24 26 28 30
Time(years)

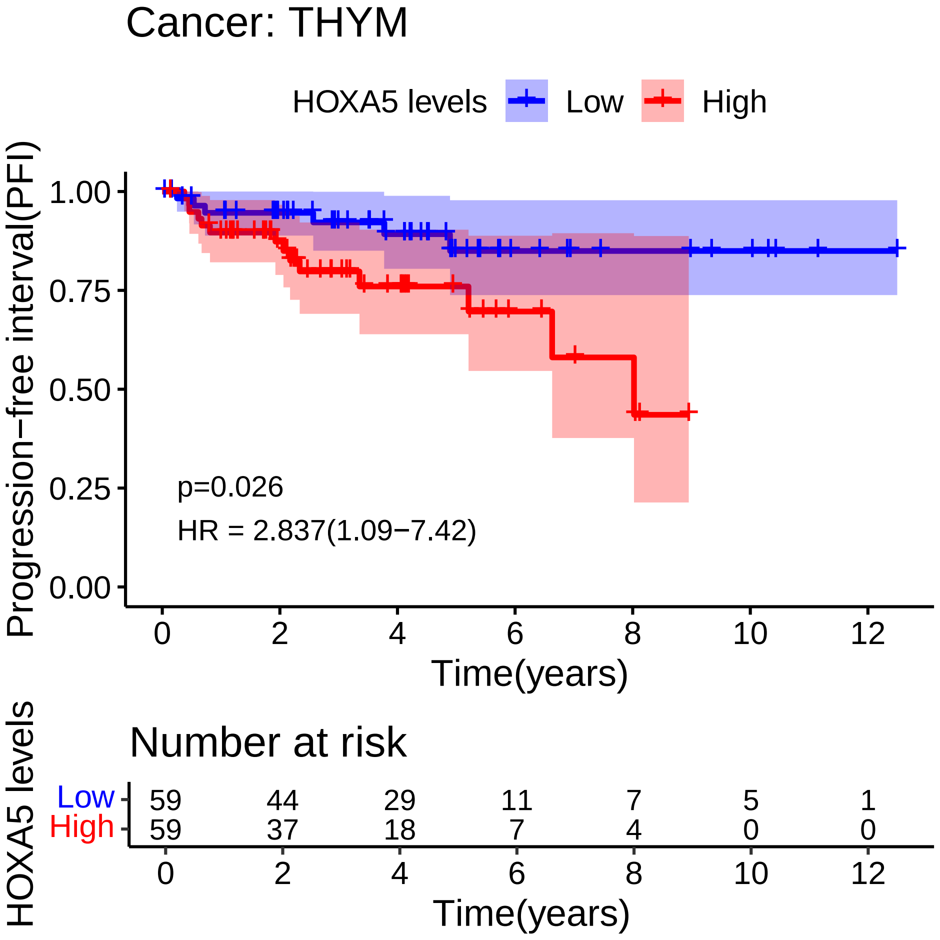
Cancer: THYM
HOXA5 levels
Low
High
Progression−free interval(PFI)
1.00
0.75
0.50
0.25
0.00
p=0.026
HR = 2.837(1.09−7.42)
0
2
4
6
8
10
12
Time(years)
Number at risk
HOXA5 levels
Low 59 44 29 11 7 5 1
High 59 37 18 7 4 0 0
Time(years)

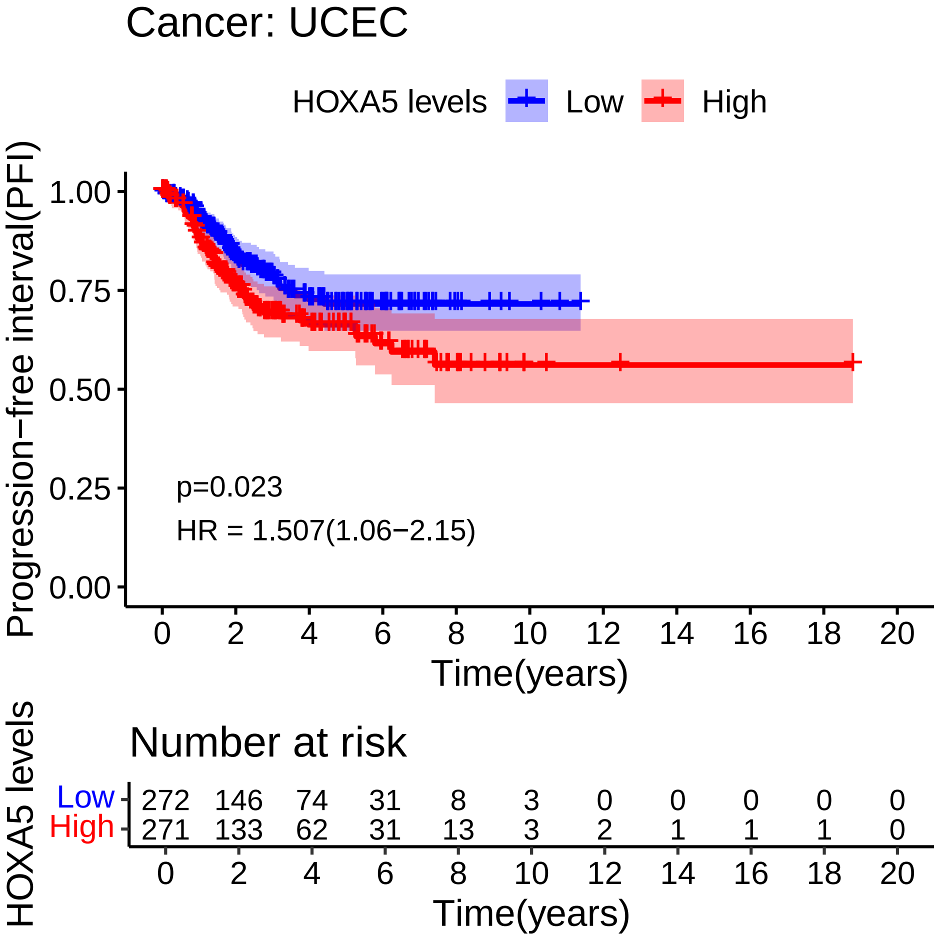

Cancer: UCEC
HOXA5 levels
Low
High
Progression−free interval(PFI)
1.00
0.75
0.50
0.25
0.00
p=0.023
HR = 1.507(1.06−2.15)
0 2 4 6 8 10 12 14 16 18 20
Time(years)
Number at risk
HOXA5 levels
Low 272 146 74 31 8 3 0 0 0 0 0
High 271 133 62 31 13 3 2 1 1 1 0
0 2 4 6 8 10 12 14 16 18 20
Time(years)

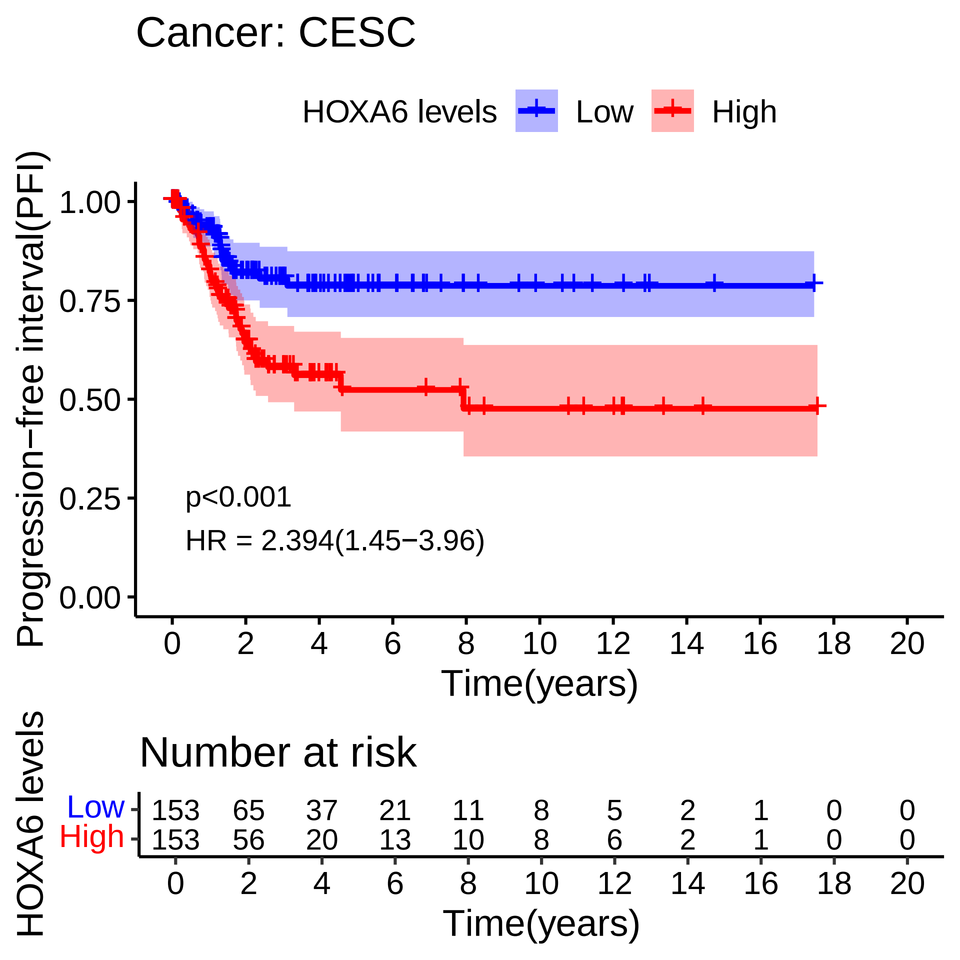

Cancer: CESC
HOXA6 levels
Low
High
Progression−free interval(PFI)
1.00
0.75
0.50
0.25
0.00
p<0.001
HR = 2.394(1.45−3.96)
0
2
4
6
8
10
12
14
16
18
20
Time(years)
Number at risk
HOXA6 levels
Low 153 65 37 21 11 8 5 2 1 0 0
High 153 56 20 13 10 8 6 2 1 0 0
Time(years)

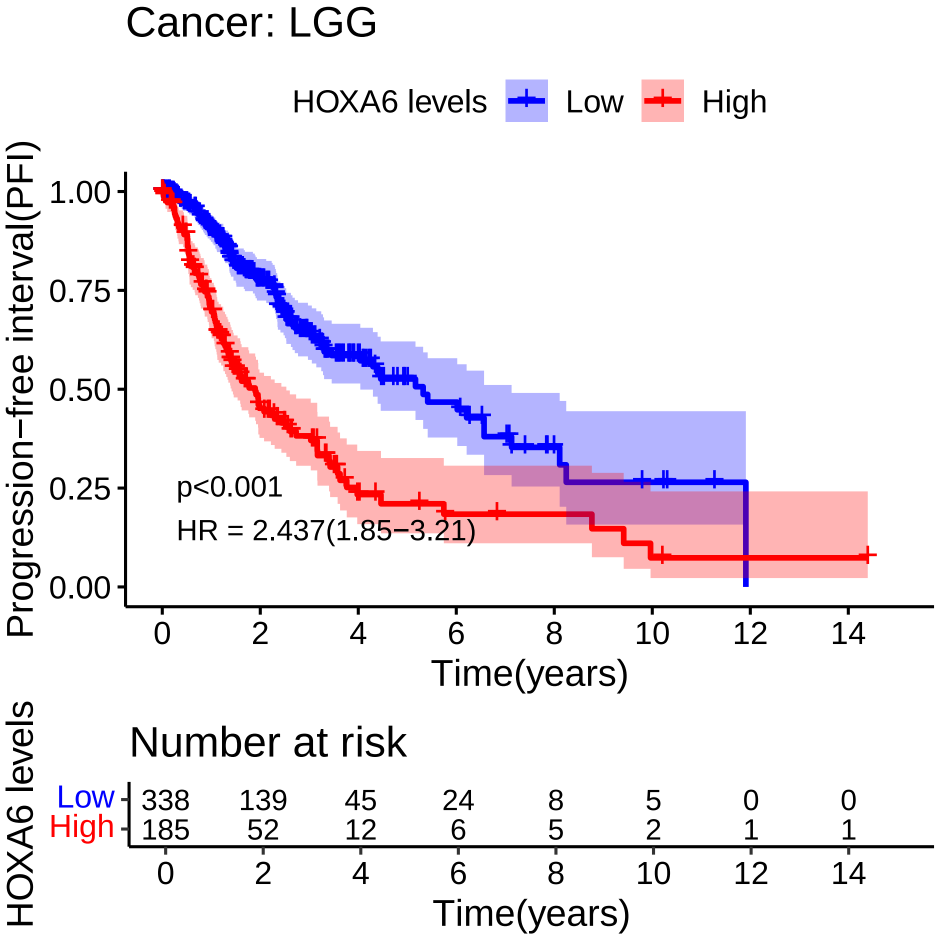
Cancer: LGG
HOXA6 levels
Low
High
Progression−free interval(PFI)
1.00
0.75
0.50
0.25
0.00
p<0.001
HR = 2.437(1.85−3.21)
0
2
4
6
8
10
12
14
Time(years)
Number at risk
HOXA6 levels
Low 338 139 45 24 8 5 0 0
High 185 52 12 6 5 2 1 1
Time(years)

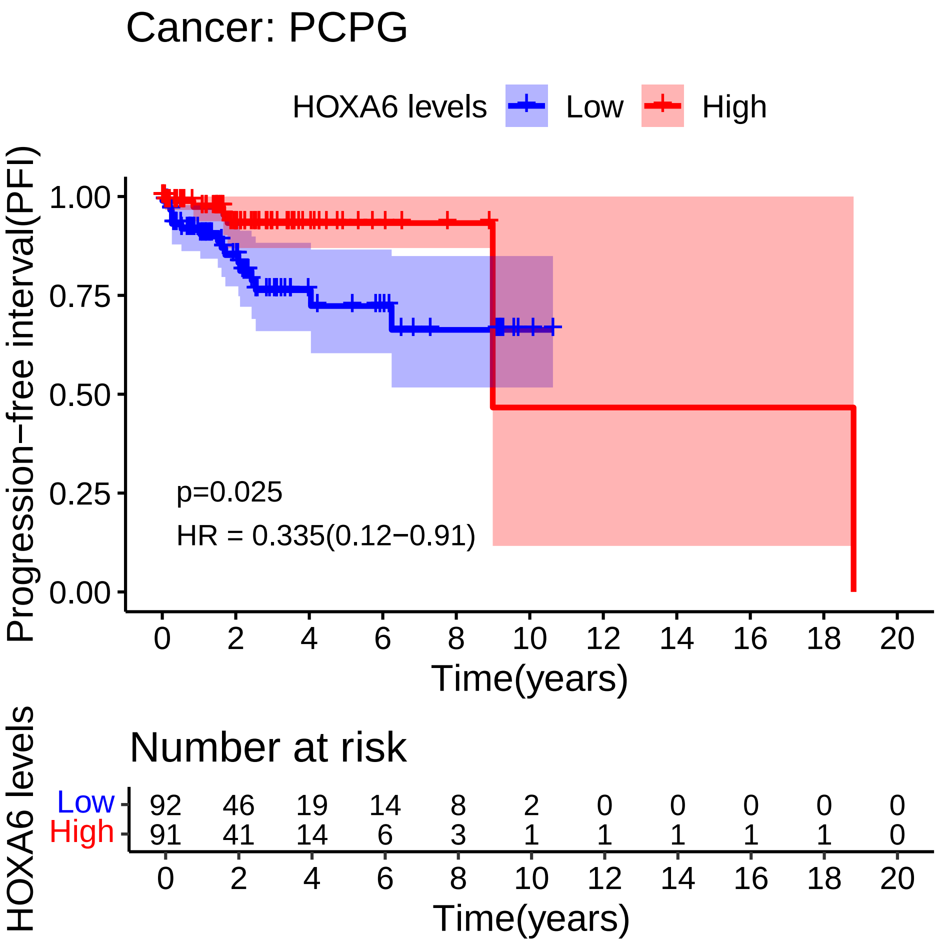
Cancer: PCPG
HOXA6 levels
Low
High
Progression−free interval(PFI)
1.00
0.75
0.50
0.25
0.00
p=0.025
HR = 0.335(0.12−0.91)
0
2
4
6
8
10
12
14
16
18
20
Time(years)
Number at risk
HOXA6 levels
Low 92 46 19 14 8 2 0 0 0 0 0
High 91 41 14 6 3 1 1 1 1 1 0
Time(years)

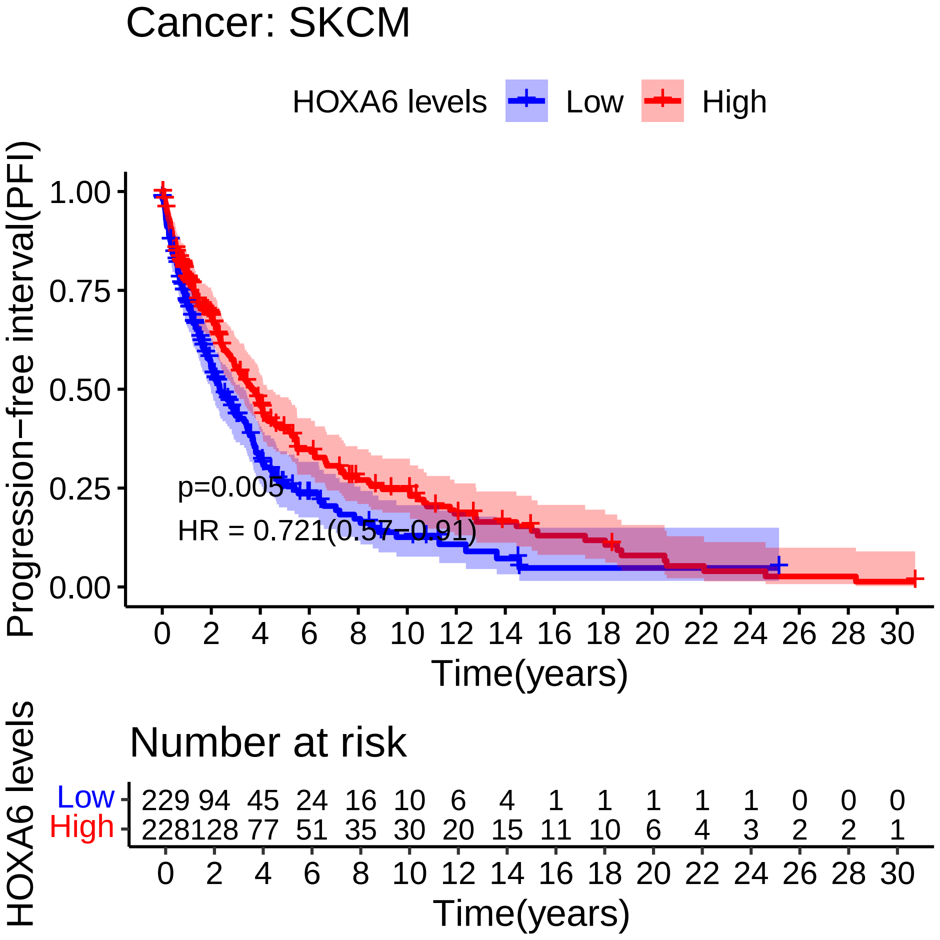
Cancer: SKCM
HOXA6 levels
Low
High
Progression−free interval(PFI)
1.00
0.75
0.50
0.25
0.00
p=0.005
HR = 0.721(0.57−0.91)
0 2 4 6 8 10 12 14 16 18 20 22 24 26 28 30
Time(years)
Number at risk
HOXA6 levels
Low 229 94 45 24 16 10 6 4 1 1 1 1 1 0 0 0
High 228 128 77 51 35 30 20 15 11 10 6 4 3 2 2 1
0 2 4 6 8 10 12 14 16 18 20 22 24 26 28 30
Time(years)

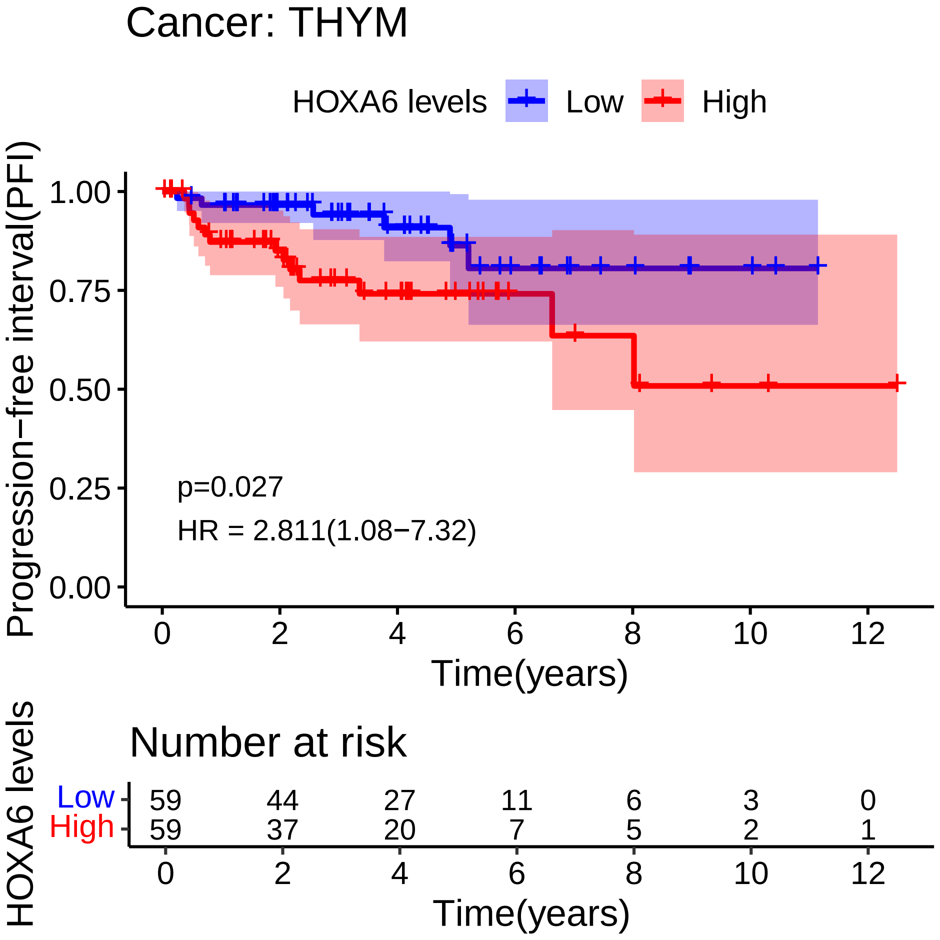

Cancer: THYM
HOXA6 levels
Low
High
Progression−free interval(PFI)
1.00
0.75
0.50
0.25
0.00
p=0.027
HR = 2.811(1.08−7.32)
0
2
4
6
8
10
12
Time(years)
Number at risk
HOXA6 levels
Low 59 44 27 11 6 3 0
High 59 37 20 7 5 2 1
Time(years)

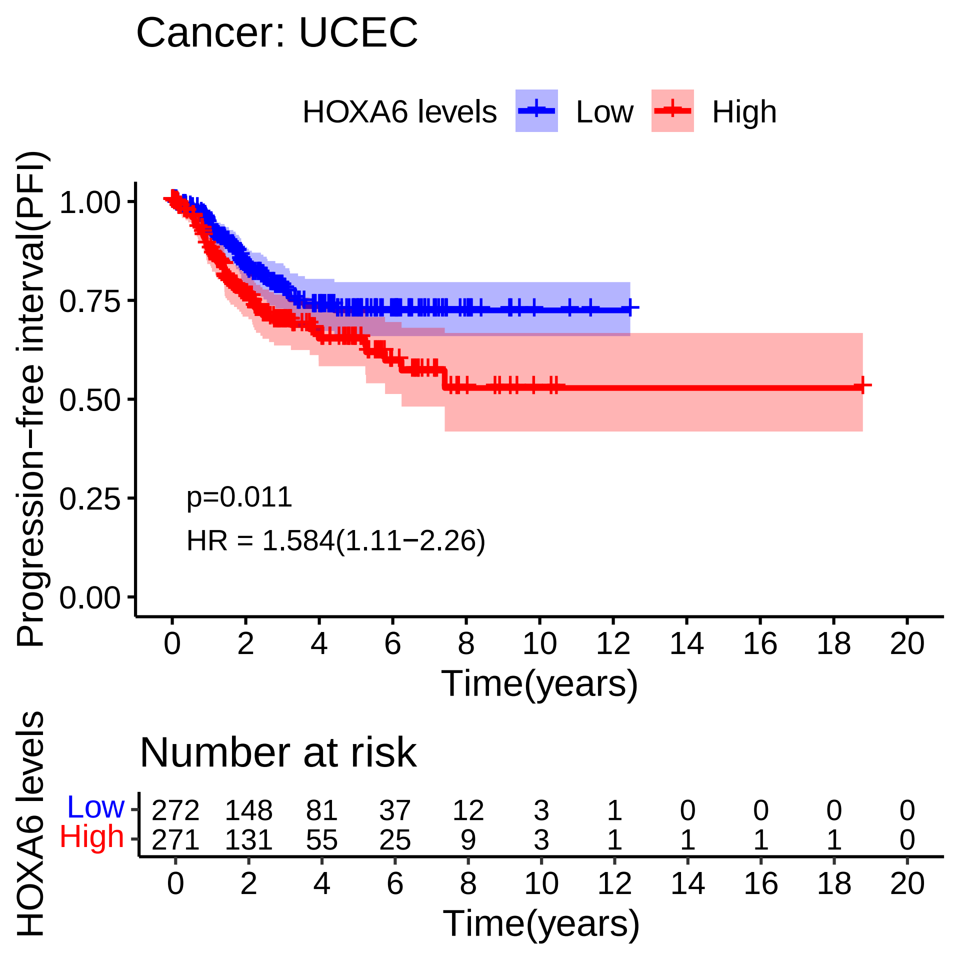
Cancer: UCEC
HOXA6 levels
Low
High
Progression−free interval(PFI)
1.00
0.75
0.50
0.25
0.00
p=0.011
HR = 1.584(1.11−2.26)
0 2 4 6 8 10 12 14 16 18 20
Time(years)
Number at risk
HOXA6 levels
Low 272 148 81 37 12 3 1 0 0 0 0
High 271 131 55 25 9 3 1 1 1 1 0
0 2 4 6 8 10 12 14 16 18 20
Time(years)

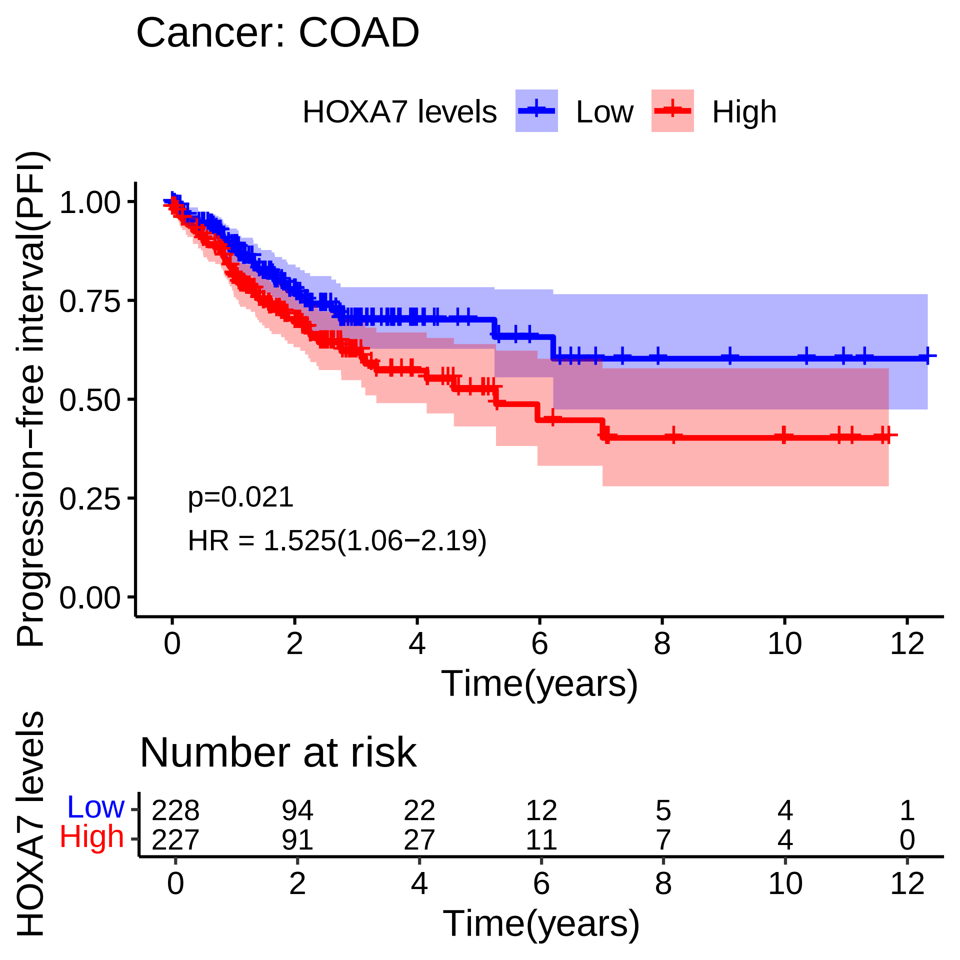
Cancer: COAD
HOXA7 levels
Low
High
Progression−free interval(PFI)
1.00
0.75
0.50
0.25
0.00
p=0.021
HR = 1.525(1.06−2.19)
0
2
4
6
8
10
12
Time(years)
Number at risk
HOXA7 levels
Low 228 94 22 12 5 4 1
High 227 91 27 11 7 4 0
Time(years)

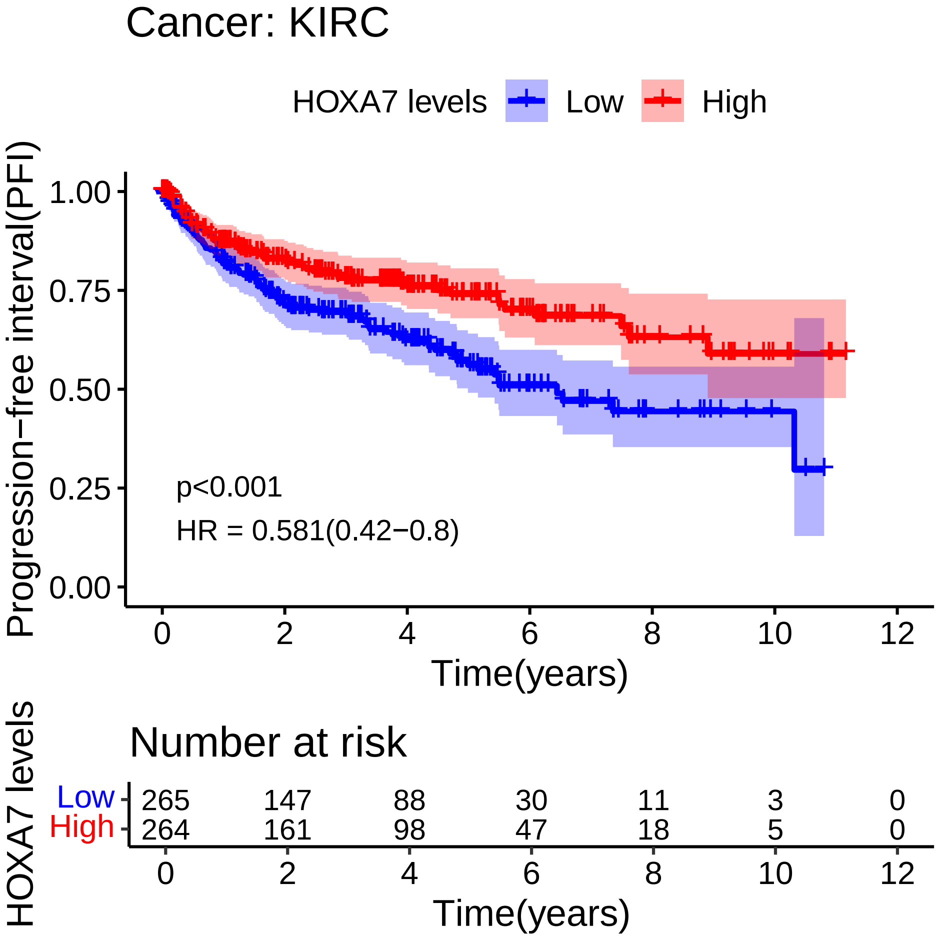
Cancer: KIRC
HOXA7 levels
Low
High
Progression−free interval(PFI)
1.00
0.75
0.50
0.25
0.00
p<0.001
HR = 0.581(0.42−0.8)
0
2
4
6
8
10
12
Time(years)
Number at risk
HOXA7 levels
Low
High
265 147 88 30 11 3 0
264 161 98 47 18 5 0
Time(years)

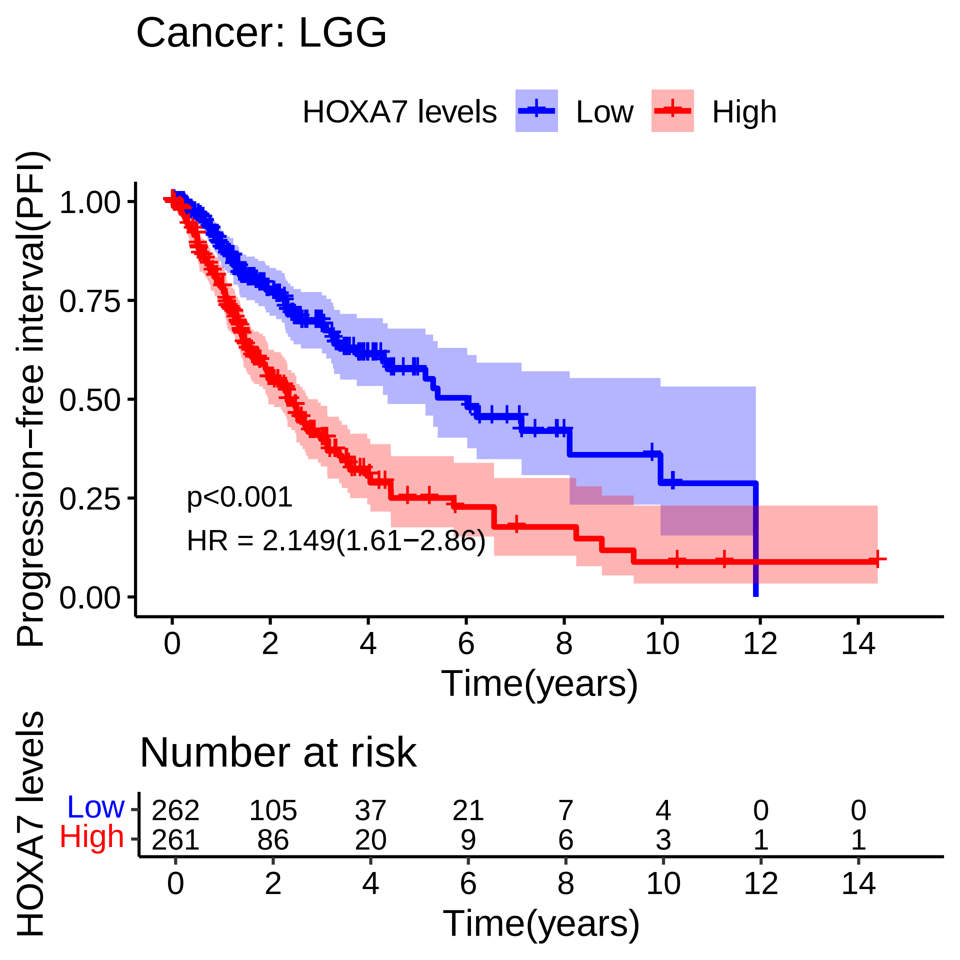
Cancer: LGG
HOXA7 levels
Low
High
Progression−free interval(PFI)
1.00
0.75
0.50
0.25
0.00
p<0.001
HR = 2.149(1.61−2.86)
0
2
4
6
8
10
12
14
Time(years)
Number at risk
HOXA7 levels
Low 262 105 37 21 7 4 0 0
High 261 86 20 9 6 3 1 1
Time(years)

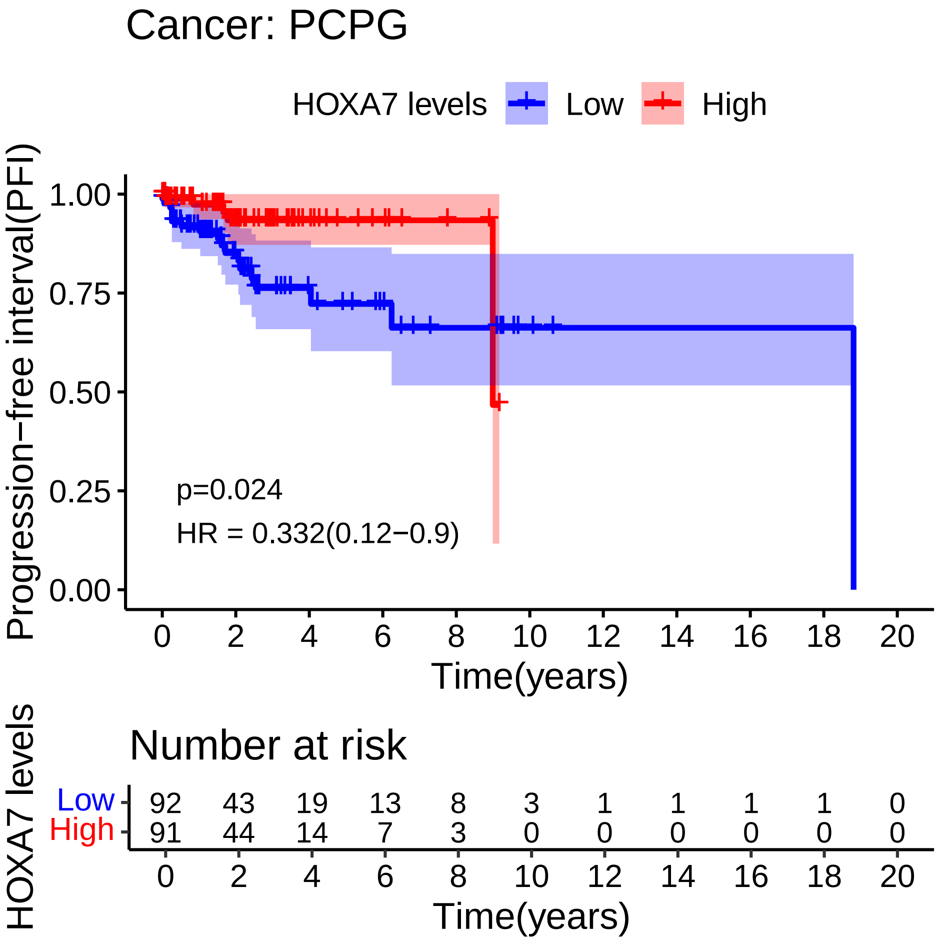

Cancer: PCPG
HOXA7 levels
Low
High
Progression−free interval(PFI)
1.00
0.75
0.50
0.25
0.00
p=0.024
HR = 0.332(0.12−0.9)
0 2 4 6 8 10 12 14 16 18 20
Time(years)
Number at risk
HOXA7 levels
Low 92 43 19 13 8 3 1 1 1 1 0
High 91 44 14 7 3 0 0 0 0 0 0
0 2 4 6 8 10 12 14 16 18 20
Time(years)

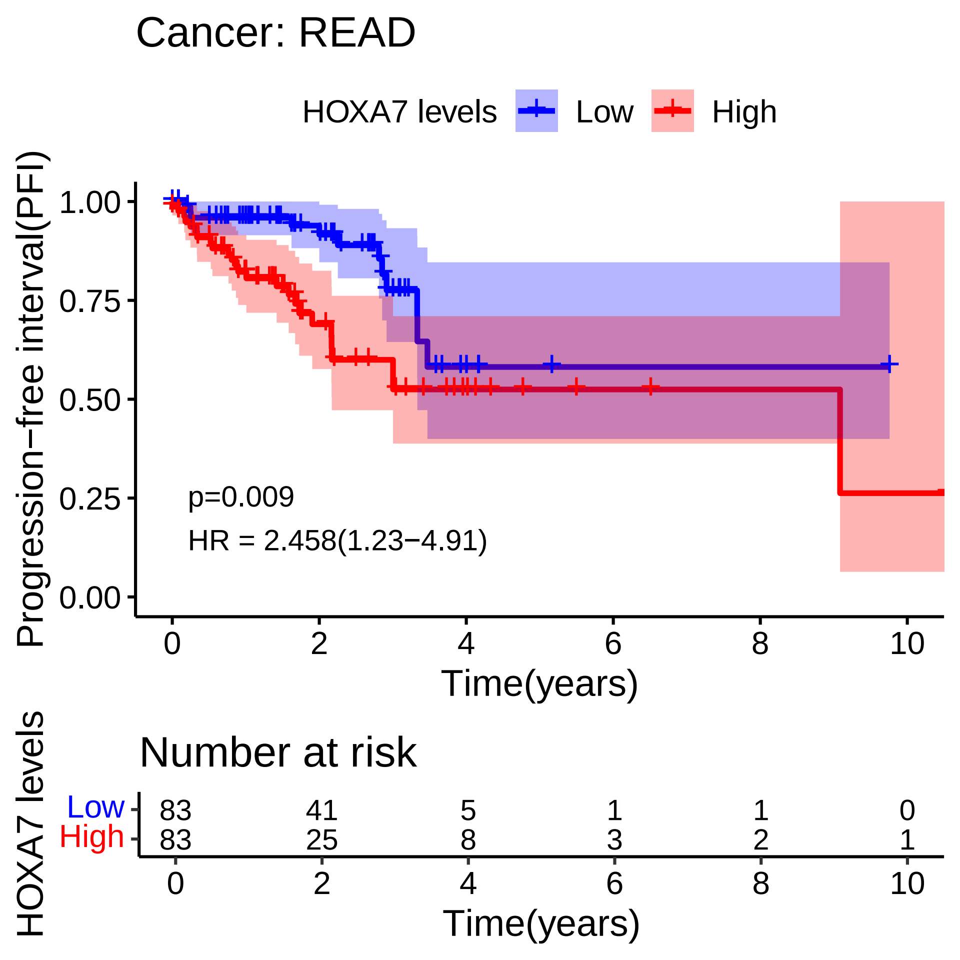
Cancer: READ
HOXA7 levels
Low
High
Progression−free interval(PFI)
1.00
0.75
0.50
0.25
0.00
p=0.009
HR = 2.458(1.23−4.91)
0
2
4
6
8
10
Time(years)
Number at risk
HOXA7 levels
Low
High
83 41 5 1 1 0
83 25 8 3 2 1
0
2
4
6
8
10
Time(years)

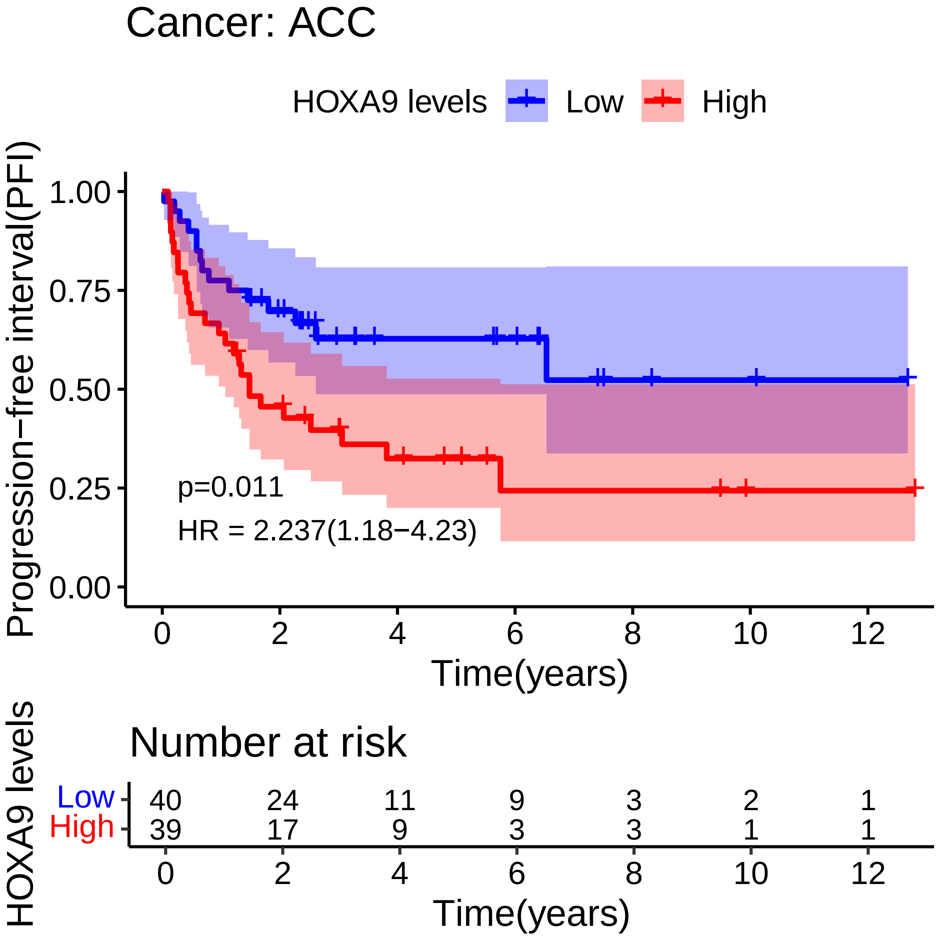
Cancer: ACC
HOXA9 levels
Low
High
Progression−free interval(PFI)
1.00
0.75
0.50
0.25
0.00
p=0.011
HR = 2.237(1.18−4.23)
0
2
4
6
8
10
12
Time(years)
Number at risk
HOXA9 levels
Low 40 24 11 9 3 2 1
High 39 17 9 3 3 1 1
0
2
4
6
8
10
12
Time(years)

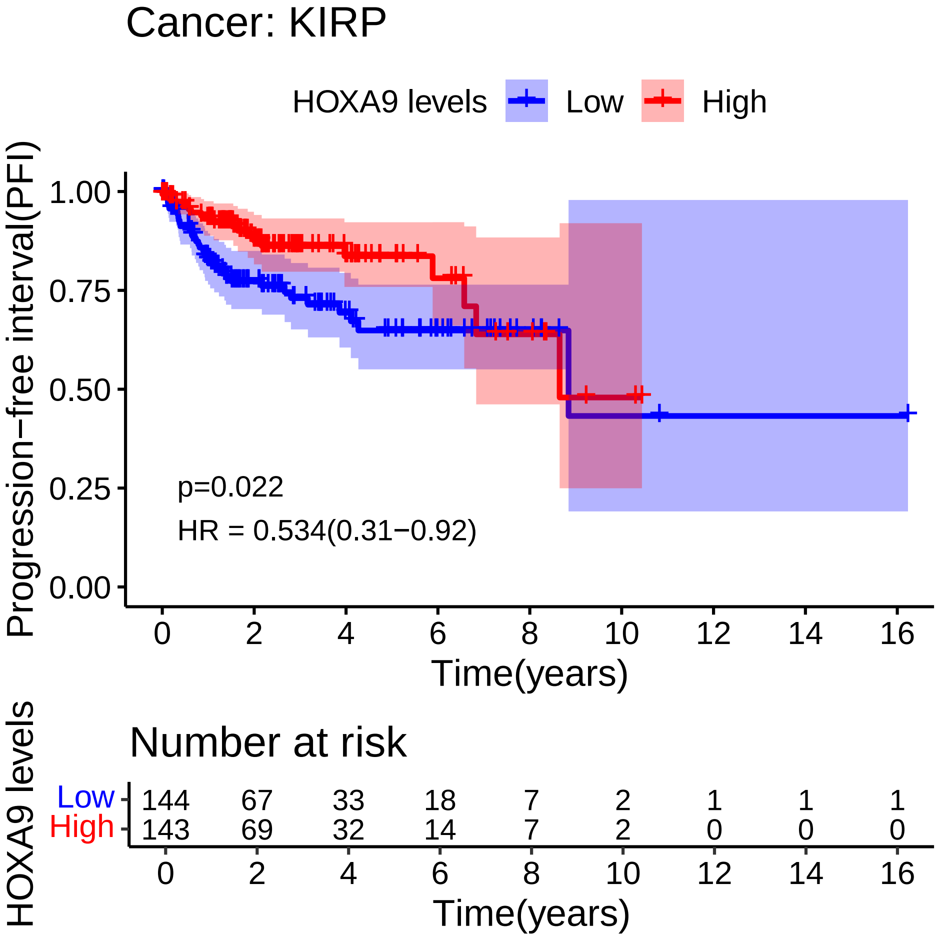

Cancer: KIRP
HOXA9 levels
Low
High
Progression−free interval(PFI)
1.00
0.75
0.50
0.25
0.00
p=0.022
HR = 0.534(0.31−0.92)
0
2
4
6
8
10
12
14
16
Time(years)
Number at risk
HOXA9 levels
Low 144 67 33 18 7 2 1 1 1
High 143 69 32 14 7 2 0 0 0
Time(years)

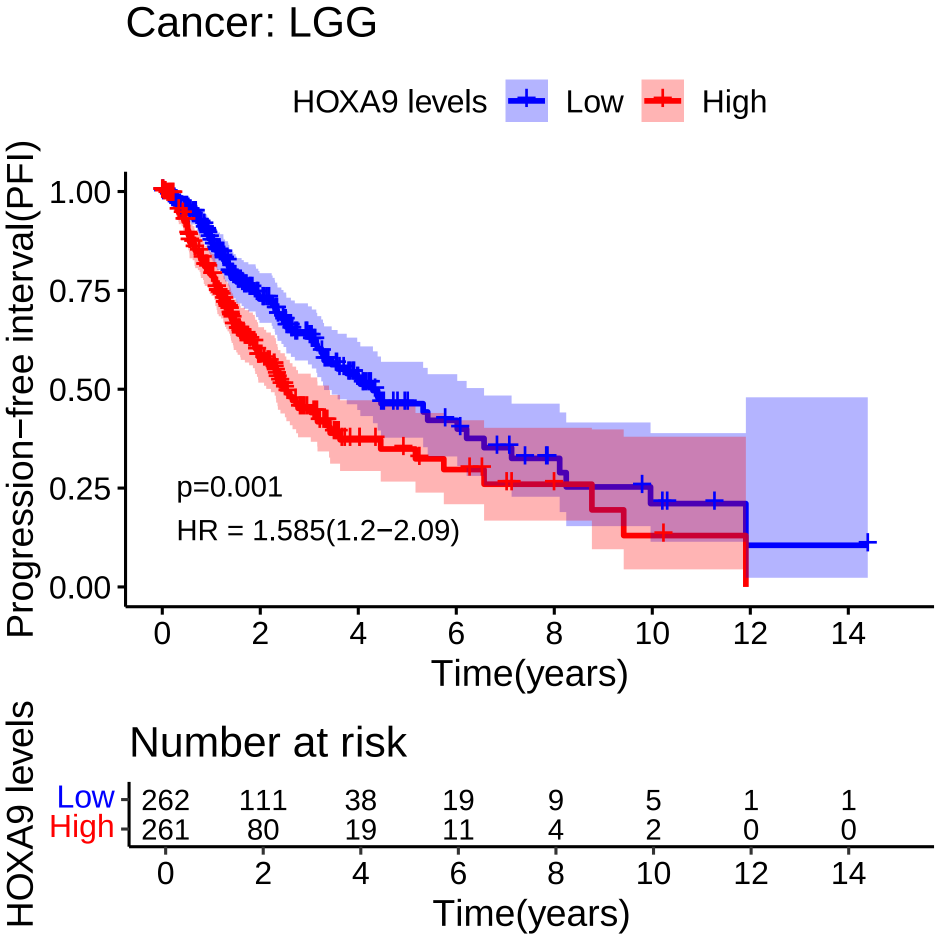

Cancer: LGG
HOXA9 levels
Low
High
Progression−free interval(PFI)
1.00
0.75
0.50
0.25
0.00
p=0.001
HR = 1.585(1.2−2.09)
0
2
4
6
8
10
12
14
Time(years)
Number at risk
HOXA9 levels
Low 262 111 38 19 9 5 1 1
High 261 80 19 11 4 2 0 0
Time(years)

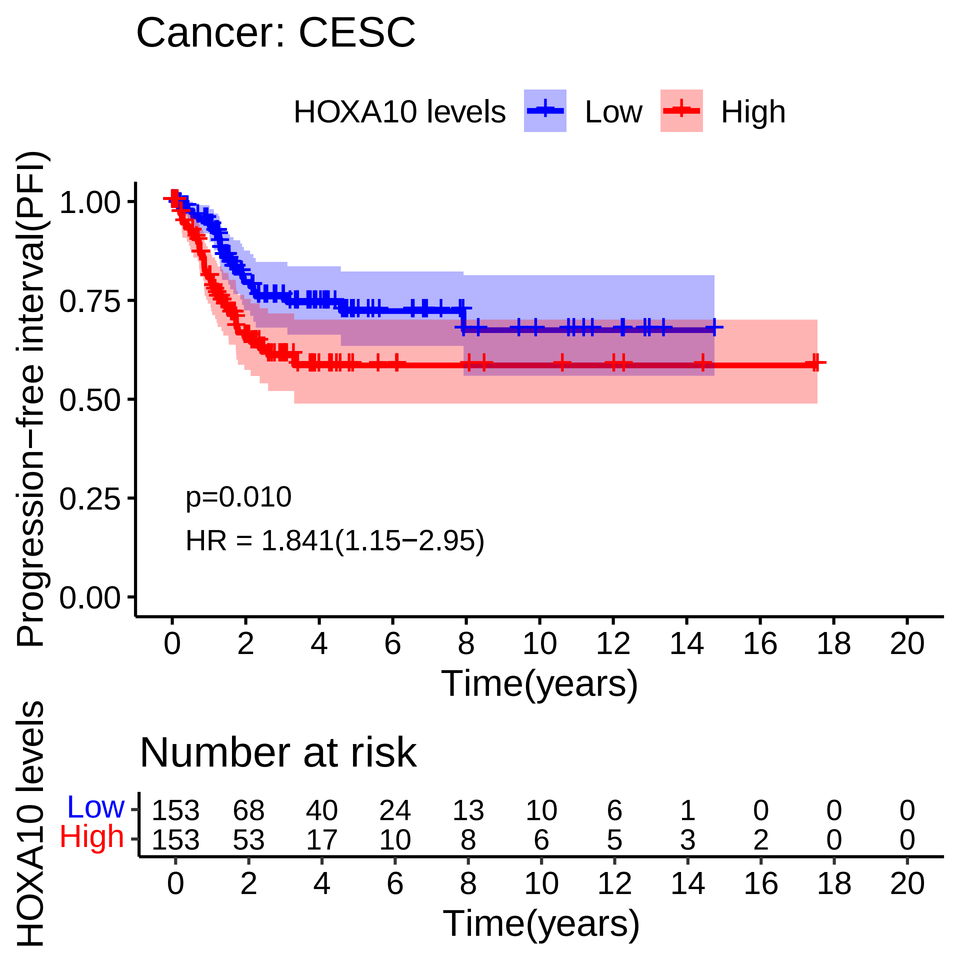

Cancer: CESC
HOXA10 levels
Low
High
Progression−free interval(PFI)
1.00
0.75
0.50
0.25
0.00
p=0.010
HR = 1.841(1.15−2.95)
0 2 4 6 8 10 12 14 16 18 20
Time(years)
Number at risk
HOXA10 levels
Low 153 68 40 24 13 10 6 1 0 0 0
High 153 53 17 10 8 6 5 3 2 0 0
0 2 4 6 8 10 12 14 16 18 20
Time(years)

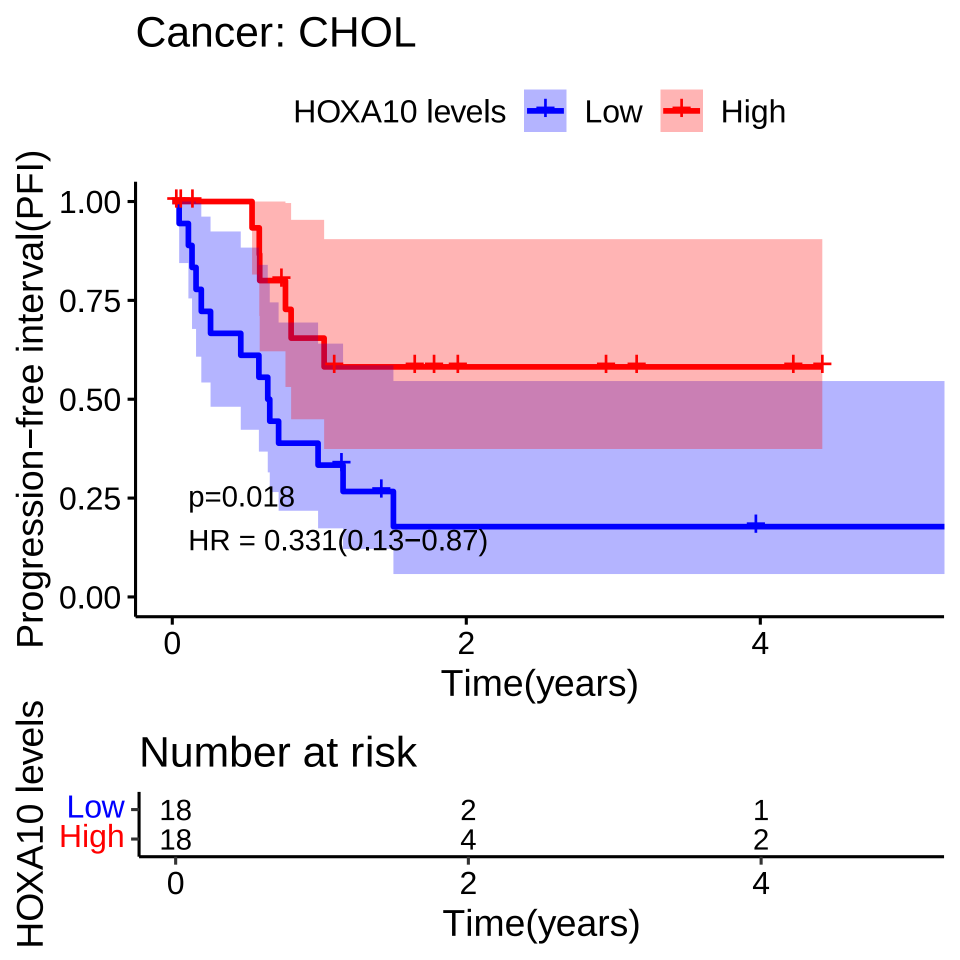
Cancer: CHOL
HOXA10 levels
Low
High
Progression−free interval(PFI)
1.00
0.75
0.50
0.25
0.00
p=0.018
HR = 0.331(0.13−0.87)
0
2
4
Time(years)
Number at risk
HOXA10 levels
Low
High
18
2
1
18
4
2
0
2
4
Time(years)

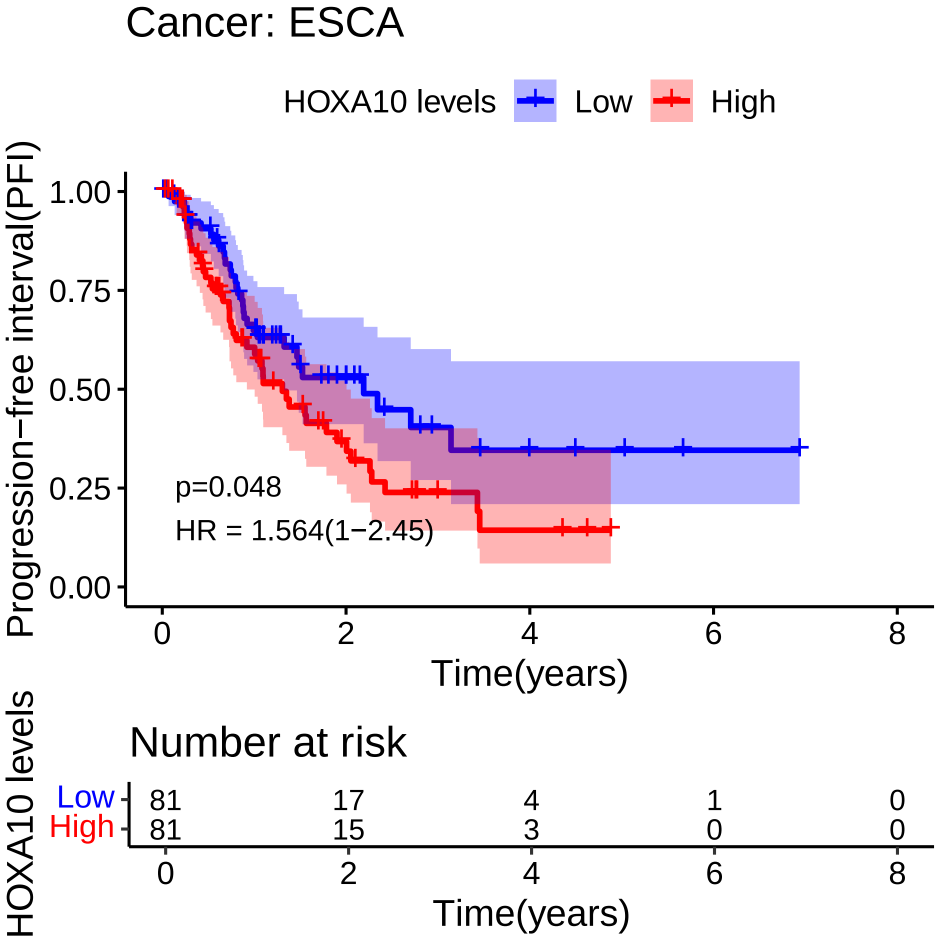
Cancer: ESCA
HOXA10 levels
Low
High
Progression−free interval(PFI)
1.00
0.75
0.50
0.25
0.00
p=0.048
HR = 1.564(1−2.45)
0
2
4
6
8
Time(years)
Number at risk
HOXA10 levels
Low
High
81
17
4
1
0
81
15
3
0
0
Time(years)

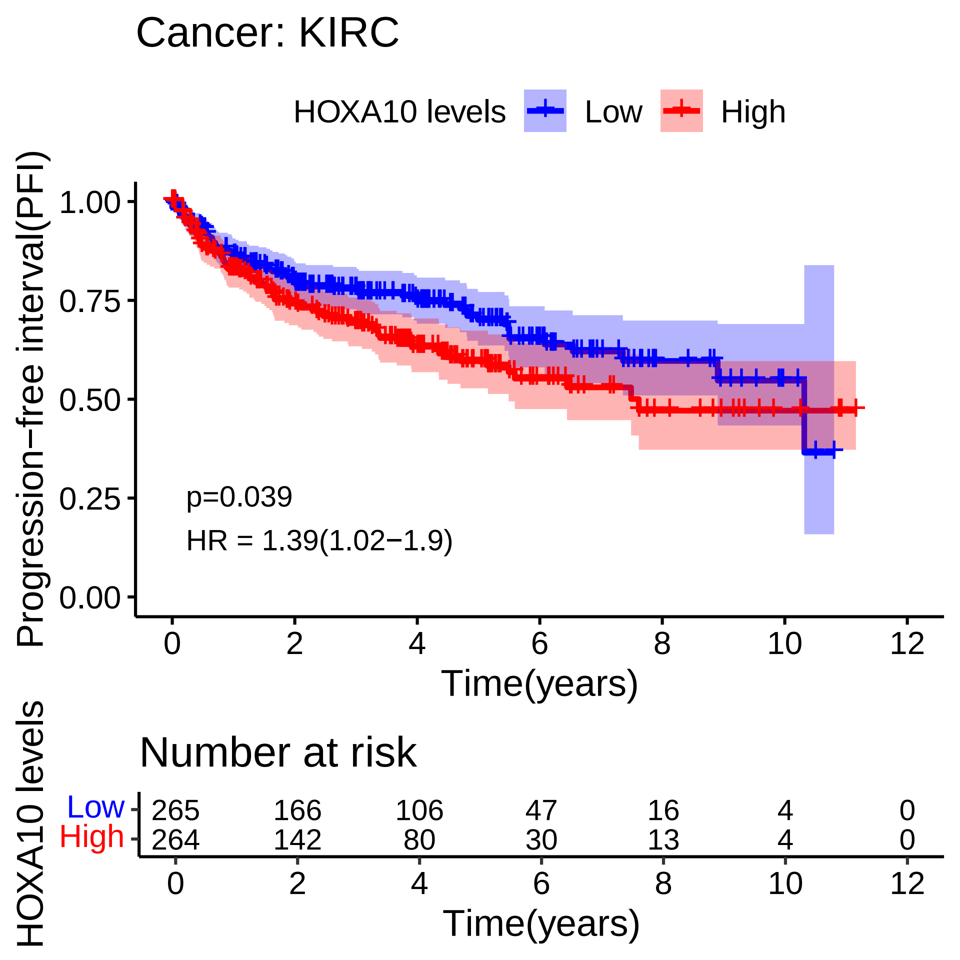
Cancer: KIRC
HOXA10 levels
Low
High
Progression−free interval(PFI)
1.00
0.75
0.50
0.25
0.00
p=0.039
HR = 1.39(1.02−1.9)
0
2
4
6
8
10
12
Time(years)
Number at risk
HOXA10 levels
Low 265 166 106 47 16 4 0
High 264 142 80 30 13 4 0
0
2
4
6
8
10
12
Time(years)

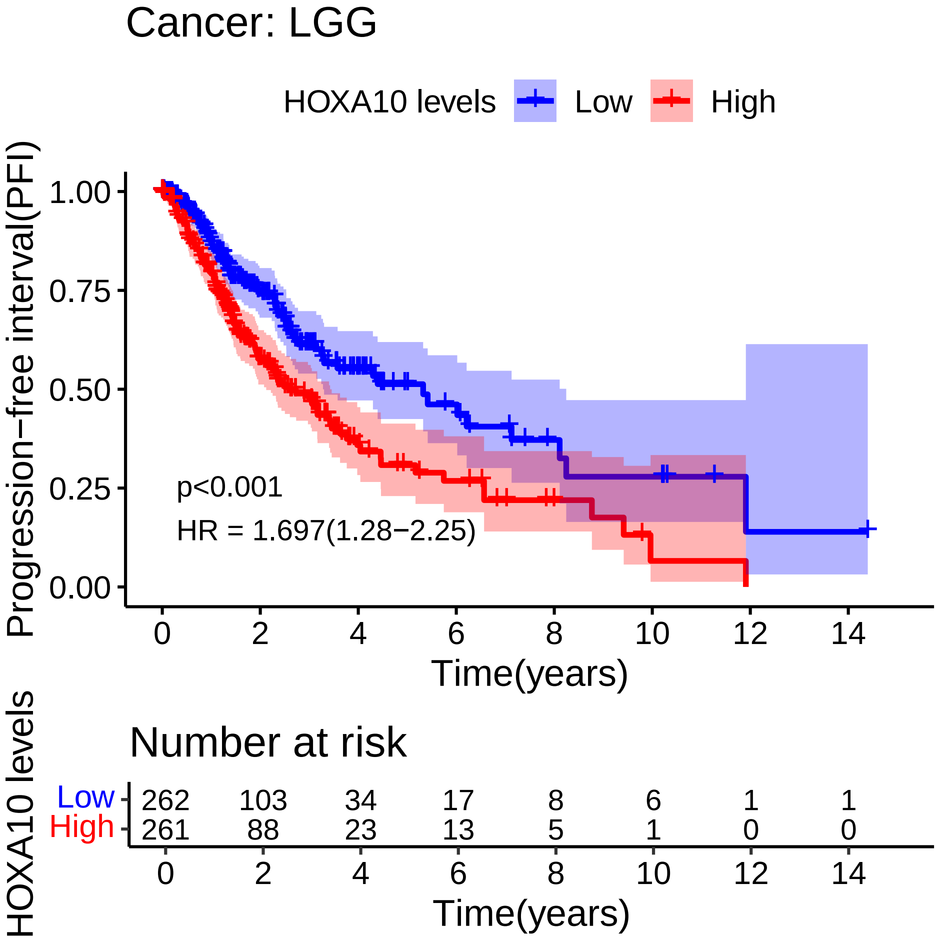

Cancer: LGG
HOXA10 levels
Low
High
Progression−free interval(PFI)
1.00
0.75
0.50
0.25
0.00
p<0.001
HR = 1.697(1.28−2.25)
0
2
4
6
8
10
12
14
Time(years)
Number at risk
HOXA10 levels
Low
High
262 103 34 17 8 6 1 1
261 88 23 13 5 1 0 0
Time(years)

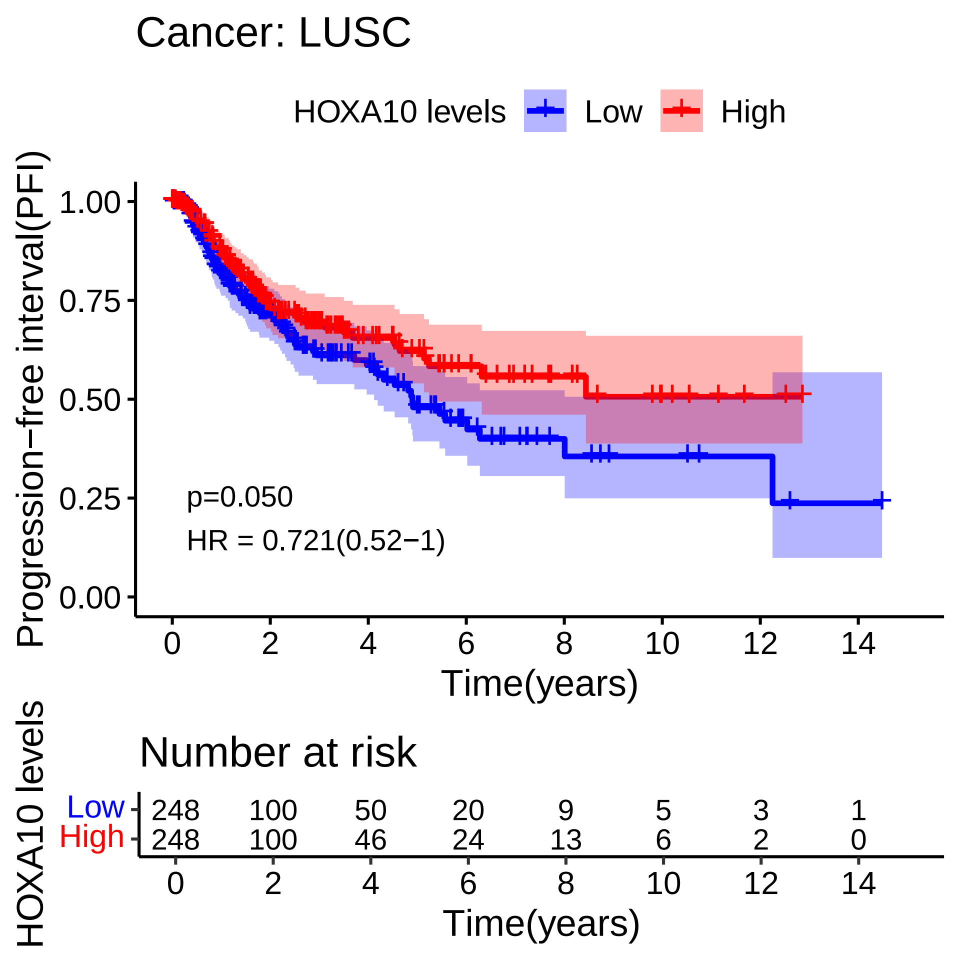
Cancer: LUSC
HOXA10 levels
Low
High
Progression−free interval(PFI)
1.00
0.75
0.50
0.25
0.00
p=0.050
HR = 0.721(0.52−1)
0
2
4
6
8
10
12
14
Time(years)
Number at risk
HOXA10 levels
Low 248 100 50 20 9 5 3 1
High 248 100 46 24 13 6 2 0
Time(years)

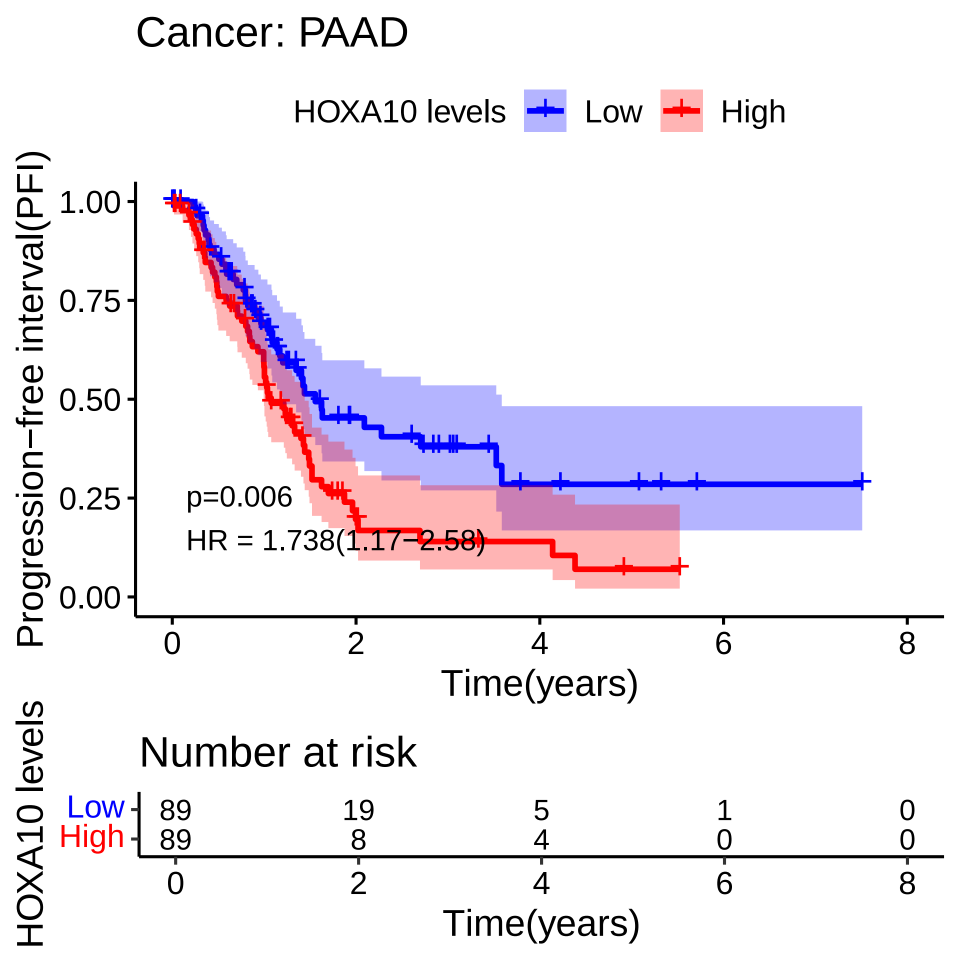

Cancer: PAAD
HOXA10 levels
Low
High
Progression−free interval(PFI)
1.00
0.75
0.50
0.25
0.00
p=0.006
HR = 1.738(1.17−2.58)
0
2
4
6
8
Time(years)
Number at risk
HOXA10 levels
Low 89 19 5 1 0
High 89 8 4 0 0
Time(years)

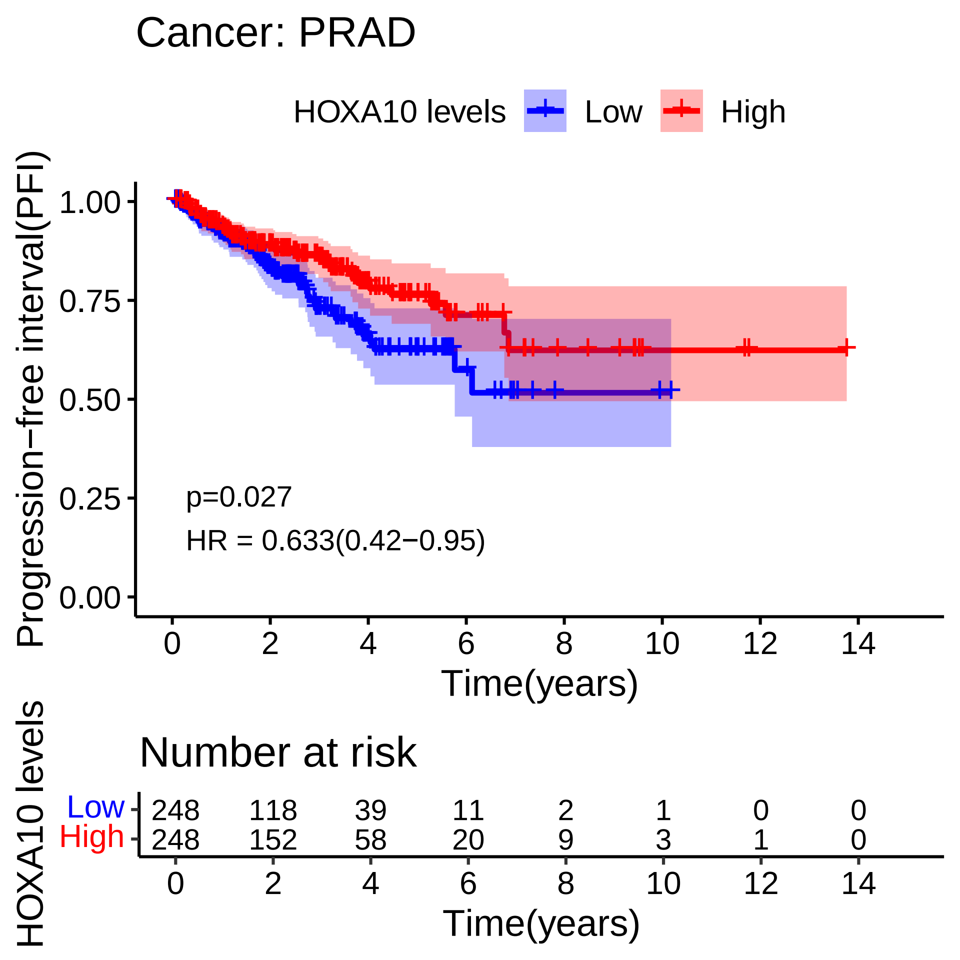
Cancer: PRAD
HOXA10 levels
Low
High
Progression−free interval(PFI)
1.00
0.75
0.50
0.25
0.00
p=0.027
HR = 0.633(0.42−0.95)
0
2
4
6
8
10
12
14
Time(years)
Number at risk
HOXA10 levels
Low 248 118 39 11 2 1 0 0
High 248 152 58 20 9 3 1 0
Time(years)

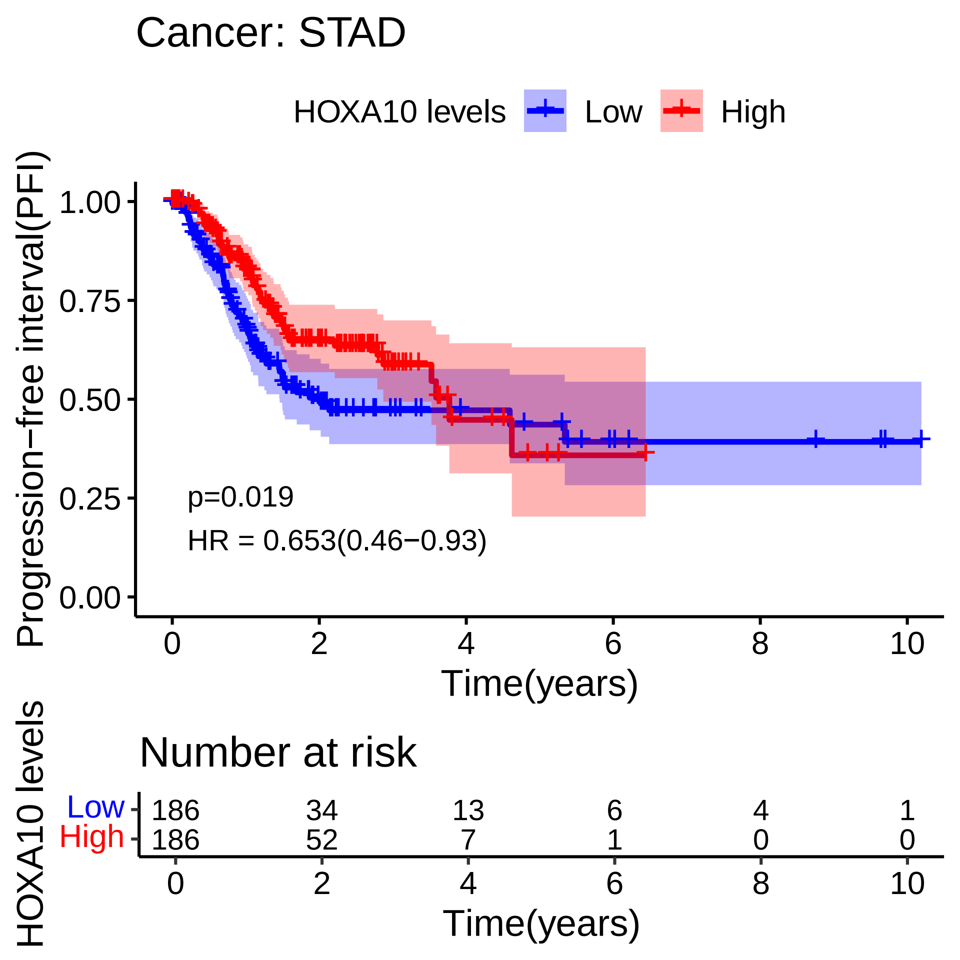

Cancer: STAD
HOXA10 levels
Low
High
Progression−free interval(PFI)
1.00
0.75
0.50
0.25
0.00
p=0.019
HR = 0.653(0.46−0.93)
0
2
4
6
8
10
Time(years)
Number at risk
HOXA10 levels
Low 186 34 13 6 4 1
High 186 52 7 1 0 0
Time(years)

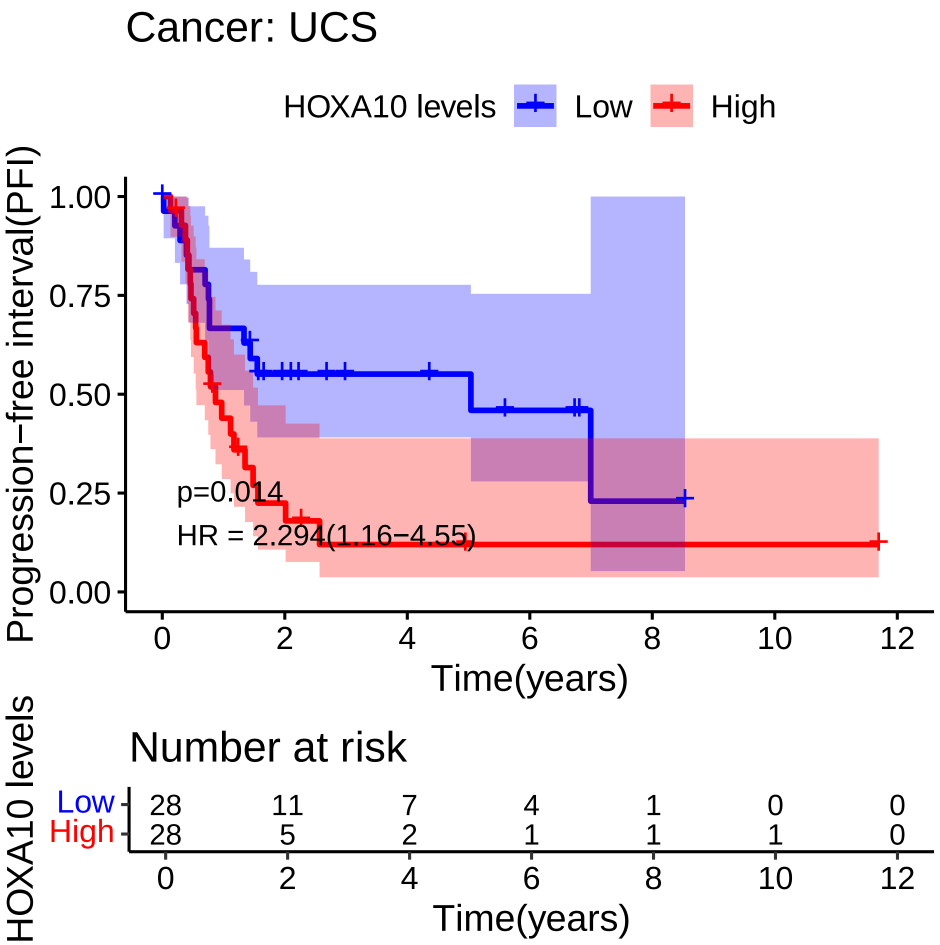

Cancer: UCS
HOXA10 levels
Low
High
Progression−free interval(PFI)
1.00
0.75
0.50
0.25
0.00
p=0.014
HR = 2.294(1.16−4.55)
0
2
4
6
8
10
12
Time(years)
Number at risk
HOXA10 levels
Low 28 11 7 4 1 0 0
High 28 5 2 1 1 1 0
Time(years)

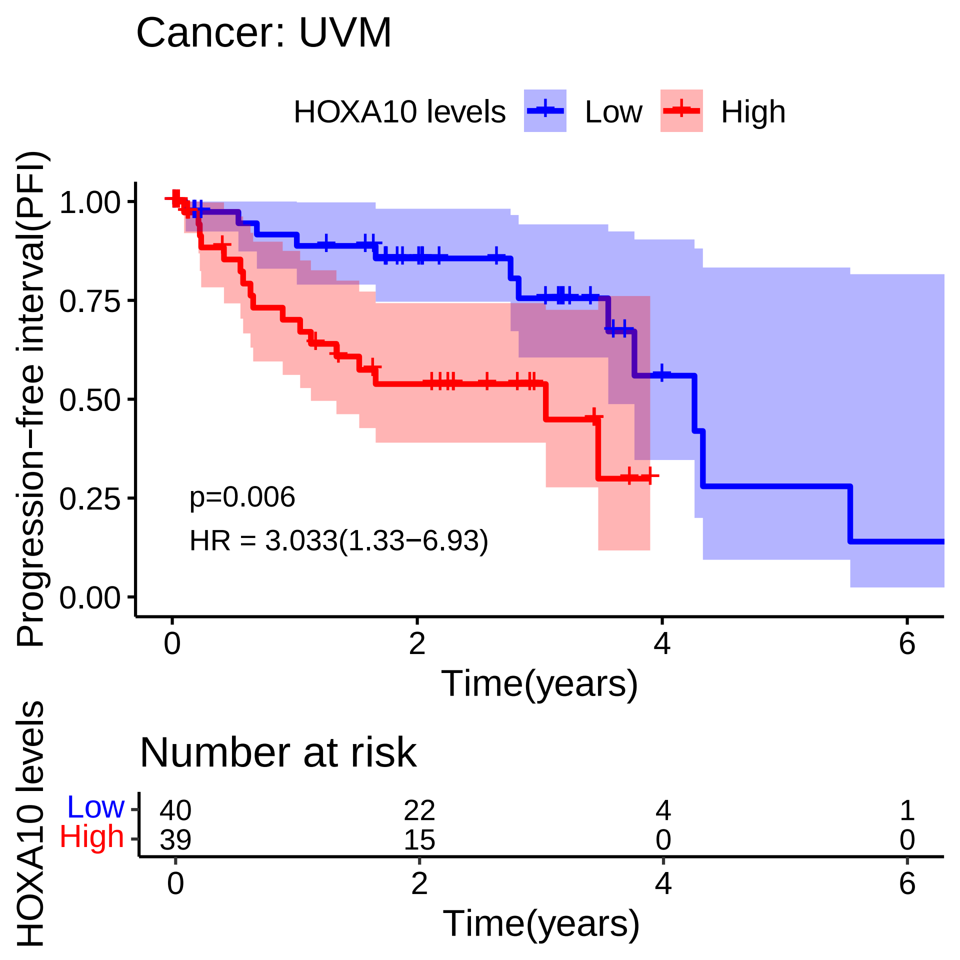

Cancer: UVM
HOXA10 levels
Low
High
Progression−free interval(PFI)
1.00
0.75
0.50
0.25
0.00
p=0.006
HR = 3.033(1.33−6.93)
0
2
4
6
Time(years)
Number at risk
HOXA10 levels
Low 40 22 4 1
High 39 15 0 0
0
2
4
6
Time(years)

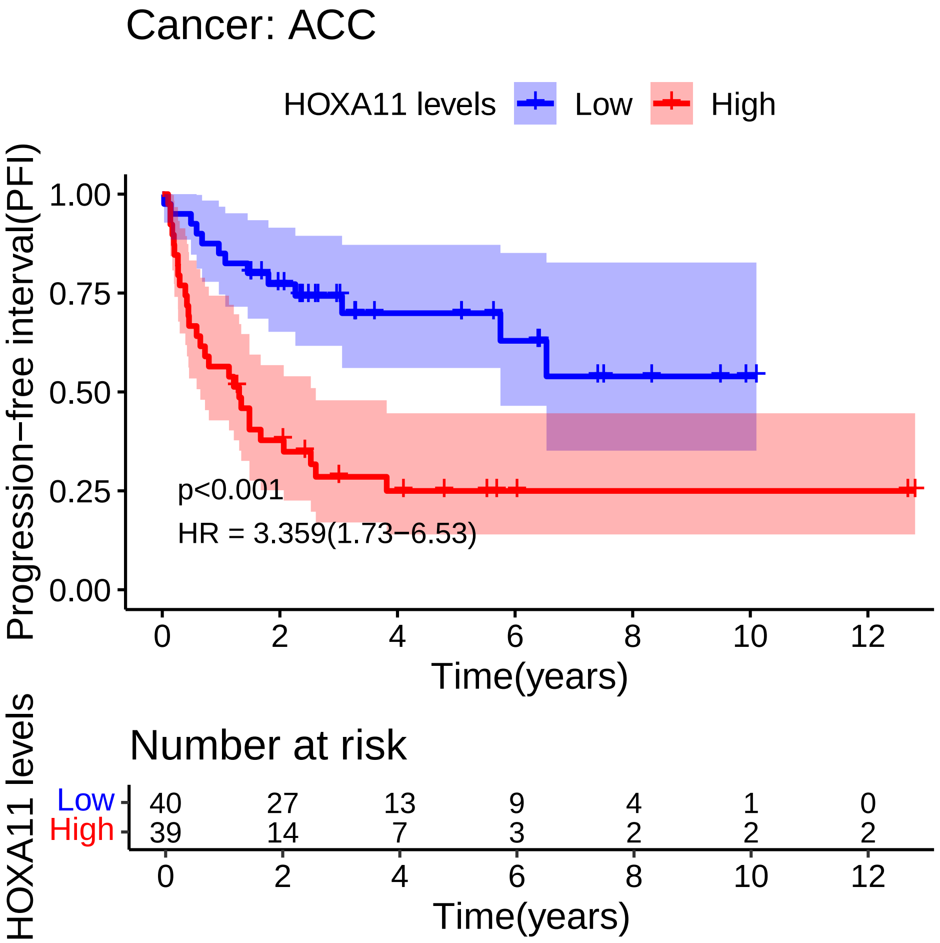
Cancer: ACC
HOXA11 levels
Low
High
Progression−free interval(PFI)
1.00
0.75
0.50
0.25
0.00
p<0.001
HR = 3.359(1.73−6.53)
0
2
4
6
8
10
12
Time(years)
Number at risk
HOXA11 levels
Low 40 27 13 9 4 1 0
High 39 14 7 3 2 2 2
0
2
4
6
8
10
12
Time(years)

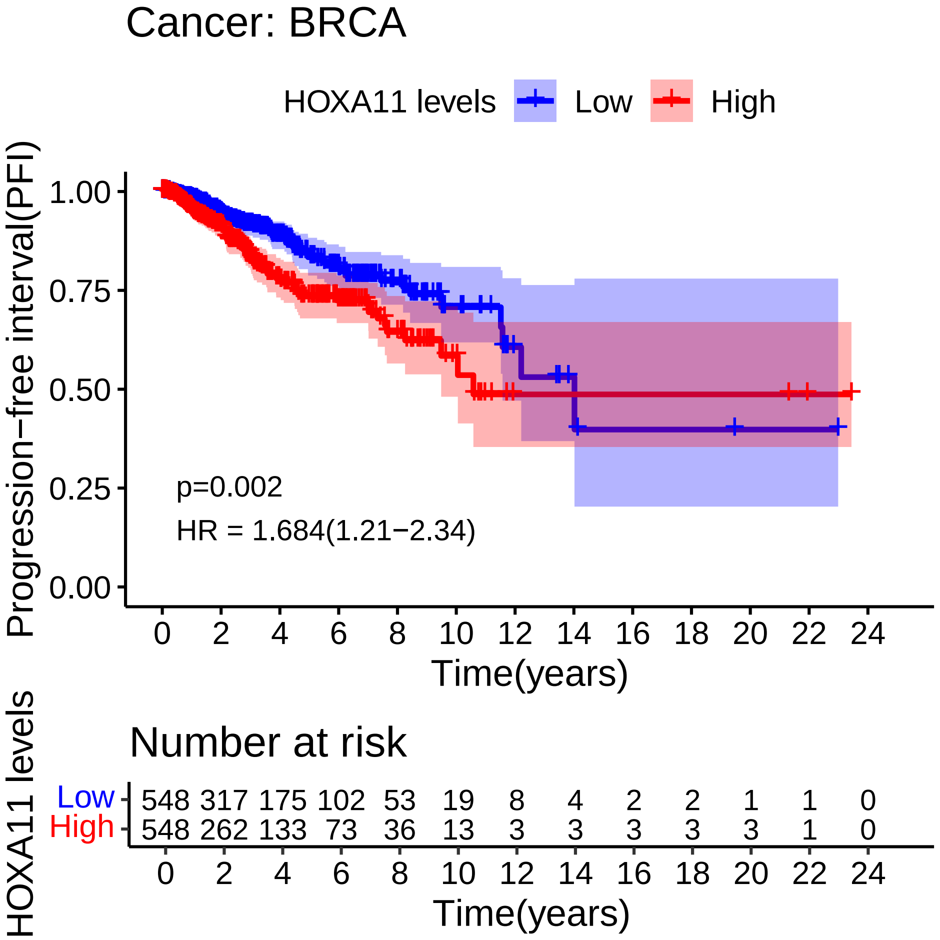

Cancer: BRCA
HOXA11 levels
Low
High
Progression−free interval(PFI)
1.00
0.75
0.50
0.25
0.00
p=0.002
HR = 1.684(1.21−2.34)
0 2 4 6 8 10 12 14 16 18 20 22 24
Time(years)
Number at risk
HOXA11 levels
Low 548 317 175 102 53 19 8 4 2 2 1 1 0
High 548 262 133 73 36 13 3 3 3 3 3 1 0
0 2 4 6 8 10 12 14 16 18 20 22 24
Time(years)

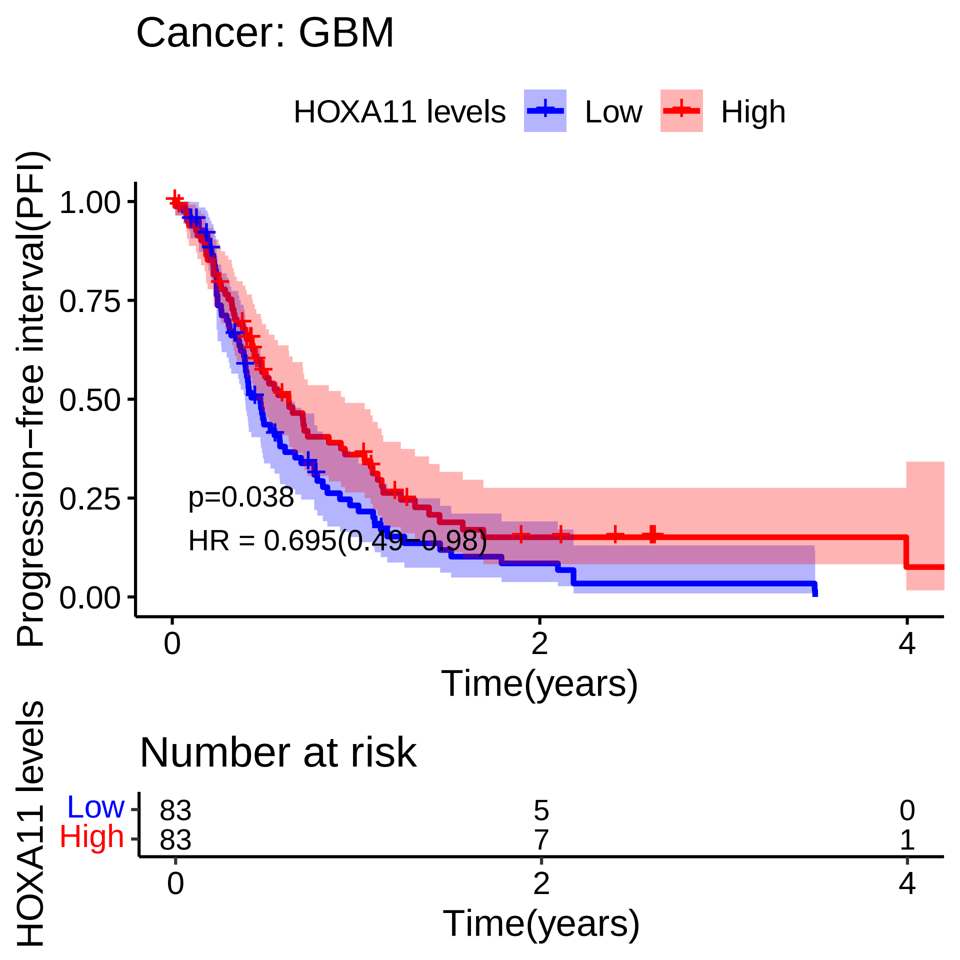

Cancer: GBM
HOXA11 levels
Low
High
Progression−free interval(PFI)
1.00
0.75
0.50
0.25
0.00
p=0.038
HR = 0.695(0.49−0.98)
0
2
4
Time(years)
Number at risk
HOXA11 levels
Low
High
83
5
0
83
7
1
0
2
4
Time(years)

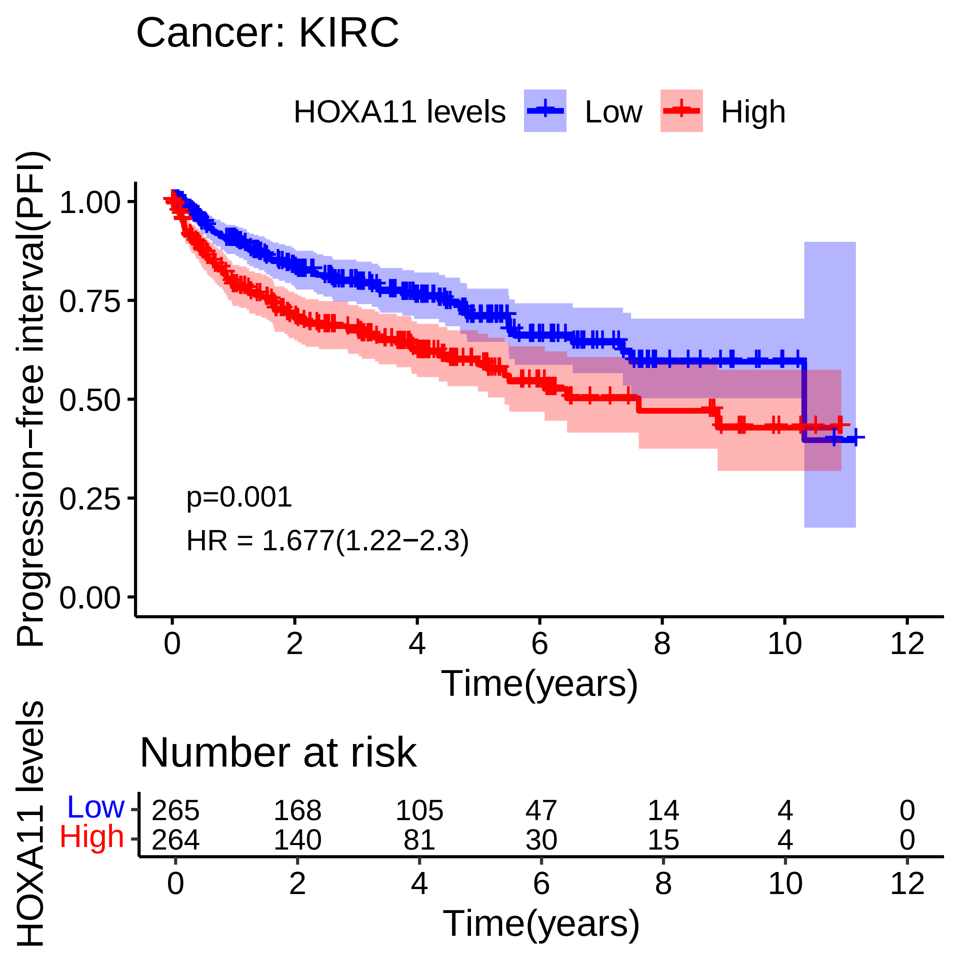

Cancer: KIRC
HOXA11 levels
Low
High
Progression−free interval(PFI)
1.00
0.75
0.50
0.25
0.00
p=0.001
HR = 1.677(1.22−2.3)
0
2
4
6
8
10
12
Time(years)
Number at risk
HOXA11 levels
Low 265 168 105 47 14 4 0
High 264 140 81 30 15 4 0
Time(years)

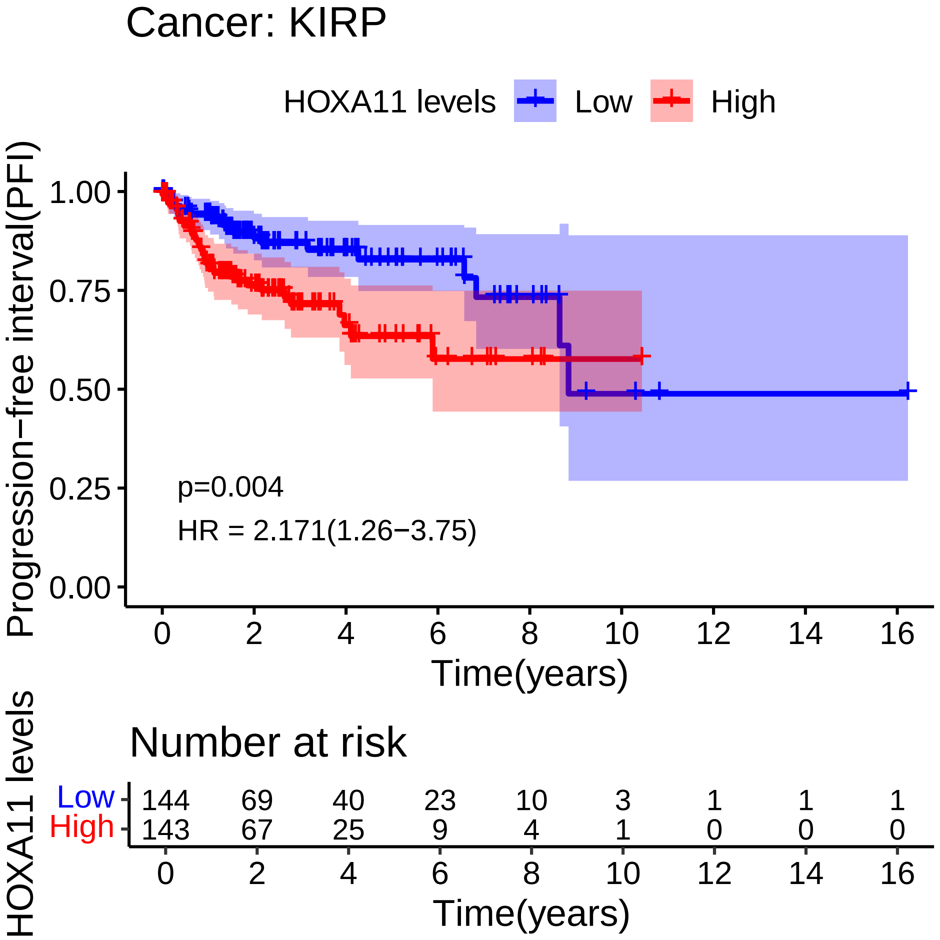

Cancer: KIRP
HOXA11 levels
Low
High
Progression−free interval(PFI)
1.00
0.75
0.50
0.25
0.00
p=0.004
HR = 2.171(1.26−3.75)
0
2
4
6
8
10
12
14
16
Time(years)
Number at risk
HOXA11 levels
Low 144 69 40 23 10 3 1 1 1
High 143 67 25 9 4 1 0 0 0
0
2
4
6
8
10
12
14
16
Time(years)

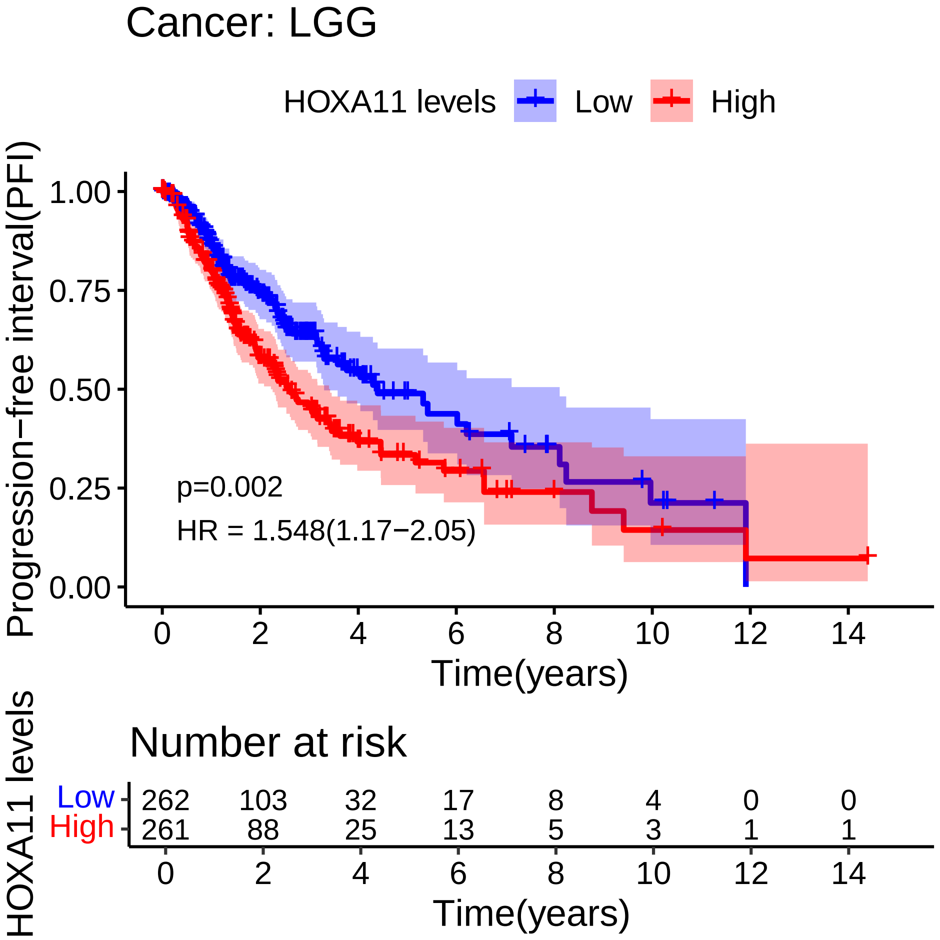

Cancer: LGG
HOXA11 levels
Low
High
Progression−free interval(PFI)
1.00
0.75
0.50
0.25
0.00
p=0.002
HR = 1.548(1.17−2.05)
0
2
4
6
8
10
12
14
Time(years)
Number at risk
HOXA11 levels
Low 262 103 32 17 8 4 0 0
High 261 88 25 13 5 3 1 1
Time(years)

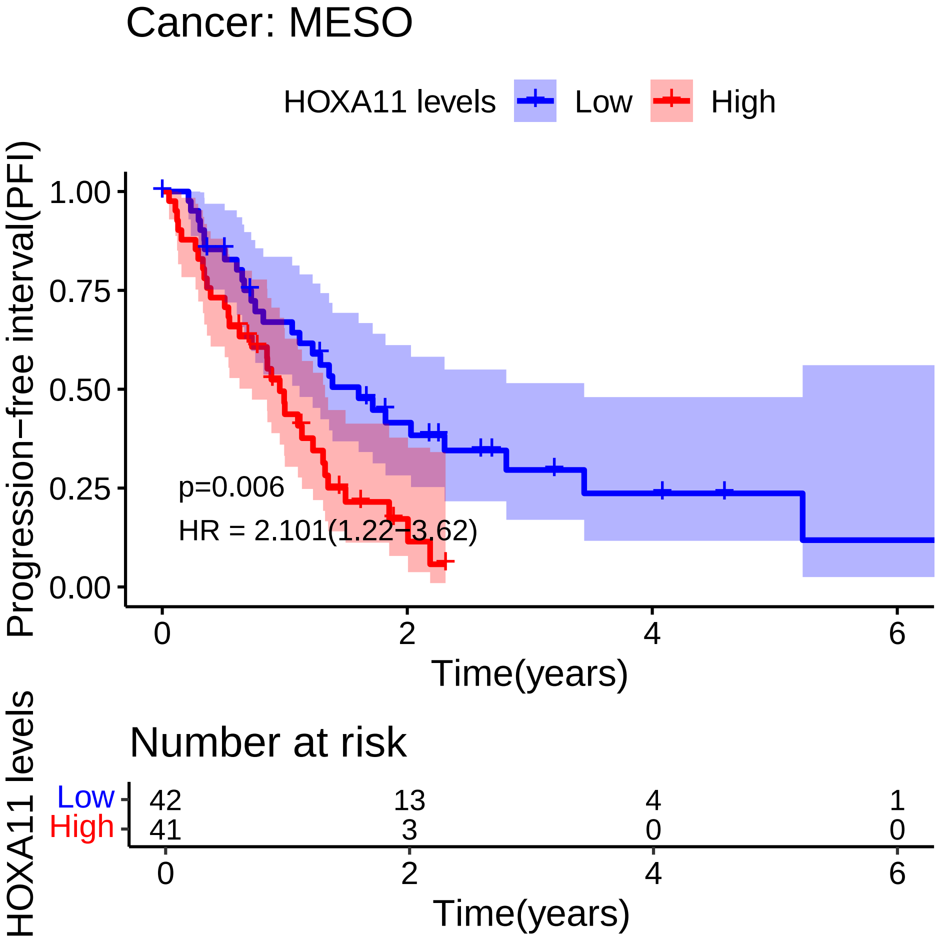
Cancer: MESO
HOXA11 levels
Low
High
Progression−free interval(PFI)
1.00
0.75
0.50
0.25
0.00
p=0.006
HR = 2.101(1.22−3.62)
0
2
4
6
Time(years)
Number at risk
HOXA11 levels
Low
High
42
13
4
1
41
3
0
0
0
2
4
6
Time(years)

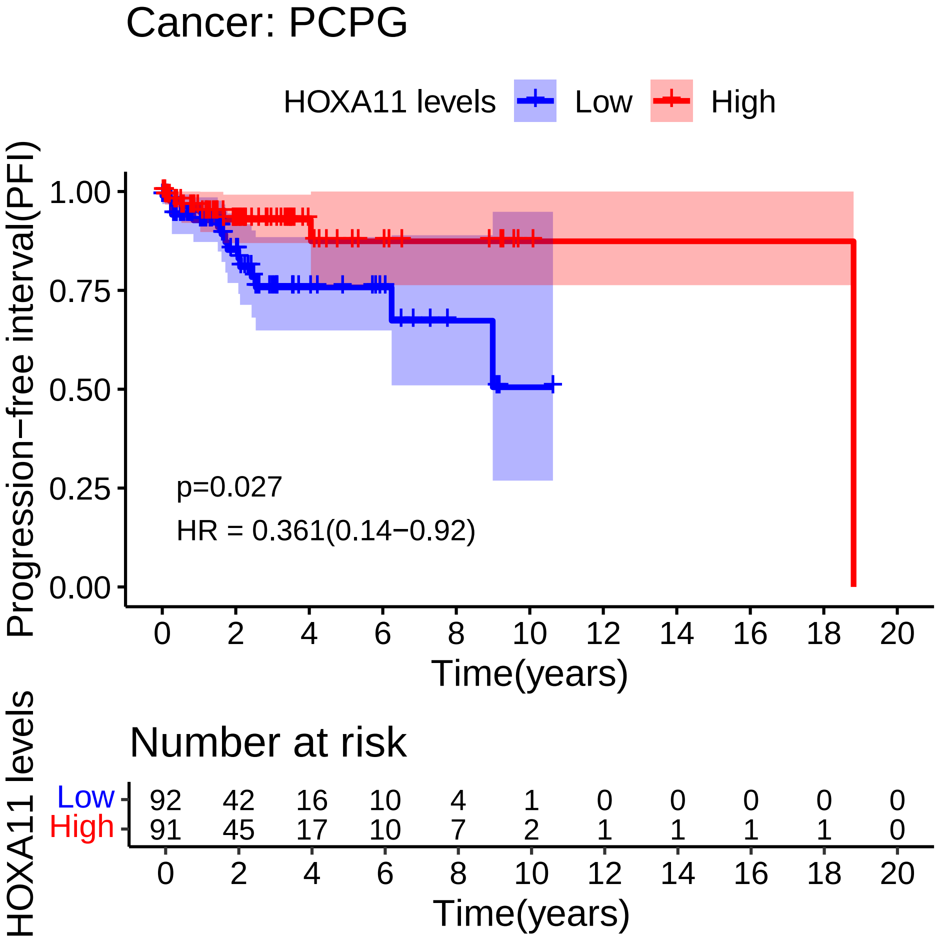

Cancer: PCPG
HOXA11 levels
Low
High
Progression−free interval(PFI)
1.00
0.75
0.50
0.25
0.00
p=0.027
HR = 0.361(0.14−0.92)
0
2
4
6
8
10
12
14
16
18
20
Time(years)
Number at risk
HOXA11 levels
Low 92 42 16 10 4 1 0 0 0 0 0
High 91 45 17 10 7 2 1 1 1 1 0
Time(years)

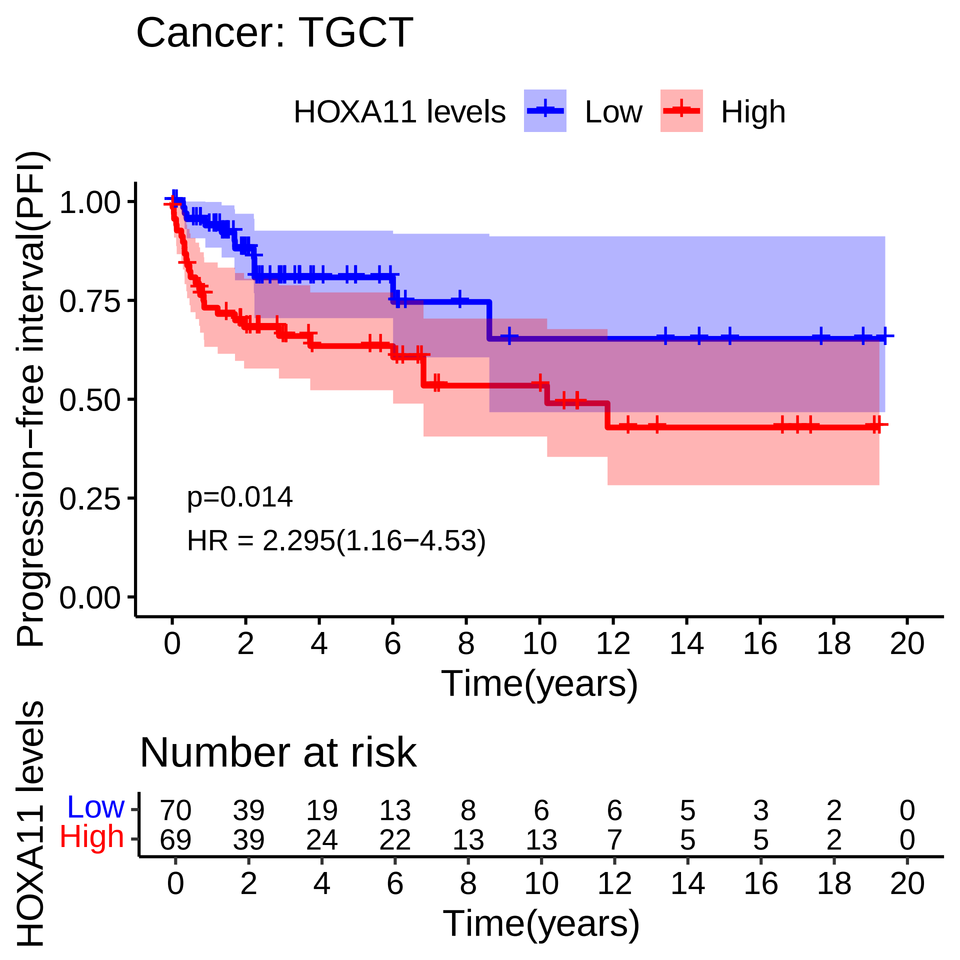

Cancer: TGCT
HOXA11 levels
Low
High
Progression−free interval(PFI)
1.00
0.75
0.50
0.25
0.00
p=0.014
HR = 2.295(1.16−4.53)
0
2
4
6
8
10
12
14
16
18
20
Time(years)
Number at risk
HOXA11 levels
Low 70 39 19 13 8 6 6 5 3 2 0
High 69 39 24 22 13 13 7 5 5 2 0
Time(years)

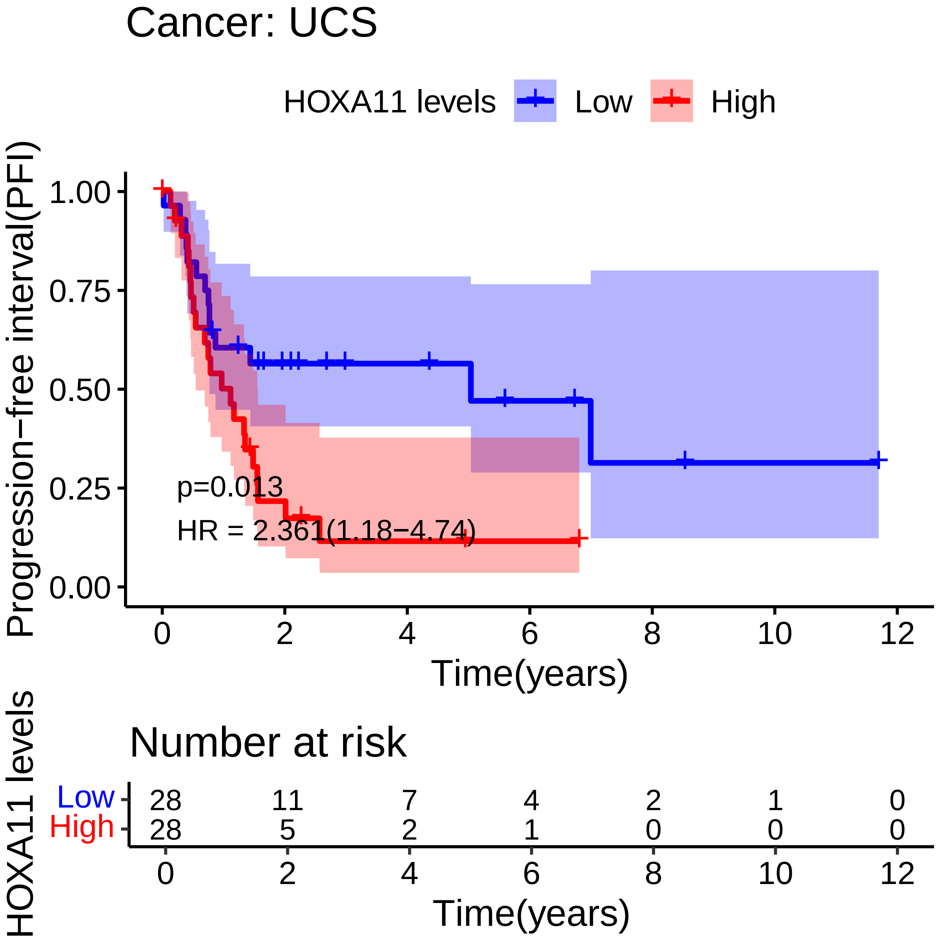
Cancer: UCS
HOXA11 levels
Low
High
Progression−free interval(PFI)
1.00
0.75
0.50
0.25
0.00
p=0.013
HR = 2.361(1.18−4.74)
0
2
4
6
8
10
12
Time(years)
Number at risk
HOXA11 levels
Low 28 11 7 4 2 1 0
High 28 5 2 1 0 0 0
Time(years)

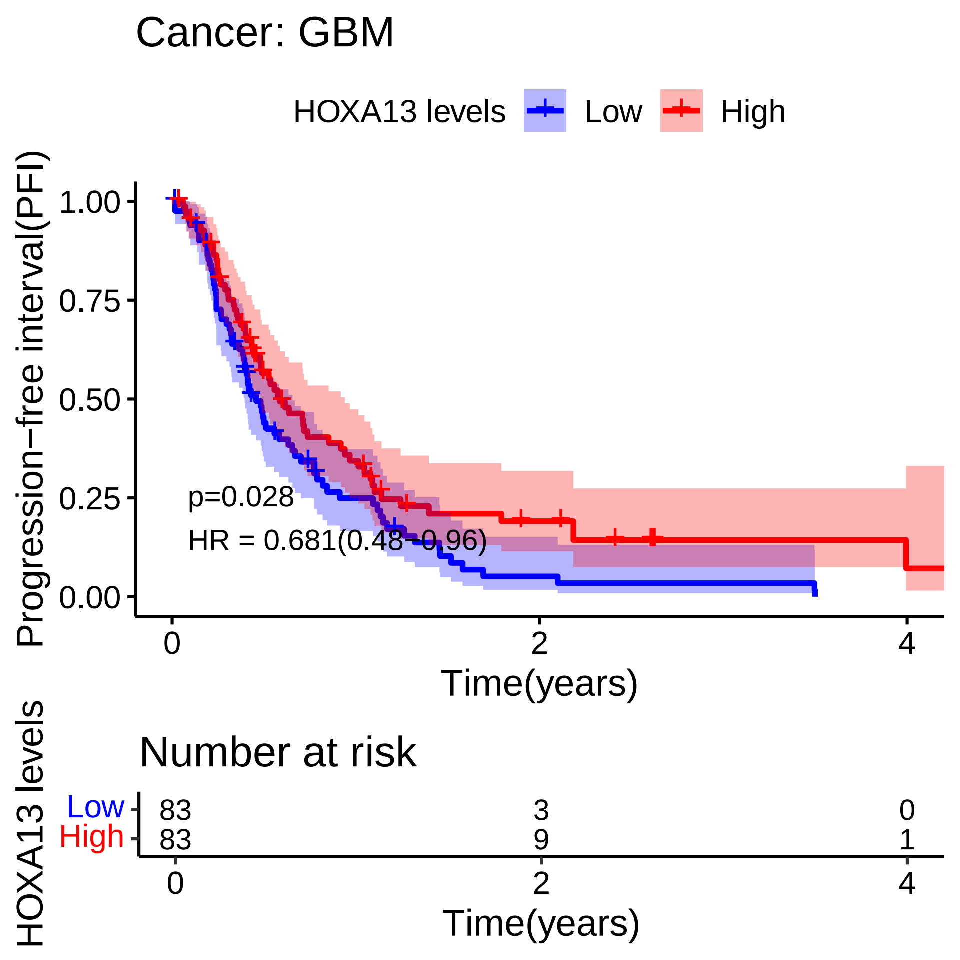
Cancer: GBM
HOXA13 levels
Low
High
Progression−free interval(PFI)
1.00
0.75
0.50
0.25
0.00
p=0.028
HR = 0.681(0.48−0.96)
0
2
4
Time(years)
Number at risk
HOXA13 levels
Low
High
83
3
0
83
9
1
0
2
4
Time(years)

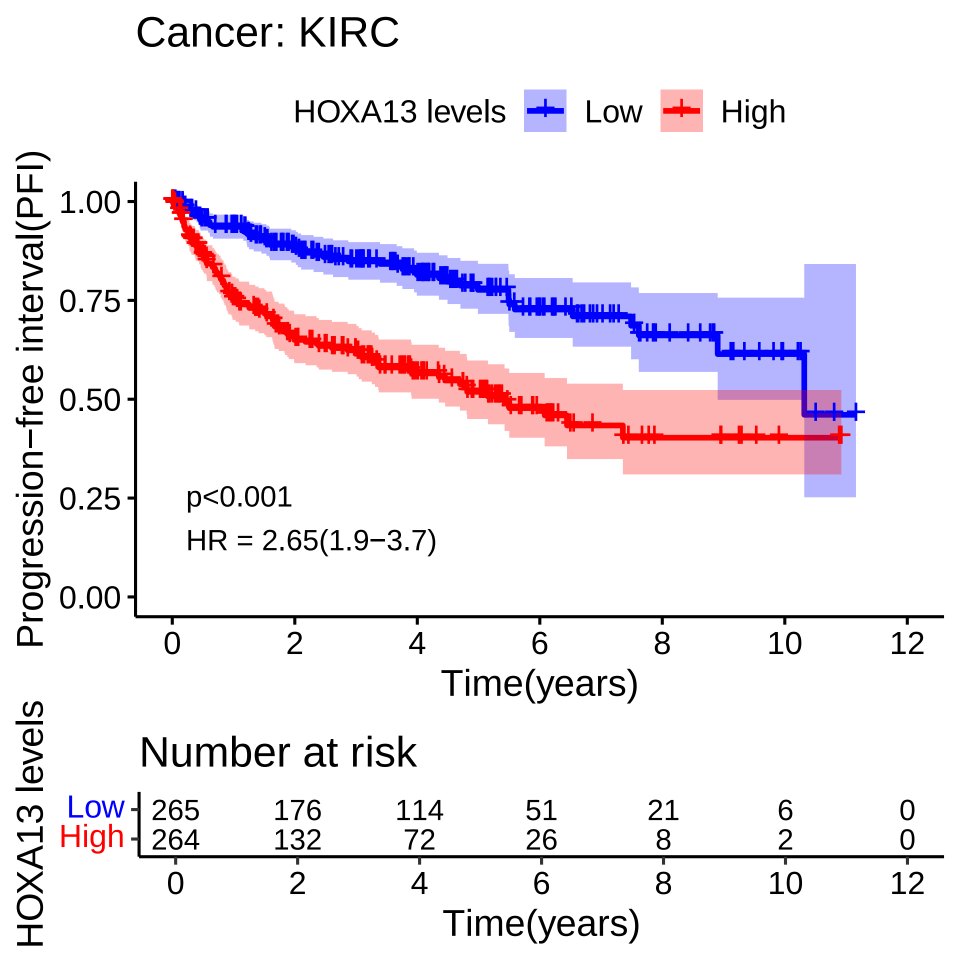

Cancer: KIRC
HOXA13 levels
Low
High
Progression−free interval(PFI)
1.00
0.75
0.50
0.25
0.00
p<0.001
HR = 2.65(1.9−3.7)
0
2
4
6
8
10
12
Time(years)
Number at risk
HOXA13 levels
Low 265 176 114 51 21 6 0
High 264 132 72 26 8 2 0
Time(years)

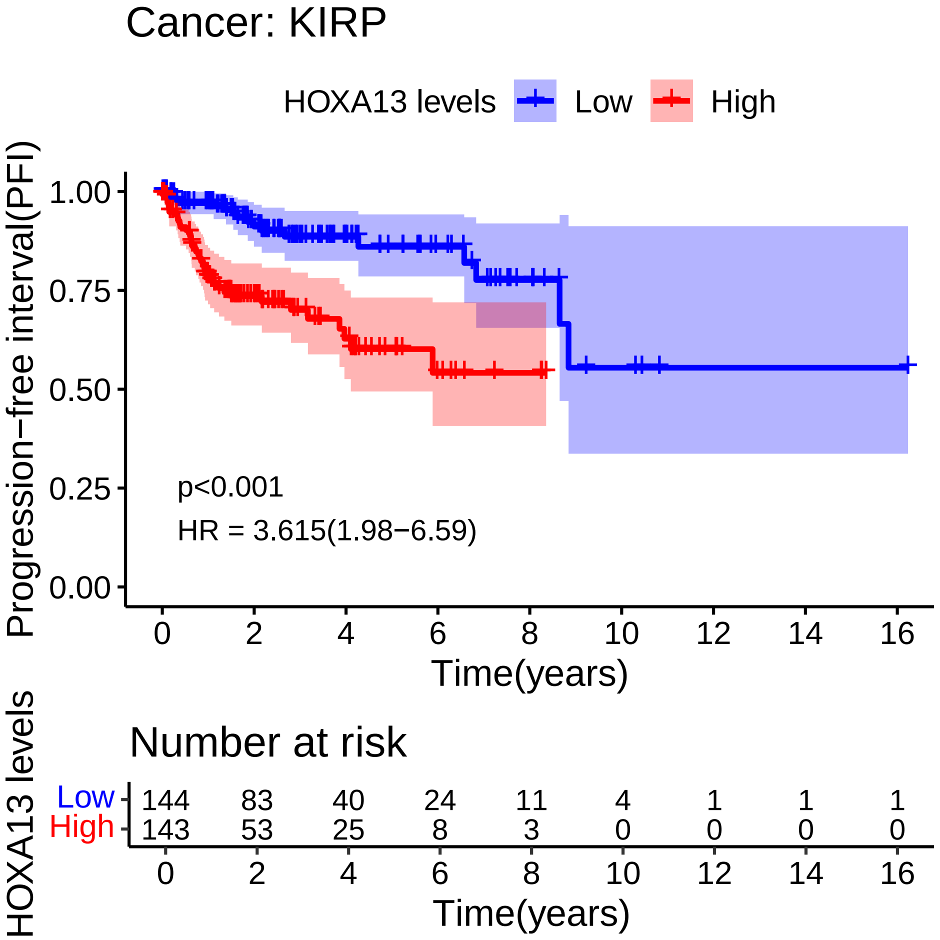

Cancer: KIRP
HOXA13 levels
Low
High
Progression−free interval(PFI)
1.00
0.75
0.50
0.25
0.00
p<0.001
HR = 3.615(1.98−6.59)
0
2
4
6
8
10
12
14
16
Time(years)
Number at risk
HOXA13 levels
Low 144 83 40 24 11 4 1 1 1
High 143 53 25 8 3 0 0 0 0
0
2
4
6
8
10
12
14
16
Time(years)

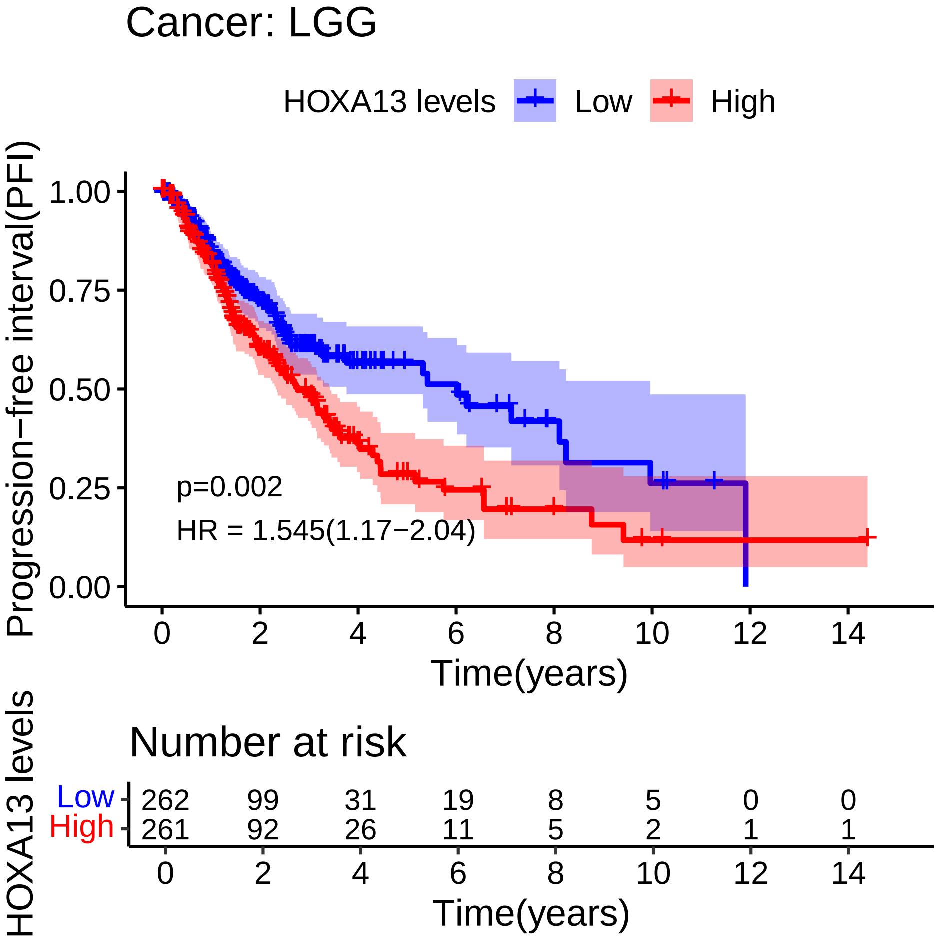
Cancer: LGG
HOXA13 levels
Low
High
Progression−free interval(PFI)
1.00
0.75
0.50
0.25
0.00
p=0.002
HR = 1.545(1.17−2.04)
0
2
4
6
8
10
12
14
Time(years)
Number at risk
HOXA13 levels
Low 262 99 31 19 8 5 0 0
High 261 92 26 11 5 2 1 1
Time(years)

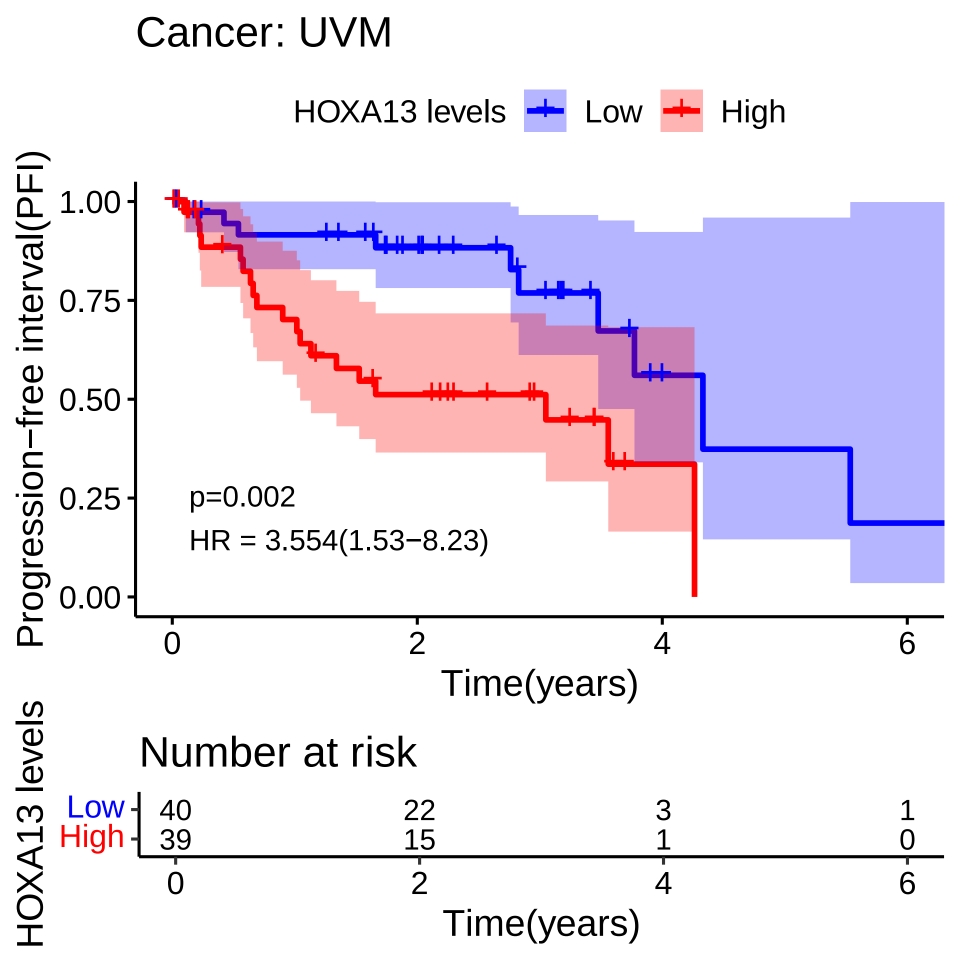

Cancer: UVM
HOXA13 levels
Low
High
Progression−free interval(PFI)
1.00
0.75
0.50
0.25
0.00
p=0.002
HR = 3.554(1.53−8.23)
0
2
4
6
Time(years)
Number at risk
HOXA13 levels
Low
High
40
22
3
1
39
15
1
0
0
2
4
6
Time(years)

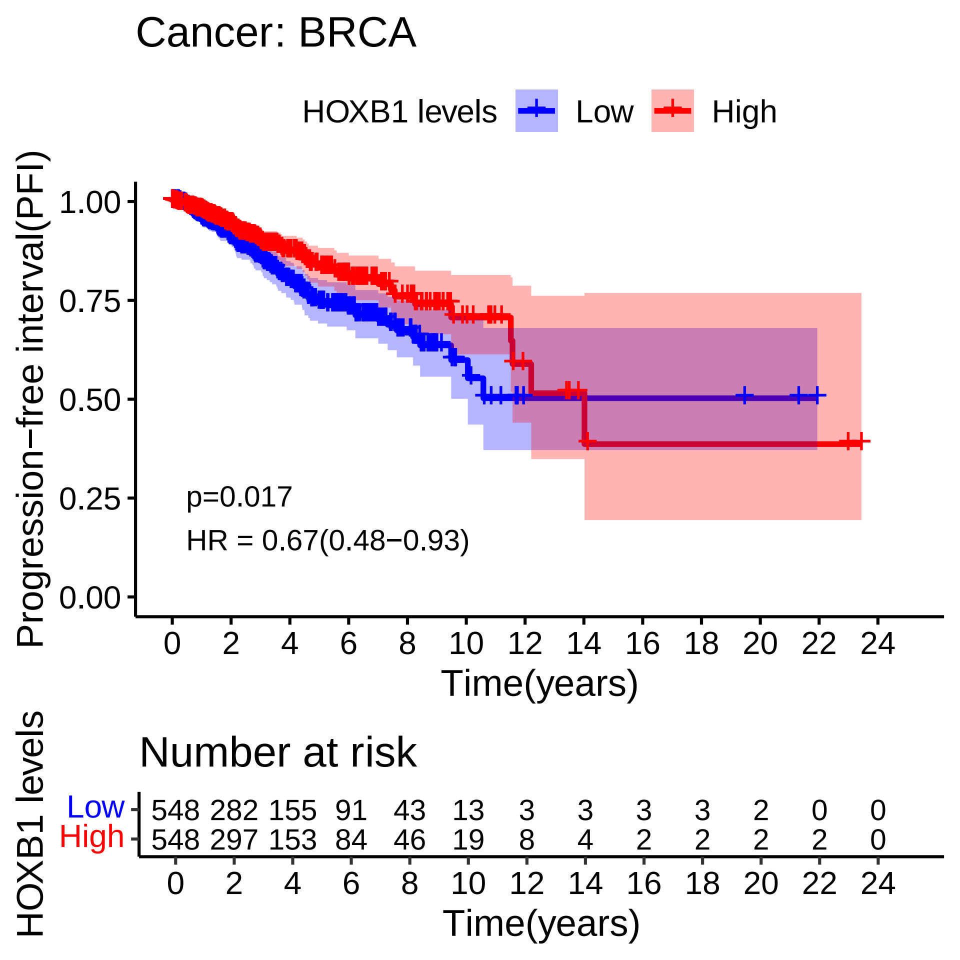
Cancer: BRCA
HOXB1 levels
Low
High
Progression−free interval(PFI)
1.00
0.75
0.50
0.25
0.00
p=0.017
HR = 0.67(0.48−0.93)
0 2 4 6 8 10 12 14 16 18 20 22 24
Time(years)
Number at risk
HOXB1 levels
Low 548 282 155 91 43 13 3 3 3 3 2 0 0
High 548 297 153 84 46 19 8 4 2 2 2 2 0
0 2 4 6 8 10 12 14 16 18 20 22 24
Time(years)

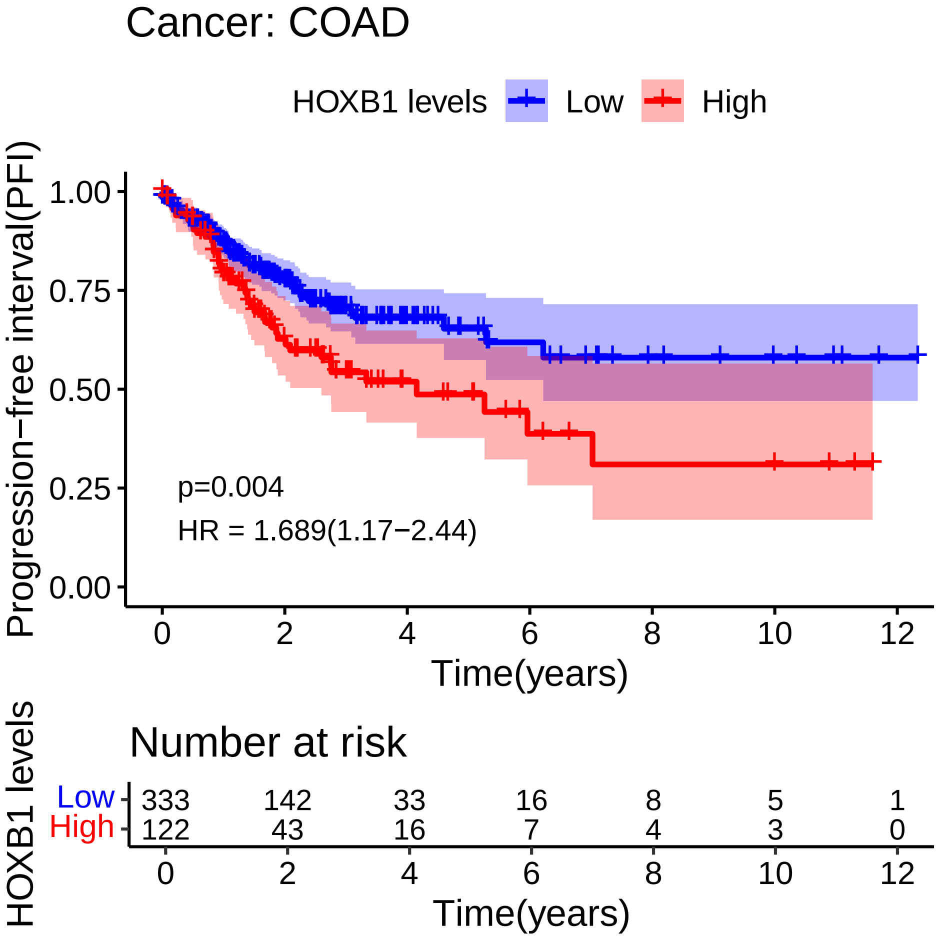

Cancer: COAD
HOXB1 levels
Low
High
Progression−free interval(PFI)
1.00
0.75
0.50
0.25
0.00
p=0.004
HR = 1.689(1.17−2.44)
0
2
4
6
8
10
12
Time(years)
Number at risk
HOXB1 levels
Low 333 142 33 16 8 5 1
High 122 43 16 7 4 3 0
Time(years)

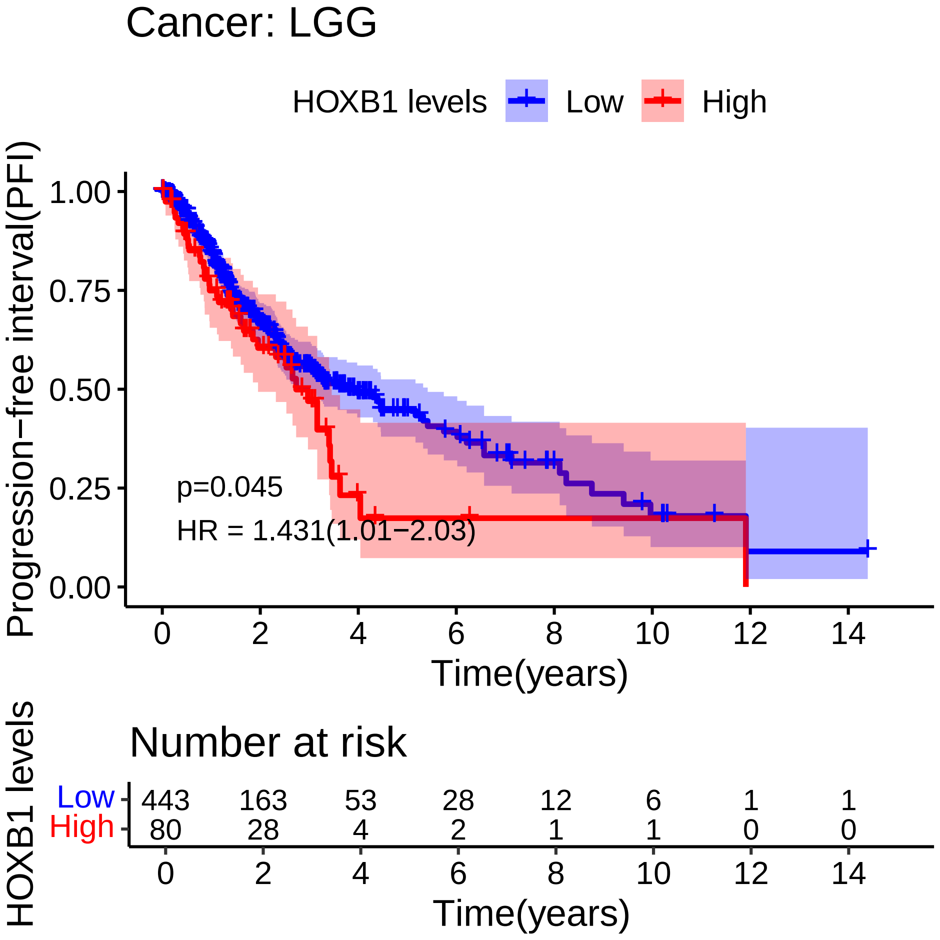

Cancer: LGG
HOXB1 levels
Low
High
Progression−free interval(PFI)
1.00
0.75
0.50
0.25
0.00
p=0.045
HR = 1.431(1.01−2.03)
0
2
4
6
8
10
12
14
Time(years)
Number at risk
HOXB1 levels
Low 443 163 53 28 12 6 1 1
High 80 28 4 2 1 1 0 0
Time(years)

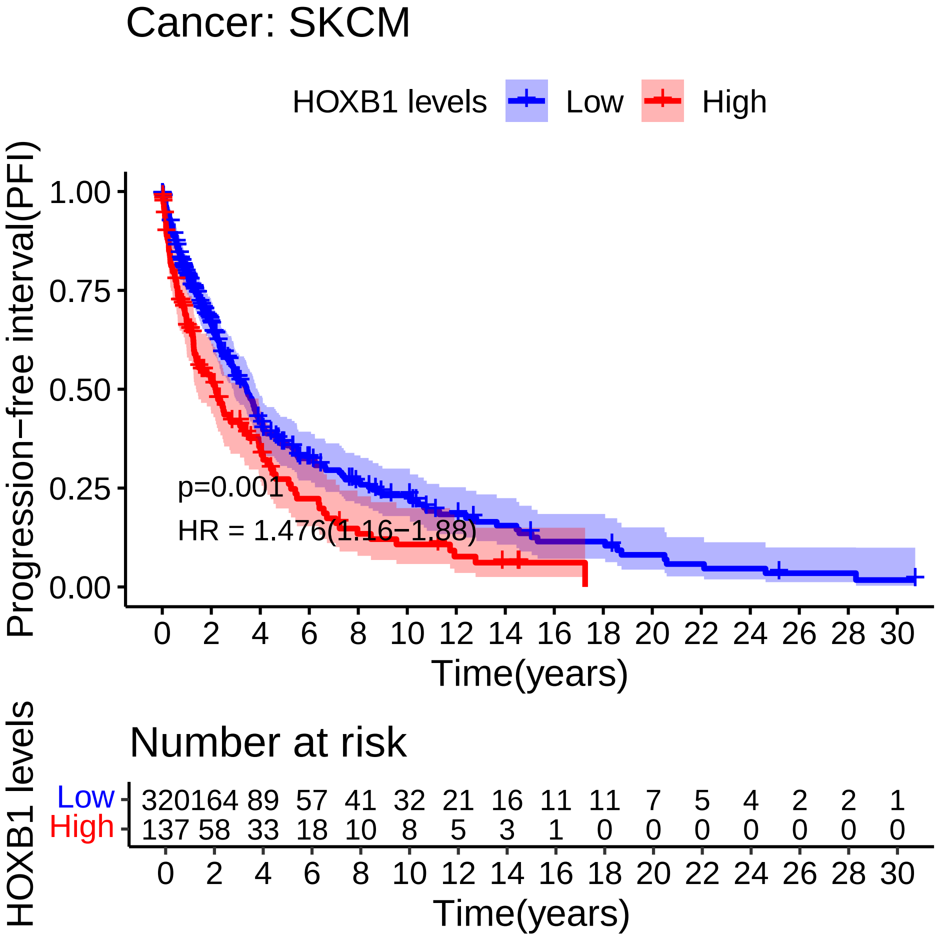

Cancer: SKCM
HOXB1 levels
Low
High
Progression−free interval(PFI)
1.00
0.75
0.50
0.25
0.00
p=0.001
HR = 1.476(1.16−1.88)
0 2 4 6 8 10 12 14 16 18 20 22 24 26 28 30
Time(years)
Number at risk
HOXB1 levels
Low 320 164 89 57 41 32 21 16 11 11 7 5 4 2 2 1
High 137 58 33 18 10 8 5 3 1 0 0 0 0 0 0 0
0 2 4 6 8 10 12 14 16 18 20 22 24 26 28 30
Time(years)

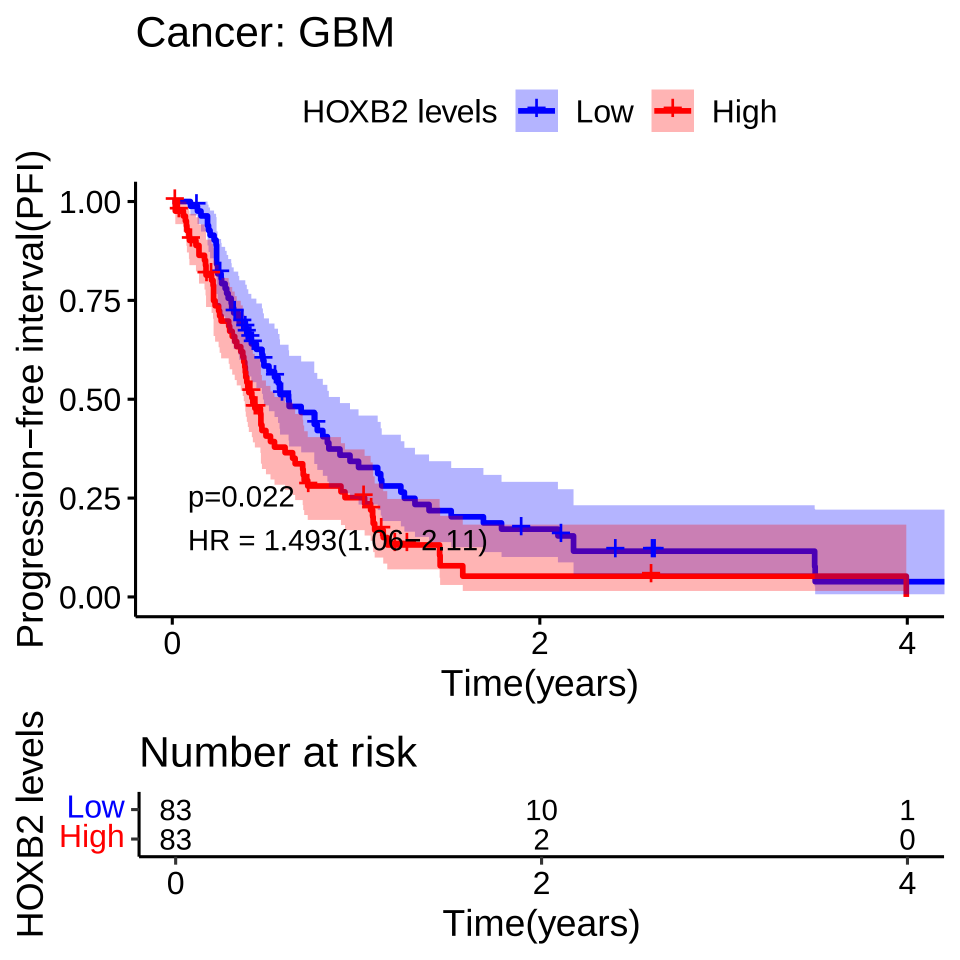

Cancer: GBM
HOXB2 levels
Low
High
Progression−free interval(PFI)
1.00
0.75
0.50
0.25
0.00
p=0.022
HR = 1.493(1.06−2.11)
0
2
4
Time(years)
Number at risk
HOXB2 levels
Low
High
83
10
1
83
2
0
0
2
4
Time(years)

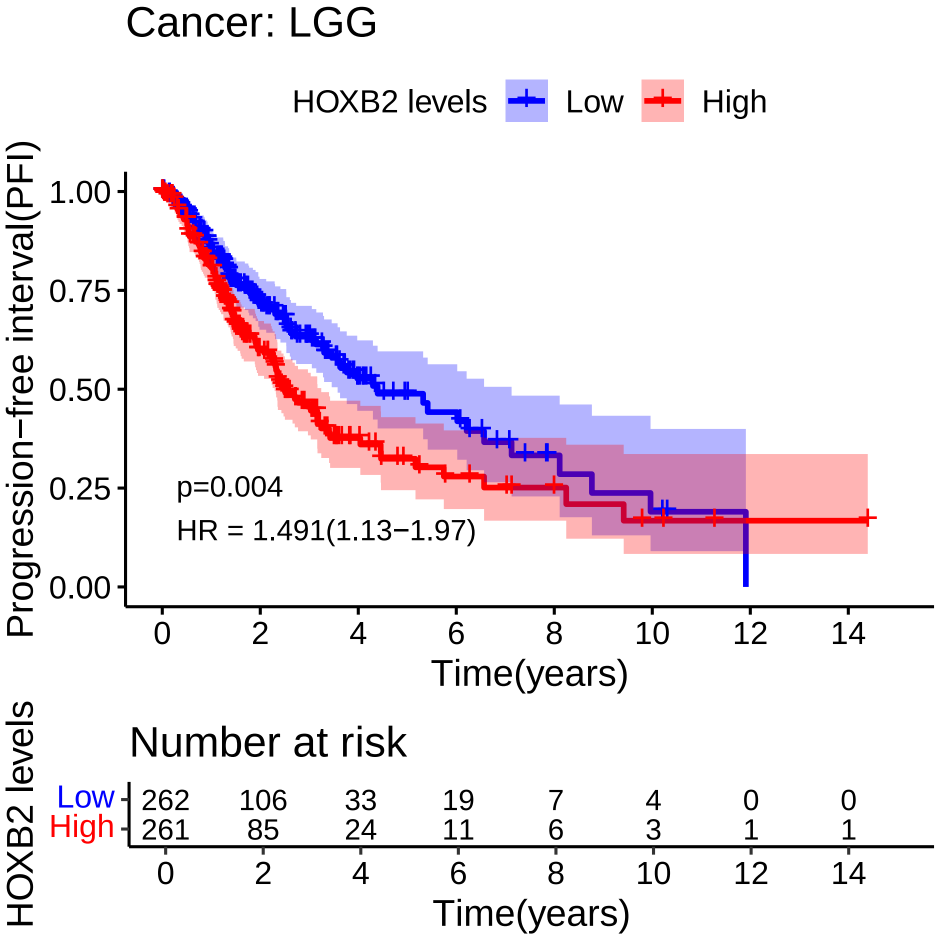

Cancer: LGG
HOXB2 levels
Low
High
Progression−free interval(PFI)
1.00
0.75
0.50
0.25
0.00
p=0.004
HR = 1.491(1.13−1.97)
0
2
4
6
8
10
12
14
Time(years)
Number at risk
HOXB2 levels
Low 262 106 33 19 7 4 0 0
High 261 85 24 11 6 3 1 1
Time(years)

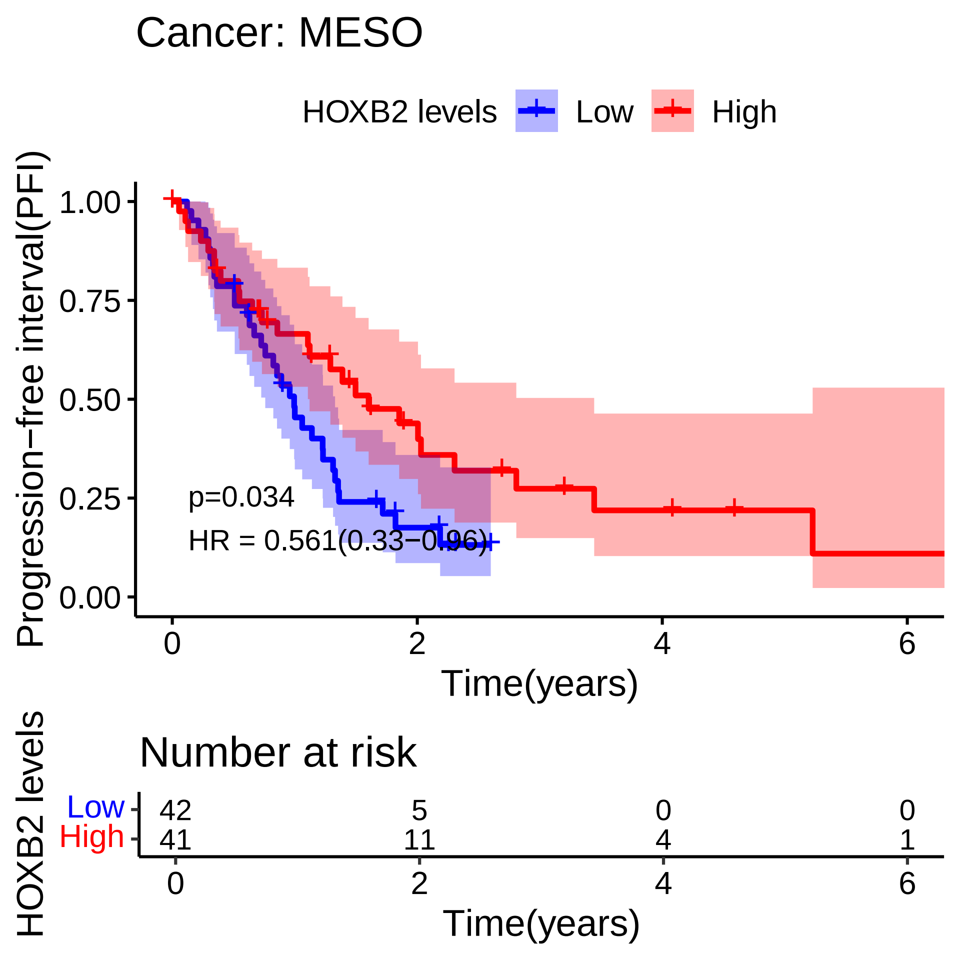

Cancer: MESO
HOXB2 levels
Low
High
Progression−free interval(PFI)
1.00
0.75
0.50
0.25
0.00
p=0.034
HR = 0.561(0.33−0.96)
0
2
4
6
Time(years)
Number at risk
HOXB2 levels
Low 42 5 0 0
High 41 11 4 1
0
2
4
6
Time(years)

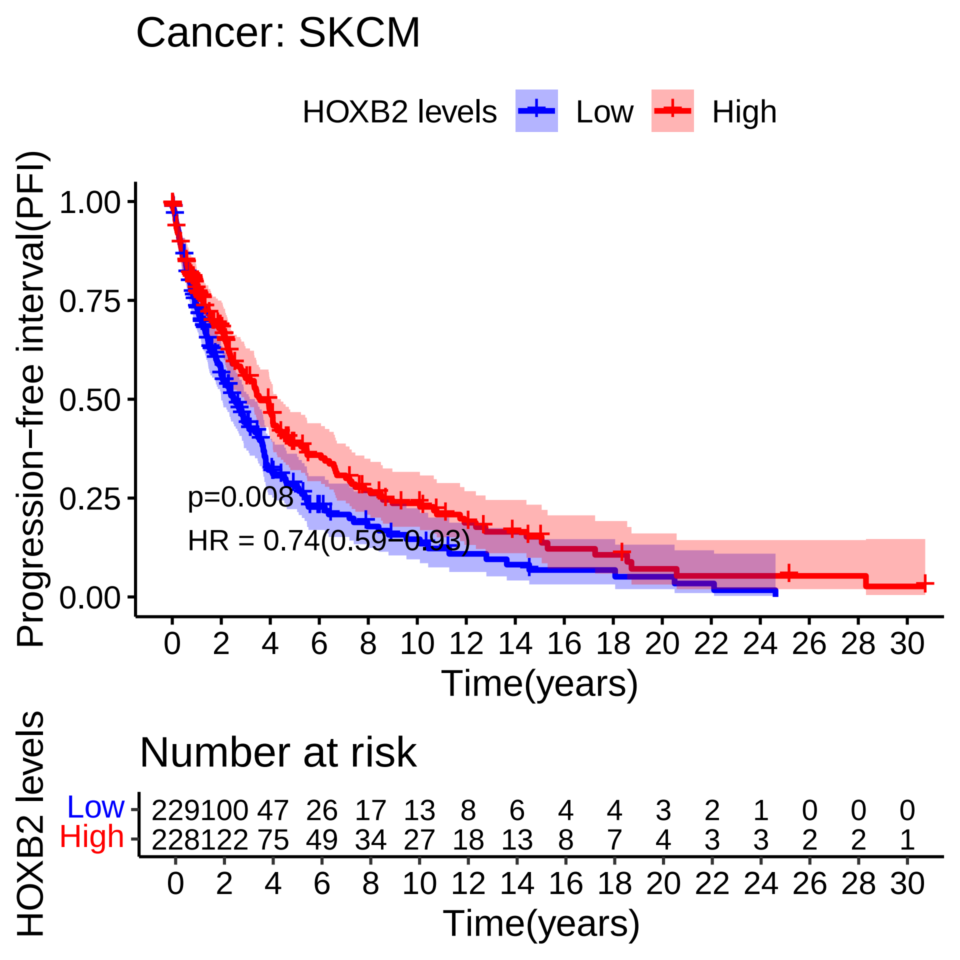
Cancer: SKCM
HOXB2 levels
Low
High
Progression−free interval(PFI)
1.00
0.75
0.50
0.25
0.00
p=0.008
HR = 0.74(0.59−0.93)
0 2 4 6 8 10 12 14 16 18 20 22 24 26 28 30
Time(years)
Number at risk
HOXB2 levels
Low 229 100 47 26 17 13 8 6 4 4 3 2 1 0 0 0
High 228 122 75 49 34 27 18 13 8 7 4 3 3 2 2 1
0 2 4 6 8 10 12 14 16 18 20 22 24 26 28 30
Time(years)

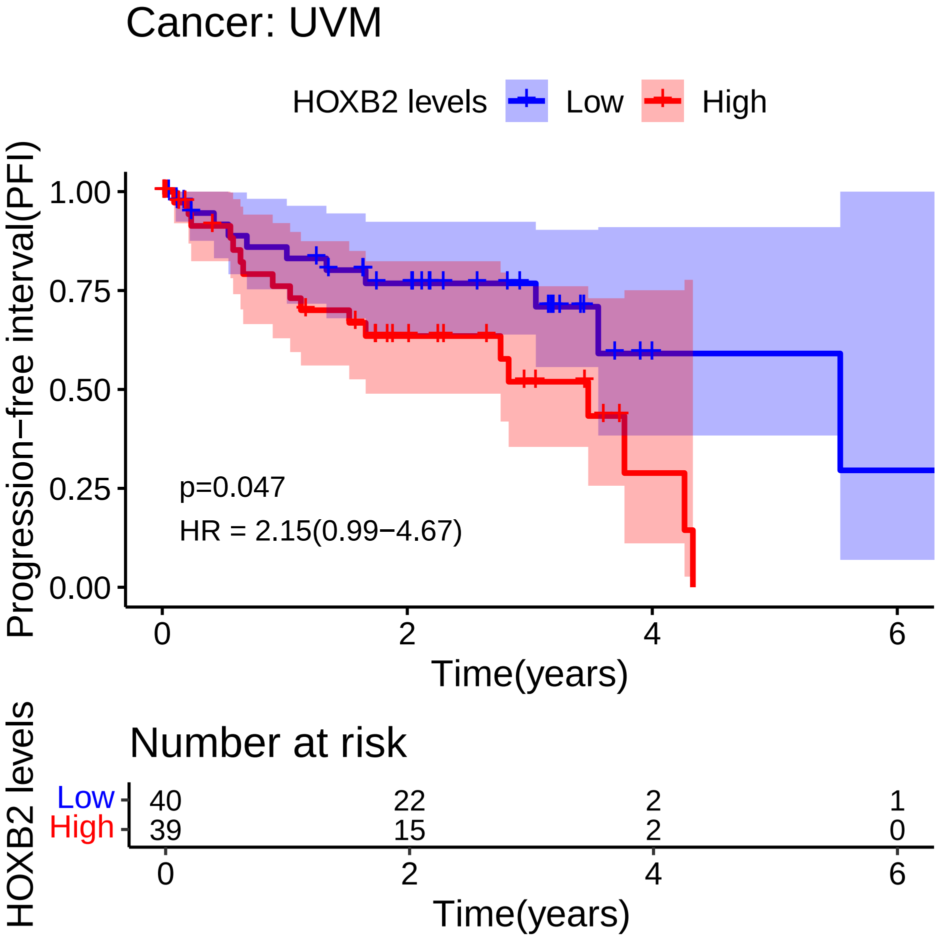
Cancer: UVM
HOXB2 levels
Low
High
Progression−free interval(PFI)
1.00
0.75
0.50
0.25
0.00
p=0.047
HR = 2.15(0.99−4.67)
0
2
4
6
Time(years)
Number at risk
HOXB2 levels
Low
High
40
22
2
1
39
15
2
0
0
2
4
6
Time(years)

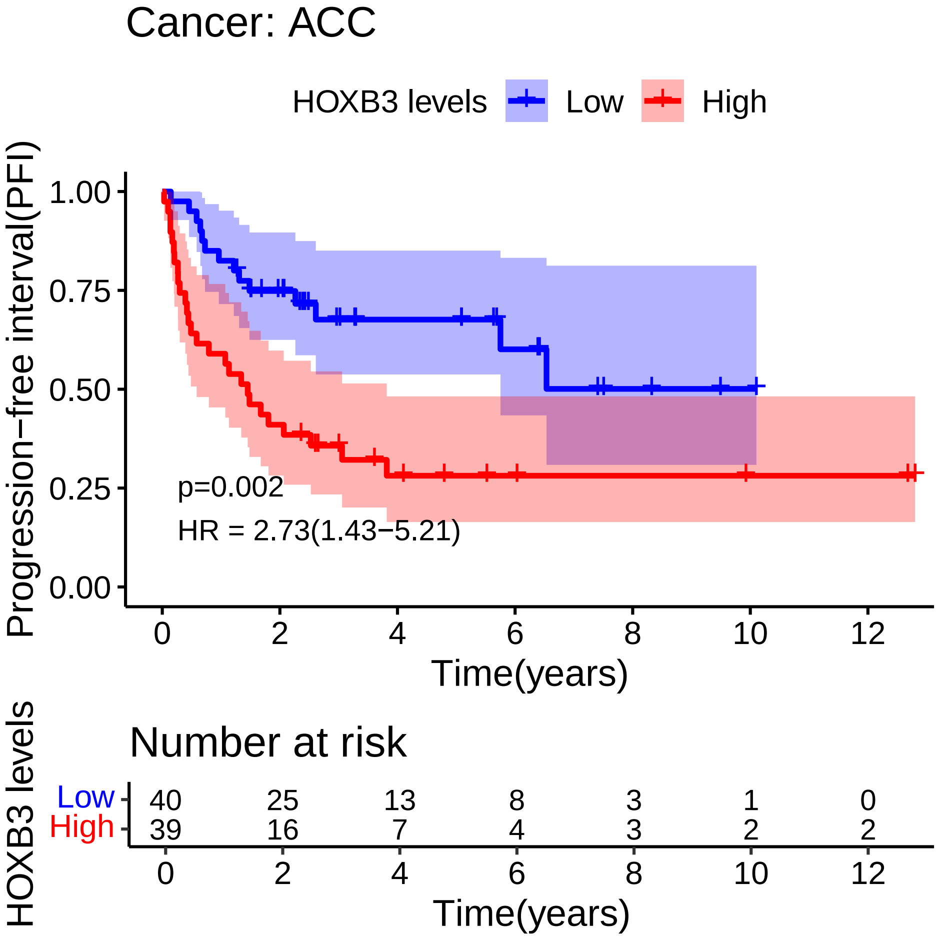
Cancer: ACC
HOXB3 levels
Low
High
Progression−free interval(PFI)
1.00
0.75
0.50
0.25
0.00
p=0.002
HR = 2.73(1.43−5.21)
0
2
4
6
8
10
12
Time(years)
Number at risk
HOXB3 levels
Low 40 25 13 8 3 1 0
High 39 16 7 4 3 2 2
0
2
4
6
8
10
12
Time(years)

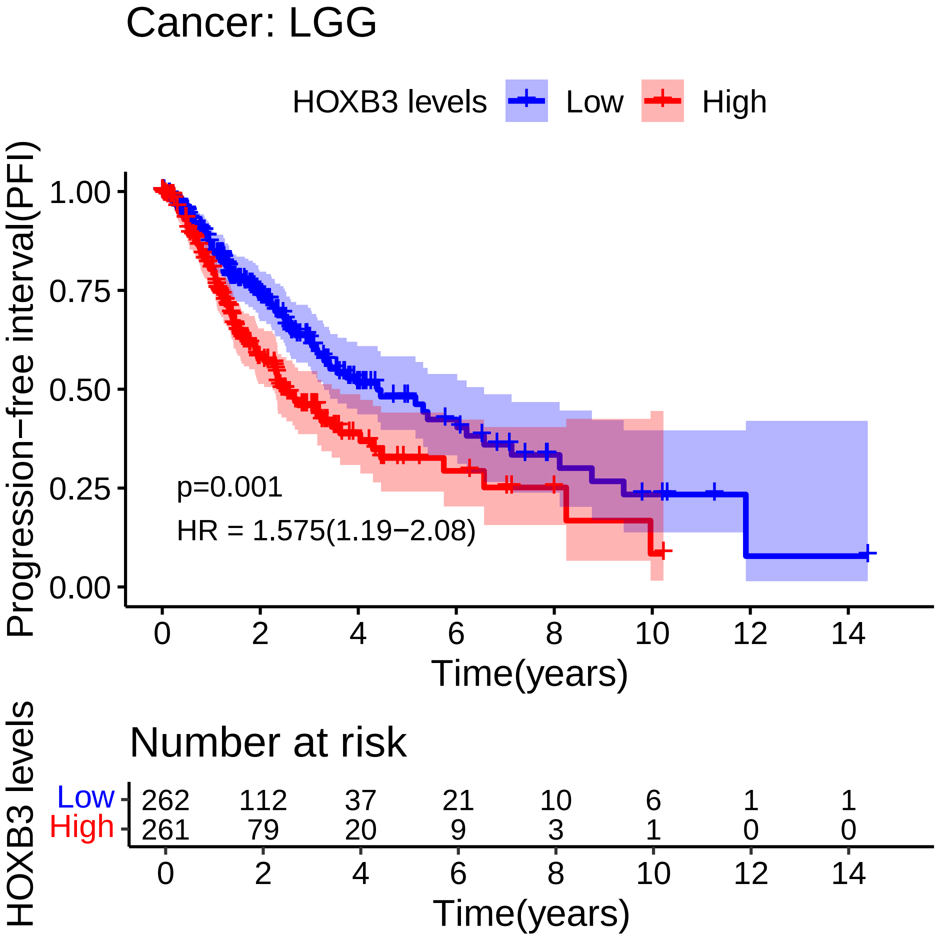
Cancer: LGG
HOXB3 levels
Low
High
Progression−free interval(PFI)
1.00
0.75
0.50
0.25
0.00
p=0.001
HR = 1.575(1.19−2.08)
0
2
4
6
8
10
12
14
Time(years)
Number at risk
HOXB3 levels
Low 262 112 37 21 10 6 1 1
High 261 79 20 9 3 1 0 0
Time(years)

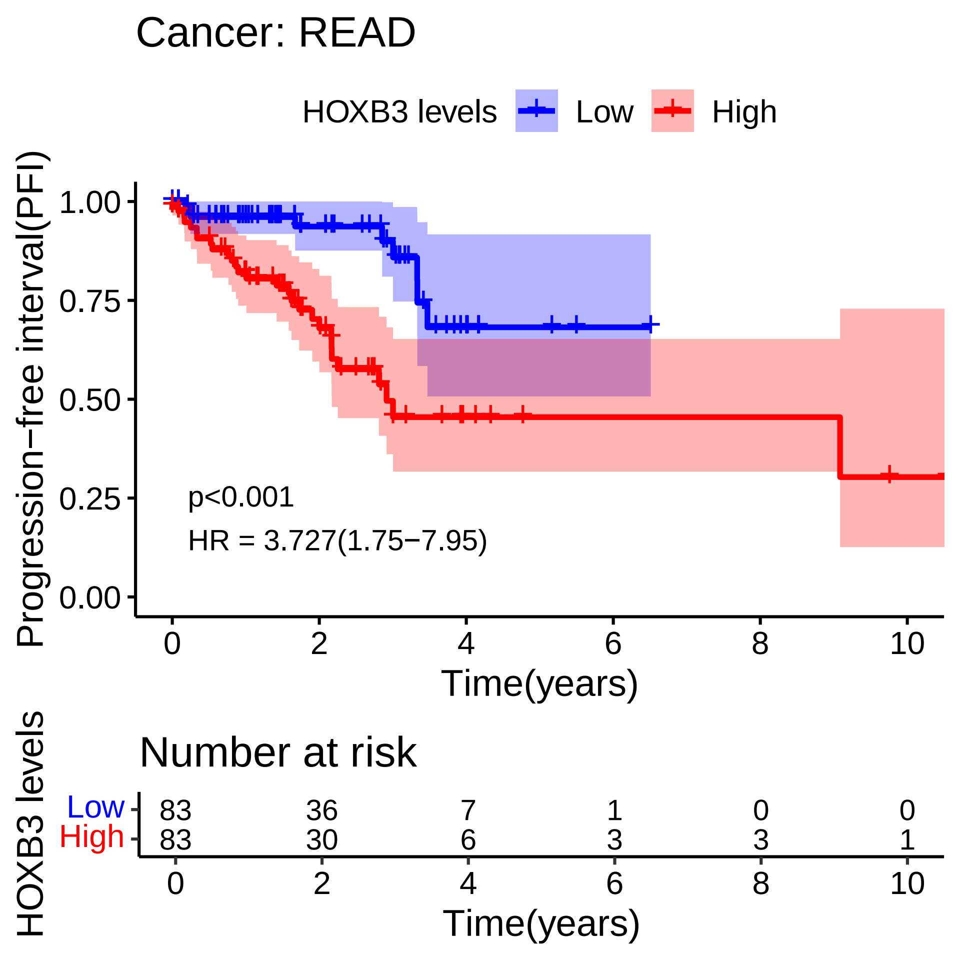

Cancer: READ
HOXB3 levels
Low
High
Progression−free interval(PFI)
1.00
0.75
0.50
0.25
0.00
p<0.001
HR = 3.727(1.75−7.95)
0
2
4
6
8
10
Time(years)
Number at risk
HOXB3 levels
Low 83 36 7 1 0 0
High 83 30 6 3 3 1
0
2
4
6
8
10
Time(years)

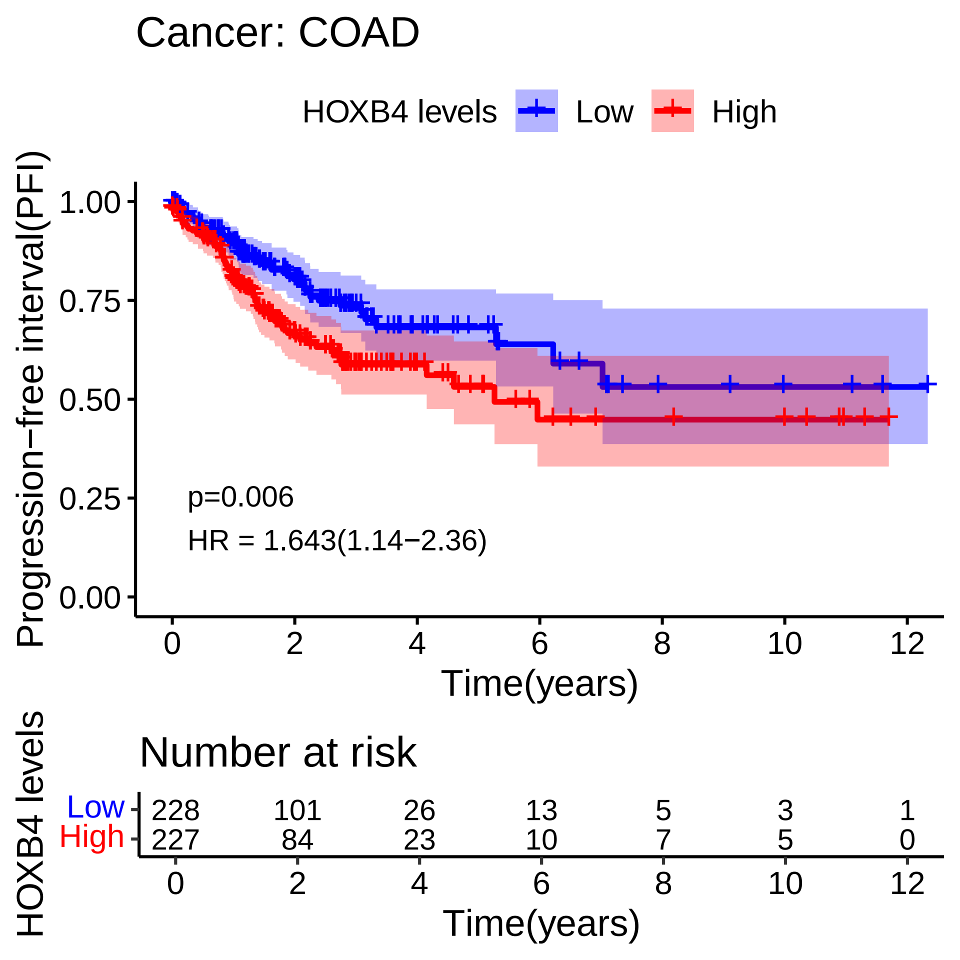

Cancer: COAD
HOXB4 levels
Low
High
Progression−free interval(PFI)
1.00
0.75
0.50
0.25
0.00
p=0.006
HR = 1.643(1.14−2.36)
0
2
4
6
8
10
12
Time(years)
Number at risk
HOXB4 levels
Low 228 101 26 13 5 3 1
High 227 84 23 10 7 5 0
Time(years)

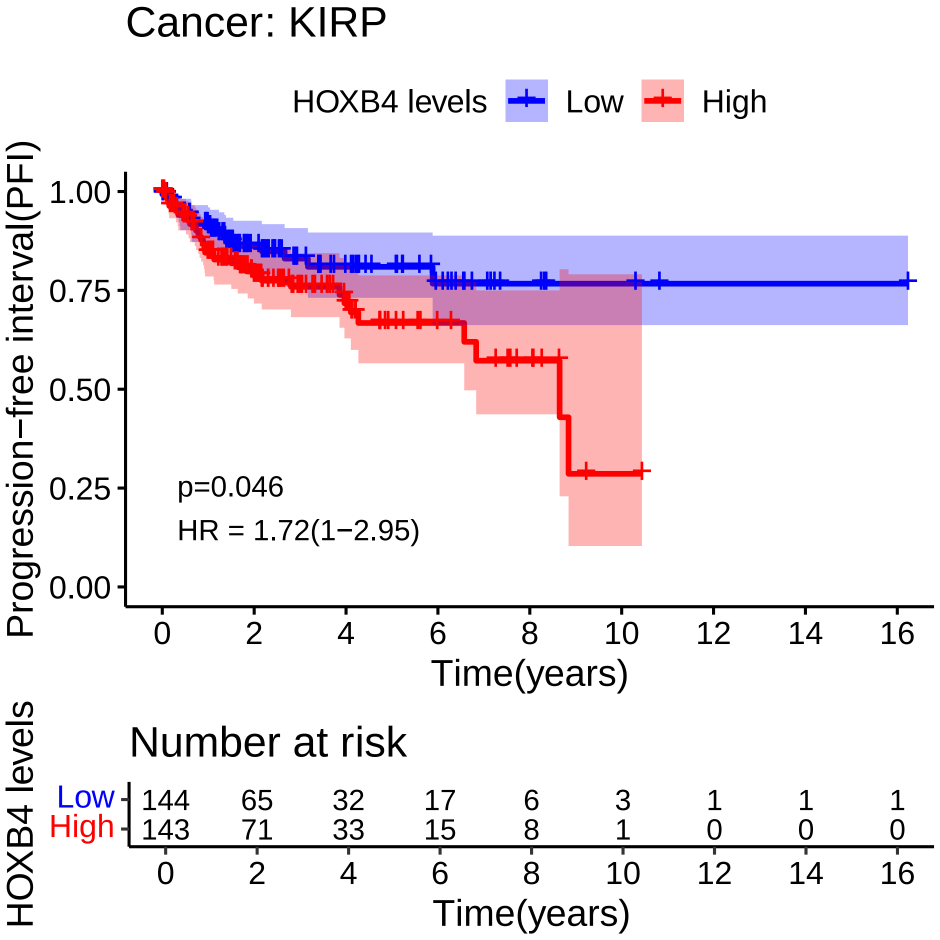

Cancer: KIRP
HOXB4 levels
Low
High
Progression−free interval(PFI)
1.00
0.75
0.50
0.25
0.00
p=0.046
HR = 1.72(1−2.95)
0
2
4
6
8
10
12
14
16
Time(years)
Number at risk
HOXB4 levels
Low 144 65 32 17 6 3 1 1 1
High 143 71 33 15 8 1 0 0 0
Time(years)

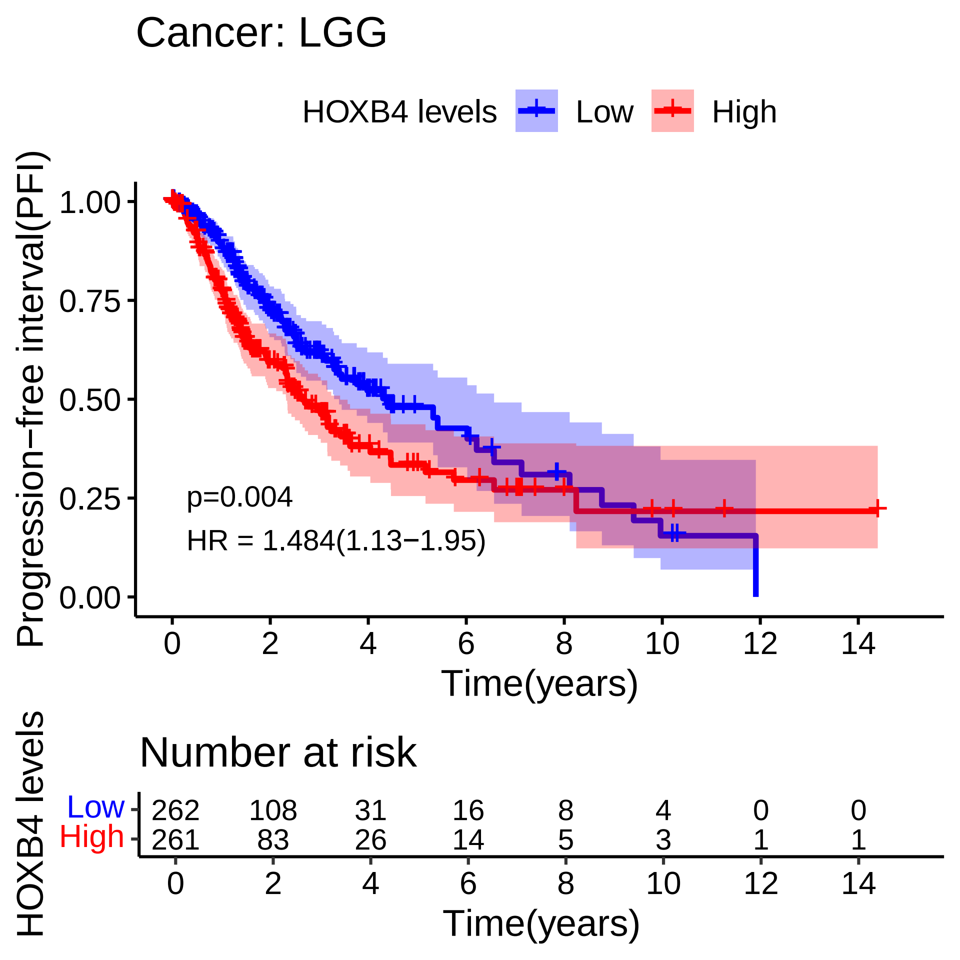

Cancer: LGG
HOXB4 levels
Low
High
Progression−free interval(PFI)
1.00
0.75
0.50
0.25
0.00
p=0.004
HR = 1.484(1.13−1.95)
0
2
4
6
8
10
12
14
Time(years)
Number at risk
HOXB4 levels
Low
High
262 108 31 16 8 4 0 0
261 83 26 14 5 3 1 1
Time(years)

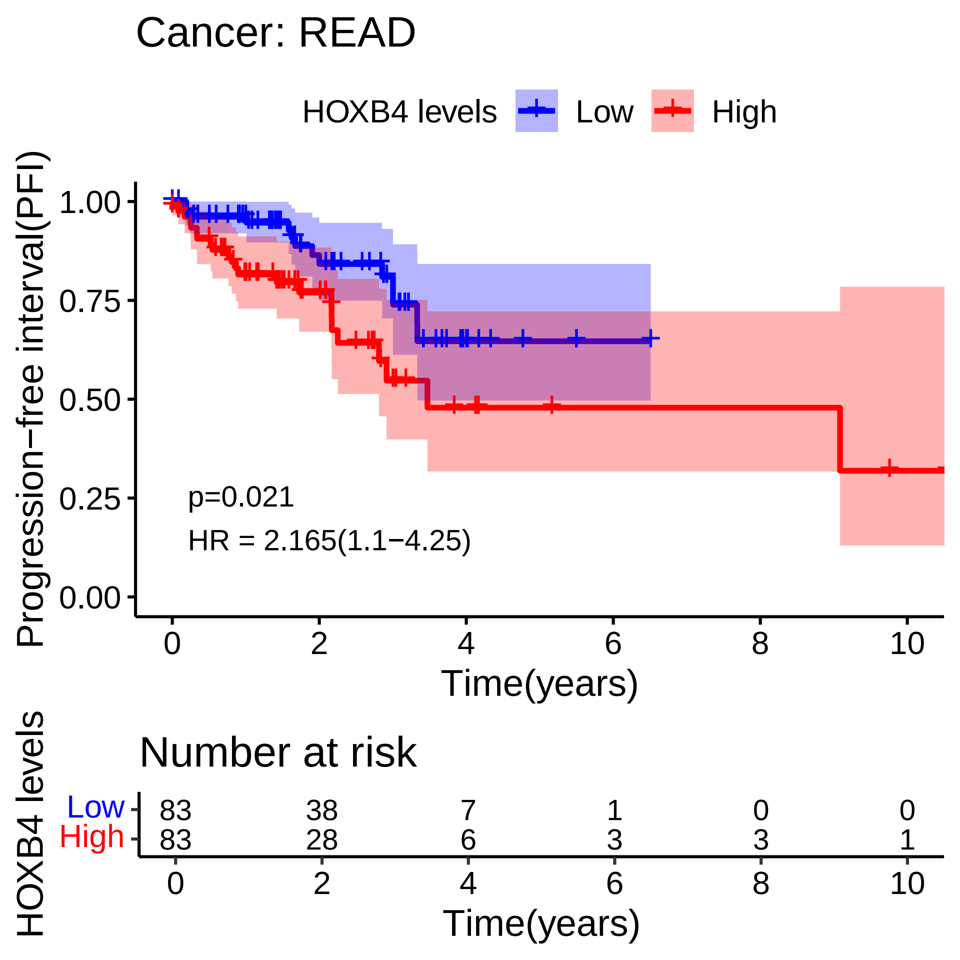

Cancer: READ
HOXB4 levels
Low
High
Progression−free interval(PFI)
1.00
0.75
0.50
0.25
0.00
p=0.021
HR = 2.165(1.1−4.25)
0
2
4
6
8
10
Time(years)
Number at risk
HOXB4 levels
Low
High
83 38 7 1 0 0
83 28 6 3 3 1
Time(years)

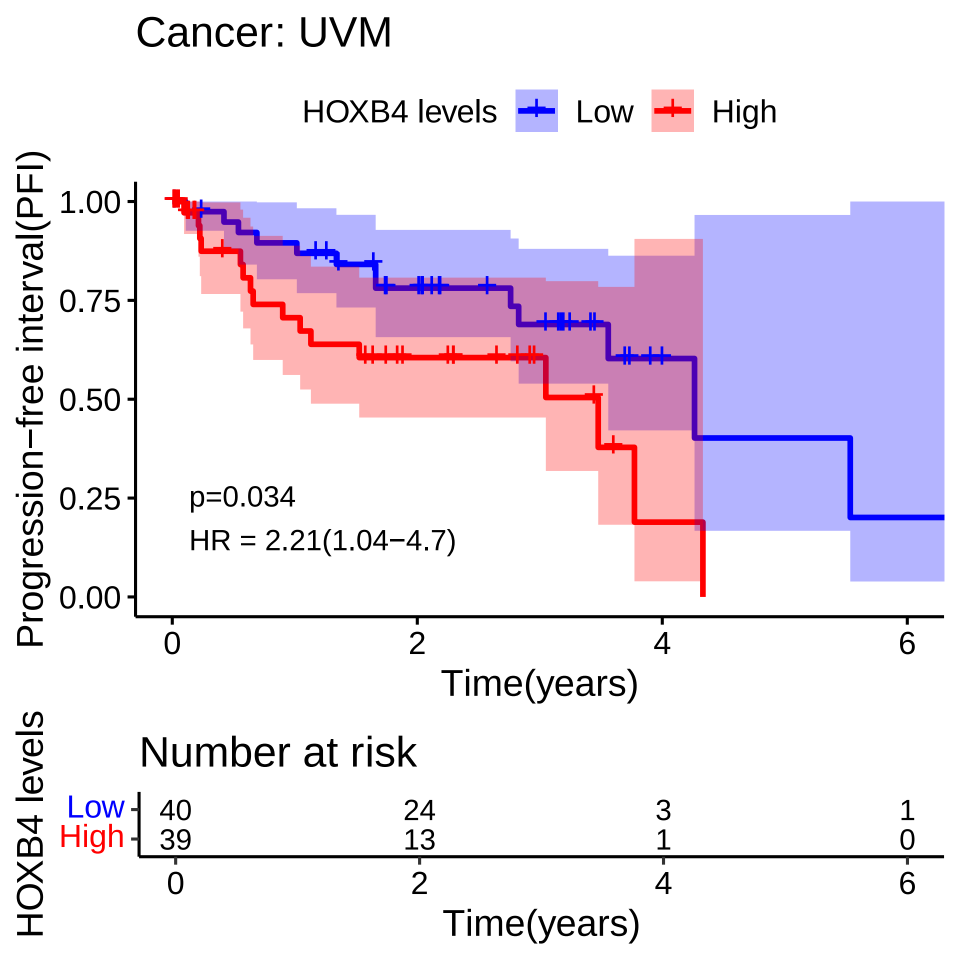

Cancer: UVM
HOXB4 levels
Low
High
Progression−free interval(PFI)
1.00
0.75
0.50
0.25
0.00
p=0.034
HR = 2.21(1.04−4.7)
0
2
4
6
Time(years)
Number at risk
HOXB4 levels
Low
High
40
24
3
1
39
13
1
0
0
2
4
6
Time(years)

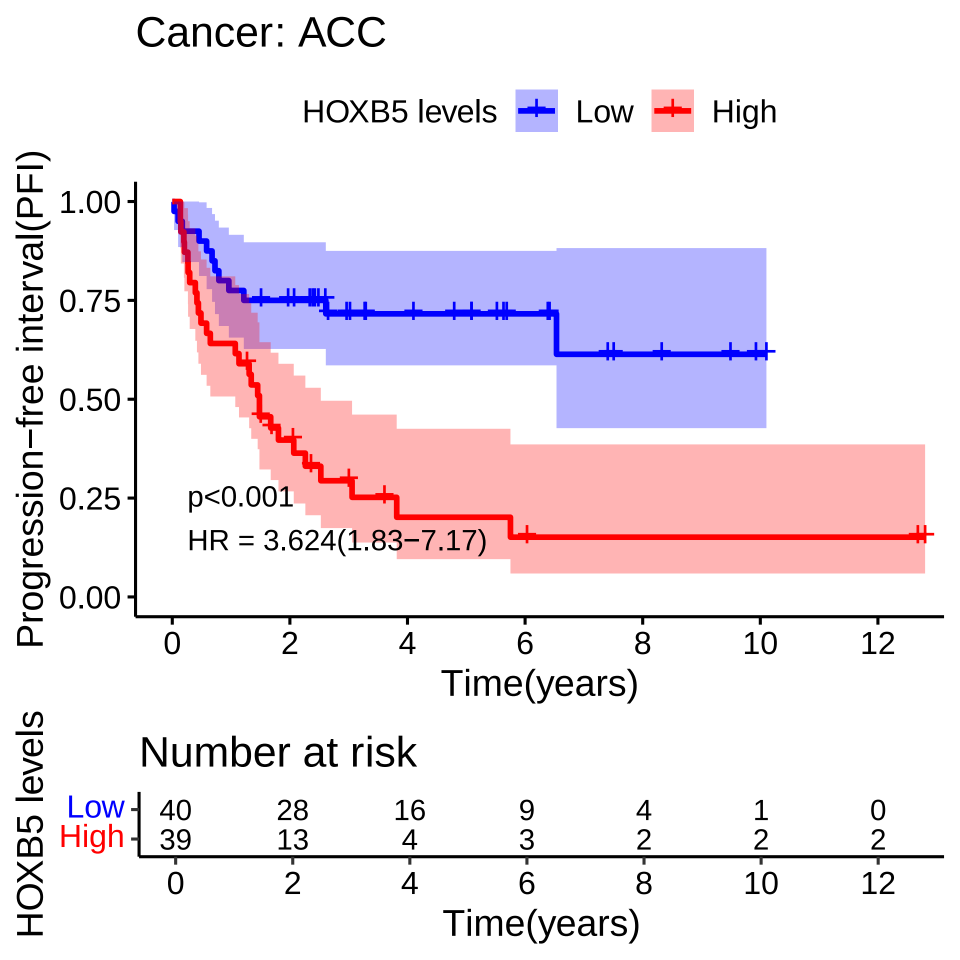

Cancer: ACC
HOXB5 levels
Low
High
Progression−free interval(PFI)
1.00
0.75
0.50
0.25
0.00
p<0.001
HR = 3.624(1.83−7.17)
0
2
4
6
8
10
12
Time(years)
Number at risk
HOXB5 levels
Low 40 28 16 9 4 1 0
High 39 13 4 3 2 2 2
Time(years)

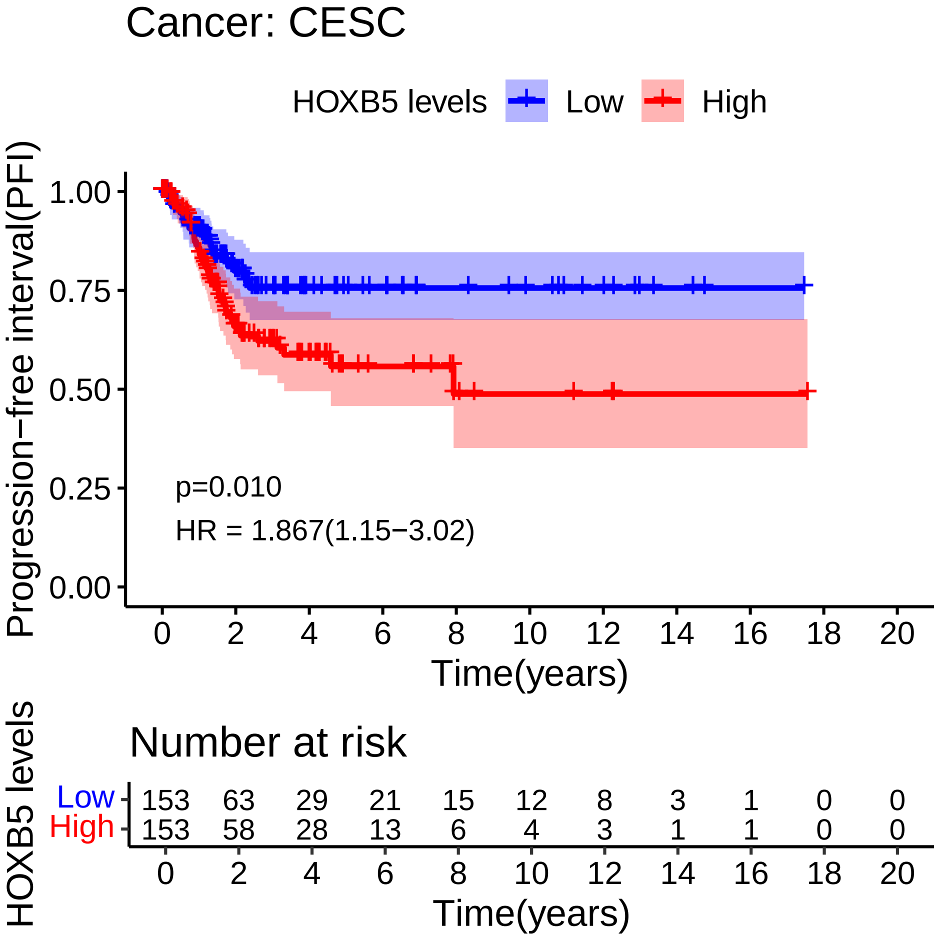

Cancer: CESC
HOXB5 levels
Low
High
Progression−free interval(PFI)
1.00
0.75
0.50
0.25
0.00
p=0.010
HR = 1.867(1.15−3.02)
0 2 4 6 8 10 12 14 16 18 20
Time(years)
Number at risk
HOXB5 levels
Low 153 63 29 21 15 12 8 3 1 0 0
High 153 58 28 13 6 4 3 1 1 0 0
0 2 4 6 8 10 12 14 16 18 20
Time(years)

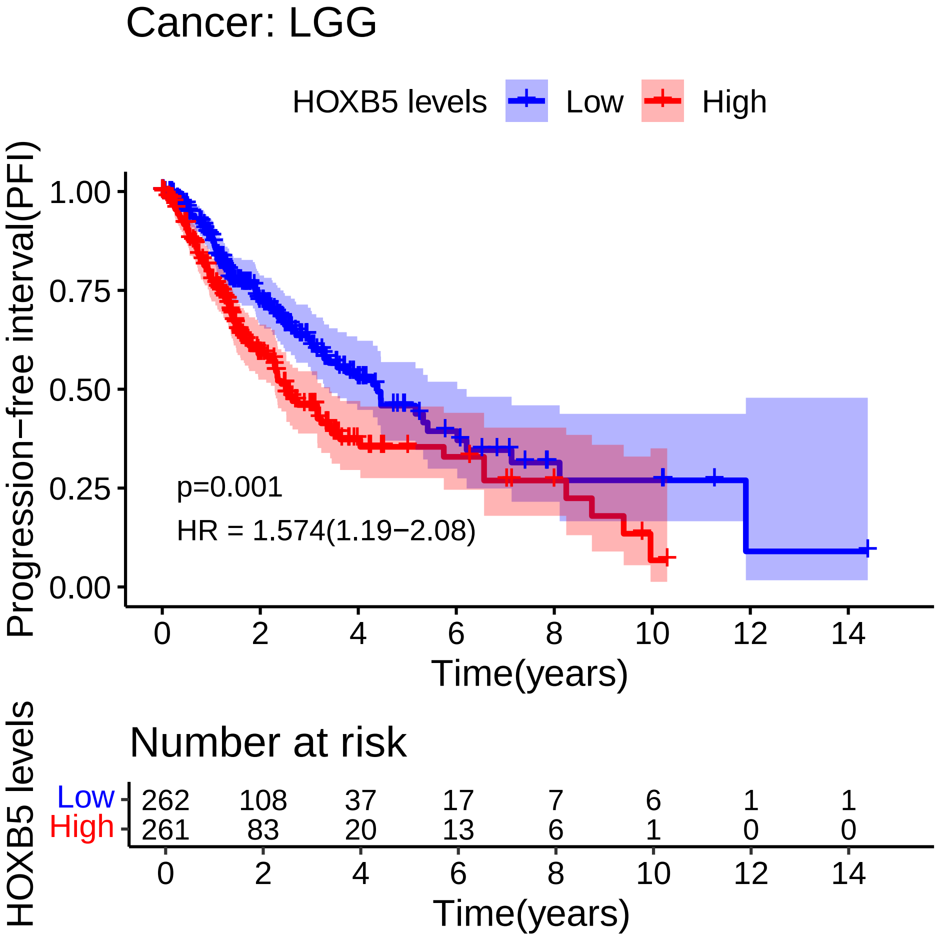

Cancer: LGG
HOXB5 levels
Low
High
Progression−free interval(PFI)
1.00
0.75
0.50
0.25
0.00
p=0.001
HR = 1.574(1.19−2.08)
0
2
4
6
8
10
12
14
Time(years)
Number at risk
HOXB5 levels
Low 262 108 37 17 7 6 1 1
High 261 83 20 13 6 1 0 0
Time(years)

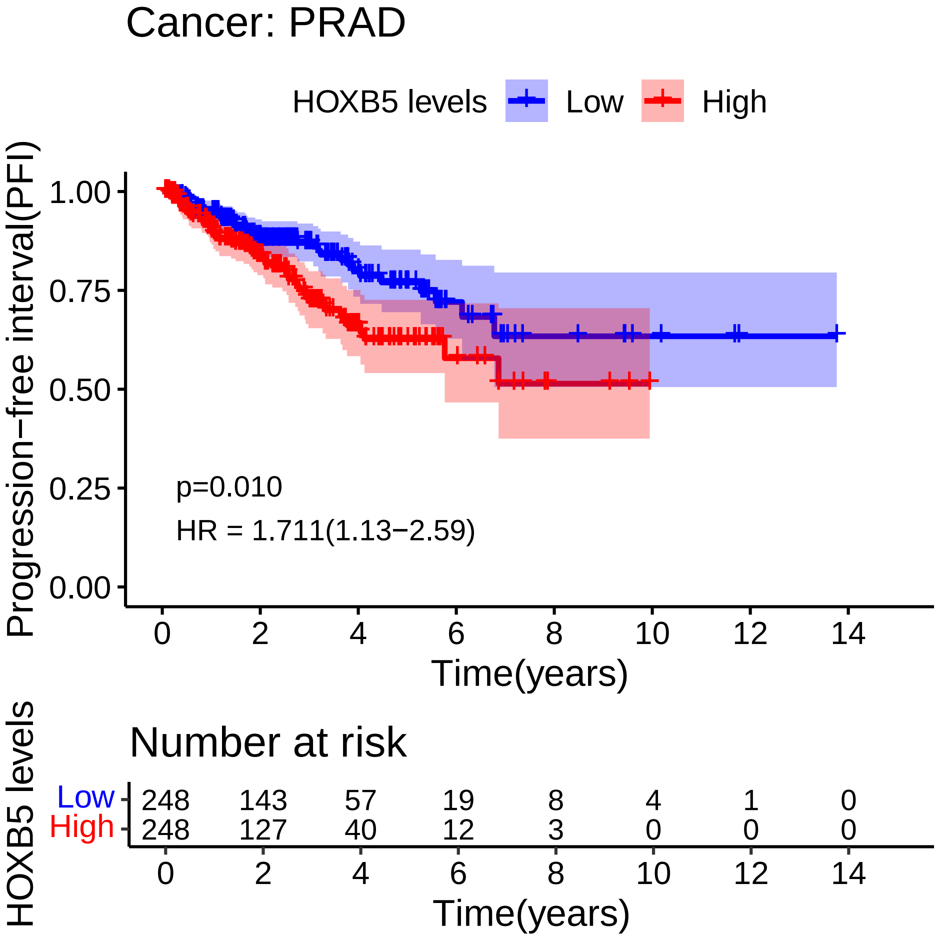

Cancer: PRAD
HOXB5 levels
Low
High
Progression−free interval(PFI)
1.00
0.75
0.50
0.25
0.00
p=0.010
HR = 1.711(1.13−2.59)
0
2
4
6
8
10
12
14
Time(years)
Number at risk
HOXB5 levels
Low 248 143 57 19 8 4 1 0
High 248 127 40 12 3 0 0 0
0
2
4
6
8
10
12
14
Time(years)

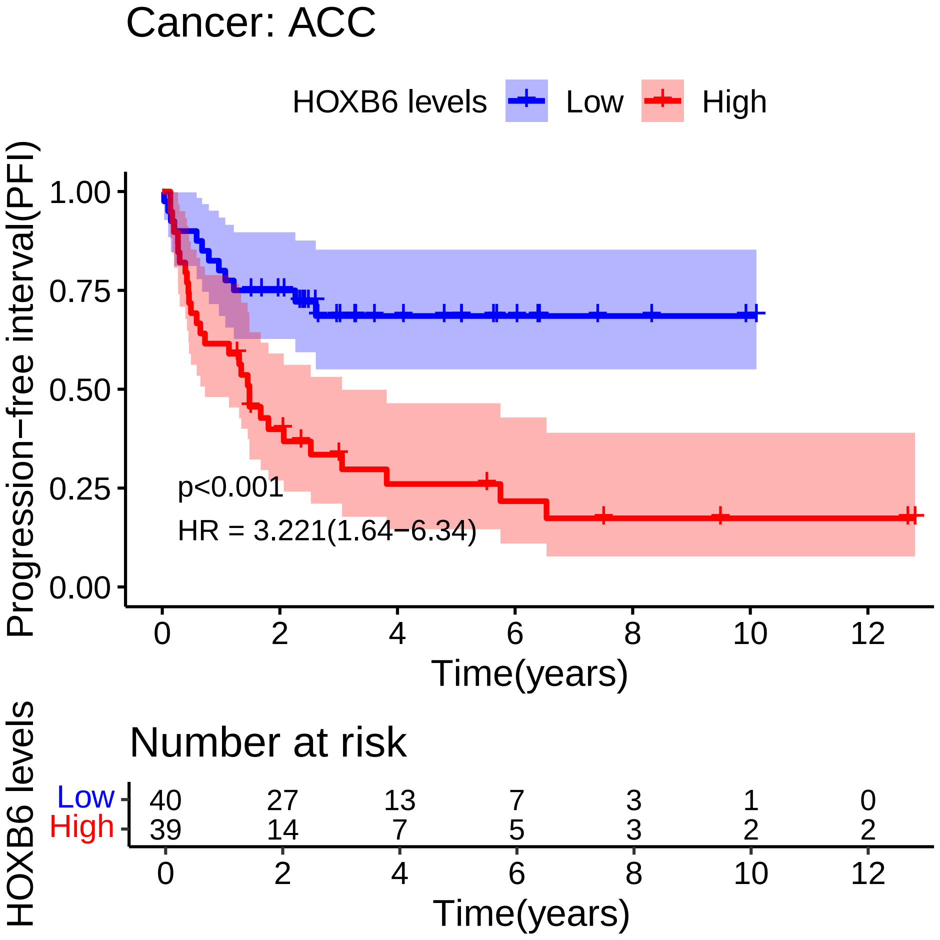
Cancer: ACC
HOXB6 levels
Low
High
Progression−free interval(PFI)
1.00
0.75
0.50
0.25
0.00
p<0.001
HR = 3.221(1.64−6.34)
0
2
4
6
8
10
12
Time(years)
Number at risk
HOXB6 levels
Low
High
40 27 13 7 3 1 0
39 14 7 5 3 2 2
Time(years)

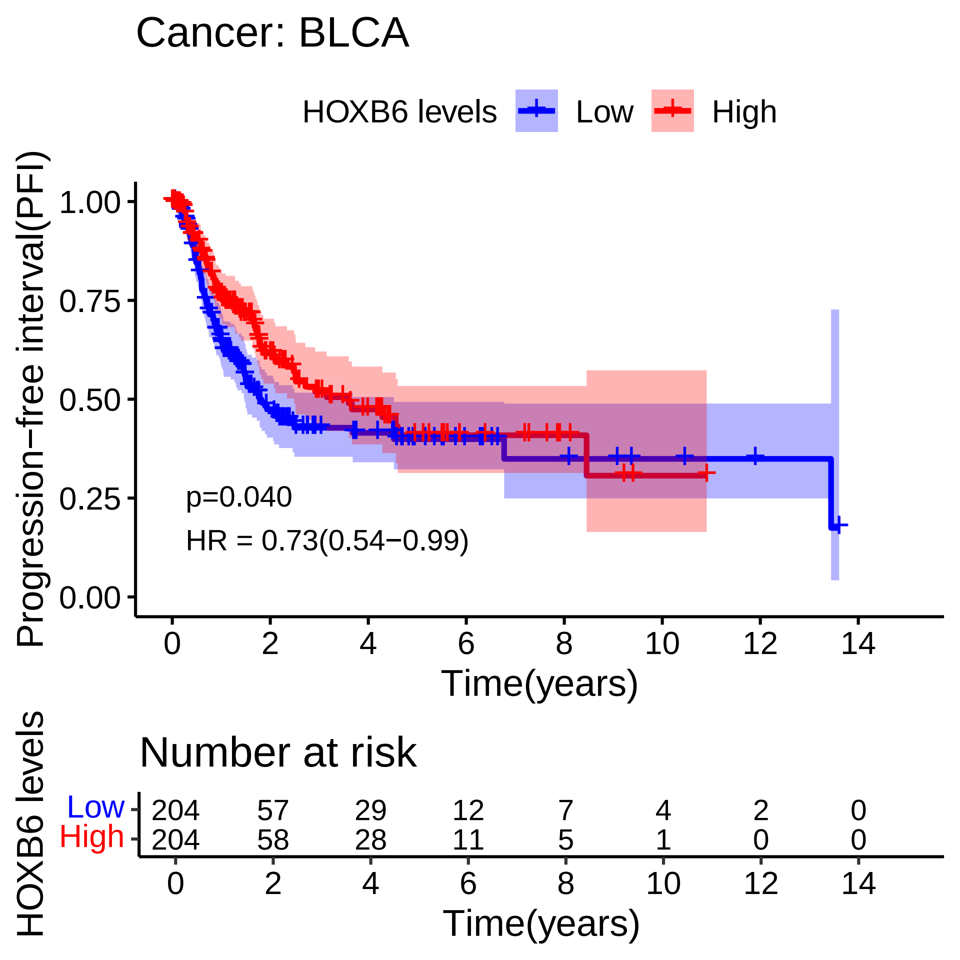
Cancer: BLCA
HOXB6 levels
Low
High
Progression−free interval(PFI)
1.00
0.75
0.50
0.25
0.00
p=0.040
HR = 0.73(0.54−0.99)
0
2
4
6
8
10
12
14
Time(years)
Number at risk
HOXB6 levels
Low
High
204 57 29 12 7 4 2 0
204 58 28 11 5 1 0 0
Time(years)

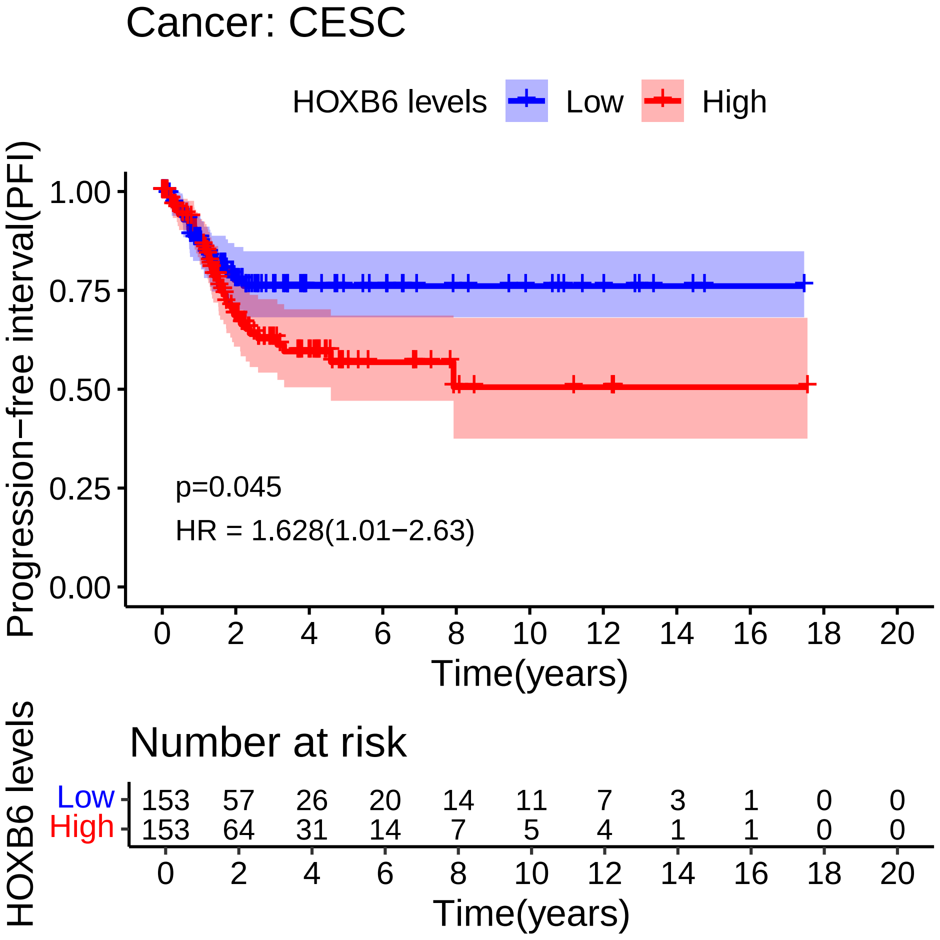

Cancer: CESC
HOXB6 levels
Low
High
Progression−free interval(PFI)
1.00
0.75
0.50
0.25
0.00
p=0.045
HR = 1.628(1.01−2.63)
0 2 4 6 8 10 12 14 16 18 20
Time(years)
Number at risk
HOXB6 levels
Low 153 57 26 20 14 11 7 3 1 0 0
High 153 64 31 14 7 5 4 1 1 0 0
0 2 4 6 8 10 12 14 16 18 20
Time(years)

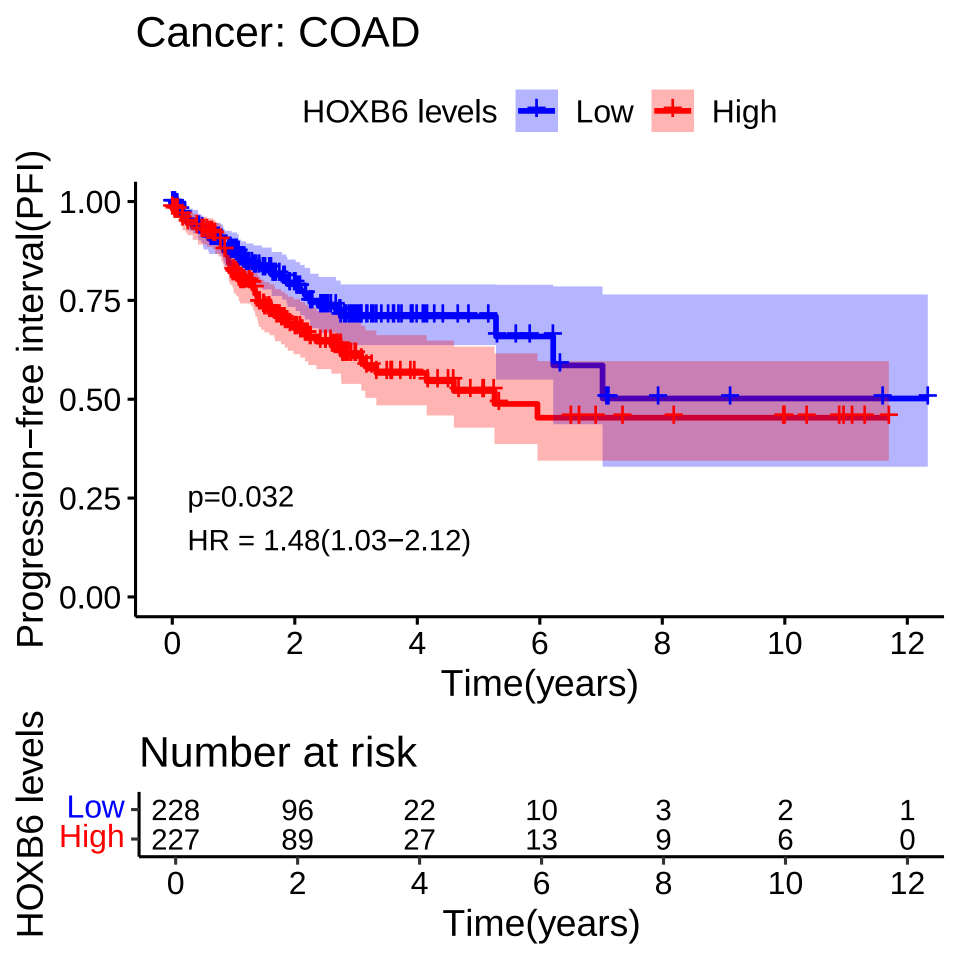

Cancer: COAD
HOXB6 levels
Low
High
Progression−free interval(PFI)
1.00
0.75
0.50
0.25
0.00
p=0.032
HR = 1.48(1.03−2.12)
0
2
4
6
8
10
12
Time(years)
Number at risk
HOXB6 levels
Low 228 96 22 10 3 2 1
High 227 89 27 13 9 6 0
Time(years)

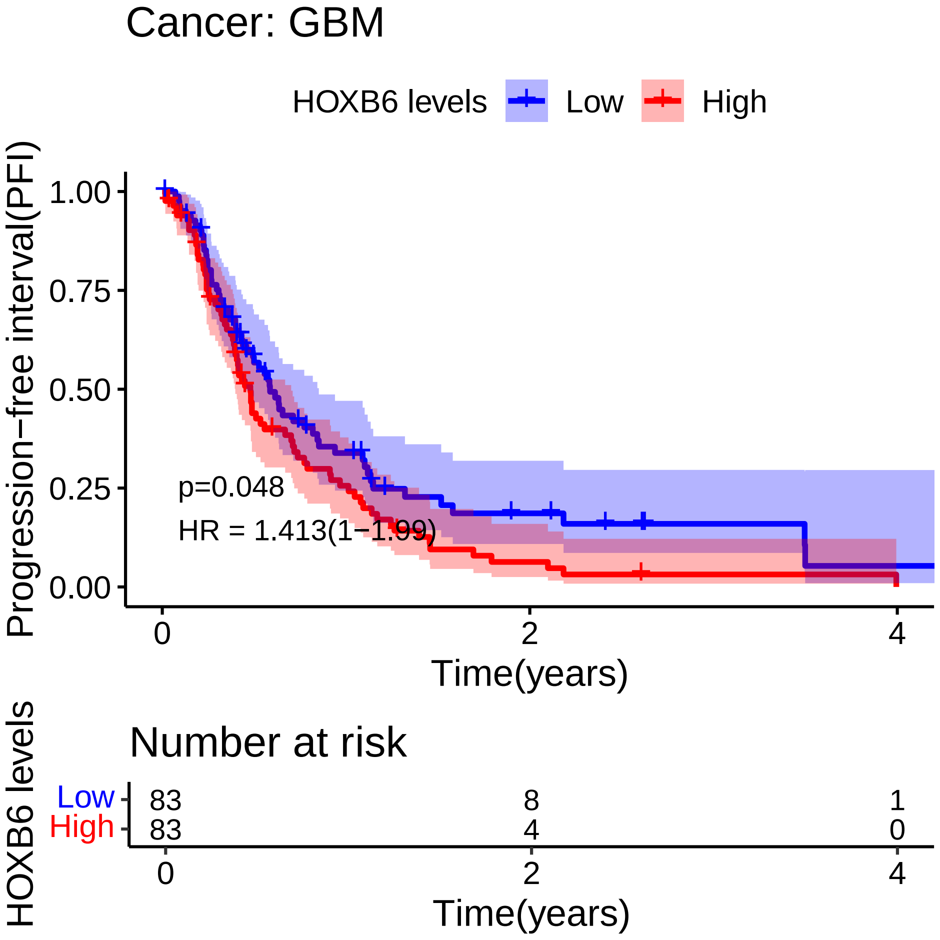

Cancer: GBM
HOXB6 levels
Low
High
Progression−free interval(PFI)
1.00
0.75
0.50
0.25
0.00
p=0.048
HR = 1.413(1−1.99)
0
2
4
Time(years)
Number at risk
HOXB6 levels
Low
High
83
8
1
83
4
0
0
2
4
Time(years)

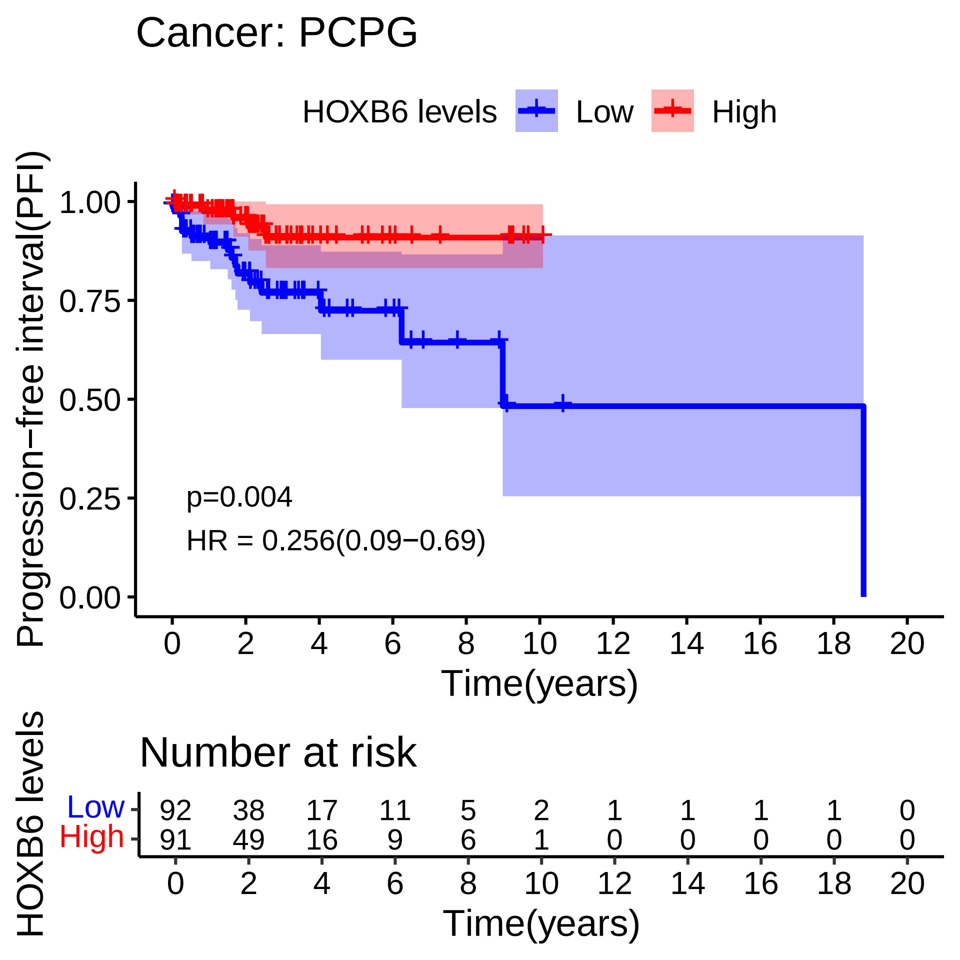

Cancer: PCPG
HOXB6 levels
Low
High
Progression−free interval(PFI)
1.00
0.75
0.50
0.25
0.00
p=0.004
HR = 0.256(0.09−0.69)
0 2 4 6 8 10 12 14 16 18 20
Time(years)
Number at risk
HOXB6 levels
Low 92 38 17 11 5 2 1 1 1 1 0
High 91 49 16 9 6 1 0 0 0 0 0
0 2 4 6 8 10 12 14 16 18 20
Time(years)

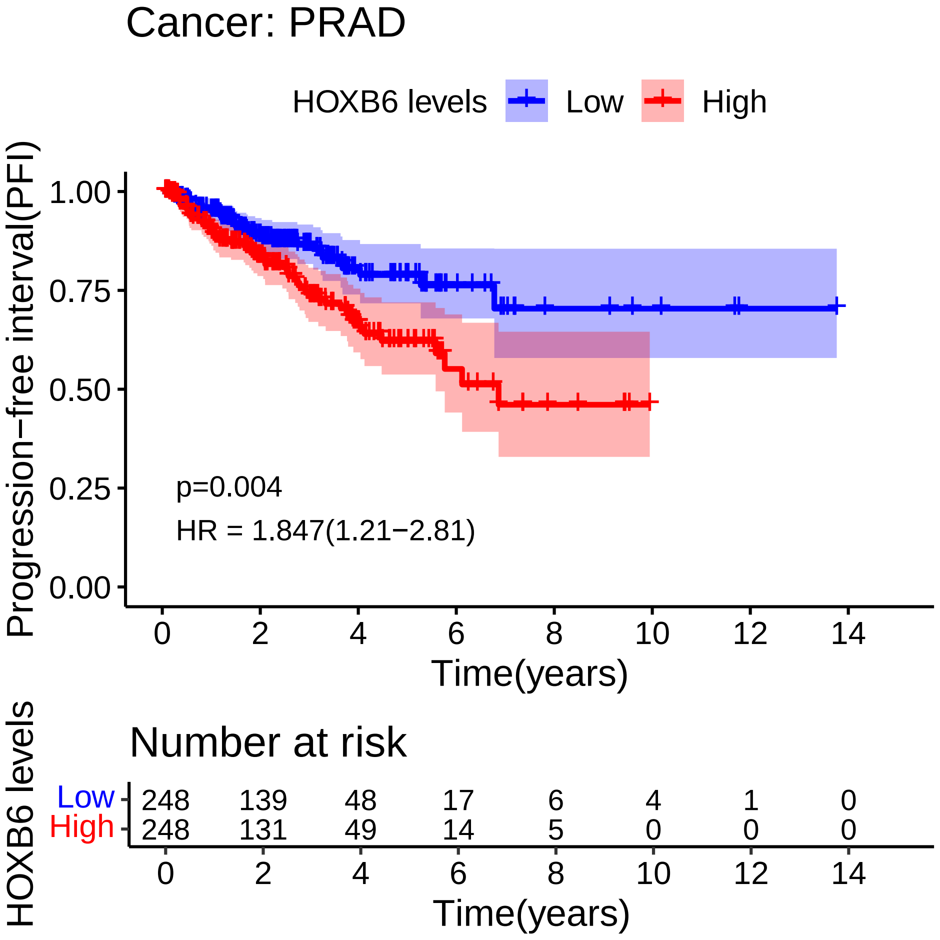

Cancer: PRAD
HOXB6 levels
Low
High
Progression−free interval(PFI)
1.00
0.75
0.50
0.25
0.00
p=0.004
HR = 1.847(1.21−2.81)
0
2
4
6
8
10
12
14
Time(years)
Number at risk
HOXB6 levels
Low 248 139 48 17 6 4 1 0
High 248 131 49 14 5 0 0 0
Time(years)

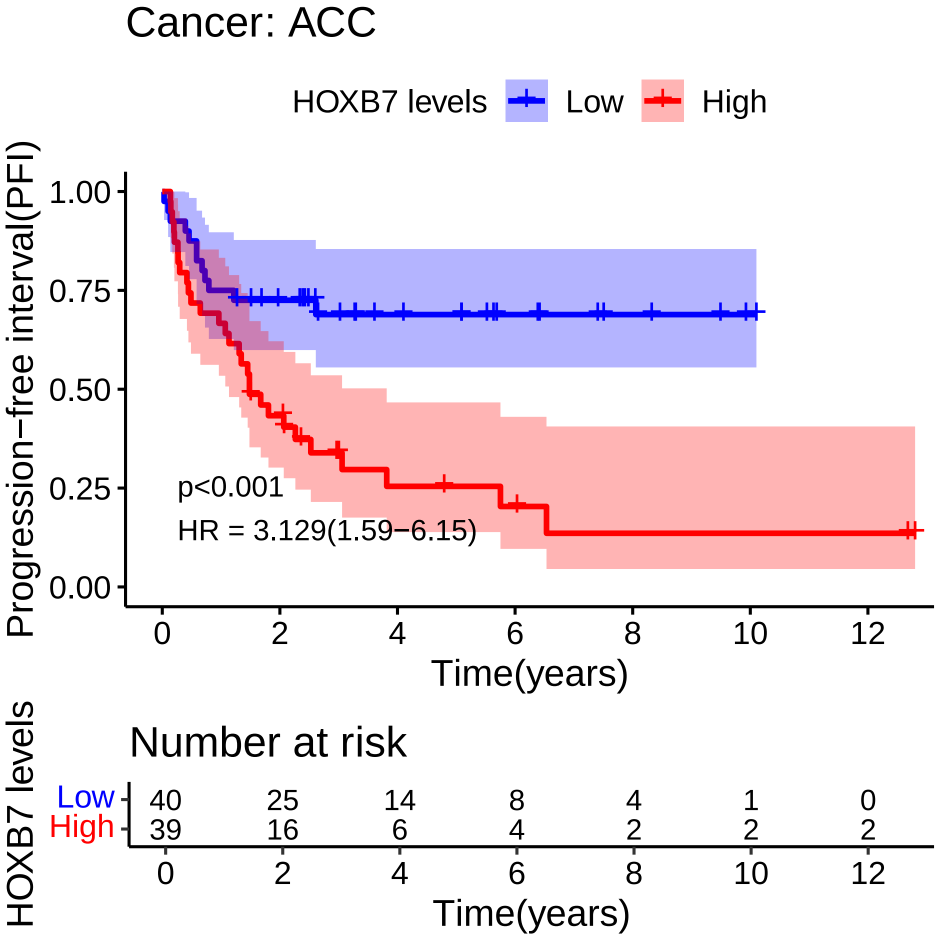

Cancer: ACC
HOXB7 levels
Low
High
Progression−free interval(PFI)
1.00
0.75
0.50
0.25
0.00
p<0.001
HR = 3.129(1.59−6.15)
0
2
4
6
8
10
12
Time(years)
Number at risk
HOXB7 levels
Low 40 25 14 8 4 1 0
High 39 16 6 4 2 2 2
Time(years)

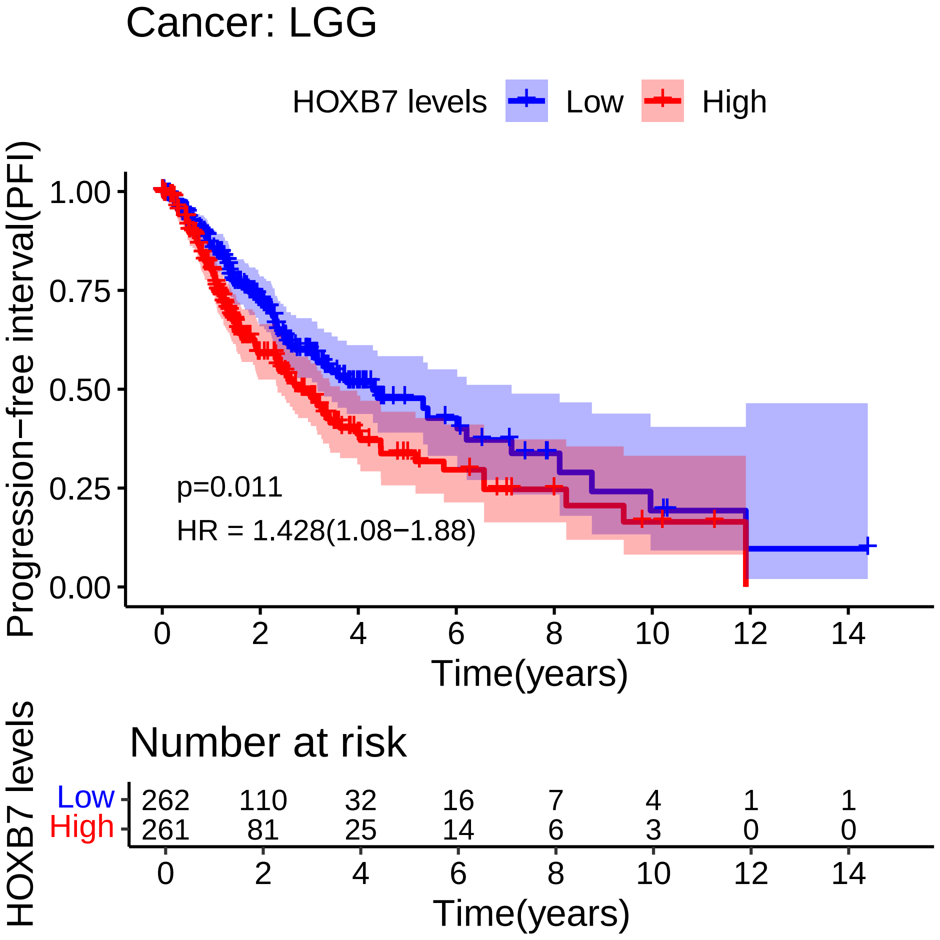
Cancer: LGG
HOXB7 levels
Low
High
Progression−free interval(PFI)
1.00
0.75
0.50
0.25
0.00
p=0.011
HR = 1.428(1.08−1.88)
0
2
4
6
8
10
12
14
Time(years)
Number at risk
HOXB7 levels
Low 262 110 32 16 7 4 1 1
High 261 81 25 14 6 3 0 0
0
2
4
6
8
10
12
14
Time(years)

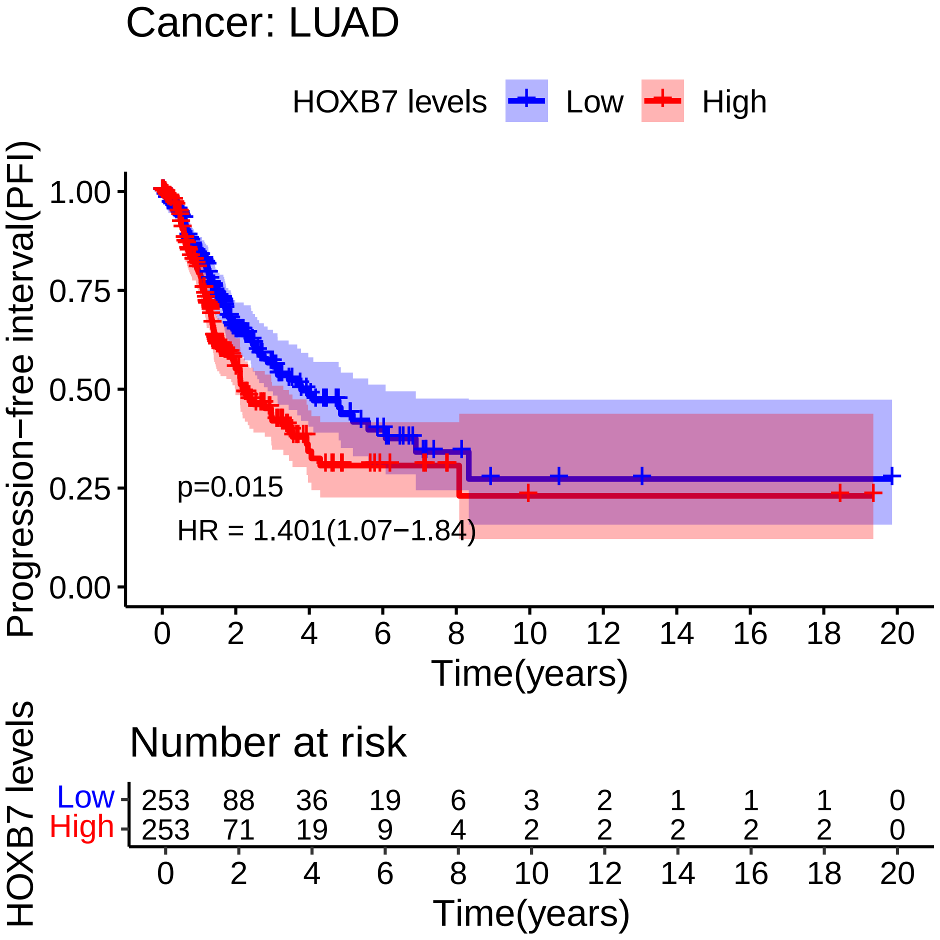

Cancer: LUAD
HOXB7 levels
Low
High
Progression−free interval(PFI)
1.00
0.75
0.50
0.25
0.00
p=0.015
HR = 1.401(1.07−1.84)
0
2
4
6
8
10
12
14
16
18
20
Time(years)
Number at risk
HOXB7 levels
Low 253 88 36 19 6 3 2 1 1 1 0
High 253 71 19 9 4 2 2 2 2 2 0
Time(years)

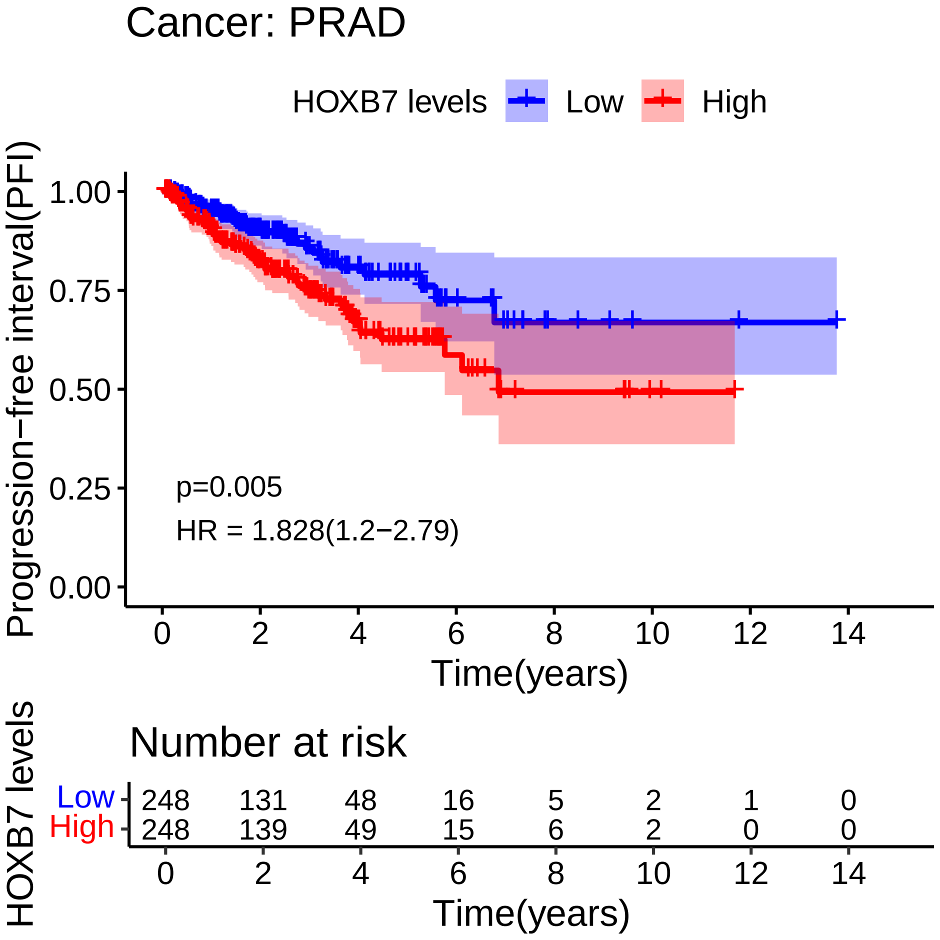
Cancer: PRAD
HOXB7 levels
Low
High
Progression−free interval(PFI)
1.00
0.75
0.50
0.25
0.00
p=0.005
HR = 1.828(1.2−2.79)
0
2
4
6
8
10
12
14
Time(years)
Number at risk
HOXB7 levels
Low 248 131 48 16 5 2 1 0
High 248 139 49 15 6 2 0 0
0
2
4
6
8
10
12
14
Time(years)

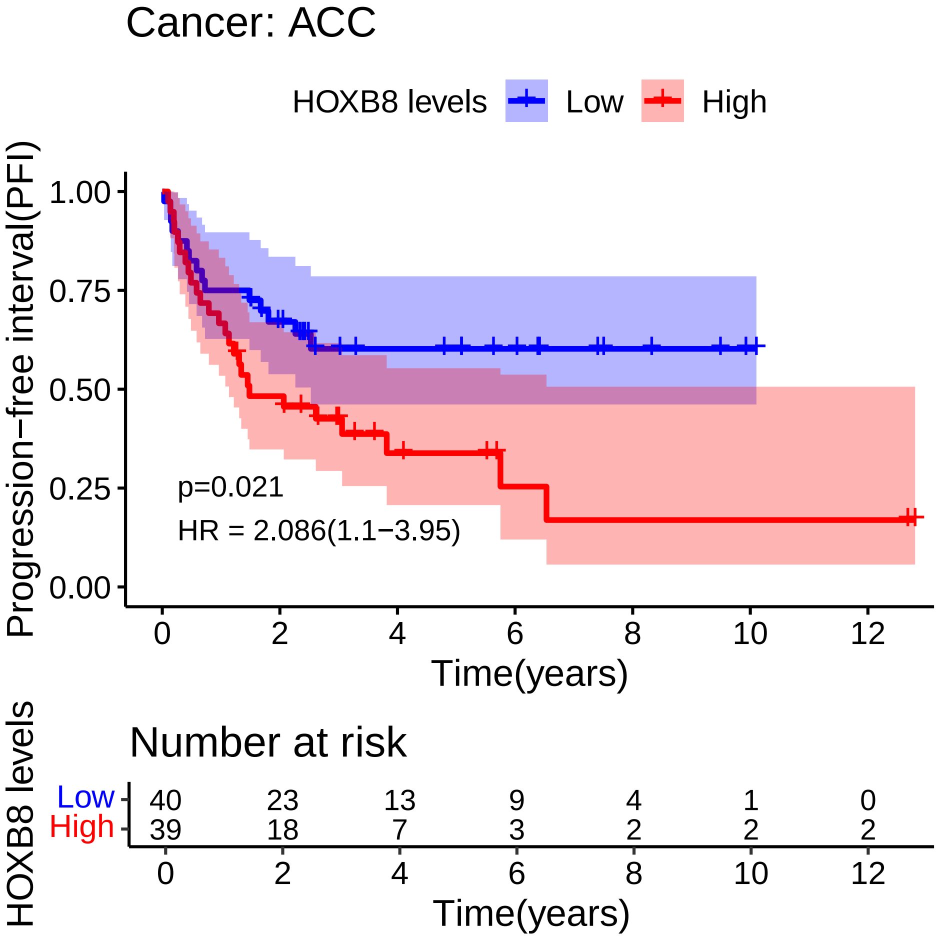

Cancer: ACC
HOXB8 levels
Low
High
Progression−free interval(PFI)
1.00
0.75
0.50
0.25
0.00
p=0.021
HR = 2.086(1.1−3.95)
0
2
4
6
8
10
12
Time(years)
Number at risk
HOXB8 levels
Low
High
40 23 13 9 4 1 0
39 18 7 3 2 2 2
Time(years)

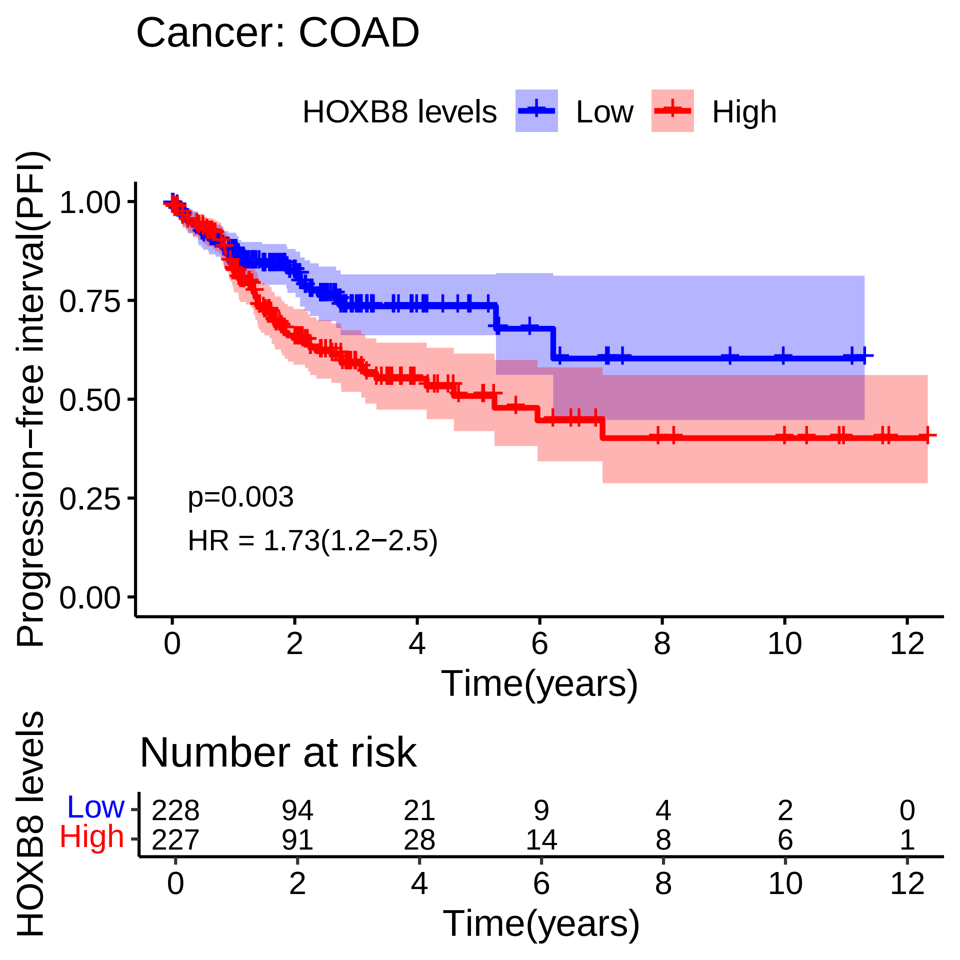

Cancer: COAD
HOXB8 levels
Low
High
Progression−free interval(PFI)
1.00
0.75
0.50
0.25
0.00
p=0.003
HR = 1.73(1.2−2.5)
0
2
4
6
8
10
12
Time(years)
Number at risk
HOXB8 levels
Low 228 94 21 9 4 2 0
High 227 91 28 14 8 6 1
Time(years)

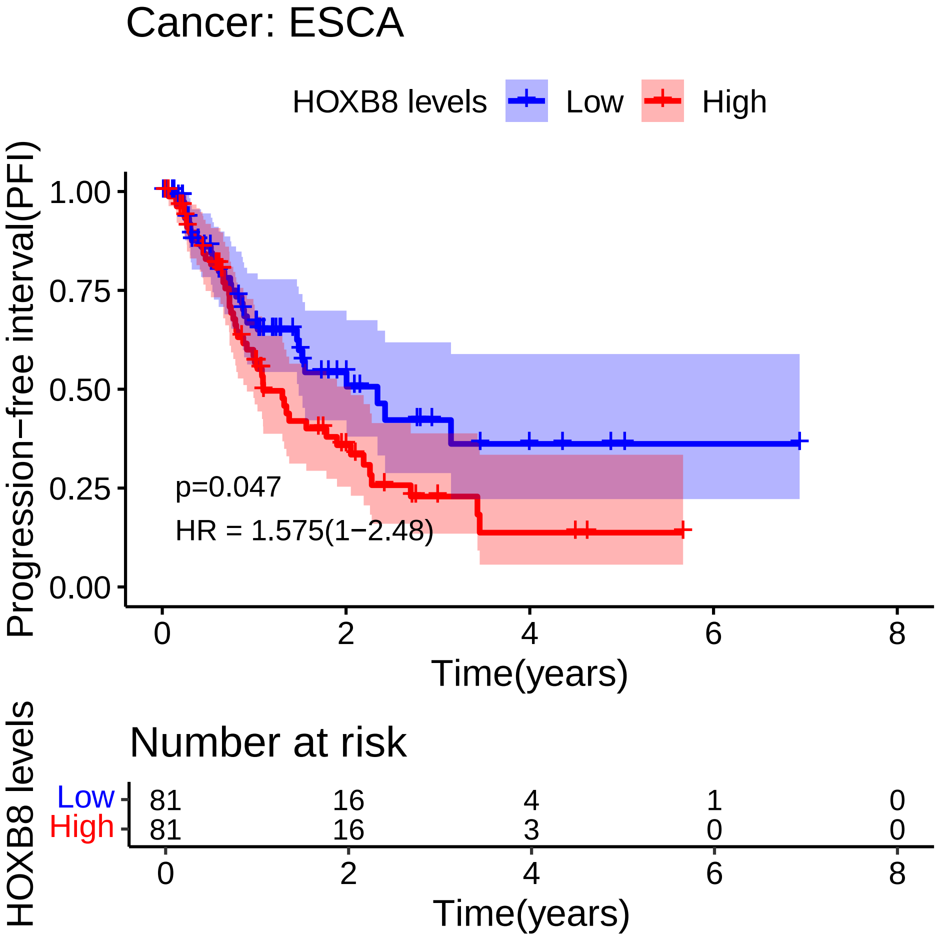

Cancer: ESCA
HOXB8 levels
Low
High
Progression−free interval(PFI)
1.00
0.75
0.50
0.25
0.00
p=0.047
HR = 1.575(1−2.48)
0
2
4
6
8
Time(years)
Number at risk
HOXB8 levels
Low 81 16 4 1 0
High 81 16 3 0 0
Time(years)

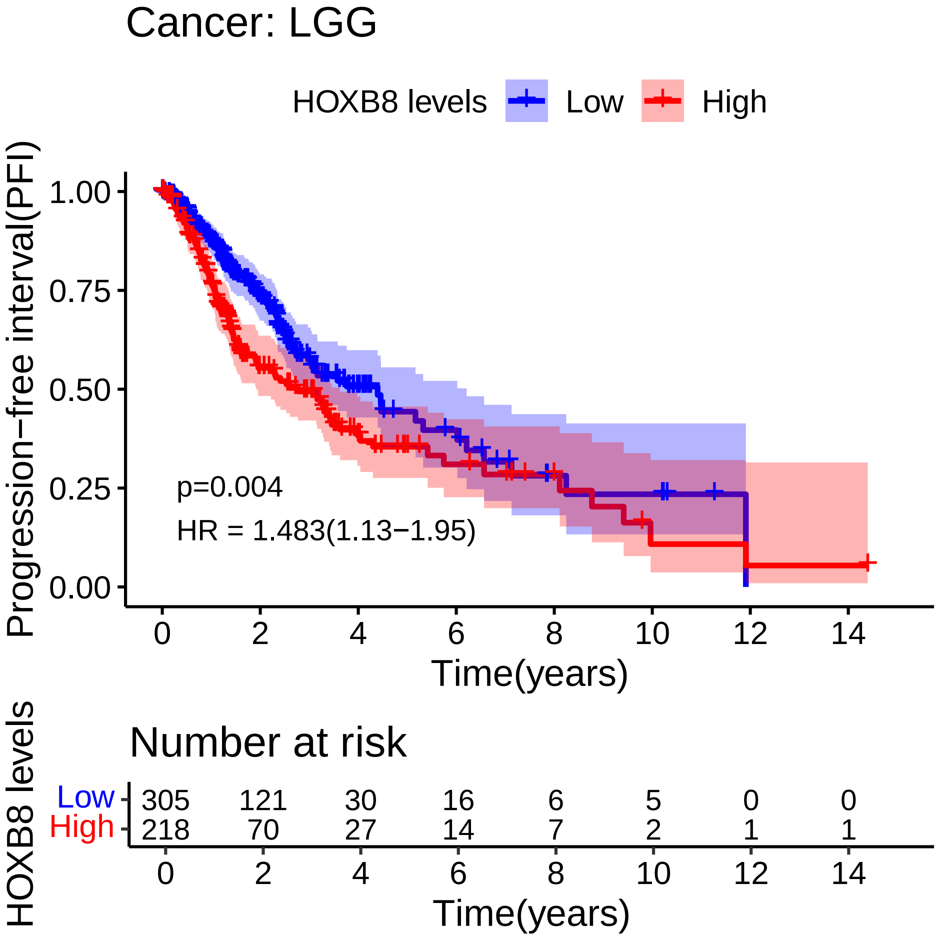

Cancer: LGG
HOXB8 levels
Low
High
Progression−free interval(PFI)
1.00
0.75
0.50
0.25
0.00
p=0.004
HR = 1.483(1.13−1.95)
0
2
4
6
8
10
12
14
Time(years)
Number at risk
HOXB8 levels
Low 305 121 30 16 6 5 0 0
High 218 70 27 14 7 2 1 1
Time(years)

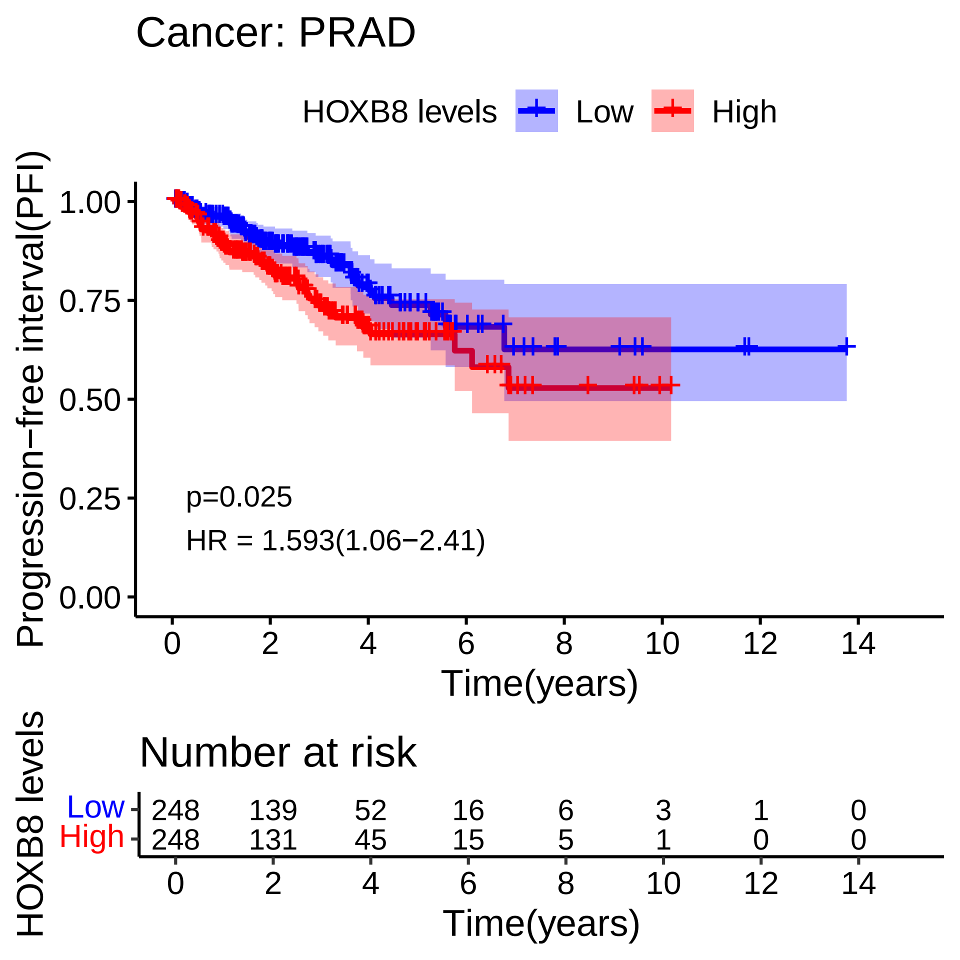

Cancer: PRAD
HOXB8 levels
Low
High
Progression−free interval(PFI)
1.00
0.75
0.50
0.25
0.00
p=0.025
HR = 1.593(1.06−2.41)
0
2
4
6
8
10
12
14
Time(years)
Number at risk
HOXB8 levels
Low 248 139 52 16 6 3 1 0
High 248 131 45 15 5 1 0 0
Time(years)

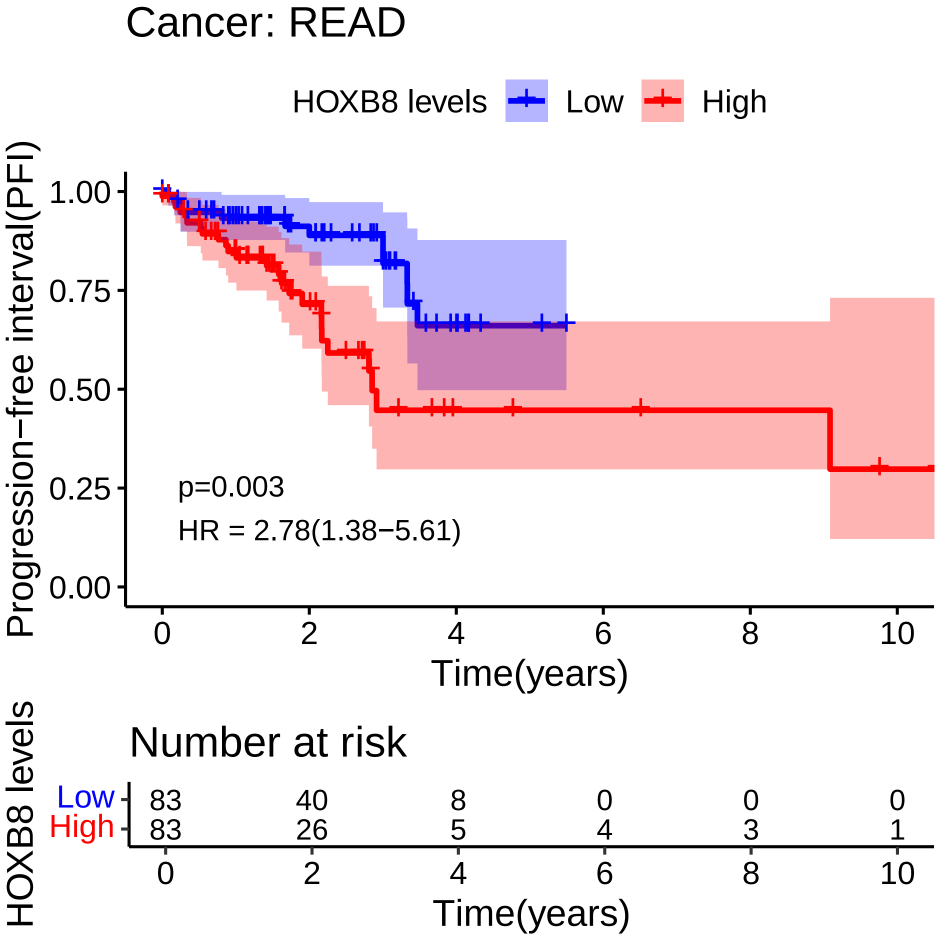

Cancer: READ
HOXB8 levels
Low
High
Progression−free interval(PFI)
1.00
0.75
0.50
0.25
0.00
p=0.003
HR = 2.78(1.38−5.61)
0
2
4
6
8
10
Time(years)
Number at risk
HOXB8 levels
Low 83 40 8 0 0 0
High 83 26 5 4 3 1
Time(years)

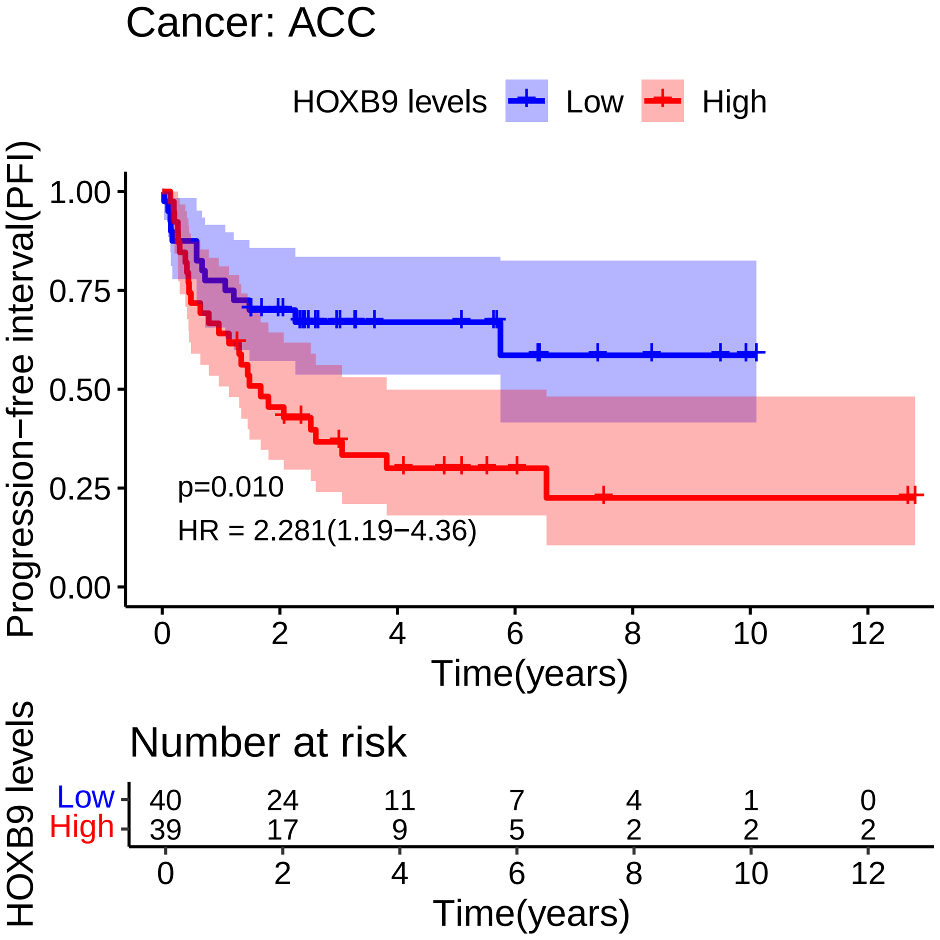

Cancer: ACC
HOXB9 levels
Low
High
Progression−free interval(PFI)
1.00
0.75
0.50
0.25
0.00
p=0.010
HR = 2.281(1.19−4.36)
0
2
4
6
8
10
12
Time(years)
Number at risk
HOXB9 levels
Low 40 24 11 7 4 1 0
High 39 17 9 5 2 2 2
Time(years)

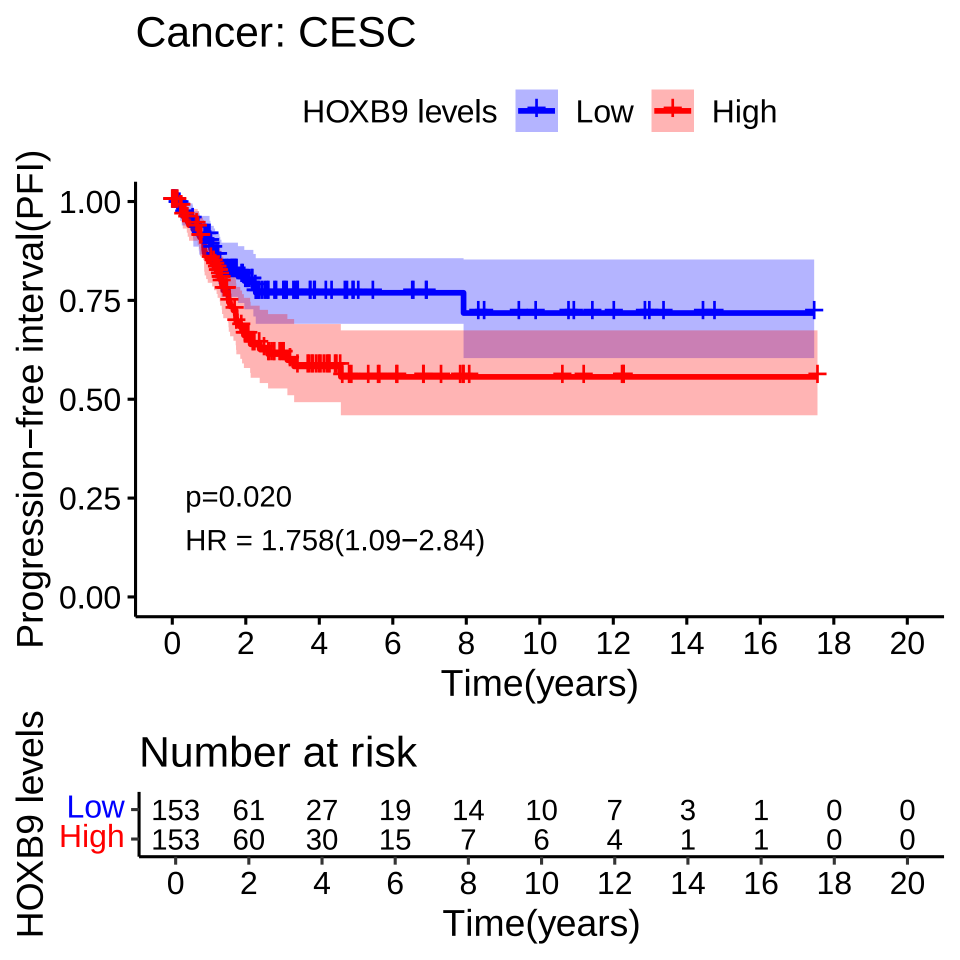
Cancer: CESC
HOXB9 levels
Low
High
Progression−free interval(PFI)
1.00
0.75
0.50
0.25
0.00
p=0.020
HR = 1.758(1.09−2.84)
0 2 4 6 8 10 12 14 16 18 20
Time(years)
Number at risk
HOXB9 levels
Low 153 61 27 19 14 10 7 3 1 0 0
High 153 60 30 15 7 6 4 1 1 0 0
0 2 4 6 8 10 12 14 16 18 20
Time(years)

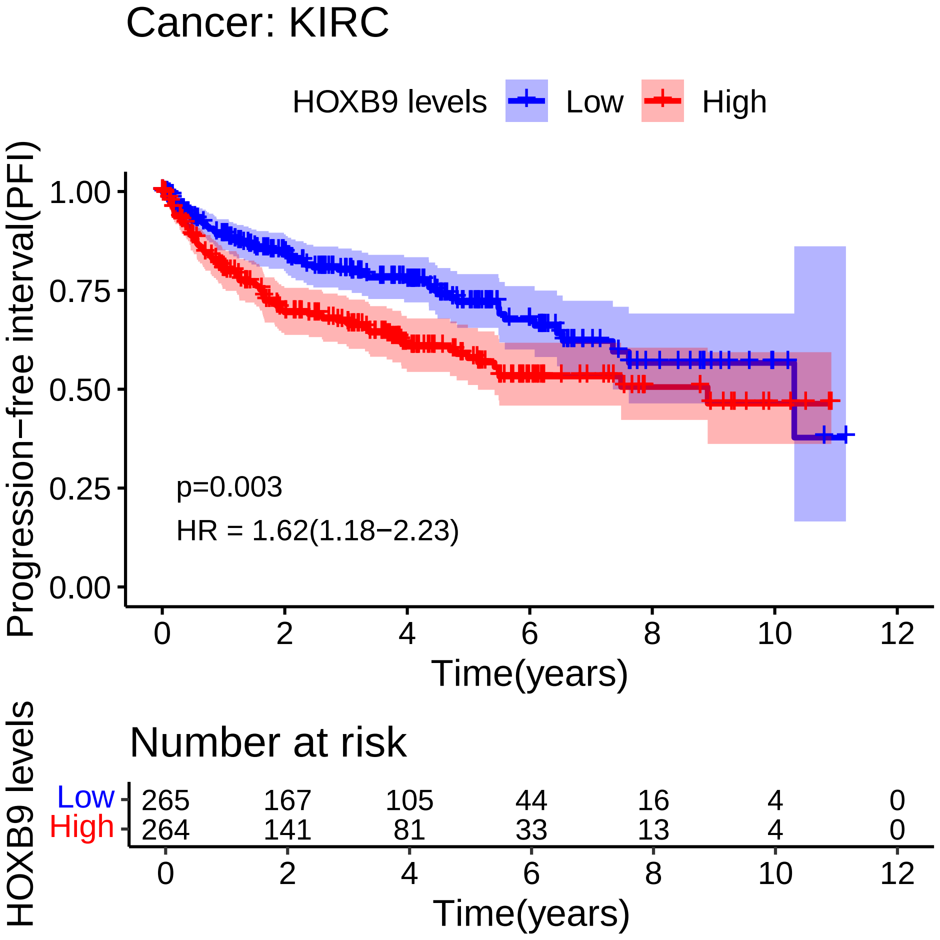
Cancer: KIRC
HOXB9 levels
Low
High
Progression−free interval(PFI)
1.00
0.75
0.50
0.25
0.00
p=0.003
HR = 1.62(1.18−2.23)
0
2
4
6
8
10
12
Time(years)
Number at risk
HOXB9 levels
Low
High
265 167 105 44 16 4 0
264 141 81 33 13 4 0
Time(years)

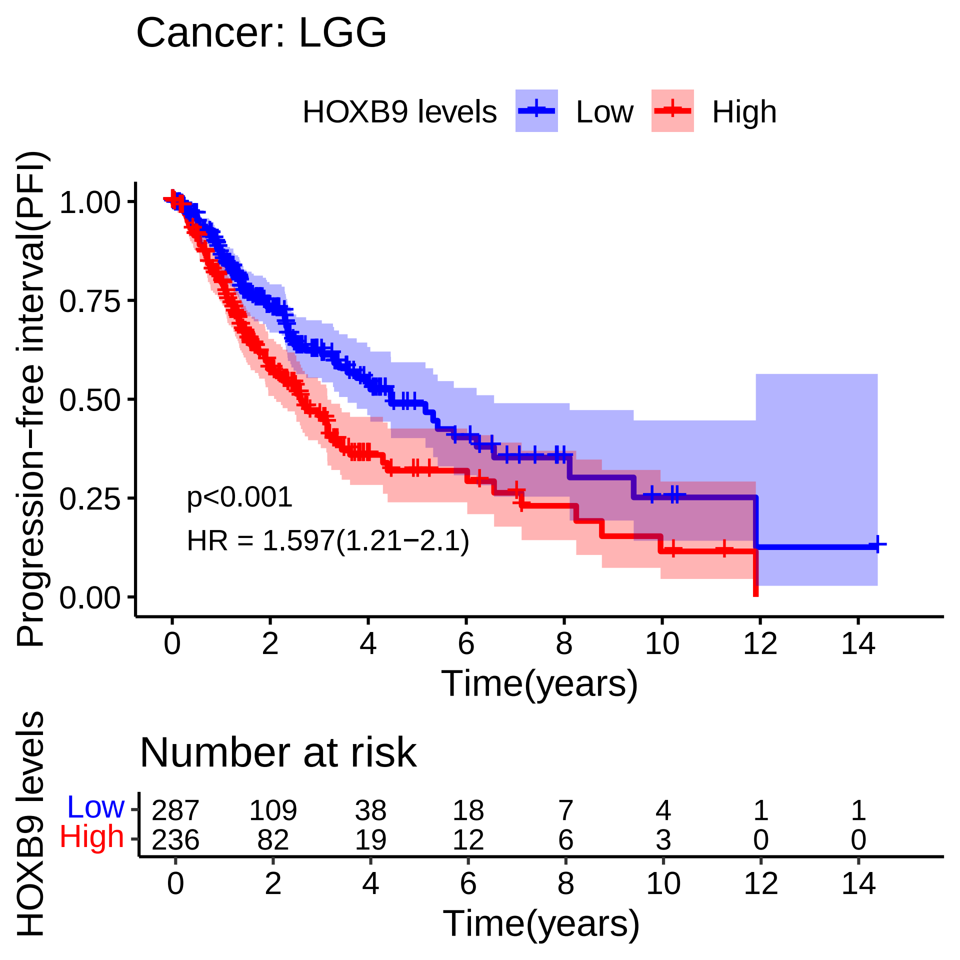
Cancer: LGG
HOXB9 levels
Low
High
Progression−free interval(PFI)
1.00
0.75
0.50
0.25
0.00
p<0.001
HR = 1.597(1.21−2.1)
0
2
4
6
8
10
12
14
Time(years)
Number at risk
HOXB9 levels
Low 287 109 38 18 7 4 1 1
High 236 82 19 12 6 3 0 0
Time(years)

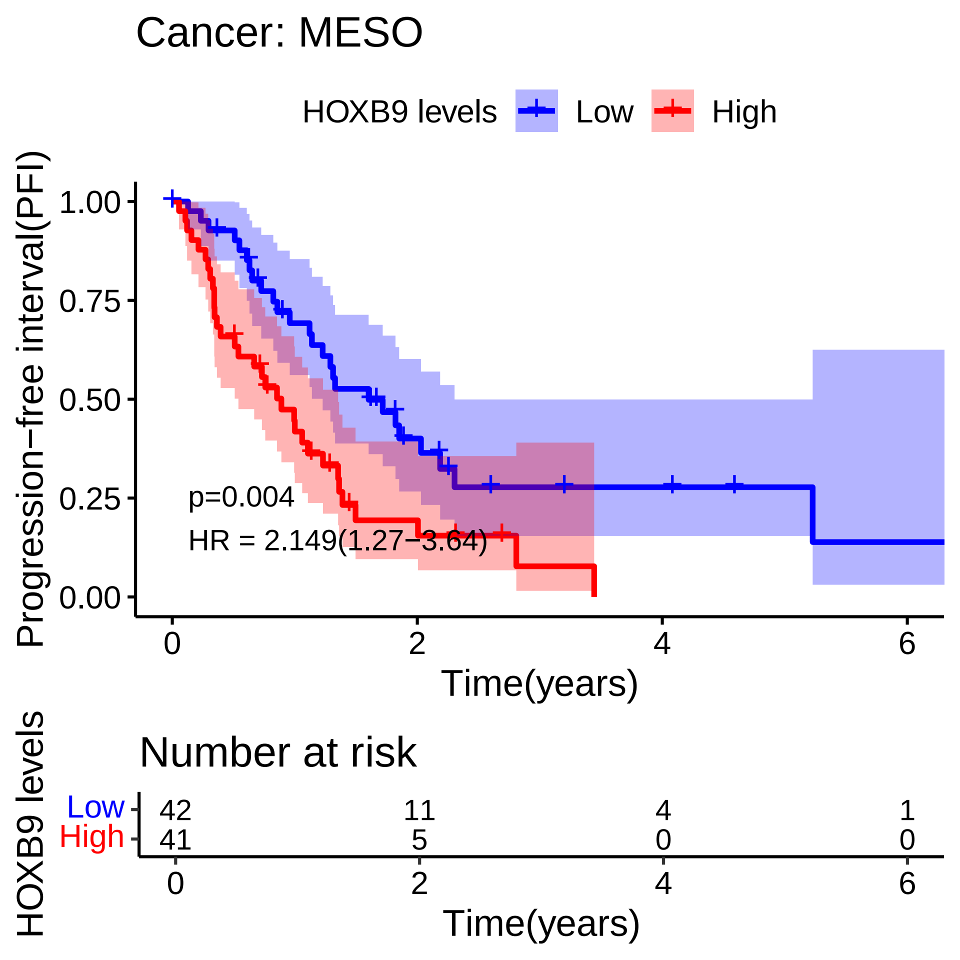
Cancer: MESO
HOXB9 levels
Low
High
Progression−free interval(PFI)
1.00
0.75
0.50
0.25
0.00
p=0.004
HR = 2.149(1.27−3.64)
0
2
4
6
Time(years)
Number at risk
HOXB9 levels
Low 42 11 4 1
High 41 5 0 0
0
2
4
6
Time(years)

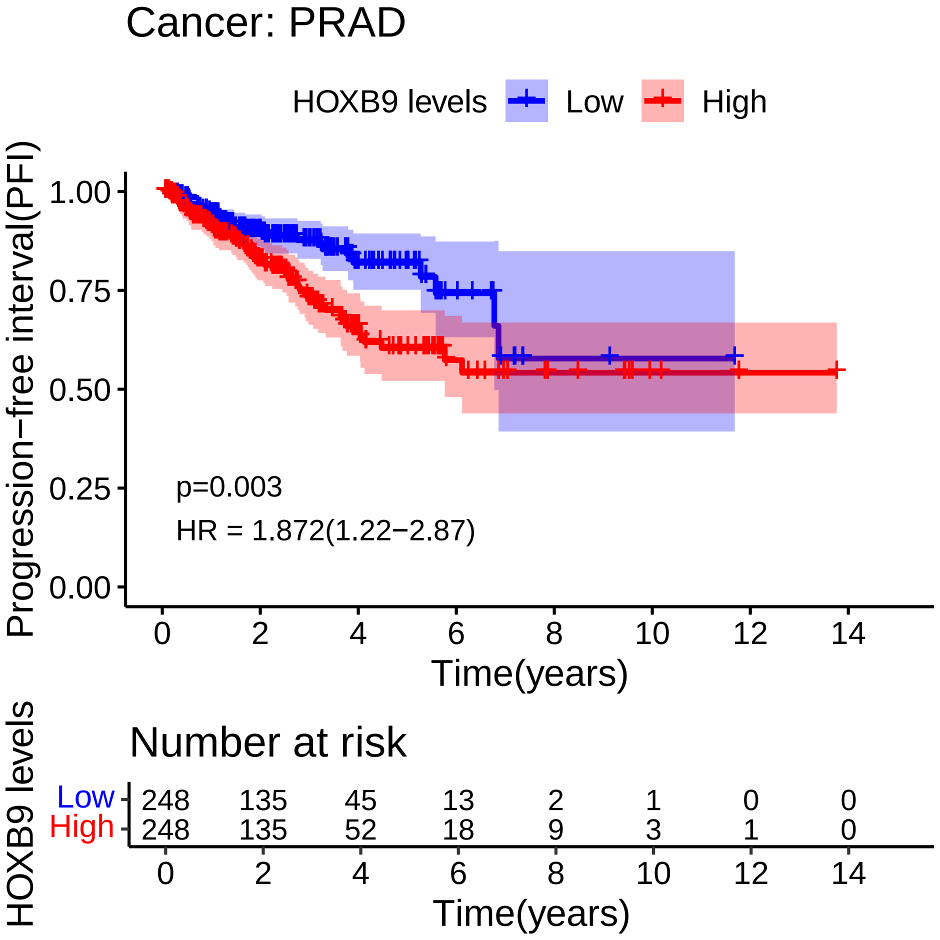

Cancer: PRAD
HOXB9 levels
Low
High
Progression−free interval(PFI)
1.00
0.75
0.50
0.25
0.00
p=0.003
HR = 1.872(1.22−2.87)
0
2
4
6
8
10
12
14
Time(years)
Number at risk
HOXB9 levels
Low 248 135 45 13 2 1 0 0
High 248 135 52 18 9 3 1 0
Time(years)

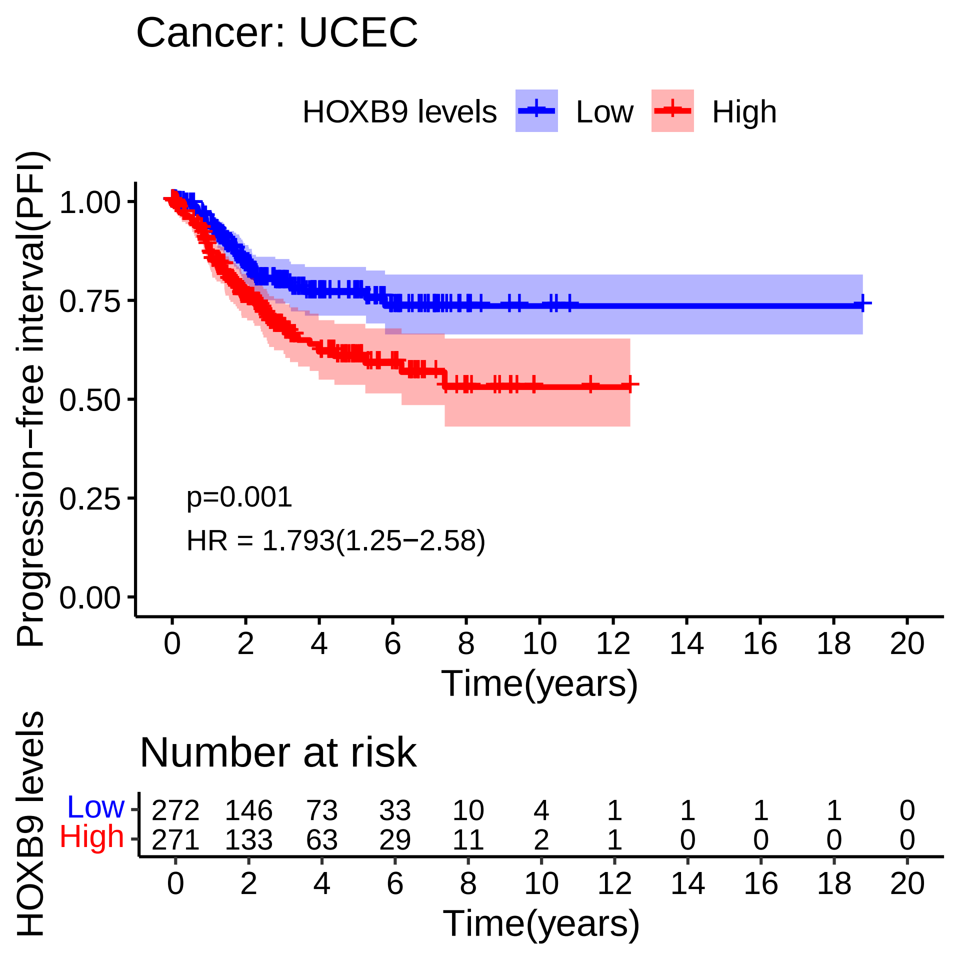
Cancer: UCEC
HOXB9 levels
Low
High
Progression−free interval(PFI)
1.00
0.75
0.50
0.25
0.00
p=0.001
HR = 1.793(1.25−2.58)
0 2 4 6 8 10 12 14 16 18 20
Time(years)
Number at risk
HOXB9 levels
Low 272 146 73 33 10 4 1 1 1 1 0
High 271 133 63 29 11 2 1 0 0 0 0
0 2 4 6 8 10 12 14 16 18 20
Time(years)

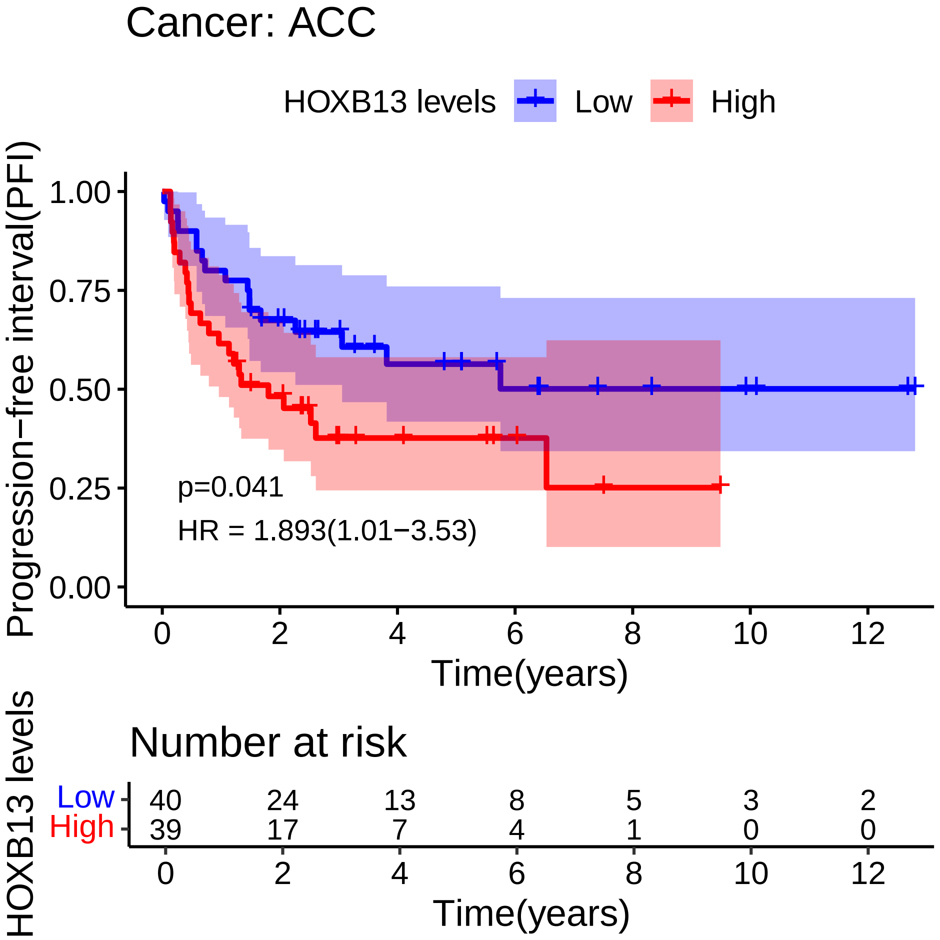

Cancer: ACC
HOXB13 levels
Low
High
Progression−free interval(PFI)
1.00
0.75
0.50
0.25
0.00
p=0.041
HR = 1.893(1.01−3.53)
0
2
4
6
8
10
12
Time(years)
Number at risk
HOXB13 levels
Low 40 24 13 8 5 3 2
High 39 17 7 4 1 0 0
0
2
4
6
8
10
12
Time(years)

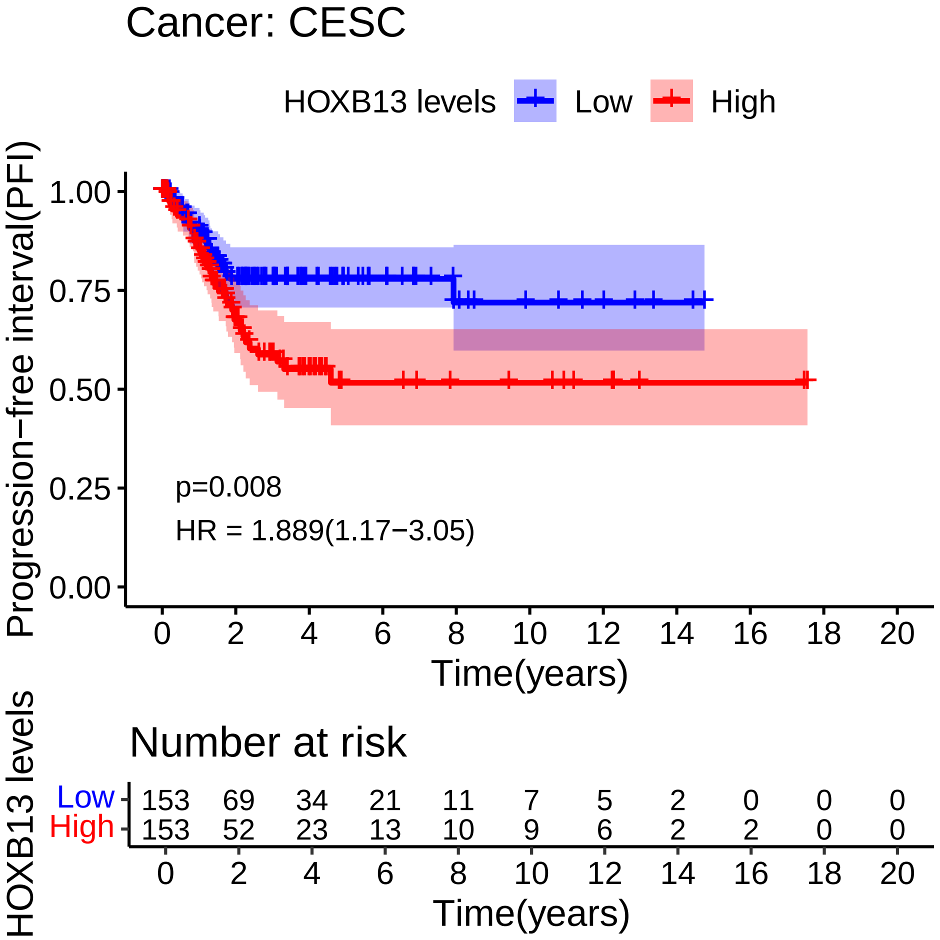

Cancer: CESC
HOXB13 levels
Low
High
Progression−free interval(PFI)
1.00
0.75
0.50
0.25
0.00
p=0.008
HR = 1.889(1.17−3.05)
0
2
4
6
8
10
12
14
16
18
20
Time(years)
Number at risk
HOXB13 levels
Low
High
153 69 34 21 11 7 5 2 0 0 0
153 52 23 13 10 9 6 2 2 0 0
Time(years)

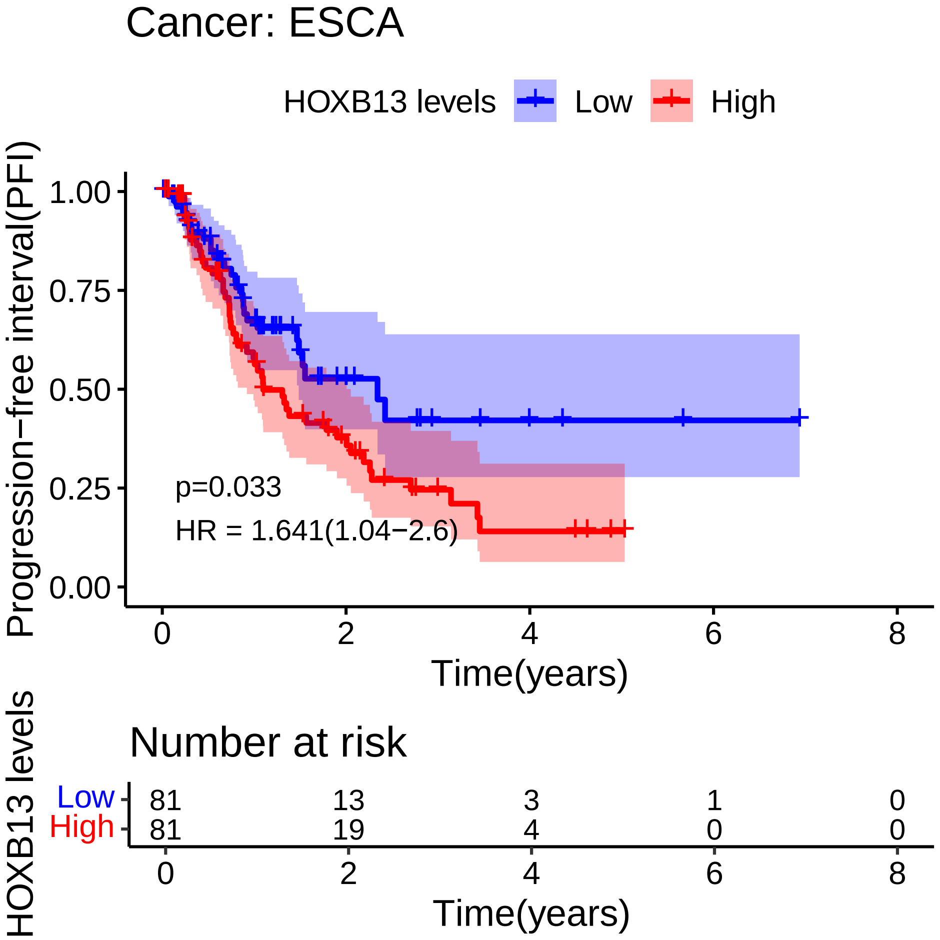
Cancer: ESCA
HOXB13 levels
Low
High
Progression−free interval(PFI)
1.00
0.75
0.50
0.25
0.00
p=0.033
HR = 1.641(1.04−2.6)
0
2
4
6
8
Time(years)
Number at risk
HOXB13 levels
Low 81 13 3 1 0
High 81 19 4 0 0
0
2
4
6
8
Time(years)

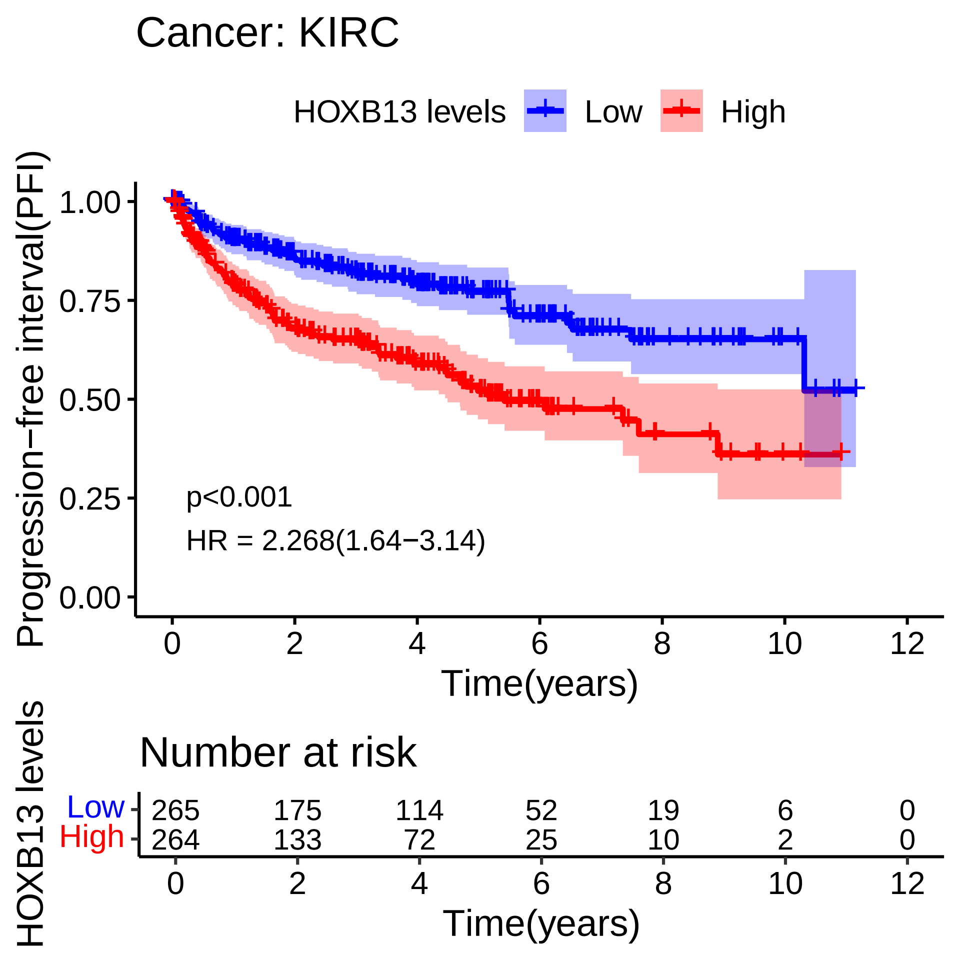

Cancer: KIRC
HOXB13 levels
Low
High
Progression−free interval(PFI)
1.00
0.75
0.50
0.25
0.00
p<0.001
HR = 2.268(1.64−3.14)
0
2
4
6
8
10
12
Time(years)
Number at risk
HOXB13 levels
Low 265 175 114 52 19 6 0
High 264 133 72 25 10 2 0
Time(years)

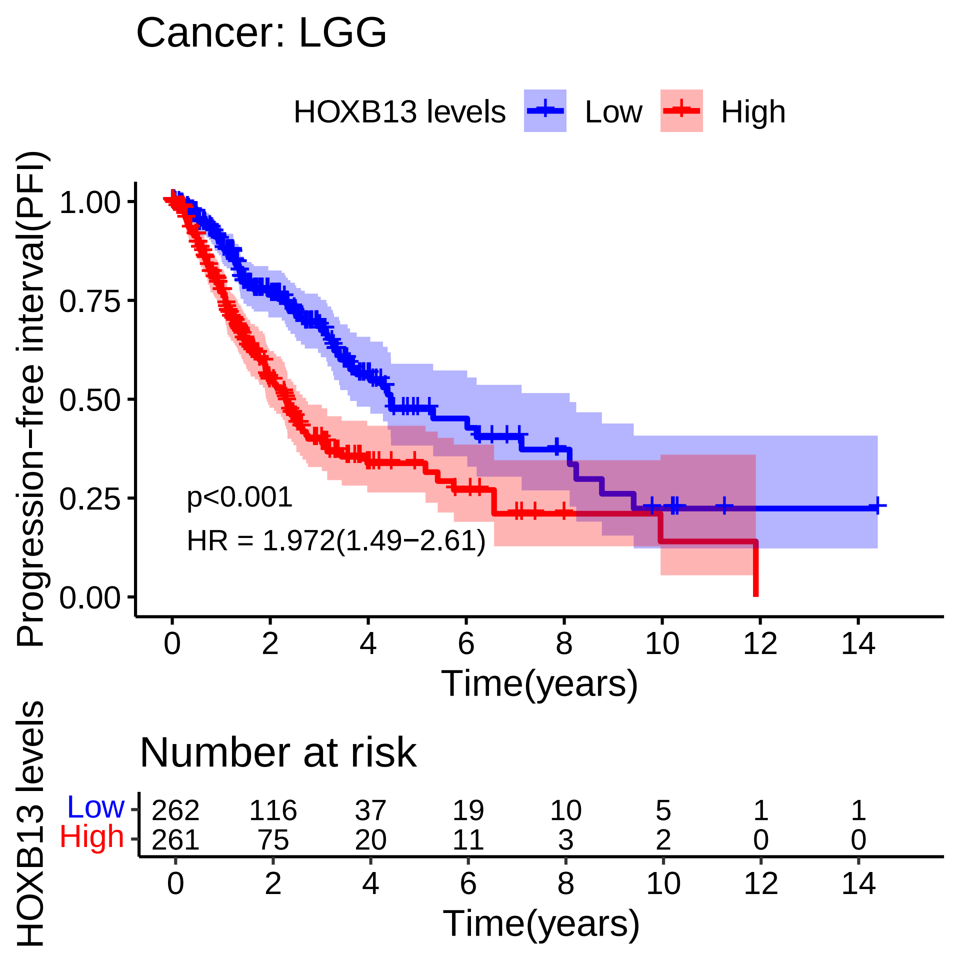
Cancer: LGG
HOXB13 levels
Low
High
Progression−free interval(PFI)
1.00
0.75
0.50
0.25
0.00
p<0.001
HR = 1.972(1.49−2.61)
0
2
4
6
8
10
12
14
Time(years)
Number at risk
HOXB13 levels
Low 262 116 37 19 10 5 1 1
High 261 75 20 11 3 2 0 0
Time(years)

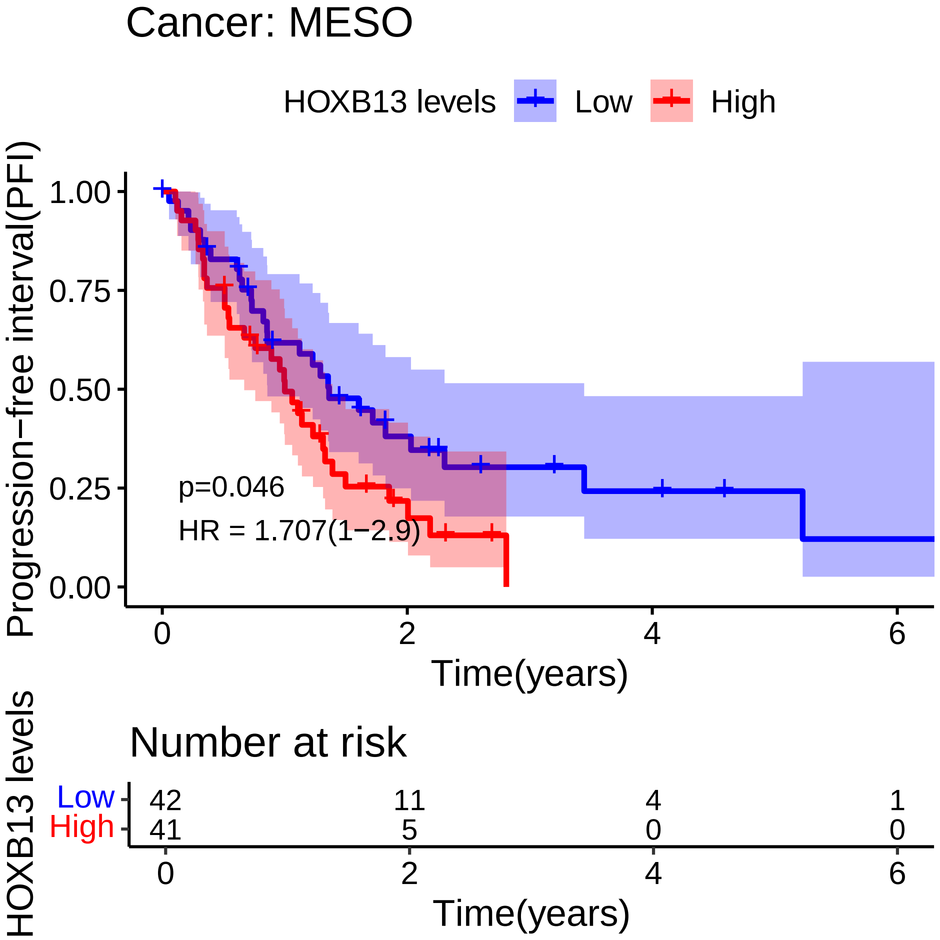

Cancer: MESO
HOXB13 levels
Low
High
Progression−free interval(PFI)
1.00
0.75
0.50
0.25
0.00
p=0.046
HR = 1.707(1−2.9)
0
2
4
6
Time(years)
Number at risk
HOXB13 levels
Low
High
42
11
4
1
41
5
0
0
0
2
4
6
Time(years)

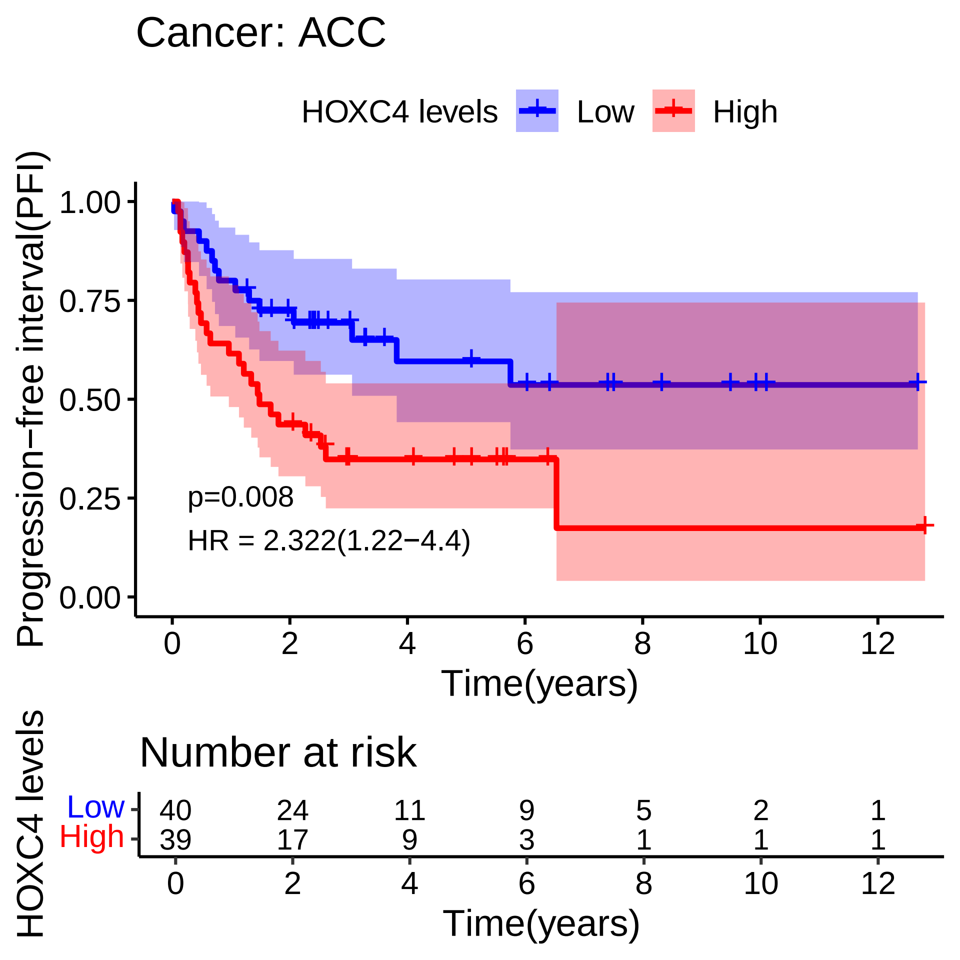

Cancer: ACC
HOXC4 levels
Low
High
Progression−free interval(PFI)
1.00
0.75
0.50
0.25
0.00
p=0.008
HR = 2.322(1.22−4.4)
0
2
4
6
8
10
12
Time(years)
Number at risk
HOXC4 levels
Low 40 24 11 9 5 2 1
High 39 17 9 3 1 1 1
Time(years)

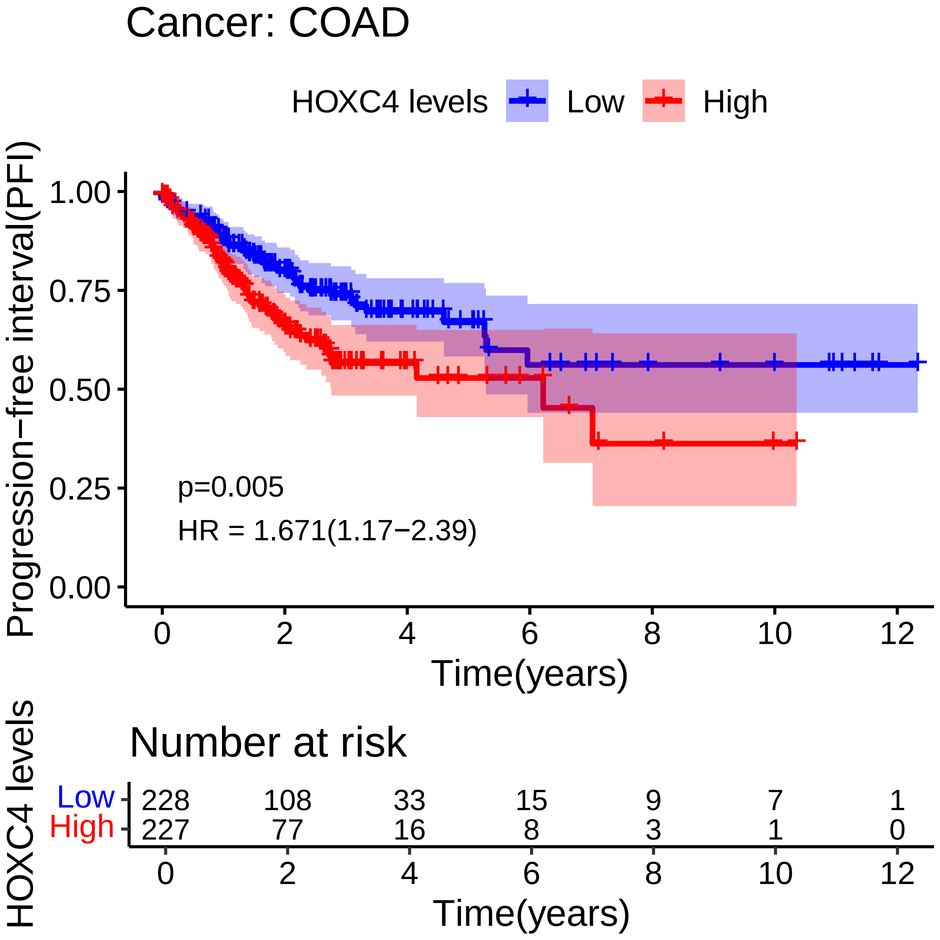

Cancer: COAD
HOXC4 levels
Low
High
Progression−free interval(PFI)
1.00
0.75
0.50
0.25
0.00
p=0.005
HR = 1.671(1.17−2.39)
0
2
4
6
8
10
12
Time(years)
Number at risk
HOXC4 levels
Low 228 108 33 15 9 7 1
High 227 77 16 8 3 1 0
Time(years)

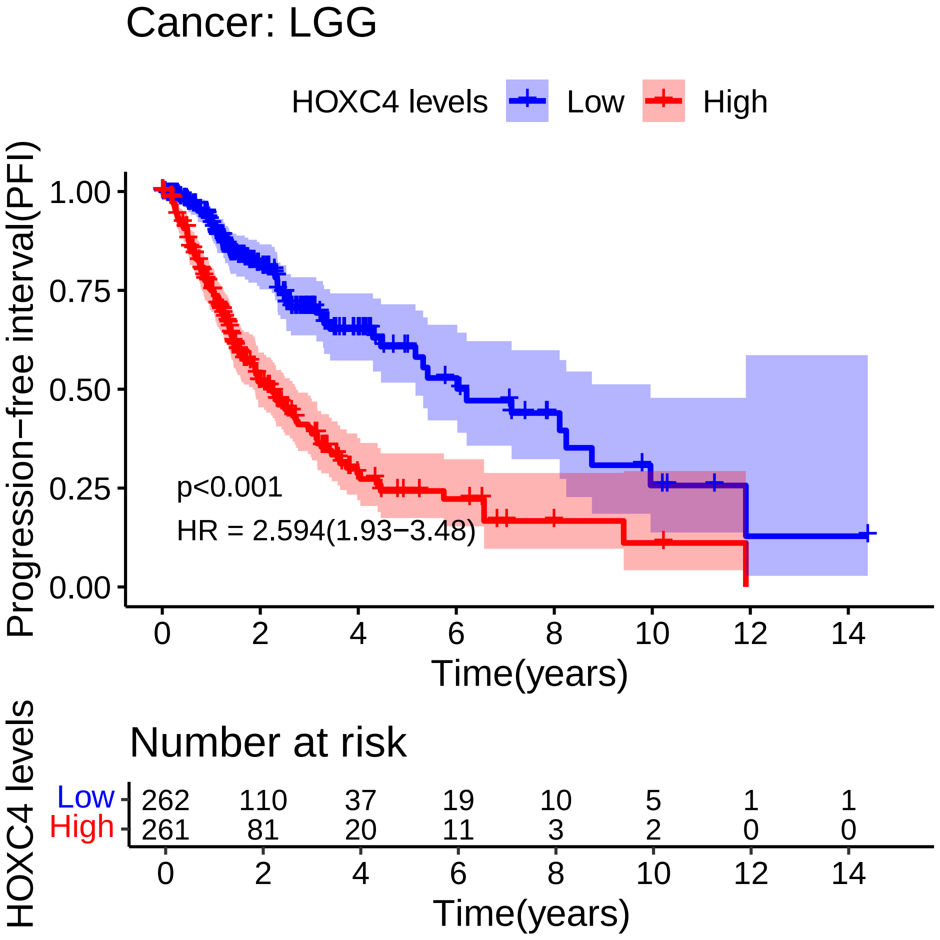
Cancer: LGG
HOXC4 levels
Low
High
Progression−free interval(PFI)
1.00
0.75
0.50
0.25
0.00
p<0.001
HR = 2.594(1.93−3.48)
0
2
4
6
8
10
12
14
Time(years)
Number at risk
HOXC4 levels
Low 262 110 37 19 10 5 1 1
High 261 81 20 11 3 2 0 0
0
2
4
6
8
10
12
14
Time(years)

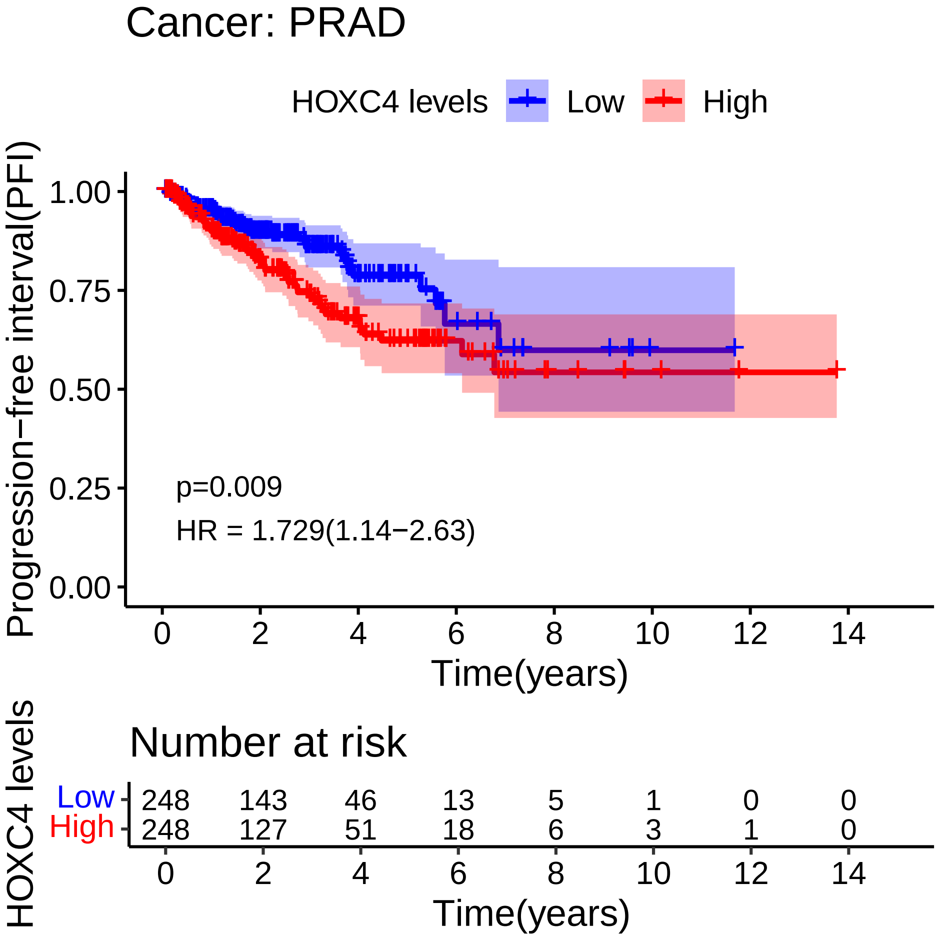
Cancer: PRAD
HOXC4 levels
Low
High
Progression−free interval(PFI)
1.00
0.75
0.50
0.25
0.00
p=0.009
HR = 1.729(1.14−2.63)
0
2
4
6
8
10
12
14
Time(years)
Number at risk
HOXC4 levels
Low 248 143 46 13 5 1 0 0
High 248 127 51 18 6 3 1 0
Time(years)

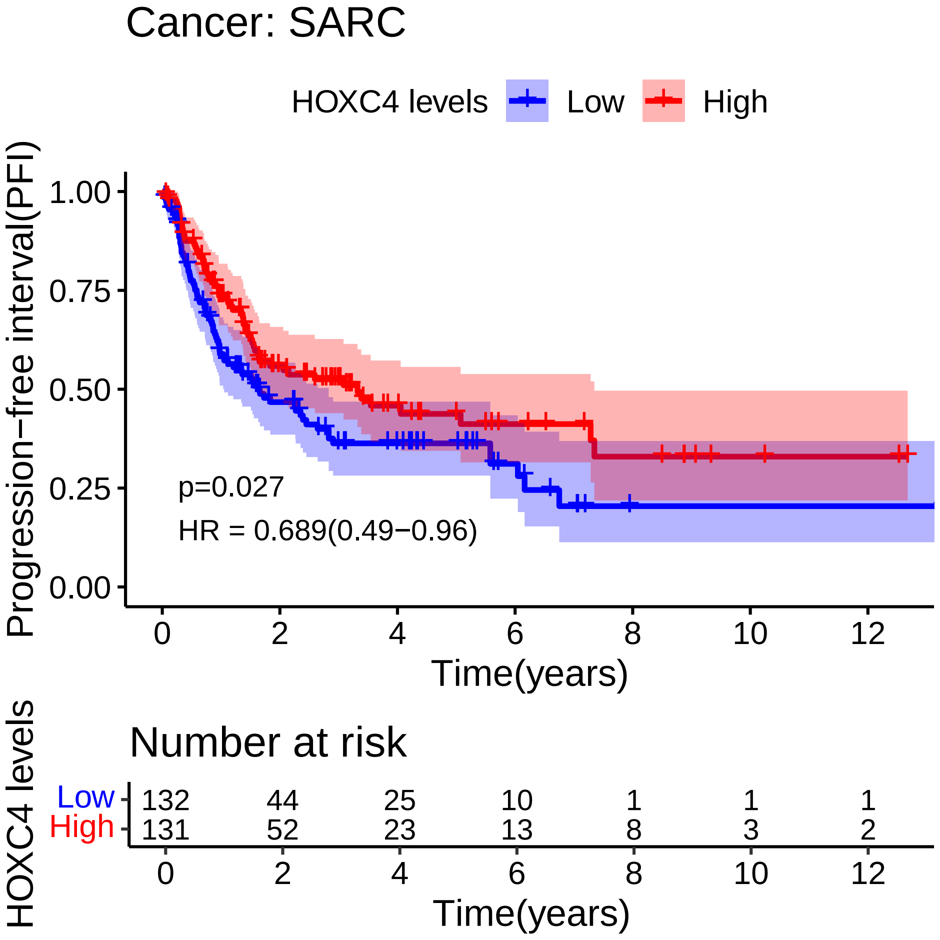
Cancer: SARC
HOXC4 levels
Low
High
Progression−free interval(PFI)
1.00
0.75
0.50
0.25
0.00
p=0.027
HR = 0.689(0.49−0.96)
0
2
4
6
8
10
12
Time(years)
Number at risk
HOXC4 levels
Low
High
132 44 25 10 1 1 1
131 52 23 13 8 3 2
0
2
4
6
8
10
12
Time(years)

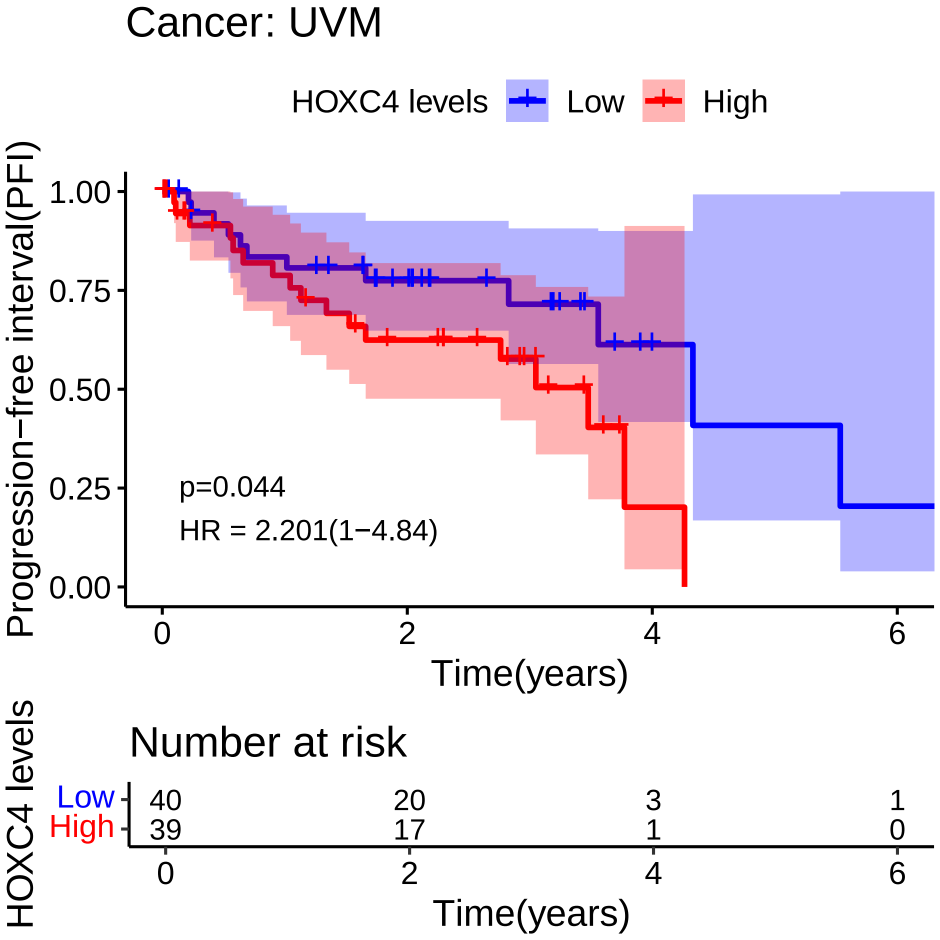

Cancer: UVM
HOXC4 levels
Low
High
Progression−free interval(PFI)
1.00
0.75
0.50
0.25
0.00
p=0.044
HR = 2.201(1−4.84)
0
2
4
6
Time(years)
Number at risk
HOXC4 levels
Low
High
40
20
3
1
39
17
1
0
0
2
4
6
Time(years)

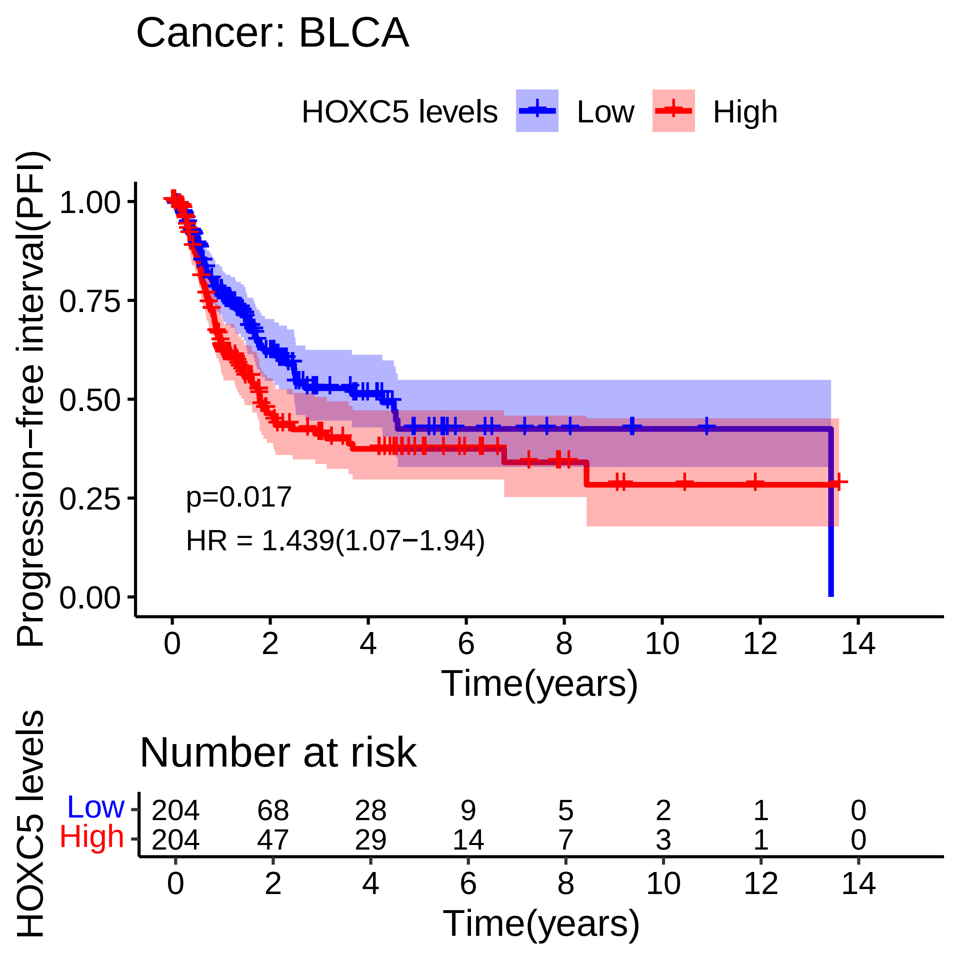

Cancer: BLCA
HOXC5 levels
Low
High
Progression−free interval(PFI)
1.00
0.75
0.50
0.25
0.00
p=0.017
HR = 1.439(1.07−1.94)
0
2
4
6
8
10
12
14
Time(years)
Number at risk
HOXC5 levels
Low 204 68 28 9 5 2 1 0
High 204 47 29 14 7 3 1 0
Time(years)

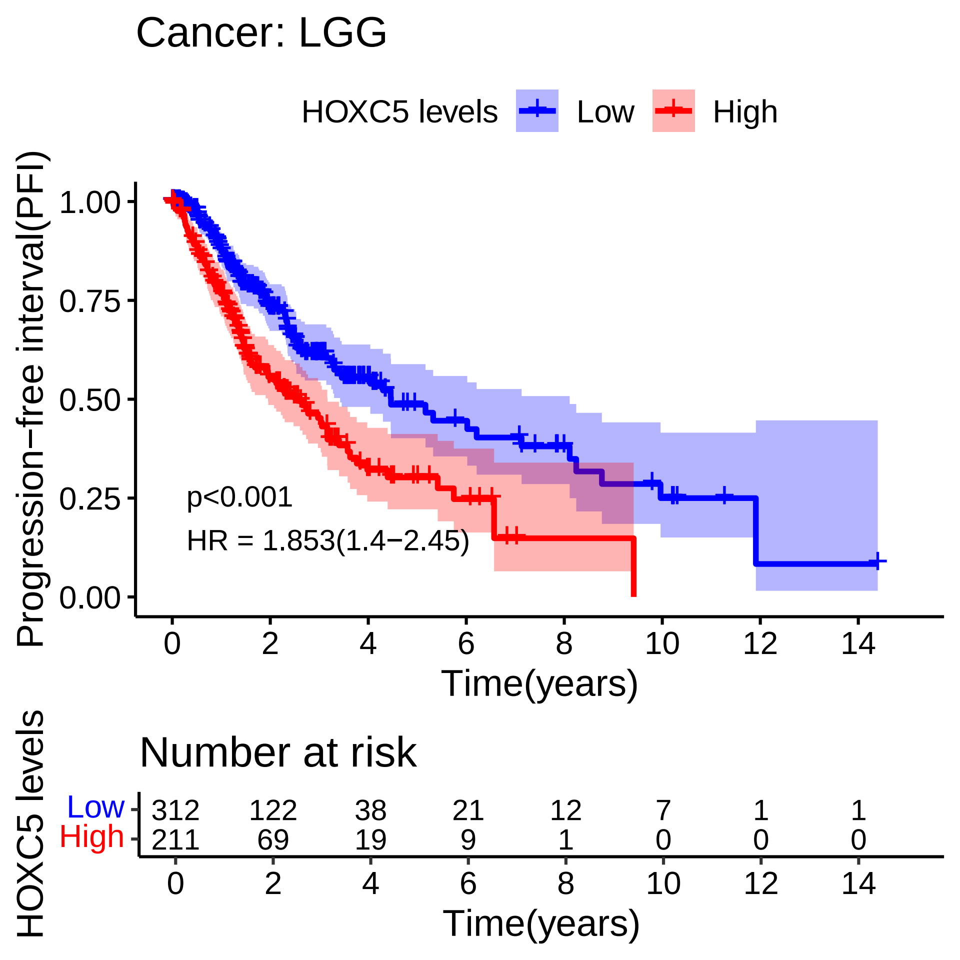
Cancer: LGG
HOXC5 levels
Low
High
Progression−free interval(PFI)
1.00
0.75
0.50
0.25
0.00
p<0.001
HR = 1.853(1.4−2.45)
0
2
4
6
8
10
12
14
Time(years)
Number at risk
HOXC5 levels
Low 312 122 38 21 12 7 1 1
High 211 69 19 9 1 0 0 0
0
2
4
6
8
10
12
14
Time(years)

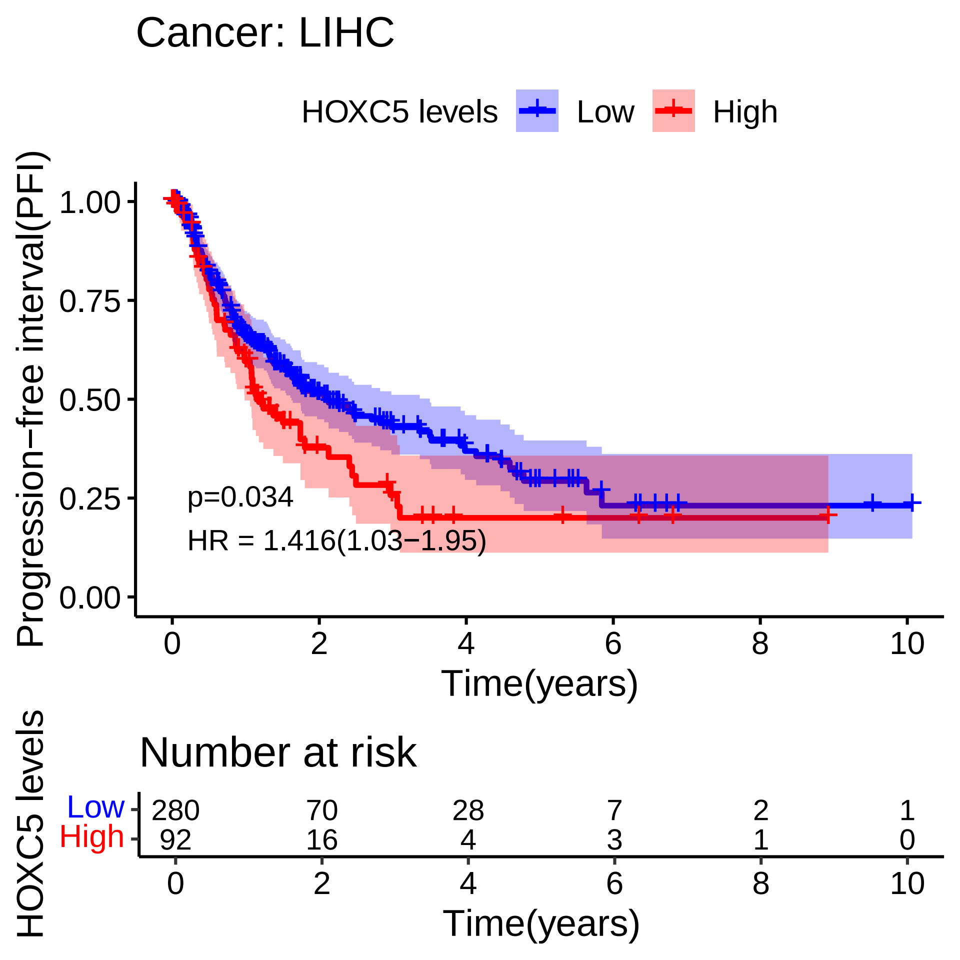

Cancer: LIHC
HOXC5 levels
Low
High
Progression−free interval(PFI)
1.00
0.75
0.50
0.25
0.00
p=0.034
HR = 1.416(1.03−1.95)
0
2
4
6
8
10
Time(years)
Number at risk
HOXC5 levels
Low 280 70 28 7 2 1
High 92 16 4 3 1 0
Time(years)

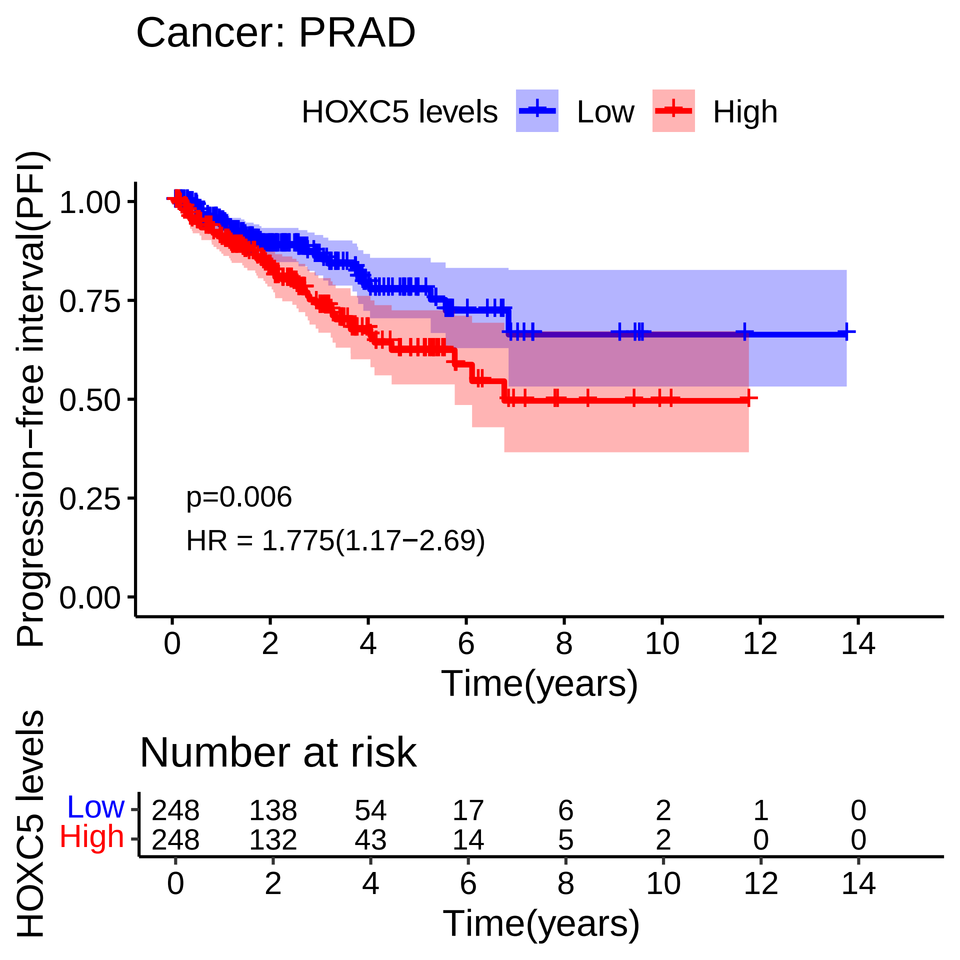
Cancer: PRAD
HOXC5 levels
Low
High
Progression−free interval(PFI)
1.00
0.75
0.50
0.25
0.00
p=0.006
HR = 1.775(1.17−2.69)
0
2
4
6
8
10
12
14
Time(years)
Number at risk
HOXC5 levels
Low 248 138 54 17 6 2 1 0
High 248 132 43 14 5 2 0 0
Time(years)

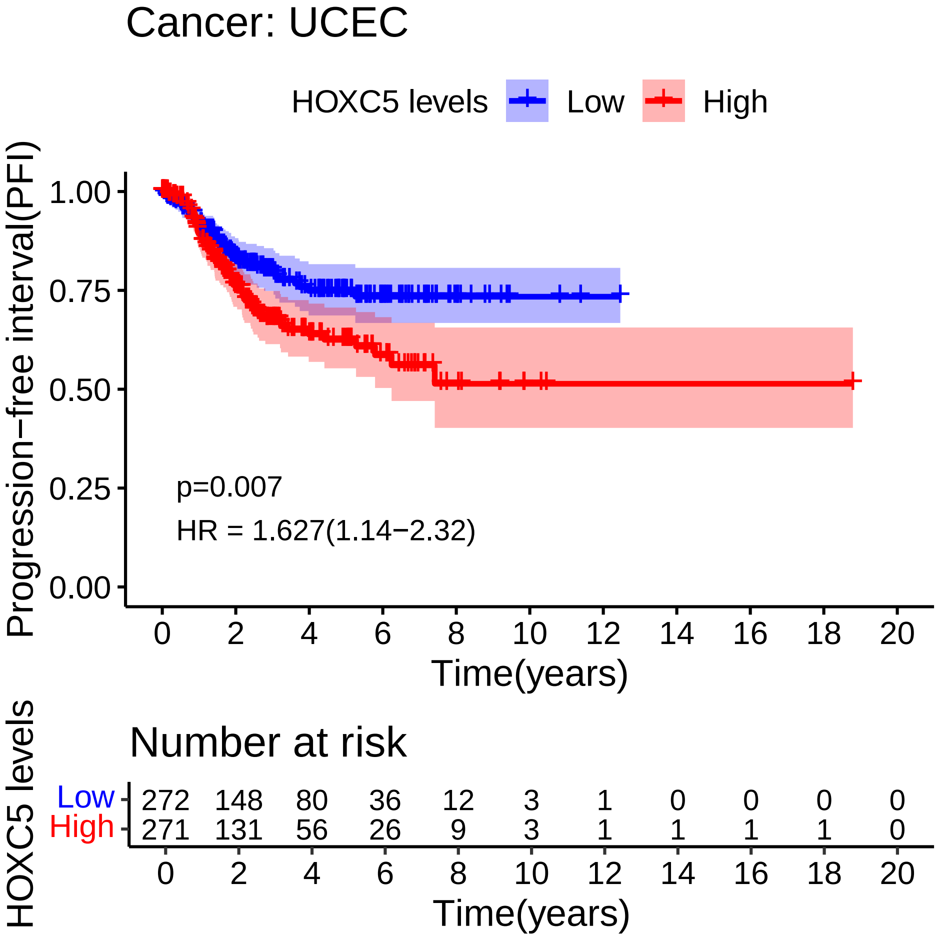
Cancer: UCEC
HOXC5 levels
Low
High
Progression−free interval(PFI)
1.00
0.75
0.50
0.25
0.00
p=0.007
HR = 1.627(1.14−2.32)
0
2
4
6
8
10
12
14
16
18
20
Time(years)
Number at risk
HOXC5 levels
Low 272 148 80 36 12 3 1 0 0 0 0
High 271 131 56 26 9 3 1 1 1 1 0
Time(years)

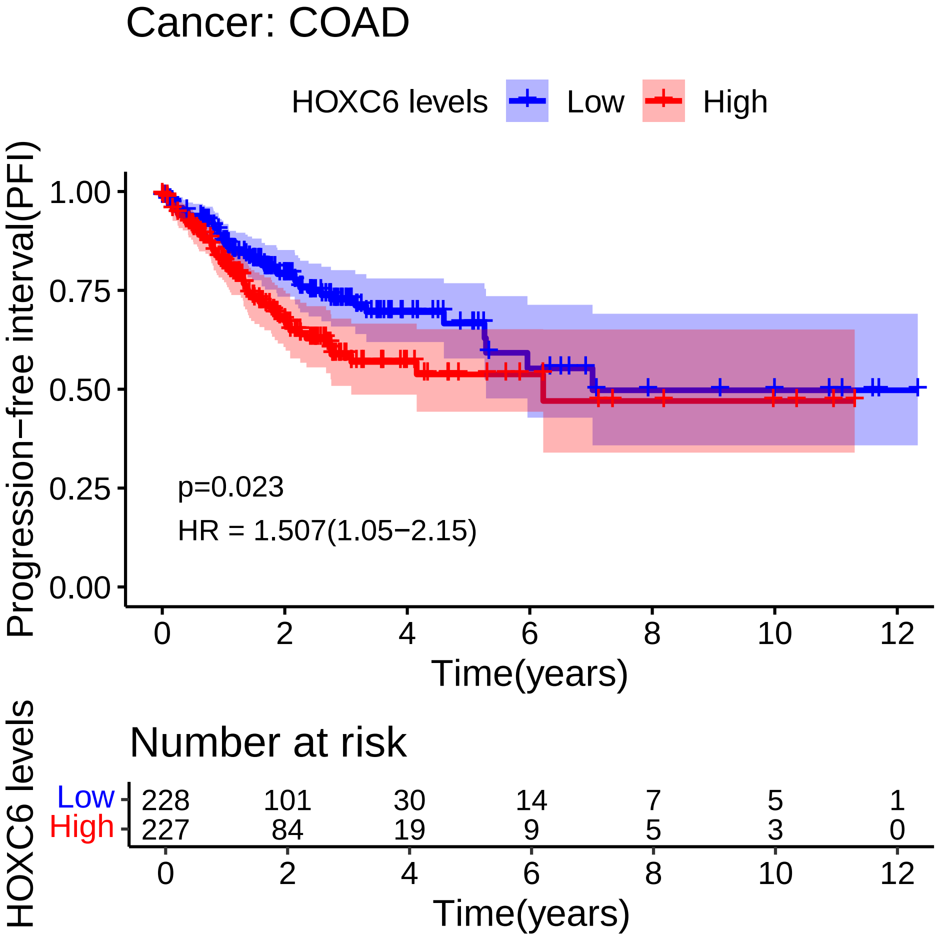

Cancer: COAD
HOXC6 levels
Low
High
Progression−free interval(PFI)
1.00
0.75
0.50
0.25
0.00
p=0.023
HR = 1.507(1.05−2.15)
0
2
4
6
8
10
12
Time(years)
Number at risk
HOXC6 levels
Low 228 101 30 14 7 5 1
High 227 84 19 9 5 3 0
Time(years)

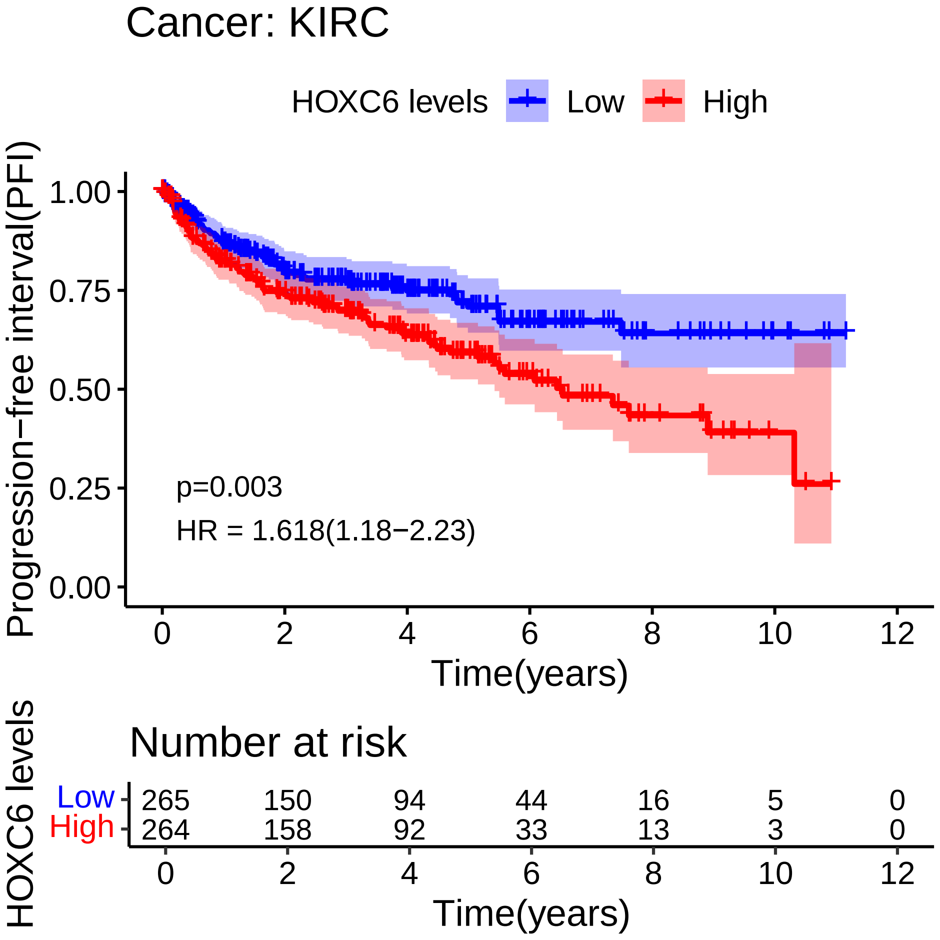

Cancer: KIRC
HOXC6 levels
Low
High
Progression−free interval(PFI)
1.00
0.75
0.50
0.25
0.00
p=0.003
HR = 1.618(1.18−2.23)
0
2
4
6
8
10
12
Time(years)
Number at risk
HOXC6 levels
Low
High
265 150 94 44 16 5 0
264 158 92 33 13 3 0
Time(years)

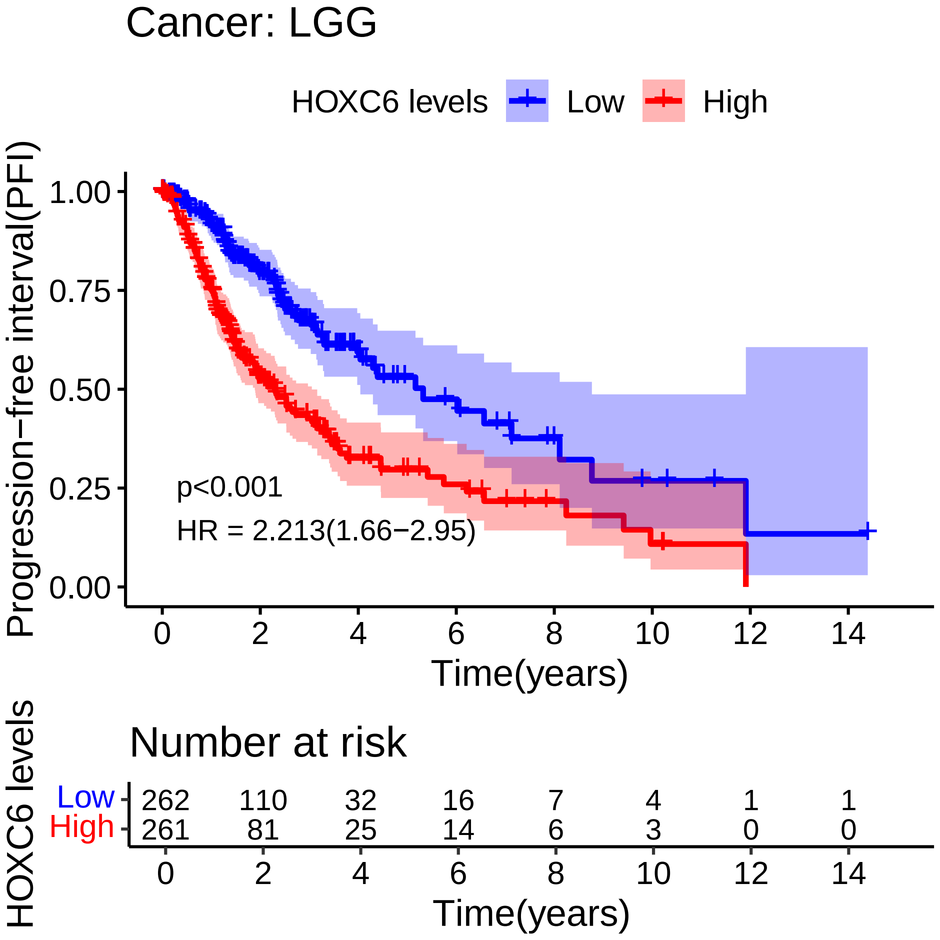

Cancer: LGG
HOXC6 levels
Low
High
Progression−free interval(PFI)
1.00
0.75
0.50
0.25
0.00
p<0.001
HR = 2.213(1.66−2.95)
0
2
4
6
8
10
12
14
Time(years)
Number at risk
HOXC6 levels
Low 262 110 32 16 7 4 1 1
High 261 81 25 14 6 3 0 0
Time(years)

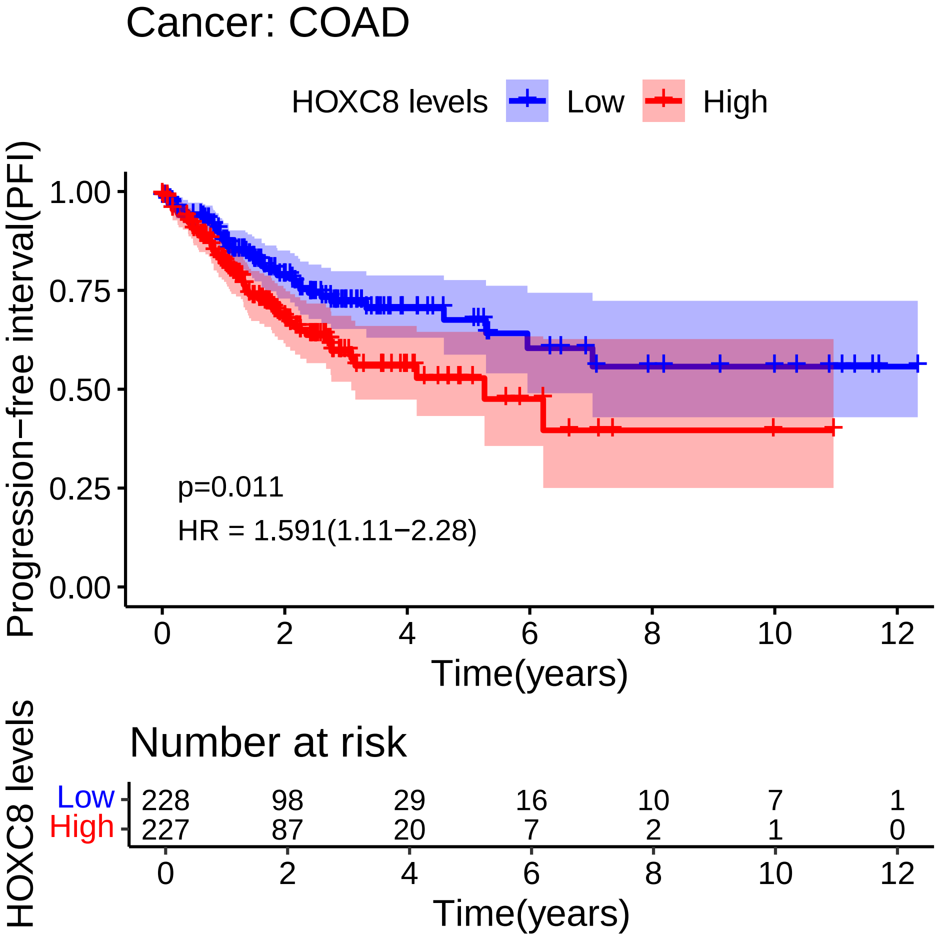
Cancer: COAD
HOXC8 levels
Low
High
Progression−free interval(PFI)
1.00
0.75
0.50
0.25
0.00
p=0.011
HR = 1.591(1.11−2.28)
0
2
4
6
8
10
12
Time(years)
Number at risk
HOXC8 levels
Low 228 98 29 16 10 7 1
High 227 87 20 7 2 1 0
0
2
4
6
8
10
12
Time(years)

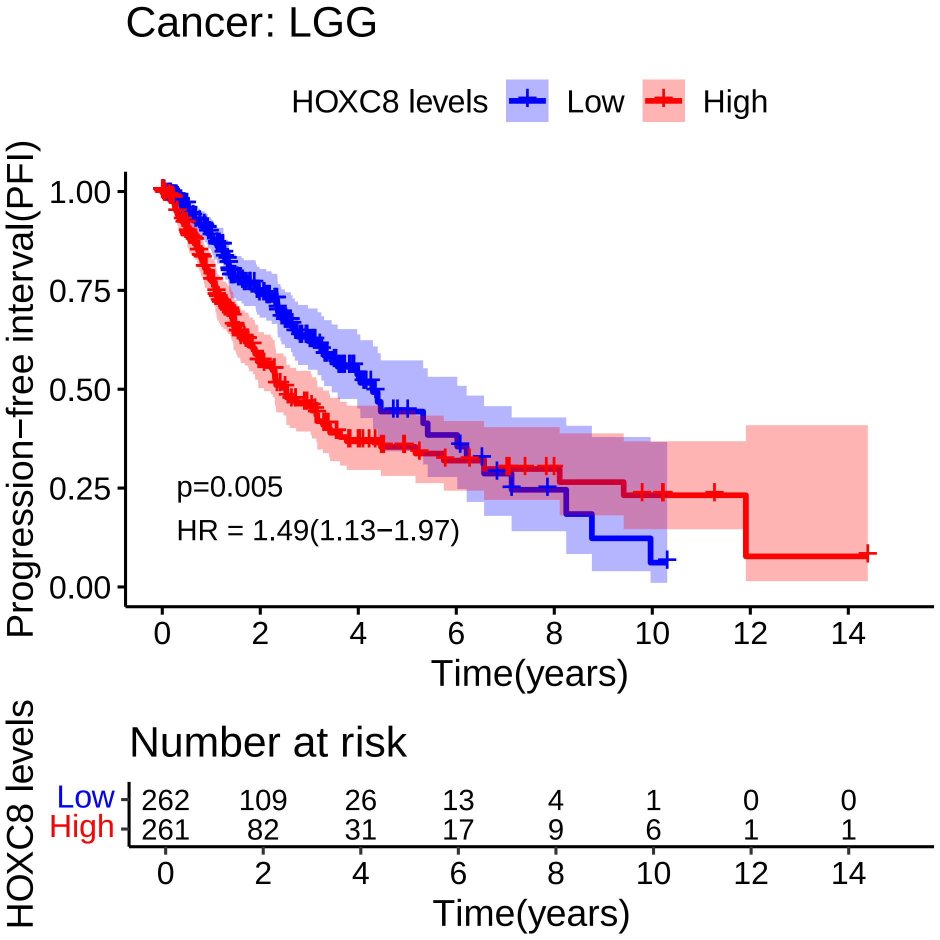
Cancer: LGG
HOXC8 levels
Low
High
Progression−free interval(PFI)
1.00
0.75
0.50
0.25
0.00
p=0.005
HR = 1.49(1.13−1.97)
0
2
4
6
8
10
12
14
Time(years)
Number at risk
HOXC8 levels
Low 262 109 26 13 4 1 0 0
High 261 82 31 17 9 6 1 1
0
2
4
6
8
10
12
14
Time(years)

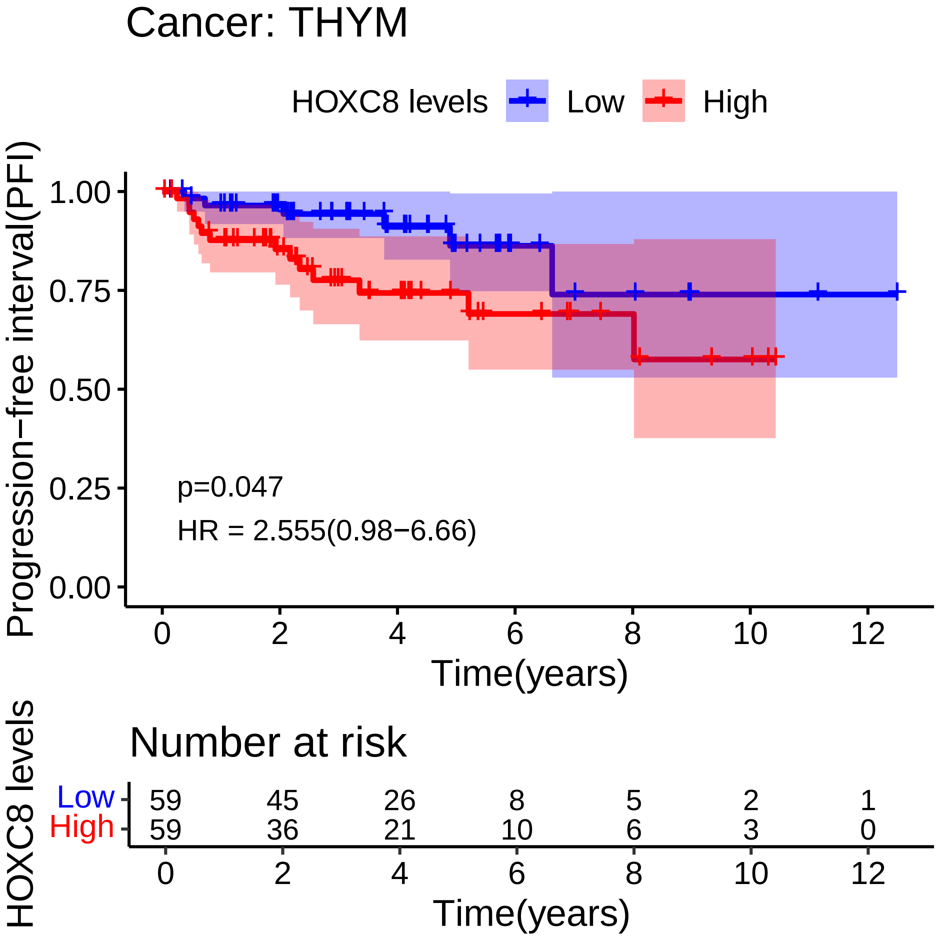
Cancer: THYM
HOXC8 levels
Low
High
Progression−free interval(PFI)
1.00
0.75
0.50
0.25
0.00
p=0.047
HR = 2.555(0.98−6.66)
0
2
4
6
8
10
12
Time(years)
Number at risk
HOXC8 levels
Low 59 45 26 8 5 2 1
High 59 36 21 10 6 3 0
Time(years)

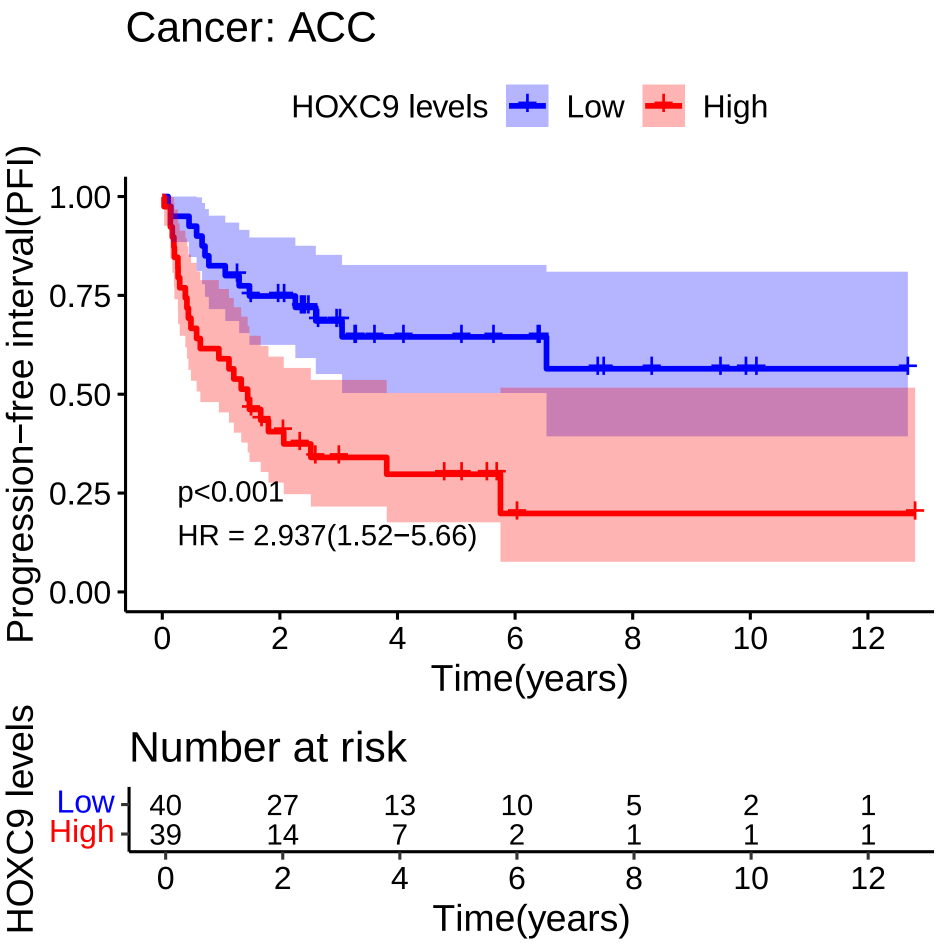

Cancer: ACC
HOXC9 levels
Low
High
Progression−free interval(PFI)
1.00
0.75
0.50
0.25
0.00
p<0.001
HR = 2.937(1.52−5.66)
0
2
4
6
8
10
12
Time(years)
Number at risk
HOXC9 levels
Low 40 27 13 10 5 2 1
High 39 14 7 2 1 1 1
Time(years)

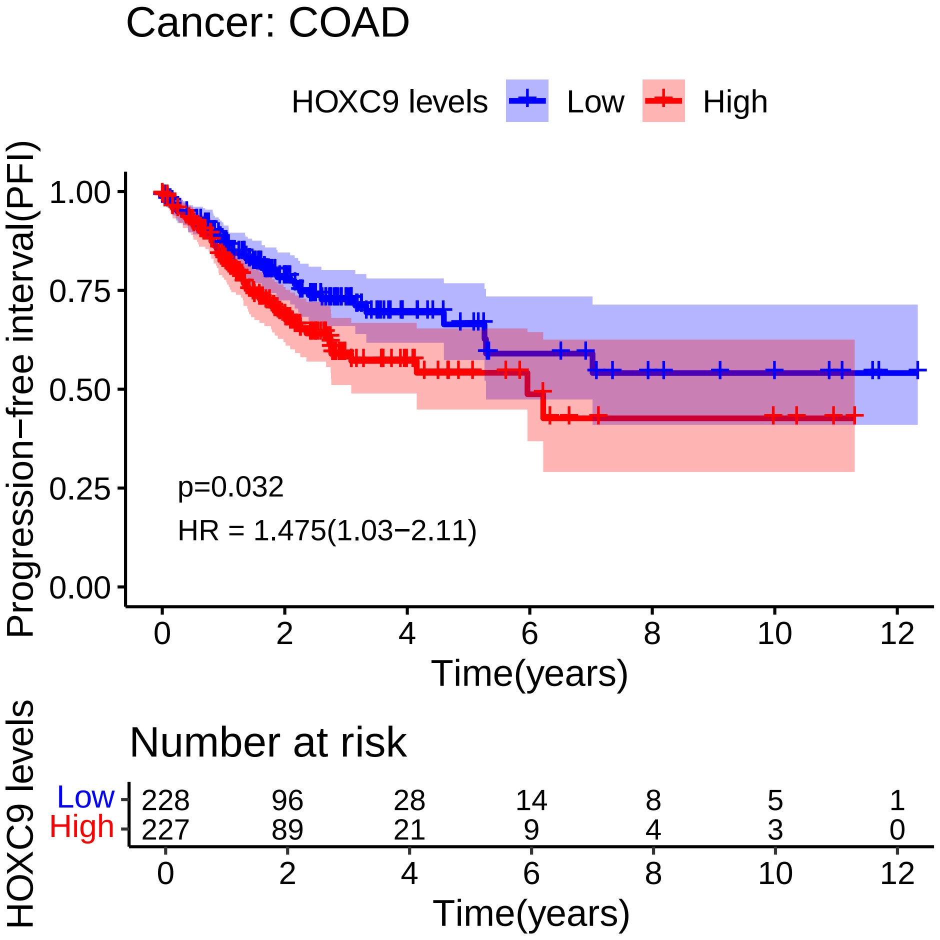
Cancer: COAD
HOXC9 levels
Low
High
Progression−free interval(PFI)
1.00
0.75
0.50
0.25
0.00
p=0.032
HR = 1.475(1.03−2.11)
0
2
4
6
8
10
12
Time(years)
Number at risk
HOXC9 levels
Low 228 96 28 14 8 5 1
High 227 89 21 9 4 3 0
Time(years)

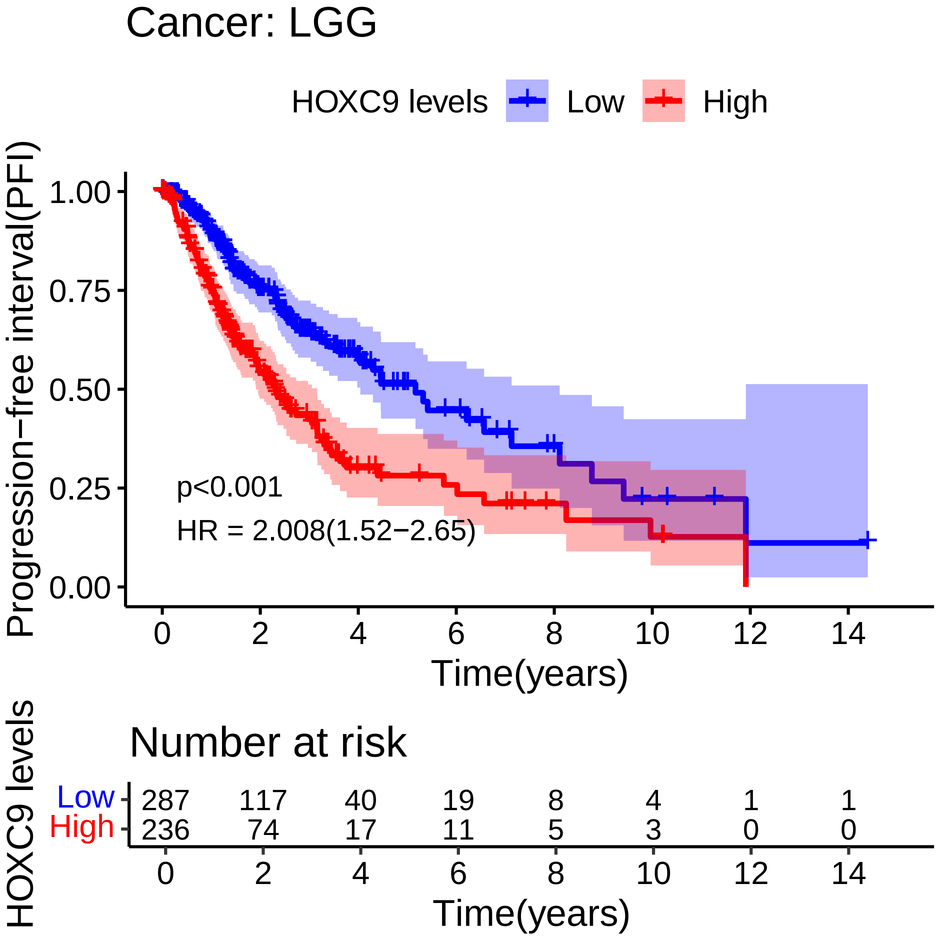
Cancer: LGG
HOXC9 levels
Low
High
Progression−free interval(PFI)
1.00
0.75
0.50
0.25
0.00
p<0.001
HR = 2.008(1.52−2.65)
0
2
4
6
8
10
12
14
Time(years)
Number at risk
HOXC9 levels
Low 287 117 40 19 8 4 1 1
High 236 74 17 11 5 3 0 0
0
2
4
6
8
10
12
14
Time(years)

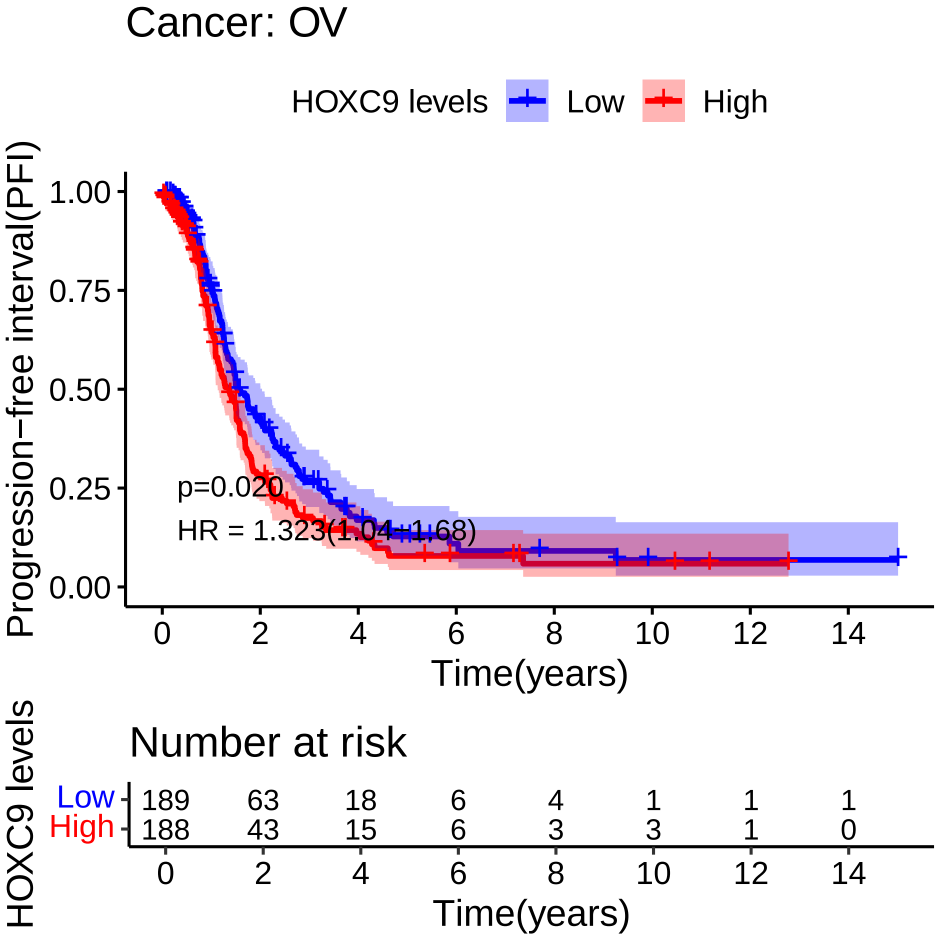
Cancer: OV
HOXC9 levels
Low
High
Progression−free interval(PFI)
1.00
0.75
0.50
0.25
0.00
p=0.020
HR = 1.323(1.04−1.68)
0
2
4
6
8
10
12
14
Time(years)
Number at risk
HOXC9 levels
Low 189 63 18 6 4 1 1 1
High 188 43 15 6 3 3 1 0
Time(years)

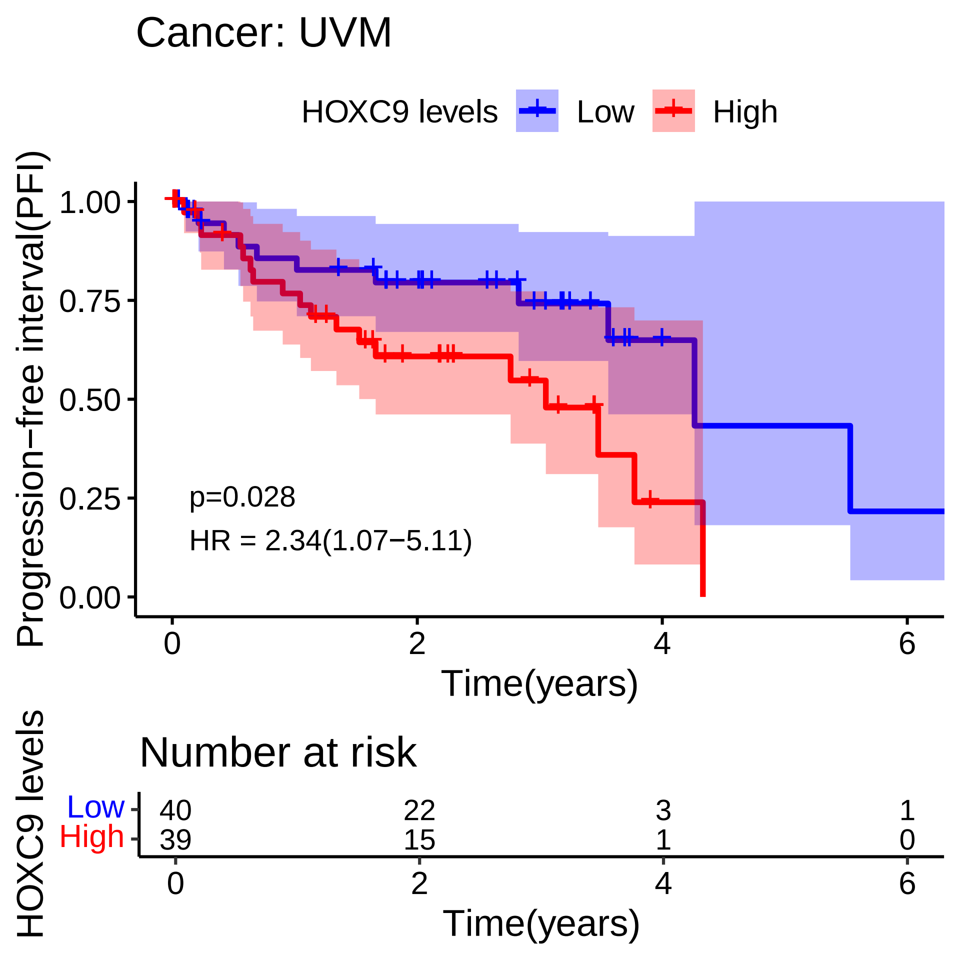
Cancer: UVM
HOXC9 levels
Low
High
Progression−free interval(PFI)
1.00
0.75
0.50
0.25
0.00
p=0.028
HR = 2.34(1.07−5.11)
0
2
4
6
Time(years)
Number at risk
HOXC9 levels
Low 40 22 3 1
High 39 15 1 0
0
2
4
6
Time(years)

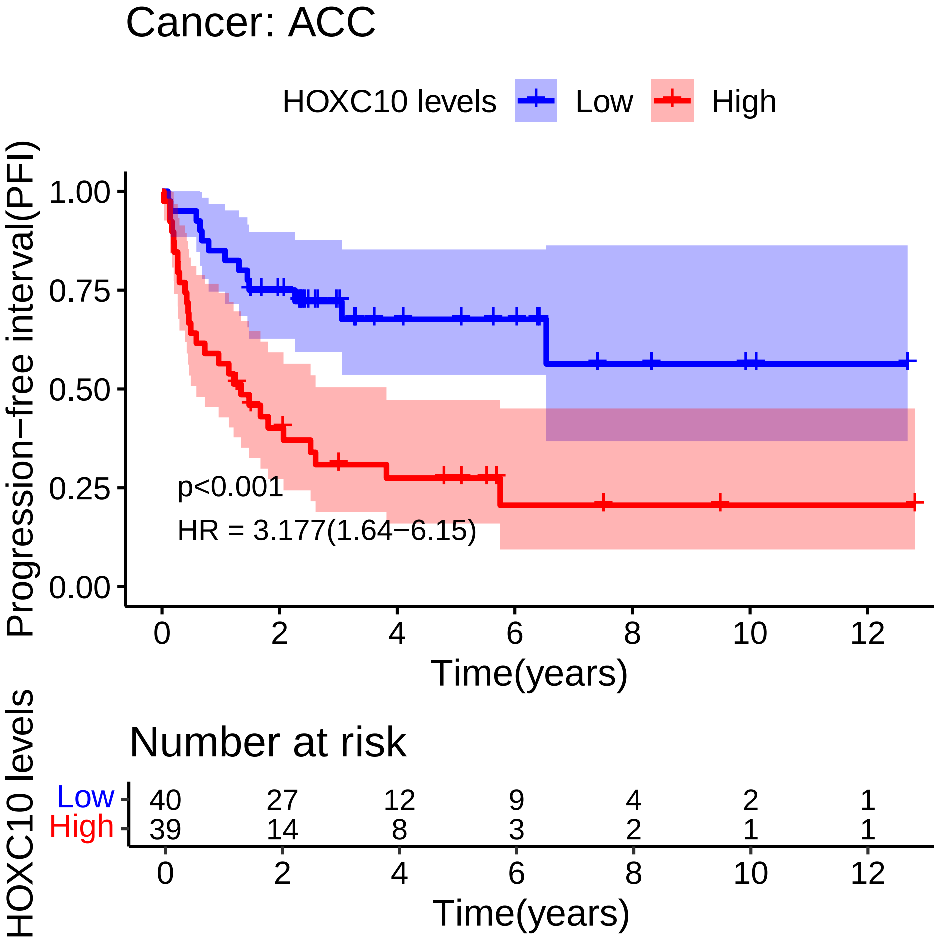

Cancer: ACC
HOXC10 levels
Low
High
Progression−free interval(PFI)
1.00
0.75
0.50
0.25
0.00
p<0.001
HR = 3.177(1.64−6.15)
0
2
4
6
8
10
12
Time(years)
Number at risk
HOXC10 levels
Low 40 27 12 9 4 2 1
High 39 14 8 3 2 1 1
Time(years)

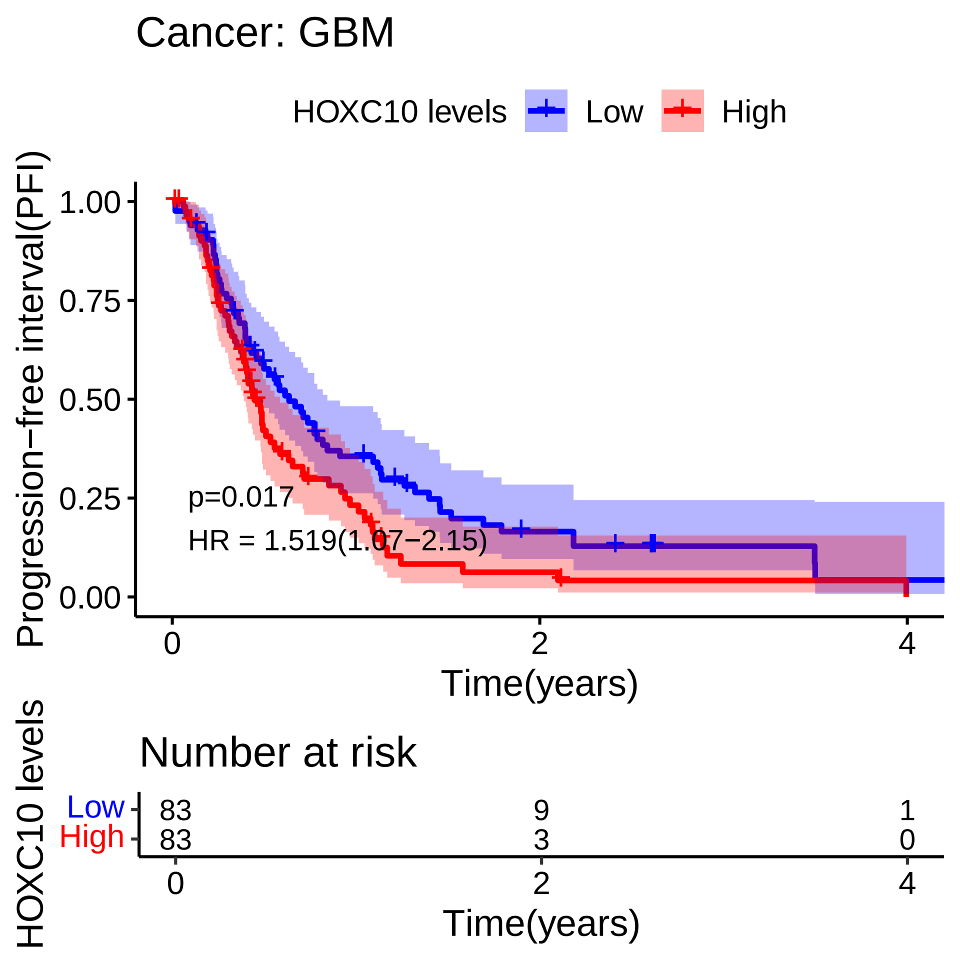

Cancer: GBM
HOXC10 levels
Low
High
Progression−free interval(PFI)
1.00
0.75
0.50
0.25
0.00
p=0.017
HR = 1.519(1.07−2.15)
0
2
4
Time(years)
Number at risk
HOXC10 levels
Low 83 9 1
High 83 3 0
0
2
4
Time(years)

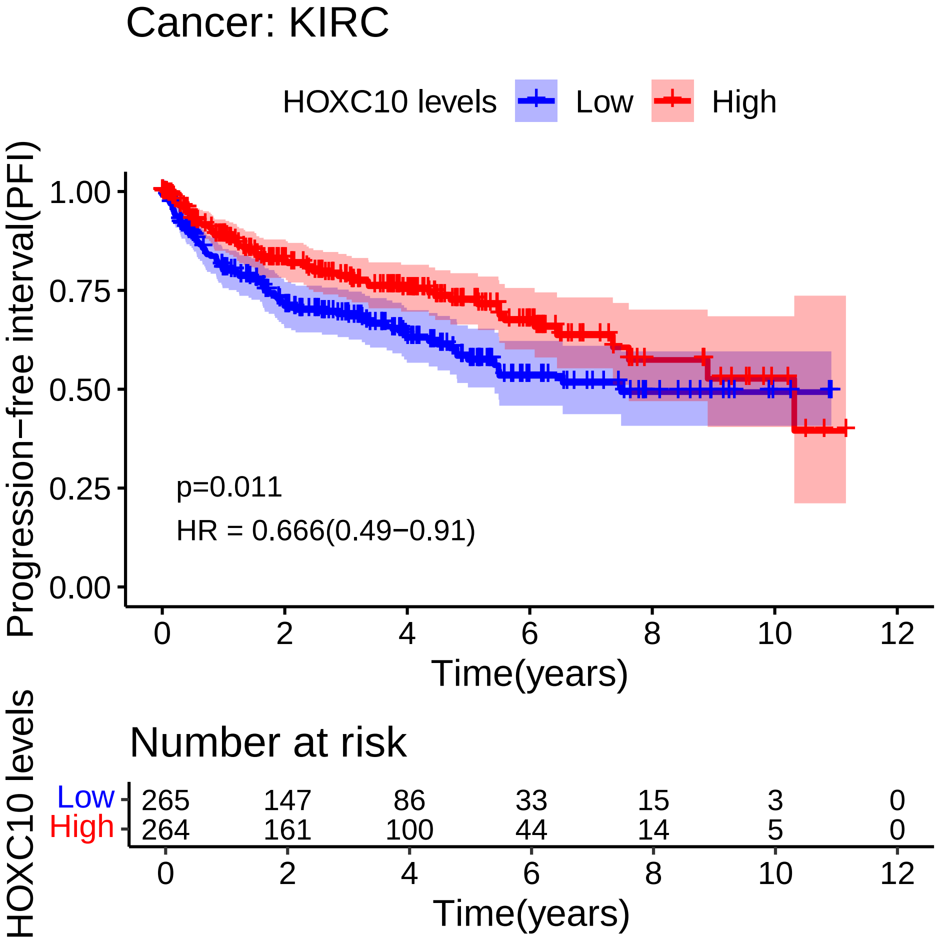
Cancer: KIRC
HOXC10 levels
Low
High
Progression−free interval(PFI)
1.00
0.75
0.50
0.25
0.00
p=0.011
HR = 0.666(0.49−0.91)
0
2
4
6
8
10
12
Time(years)
Number at risk
HOXC10 levels
Low 265 147 86 33 15 3 0
High 264 161 100 44 14 5 0
0
2
4
6
8
10
12
Time(years)

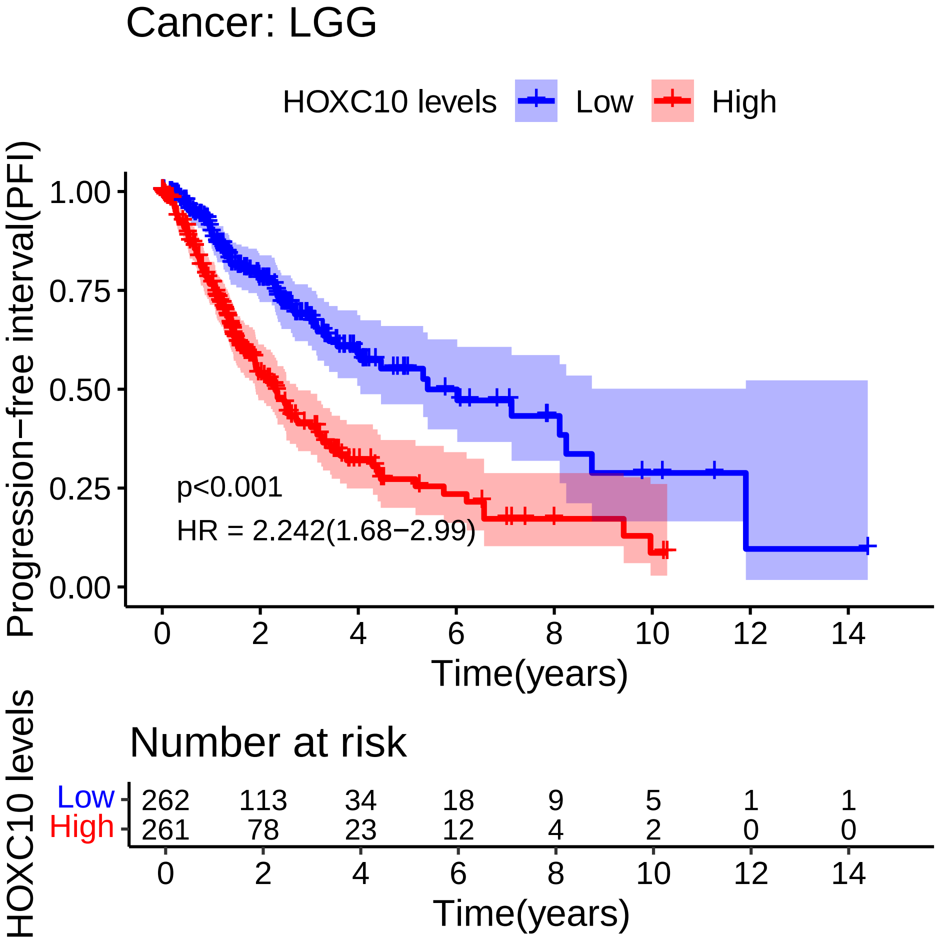
Cancer: LGG
HOXC10 levels
Low
High
Progression−free interval(PFI)
1.00
0.75
0.50
0.25
0.00
p<0.001
HR = 2.242(1.68−2.99)
0
2
4
6
8
10
12
14
Time(years)
Number at risk
HOXC10 levels
Low 262 113 34 18 9 5 1 1
High 261 78 23 12 4 2 0 0
0
2
4
6
8
10
12
14
Time(years)

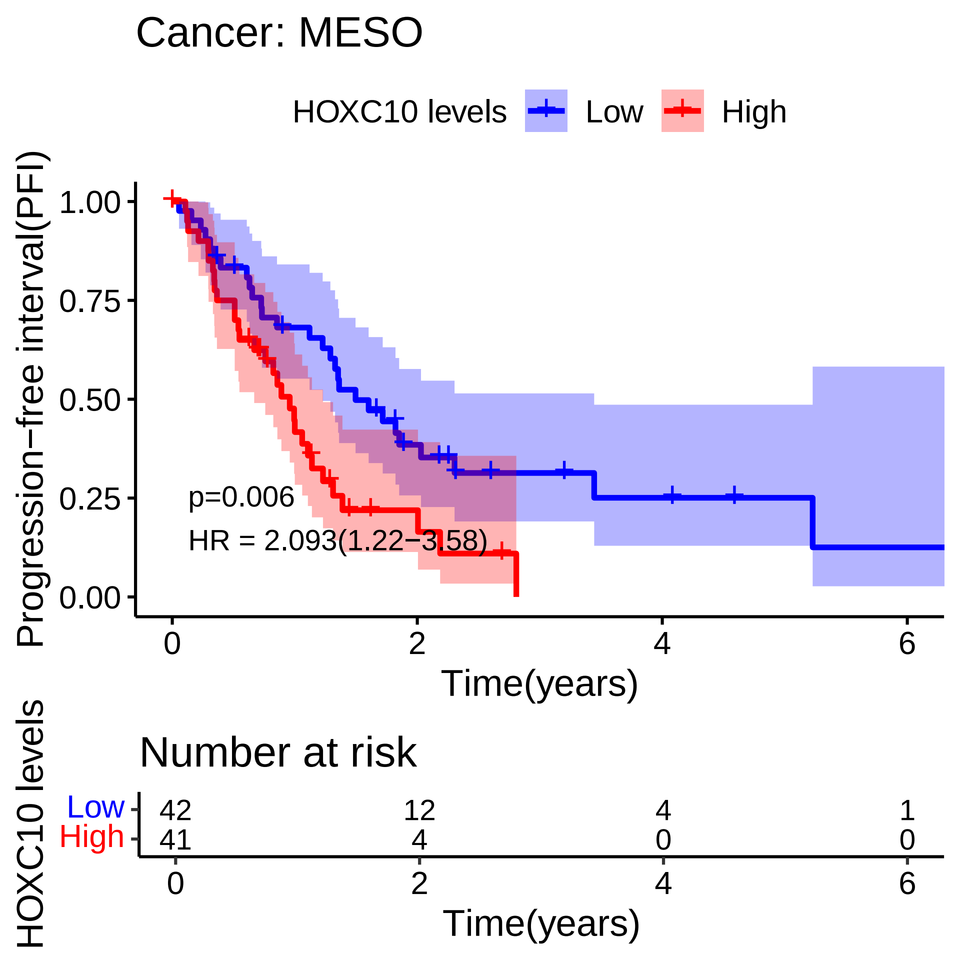

Cancer: MESO
HOXC10 levels
Low
High
Progression−free interval(PFI)
1.00
0.75
0.50
0.25
0.00
p=0.006
HR = 2.093(1.22−3.58)
0
2
4
6
Time(years)
Number at risk
HOXC10 levels
Low 42 12 4 1
High 41 4 0 0
Time(years)

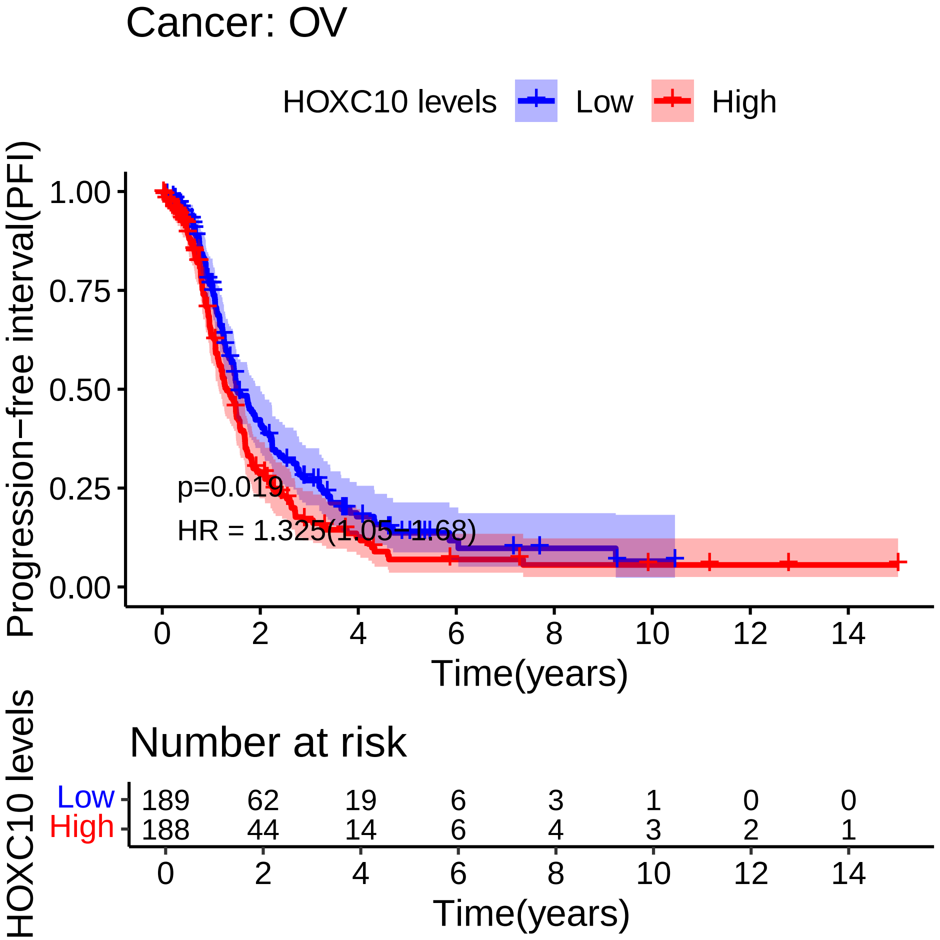

Cancer: OV
HOXC10 levels
Low
High
Progression−free interval(PFI)
1.00
0.75
0.50
0.25
0.00
p=0.019
HR = 1.325(1.05−1.68)
0
2
4
6
8
10
12
14
Time(years)
Number at risk
HOXC10 levels
Low 189 62 19 6 3 1 0 0
High 188 44 14 6 4 3 2 1
Time(years)

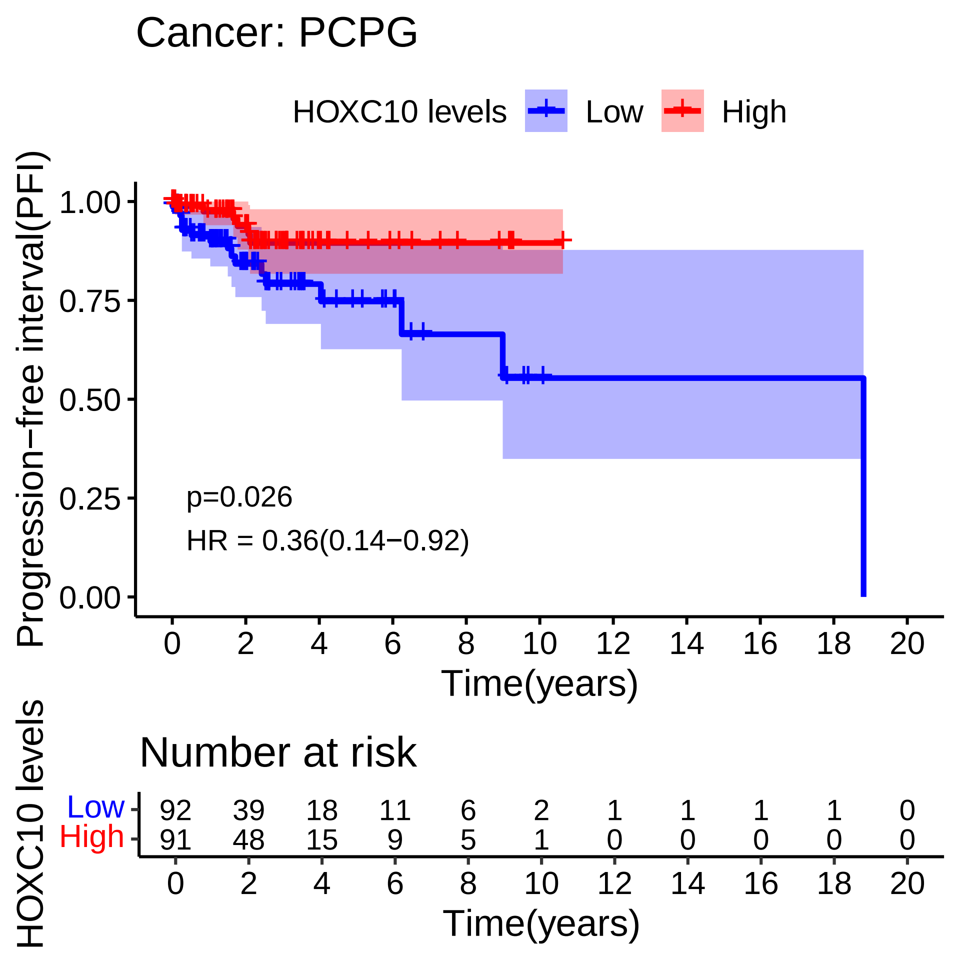

Cancer: PCPG
HOXC10 levels
Low
High
Progression−free interval(PFI)
1.00
0.75
0.50
0.25
0.00
p=0.026
HR = 0.36(0.14−0.92)
0 2 4 6 8 10 12 14 16 18 20
Time(years)
Number at risk
HOXC10 levels
Low 92 39 18 11 6 2 1 1 1 1 0
High 91 48 15 9 5 1 0 0 0 0 0
0 2 4 6 8 10 12 14 16 18 20
Time(years)

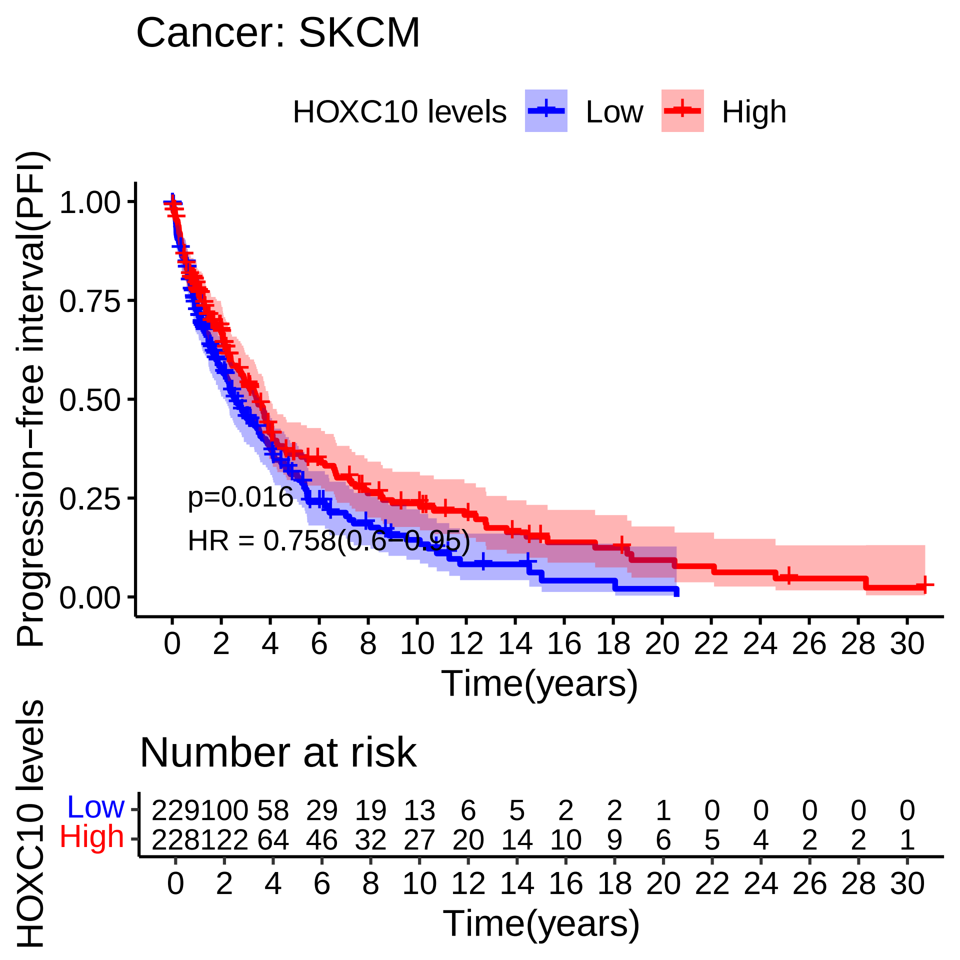

Cancer: SKCM
HOXC10 levels
Low
High
Progression−free interval(PFI)
1.00
0.75
0.50
0.25
0.00
p=0.016
HR = 0.758(0.6−0.95)
0 2 4 6 8 10 12 14 16 18 20 22 24 26 28 30
Time(years)
Number at risk
HOXC10 levels
Low 229 100 58 29 19 13 6 5 2 2 1 0 0 0 0 0
High 228 122 64 46 32 27 20 14 10 9 6 5 4 2 2 1
0 2 4 6 8 10 12 14 16 18 20 22 24 26 28 30
Time(years)

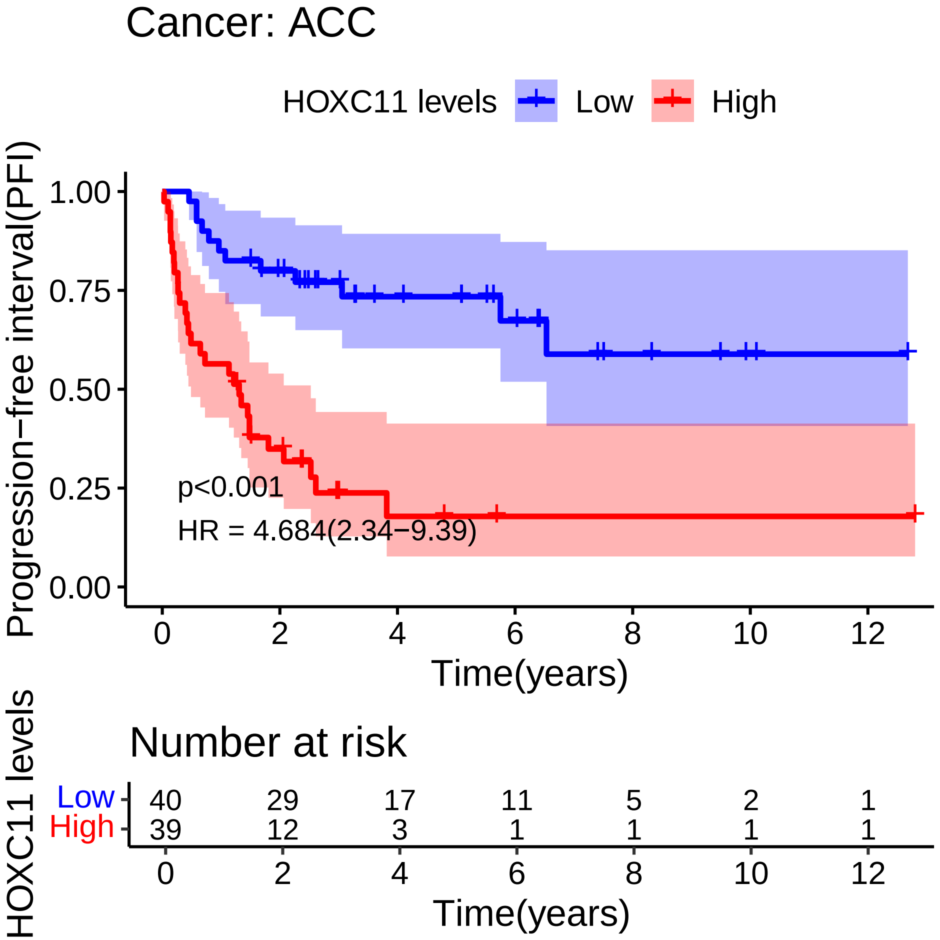

Cancer: ACC
HOXC11 levels
Low
High
Progression−free interval(PFI)
1.00
0.75
0.50
0.25
0.00
p<0.001
HR = 4.684(2.34−9.39)
0
2
4
6
8
10
12
Time(years)
Number at risk
HOXC11 levels
Low 40 29 17 11 5 2 1
High 39 12 3 1 1 1 1
Time(years)

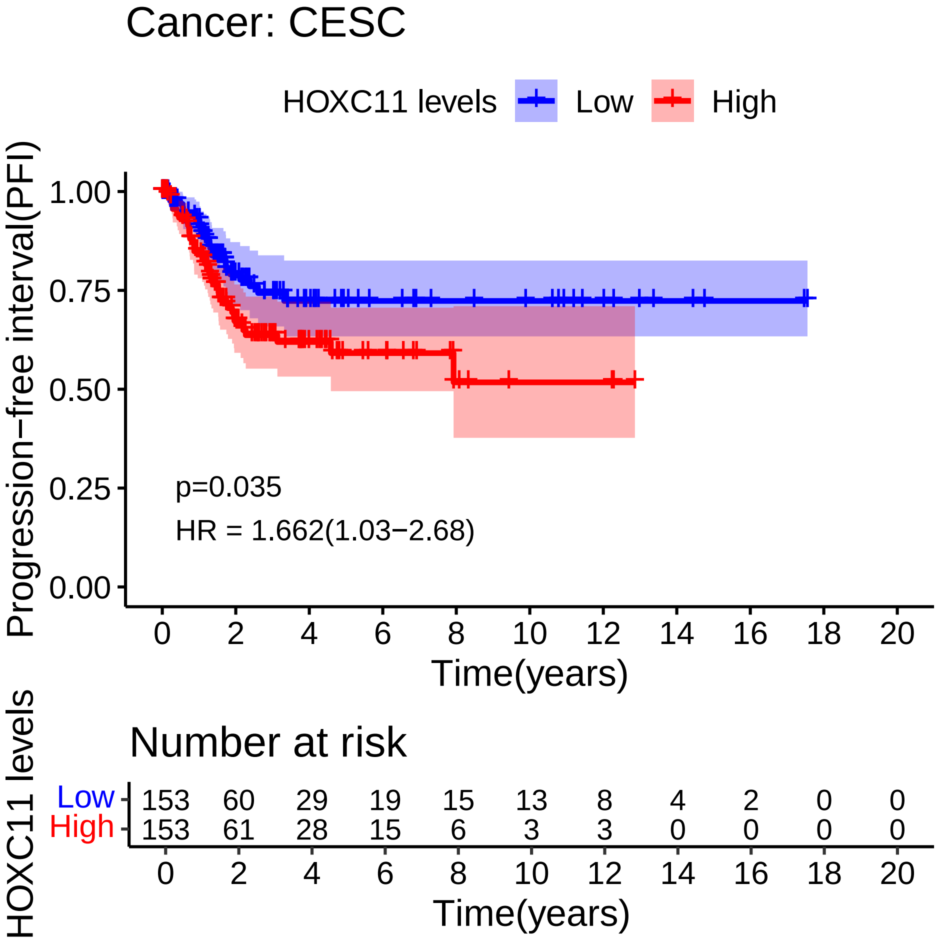

Cancer: CESC
HOXC11 levels
Low
High
Progression−free interval(PFI)
1.00
0.75
0.50
0.25
0.00
p=0.035
HR = 1.662(1.03−2.68)
0 2 4 6 8 10 12 14 16 18 20
Time(years)
Number at risk
HOXC11 levels
Low 153 60 29 19 15 13 8 4 2 0 0
High 153 61 28 15 6 3 3 0 0 0 0
0 2 4 6 8 10 12 14 16 18 20
Time(years)

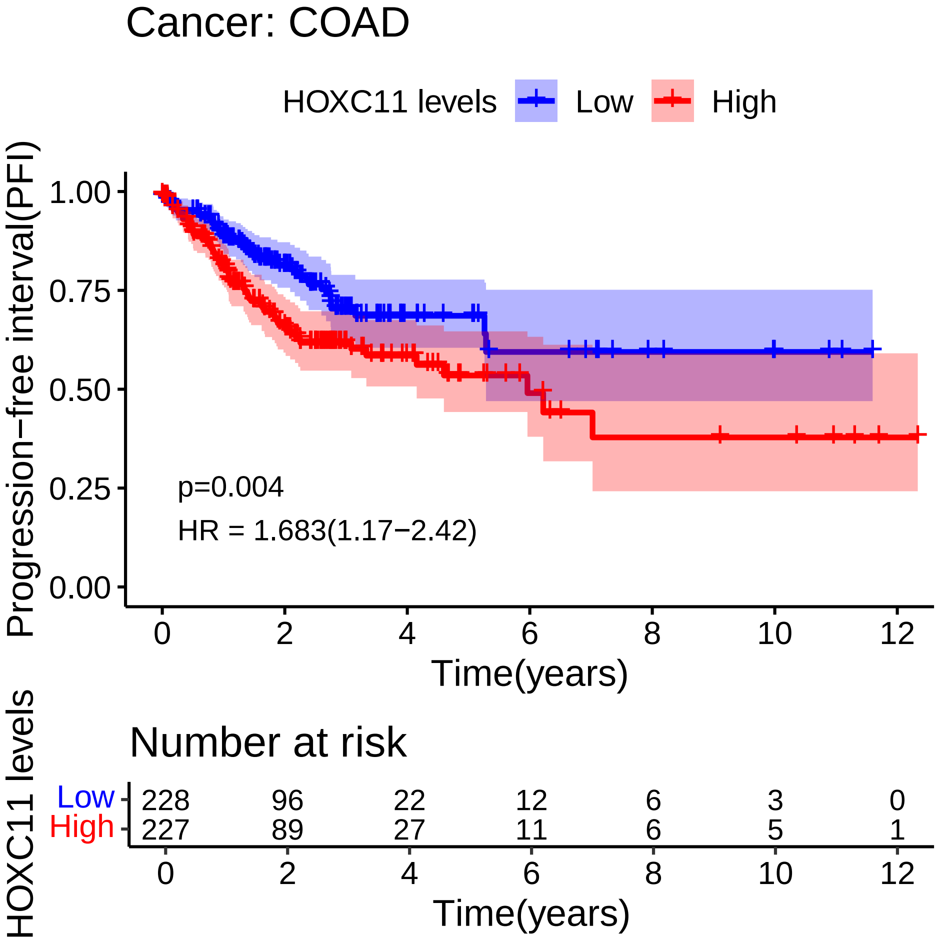

Cancer: COAD
HOXC11 levels
Low
High
Progression−free interval(PFI)
1.00
0.75
0.50
0.25
0.00
p=0.004
HR = 1.683(1.17−2.42)
0
2
4
6
8
10
12
Time(years)
Number at risk
HOXC11 levels
Low 228 96 22 12 6 3 0
High 227 89 27 11 6 5 1
Time(years)

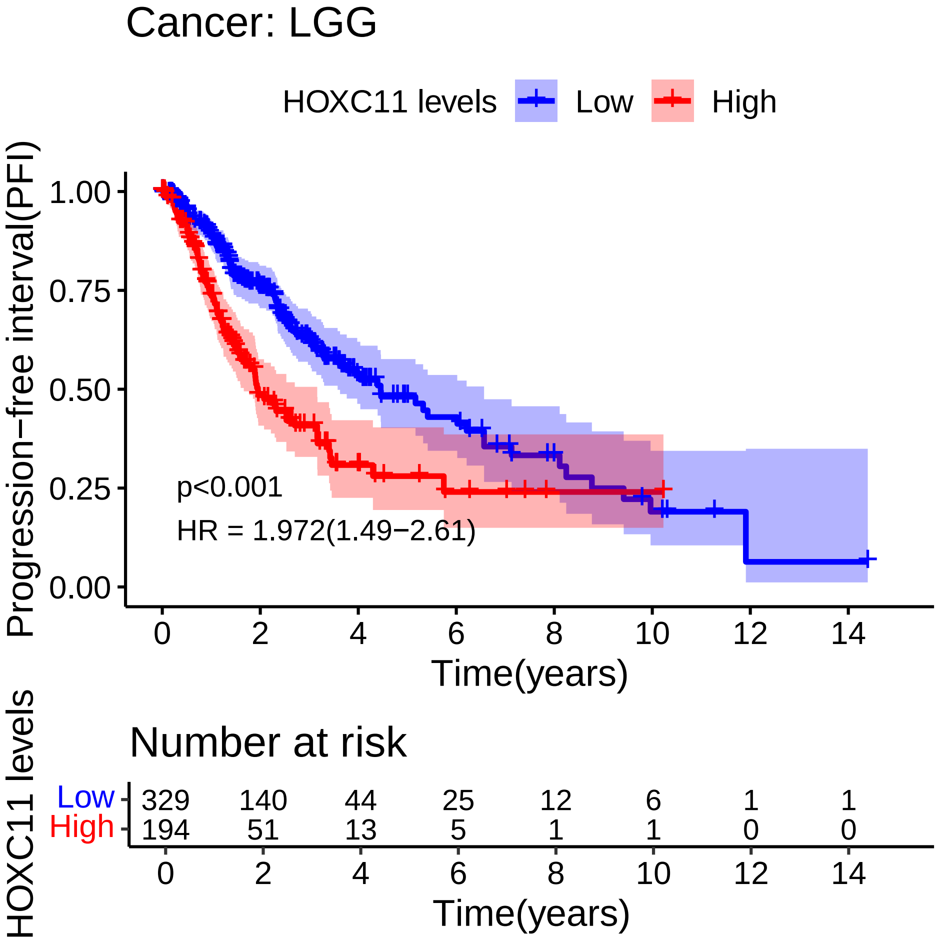

Cancer: LGG
HOXC11 levels
Low
High
Progression−free interval(PFI)
1.00
0.75
0.50
0.25
0.00
p<0.001
HR = 1.972(1.49−2.61)
0
2
4
6
8
10
12
14
Time(years)
Number at risk
HOXC11 levels
Low
High
329 140 44 25 12 6 1 1
194 51 13 5 1 1 0 0
0
2
4
6
8
10
12
14
Time(years)

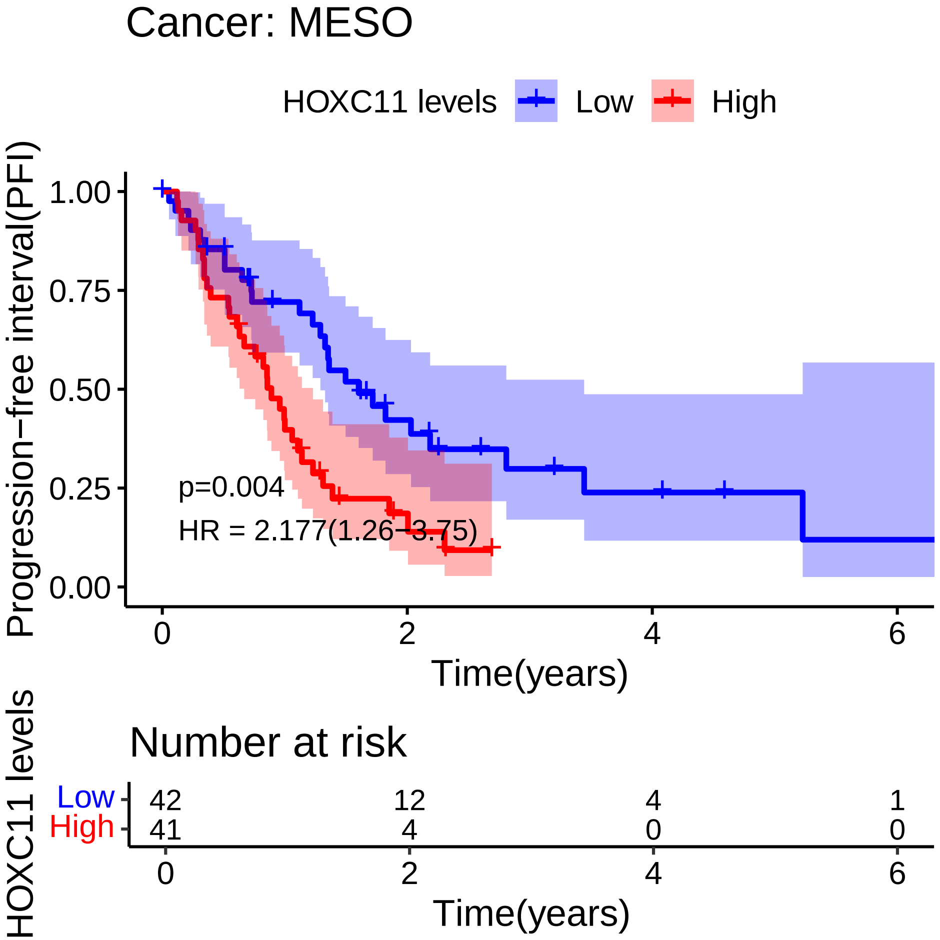
Cancer: MESO
HOXC11 levels
Low
High
Progression−free interval(PFI)
1.00
0.75
0.50
0.25
0.00
p=0.004
HR = 2.177(1.26−3.75)
0
2
4
6
Time(years)
Number at risk
HOXC11 levels
Low
High
42
12
4
1
41
4
0
0
0
2
4
6
Time(years)

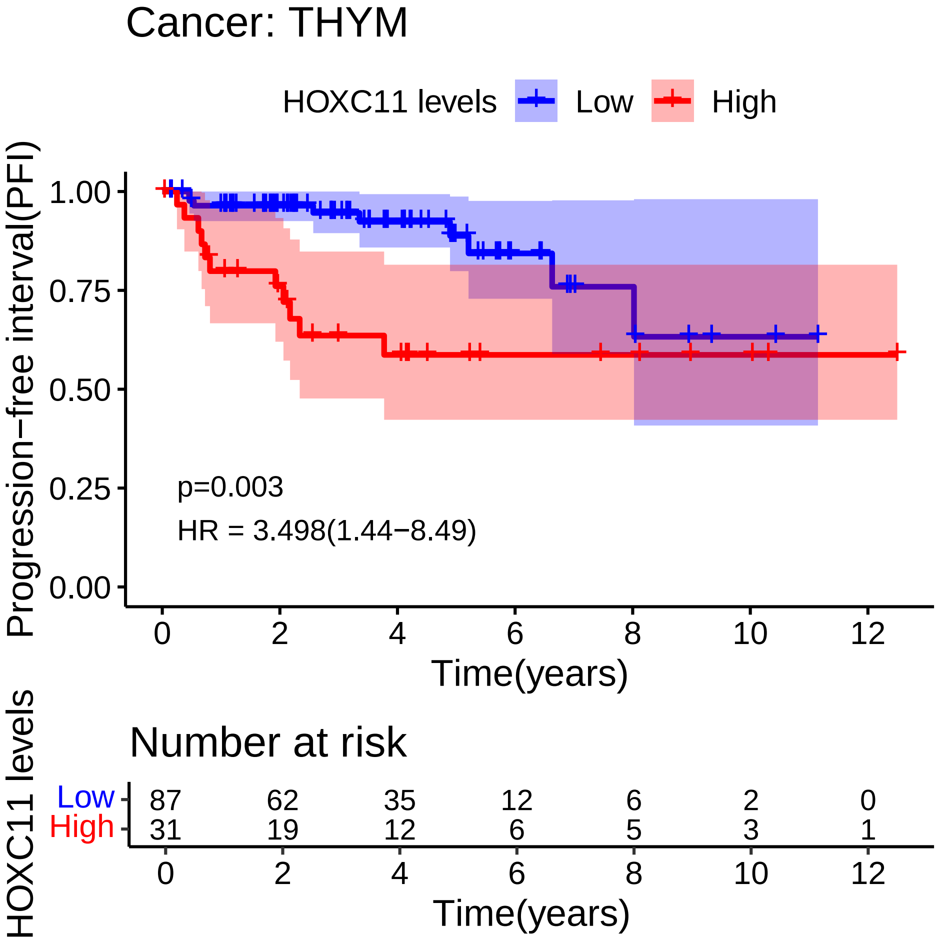
Cancer: THYM
HOXC11 levels
Low
High
Progression−free interval(PFI)
1.00
0.75
0.50
0.25
0.00
p=0.003
HR = 3.498(1.44−8.49)
0
2
4
6
8
10
12
Time(years)
Number at risk
HOXC11 levels
Low 87 62 35 12 6 2 0
High 31 19 12 6 5 3 1
0
2
4
6
8
10
12
Time(years)

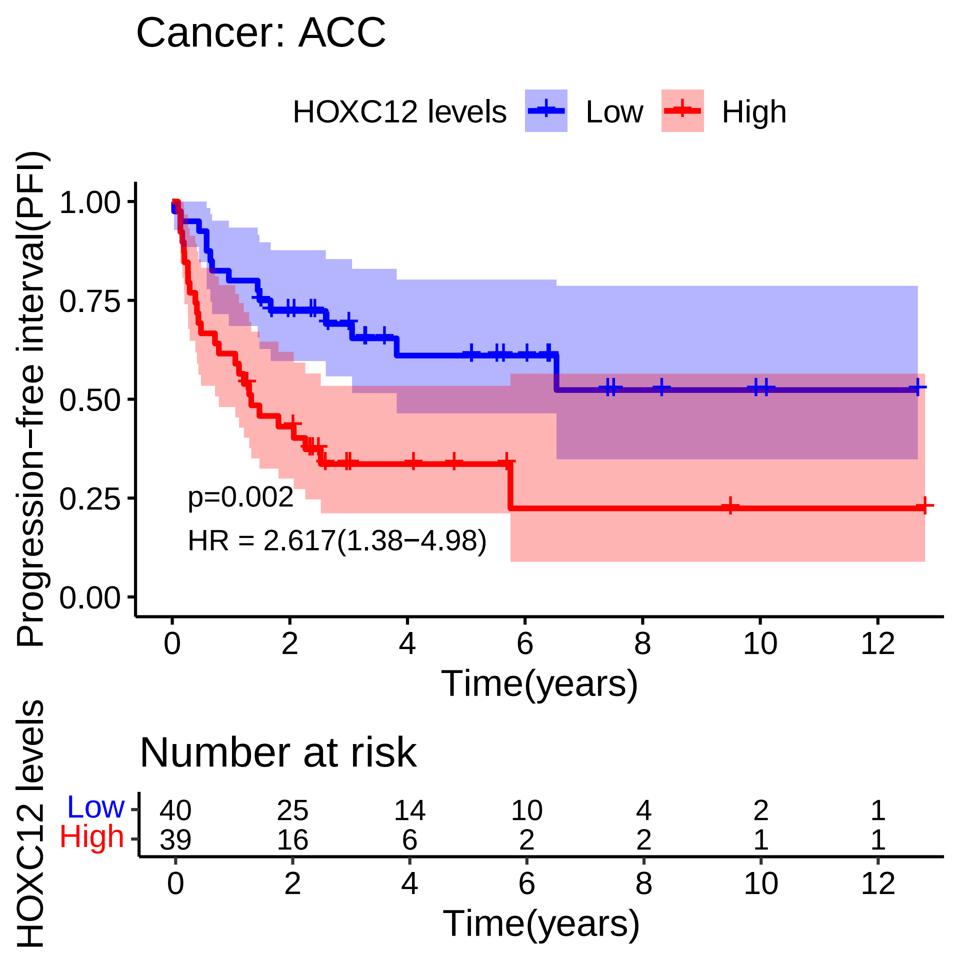

Cancer: ACC
HOXC12 levels
Low
High
Progression−free interval(PFI)
1.00
0.75
0.50
0.25
0.00
p=0.002
HR = 2.617(1.38−4.98)
0
2
4
6
8
10
12
Time(years)
Number at risk
HOXC12 levels
Low
High
40 25 14 10 4 2 1
39 16 6 2 2 1 1
Time(years)

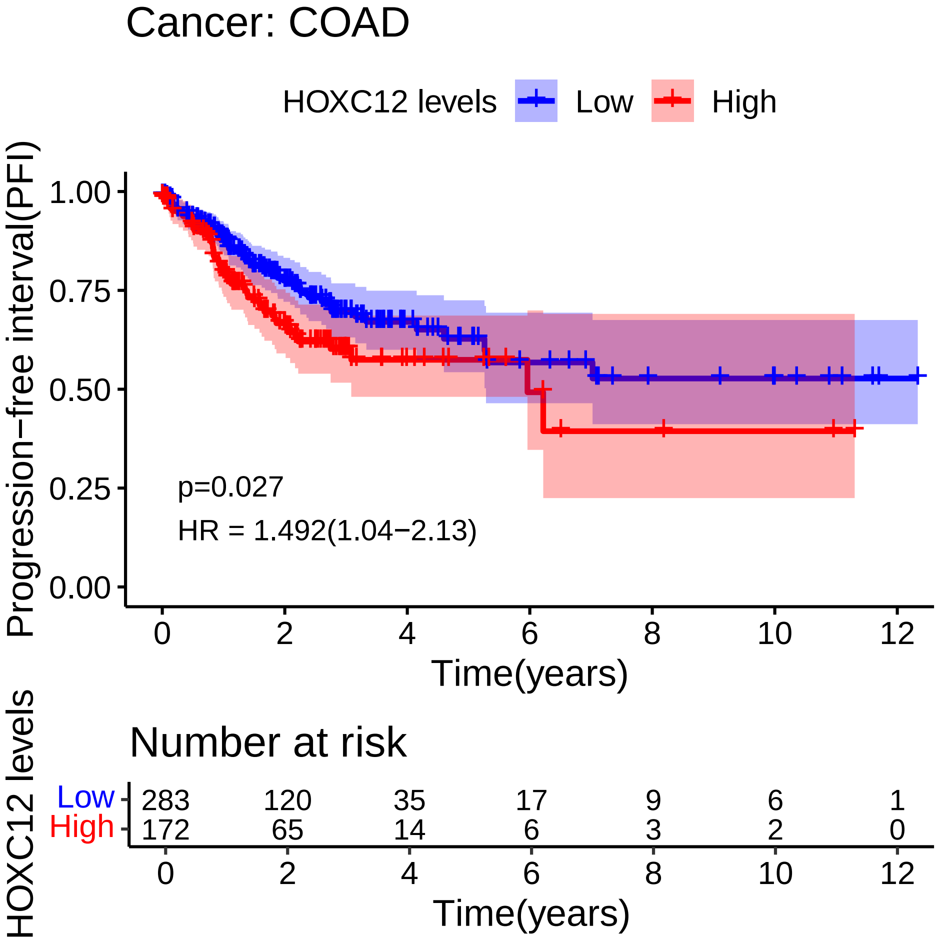

Cancer: COAD
HOXC12 levels
Low
High
Progression−free interval(PFI)
1.00
0.75
0.50
0.25
0.00
p=0.027
HR = 1.492(1.04−2.13)
0
2
4
6
8
10
12
Time(years)
Number at risk
HOXC12 levels
Low
High
283 120 35 17 9 6 1
172 65 14 6 3 2 0
Time(years)

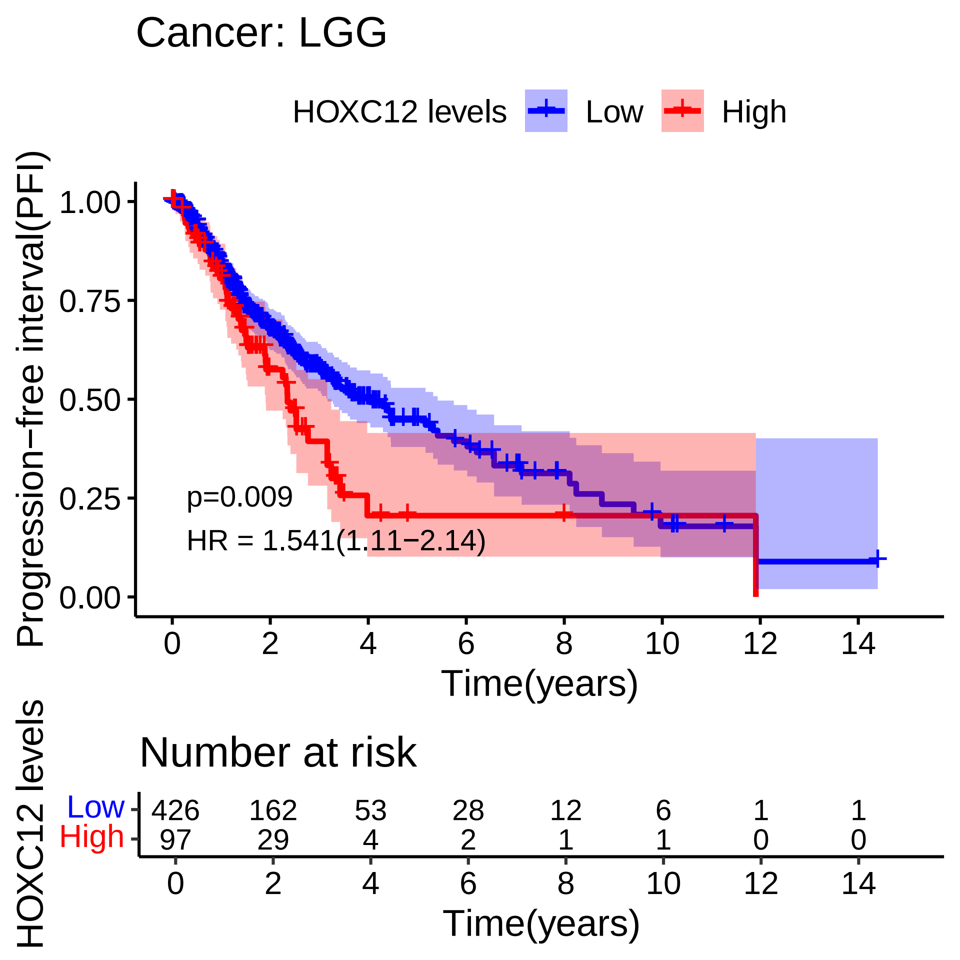

Cancer: LGG
HOXC12 levels
Low
High
Progression−free interval(PFI)
1.00
0.75
0.50
0.25
0.00
p=0.009
HR = 1.541(1.11−2.14)
0
2
4
6
8
10
12
14
Time(years)
Number at risk
HOXC12 levels
Low 426 162 53 28 12 6 1 1
High 97 29 4 2 1 1 0 0
0
2
4
6
8
10
12
14
Time(years)

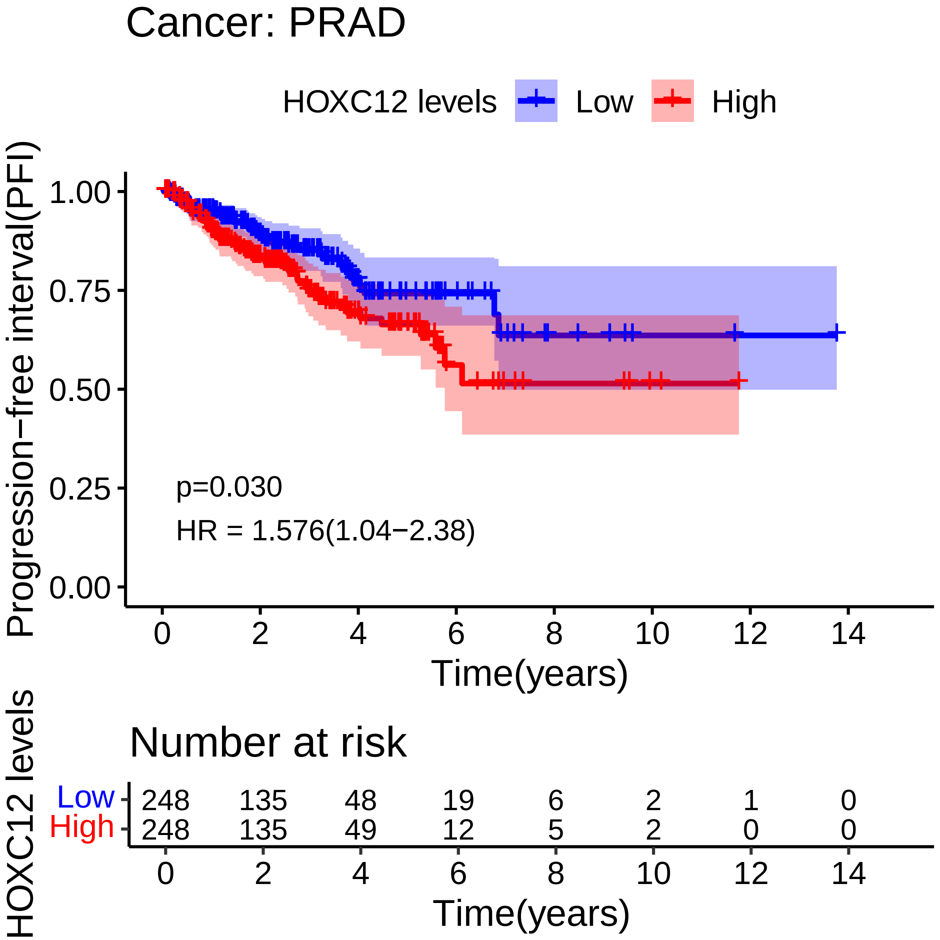

Cancer: PRAD
HOXC12 levels
Low
High
Progression−free interval(PFI)
1.00
0.75
0.50
0.25
0.00
p=0.030
HR = 1.576(1.04−2.38)
0
2
4
6
8
10
12
14
Time(years)
Number at risk
HOXC12 levels
Low 248 135 48 19 6 2 1 0
High 248 135 49 12 5 2 0 0
Time(years)

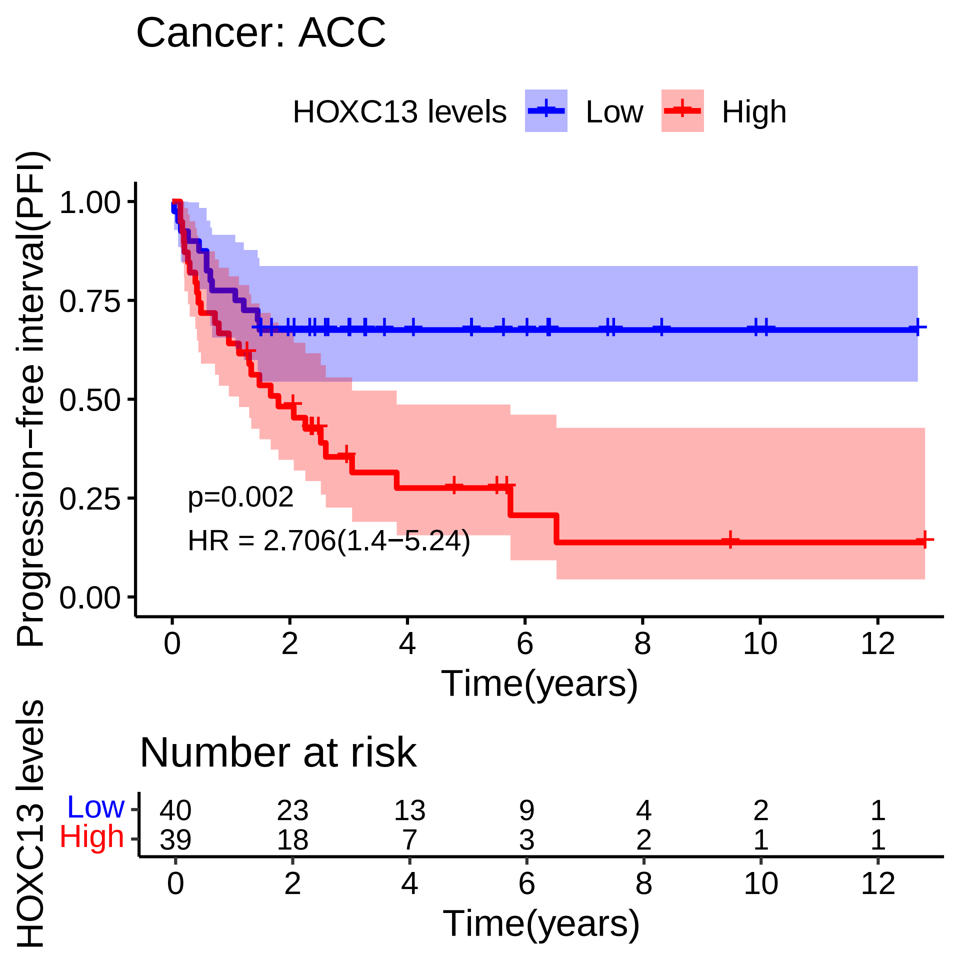

Cancer: ACC
HOXC13 levels
Low
High
Progression−free interval(PFI)
1.00
0.75
0.50
0.25
0.00
p=0.002
HR = 2.706(1.4−5.24)
0
2
4
6
8
10
12
Time(years)
Number at risk
HOXC13 levels
Low 40 23 13 9 4 2 1
High 39 18 7 3 2 1 1
0
2
4
6
8
10
12
Time(years)

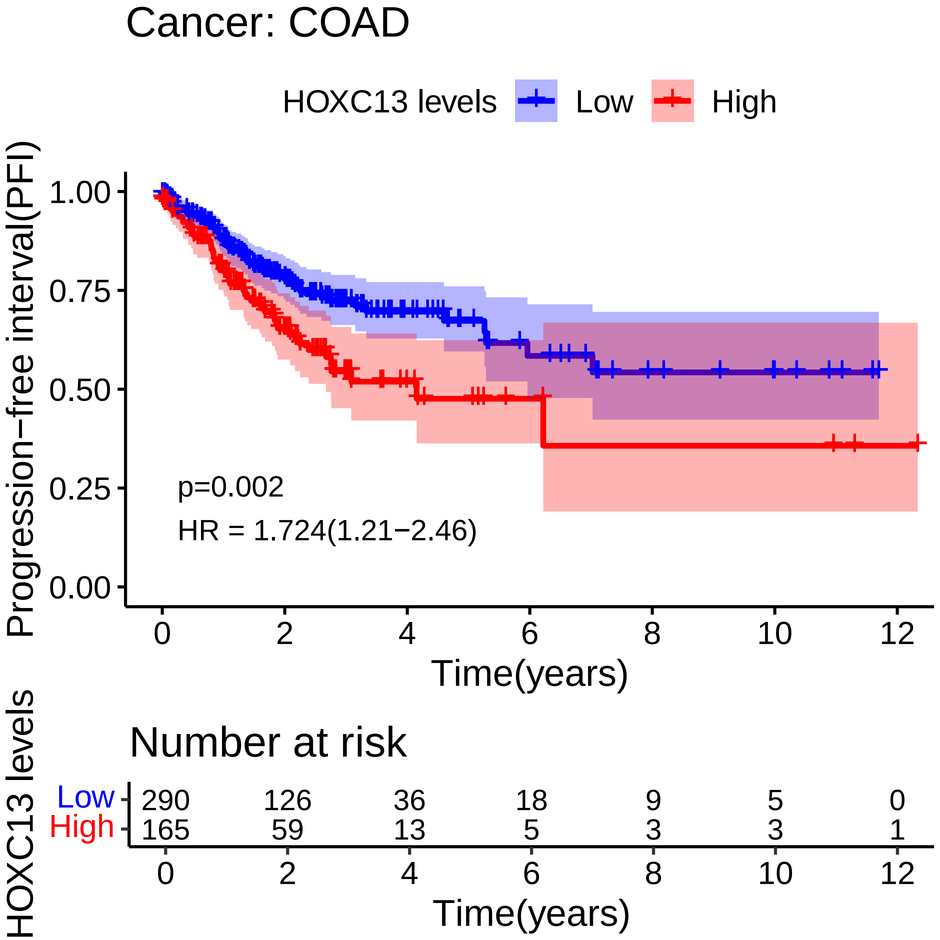
Cancer: COAD
HOXC13 levels
Low
High
Progression−free interval(PFI)
1.00
0.75
0.50
0.25
0.00
p=0.002
HR = 1.724(1.21−2.46)
0
2
4
6
8
10
12
Time(years)
Number at risk
HOXC13 levels
Low
High
290 126 36 18 9 5 0
165 59 13 5 3 3 1
Time(years)

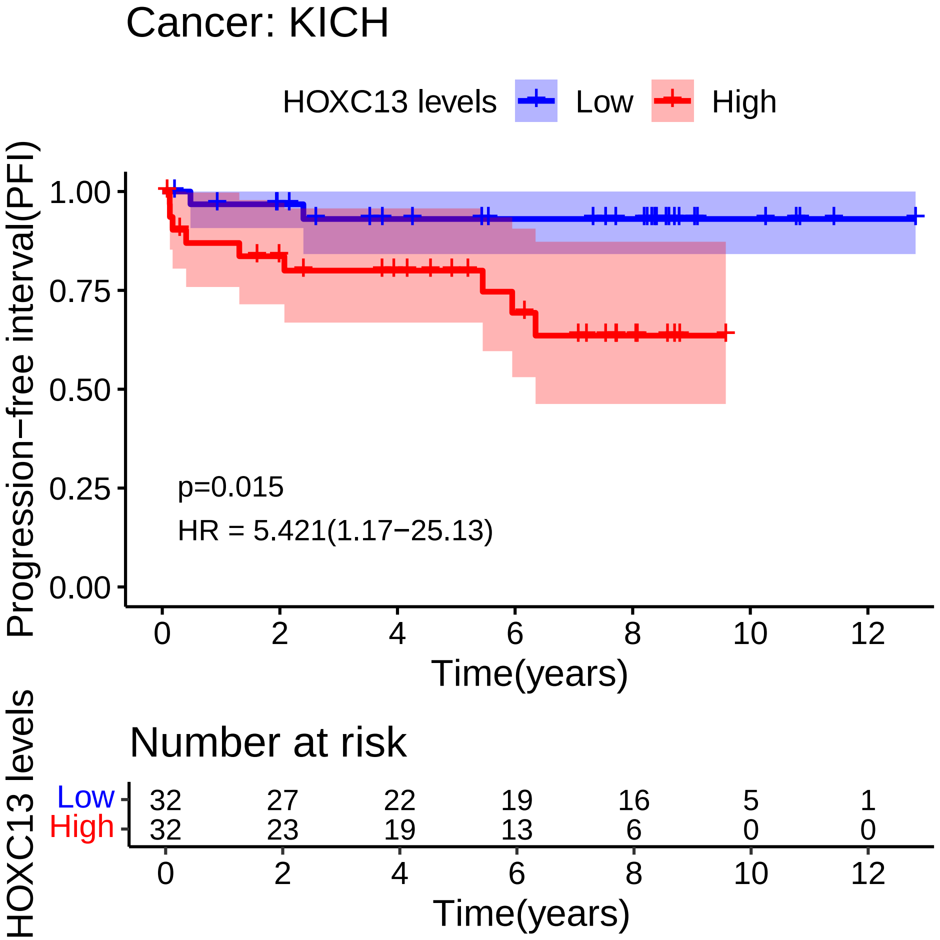

Cancer: KICH
HOXC13 levels
Low
High
Progression−free interval(PFI)
1.00
0.75
0.50
0.25
0.00
p=0.015
HR = 5.421(1.17−25.13)
0
2
4
6
8
10
12
Time(years)
Number at risk
HOXC13 levels
Low 32 27 22 19 16 5 1
High 32 23 19 13 6 0 0
Time(years)

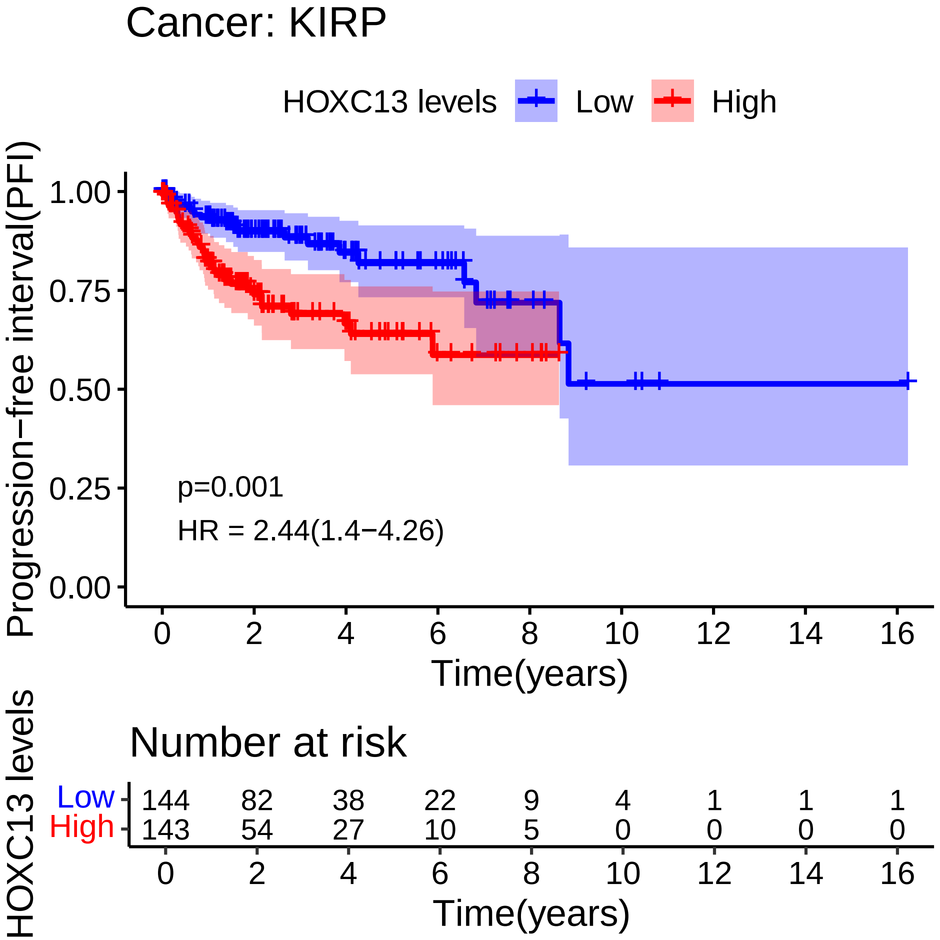

Cancer: KIRP
HOXC13 levels
Low
High
Progression−free interval(PFI)
1.00
0.75
0.50
0.25
0.00
p=0.001
HR = 2.44(1.4−4.26)
0
2
4
6
8
10
12
14
16
Time(years)
Number at risk
HOXC13 levels
Low 144 82 38 22 9 4 1 1 1
High 143 54 27 10 5 0 0 0 0
Time(years)

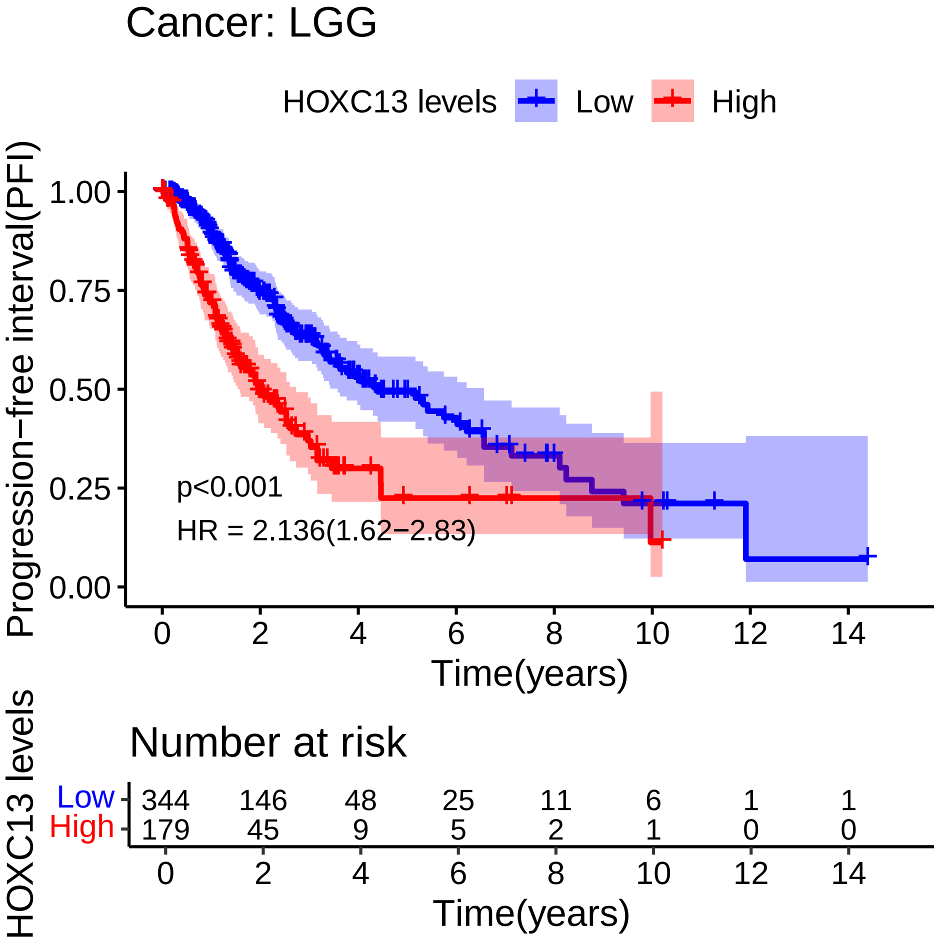
Cancer: LGG
HOXC13 levels
Low
High
Progression−free interval(PFI)
1.00
0.75
0.50
0.25
0.00
p<0.001
HR = 2.136(1.62−2.83)
0
2
4
6
8
10
12
14
Time(years)
Number at risk
HOXC13 levels
Low 344 146 48 25 11 6 1 1
High 179 45 9 5 2 1 0 0
Time(years)

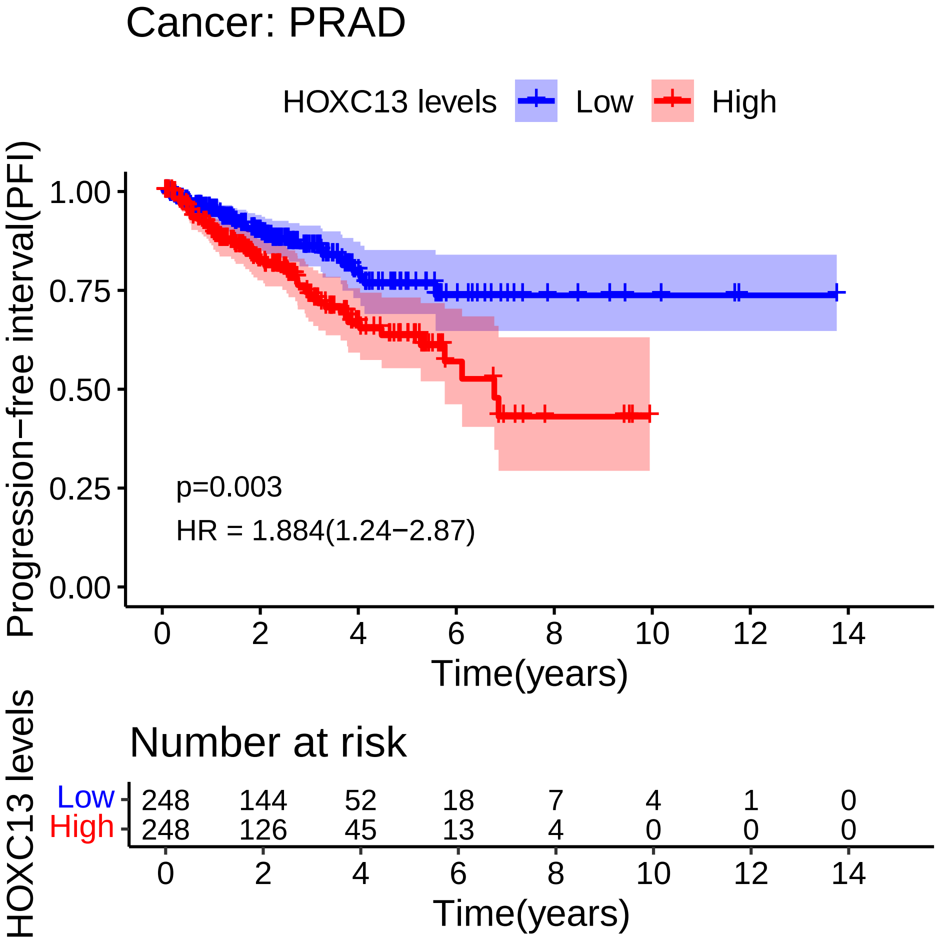

Cancer: PRAD
HOXC13 levels
Low
High
Progression−free interval(PFI)
1.00
0.75
0.50
0.25
0.00
p=0.003
HR = 1.884(1.24−2.87)
0
2
4
6
8
10
12
14
Time(years)
Number at risk
HOXC13 levels
Low 248 144 52 18 7 4 1 0
High 248 126 45 13 4 0 0 0
Time(years)

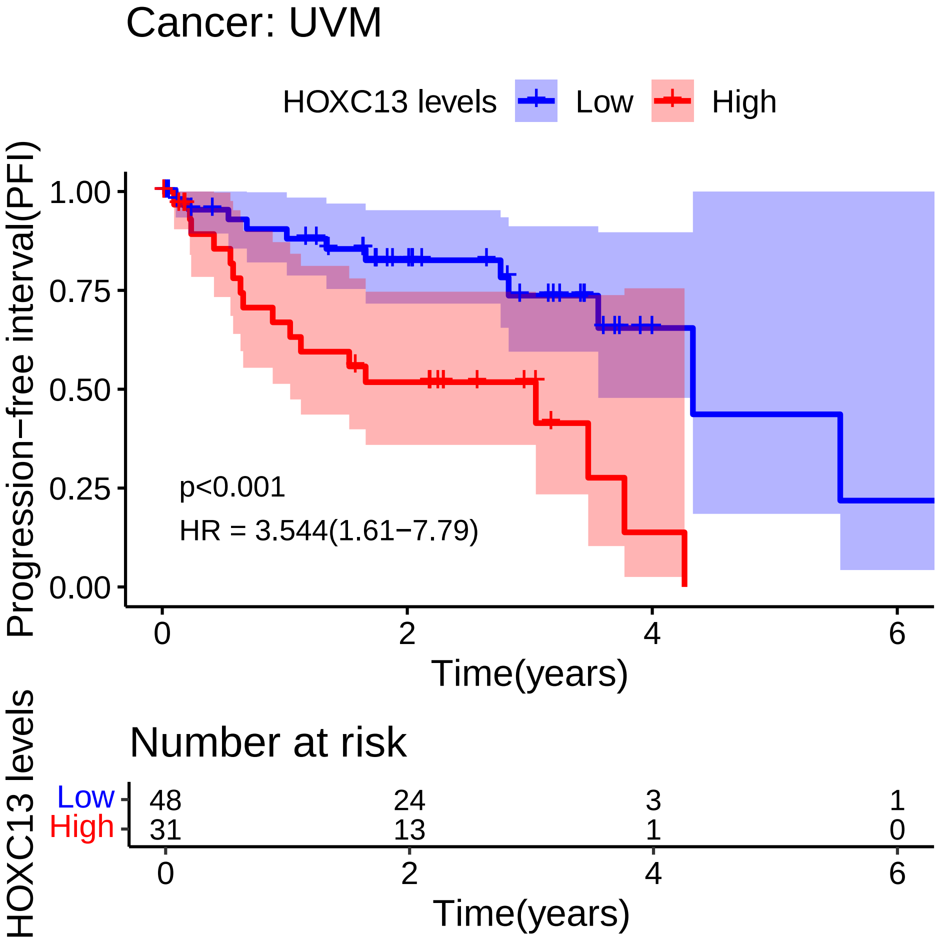
Cancer: UVM
HOXC13 levels
Low
High
Progression−free interval(PFI)
1.00
0.75
0.50
0.25
0.00
p<0.001
HR = 3.544(1.61−7.79)
0
2
4
6
Time(years)
Number at risk
HOXC13 levels
Low
High
48
24
3
1
31
13
1
0
Time(years)

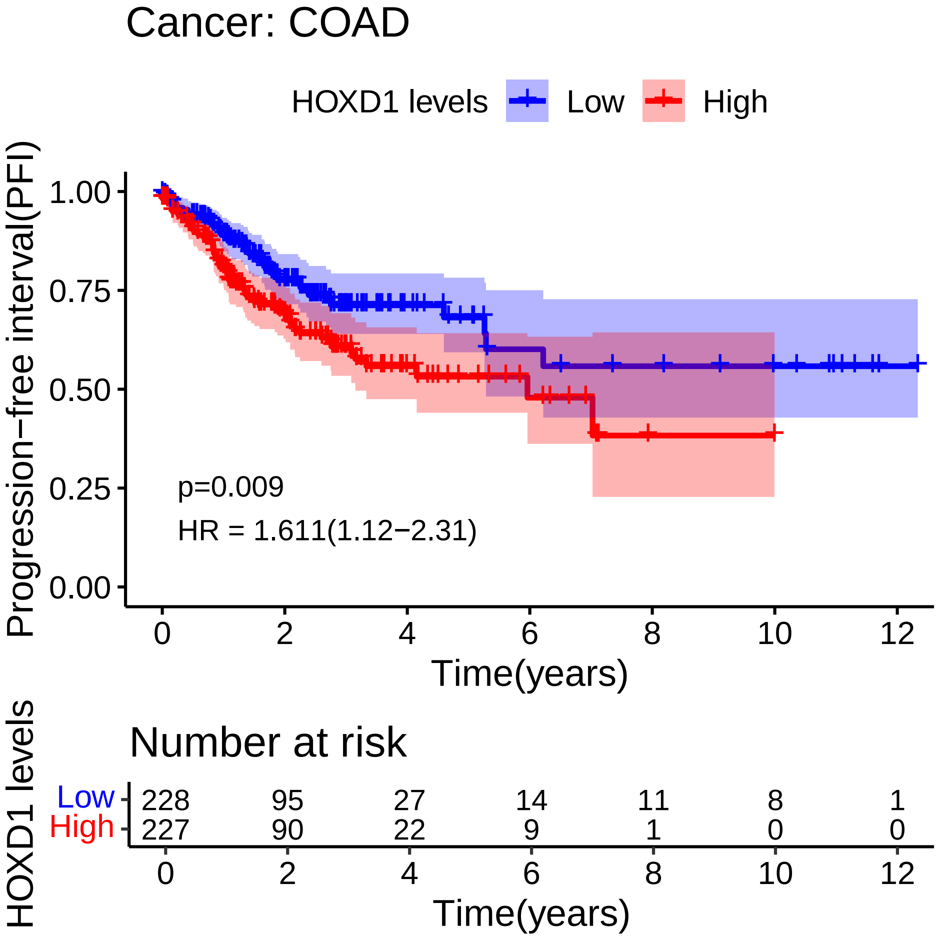
Cancer: COAD
HOXD1 levels
Low
High
Progression−free interval(PFI)
1.00
0.75
0.50
0.25
0.00
p=0.009
HR = 1.611(1.12−2.31)
0
2
4
6
8
10
12
Time(years)
Number at risk
HOXD1 levels
Low
High
228 95 27 14 11 8 1
227 90 22 9 1 0 0
0
2
4
6
8
10
12
Time(years)

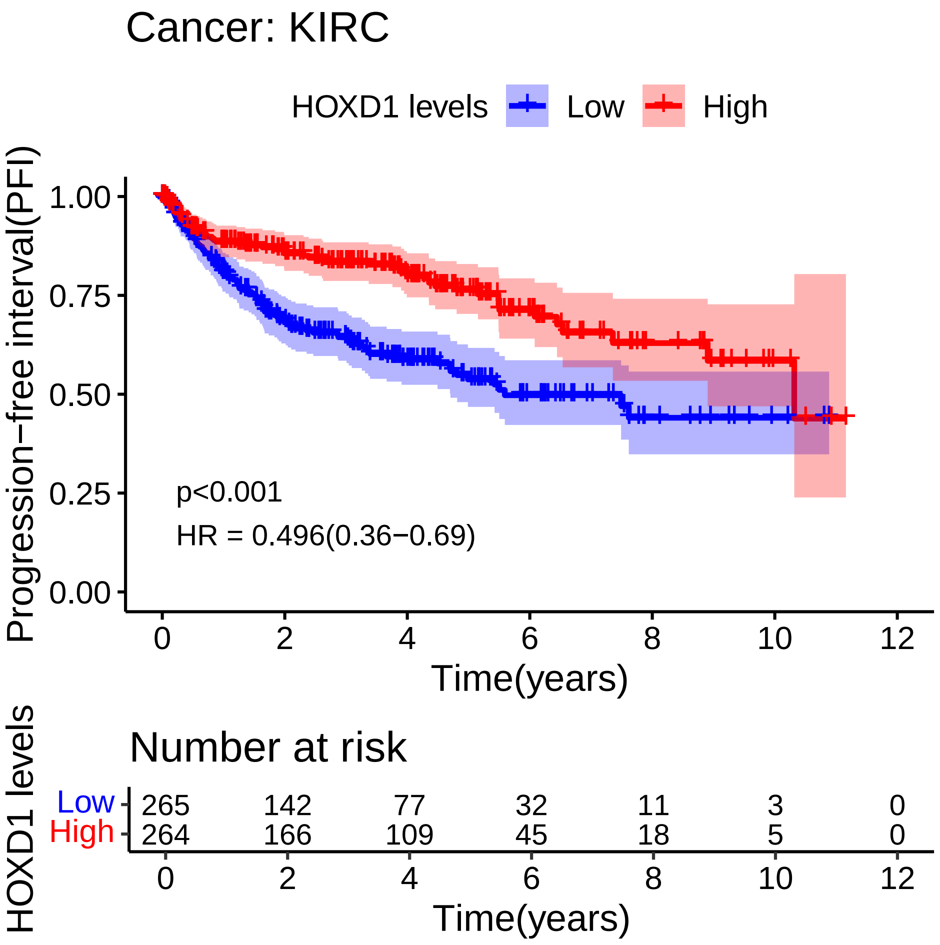

Cancer: KIRC
HOXD1 levels
Low
High
Progression−free interval(PFI)
1.00
0.75
0.50
0.25
0.00
p<0.001
HR = 0.496(0.36−0.69)
0
2
4
6
8
10
12
Time(years)
Number at risk
HOXD1 levels
Low 265 142 77 32 11 3 0
High 264 166 109 45 18 5 0
Time(years)

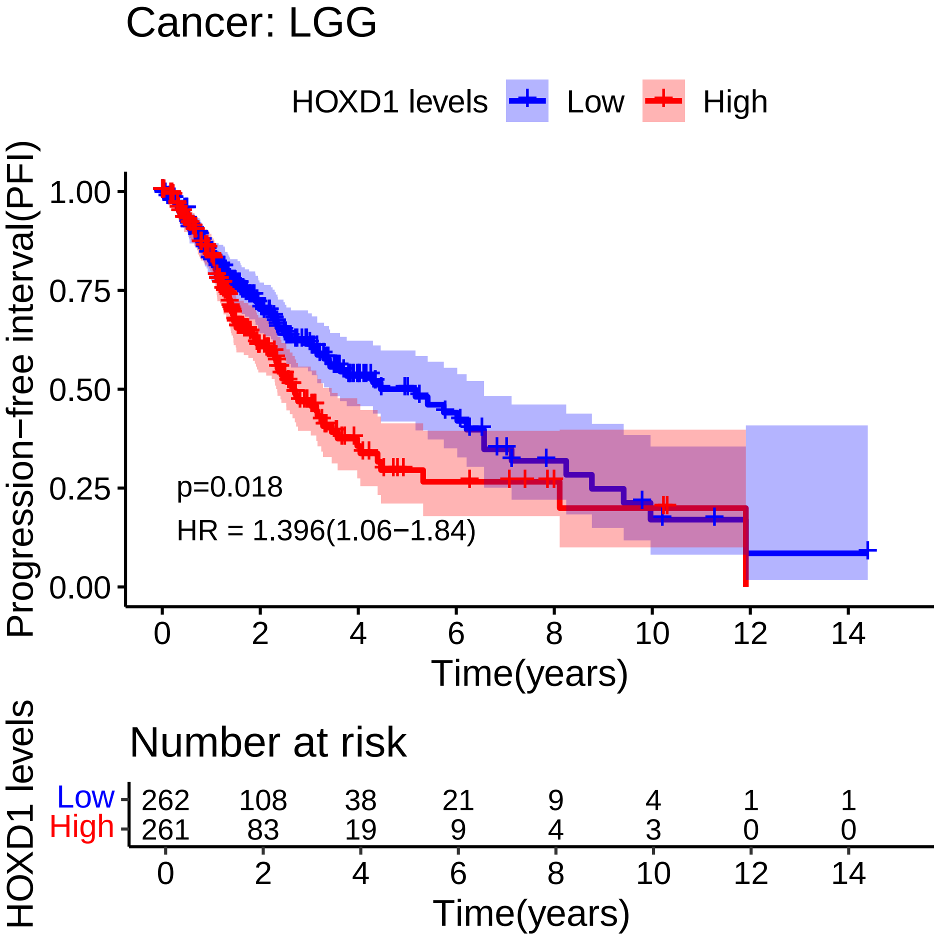
Cancer: LGG
HOXD1 levels
Low
High
Progression−free interval(PFI)
1.00
0.75
0.50
0.25
0.00
p=0.018
HR = 1.396(1.06−1.84)
0
2
4
6
8
10
12
14
Time(years)
Number at risk
HOXD1 levels
Low 262 108 38 21 9 4 1 1
High 261 83 19 9 4 3 0 0
0
2
4
6
8
10
12
14
Time(years)

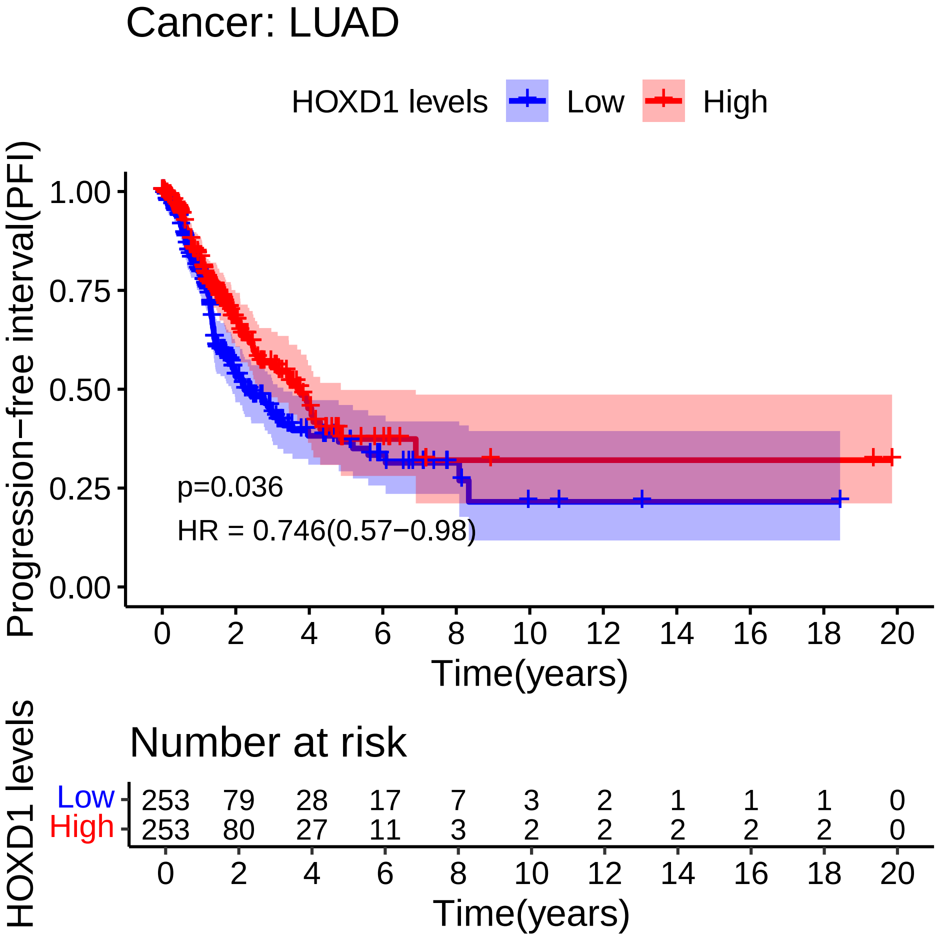
Cancer: LUAD
HOXD1 levels
Low
High
Progression−free interval(PFI)
1.00
0.75
0.50
0.25
0.00
p=0.036
HR = 0.746(0.57−0.98)
0
2
4
6
8
10
12
14
16
18
20
Time(years)
Number at risk
HOXD1 levels
Low 253 79 28 17 7 3 2 1 1 1 0
High 253 80 27 11 3 2 2 2 2 2 0
Time(years)

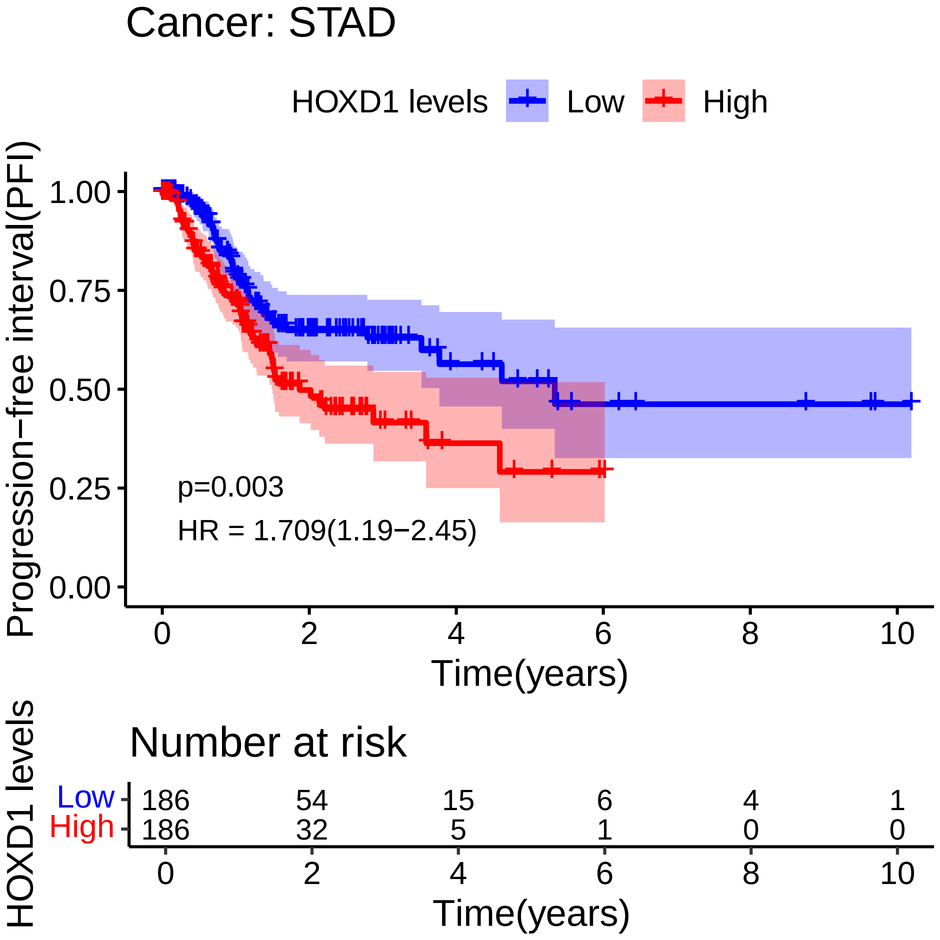
Cancer: STAD
HOXD1 levels
Low
High
Progression−free interval(PFI)
1.00
0.75
0.50
0.25
0.00
p=0.003
HR = 1.709(1.19−2.45)
0
2
4
6
8
10
Time(years)
Number at risk
HOXD1 levels
Low 186 54 15 6 4 1
High 186 32 5 1 0 0
0
2
4
6
8
10
Time(years)

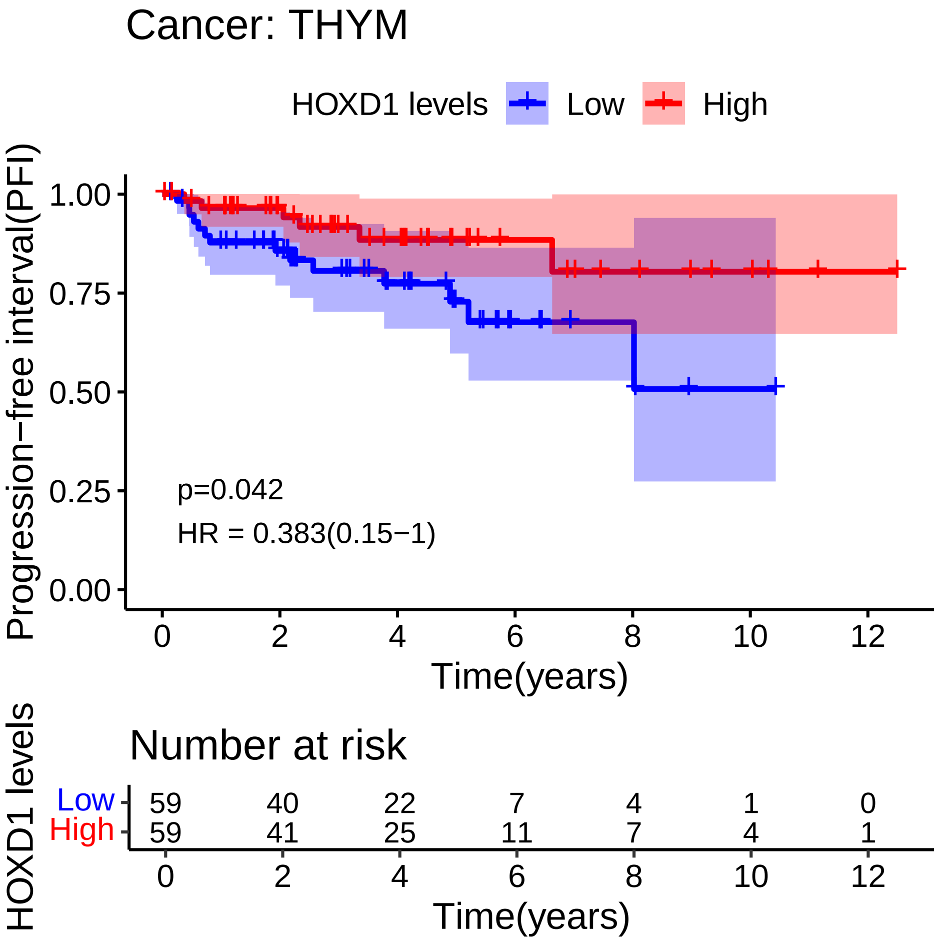

Cancer: THYM
HOXD1 levels
Low
High
Progression−free interval(PFI)
1.00
0.75
0.50
0.25
0.00
p=0.042
HR = 0.383(0.15−1)
0
2
4
6
8
10
12
Time(years)
Number at risk
HOXD1 levels
Low 59 40 22 7 4 1 0
High 59 41 25 11 7 4 1
0
2
4
6
8
10
12
Time(years)

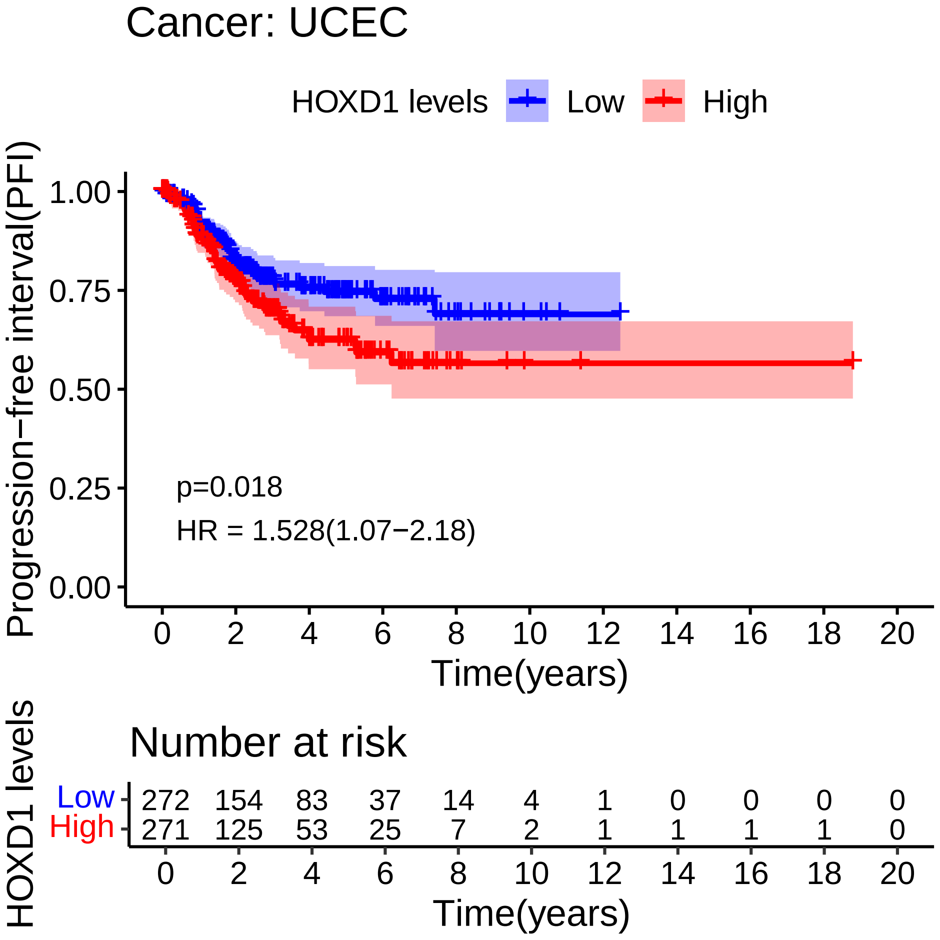
Cancer: UCEC
HOXD1 levels
Low
High
Progression−free interval(PFI)
1.00
0.75
0.50
0.25
0.00
p=0.018
HR = 1.528(1.07−2.18)
0 2 4 6 8 10 12 14 16 18 20
Time(years)
Number at risk
HOXD1 levels
Low 272 154 83 37 14 4 1 0 0 0 0
High 271 125 53 25 7 2 1 1 1 1 0
0 2 4 6 8 10 12 14 16 18 20
Time(years)

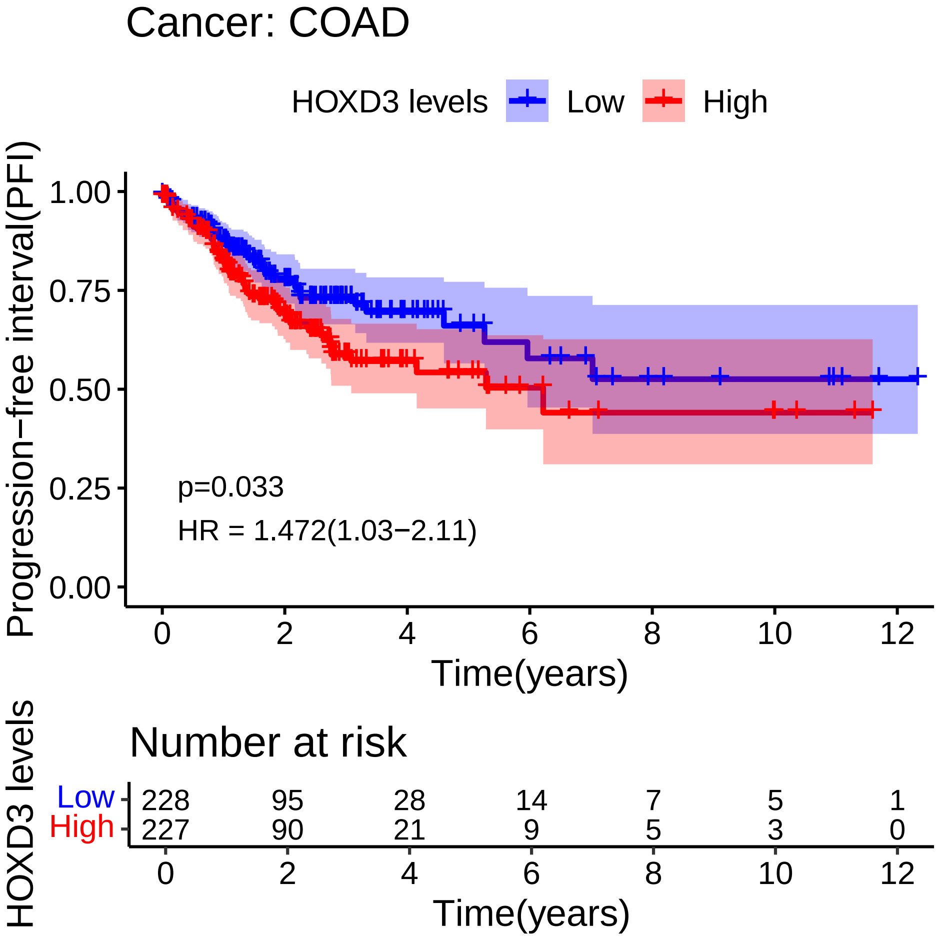

Cancer: COAD
HOXD3 levels
Low
High
Progression−free interval(PFI)
1.00
0.75
0.50
0.25
0.00
p=0.033
HR = 1.472(1.03−2.11)
0
2
4
6
8
10
12
Time(years)
Number at risk
HOXD3 levels
Low
High
228 95 28 14 7 5 1
227 90 21 9 5 3 0
Time(years)

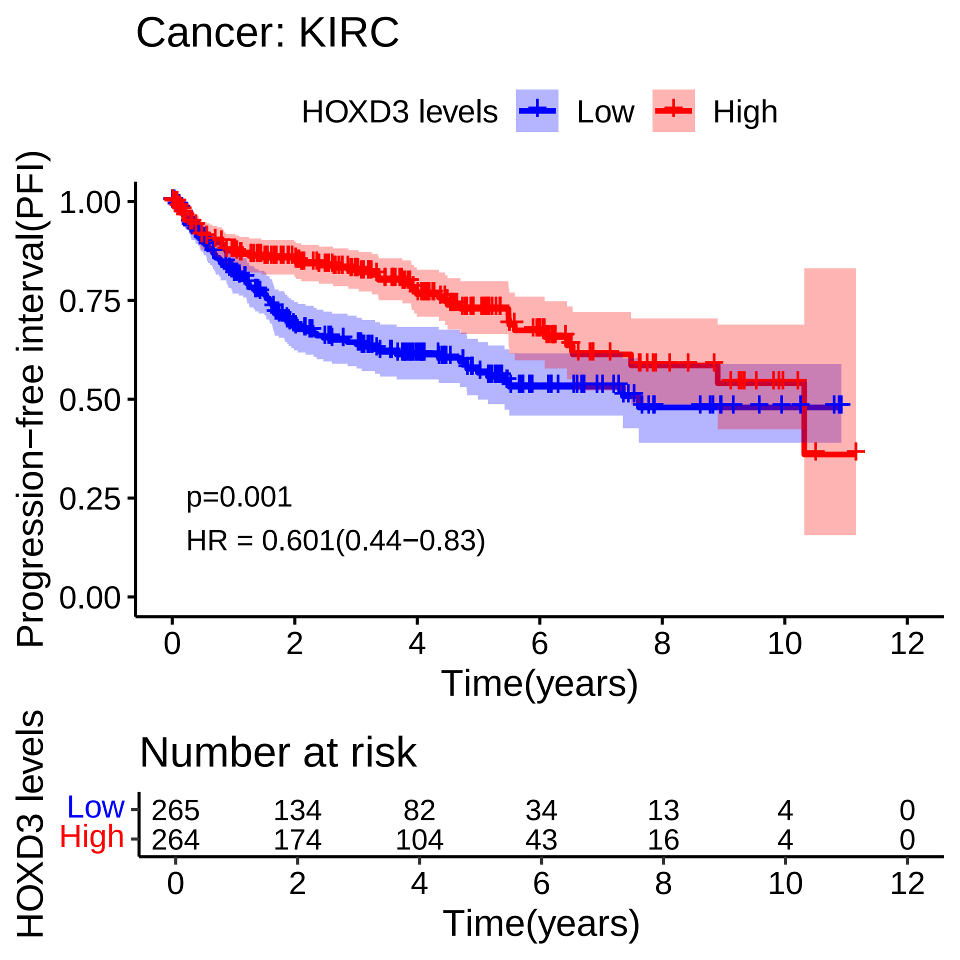

Cancer: KIRC
HOXD3 levels
Low
High
Progression−free interval(PFI)
1.00
0.75
0.50
0.25
0.00
p=0.001
HR = 0.601(0.44−0.83)
0
2
4
6
8
10
12
Time(years)
Number at risk
HOXD3 levels
Low 265 134 82 34 13 4 0
High 264 174 104 43 16 4 0
Time(years)

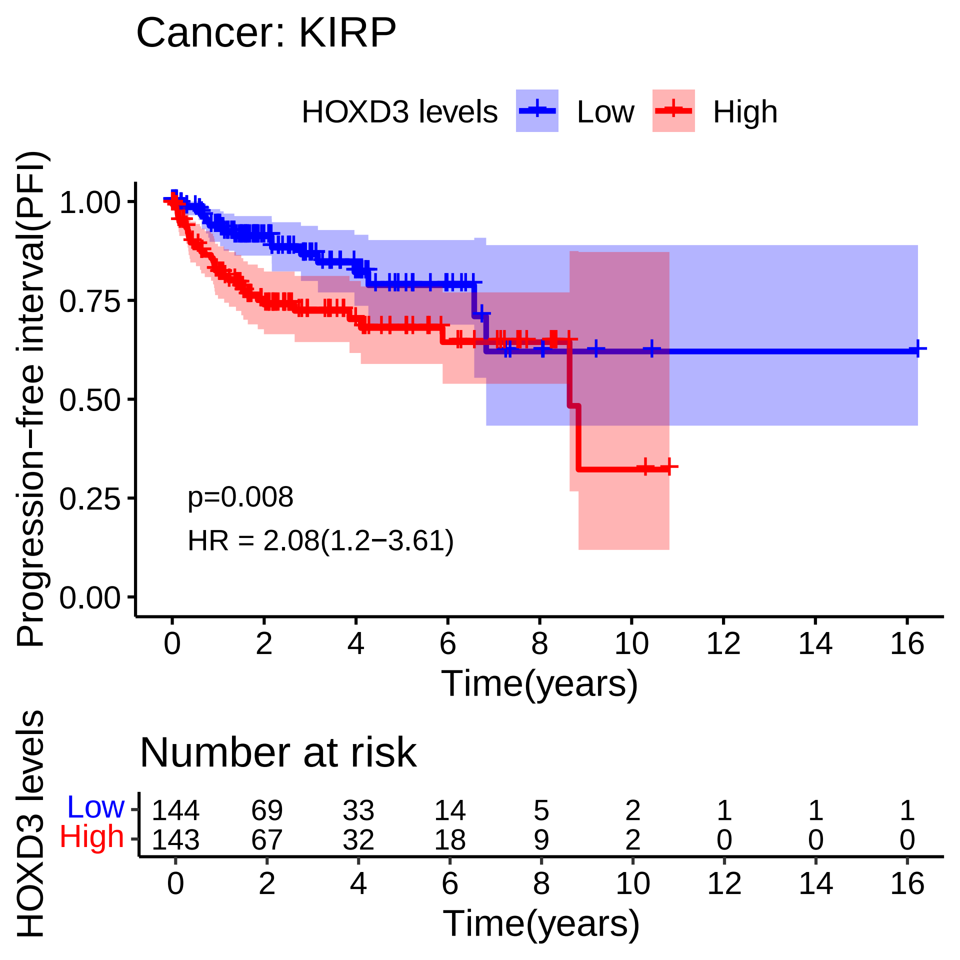
Cancer: KIRP
HOXD3 levels
Low
High
Progression−free interval(PFI)
1.00
0.75
0.50
0.25
0.00
p=0.008
HR = 2.08(1.2−3.61)
0
2
4
6
8
10
12
14
16
Time(years)
Number at risk
HOXD3 levels
Low 144 69 33 14 5 2 1 1 1
High 143 67 32 18 9 2 0 0 0
Time(years)

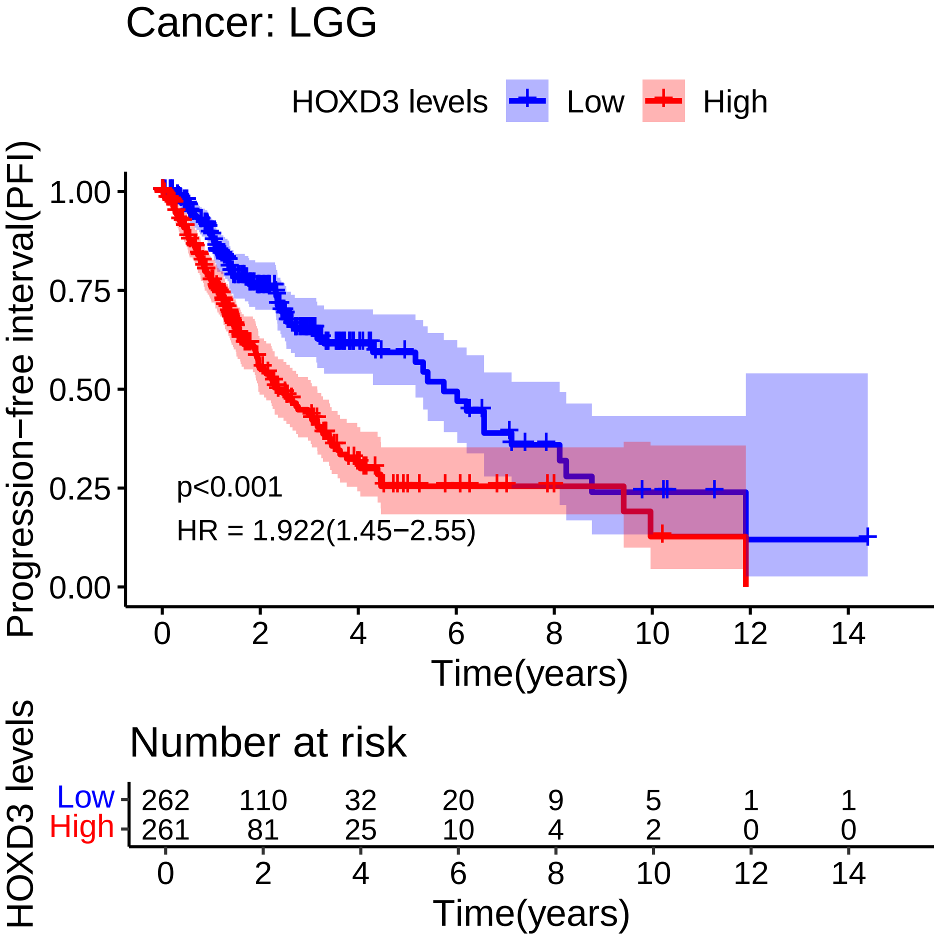

Cancer: LGG
HOXD3 levels
Low
High
Progression−free interval(PFI)
1.00
0.75
0.50
0.25
0.00
p<0.001
HR = 1.922(1.45−2.55)
0
2
4
6
8
10
12
14
Time(years)
Number at risk
HOXD3 levels
Low 262 110 32 20 9 5 1 1
High 261 81 25 10 4 2 0 0
Time(years)

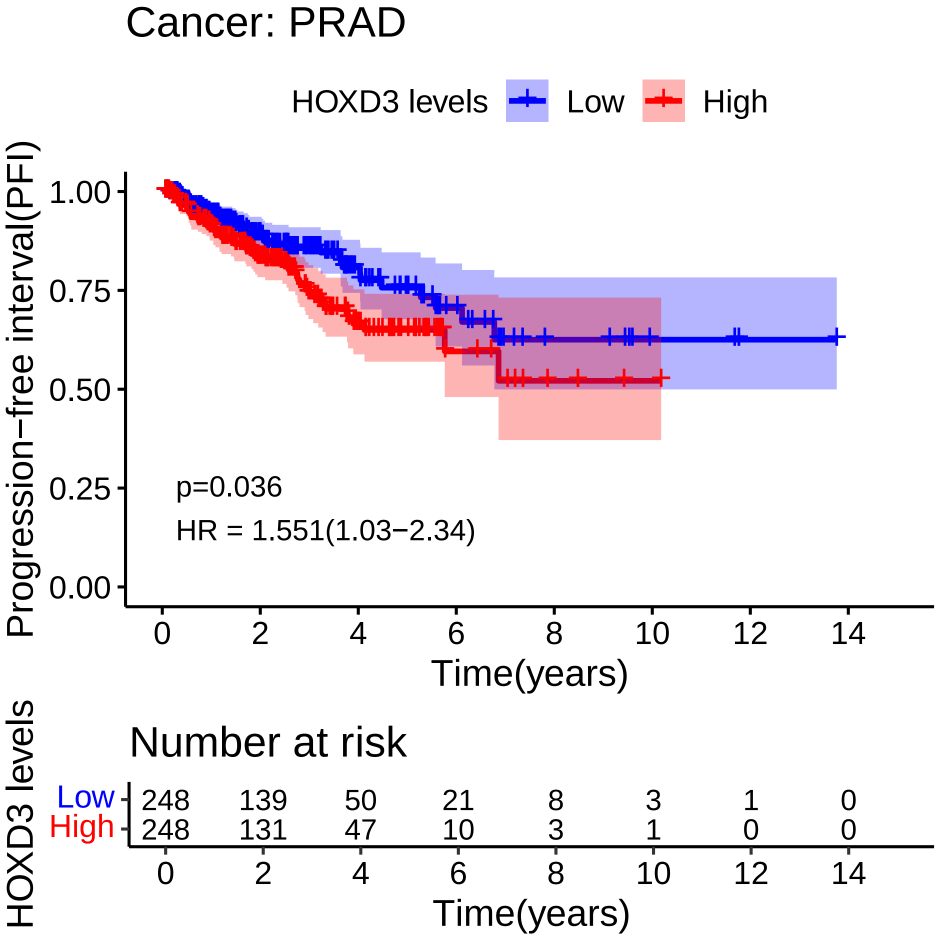
Cancer: PRAD
HOXD3 levels
Low
High
Progression−free interval(PFI)
1.00
0.75
0.50
0.25
0.00
p=0.036
HR = 1.551(1.03−2.34)
0
2
4
6
8
10
12
14
Time(years)
Number at risk
HOXD3 levels
Low 248 139 50 21 8 3 1 0
High 248 131 47 10 3 1 0 0
Time(years)

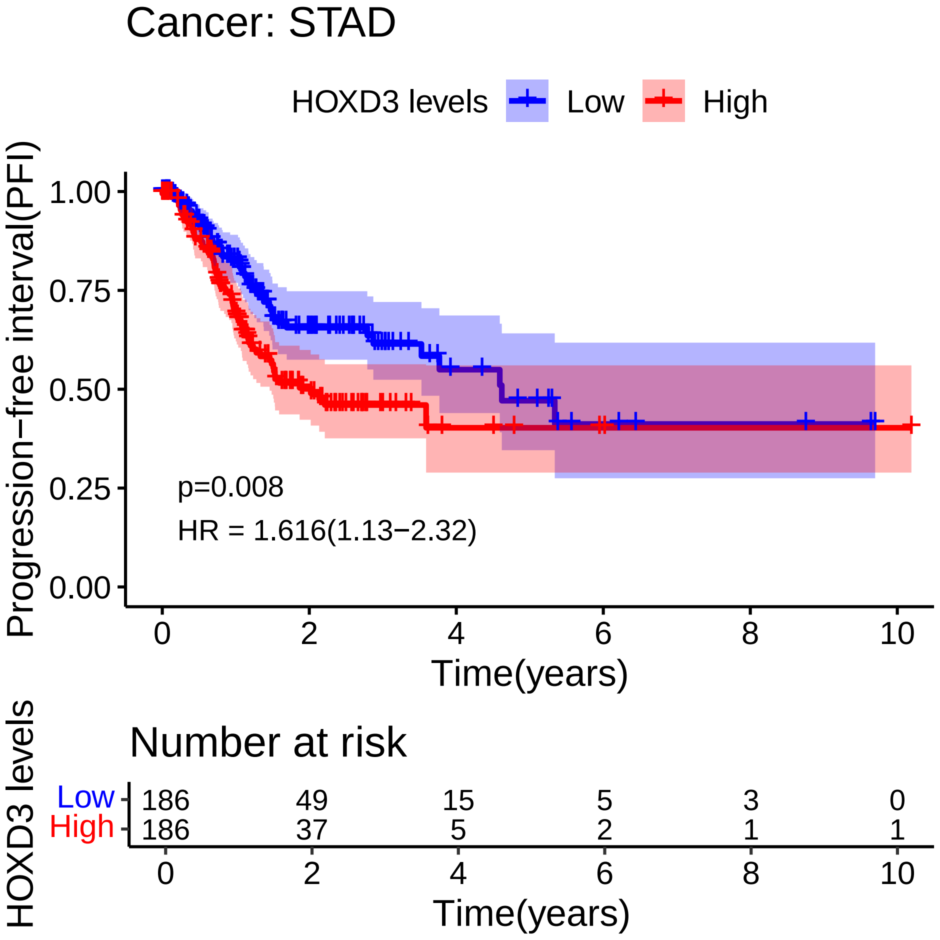
Cancer: STAD
HOXD3 levels
Low
High
Progression−free interval(PFI)
1.00
0.75
0.50
0.25
0.00
p=0.008
HR = 1.616(1.13−2.32)
0
2
4
6
8
10
Time(years)
Number at risk
HOXD3 levels
Low 186 49 15 5 3 0
High 186 37 5 2 1 1
0
2
4
6
8
10
Time(years)

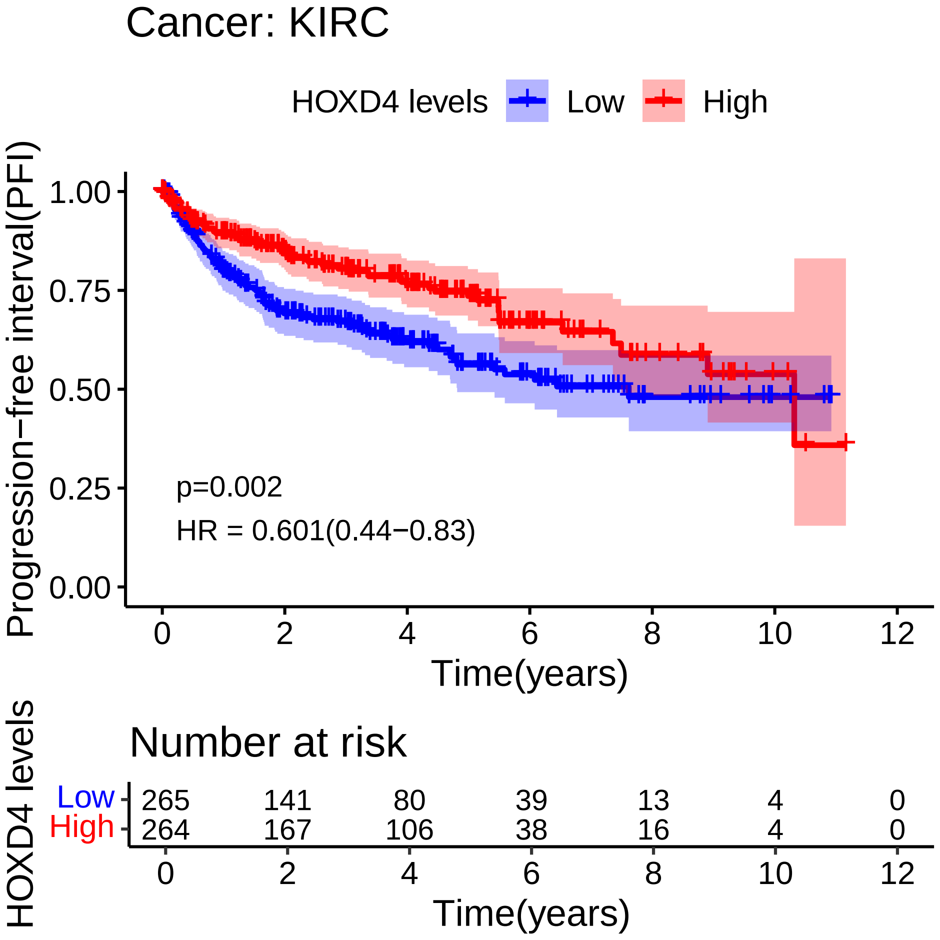
Cancer: KIRC
HOXD4 levels
Low
High
Progression−free interval(PFI)
1.00
0.75
0.50
0.25
0.00
p=0.002
HR = 0.601(0.44−0.83)
0
2
4
6
8
10
12
Time(years)
Number at risk
HOXD4 levels
Low 265 141 80 39 13 4 0
High 264 167 106 38 16 4 0
Time(years)

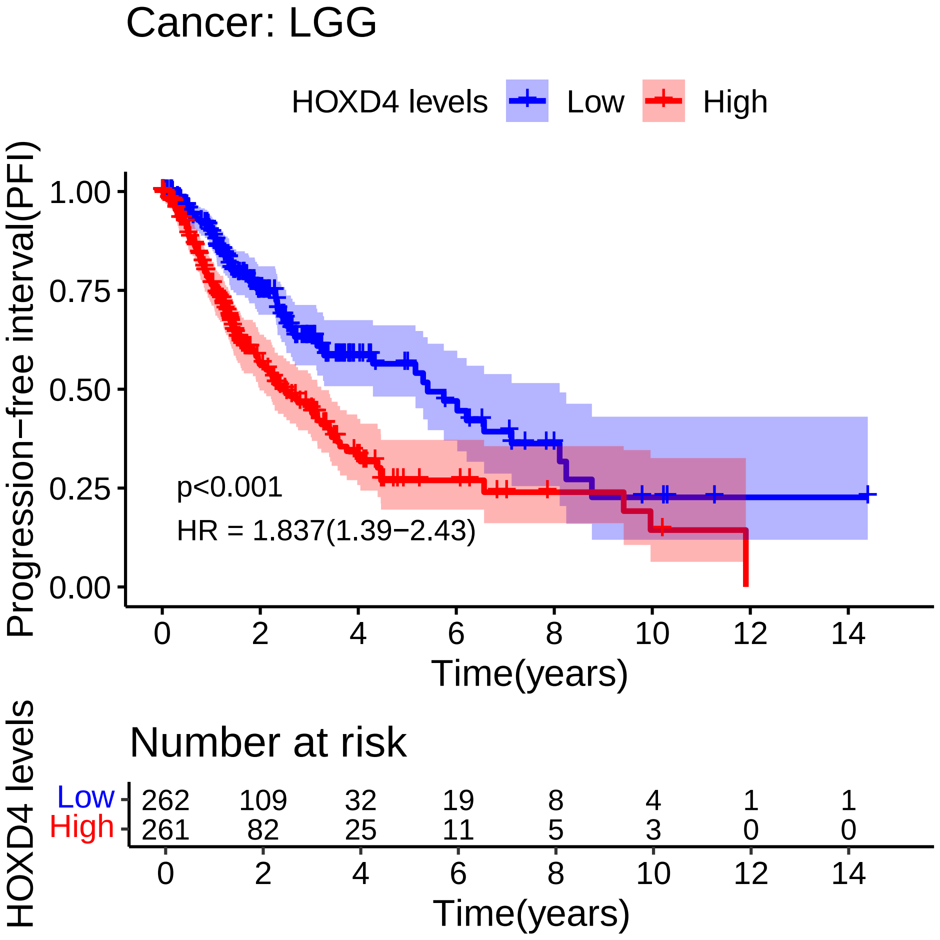
Cancer: LGG
HOXD4 levels
Low
High
Progression−free interval(PFI)
1.00
0.75
0.50
0.25
0.00
p<0.001
HR = 1.837(1.39−2.43)
0
2
4
6
8
10
12
14
Time(years)
Number at risk
HOXD4 levels
Low 262 109 32 19 8 4 1 1
High 261 82 25 11 5 3 0 0
0
2
4
6
8
10
12
14
Time(years)

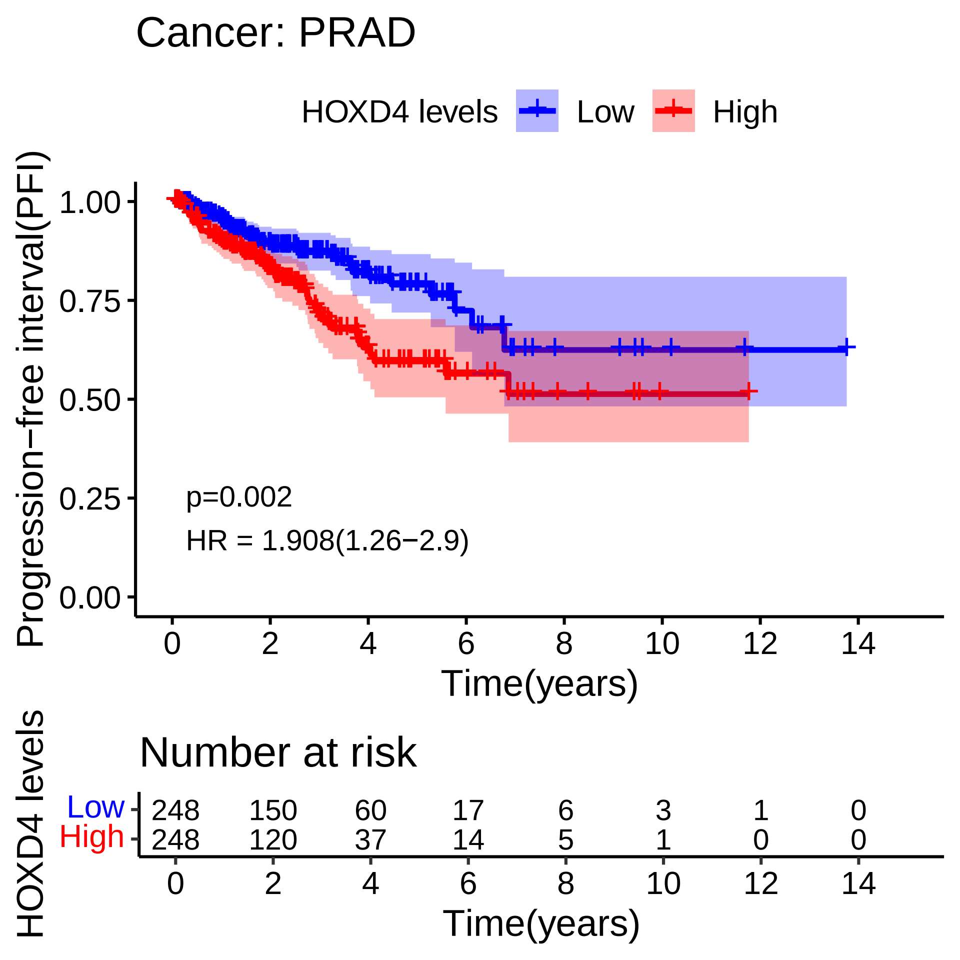

Cancer: PRAD
HOXD4 levels
Low
High
Progression−free interval(PFI)
1.00
0.75
0.50
0.25
0.00
p=0.002
HR = 1.908(1.26−2.9)
0
2
4
6
8
10
12
14
Time(years)
Number at risk
HOXD4 levels
Low 248 150 60 17 6 3 1 0
High 248 120 37 14 5 1 0 0
Time(years)

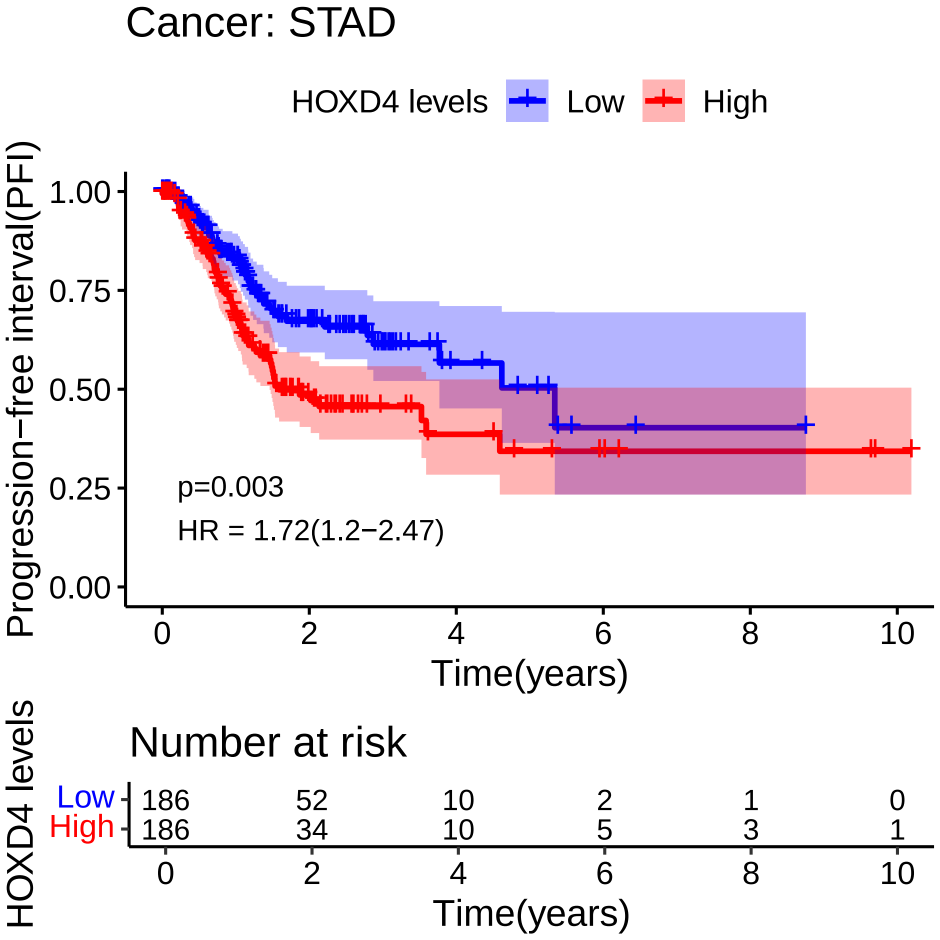
Cancer: STAD
HOXD4 levels
Low
High
Progression−free interval(PFI)
1.00
0.75
0.50
0.25
0.00
p=0.003
HR = 1.72(1.2−2.47)
0
2
4
6
8
10
Time(years)
Number at risk
HOXD4 levels
Low
High
186 52 10 2 1 0
186 34 10 5 3 1
0
2
4
6
8
10
Time(years)

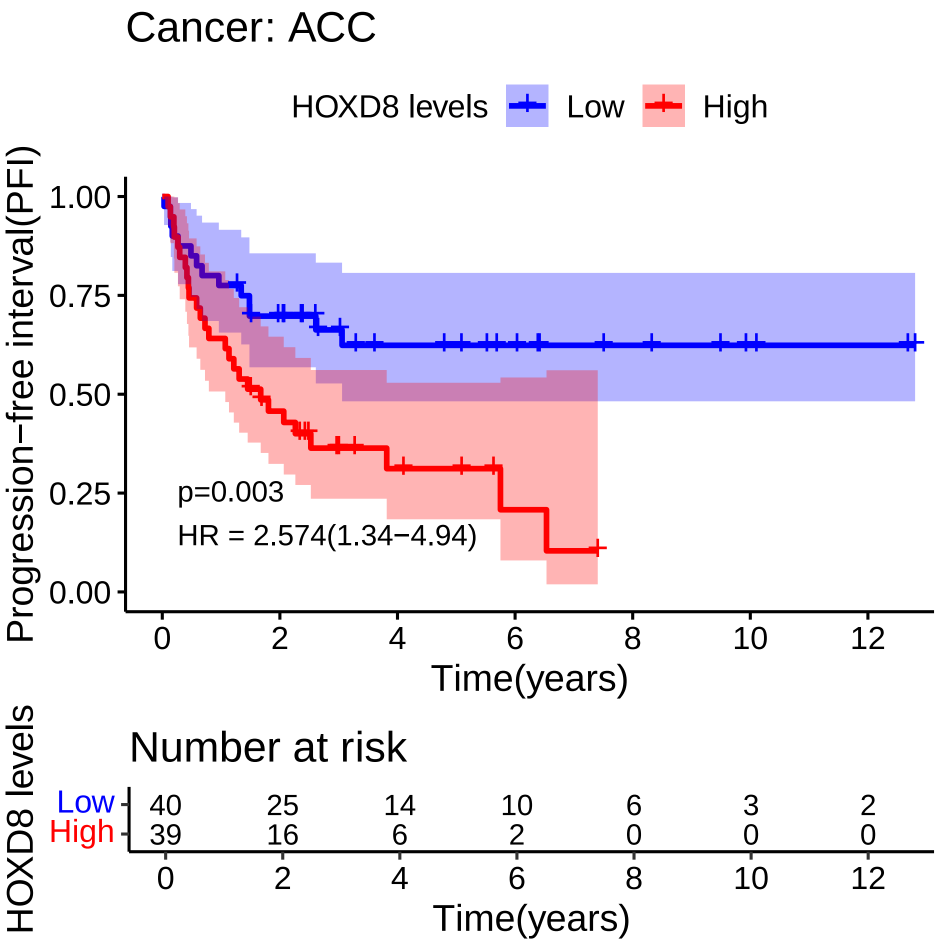

Cancer: ACC
HOXD8 levels
Low
High
Progression−free interval(PFI)
1.00
0.75
0.50
0.25
0.00
p=0.003
HR = 2.574(1.34−4.94)
0
2
4
6
8
10
12
Time(years)
Number at risk
HOXD8 levels
Low 40 25 14 10 6 3 2
High 39 16 6 2 0 0 0
Time(years)

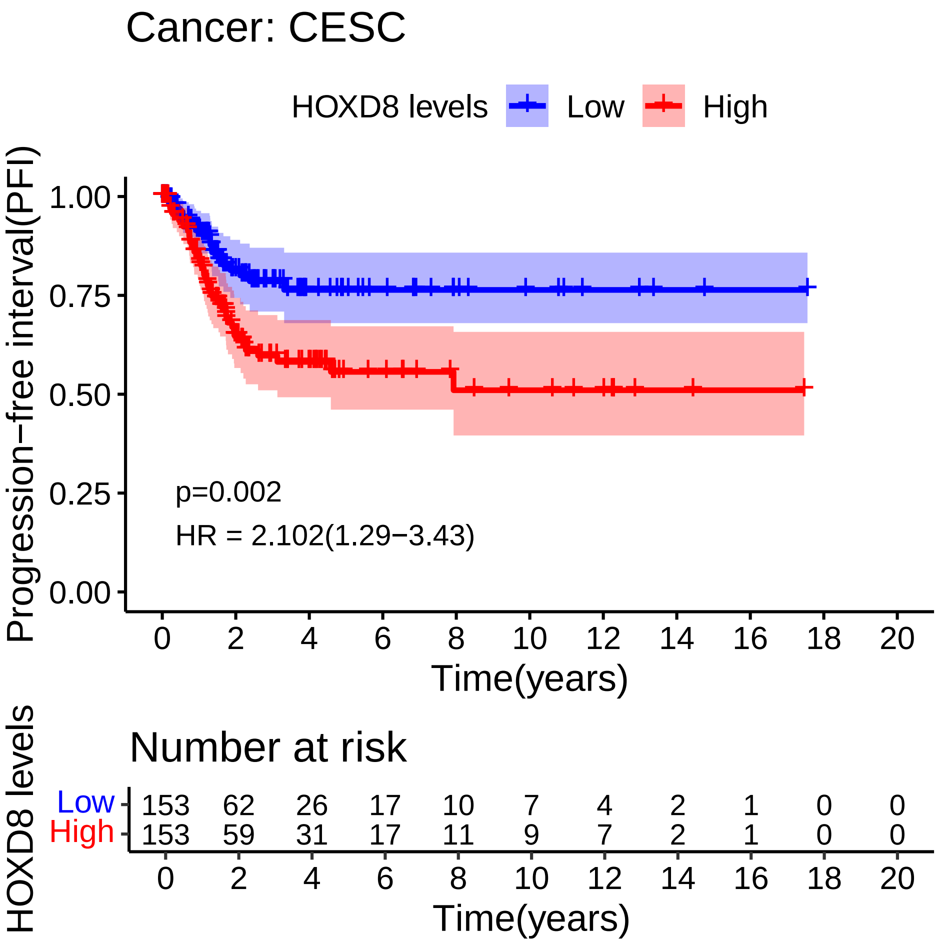

Cancer: CESC
HOXD8 levels
Low
High
Progression−free interval(PFI)
1.00
0.75
0.50
0.25
0.00
p=0.002
HR = 2.102(1.29−3.43)
0 2 4 6 8 10 12 14 16 18 20
Time(years)
Number at risk
HOXD8 levels
Low 153 62 26 17 10 7 4 2 1 0 0
High 153 59 31 17 11 9 7 2 1 0 0
0 2 4 6 8 10 12 14 16 18 20
Time(years)

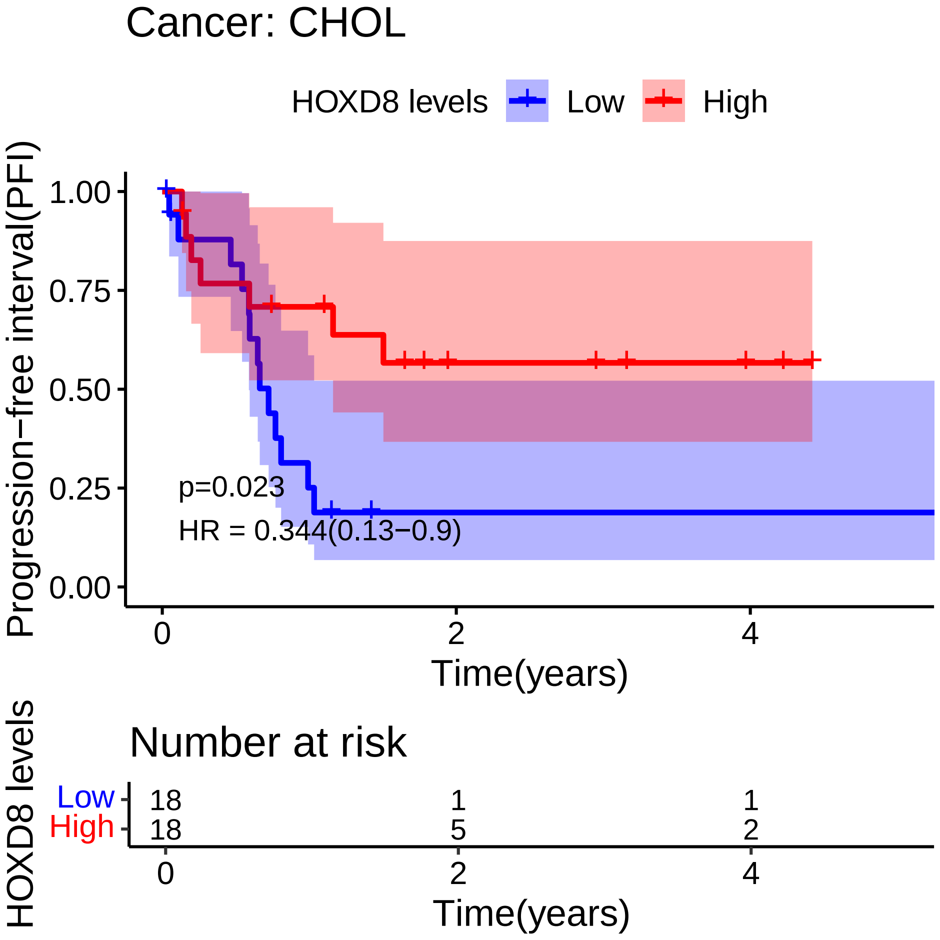

Cancer: CHOL
HOXD8 levels
Low
High
Progression−free interval(PFI)
1.00
0.75
0.50
0.25
0.00
p=0.023
HR = 0.344(0.13−0.9)
0
2
4
Time(years)
Number at risk
HOXD8 levels
Low 18 1 1
High 18 5 2
0
2
4
Time(years)

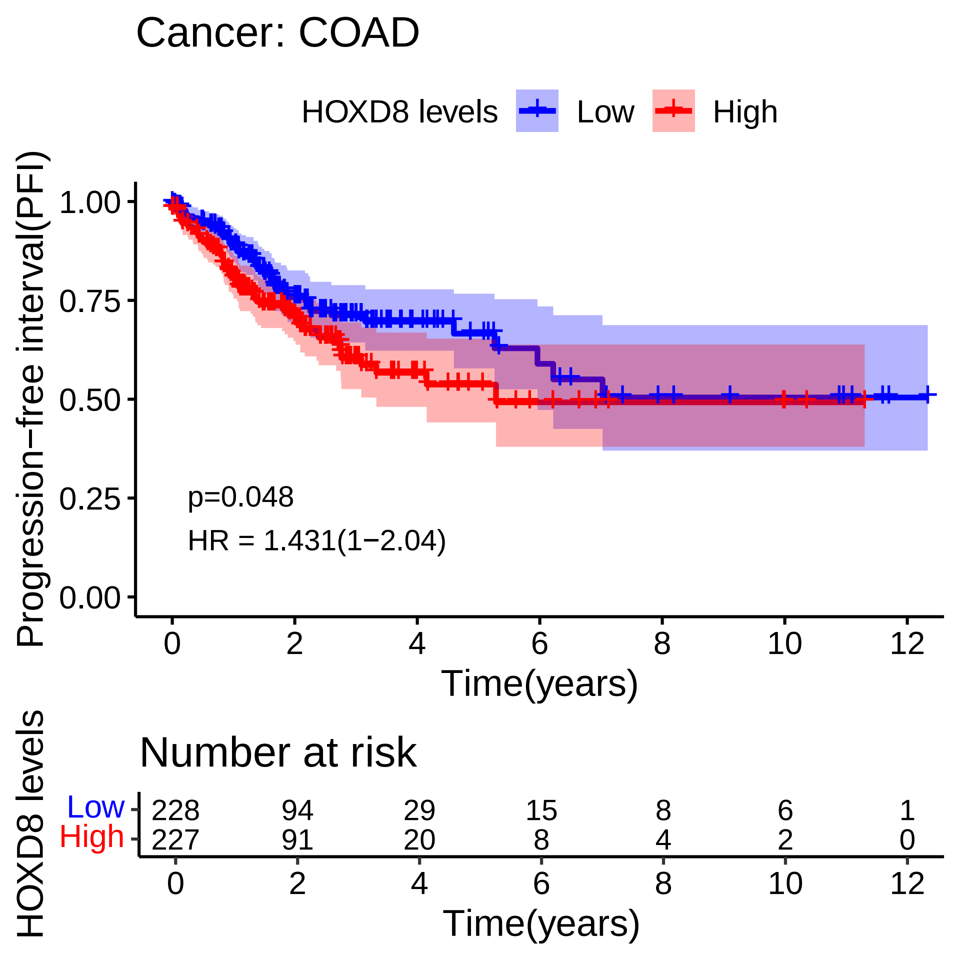

Cancer: COAD
HOXD8 levels
Low
High
Progression−free interval(PFI)
1.00
0.75
0.50
0.25
0.00
p=0.048
HR = 1.431(1−2.04)
0
2
4
6
8
10
12
Time(years)
Number at risk
HOXD8 levels
Low 228 94 29 15 8 6 1
High 227 91 20 8 4 2 0
Time(years)

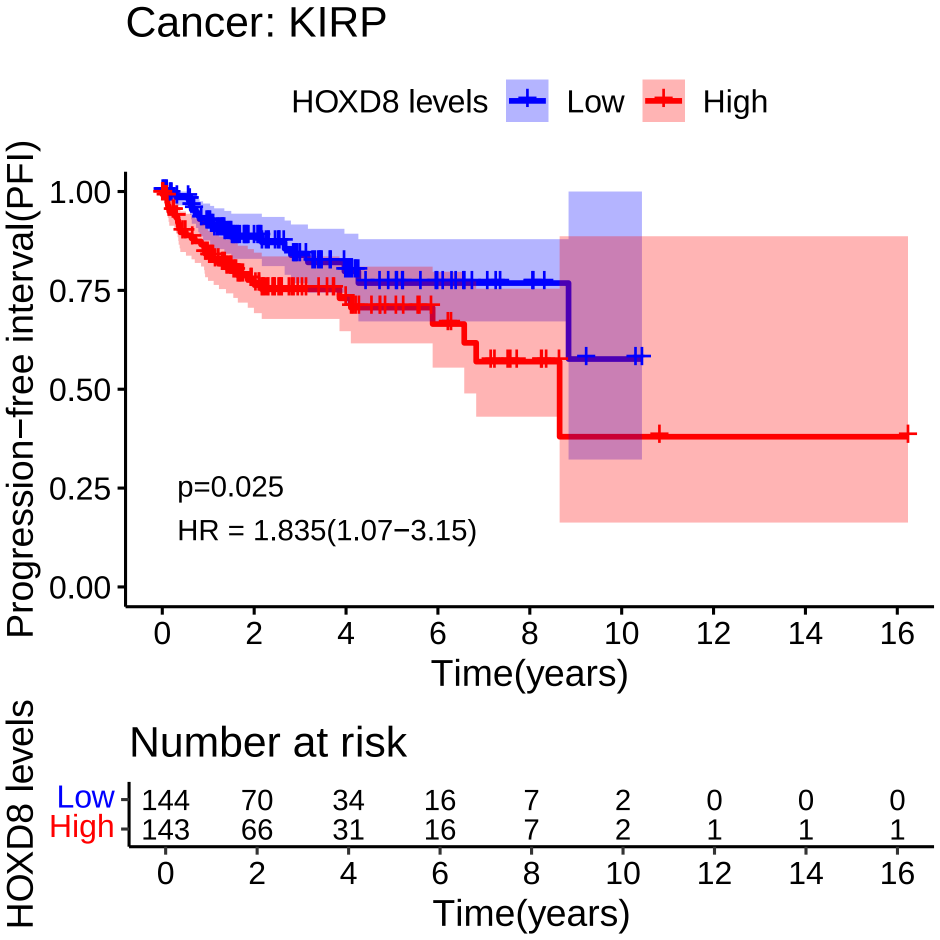

Cancer: KIRP
HOXD8 levels
Low
High
Progression−free interval(PFI)
1.00
0.75
0.50
0.25
0.00
p=0.025
HR = 1.835(1.07−3.15)
0
2
4
6
8
10
12
14
16
Time(years)
Number at risk
HOXD8 levels
Low 144 70 34 16 7 2 0 0 0
High 143 66 31 16 7 2 1 1 1
Time(years)

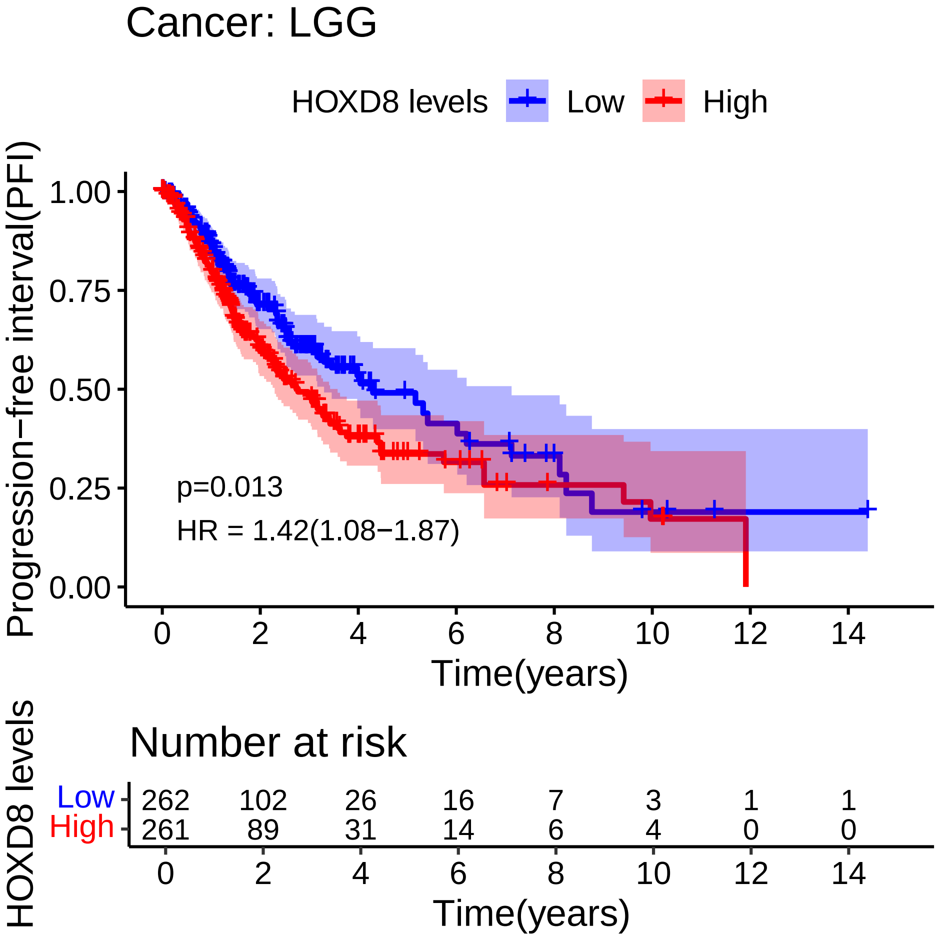

Cancer: LGG
HOXD8 levels
Low
High
Progression−free interval(PFI)
1.00
0.75
0.50
0.25
0.00
p=0.013
HR = 1.42(1.08−1.87)
0
2
4
6
8
10
12
14
Time(years)
Number at risk
HOXD8 levels
Low 262 102 26 16 7 3 1 1
High 261 89 31 14 6 4 0 0
0
2
4
6
8
10
12
14
Time(years)

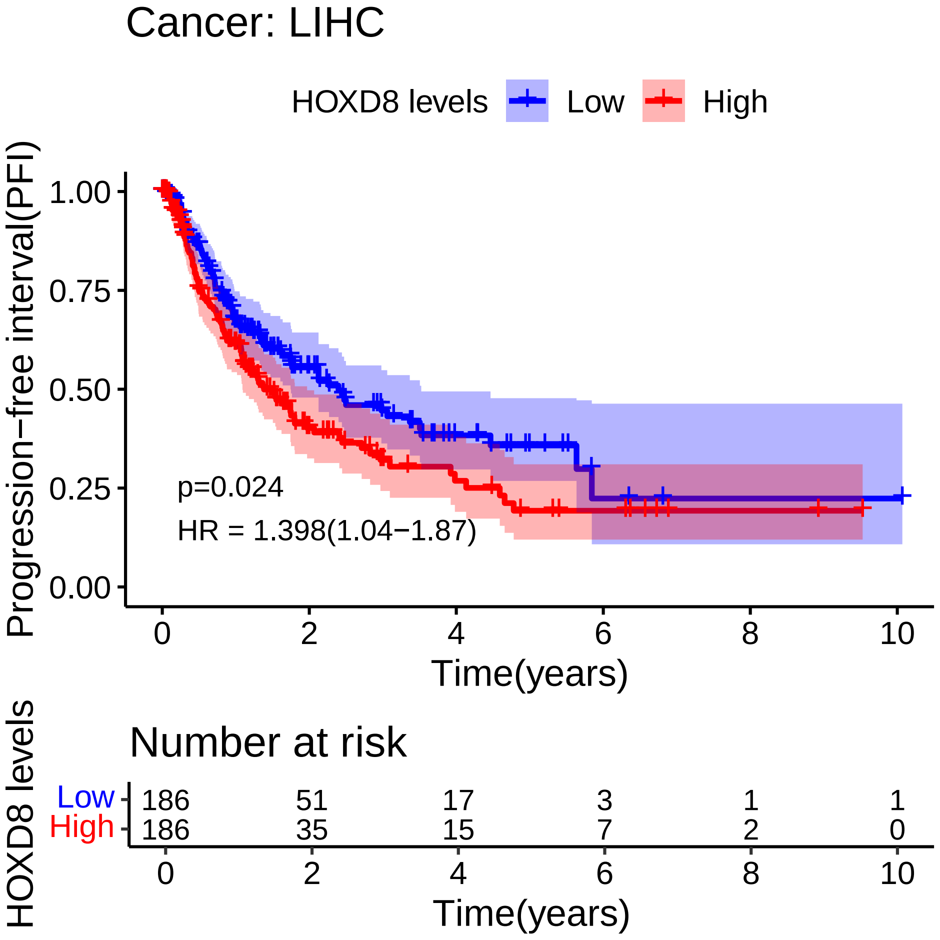
Cancer: LIHC
HOXD8 levels
Low
High
Progression−free interval(PFI)
1.00
0.75
0.50
0.25
0.00
p=0.024
HR = 1.398(1.04−1.87)
0
2
4
6
8
10
Time(years)
Number at risk
HOXD8 levels
Low 186 51 17 3 1 1
High 186 35 15 7 2 0
0
2
4
6
8
10
Time(years)

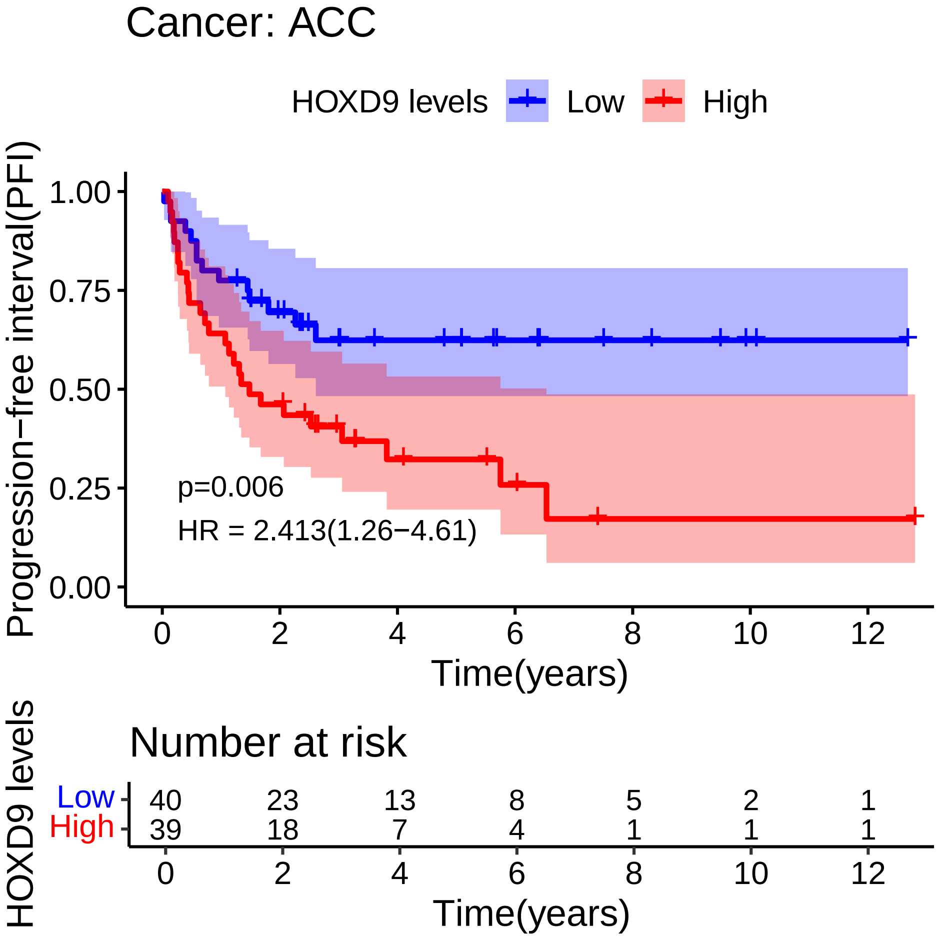
Cancer: ACC
HOXD9 levels
Low
High
Progression−free interval(PFI)
1.00
0.75
0.50
0.25
0.00
p=0.006
HR = 2.413(1.26−4.61)
0
2
4
6
8
10
12
Time(years)
Number at risk
HOXD9 levels
Low 40 23 13 8 5 2 1
High 39 18 7 4 1 1 1
Time(years)

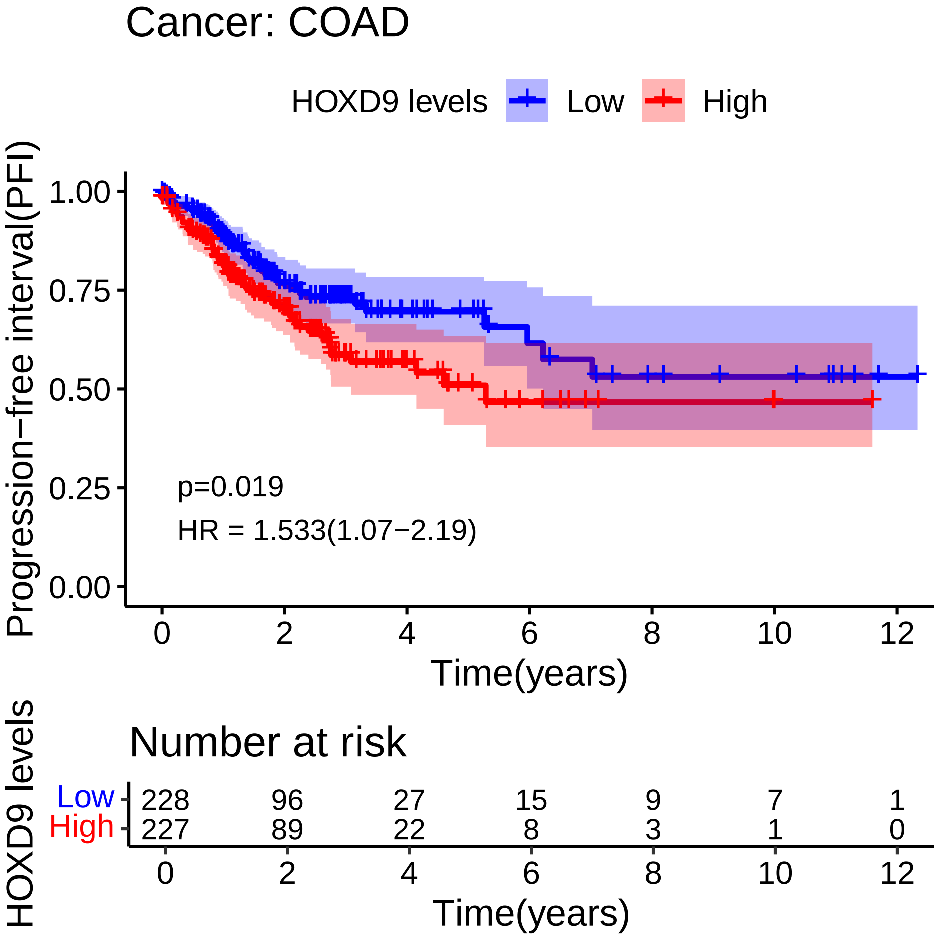

Cancer: COAD
HOXD9 levels
Low
High
Progression−free interval(PFI)
1.00
0.75
0.50
0.25
0.00
p=0.019
HR = 1.533(1.07−2.19)
0
2
4
6
8
10
12
Time(years)
Number at risk
HOXD9 levels
Low 228 96 27 15 9 7 1
High 227 89 22 8 3 1 0
Time(years)

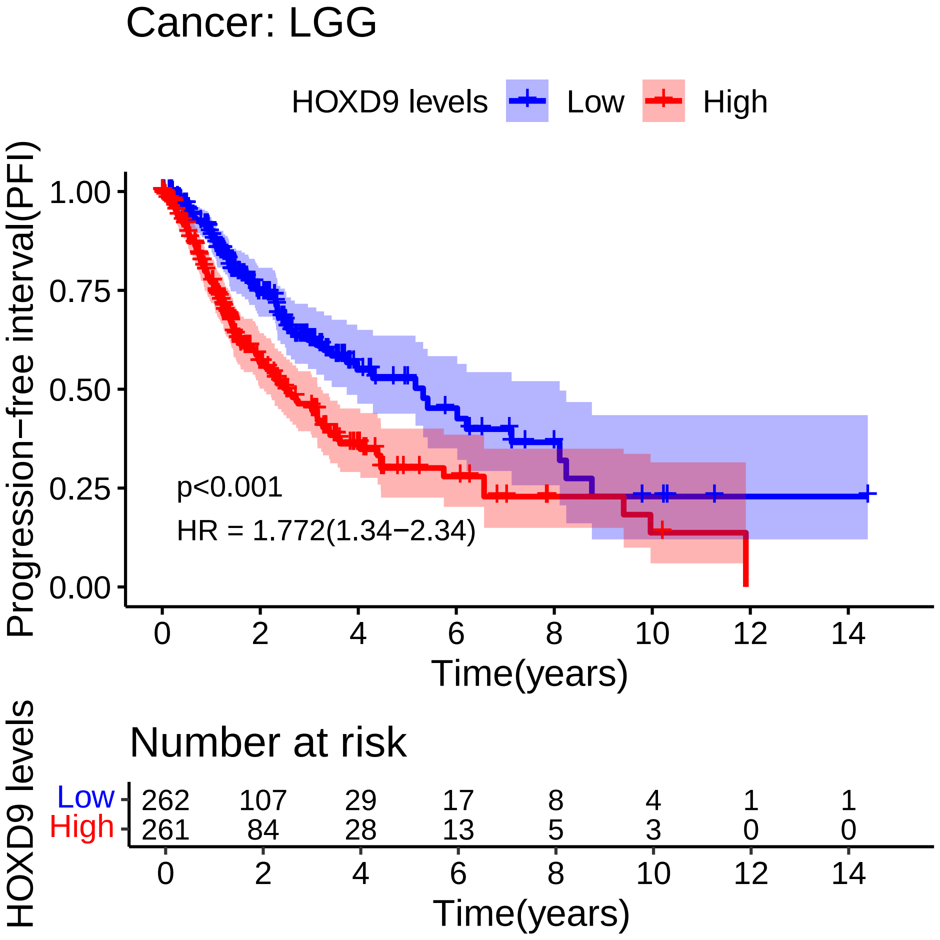
Cancer: LGG
HOXD9 levels
Low
High
Progression−free interval(PFI)
1.00
0.75
0.50
0.25
0.00
p<0.001
HR = 1.772(1.34−2.34)
0 2 4 6 8 10 12 14
Time(years)
Number at risk
HOXD9 levels
Low 262 107 29 17 8 4 1 1
High 261 84 28 13 5 3 0 0
0 2 4 6 8 10 12 14
Time(years)

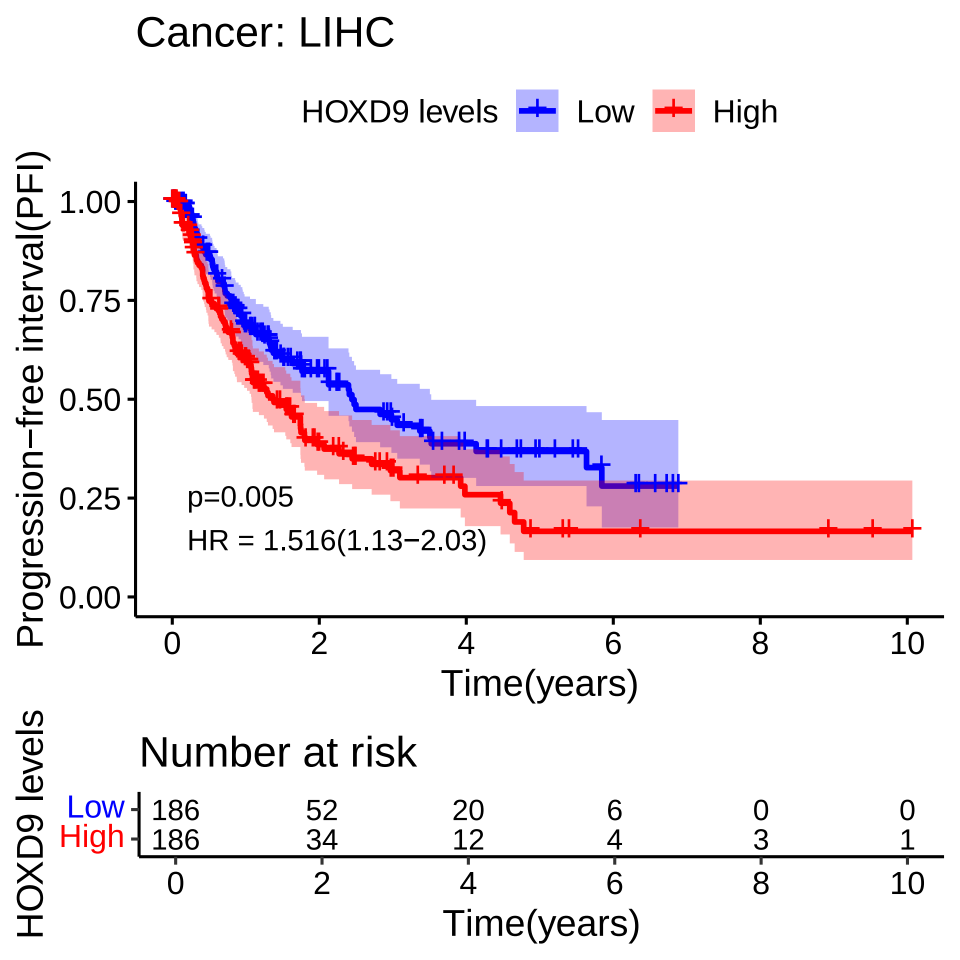

Cancer: LIHC
HOXD9 levels
Low
High
Progression−free interval(PFI)
1.00
0.75
0.50
0.25
0.00
p=0.005
HR = 1.516(1.13−2.03)
0
2
4
6
8
10
Time(years)
Number at risk
HOXD9 levels
Low 186 52 20 6 0 0
High 186 34 12 4 3 1
0
2
4
6
8
10
Time(years)

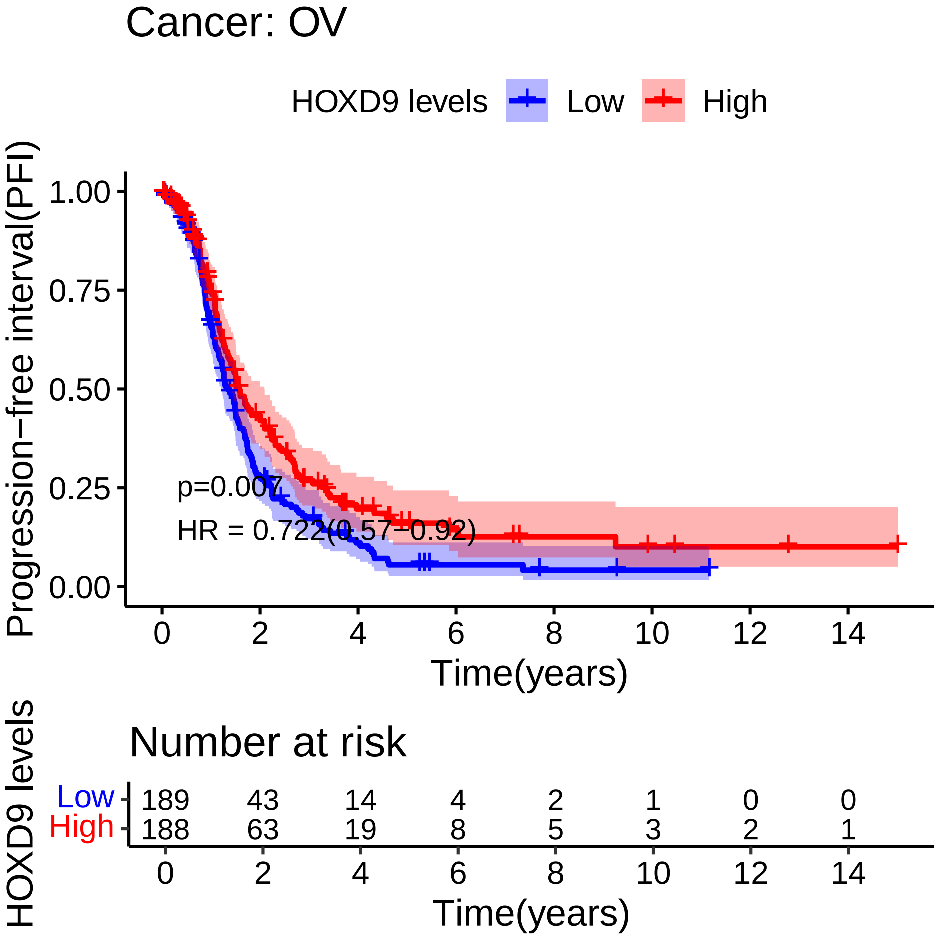

Cancer: OV
HOXD9 levels
Low
High
Progression−free interval(PFI)
1.00
0.75
0.50
0.25
0.00
p=0.007
HR = 0.722(0.57−0.92)
0
2
4
6
8
10
12
14
Time(years)
Number at risk
HOXD9 levels
Low
High
189 43 14 4 2 1 0 0
188 63 19 8 5 3 2 1
0
2
4
6
8
10
12
14
Time(years)

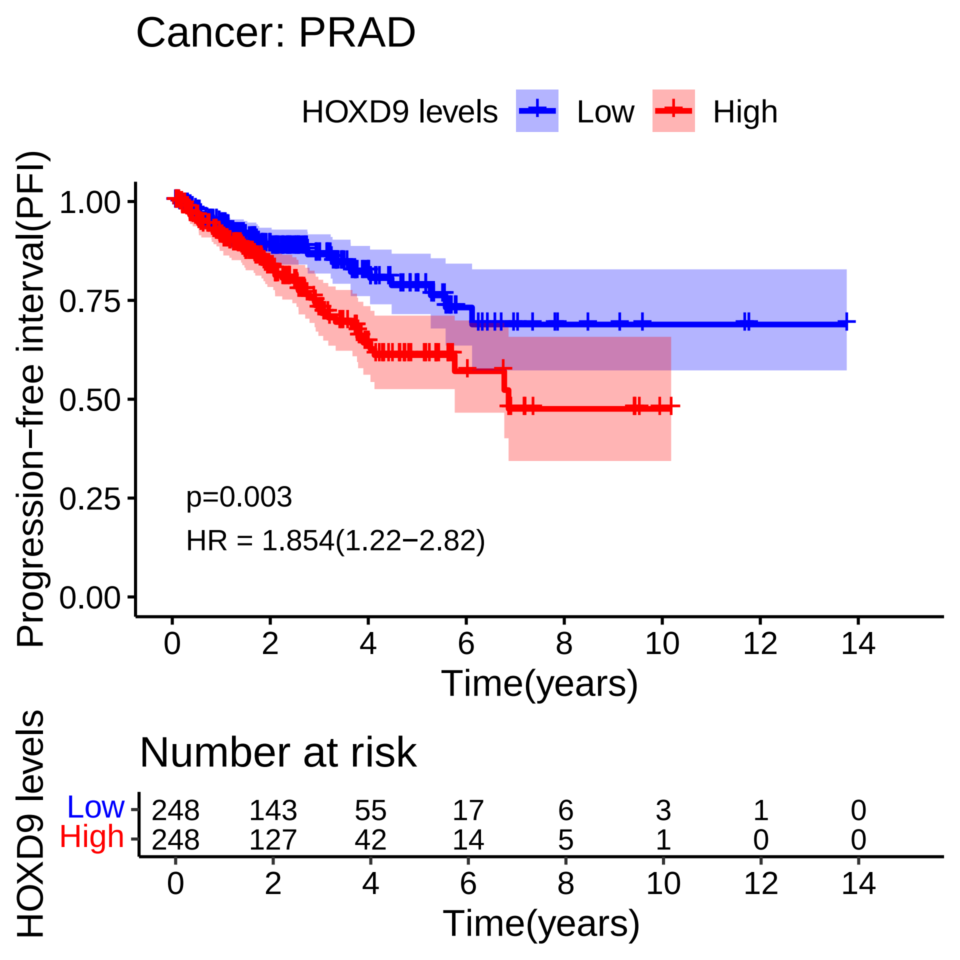

Cancer: PRAD
HOXD9 levels
Low
High
Progression−free interval(PFI)
1.00
0.75
0.50
0.25
0.00
p=0.003
HR = 1.854(1.22−2.82)
0
2
4
6
8
10
12
14
Time(years)
Number at risk
HOXD9 levels
Low 248 143 55 17 6 3 1 0
High 248 127 42 14 5 1 0 0
0
2
4
6
8
10
12
14
Time(years)

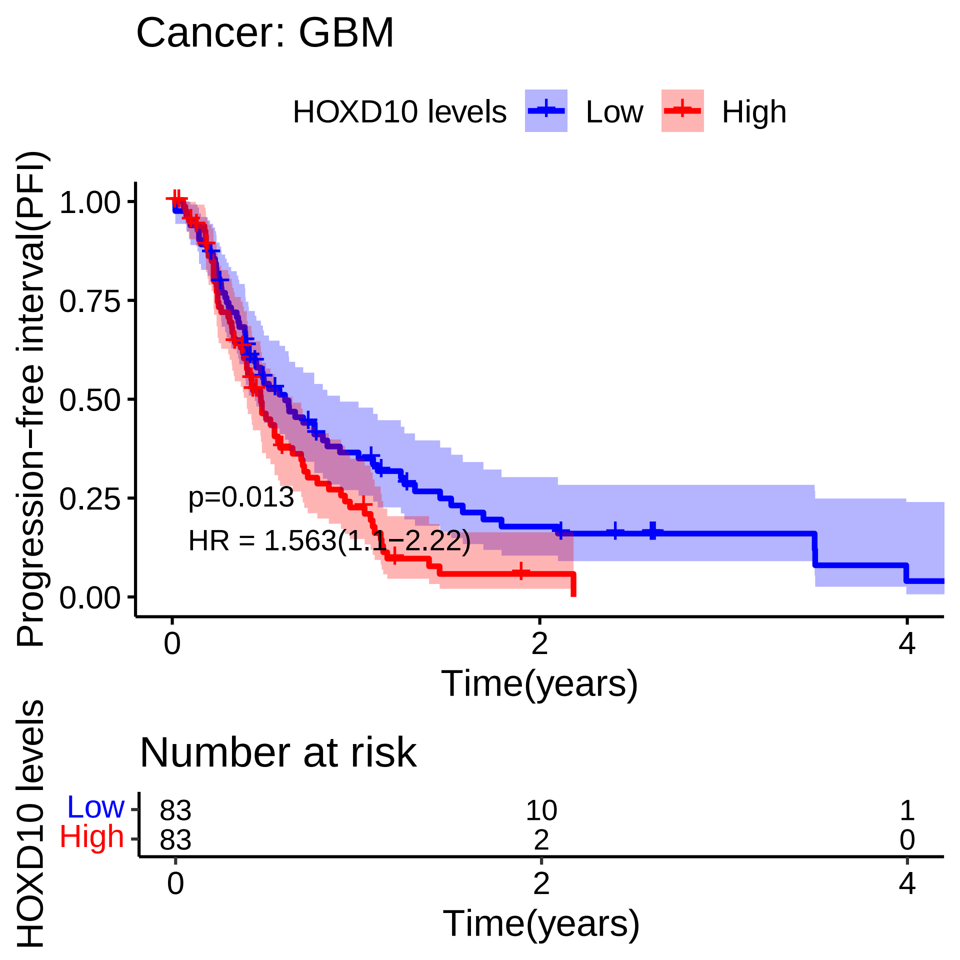
Cancer: GBM
HOXD10 levels
Low
High
Progression−free interval(PFI)
1.00
0.75
0.50
0.25
0.00
p=0.013
HR = 1.563(1.1−2.22)
0
2
4
Time(years)
Number at risk
HOXD10 levels
Low
High
83
10
1
83
2
0
0
2
4
Time(years)

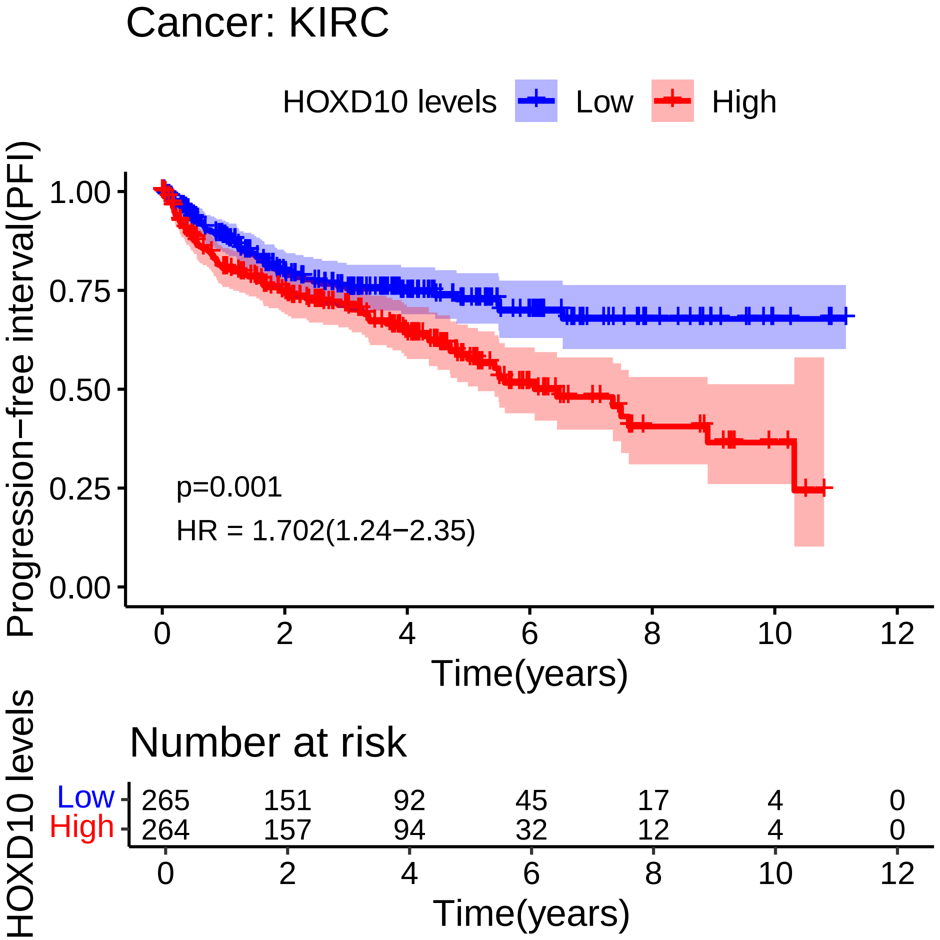

Cancer: KIRC
HOXD10 levels
Low
High
Progression−free interval(PFI)
1.00
0.75
0.50
0.25
0.00
p=0.001
HR = 1.702(1.24−2.35)
0
2
4
6
8
10
12
Time(years)
Number at risk
HOXD10 levels
Low 265 151 92 45 17 4 0
High 264 157 94 32 12 4 0
0
2
4
6
8
10
12
Time(years)

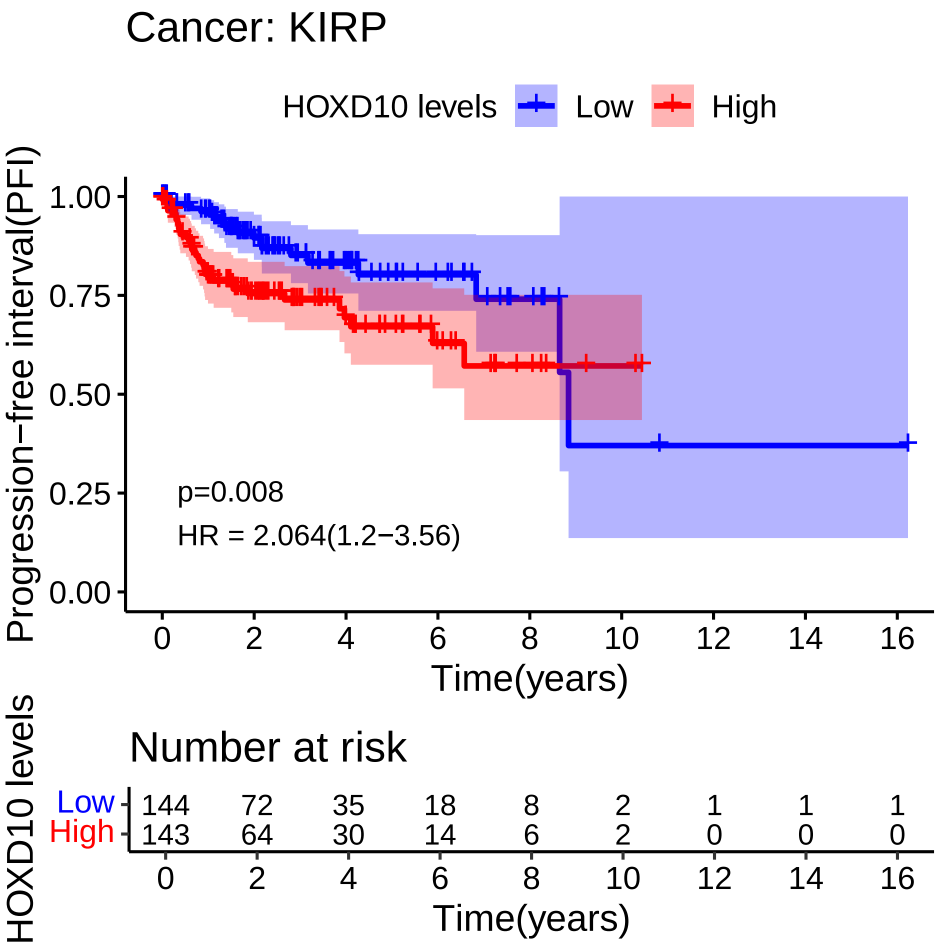

Cancer: KIRP
HOXD10 levels
Low
High
Progression−free interval(PFI)
1.00
0.75
0.50
0.25
0.00
p=0.008
HR = 2.064(1.2−3.56)
0
2
4
6
8
10
12
14
16
Time(years)
Number at risk
HOXD10 levels
Low 144 72 35 18 8 2 1 1 1
High 143 64 30 14 6 2 0 0 0
Time(years)

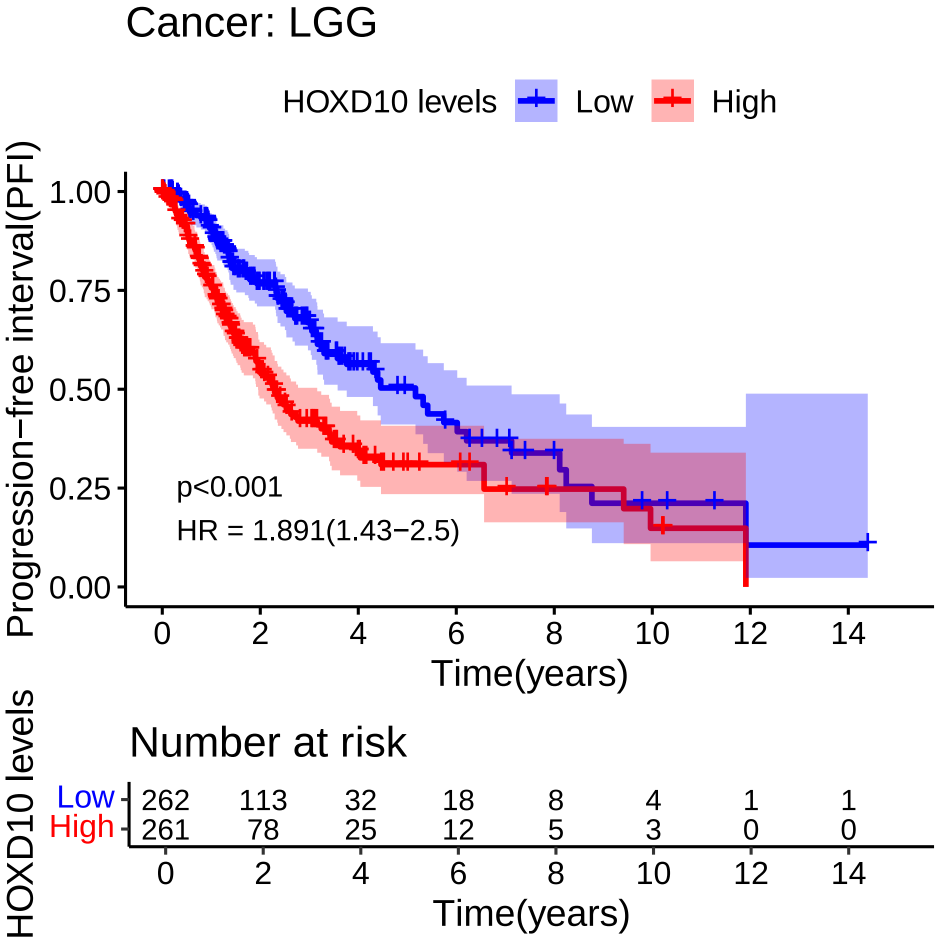

Cancer: LGG
HOXD10 levels
Low
High
Progression−free interval(PFI)
1.00
0.75
0.50
0.25
0.00
p<0.001
HR = 1.891(1.43−2.5)
0
2
4
6
8
10
12
14
Time(years)
Number at risk
HOXD10 levels
Low 262 113 32 18 8 4 1 1
High 261 78 25 12 5 3 0 0
Time(years)

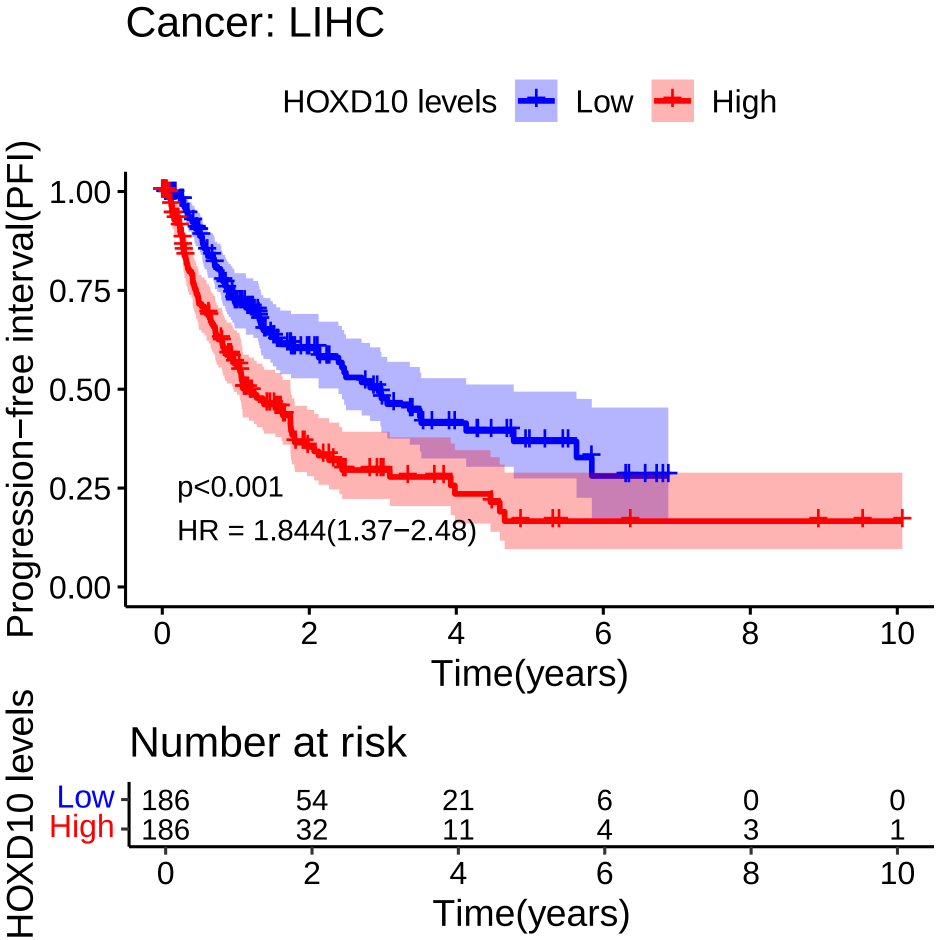
Cancer: LIHC
HOXD10 levels
Low
High
Progression−free interval(PFI)
1.00
0.75
0.50
0.25
0.00
p<0.001
HR = 1.844(1.37−2.48)
0
2
4
6
8
10
Time(years)
Number at risk
HOXD10 levels
Low
High
186 54 21 6 0 0
186 32 11 4 3 1
0
2
4
6
8
10
Time(years)

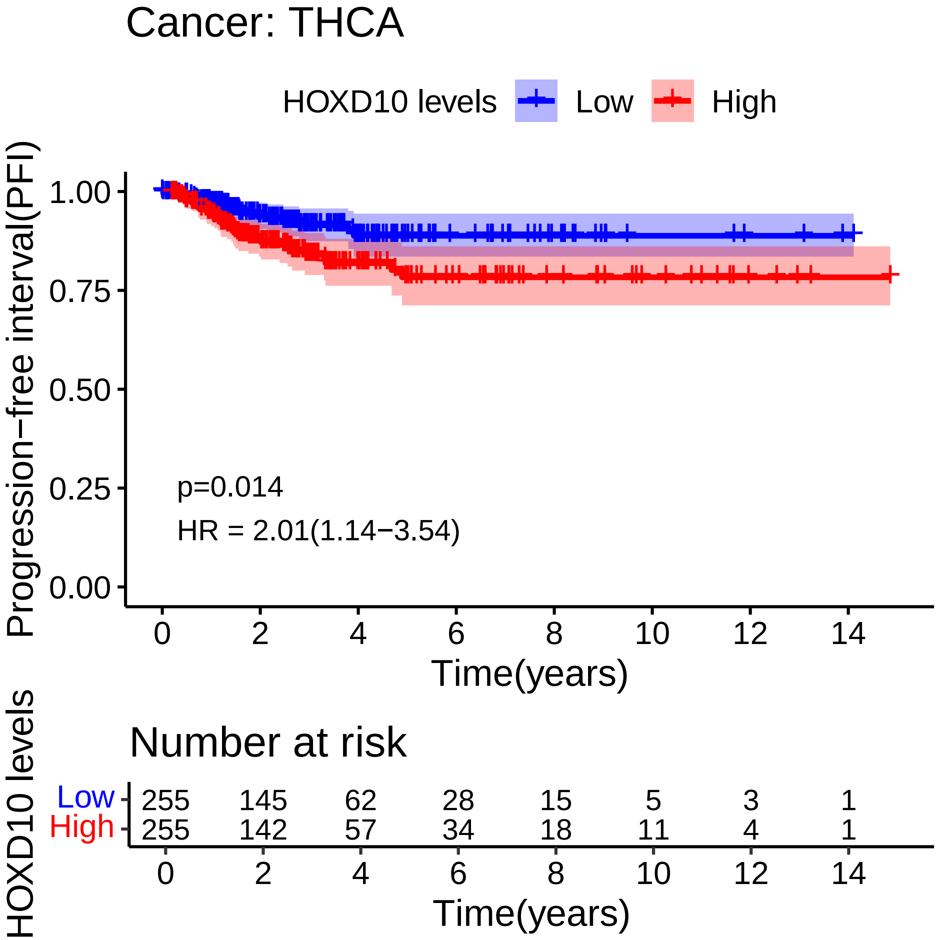

Cancer: THCA
HOXD10 levels
Low
High
Progression−free interval(PFI)
1.00
0.75
0.50
0.25
0.00
p=0.014
HR = 2.01(1.14−3.54)
0
2
4
6
8
10
12
14
Time(years)
Number at risk
HOXD10 levels
Low 255 145 62 28 15 5 3 1
High 255 142 57 34 18 11 4 1
Time(years)

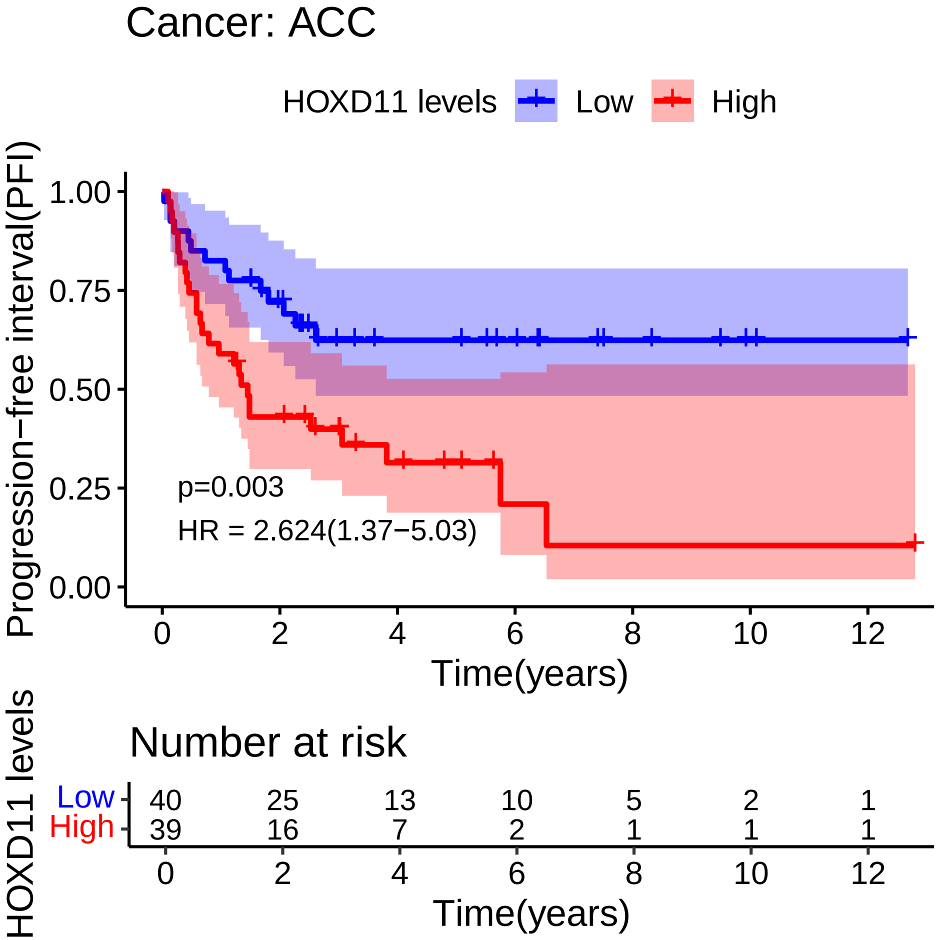

Cancer: ACC
HOXD11 levels
Low
High
Progression−free interval(PFI)
1.00
0.75
0.50
0.25
0.00
p=0.003
HR = 2.624(1.37−5.03)
0
2
4
6
8
10
12
Time(years)
Number at risk
HOXD11 levels
Low 40 25 13 10 5 2 1
High 39 16 7 2 1 1 1
Time(years)

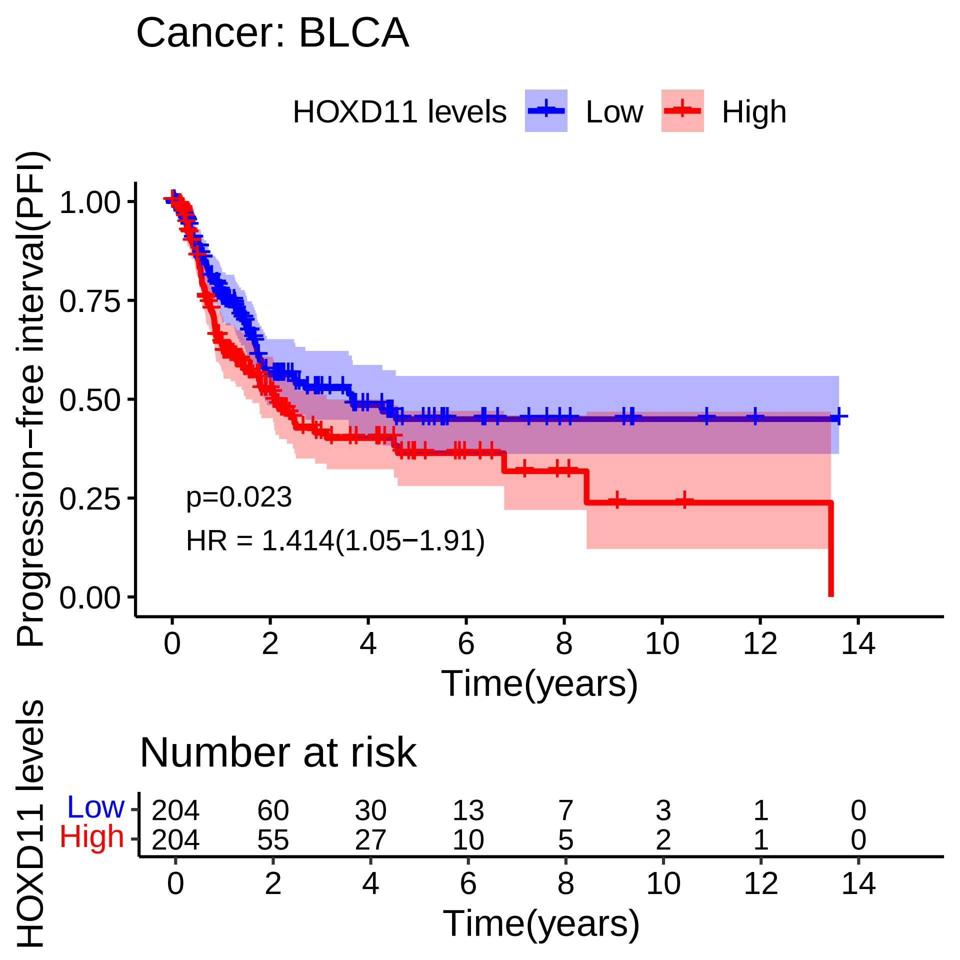
Cancer: BLCA
HOXD11 levels
Low
High
Progression−free interval(PFI)
1.00
0.75
0.50
0.25
0.00
p=0.023
HR = 1.414(1.05−1.91)
0
2
4
6
8
10
12
14
Time(years)
Number at risk
HOXD11 levels
Low 204 60 30 13 7 3 1 0
High 204 55 27 10 5 2 1 0
Time(years)

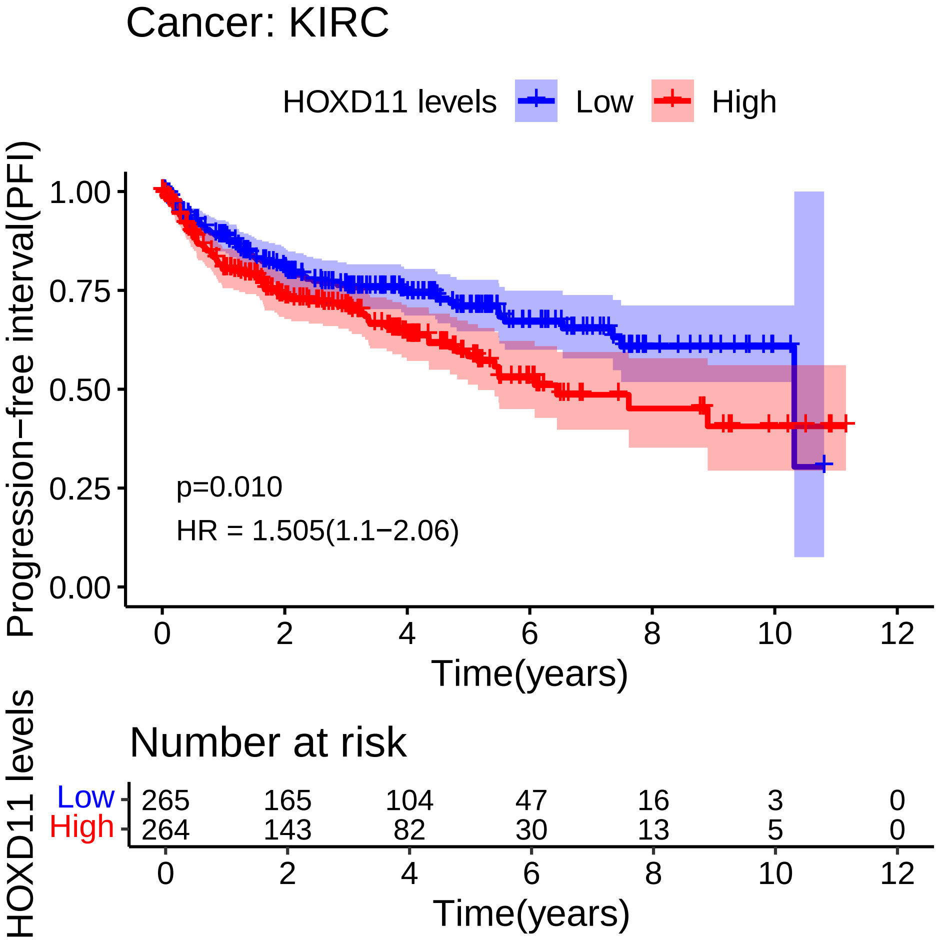

Cancer: KIRC
HOXD11 levels
Low
High
Progression−free interval(PFI)
1.00
0.75
0.50
0.25
0.00
p=0.010
HR = 1.505(1.1−2.06)
0
2
4
6
8
10
12
Time(years)
Number at risk
HOXD11 levels
Low 265 165 104 47 16 3 0
High 264 143 82 30 13 5 0
Time(years)

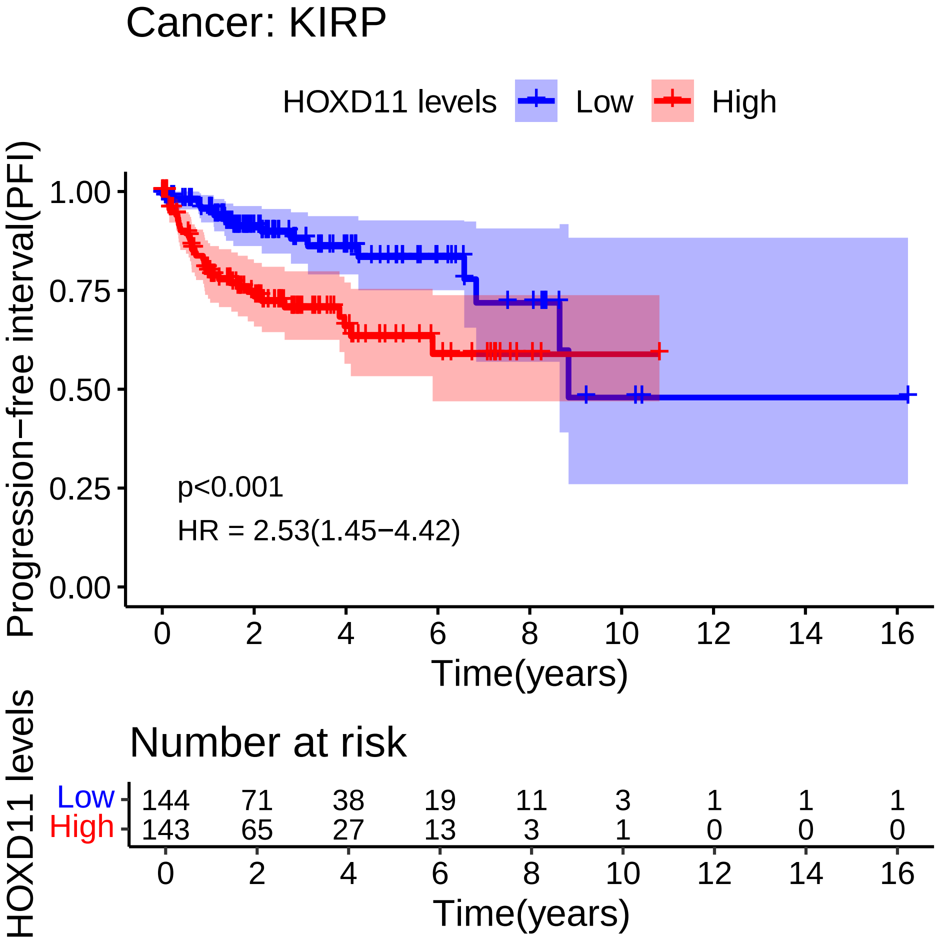
Cancer: KIRP
HOXD11 levels
Low
High
Progression−free interval(PFI)
1.00
0.75
0.50
0.25
0.00
p<0.001
HR = 2.53(1.45−4.42)
0
2
4
6
8
10
12
14
16
Time(years)
Number at risk
HOXD11 levels
Low 144 71 38 19 11 3 1 1 1
High 143 65 27 13 3 1 0 0 0
Time(years)

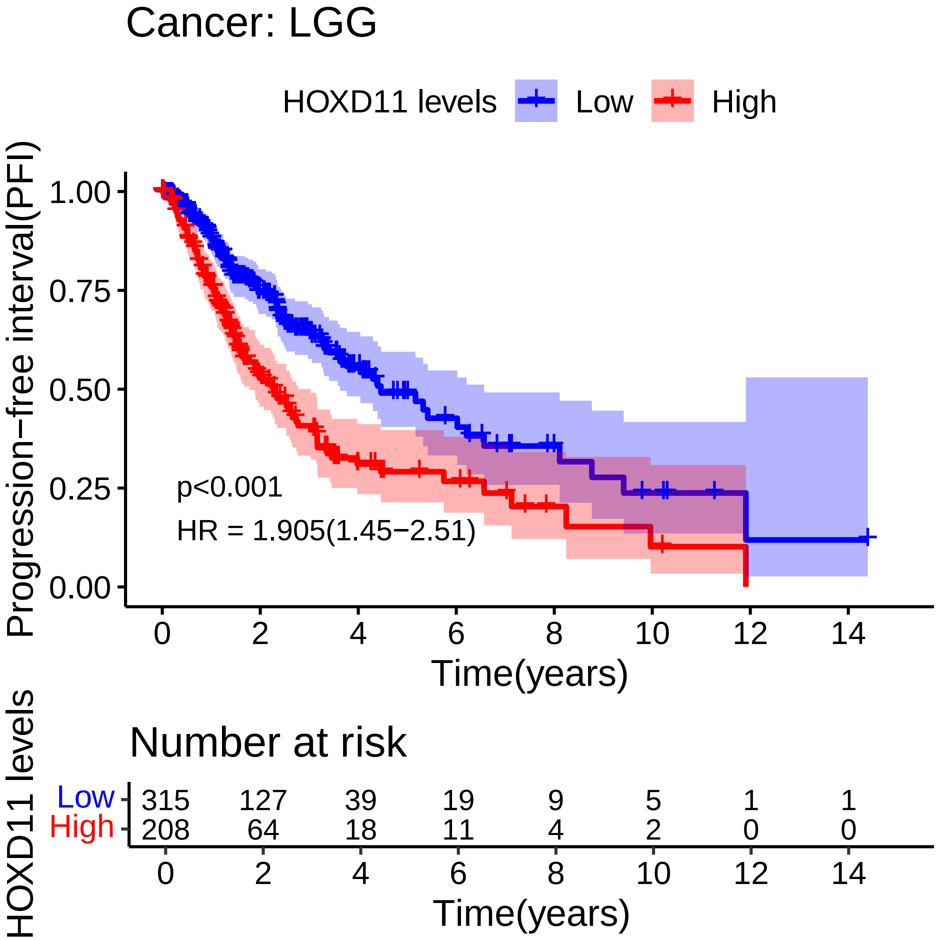

Cancer: LGG
HOXD11 levels
Low
High
Progression−free interval(PFI)
1.00
0.75
0.50
0.25
0.00
p<0.001
HR = 1.905(1.45−2.51)
0
2
4
6
8
10
12
14
Time(years)
Number at risk
HOXD11 levels
Low 315 127 39 19 9 5 1 1
High 208 64 18 11 4 2 0 0
Time(years)

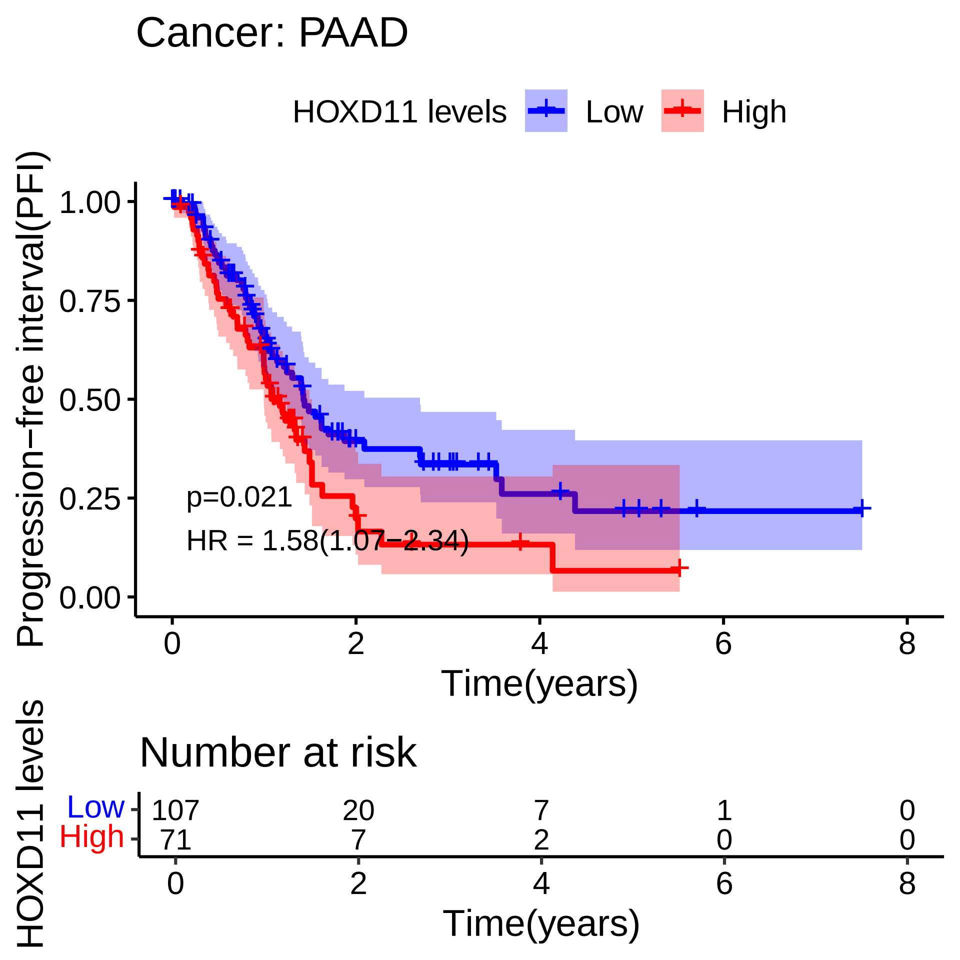
Cancer: PAAD
HOXD11 levels
Low
High
Progression−free interval(PFI)
1.00
0.75
0.50
0.25
0.00
p=0.021
HR = 1.58(1.07−2.34)
0
2
4
6
8
Time(years)
Number at risk
HOXD11 levels
Low 107 20 7 1 0
High 71 7 2 0 0
0
2
4
6
8
Time(years)

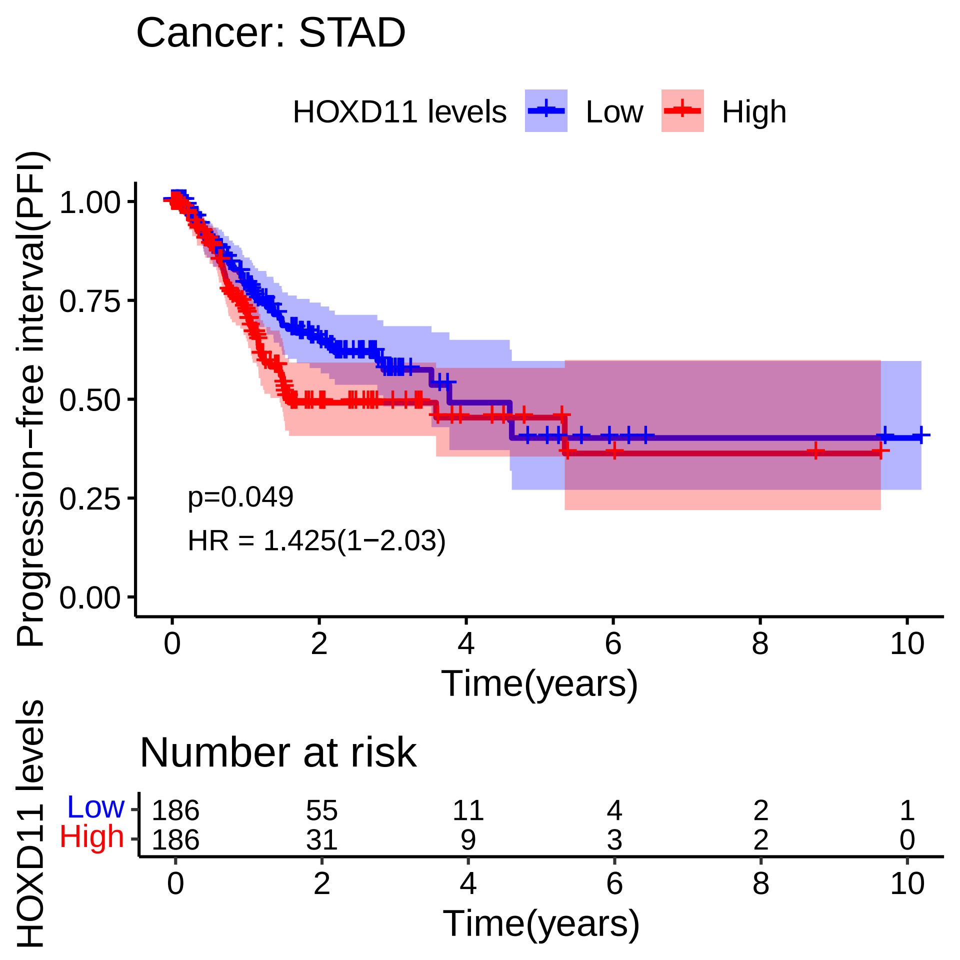
Cancer: STAD
HOXD11 levels
Low
High
Progression−free interval(PFI)
1.00
0.75
0.50
0.25
0.00
p=0.049
HR = 1.425(1−2.03)
0
2
4
6
8
10
Time(years)
Number at risk
HOXD11 levels
Low 186 55 11 4 2 1
High 186 31 9 3 2 0
Time(years)

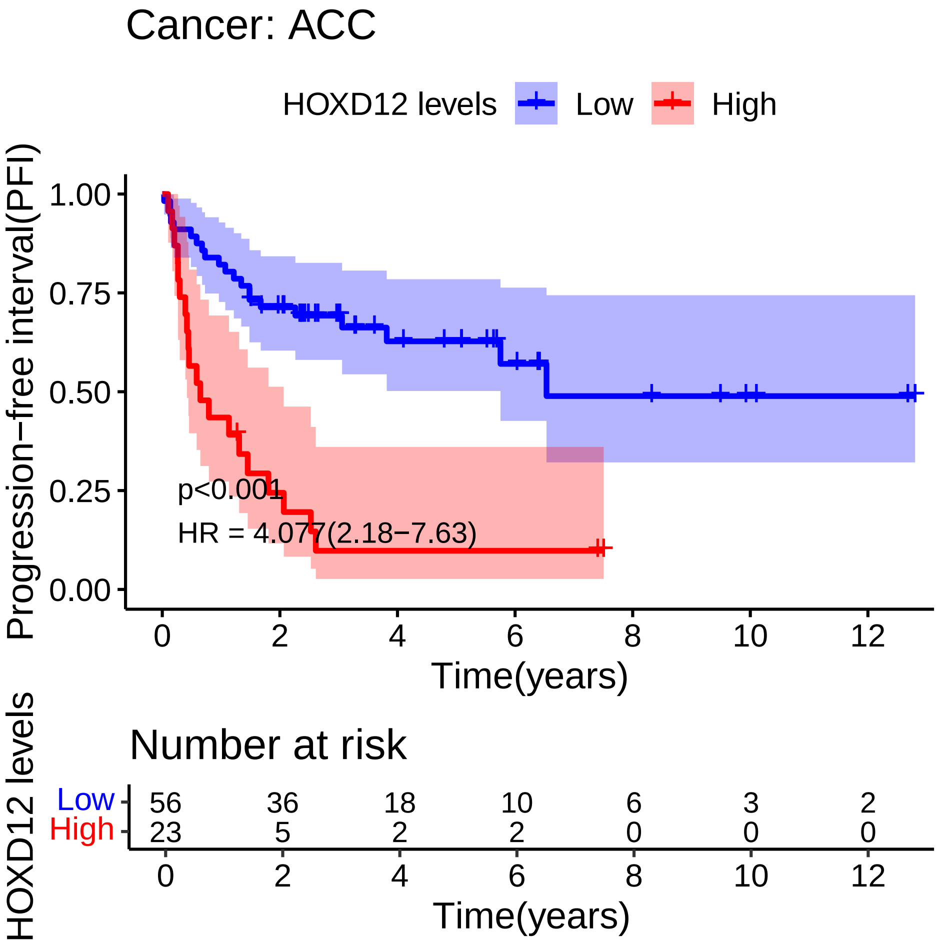

Cancer: ACC
HOXD12 levels
Low
High
Progression−free interval(PFI)
1.00
0.75
0.50
0.25
0.00
p<0.001
HR = 4.077(2.18−7.63)
0
2
4
6
8
10
12
Time(years)
Number at risk
HOXD12 levels
Low 56 36 18 10 6 3 2
High 23 5 2 2 0 0 0
0
2
4
6
8
10
12
Time(years)

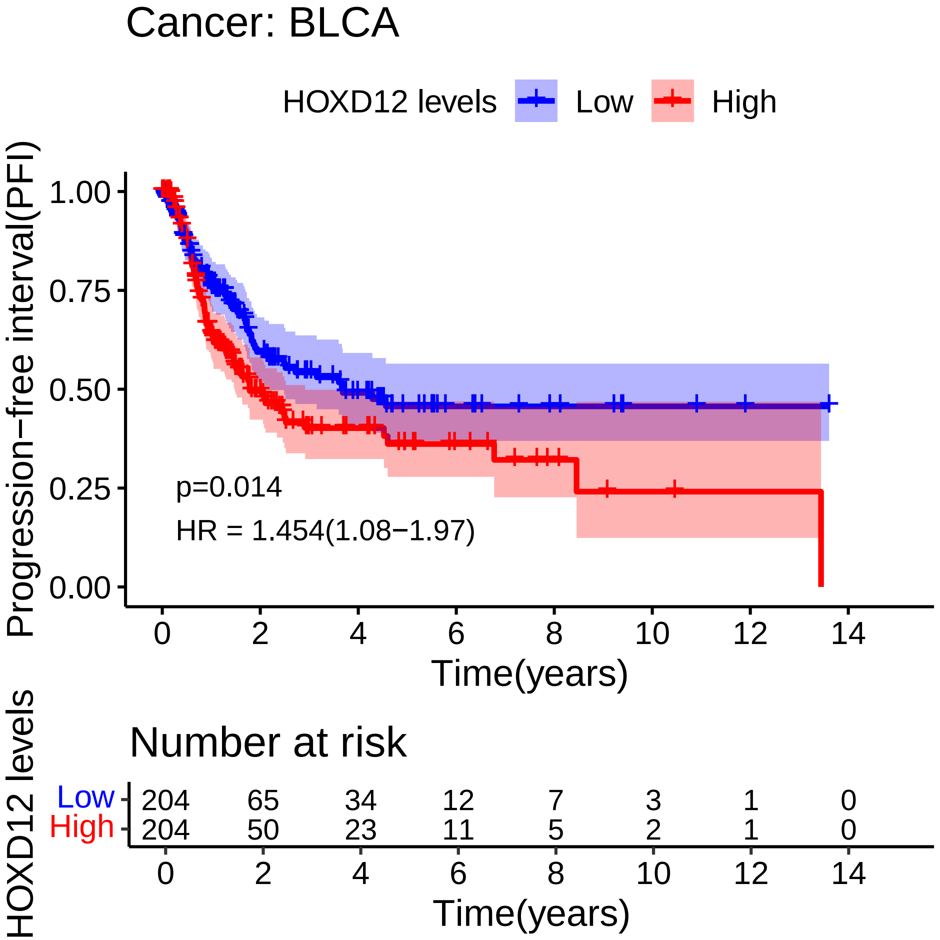

Cancer: BLCA
HOXD12 levels
Low
High
Progression−free interval(PFI)
1.00
0.75
0.50
0.25
0.00
p=0.014
HR = 1.454(1.08−1.97)
0
2
4
6
8
10
12
14
Time(years)
Number at risk
HOXD12 levels
Low 204 65 34 12 7 3 1 0
High 204 50 23 11 5 2 1 0
Time(years)

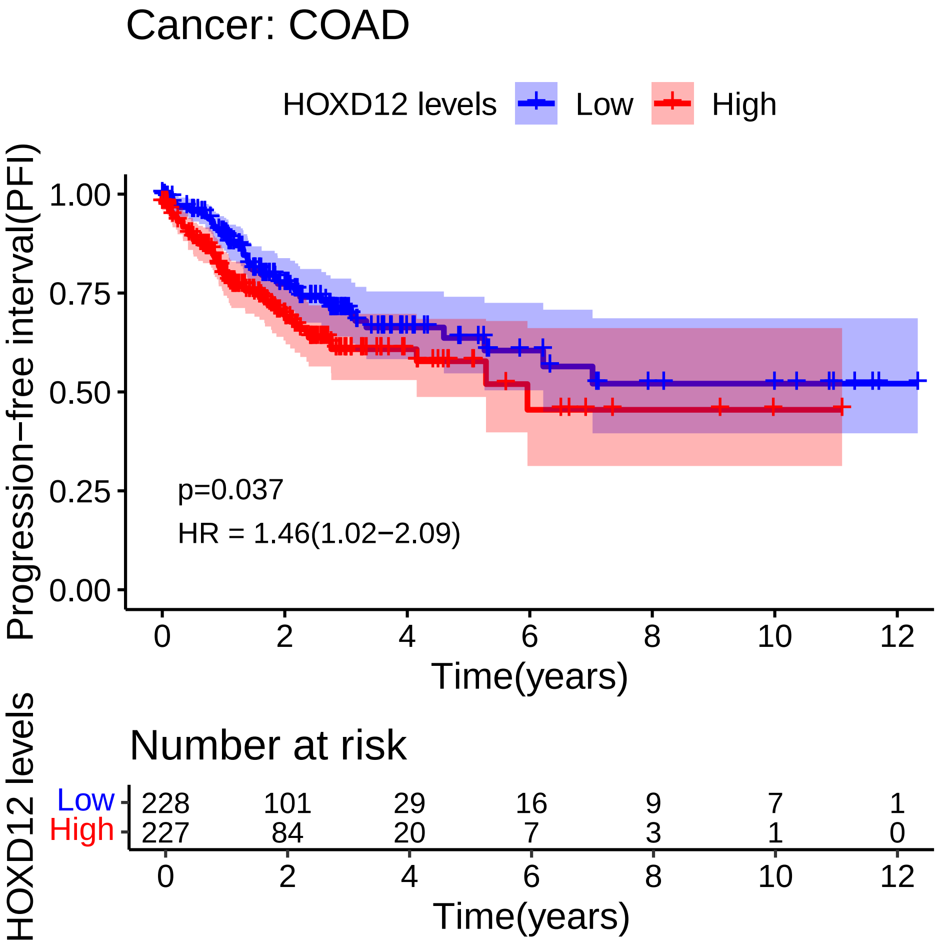
Cancer: COAD
HOXD12 levels
Low
High
Progression−free interval(PFI)
1.00
0.75
0.50
0.25
0.00
p=0.037
HR = 1.46(1.02−2.09)
0
2
4
6
8
10
12
Time(years)
Number at risk
HOXD12 levels
Low 228 101 29 16 9 7 1
High 227 84 20 7 3 1 0
Time(years)

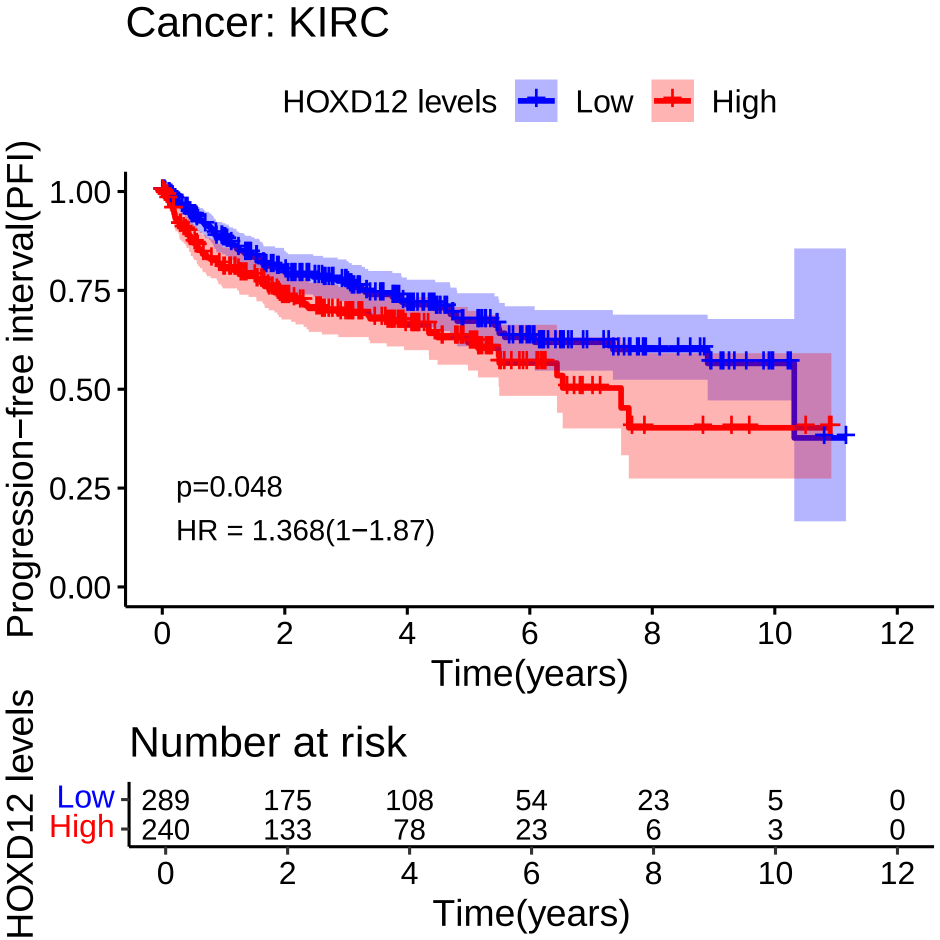
Cancer: KIRC
HOXD12 levels
Low
High
Progression−free interval(PFI)
1.00
0.75
0.50
0.25
0.00
p=0.048
HR = 1.368(1−1.87)
0
2
4
6
8
10
12
Time(years)
Number at risk
HOXD12 levels
Low
High
289 175 108 54 23 5 0
240 133 78 23 6 3 0
Time(years)

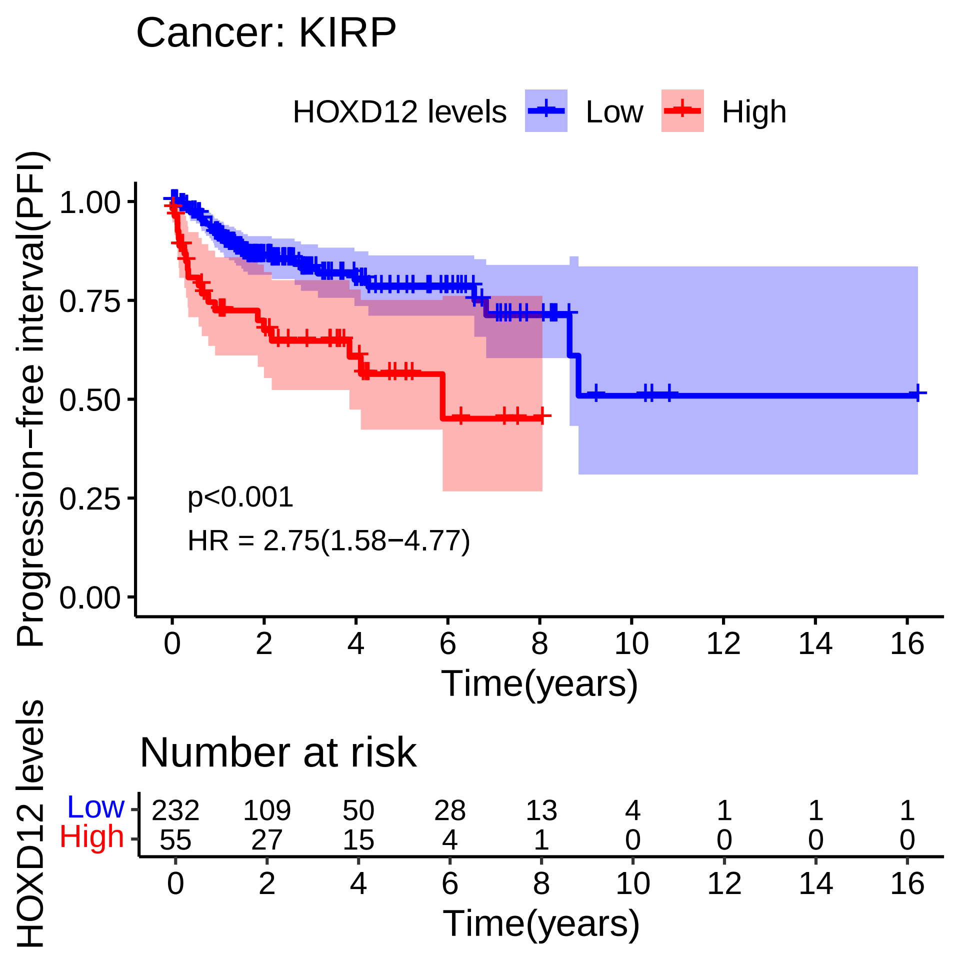
Cancer: KIRP
HOXD12 levels
Low
High
Progression−free interval(PFI)
1.00
0.75
0.50
0.25
0.00
p<0.001
HR = 2.75(1.58−4.77)
0
2
4
6
8
10
12
14
16
Time(years)
Number at risk
HOXD12 levels
Low 232 109 50 28 13 4 1 1 1
High 55 27 15 4 1 0 0 0 0
Time(years)

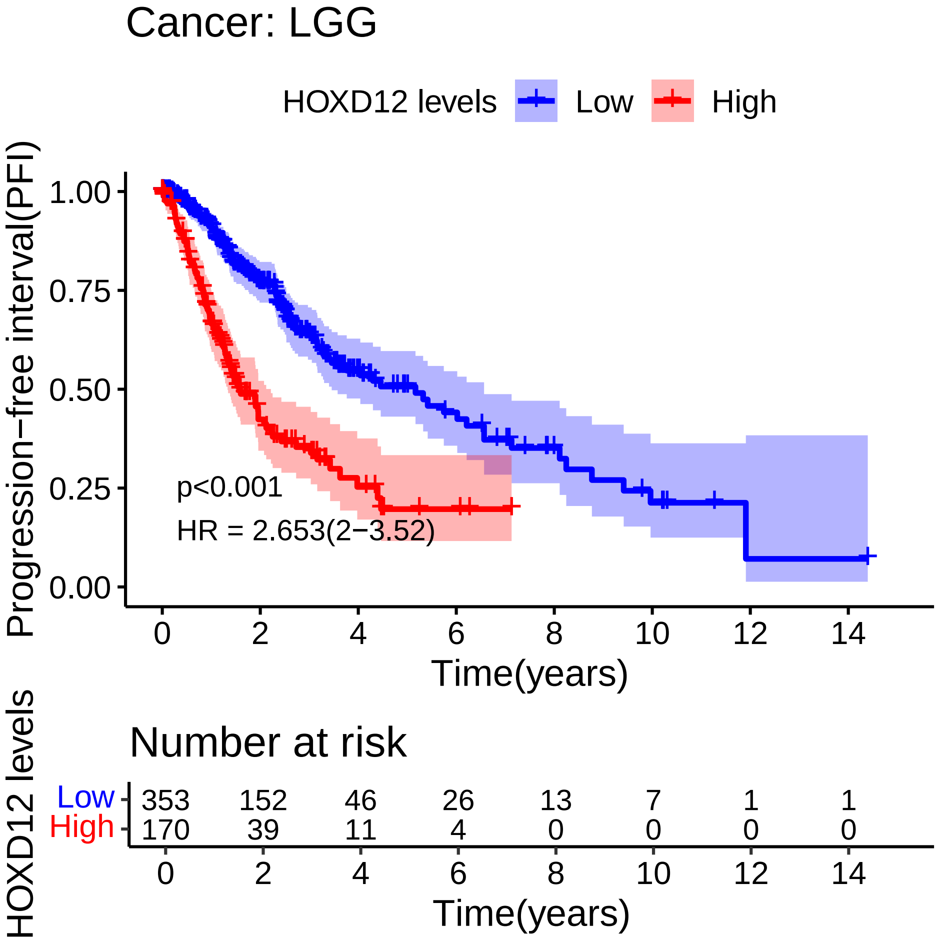
Cancer: LGG
HOXD12 levels
Low
High
Progression−free interval(PFI)
1.00
0.75
0.50
0.25
0.00
p<0.001
HR = 2.653(2−3.52)
0
2
4
6
8
10
12
14
Time(years)
Number at risk
HOXD12 levels
Low 353 152 46 26 13 7 1 1
High 170 39 11 4 0 0 0 0
0
2
4
6
8
10
12
14
Time(years)

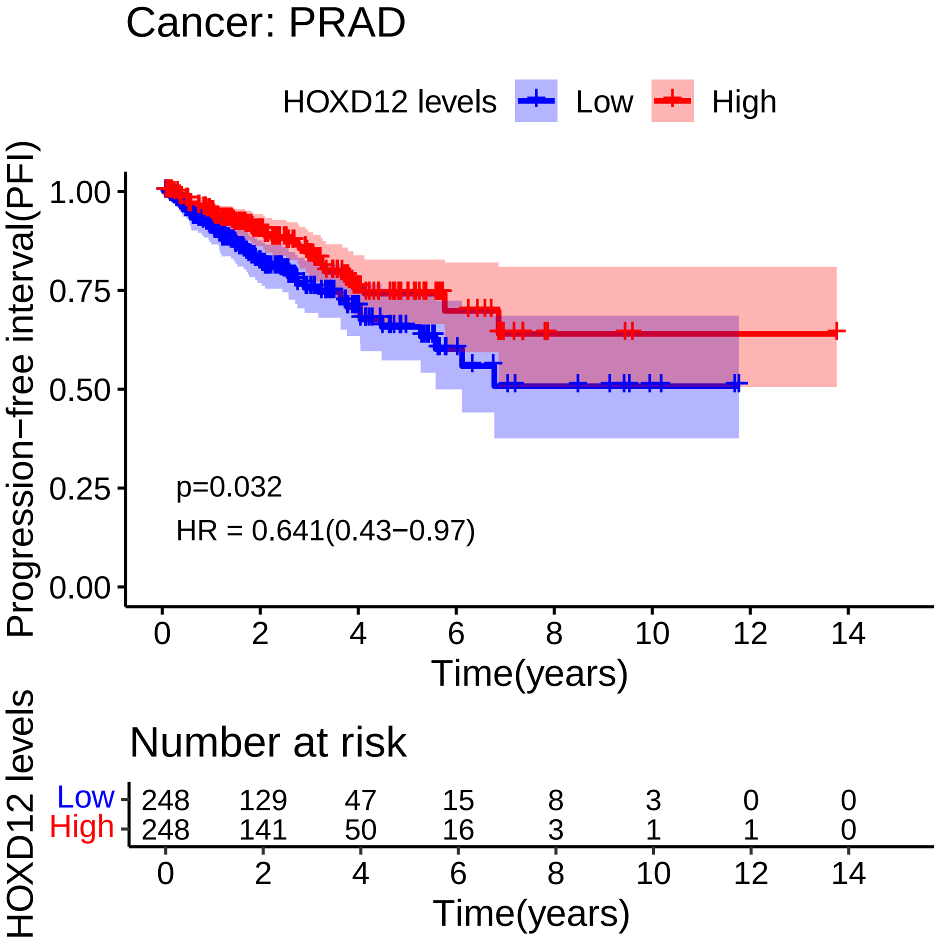
Cancer: PRAD
HOXD12 levels
Low
High
Progression−free interval(PFI)
1.00
0.75
0.50
0.25
0.00
p=0.032
HR = 0.641(0.43−0.97)
0
2
4
6
8
10
12
14
Time(years)
Number at risk
HOXD12 levels
Low 248 129 47 15 8 3 0 0
High 248 141 50 16 3 1 1 0
Time(years)

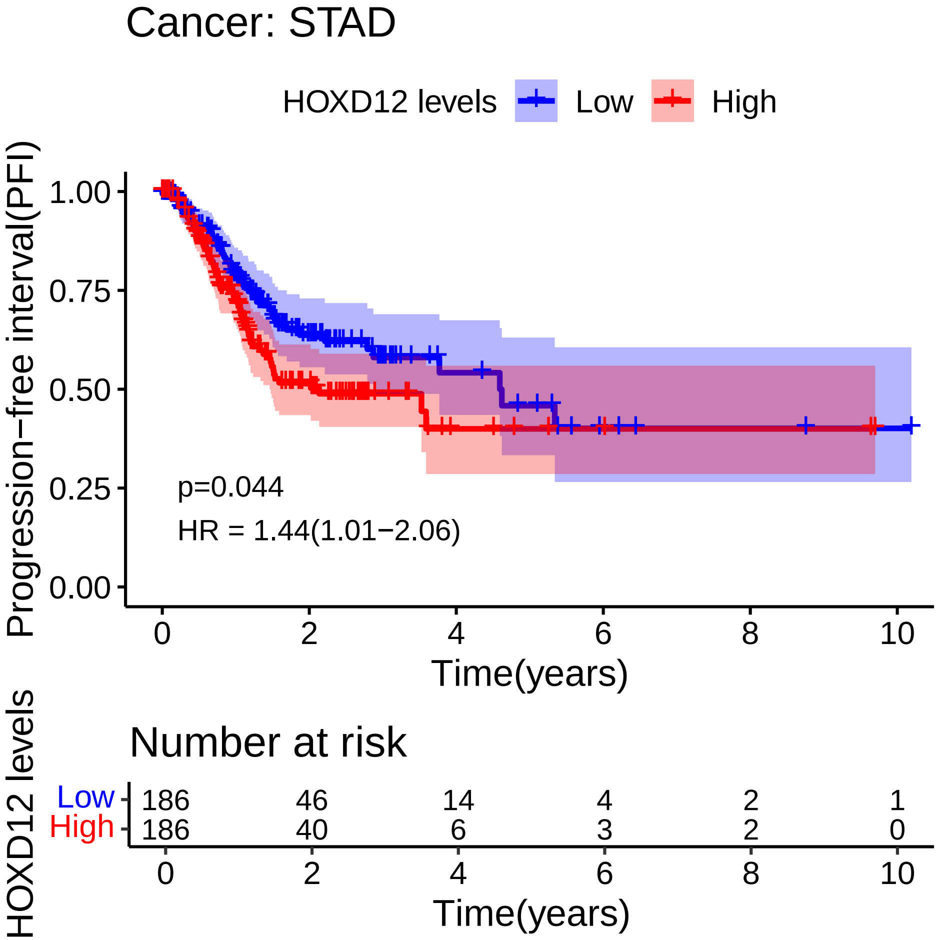

Cancer: STAD
HOXD12 levels
Low
High
Progression−free interval(PFI)
1.00
0.75
0.50
0.25
0.00
p=0.044
HR = 1.44(1.01−2.06)
0
2
4
6
8
10
Time(years)
Number at risk
HOXD12 levels
Low 186 46 14 4 2 1
High 186 40 6 3 2 0
Time(years)

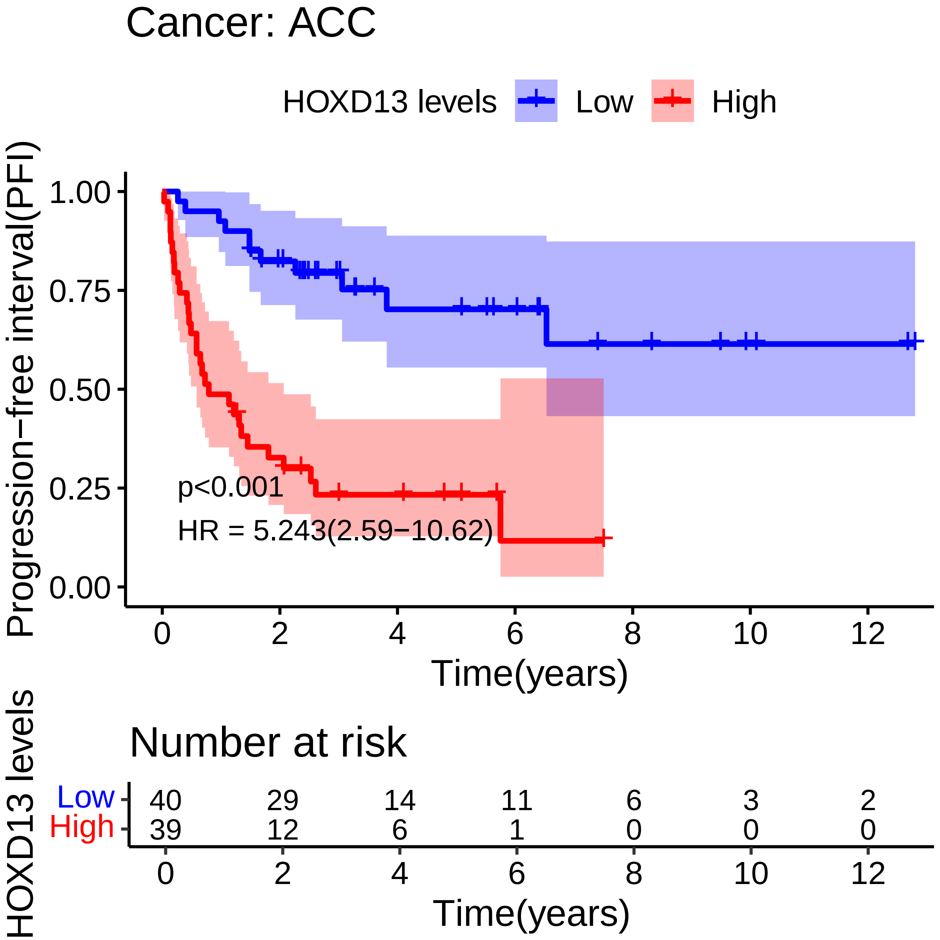

Cancer: ACC
HOXD13 levels
Low
High
Progression−free interval(PFI)
1.00
0.75
0.50
0.25
0.00
p<0.001
HR = 5.243(2.59−10.62)
0
2
4
6
8
10
12
Time(years)
Number at risk
HOXD13 levels
Low 40 29 14 11 6 3 2
High 39 12 6 1 0 0 0
Time(years)

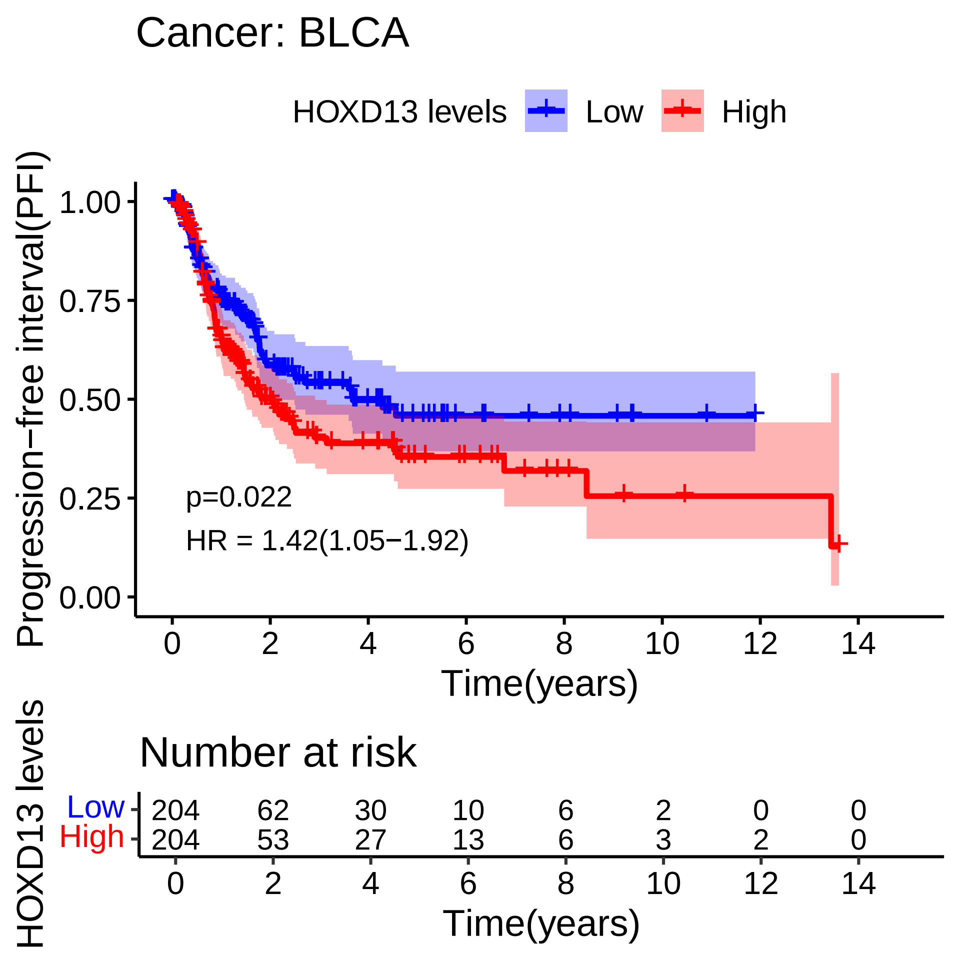

Cancer: BLCA
HOXD13 levels
Low
High
Progression−free interval(PFI)
1.00
0.75
0.50
0.25
0.00
p=0.022
HR = 1.42(1.05−1.92)
0
2
4
6
8
10
12
14
Time(years)
Number at risk
HOXD13 levels
Low 204 62 30 10 6 2 0 0
High 204 53 27 13 6 3 2 0
0
2
4
6
8
10
12
14
Time(years)

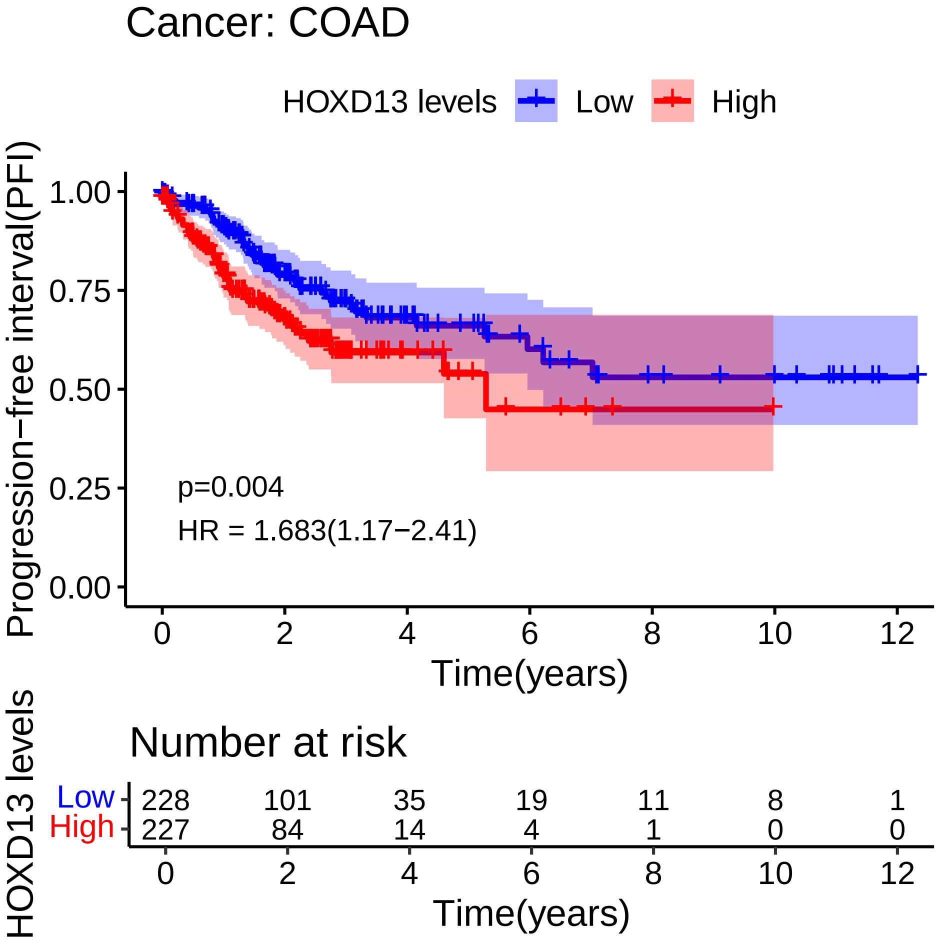

Cancer: COAD
HOXD13 levels
Low
High
Progression−free interval(PFI)
1.00
0.75
0.50
0.25
0.00
p=0.004
HR = 1.683(1.17−2.41)
0
2
4
6
8
10
12
Time(years)
Number at risk
HOXD13 levels
Low
High
228 101 35 19 11 8 1
227 84 14 4 1 0 0
Time(years)

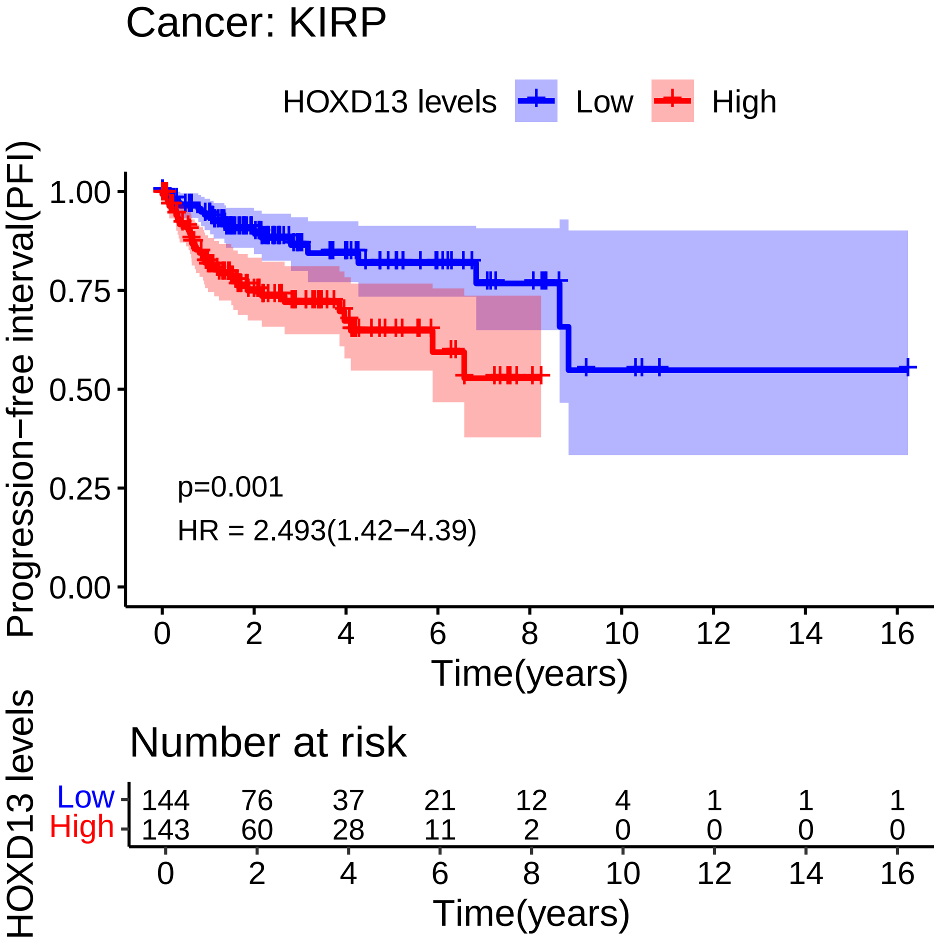

Cancer: KIRP
HOXD13 levels
Low
High
Progression−free interval(PFI)
1.00
0.75
0.50
0.25
0.00
p=0.001
HR = 2.493(1.42−4.39)
0
2
4
6
8
10
12
14
16
Time(years)
Number at risk
HOXD13 levels
Low 144 76 37 21 12 4 1 1 1
High 143 60 28 11 2 0 0 0 0
Time(years)

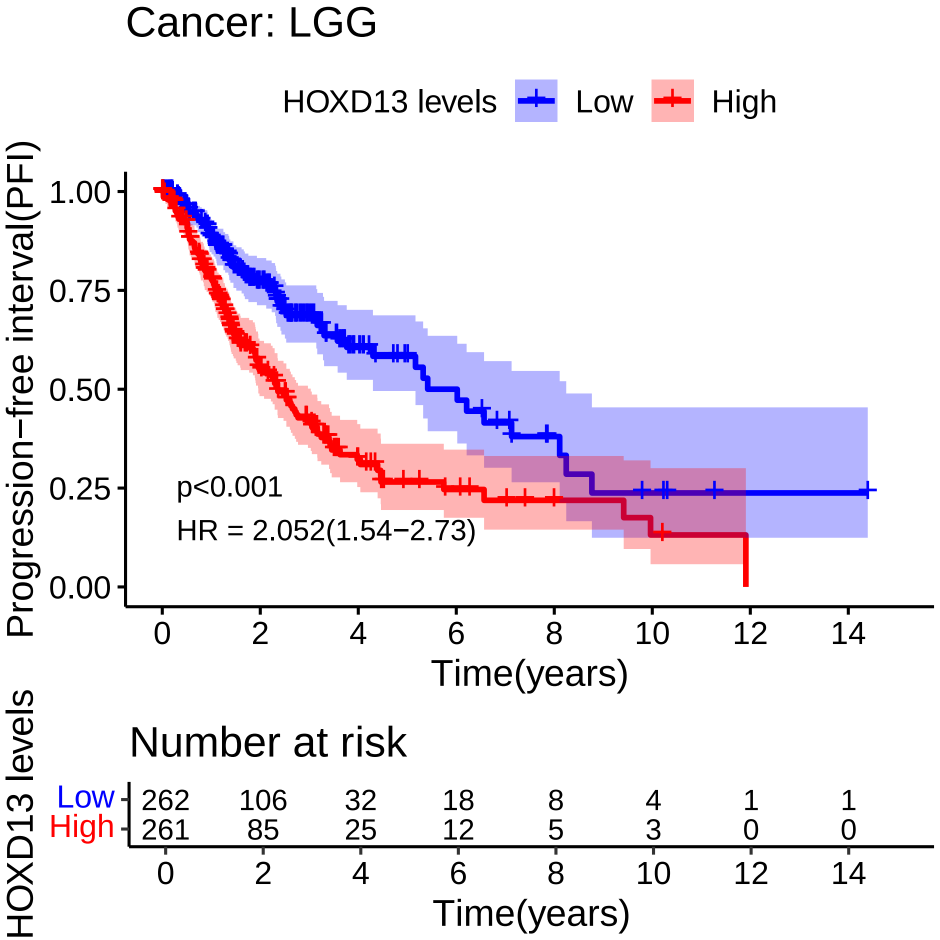
Cancer: LGG
HOXD13 levels
Low
High
Progression−free interval(PFI)
1.00
0.75
0.50
0.25
0.00
p<0.001
HR = 2.052(1.54−2.73)
0
2
4
6
8
10
12
14
Time(years)
Number at risk
HOXD13 levels
Low 262 106 32 18 8 4 1 1
High 261 85 25 12 5 3 0 0
0
2
4
6
8
10
12
14
Time(years)

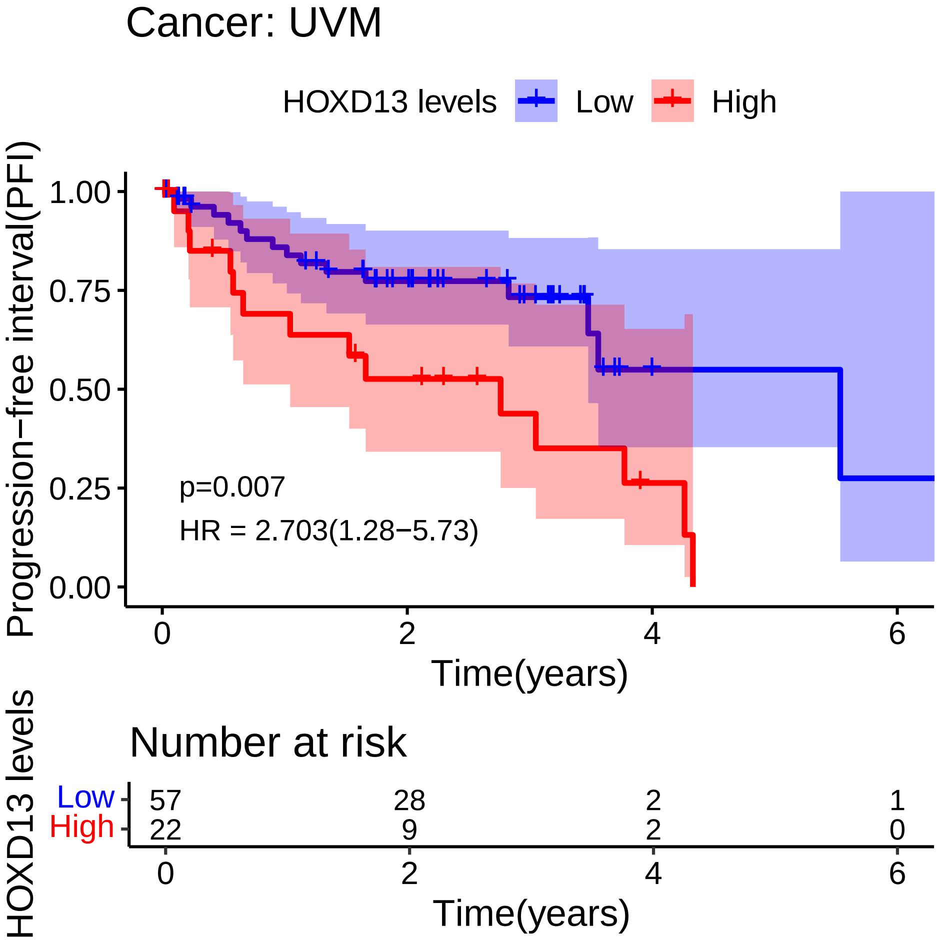

Cancer: UVM
HOXD13 levels
Low
High
Progression−free interval(PFI)
1.00
0.75
0.50
0.25
0.00
p=0.007
HR = 2.703(1.28−5.73)
0
2
4
6
Time(years)
Number at risk
HOXD13 levels
Low
High
57
28
2
1
22
9
2
0
0
2
4
6
Time(years)
