# Supplementary material for: A pan-cancer analysis of homeobox family: expression characteristics and latent significance in prognosis and immune microenvironment
Source: Front Oncol. 2025 Feb 6;15:1521652. doi: 10.3389/fonc.2025.1521652 (PMC11840236; doi:10.3389/fonc.2025.1521652)
Supplement: Supplementary Figure 1 — Mutations of each HOX gene in 32 cancers (TCGA, PanCancer Atlas) by cBioportal. [file DataSheet1.zip › Suppl.files/Supplementary methods.docx]

**Supplementary methods**

**Data Acquisition**

Expression data, survival data, and phenotypic data for 33 types of cancer were all downloaded from the UCSC Xena. Based on the human GRCh38 genome data, Ensemble IDs were converted to symbol IDs before proceeding with further analysis.

**Genetic alteration analysis**

By using the cBioportal database in April 2023 (https://www.cbioportal.org/), we inquired about the mutations of HOX family across 32 cancer studies (TCGA, Pan-Cancer Atlas), containing 10,967 samples total. Among them, mutation data, structural variants, and putative copy-number alterations (CNA) from GISTIC were chosen for genomic profiles, and the final result was displayed through the Oncoprint plate (showing the 'study of origin' and mutation of each HOX family gene). In addition, the Cancer Type Summary section showed the summary statistics of mutations in pan-cancer. Genetic alterations defined in cBioPortal are 'Inframe Mutation', 'Missense Mutation', 'Splice Mutation', 'Truncating Mutation', 'Structural Variant', 'Amplification', and 'Deep Deletion'. Different colors in Figures were used to indicate each of the 32 pan-cancer studies or genetic alterations.

**Expression Analysis of HOX Family**

All cancers’ HTSeq-FPKM gene expression RNAseq data from the UCSC Xena pan-cancer portal were downloaded for pan-cancer expression analysis and correlation analysis (https://xenabrowser.net/datapages/). Expression data has been log2(FPKM+1) converted. The expression of the HOX gene family in each sample was extracted using the 'limma' package, and corresponding boxplot graphs were plotted to display the expression patterns of the HOX gene family in different cancers. The results of differential expression analysis for each HOX gene between normal and tumor tissues in cancers with normal samples of not less than 5 were visualized using the 'ggpubr' package. Wilcoxon rank test was used to test the significance level. Pan-cancer expression heatmaps are plotted by the R package 'pheatmap', and it also performs clustering and column normalization, only showing cancers with normal samples of not less than 5. Besides, the correlation matrix based on HOX subgroups were calculated by Spearman's test and the 'cor.test' function in R package 'corrplot' was utilized to evaluate the statistical significance of the correlation between HOX genes.

**Univariate Cox Regression Analysis and Survival Analysis**

Survival data for 33 cancer types was also downloaded from UCSC Xena. Using the median expression of each gene as the cutoff value, the high and low expression groups of HOX genes were compared through Kaplan-Meier survival curve analysis, including overall survival (OS), disease-specific survival (DSS), progression-free interval (PFI). The proportional hazards assumption was tested using the 'survival' package, followed by the fitting of a univariate Cox regression model. The hazard ratios (HR) derived from univariate Cox regression analysis are graphically represented through a dot plot, facilitating a more nuanced interpretation of the relative risks associated with each HOX gene. The survival analysis was carried out by Cox proportional hazards regression using the function coxph() from the 'survival' package. Kaplan-Meier survival plots with p-values and HRs were visualized using the 'survminer' and 'ggplot2' packages. Only survival curves with p-values < 0.05 could be visualized.

**Correlation Analysis Between HOX and Tumor Microenvironment**

Data relevant to immune subtypes, tumor mutation burden (VarScan2 Variant Aggregation and Masking from Somatic Mutation), and tumor stemness were downloaded from UCSC Xena for further analysis. The spearman correlation between the HOX family and immune cell infiltration levels in pan-cancer was calculated using CIBERSORT. Six immune subtypes were employed for calculating correlations with HOX family expression levels by Kruskal-Wallis test. The R package 'estimate' was used to examine the stromal score, immune score, and estimate score in the immune microenvironment, which can reflect the stromal and immune cell infiltration levels of each cancer. Additionally, we compute the Spearman correlation between HOX expression and these scores, and the outcomes are shown as a heatmap. Additionally, a perl script was used to generate the tumor mutation burden score (TMB) for each sample. Based on somatic mutation data obtained from UCSC Xena (https://tcga.xenahubs.net), Microsatellite instability (MSI) scores were calculated for each sample. The Spearman correlation between TMB, MSI, DNA stemness score (DNAss), RNA stemness score (RNAss), and HOX expression were also examined. The radar chart of the Xiantao tool (https://www.xiantaozi.com/) shows the relationship between TMB, MSI, and the expression of HOXB7 and HOXC6. The correlation between the expression levels of HOXB7 and HOXC6 and the infiltration levels of 22 kinds of immune cells in LUAD were evaluated by 'CIBERSORT.R' and R-package 'ggplot2', 'ggpubr' and 'ggExtra'. The correlation analysis of TMB, MSI, DNAss, and RNAss with gene expression was all conducted using the Spearman correlation test. The P-values were generated by the cor.test function to represent statistical significance.

**The HOX score and Connectivity map analysis**

The HOX score was calculated by z-normalized expression for the 39 HOX genes across all of the samples within each cancer type. The HOX score per sample was determined by calculating the mean value across the 39 HOX genes, which represents a relative and overall estimate of the HOX family. In light of the previous results, we next calculate the spearman correlation between HOX score and mRNA expression of all coding genes in each sample. For each cancer type, we identified the top 150 most positively and negatively correlated protein-coding genes with valid gene symbols in the database, which were then curated as input for the 'Query' module of the Connectivity Map 1.0 (http://clue.io). Query parameters were set 'gene expression(L1000)'. Subsequently, the most relevant compounds (type: cp) for each of the 33 cancers were obtained. Finally, compounds with enrichment scores > 90 or < -90 in at least eight cancer types were selected for visualization to elucidate their potential therapeutic relevance.

**Protein–Protein Interaction Network Construction and functional enrichment**

The STRING online database (https://string-db.org/) was used to build the protein-protein interaction (PPI) network. Gene symbol names (HOXB7 and HOXC6) were subjected to PPI analysis. The minimum interaction score was set to medium confidence (0.400), and max number of interactors to show of 1st and 2st shell were all no more than 60 and 70 interactors. Next, we output the analysis results to a TSV format file, chose top 100 interacting proteins, and they were imported into Cytoscape v3.7.2 for detailed processing and visualization. The cytoHubba plugin was used to perform network topology analysis and node centrality analysis to find its hub genes and sub-networks. Functional enrichment analysis including Gene ontology (GO) and Kyoto Encyclopedia of Genes and Genomes (KEGG) were performed using the R-packages 'limma', 'org.Hs.eg.db', 'clusterProfiler' and 'enrichplot' based on the top 100 node genes.

**Immunohistochemistry**

Three lung adenocarcinoma cases and corresponding adjacent normal tissues collected from the Department of Pathology, Beijing Ditan Hospital, Capital Medical University were used to verify the expression of HOXB7 and HOXC6 by using immunohistochemical staining (IHC), respectively. The slides performed a series of procedures, including deparaffinization, antigen retrieval solution for antigen restoration, 3% hydrogen peroxide to suppress endogenous peroxidase activity, and goat serum to minimize non-specific staining. Subsequently, HOXB7 rabbit polyclonal antibody (bs-17364R, 1:100, Bioss Antibodies, USA) and HOXC6 (PA4010, 1:100, Abmart, China) covered the slides respectively, and they were left at 4°C for the whole night. The next day, sheep anti-rabbit IgG polymer (PV-6000, Zhongshan Jinqiao Biotechnology Company, Beijing, China) was added for 30 minutes at room temperature, and then DAB was added for three 3 minutes. Finally, they were counterstained with hematoxylin, dehydrated and sealed with neutral glue. All the images were taken under a 20x microscope.

**Cell culture**

The human embryonic kidney cell line 293T (HEK293T), the human lung cancer A549 and NCI-H1975 cell lines originated from American Type Culture Collection (ATCC). Both A549 and NCI-H1975 are human lung adenocarcinoma cells. 293T and A549 were all cultured in DMEM (HyClone, Logan, UT, USA) supplemented with 10% Fetal Bovine Serum (FBS; ExCell Bio, China) and 1% Penicillin-Streptomycin (Yuchun Bio, Shanghai, China) at 37°C and 5% CO2. The NCI-H1975 cell line were maintained in RPMI-1640 (ZETA life, USA) containing the same 10% FBS and 1% Penicillin-Streptomycin.

**Construction of shRNA stably transfected cell line**

All target shRNA were designed by GPP Web Portal of Broad institute (https://portals.broadinstitute.org/gpp/public/gene/search_clones). The oligonucleotide primers were annealed and connected to the pLKO.1 vector to produce the final plasmid expressing the target shRNA, and the shRNA sequence was synthesized by Tsingke Biotech (China). The target plasmid and packaging plasmids (psPAX2 and pMD2.G) were co-transfected into 293T cells using LipofectamineTM 2000 reagent (Invitrogen, USA). After 48 hours, the virus supernatant was collected, concentrated, and stored at -80°C. NSCLC cells were then transfected with lentivirus carrying shRNA to generate HOXB7 or HOXC6 knockdown LUAD cells, respectively. A549 or NCI-H1975 cells were infected with packaged lentivirus and maintained with puromycin screening to establish stable transfection cell lines after 14 days. Oligo Sequences of shRNA were: shHOXB7:5'-CCGGCCTCACGGAAAGACAGATCAACTCGAGTTGATCTGTCTTTCCGTGAGGTTTTTG-3';shHOXC6:5'-CCGGGTGAGGCATTTCTCGACCTATCTCGAGATAGGTCGAGAAATGCCTCACTTTTTG-3';shControl:5'-CCGGGTTCTTGCGATTGTCTCTATTCTCGAGAATAGAGACAATCGCAAGAACTTTTTG-3'.

**Quantitative RT-PCR**

Total RNA was extracted using Trizol and One step qRT-PCR SYBR Green Kit (Vazyme Biotech Co., Nanjing, China) was used for Quantitative RT-PCR (qRT-PCR) assay. Ct values detected by Bio-Rad qRT-PCR were processed using GAPDH as an internal parameter. The 2-ΔΔCt approach was employed to calculate the comparative expression. GraphPad Prism (9.0.0 version) was used for visualization. Primers used were shown as follows: GAPDH-F:5'-CATGTTCGTCATGGGTGTGAACCA-3', GAPDH-R:5'-ATGGCATGGACTGTGGTCATGAGT-3';HOXB7-F:5'-TCCACATTACCGGGAGCC-3',HOXB7-R:5'-CTGGGAGCACTCTGGACG-3';HOXC6-F:5'-TGACATCTGGCTTGCGATTG-3'; HOXC6-R: 5'-GGCCCTCCAATCCGTCAG-3'.

**CCK8, colony formation assay and wound healing assay**

The shRNA-transfected A549 or NCI-H1975 cells were fully digested, inoculated into 96-well culture plates at 2×10³ cells per well, and incubated at 37°C for different days. The original culture medium was removed and replaced with 100 µL of fresh culture medium and 10 µL of working solution containing CCK-8 working solution (TargetMol, USA) in each well. Then, the cells were incubated at 37°C for 2 h, and the absorbance was measured using the CCK-8 at 450 nm on days 1, 3, 5, and 7, respectively. A549 or NCI-H1975 cells stably transfected with shcontrol, shHOXB7 and shHOXC6 were positioned in a new 6-well plate at a density of 2×10^3^, and cultured for 2 weeks as described above, during which the medium was changed every 3 days. Formed colonies were fixed in 4% paraformaldehyde for 20 minutes, washed with PBS, and stained with Crystalline Violet (Beyotime, Shanghai, China) for 15-20 minutes. Finally, washed 3 times by pure water and photographed. Evenly transfected A549 or NCI-H1975 cells were inoculated in 6-well plates at 6×10^5^ cells per well for wound healing assay. After the cells completely adhered to the 6-well plates, a straight line was drawn vertically in the center of each well with a sterile 20μL pipette tip to ensure that the cells on this line were scraped off, which was regarded as 0 hour and marked it. After washing off the cell debris with PBS, the cells were incubated for 48 h. The migration of the cells was observed at 0 h, 24 h and 48 h, respectively.

**Statistical analysis**

The statistical software R (v4.2.1) was applied to perform the statistical analysis. P-value < 0.05 (double-tailed test) is deemed statistically significant. *P<0.05; **P<0.01; ***P<0.001. For comparing two normally distributed variables, we employ the independent samples t-test to assess their differences; for non-normally distributed variables, the Wilcoxon rank-sum test is utilized. When comparing three or more variables, the Kruskal-Wallis test (a non-parametric approach) or one-way ANOVA (parametric approach) can be applied. The correlation between variables is determined by calculating Pearson and Spearman correlation coefficients. To evaluate the prognostic differences between two groups, we use the "survfit" function from the R package "Survminer". Furthermore, the Kaplan-Meier method is employed to plot survival curves, and the log-rank test is conducted to identify statistically significant differences. The qRT-PCR and CCK-8 assays employed the Student's t-test to evaluate the statistical significance of differences between two groups. Preprocessing of expression data and calculation of TMB were analyzed by Perl (v5.30.0).
