# Supplementary figures and images for: A pan-cancer analysis of homeobox family: expression characteristics and latent significance in prognosis and immune microenvironment
Source: Front Oncol. 2025 Feb 6;15:1521652. doi: 10.3389/fonc.2025.1521652 (PMC11840236; doi:10.3389/fonc.2025.1521652)

# HOXA1

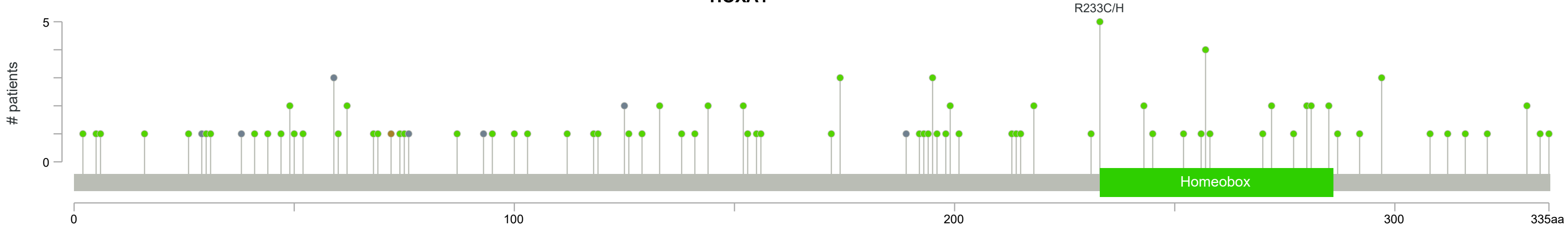

Supplement: Supplementary Figure 1 — Mutations of each HOX gene in 32 cancers (TCGA, PanCancer Atlas) by cBioportal. [file DataSheet1.zip › Suppl.files/S1_File.pdf]

# DNAss

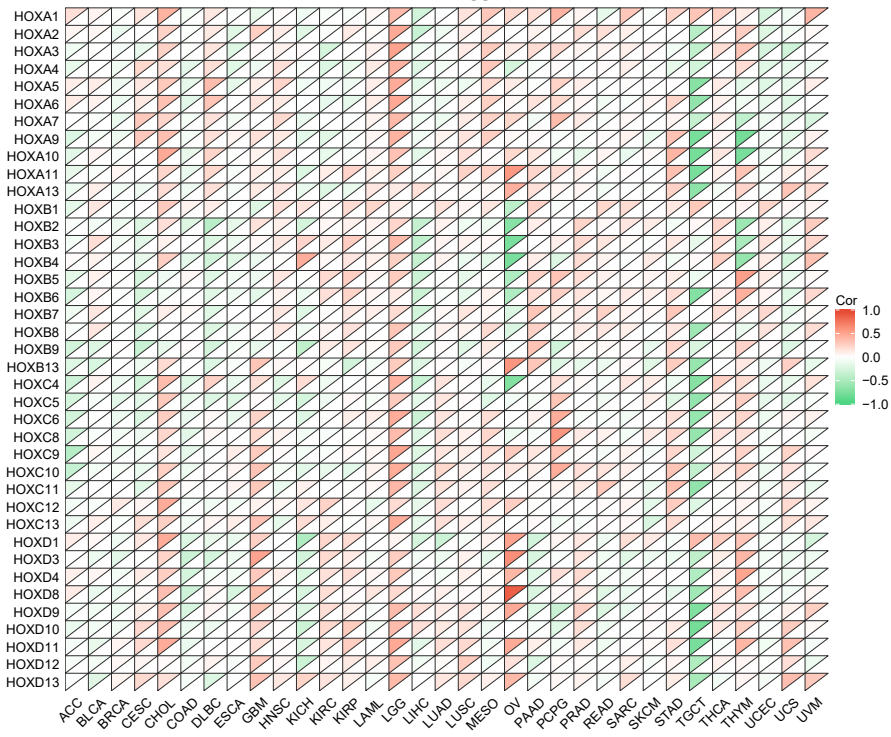

# RNAss

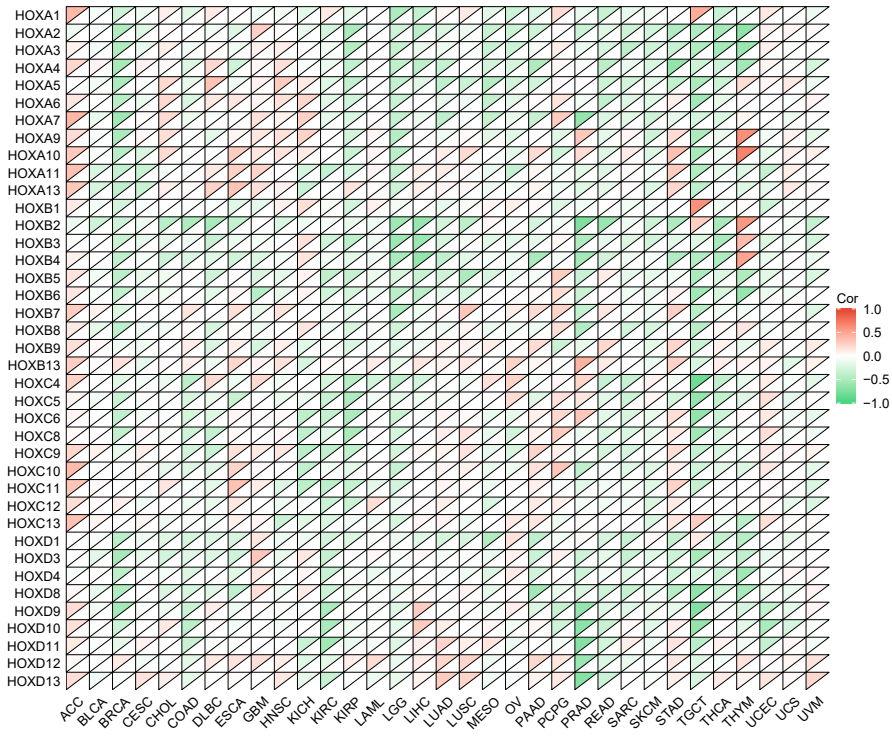

Supplement: Supplementary Figure 1 — Mutations of each HOX gene in 32 cancers (TCGA, PanCancer Atlas) by cBioportal. [file DataSheet1.zip › Suppl.files/S5_File.pdf]

**A**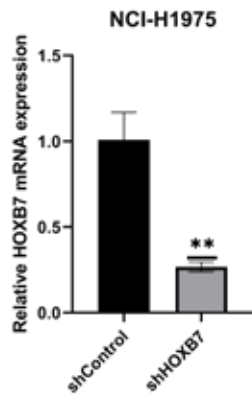**B**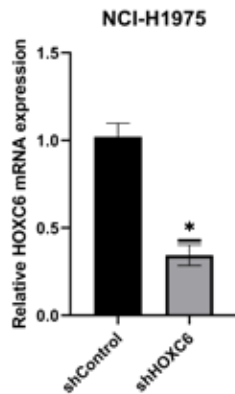**C**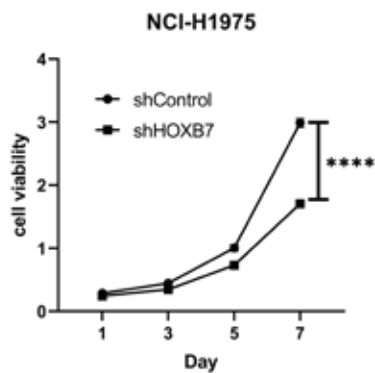**D**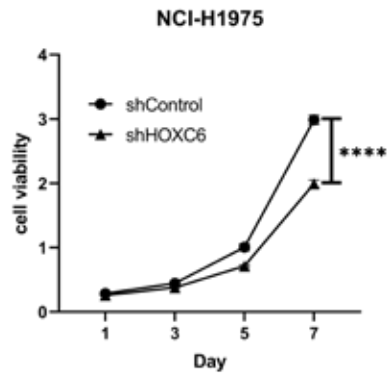**E****NCI-H1975**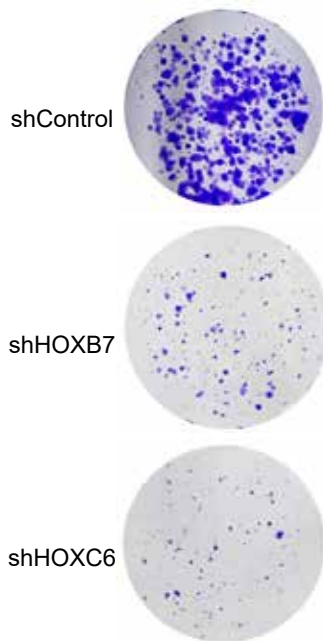**F****NCI-H1975**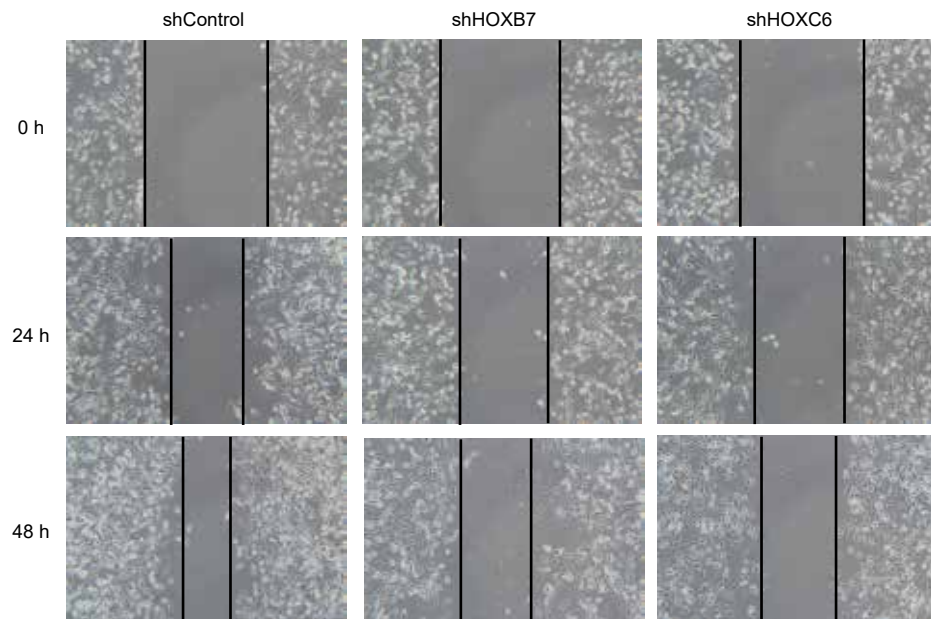

Supplement: Supplementary Figure 1 — Mutations of each HOX gene in 32 cancers (TCGA, PanCancer Atlas) by cBioportal. [file DataSheet1.zip › Suppl.files/S6_Fig.pdf]
